# Williams–Beuren syndrome shapes the gut microbiota metaproteome

**DOI:** 10.1038/s41598-023-46052-9

**Published:** 2023-11-03

**Authors:** Valeria Marzano, Stefano Levi Mortera, Pamela Vernocchi, Federica Del Chierico, Chiara Marangelo, Valerio Guarrasi, Simone Gardini, Maria Lisa Dentici, Rossella Capolino, Maria Cristina Digilio, Maddalena Di Donato, Iolanda Spasari, Maria Teresa Abreu, Bruno Dallapiccola, Lorenza Putignani

**Affiliations:** 1 Research Unit of Human Microbiome, Bambino Gesù Children’s Hospital, IRCCS, Rome, Italy; 2 GenomeUp s.r.l., Rome, Italy; 3 Genetics and Rare Diseases Research Division, Medical Genetics Department, Bambino Gesù Children’s Hospital, IRCCS, Rome, Italy; 4 Translational Cytogenomics Research Unit, Bambino Gesù Children’s Hospital, IRCCS, Rome, Italy; 5 Division of Digestive Health and Liver Diseases, Department of Medicine, Crohn’s and Colitis Center, University of Miami Miller School of Medicine, Miami, FL USA; 6 Scientific Directorate, Bambino Gesù Children’s Hospital, IRCCS, Rome, Italy; 7 Unit of Microbiomics and Research Unit of Human Microbiome, Bambino Gesù Children’s Hospital, IRCCS, Rome, Italy; 8 Present Address: Unit of Computer Systems and Bioinformatics, Department of Engineering, University Campus Bio-Medico of Rome, Rome, Italy

**Keywords:** Microbial communities, Proteomic analysis

## Abstract

Williams–Beuren syndrome (WBS) is a rare genetic neurodevelopmental disorder with multi-systemic manifestations. The evidence that most subjects with WBS face gastrointestinal (GI) comorbidities, have prompted us to carry out a metaproteomic investigation of their gut microbiota (GM) profile compared to age-matched healthy subjects (CTRLs). Metaproteomic analysis was carried out on fecal samples collected from 41 individuals with WBS, and compared with samples from 45 CTRLs. Stool were extracted for high yield in bacterial protein group (PG) content, trypsin-digested and analysed by nanoLiquid Chromatography-Mass Spectrometry. Label free quantification, taxonomic assignment by the lowest common ancestor (LCA) algorithm and functional annotations by COG and KEGG databases were performed. Data were statistically interpreted by multivariate and univariate analyses. A WBS GM functional dissimilarity respect to CTRLs, regardless age distribution, was reported. The alterations in function of WBSs GM was primarily based on bacterial pathways linked to carbohydrate transport and metabolism and energy production. Influence of diet, obesity, and GI symptoms was assessed, highlighting changes in GM biochemical patterns, according to WBS subsets’ stratification. The LCA-derived ecology unveiled WBS-related functionally active bacterial signatures: Bacteroidetes related to over-expressed PGs, and Firmicutes, specifically the specie *Faecalibacterium prausnitzii*, linked to under-expressed PGs, suggesting a depletion of beneficial bacteria. These new evidences on WBS gut dysbiosis may offer novel targets for tailored interventions.

## Introduction

Williams–Beuren syndrome (WBS, OMIM # 194050) is a rare genetic neurodevelopmental disorder with multi-systemic manifestations, with a prevalence of 1 in 7,500 live births as stated by a Norwegian epidemiological study^1^. The syndrome is caused by a de novo continuous hemizygous microdeletion (< 2-megabase pair) in the q11.23 region of the long arm of chromosome 7 spanning approximately 25–27 genes, described as the WBS critical region (WBSCR), and occurring during meiosis from recombination between misaligned repeat sequences flanking the critical region^2,3^.

WBS is associated with a constellation of clinical phenotypic appearances such as cardiac anomalies (mainly supravalvar aortic stenosis, SVAS; hypertension, elastin arteriopathy, peripheral pulmonary stenosis), a distintive facial dysmorphism, a specific neurobehavioral phenotype (*e.g*., mild-to-moderate intellectual disability, major impairment in visuospatial abilities, expressive language skills, hyper-sociable behaviour), connective tissue abnormalities, growth abnormalities, and endocrine anomalies^2–4^.

The relationships between the loss of each single WBSCR gene, or combination of them, and the WBS phenotypic manifestations are not clear; until now, only for some genes it is possible to establish a strong correlation. Among them, deletion or loss-of-function mutations of the gene *eln,* encoding the protein elastin^5^, account for some classical WBS features such as SVAS, cardiovascular manifestations, *cutis laxa*. Among other common clinical features, WBS patients report gastrointestinal (GI) disturbances including difficulties with feeding and swallowing, vomiting, gastroesophageal reflux, diverticular diseases, diverticulitis, celiac disease, chronic constipation, diarrhoea, malabsorption, diverticulosis/diverticulitis, and rectal prolapse^2,3,6–9^, which may be consequence of the impaired intestinal wall elasticity, associated to lack of a functional elastin and/or of eating habits with a low dietary fiber intake^2,3,9^. Therefore, we hypothesized that these GI symptoms could be related to an alteration of the gut microbiota (GM). In fact, GM alteration, or dysbiosis, is involved in the development of different GI and extra-intestinal pathologic conditions, and in many chronic diseases, such as autoimmune and allergic diseases, non-alcoholic fatty liver disease, inflammatory bowel diseases, obesity, diabetes^10–15^. Amongst the other functions, the GM is specialized in both collecting and storing energy and exerting a variety of metabolic functions, such as fermentation and up-take of undigested carbohydrates, synthesis, and absorption of bile acids, vitamins, amino acids, and short-chain fatty acids (SCFAs)^16^.

Therefore, in this study, we conducted a metaproteomic approach to unveil functional features of the GM profile in WBS patients compared to age-matched healthy subjects. Possible roles of altered molecular mechanisms and crucial bacterial signatures in the pathophysiology of WBS were assessed to pave the way for targeted approaches to improve health quality and clinical management.

## Results

### Subjects’ cohort and study design

Metaproteomic profiling of GM was performed on stool samples of 41 WBS patients (min 1–max 39 years, mean of 14 years ± 9 s.d., 17 males and 24 females, male gender frequency of 41%) compared to feces from 45 healthy subjects (controls, CTRLs) (aged 1–42 years, mean of 13 years ± 10 s.d.; 23 males and 22 females, male gender frequency of 51%), to evaluate GM ecology, microbial and functional signatures. Patients’ anthropometric, demographic and clinical data were recorded at the time of enrollment (Table 1 and Supplementary file 1).

**Table 1:** Demographic and clinical features of WBS patients.

Variable	Cases N	Mean ± s.d. or frequency
Age (years)	41	14 ± 9
Weight (kg)	41	44.5 ± 24.5
Height (cm)	41	137.1 ± 25.3
BMI (kg/m^2^)	41	21.61 ± 7.3
Female sex	24	59%
Facial dysmorphism	37	90%
Motor and/or cognitive impairments	27	66%
Cardiovascular abnormalities	23	56%
Hypertension	17	41%
Hypothyroidism	8	20%
Omnivorous diet	32	78%
Obesity	8	20%
GERD	10	24%
Constipation	10	24%
Diarrhea	6	15%
Abdominal pain	6	15%

*s.d.* standard deviation, *BMI* body mass index, *GERD* Gastroesophageal reflux disease.

WBS and CTRL sample groups showed no significant difference in distribution based on age and gender (*p* value = 0.4131 and 0.4954, respectively, Mann–Whitney test), neither in the number of females and males in each groups (WBS, *p* value = 0.3489 and CTRL, *p* value = 1.0000; Binomial two-tailed test).

The distribution of the subjects’ age was considered also by stratifying the subjects into four groups: namely, group 0, toddlerhood, 1 < years < 5 (5 WBSs; 5 CTRLs); group 1, childhood, 5 ≤ years < 13 (22 WBSs and 14 CTRLs); group 2, adolescence, 13 ≤ years < 18 (11 WBSs and 9 CTRLs); and group 3, adulthood, 18 ≤ years ≤ 42 (7 WBS and 13 CTRLs), evidencing no statistically differences between WBSs and CTRLs subgroups (Supplementary File 1).

### WBSs and CTRLs GM metaproteomes are similar in peptide and protein content

From WBS and CTRLs stool samples, 1,078.63 ± 550.82 µg (mean ± s.d.) and 973.14 ± 437.12 µg of proteins were extracted, respectively (Supplementary Figure 1.A), while after enzymatic digestion of 50 µg, 40.86 ± 11.80 µg and 42.18 ± 16.47 µg of peptides were obtained for each of the two groups (Supplementary Figure 1.B).

The nLC-ESI–MS/MS procedure yielded a total of 33,952 proteins with 128,829 unique peptide sequences, corresponding to an average of 1,697 ± 555 bacterial Protein Groups (PGs) identified by 13,189 ± 3921 unique peptides and 48 ± 13 human PGs, associated to 705 ± 170 unique peptides per sample. After the filtering and pre-processing steps, 638 bacterial PGs and 42 human PGs (Supplementary File 2 and 3, respectively) were retained.

### The GM metaproteome of WBSs is less heterogeneous than that of CTRLs considering age variable

**Figure 1:**
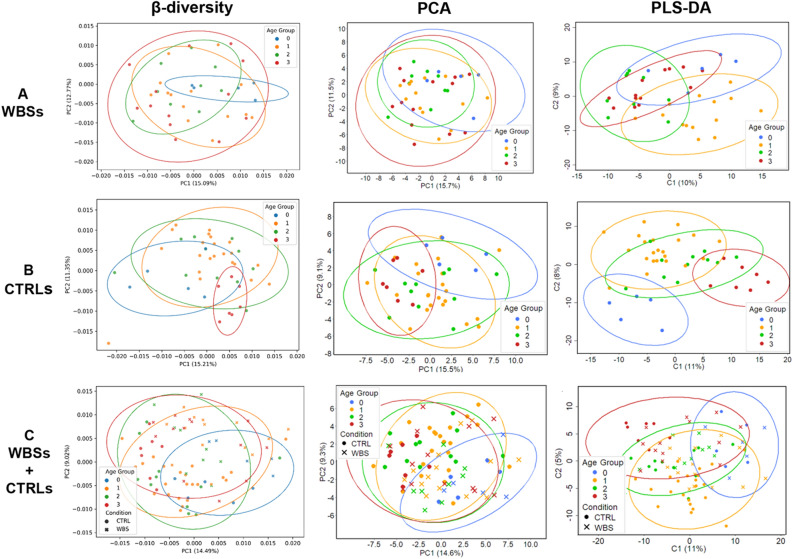
Analyses of bacterial protein content from stool samples of WBSs and age-matched CTRLs, based on age distribution. Bray–Curtis β-diversity, unsupervised Principal Component Analysis (PCA) and supervised Partial Least Squares-Discriminant Analysis (PLS-DA) were carried out separately on WBSs (**A**), CTRLs (**B**), and on the entire dataset of WBSs + CTRLs (**C**). The variances explained by each component are stated in brackets. Color code: blue, age group 0, < 5 years; orange, age group 1, 5 ≤ years < 13; green, age group 2, 13 ≤ years < 18; red, age group 3, 18 ≤ years ≤ 42.

Because of the extended age range of WBSs and CTRLs, its influence on bacteria PGs’ abundance distribution was considered by stratifying subjects into the four age groups 0–1–2–3. WBS, CTRL data sets and WBS + CTRL whole data set did not show statistically significant differences, as inferred by β-diversity applied on Label Free Quantification (LFQ) bacteria PGs’ matrix (PERMANOVA test *p* value = 0.2342, 0.4374 and 0.3373) (Fig. 1). However, both Principal Component Analysis (PCA) and Partial Least Squares-Discriminant Analysis (PLS-DA) showed for the CTRLs dataset that the age-related ellipses were more distant each other, compared to WBSs, with special reference to early childhood (group 0) and adulthood (group 3) (Fig. 1). In particular, for the PGs prevalently contributing to the PC1 projection of the PCA, MANOVA analysis showed a statistically significant difference for only CTRL and whole WBS + CTRL data sets (*p* value = 0.0249 and 0.0088, respectively), but not for the WBS set (*p* value = 0.1806) (Supplementary File 4).

Furthermore, to analyze how the averaged intensities of bacteria PGs differed amongst and within WBSs and CTRLs groups, with respect to age, univariate analysis was also performed. Indeed, while WBSs showed differences only between group 0 and 1 (Mann–Whitney test *p* value = 0.0221), all comparisons amongst CTRLs age groups revealed statistically significant differences (*p* values < 0.05).

Comparing the age groups within the whole dataset of WBSs + CTRLs or the age groups between WBSs and CTRLs groups, differences resulted always statistically significant except for the comparison of group 2 (teens) *vs.* group 1 (toddlers) in WBS + CTRL dataset and only for the group 2 in the WBSs *vs*. CTRLs matching (Supplementary File 4).

The absence of overall statistical significance amongst WBSs age stratified subsets suggested that the WBS disease phenotype plays a significant role in shaping the functional signatures of GM and led us to investigate the differentially expressed PGs between all WBS samples compared to all CTRLs without taking into consideration the age distribution.

### WBS GM metaproteome has a specific disease related-pattern

**Figure 2:**
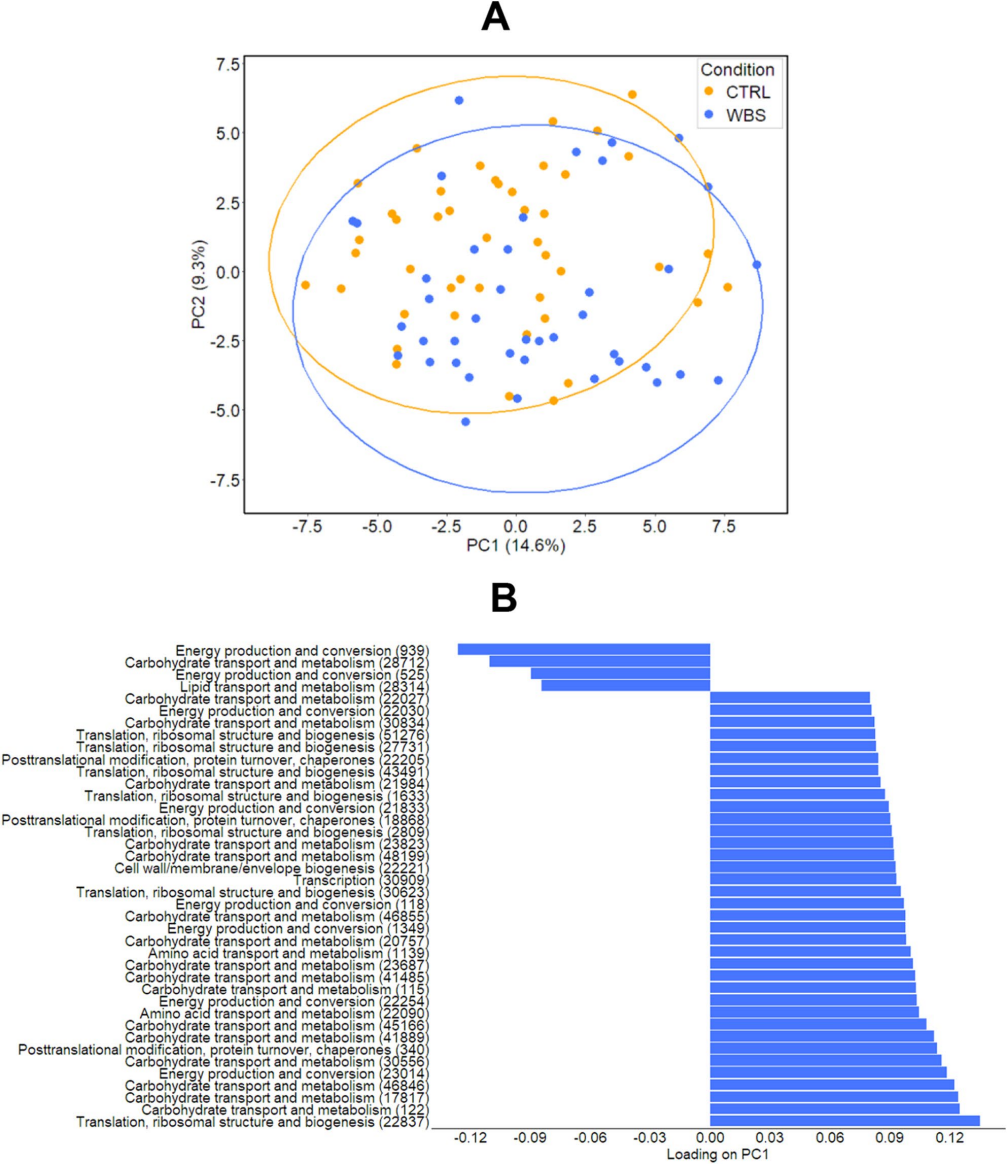
Analyses of identified bacteria protein groups (PGs) from stool samples of patients (WBSs, blue colour) and age-matched healthy subjects (CTRLs, orange colour). (**A**) Unsupervised Principal Component Analysis shows a segregation between WBS and CTRL groups. The variances explained by each component are stated in brackets. (**B**) The top forty PGs contributing of PC1 loadings are displayed with their corresponding COG category and ID in brackets.

Beta-diversity applied on bacteria PGs’ abundance distribution in WBSs and CTRLs showed statistically significant differences (*p* value < 0.001) (Supplementary Figure 2.A). PCA analysis, performed on the same matrix, displayed a slightly separation between WBSs and CTRLs (Fig. 2A), while MANOVA test, performed by including the top 40 PGs contributing to PC1 loadings, revealed statistically significant difference (*p* value = 0.0320). These PGs were mainly associated to Carbohydrate transport and metabolism [G] (17 PGs), Energy production and conversion [C] (8 PGs), Translation, ribosomal structure and biogenesis [J] (7 PGs) and Posttranslational modification, protein turnover, chaperones [O] (3 PGs) COG categories (Fig. 2B and Supplementary File 2). The associated KEGGs were Glycolysis/Gluconeogenesis (11 PGs), Ribosome (5 PGs), Pentose phosphate pathway (4 PGs), ABC transporters (4 PGs), and Pyruvate metabolism (3 PGs) (Supplementary File 2). Also, the PLS-DA algorithm showed a slightly separation between WBSs and CTRLs (Supplementary Figure 2.B). The 21 most important PGs contributing to the separation model, with Variable importance in projection (VIP) scores > 2.0, were associated to C (5 PGs), J (4 PGs); and G (3 PGs) COGs. (Supplementary File 2). Therefore, both PCA and PLS-DA highlighted distinctive WBS-related functional signatures, likely reflecting a switching of WBSs metabolic activities towards carbohydrate transport and metabolism and energy pathways.

Moreover, WBSs and CTRLs showed also statistically significant difference in the average total PG’ intensities (Mann–Whitney test *p* value < 0.001) assessed by univariate analysis. Eighty-nine differentially abundant PGs were found in WBSs compared to CTRLs, of which 83 had an assigned COG name, 62 a KEGG name and 86 a taxonomic assignment, for a total of 60 different COG and 25 KEGG names and 34 unique taxa defined by LCA algorithm (Supplementary File 2 and Supplementary Figure 3).

Seventeen PGs (19.10%) were assigned to Actinobacteria, represented by a prevalence of Coriobacteriaceae and Bifidobacteriaceae; 16 PGs (17.98%) accounted to Bacteroidetes, with an almost complete prevalence of Bacteroidaceae; and 53 PGs (59.55%) were Firmicutes with a prevalence of assigned Ruminococcaceae. Overall, 43 PGs were over-expressed and 46 were under-expressed in WBSs respect to CTRLs. The over-expressed PGs were attributable for 25.58% (11 PGs) to Actinobacteria, for 37.21% (16 PGs) to Bacteroidetes and for 30.23% (13 PGs) to Firmicutes; the under-expressed PGs were related for 13.04% (6 PGs) to Actinobacteria and the for the 86.96% (40 PGs) to Firmicutes, mainly Clostridiales (38 PGs), Ruminococcaceae (17 PGs), until *Faecalibacterium prausnitzii* (7 PGs) and *Subdoligranulum variabile* (8 PGs) species (Table 2 and Supplementary File 2)*.*

**Table 2:** Association of differentially expressed bacteria protein groups to taxonomy.

N PGs and type of expression	Phylum (L2)	Distribution (%)	N PGs	Family (L5)	N PGs	Genus (L6)	N PGs
89 differentially expressed	Actinobacteria	19.10	17	Coriobacteriaceae	9	*Collinsella*	8
				Bifidobacteriaceae	7	*Bifidobacterium*	7
	Bacteroidetes	17.98	16	Bacteroidaceae	14	*Bacteroides*	14
	Firmicutes	59.55	53	Ruminococcaceae	21	*Faecalibacterium*	8
						*Ruminococcus*	4
						*Subdoligranulum*	8
43 over-expressed in WBSs	Actinobacteria	25.58	11	Coriobacteriaceae	8	*Collinsella*	8
				Bifidobacteriaceae	3	*Bifidobacterium*	3
	Bacteroidetes	37.21	16	Bacteroidaceae	14	*Bacteroides*	14
	Firmicutes	30.23	13	Ruminococcaceae	4	*Faecalibacterium*	1
						*Ruminococcus*	3
						*Subdoligranulum*	0
46 under-expressed in WBSs	Actinobacteria	13.04	6	Coriobacteriaceae	1	*Collinsella*	0
				Bifidobacteriaceae	4	*Bifidobacterium*	4
	Bacteroidetes	0.00	0	Bacteroidaceae	0	*Bacteroides*	0
	Firmicutes	86.96	40	Ruminococcaceae	17	*Faecalibacterium*	7
						*Ruminococcus*	1
						*Subdoligranulum*	8

Taxonomic node level: L2, Phylum; L5, Family; and L6, Genus.

**Figure 3:**
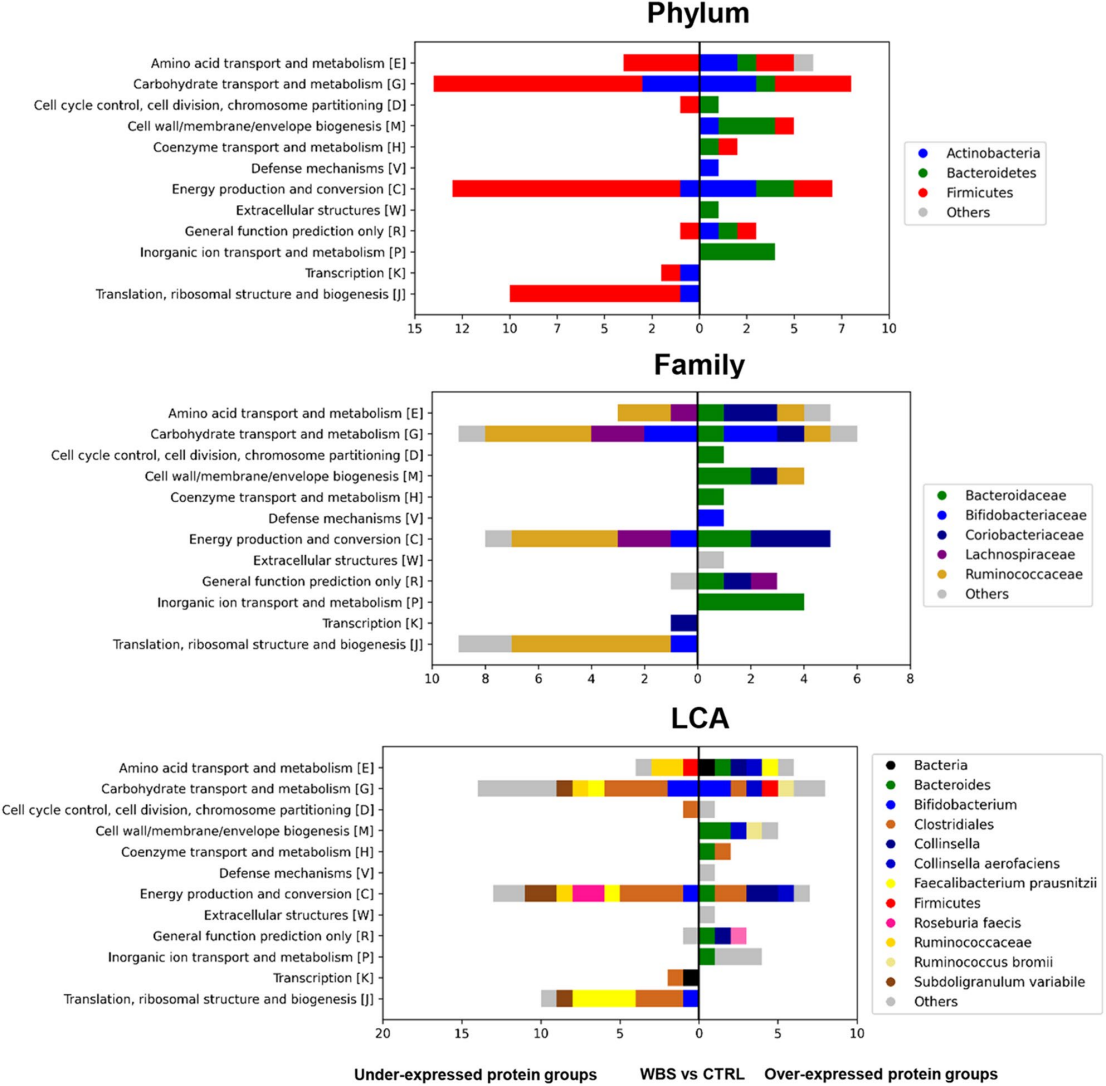
Graphical sketch of the modulated COG categories corresponding to the differentially expressed bacteria protein groups (PGs) comparing WBSs to CTRLs. Bars represent the number of over- and under-expressed PGs, associated with the respective functional annotation and with the Lowest common ancestor (LCA) algorithm outcome depicted by a colour code.

The functional annotation showed that differential expressed PGs in WBSs were mainly associated with Amino acid transport and metabolism [E], G, C, and J COG categories. The E, C, G categories as well as Cell cycle control, cell division, chromosome partitioning [D] and General function prediction only [R] were represented in both over- and under-expressed PGs. Noteworthy, Cell wall/membrane/envelope biogenesis [M], Coenzyme transport and metabolism [H], Defense mechanisms [V], Extracellular structures [W], and Inorganic ion transport and metabolism [P] COG categories were ascribed only to WBS over-expressed PGs. On the contrary two COG categories, namely Transcription [K] and J, were present only in over-expressed PGs of CTRLs (Fig. 3).

Finally, E, G, M C, K, and J COG categories, obtained from both univariate and multivariate approaches, provided the major contribution to the functional diverseness between WBSs and CTRLs GM metaproteome, with G and C accounting for the largest number of associated PGs (Supplementary Table 1).

Relating to KEGG pathways, a full description of under- and over-expressed PGs is reported in Supplementary Figure 4.

**Figure 4:**
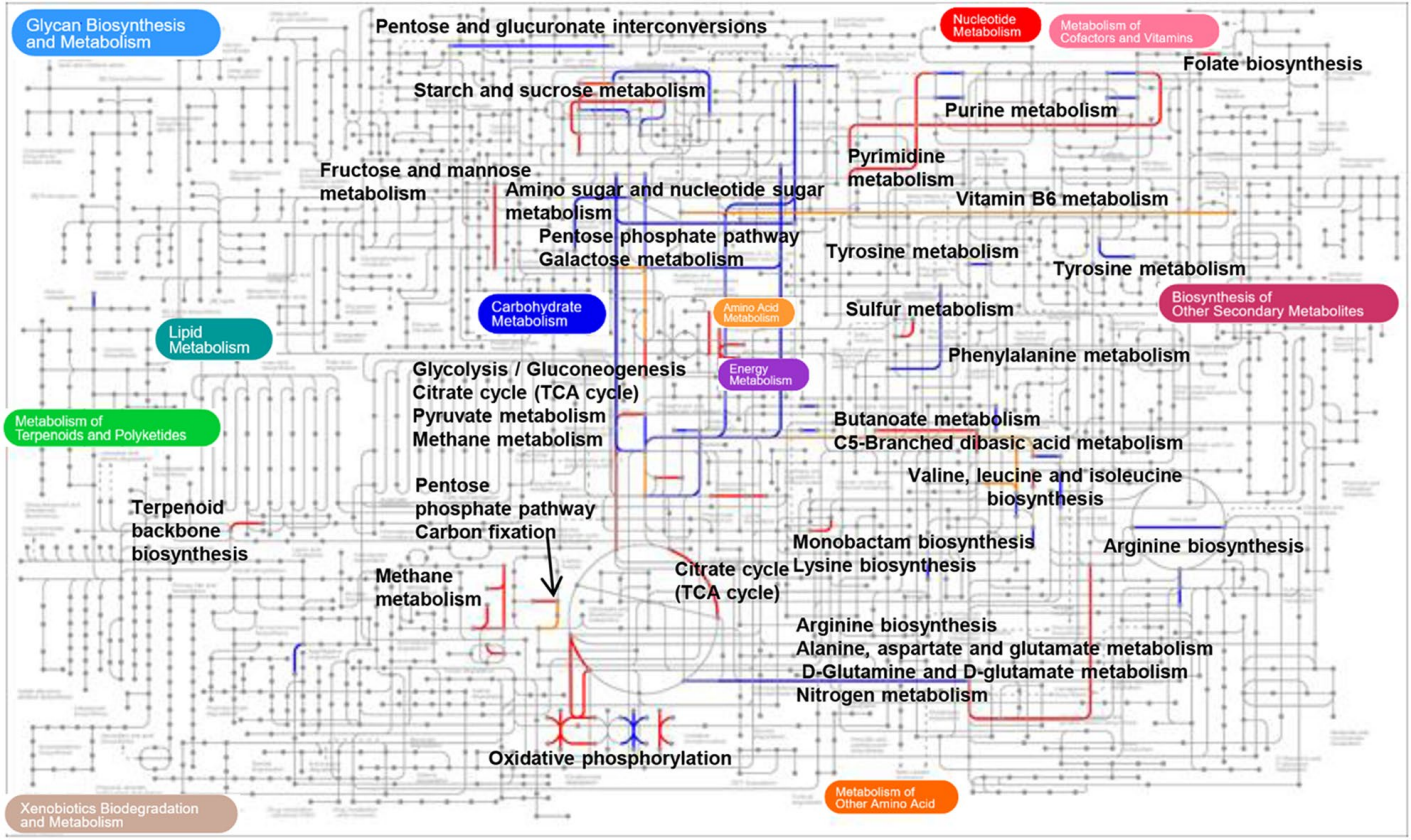
Image of the modulated metabolic networks corresponding to the differentially expressed bacteria protein groups (PGs) comparing WBSs to CTRLs. Blue, red and orange colors refer to metabolic reactions associated to COG accession of over-expressed, under-expressed and both over- and under-expressed PGs, respectively. Image modified from Interactive Pathways Explorer 3 (iPath3.0, https://pathways.embl.de/).

To obtain a comprehensive image of the WBSs GM-related biochemical activity, the differently expressed bacterial COGs were mapped in a complete metabolic pathways’ chart, highlighting carbohydrate, amino acid, energy, glycan, and terpenoid metabolisms as the major metabolic pathways of the gut metaproteome in WBS (Fig. 4).

### GM metaproteome of WBS patients in relation to diet, obesity and GI symptoms

To determine the influence of patients’ clinical data (Table 1 and Supplementary File 1) on GM metaproteome, distribution of LFQ bacteria PGs were investigated based on: nutritional habits (A: 32 patients who followed an omnivorous diet *vs*. 9 patients who did not follow such a diet); obesity status (B: 8 obese patients *vs*. 32 non-obese); and presence of functional GI disturbances such as GERD, constipation, diarrhea and abdominal pain, particularly presence of no GI symptom *vs.* one or more symptoms (C: 22 patients who presented at least one symptoms *vs*. 19 who did not). We analyzed the variation of WBS GM in relation to these three distinct characteristics, which have been shown to have a major impact on the GM metaproteome.

**Figure 5:**
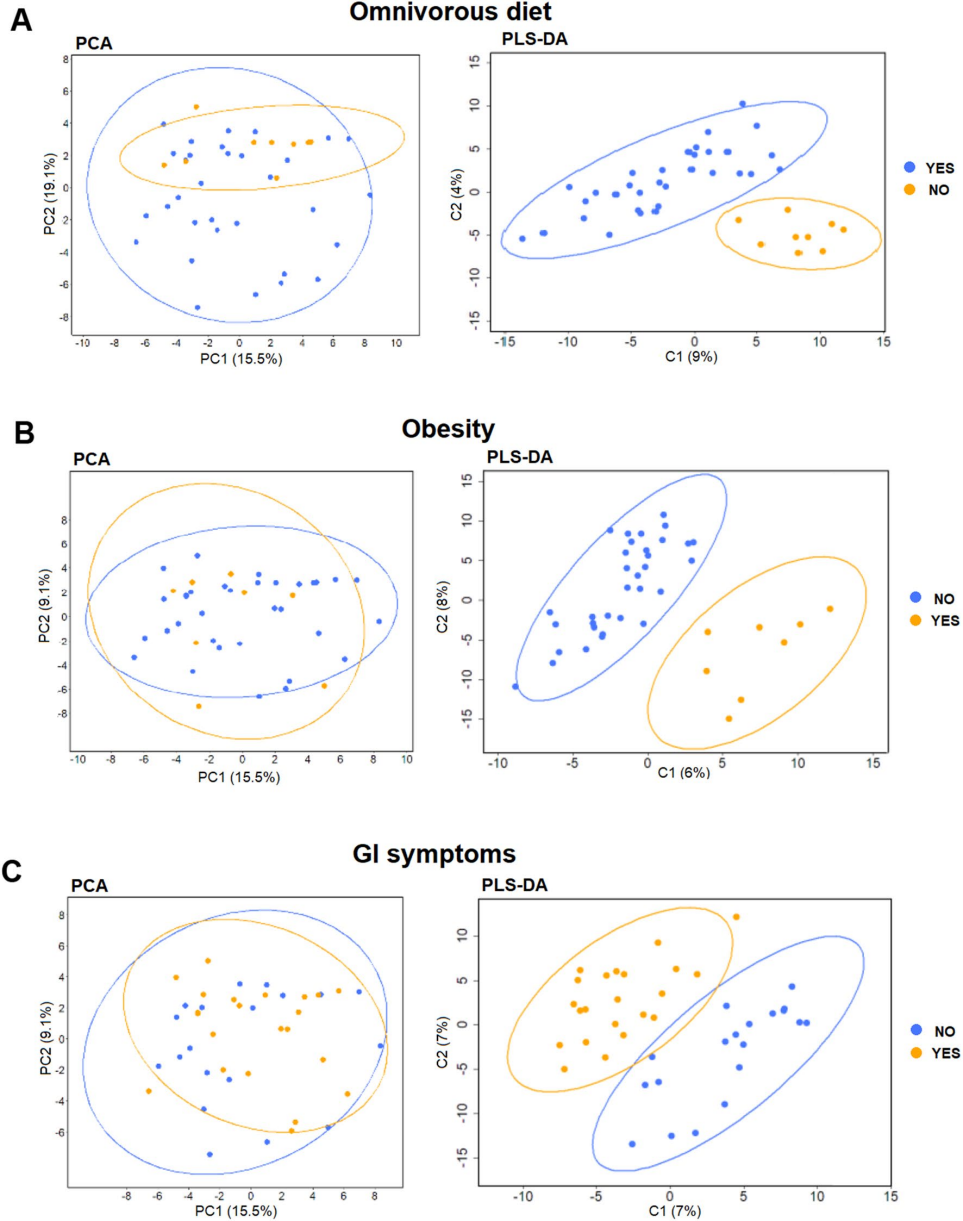
Analyses of identified bacteria from stool samples of patients based on protein content and clinical data. PCA and PLS-DA analyses were carried out separately stratifying samples by: (**A**) omnivorous diet (YES = patients who followed an omnivorous diet, blue color; NO = patients who did not follow such a diet, orange color); (**B**) obesity (NO = non-obese patients, blue color; YES = obese patients, orange color); and (**C**) gastrointestinal (GI) symptoms (NO = absence of symptoms, blue color, YES = presence of at least one symptom, orange color).

The three WBS groups, stratified on the bases of the above described clinical features, did not show statistically significant differences by β-diversity analysis (*p* value = 0.9760, 0.5325 and 0.6196) (Supplementary Figure 5) and MANOVA test. Compared to PCA, the pairwise PLS-DA representation showed a clear PGs clustering amongst WBS subgroups based on clinical features (Fig. 5). The univariate comparisons showed statistically significant difference in PGs’ averaged intensities only for the obesity status (Mann–Whitney *p* value = 0.0040).

T-test showed 42 differentially expressed PGs (39 COG names, 12 COG category, 28 KEGG names, 42 taxonomic assignments) in WBSs based on nutritional habit; 20 PGs were over-, and 22 PGs under-expressed in patients who didn’t follow compared to those who followed an omnivorous diet (Supplementary File 5). Actinobacteria accounted for the 11.90% of quantified PGs (5 PGs, under-expressed), Bacteroidetes for the 30.95% (13 PGs, over-expressed) and Firmicutes 57.14% (7 over-expressed and 17 under-expressed PGs). Twelve PGs (28.57%) were attributable to the G COG category and six PGs (14.29%) to C. Five PGs were associated to ABC transporters KEGG classification, 3 PGs to Galactose metabolism and 3 PGs to Glycolysis/Gluconeogenesis. Among these modulated PGs, 22 had VIP > 2; four belonged to M COG category [Outer membrane protein TolC, Opacity protein and related surface antigens, Outer membrane protein OmpA and related peptidoglycan-associated (lipo)proteins, Dihydrodipicolinate synthase/N-acetylneuraminate lyase]; three belonged to G COG category (Triosephosphate isomerase, ABC-type glycerol-3-phosphate transport system, periplasmic component and ABC-type sugar transport system, periplasmic component, contains N-terminal xre family HTH domain).

Seven and 14 PGs were under- and over-expressed, respectively in obese WBSs compared to non-obese WBSs. The all 21 PGs were associated to 18 COG names, 8 COG category, 17 KEGG names, and 19 taxonomic assignments (Supplementary File 5). Nineteen PGs (90.48%) were associated to Firmicutes: 33% were over-expressed (7 total PGs, 4 Ruminococcaceae, 3 *Ruminococcus bromii* PGs) and 57.14% were under-expressed (14 PGs, 3 Ruminococcaceae, 2 *Faecalibacterium prausnitzii* PGs). All these 21 differentially expressed PGs had also VIP > 2 respect the 34 total PGs with VIP > 2. The main represented COG and KEGG categories were: Carbohydrate transport and metabolism [G] (6 PGs), Amino acid transport and metabolism [E] (3 PGs), ABC transporters (5 PGs), Alanine, aspartate and glutamate metabolism (2 PGs).

By considering GI symptoms, the comparison between presence *vs.* absence allowed us to identify 17 differentially expressed PGs (17 COG names, 6 COG categories, 14 KEGG accession, and 17 taxa assignments): 5 PGs were over-expressed in WBS patients with GI symptoms and 12 were under-expressed (Supplementary File 5). All 17 PGs belonged to the 29 PGs group with VIP > 2. Three over- and 12 under-expressed PGs were associated to Firmicutes (88.24%), while 1 over-expressed PG was associated to Bacteroidetes (5.88%). The main represented COG and KEGG categories were: Energy production and conversion [C] (6 PGs), Carbohydrate transport and metabolism [G] (4 PGs), Nucleotide transport and metabolism [F] (3 PGs), Purine metabolism (4 PGs) and Glycolysis/Gluconeogenesis (3 PGs).

Taking into consideration the comparison amongst WBSs, stratified for diet, obesity and GI symptoms but also the comparison between all WBSs and CTRLs, the frequency of modulated PGs organized in COG categories highlighted two main categories with a trend from CTRLs to the WBSs group with GI symptoms, going through all WBS, no omnivorous WBSs, obese WBSs: increased for V and decreased for J COGs, respectively (Supplementary Figure 6.A). The same comparison based on KEGG names evidenced an increment of Alanine, aspartate and glutamate metabolism and Galactose metabolism, and a decrement of Ribosome and Taurine and hypotaurine metabolism (Supplementary Figure 6.B).

### Host protein counterpart in WBS-related GM metaproteome

**Figure 6:**
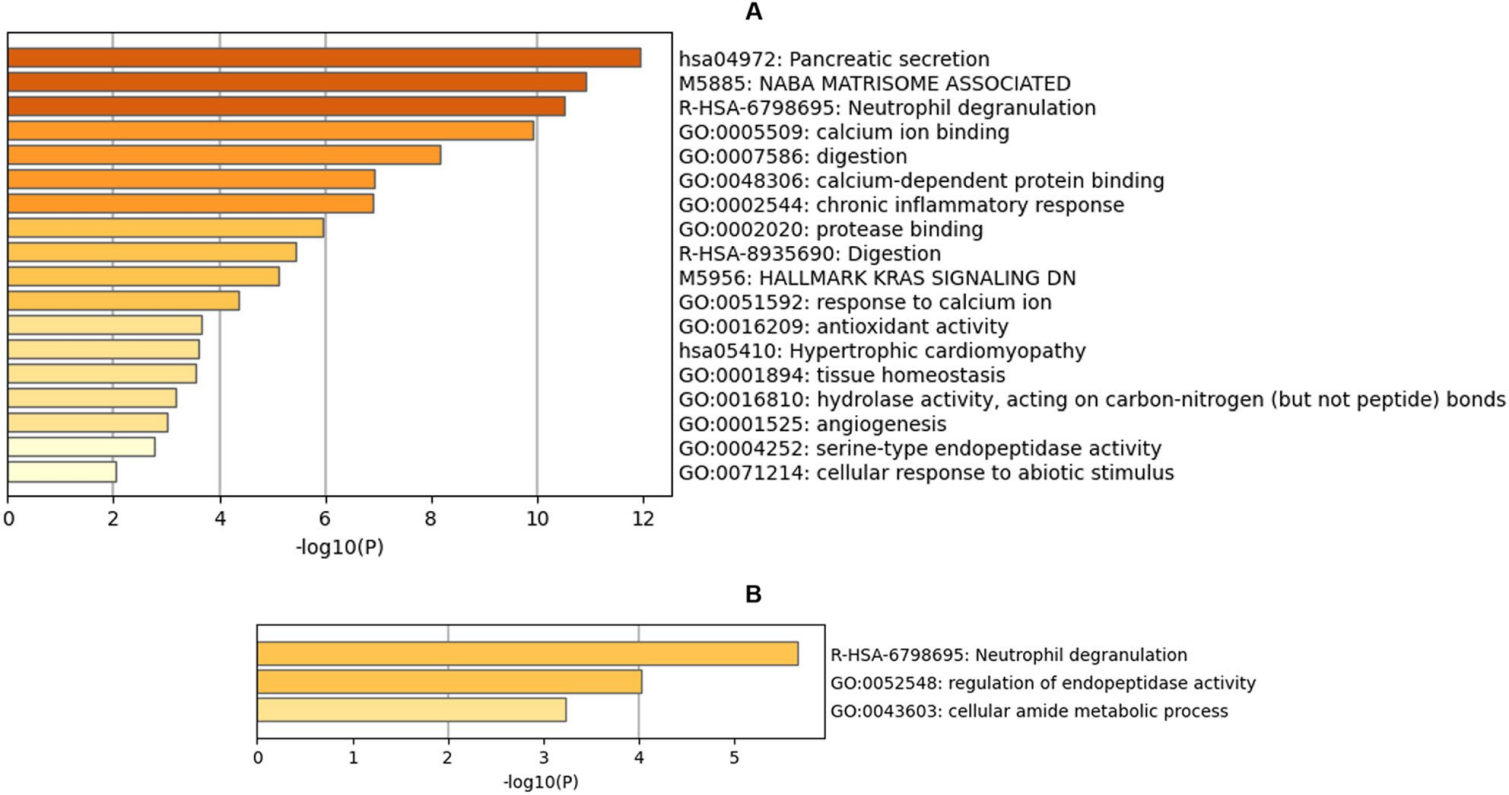
Bar graph of enriched terms (top clusters) across input human protein list of: (**A**) 42 identified human proteins and (**B**) 7 differentially expressed human proteins for the comparison WBSs versus CTRLs, coloured by *p* values. Image created by Metascape (version 3.5, https://metascape.org/).

Forty-two human PGs were characterized by an enrichment process of human pathways from different ontology sources. Hence, Pancreatic secretion and NABA MATRISOME ASSOCIATED (i.e., extracellular matrix (ECM)-associated) proteins, were primarily identified (Fig. 6A, Supplementary Files 3 and 6, and Supplementary Figure 7).

Beta-diversity analysis assessed a statistically significant difference between WBS and CTRL groups (*p* value = 0.008); their separation was slightly reported also by PCA and PLS-DA (Supplementary Figure 7). MANOVA test also yielded a statistically significant difference (*p* value = 0.0005) using the first top 29/42 PGs determining the PC1 loadings coefficient of PCA.

Also, univariate analysis, performed on the total average proteins’ intensities, produced significant difference (Mann–Whitney test *p* value < 0.0001). Then, considering only the seven over-expressed proteins of WBSs respect to CTRLs, with t-test *p* value < 0.05 (Supplementary File 3), the first enriched cluster was associated to the Neutrophil degranulation REACTOME pathway (R-HSA-6798695), including Aminopeptidase N, Annexin A2, Protein S100-A8, and Pantetheinase (Fig. 6B and Supplementary File 6). To note, these 7 over-expressed proteins belonged to the most important features’ group (*i.e*., 29) contributing to PC1, but also were part of the proteins’ group with VIP scores > 1 (*i.e*., 19) contributing to the PLS-DA model (Supplementary File 3).

## Discussion

The GM is modulated from birth to senescence, changing significantly during early life span and apparently stabilizing in healthy adults^17,18^. Our patient cohort’s age was wide, ranging from 1 to 42 years, hence the presence of an age-driven factor in the modulation of GM bacteria PGs’ was investigated. The sample sizes obtained by stratifying our cohort into age groups may have reduced the statistical power of the results and may represent a limitation of the study. This was particularly evident in the comparison of 5 toddlers with WBS *vs*. 5 CTRLs, and 7 WBS *vs*. 13 CTRLs in adults. However the results was intriguingly. Indeed, only for the healthy subjects, included in the study as reference GM baseline, the GM metaproteome was affected by differences through the entire age range (toddlerhood, childhood, adolescence, and adulthood), consistently with literature^17,18^. Conversely, for the WBS patients, GM metaproteome variations were reported only by the pairwise comparison between toddlerhood and childhood by univariate analysis. Thus, the metaproteomic analysis suggests that age did not significantly affect the composition of the WBS GM, in contrast to the impact observed in the CTRL group. This evidence led us to speculate a major role of the WBS disease phenotype in the shaping of the GM functional signatures, compared to age dependency. Indeed, a previous 16S rRNA-based metagenomic study revealed the absence of statistically significant differences amongst age for the WBS group excluding the variable age as confounding factor, and provided interesting results comparing the WBSs to age-matched healthy subjects^19^. Therefore, we proceeded by analyzing all WBS samples and comparing them to the CTRL group, regardless of age, to establish a comprehensive understanding of the WBS impact on GM functionality. The examined stool samples of 41 WBS patients derived from the identical cohort as the metagenomic study, which had an extra 5 samples excluded from the metaproteomic analysis due to limited availability. Comparisons were made with a group of 45 healthy subjects, out of which 16 individuals overlapped with the CTRL metagenomic cohort.

Moreover, local “microbiome branches” that are dynamically linked within global GM gradients^20^ or “guilds” of “enterosignatures”^21^, which describe most of the gut microbial variance at the genus-level composition, representing an evolution of the *Prevotella* and *Bacteroides* enterotype concept^22^, may result in an important GM variable in ecological studies, implying the need for a strict characterization of healthy subjects’ datasets. Therefore, as our CTRL baseline is mainly characterized by a *Bacteroides* enterotype, it may be appropriate to scale up our CTRL populations in further studies to fully address GM markers of diseases also in metaproteomics.

However, a specific GM functional disease related-pattern was provided by both multivariate and univariate analyses: WBS-related GM functional signatures appeared linked to protein biosynthesis, biogenesis of cellular structures, and metabolic pathways related to amino acid, carbohydrate, and energy production, as already inferred from the targeted metagenomic approach performed on the same cohort of patients^19^. In particular, the differential expression of Glyceraldehyde-3-phosphate dehydrogenase and Ferredoxin highlighted a modulation of the Glycolysis/Gluconeogenesis pathway, while the differential expression of Pyruvate-formate lyase and Acetate kinase suggested a modulation of the metabolic patterns downstream the pyruvate cycle, and towards formate and acetyl-CoA dependent acetate production (Supplementary Figure 9). Interestingly, Acetate kinase takes also part of the Taurine and hypotaurine metabolism, which in turn exhibits anti-oxidative activities and plays an important role in bile acid metabolism pathway^23–25^. Over-expression of Pyruvate-formate lyase and the consequent formate production may be an evidence of WBS gut inflammation^26^. Ferredoxin is a redox protein that carries electrons and facilitates electron transfer in various metabolic reactions by means of its iron-sulphur clusters to assist in the generation of ion motive forces. It has been hypothesised that Firmicutes make larger use of low-potential electron transporters such as ferredoxin, although Bacteroidetes taxa have more types of genomic ferredoxin clusters than Firmicutes^27^. This difference in utilization likely results in the use of a distinct redox tower scale for metabolic reactions in Firmicutes^28^. Indeed, several PGs associated with the Ferrodoxin COG name were identified. However, all the PGs were down-expressed in the WBS groups and it could be attributed to Firmicutes (Clostridiales, *F. prausnitzii*, and *Roseburia faecis*). This outcome might be linked to an imbalance in the metabolism of the GM of WBS, which could be due to important loss of functional Firmicutes.

Compared with CTRLs, WBSs showed differences also in GM pathways associated to carbohydrate transport. The differential expression of Phosphoenolpyruvate-protein kinase (Enzyme I component of the phosphotransferase system [PTS] in bacteria)^29^ and ABC-type glycerol-3-phosphate transport system, a periplasmic component^30^, may highlight a possible impact of WBS syndrome through a change in the absorption and fluxes of sugars and their derivatives.

Non-omnivorous diet, obesity and GI symptoms also influenced the GM of WBSs. Indeed, patients’ GM with these features showed, compared to patients without these traits, an increment of PGs linked to Defense mechanisms, Alanine, aspartate and glutamate metabolism and Galactose metabolism and a decrement of PGs associated to Translation, ribosomal structure and biogenesis, Ribosome and Taurine and hypotaurine metabolism. The alteration of GM proteome scaffold seems to reflect a worsening of the gut eubiosis through a growing functional impairment in CTRLs, WBSs, WBSs with incorrect dietary habits, obese WBSs and WBSs with GI symptoms.

Moreover, the taxonomic assignments, performed by LCA algorithm, unveiled a specific WBS ecological pattern: Bacteroidetes and Firmicutes were the main phyla associated to GM over- and under-expressed bacterial PGs, respectively. The ratio of Firmicutes/Bacteroidetes, the two GM dominant phyla, has being taken into consideration as a putative marker for dysbiosis and inflammatory diseases; indeed, it has been hypothesized as relevant hallmark for obesity and inflammatory diseases although with discordant trend and controversial results^31^. Among GM species belonging to Firmicutes phylum, key butyrogenic bacteria are *F. prausnitzii*, *Roseburia intestinalis*, and *Agathobacter rectale*. Butyrate, a SCFA, is a crucial metabolite generated by the disruption of dietary nondigestible carbohydrates such as cellulose, inulin, and resistant starch in the intestine’s lumen. Together with the other SCFAs present in the gut, such as acetate and propionate, butyrate has profound beneficial effects on the health and physiology of the human host preserving the gut barrier from injuries, giving energy to gut epithelium cells and activating anti-inflammatory pathways^32^. Indeed, *F. prausnitzii* has been identified as disease-related microbial marker; its depletion has been associated to several diseases (obesity, type 2 diabetes, inflammatory disease) and it has been described as a candidate for next-generation probiotics^33,34^.

Amongst the 53 differentially expressed PGs associated to Firmicutes, for the LCA taxonomic assignment to species level, 8 PGs were attributable to *F. prausnitzii*, of which 7/8 were down-expressed compared to CTRLs, suggesting that *F. prausnitzii* functional pathways were almost impaired, as also observed for the *S. variabile* species, associated to 8 down-expressed PGs (Table 2 and Supplementary File 2)*.* Remarkably, *S. variabile* is a butyrate-producer and *Subdoligranulum* spp. has been already negatively associated with metabolic risk^35^. Reduction of Firmicutes was also evidenced in WBSs patients by metataxonomy^19^. Anyway, metaproteomic results were not completely overlapping to that obtained by the previous cited 16S rRNA-based metagenomic study. Indeed, especially when dealing with complex microbial populations and lacking sample-specific shotgun metagenomic derived databases, taxonomic outcomes may vary significantly. This stems from the fact that meta-omic disciplines employ distinct analytical techniques on various molecular species^36^.

Regarding the human PGs, only few were highlighted in the comparison of the GM of WBSs compared to CTRLs, but almost all of them were ascribed to defense mechanisms, most likely due to inflammatory host response to WBS dysbiotic gut. The modulated neutrophil degranulation pathway and the over-expression of the calprotectin S100A8 subunit were identified in WBSs. In particular, the S100A8 is a calcium- and zinc-binding protein which, together with the S100A9, forms the hetero complex of the calprotectin derived from human neutrophils, monocytes, and macrophage activities^37^. Calprotectin has a plethora of intra- and extracellular functions and, in particular, is released in the extracellular milieu after stimulation or by cell disruption or death, exerting antimicrobial, oxidant-scavenging and apoptosis-inducing activities and playing a prominent role in the regulation of pro-inflammatory processes and immune response^38^. Calprotectin levels in feces represent a valuable marker of intestinal inflammation because quantitatively relate to neutrophil migration toward the GI tissue and seem to be not affected by causes of inflammation other than intestinal ones, unlike other systemic inflammatory markers. This evidence could suggest the presence of an inflamed state at the level of the WBS intestine.

Hence, regardless selective extraction procedure for bacteria enrichment, the human characterized PGs were ascribable to inflammatory state biomarkers of the WBS gut. This evidence could be consistent with the decrease of PGs associated to SCFA-producing bacteria in agreement with an impairment of the SCFA metabolism pathways, and then with a less healthy status of the enterocytes. All together, these data indicate a dysbiotic alteration of WBS GM communities compared to age-matched CTRLs, suggesting a significant impairment of beneficial gut bacteria metabolism. Specifically, the evidence of decreased *F. prausnitzii* levels may suggest a target for tailored interventions through novel probiotics and postbiotics that can improve GI comorbidities in WBS patients.

## Subjects and methods

### Patient enrolment and sample collection

Patients with a diagnosis of WBS, based on clinical assessment and detection of recurrent heterozygous microdeletion of 7q11.23 chromosome, confirmed by either Fluorescent In Situ Hybridization^39^ or Chromosomal Microarray Analysis^40,41^, were enrolled at Medical Genetics Unit, Bambino Gesù Children's Hospital (OPBG), Rome, Italy.

Stool were stored at − 80 °C, until processing, while fecal samples from healthy age-matched subjects were available at the Microbiome Biobank of OPBG, Italian node of the Biobanking and Bio Molecular Resources Research Infrastructure. Healthy subjects were normal weight, with omnivorous dietary habits (mainly Mediterranean diet) and did not reported any GI diseases or functional symptoms such as diarrhea, constipation, abdominal pain.

Neither patients nor healthy subjects had taken antibiotics, prebiotics or probiotics within one month before sampling.

The study protocol was performed in accordance with the Principles of Good Clinical Practice and Declaration of Helsinki, and was approved by OPBG Ethical Committee (protocol code 2590_OPBG_2021 and healthy subjects: 1113_OPBG_2016). Written informed consent to participate in this study was provided by patients or the children’ legal guardian/next of kin.

### Protein extraction and enzymatic digestion

Fecal bacteria were isolated by suspension of 300 mg of thawed material in 6 mL of ice-cold phosphate buffer. Protein extraction was performed with 300 μL lysis buffer [4% sodium dodecyl sulphate (SDS), 100 mM Dithiothreitol (DTT) in 50 mM Tris–HCl pH 8, with addition of Halt Protease and Phosphatase Inhibitor Cocktail (Thermo Fisher Scientific, Waltham, MA, USA)], with further heating, sonication, centrifugation and protein quantification^42^.

After disulphite bonds reduction and alkylation, 50 μg of each protein extract was digested according to filter-aided sample preparation (FASP) protocol^43,44^ with Sequencing grade Trypsin (Promega, Milan, Italy) at a 1:50 (w:w) ratio of enzyme to sample for 16 h. Peptides were eluted from the Microcon, speedvac dried and resuspended in 2% Acetonitrile (ACN), 0.1% Formic Acid (FA) and 97.9% water. Total peptide content was determined by NanoDrop 2000 (Thermo Fisher Scientific) analysis with a standard curve of MassPrep *Escherichia coli* digestion (Waters, Milford, Massachusetts, USA).

### Mass spectrometry analysis

NanoLiquid Chromatography-ElectroSpray Ionization-tandem mass spectrometry (nLC-ESI–MS/MS) experiments were carried out using an UltiMate3000 RSLCnano System coupled to a Orbitrap Fusion Tribrid mass spectrometer through a nanoESI source (EASY-Spray NG) (Thermo Fisher Scientific) as already published^42^. An EASY-Spray PepMap RSLC C18 column (2 μm particle size, 100 Å pore size, 75 μm i.d. × 50 cm length, Thermo Fisher Scientific) was employed to separate the digested samples hyphenated with a μ-Precolumn C18 PepMap100 (5 µm particle size, 100 Å pore size, 300 µm i.d. x 5 mm length, Thermo Fisher Scientific) operating at 10 μL/min, 3 min, to concentrate and desalt 1.95 μg of peptides. Reverse-phase chromatography was performed in 143 min, and total LC-run of 181 min at a flow rate of 250 nL/min and temperature of 35 °C. MS analysis was done with a full MS scan from 375 to 1,500 *m/z* in Orbitrap at a resolving power of 120 K and positive ionization mode, followed by data-dependent MS/MS scan recorded by Ion Trap at rapid scan rate, in top speed mode with a 3 s cycle-time, with dynamic exclusion enabled for 1 min. Two technical replicates were acquired for each sample.

### Metaproteomic data processing

nLC-ESI–MS/MS data were analyzed by MaxQuant version 2.0.1^45,46^ and MetaLab desktop version 2.0^47^ in order to perform both the label free quantification (LFQ) and the taxonomic and functional annotation analyses. Data normalization was performed by MaxQuant LFQ algorithm^45^. Search parameters included carbamidomethylation of cysteine as fixed modification, protein N-terminal acetylation and oxidation of methionine as variable modification, maximum two missed cleavages, High-Low instrument resolution. Databank searching was performed versus the IGC-“Integrated reference catalog of the human gut microbiome”, Human Gut (http://meta.genomics.cn/—9.9 M) and *Homo sapiens* UniProtKB/Swiss-Prot reference proteome (UP000005640, release 2021_03 of 02-Jun-21) databases. A false discovery rate of 0.01 was set for the identification of Proteins Groups (PG) and peptides. MetaLab parameters were set in order to generate sample specific database, to adopt spectra clustering strategy during the first search, and to perform taxonomic assignment of bacteria peptides by built-in database using the Lowest Common ncestor (LCA) algorithm^48^.

The bioinformatic pipeline was performed by Python version 3.7 ad hoc scripts applying pandas, numpy, scipy, skbio.diversity, and scikit-learn as main packages, including parallel processing steps for bacteria and human PGs. MetaLab output files were collapsed into a final comprehensive matrix where PGs, along with their LFQ intensity, were associated to functional annotations [Clusters of Orthologous Groups (COG) name and category of proteins; Non-supervised Orthologous Groups (NOG) name; Kyoto Encyclopedia of Genes and Genomes (KEGG)^49^ and Gene Ontology (GO) accessions] and taxonomy based on LCA^42^.

### Biostatistics and biochemical pathway analyses

Multivariate Bray–Curtis β-diversity, Principal Component Analysis (PCA), Partial Least Squares-Discriminant Analysis (PLS-DA), were computed on the final LFQ intensities’ PG matrix. To test association between covariates and Bray–Curtis β-diversity, permutational multivariate analysis of variance (PERMANOVA, 9,999 permutations) was employed, while to compare the multivariate means of subgroups MANOVA test was calculated.

Univariate analyses were conducted on the final LFQ intensities’ PG matrix to analyze the average intensities of PGs between sample groups, utilizing the Mann–Whitney test.

After conducting a Shapiro–Wilk test to investigate data distribution, univariate comparisons to identify differential individual PGs between the groups were carried out on the final LFQ intensities’ PG matrix, applying t-test and filtering values by abundance ratio ≥ 1.5 and ≤ 0.67, and *p* value < 0.05.

The COGs list of differentially expressed bacteria PGs were analyzed using Interactive Pathways Explorer v3^50^.

Identified and differentially expressed human PGs’ list was uploaded in Metascape 3.5 for human pathway and process enrichment analysis with different ontology sources updated on 2022-04-22^51^. In this analysis *p* values were calculated based on the accumulative hypergeometric distribution, while for q-values the Benjamini–Hochberg procedure was applied to account for multiple testings on pathway enrichment. Terms with a *p* value < 0.01, a minimum count of 3, and an enrichment factor > 1.5 were collected and grouped into clusters based on their membership similarities. Kappa scores were used as the similarity metric when performing hierachical clustering on the enriched terms, and sub-trees with a similarity of > 0.3 were considered a cluster. The most statistically significant term within a cluster was chosen to represent the cluster.

### Supplementary Information

Supplementary Figures and Table.

Supplementary File 1.

Supplementary File 2.

Supplementary File 3.

Supplementary File 4.

Supplementary File 5.

Supplementary File 6.

## Data Availability

Mass spectrometry data presented in the study can be retrieved via MASSIVE public repository with the identifier code MSV000089691.
