# Supplementary material for: Williams–Beuren syndrome shapes the gut microbiota metaproteome
Source: Sci Rep. 2023 Nov 3;13:18963. doi: 10.1038/s41598-023-46052-9 (PMC10624682; doi:10.1038/s41598-023-46052-9)
Supplement: Supplementary file 1 — Supplementary Figures and Table. [file 41598_2023_46052_MOESM1_ESM.pdf]

Supplementary materials

(Figures, Table and Files)

Supplementary Figures.

Supplementary Figure 1. Scatter plot graph of quantified extracted proteins (A), obtained from stool samples of patients (WBSs) and age-matched healthy subjects (CTRLs), and purified peptides (B). Mean values are evidenced as red bar.

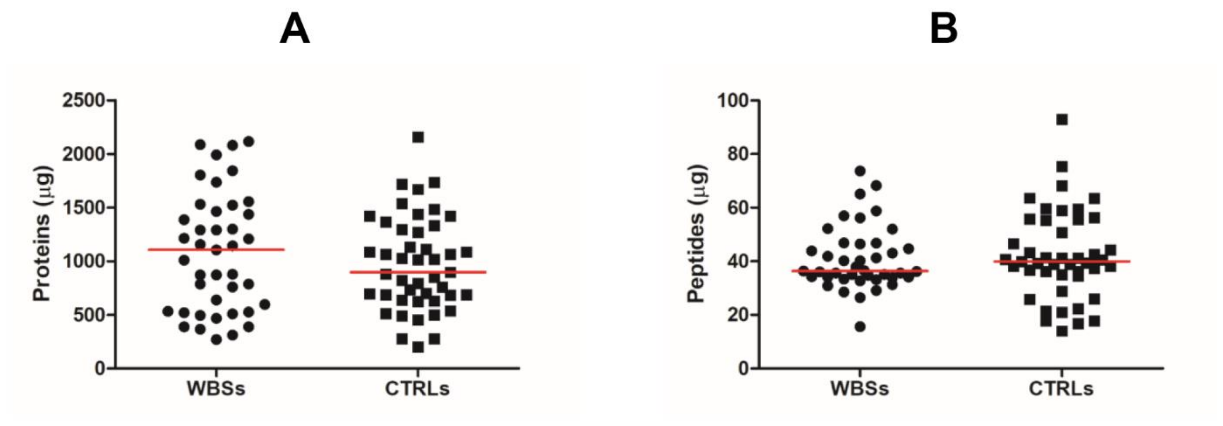

Supplementary Figure 2. Analyses of identified bacteria protein groups from stool samples of patients (WBSs, blue color) and age-matched healthy subjects (CTRLs, orange color). (A) The dissimilarity between samples' groups by the unsupervised Bray-Curtis β-diversity analysis was measured according to their proteins' content. WBS and CTRL groups showed a statistically significant (p-value < 0.001) differences assessed by PERMANOVA test. (B) Partial Least Squares-Discriminant Analysis showed a segregation between WBS and CTRL groups. The variances explained by each component are stated in brackets. (C) Variable importance in projection (VIP scores) showing the 21 protein groups, displayed with their corresponding COG category and ID in brackets, providing the major contribution to the separation of WBSs from CTRLs. N.A.: not available

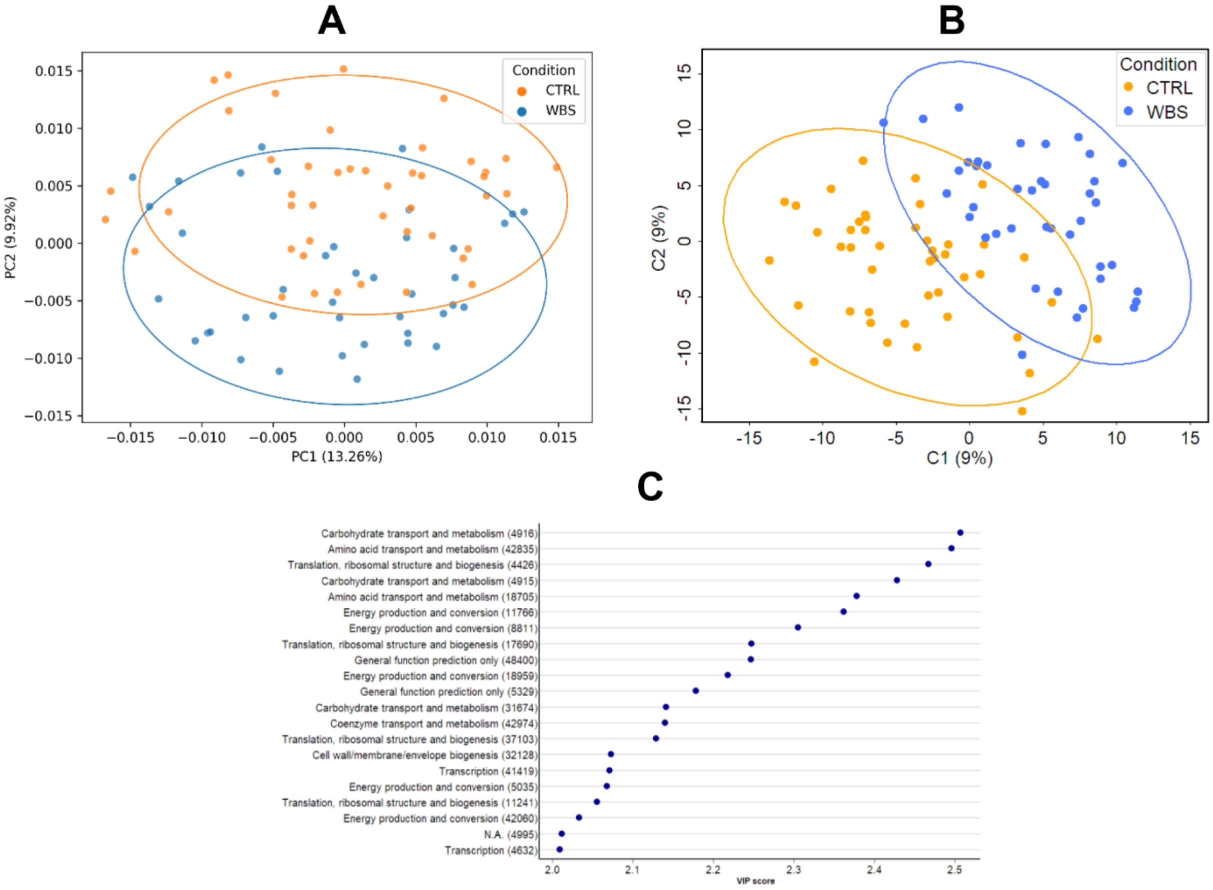

Supplementary Figure 3. Graphical representation of the differentially expressed bacteria protein groups comparing WBSs to CTRLs. (A) Bars represent the ratio of the protein groups' intensities expressed as [log10 (abundance WBS) - log10 (abundance CTRL)], associated with the respective functional annotation (COG name) and with the LCA depicted by a colour code. (B) Volcano plot of all comparisons filtering values by abundance ratio ≥ 1.500 and ≤ 0.667, and p-value < 0.05 established by t-test. Dashed black lines indicate the set limits of abundance ratio and statistically significance values. Green circles indicate the changes for significant protein groups. Volcano plots were displayed using log10[abundance ratio] and –log10[p-value].

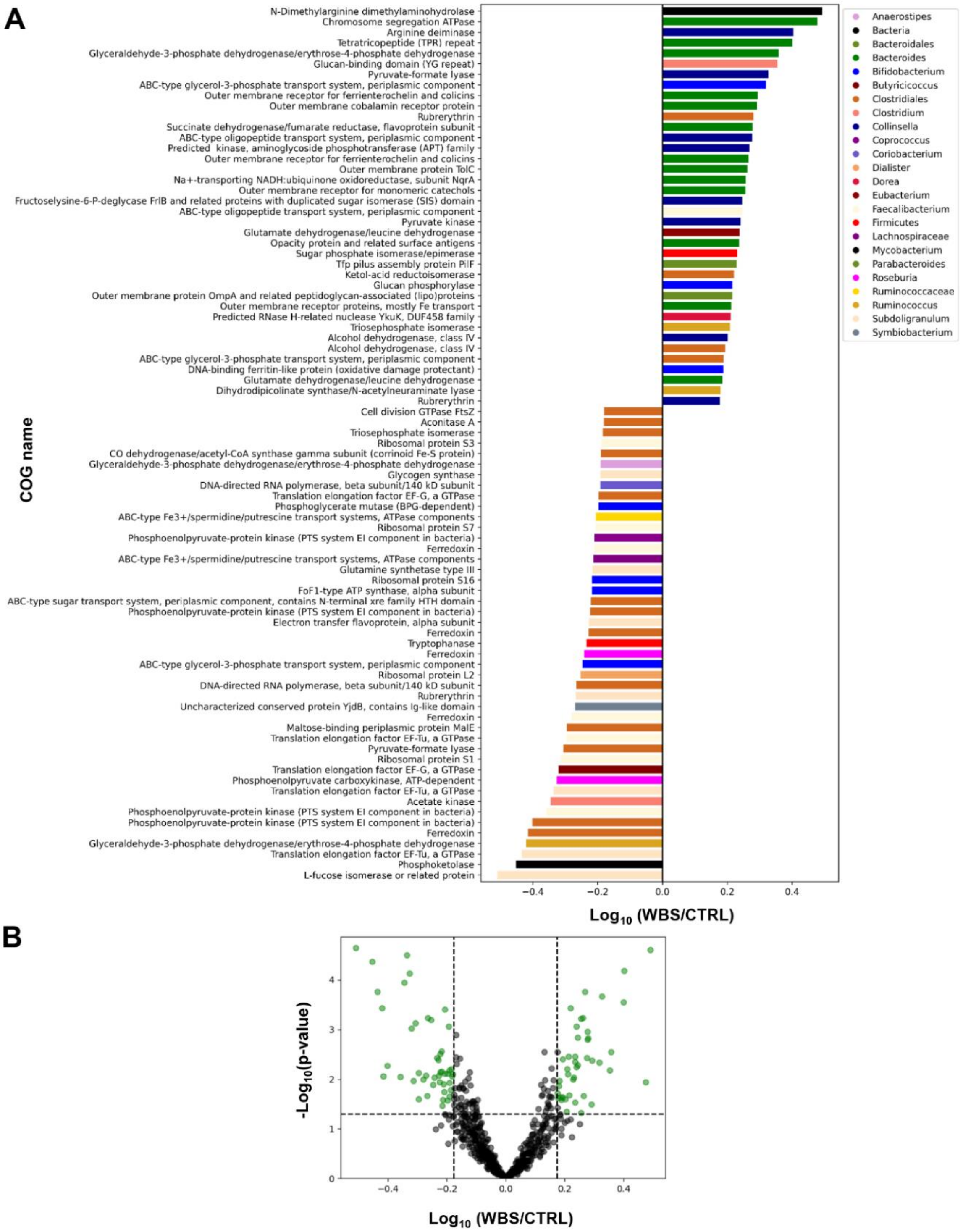

Supplementary Figure 4. Graphical sketch of the modulated KEGG categories corresponding to the differentially expressed bacteria protein groups comparing WBSs to CTRLs. Bars represent the number of over- and under-expressed PGs, associated with the respective functional annotation and with the LCA depicted by a color code. The most represented terms were Glycolysis/Gluconeogenesis, ABC transporters, Pyruvate metabolism, Alanine, aspartate and glutamate metabolism, and Ribosome. Two-third of PGs linked to Glycolysis/Gluconeogenesis were under-expressed in WBS group, as well as almost all PGs associated to Pyruvate metabolism, and all PGs related to Ribosome; Purine, Tyrosine, Fructose and mannose, Taurine and hypotaurine, Methane, and Nitrogen metabolism; Pentose phosphate pathway; Oxidative phosphorylation, Terpenoid backbone biosynthesis. Terms characterizing over-expressed PGs were: Alanine, aspartate and glutamate, Glycine, serine and threonine, Arginine and proline, Inositol phosphate Starch and sucrose metabolism; Lysine, Valine, leucine and isoleucine biosynthesis; Bacterial secretion system.

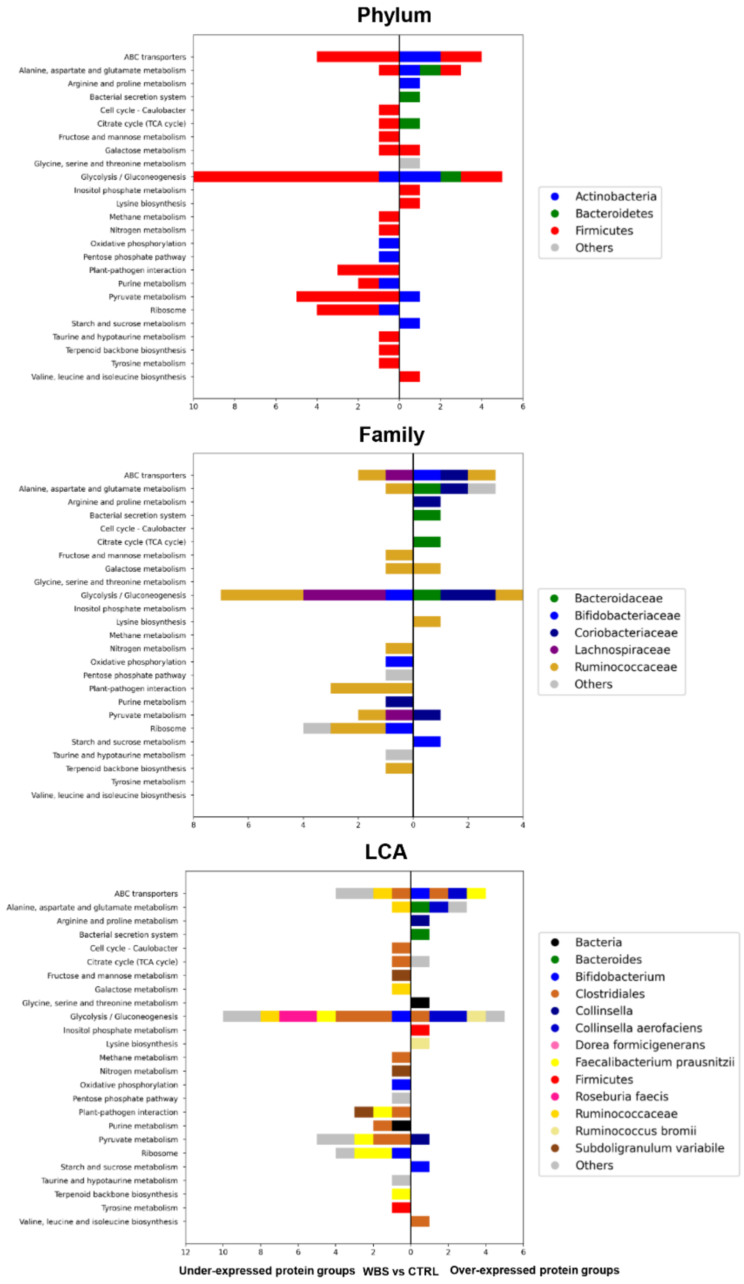

Supplementary Figure 5. Bray-Curtis β-diversity graphs of identified bacteria protein groups from WBS stool samples. Analysis were carried out separately stratifying samples by: A, omnivorous diet (YES = patients who followed an omnivorous diet, blue color; NO = patients who did not follow such a diet, orange color); B, obesity (NO = non-obese patients, blue color; YES = obese patients, orange color); and C, gastrointestinal (GI) symptoms (NO = absence of symptoms, blue color; YES = presence of at least one symptoms, orange color).

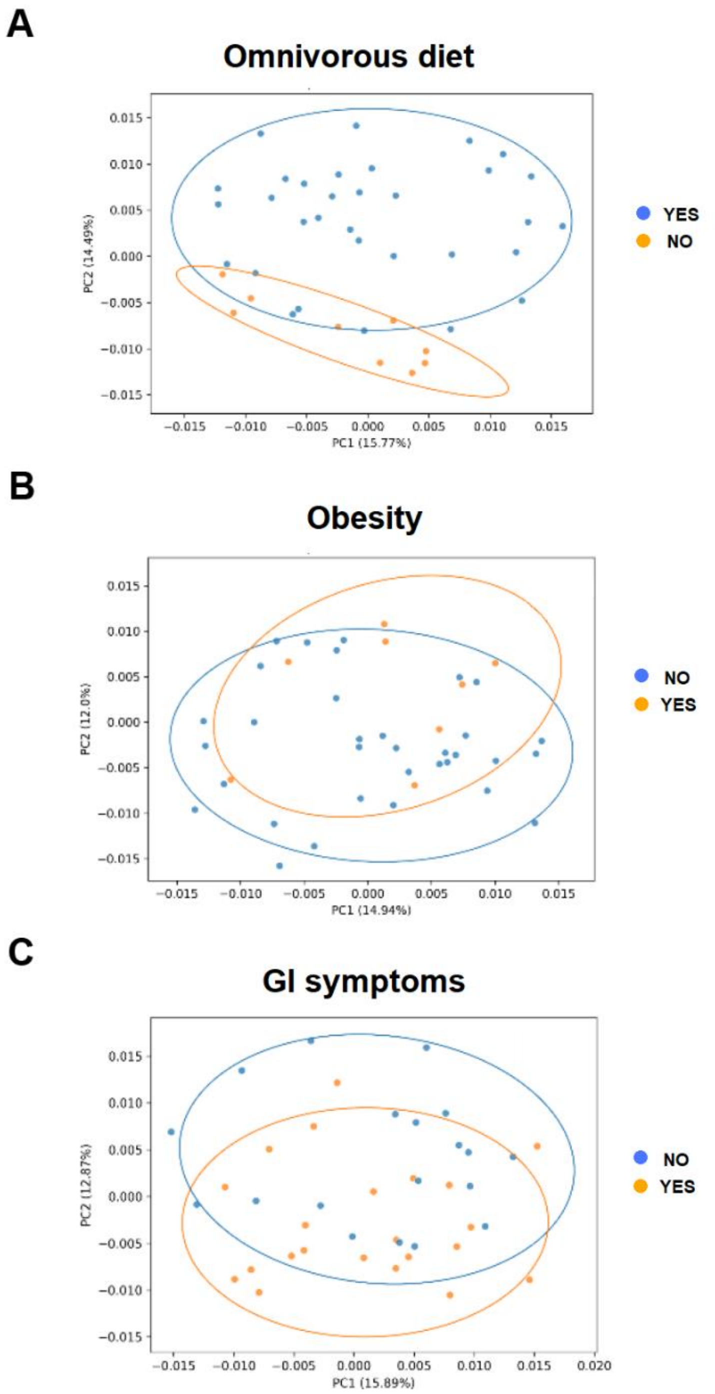

Supplementary Figure 6. Analysis of the frequency of number of modulated PGs of each COG category (A) and KEGG pathway (B) across the entire dataset of identified PGs and in the differentially expressed PGs.

A

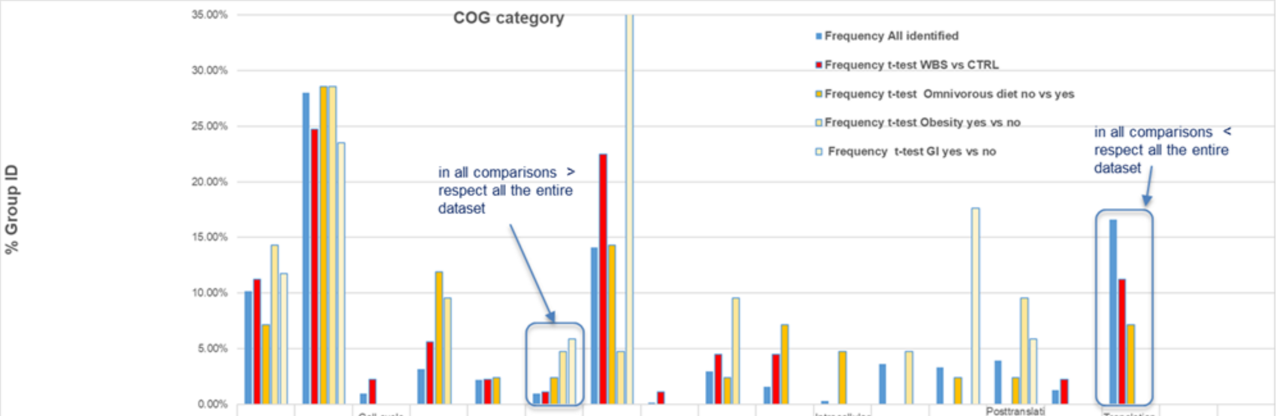

	Amino acid transport and metabolism [E]	Carbohydrate transport and metabolism [G]	Cell cycle control, cell division, chromosome partitioning [D]	Cell wall/membrane/envelope biogenesis [M]	Coenzyme transport and metabolism [H]	Defense mechanisms [V]	Energy production and conversion [C]	Extracellular structures [W]	General function prediction only [R]	Inorganic ion transport and metabolism [P]	Intracellular trafficking, secretion, and vesicular transport [U]	Lipid transport and metabolism [I]	Nucleotide transport and metabolism [F]	Posttranslational modification, protein turnover, chaperones [O]	Transcription [K]	Translation, ribosomal structure and biogenesis [J]
Frequency All identified	10.17%	28.01%	0.94%	3.13%	2.19%	0.94%	14.08%	0.16%	2.97%	1.56%	0.31%	3.60%	3.29%	3.91%	1.25%	16.59%
Frequency t-test WBS vs CTRL	11.24%	24.72%	2.25%	5.62%	2.25%	1.12%	22.47%	1.12%	4.49%	4.49%	0.00%	0.00%	0.00%	0.00%	2.25%	11.24%
Frequency t-test Omnivorous diet no vs yes	7.14%	28.57%	0.00%	11.90%	2.38%	2.38%	14.29%	0.00%	2.38%	7.14%	4.76%	0.00%	2.38%	2.38%	0.00%	7.14%
Frequency t-test Obesity yes vs no	14.29%	28.57%	0.00%	9.52%	0.00%	4.76%	4.76%	0.00%	9.52%	0.00%	0.00%	4.76%	0.00%	9.52%	0.00%	0.00%
Frequency t-test GI yes vs no	11.76%	23.53%	0.00%	0.00%	0.00%	5.88%	35.29%	0.00%	0.00%	0.00%	0.00%	0.00%	17.65%	5.88%	0.00%	0.00%

B

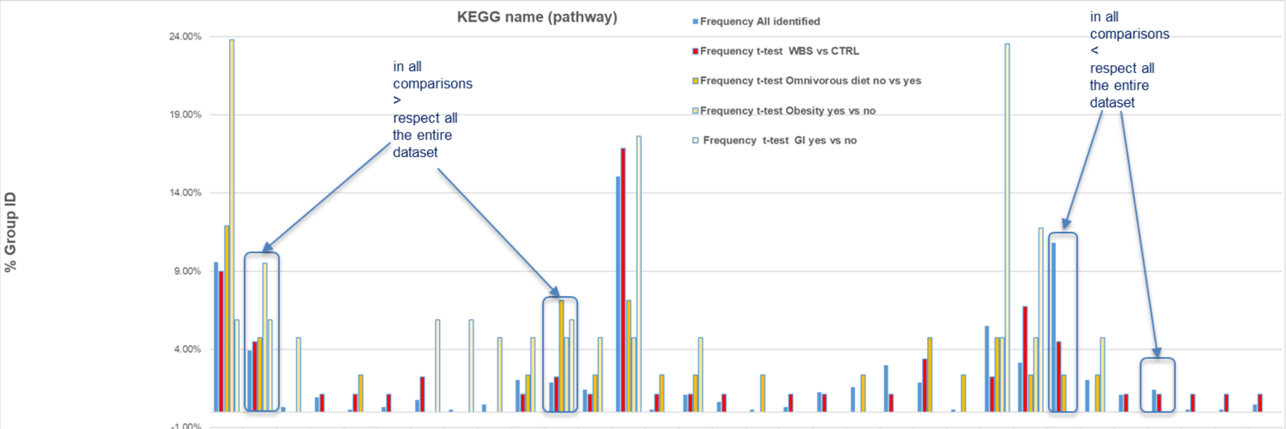

	ABC transporters	Alanine, aspartate and glutamate metabolism	Amino sugar and nucleotide sugar metabolism	Arginine and proline metabolism	Bacterial secretion system	Cell cycle - Caulobacter	Citrate cycle (TCA cycle)	Cyano amino acid metabolism	Fatty acid biosynthesis	Fructose and mannose metabolism	Galactose metabolism	Glycine, serine and threonine metabolism	Glycolysis / Gluconeogenesis
Frequency All identified	9.56%	3.92%	0.31%	0.94%	0.16%	0.31%	0.78%	0.16%	0.47%	2.04%	1.88%	1.41%	15.05%
Frequency t-test WBS vs CTRL	8.99%	4.49%	0.00%	1.12%	1.12%	1.12%	2.25%	0.00%	0.00%	1.12%	2.25%	1.12%	16.85%
Frequency t-test Omnivorous diet no vs yes	11.90%	4.76%	0.00%	0.00%	2.38%	0.00%	0.00%	0.00%	0.00%	2.38%	7.14%	2.38%	7.14%
Frequency t-test Obesity yes vs no	23.81%	9.52%	4.76%	0.00%	0.00%	0.00%	0.00%	0.00%	4.76%	4.76%	4.76%	4.76%	4.76%
Frequency t-test GI yes vs no	5.88%	5.88%	0.00%	0.00%	0.00%	0.00%	5.88%	5.88%	0.00%	0.00%	5.88%	0.00%	17.65%

	Inositol phosphate metabolism	Lysine biosynthesis	Methane metabolism	Microbial metabolism in diverse environments	Nitrogen metabolism	Oxidative phosphorylation	Pentose and glucuronate interconversions	Pentose phosphate pathway	Plant-pathogen interaction	Protein export	Purine metabolism	Pyruvate metabolism	Ribosome	RNA degradation	Starch and sucrose metabolism	Taurine and hypotaurine metabolism	Terpenoid backbone biosynthesis	Tyrosine metabolism	Valine, leucine and isoleucine biosynthesis
Frequency All identified	0.16%	1.10%	0.63%	0.16%	0.31%	1.25%	1.57%	2.98%	1.88%	0.16%	5.49%	3.13%	10.82%	2.04%	1.10%	1.41%	0.16%	0.16%	0.47%
Frequency t-test WBS vs CTRL	1.12%	1.12%	1.12%	0.00%	1.12%	1.12%	0.00%	1.12%	3.37%	0.00%	2.25%	6.74%	4.49%	0.00%	1.12%	1.12%	1.12%	1.12%	1.12%
Frequency t-test Omnivorous diet no vs yes	2.38%	2.38%	0.00%	2.38%	0.00%	0.00%	2.38%	0.00%	4.76%	2.38%	4.76%	2.38%	2.38%	2.38%	0.00%	0.00%	0.00%	0.00%	0.00%
Frequency t-test Obesity yes vs no	0.00%	4.76%	0.00%	0.00%	0.00%	0.00%	0.00%	0.00%	0.00%	0.00%	4.76%	4.76%	0.00%	4.76%	0.00%	0.00%	0.00%	0.00%	0.00%
Frequency t-test GI yes vs no	0.00%	0.00%	0.00%	0.00%	0.00%	0.00%	0.00%	0.00%	0.00%	0.00%	23.53%	11.76%	0.00%	0.00%	0.00%	0.00%	0.00%	0.00%	0.00%

Supplementary Figure 7. Image of the Pancreatic secretion KEGG (Release 102.0, April 1, 2022) pathway (hsa04972) showing involved factors/effectors, their subcellular distribution and their molecular interactions, reactions and relation networks. Red light box = human input proteins, identified in our dataset: amyA = Alpha-amylase 1A (P0DUB6) and Amylase alpha 2B (P19961, hsa:280); PRSS: Trypsin-2 (Serine protease 2, P07478, hsa:5645); CTRB1 = Chymotrypsinogen B2 (Q6GPI1, hsa:440387); CPA = Carboxypeptidase A1 (P15085, hsa:1357) and Carboxypeptidase A2 (P48052, hsa:1358); CPB = Carboxypeptidase B (Pancreas-specific protein) (P15086, hsa:1360); PLA2 = Phospholipase A2 (P04054, hsa:5319). [https://www.genome.jp/kegg/mapper/search.html; Kanehisa M., Protein Sci. 2019 Nov;28(11):1947-1951. doi: 10.1002/pro.3715]

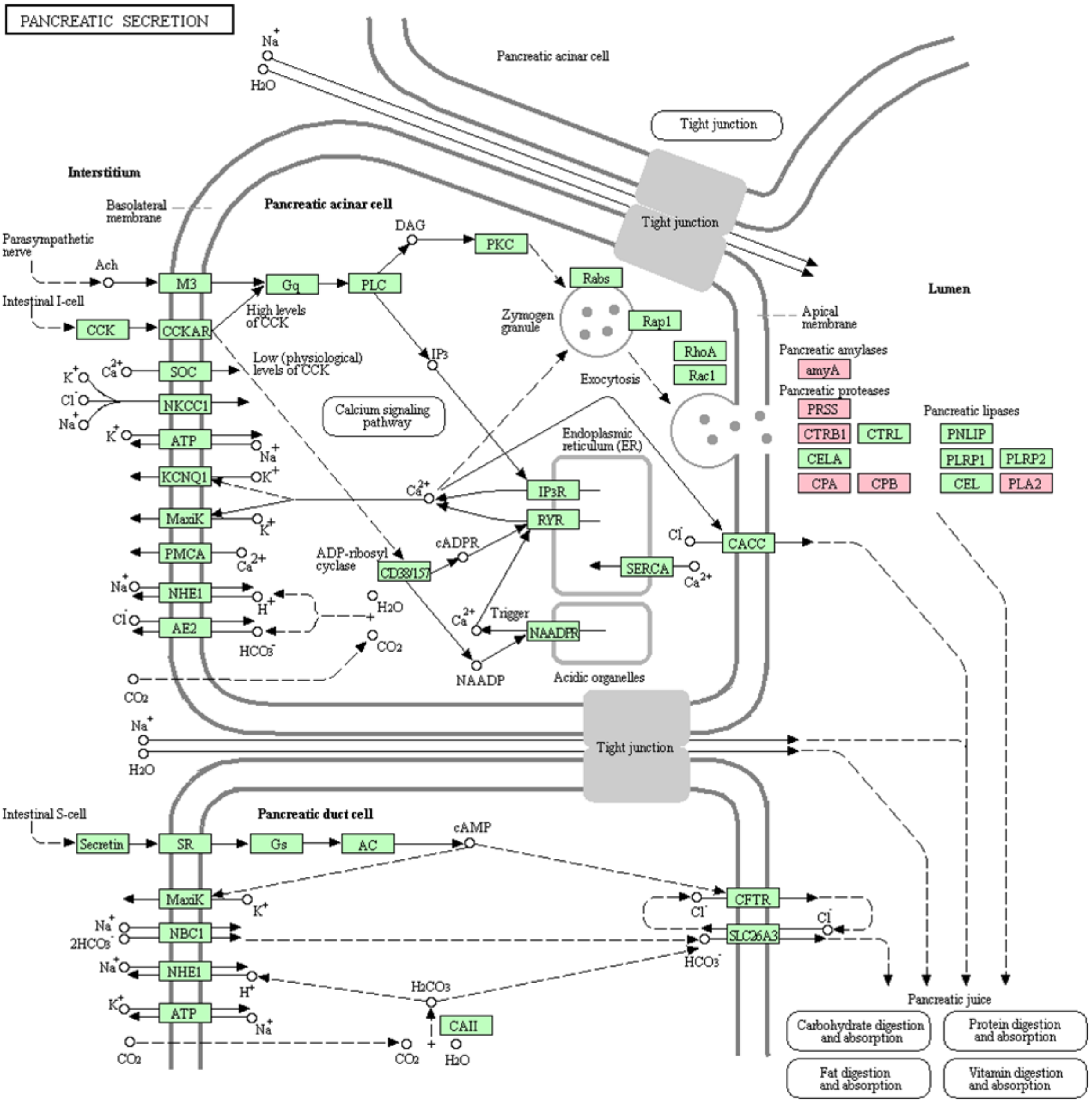

Supplementary Figure 8. Analyses of identified human protein groups (PGs) from stool samples of patients (WBSs, blue colour) and age-matched healthy subjects (CTRLs, orange colour). (A) The dissimilarity between samples' groups by the unsupervised Bray-Curtis β-diversity analysis was measured according to their proteins' content. WBS and CTRL groups showed a statistically significant (p-value < 0.001) differences assessed by PERMANOVA test. (B) Unsupervised Principal Component Analysis and (D) supervised Partial Least Squares-Discriminant Analysis displayed a slight separation amongst the two groups. The variances explained by each component are stated in brackets. (C) The top 29 PGs contributing to PCA's PC1 loadings are displayed with their corresponding values. (E) Variable importance in projection (VIP scores) show the 19 PGs providing the major contribution to the PLS-DA model.

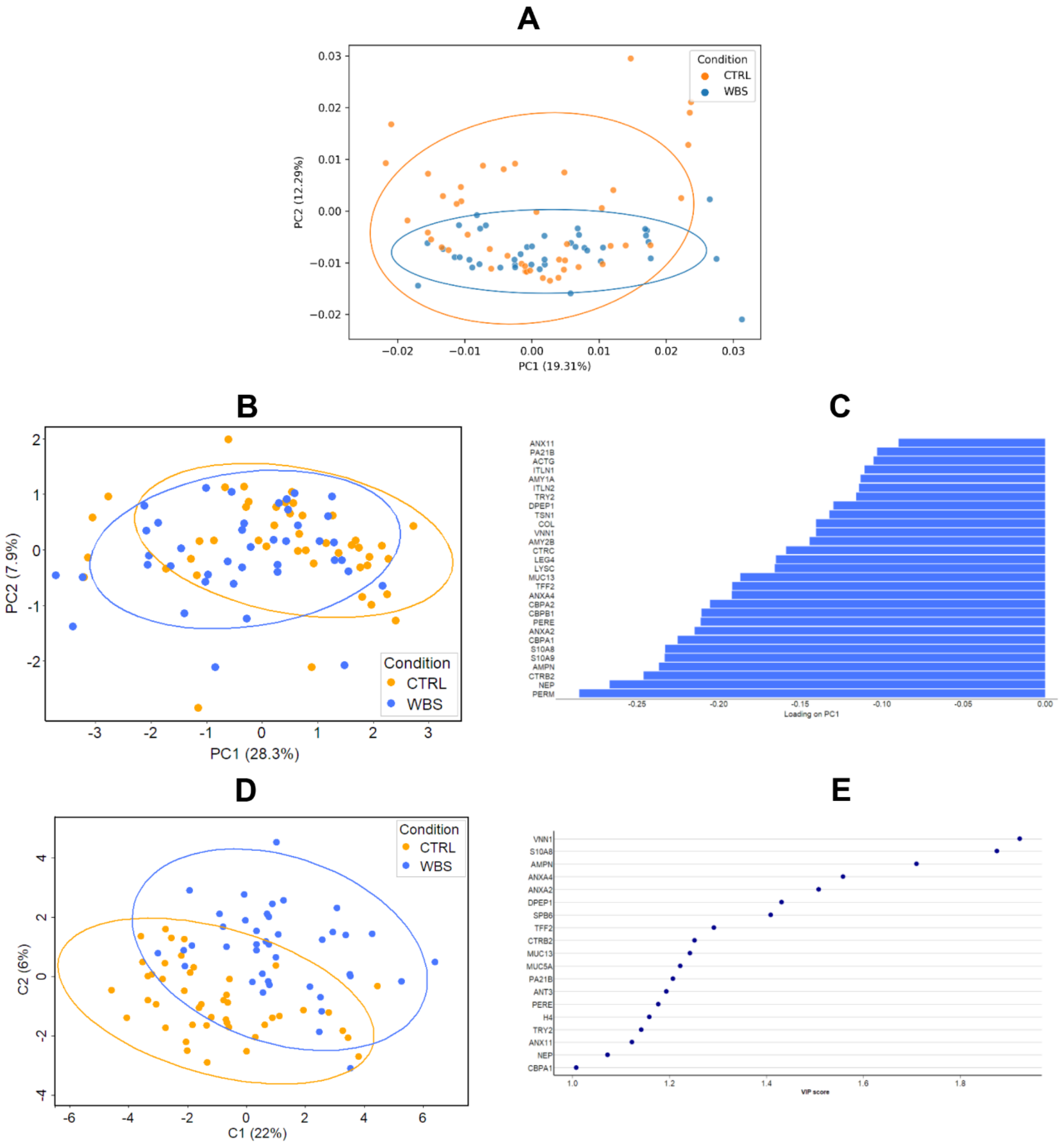

Supplementary Figure 9. Sketch of gut microbiota metabolic pathways from carbohydrate transport, degradation to the production of short-chain fatty acids. Created with BioRender.com.

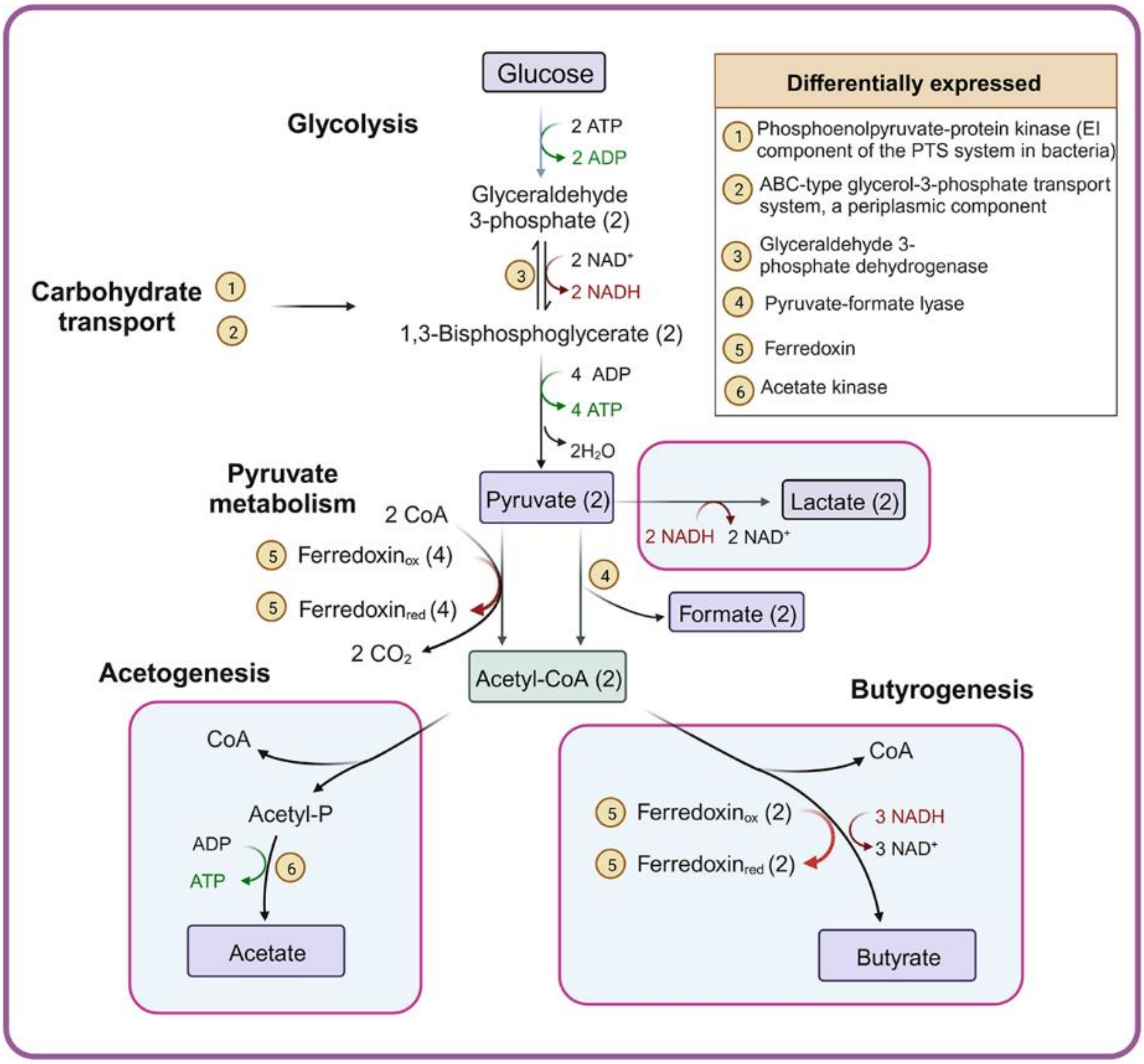

Supplementary Table

Supplementary Table 1. Number of Protein Groups (PGs), and their associated COG categories, which contributed the most to the actual separation of WBSs from CTRLs.

COG category	# PGs		
	Multivariate analysis		Univariate analysis
	PCA	PLS-DA	
Amino acid transport and metabolism [E]	2	2	10
Carbohydrate transport and metabolism [G]	17	3	22
Cell wall/membrane/envelope biogenesis [M]	1	1	5
Energy production and conversion [C]	8	5	20
Transcription [K]	1	2	2
Translation, ribosomal structure and biogenesis [J]	7	4	10

Supplementary Files

Supplementary File 1. Metadata associated to WBS patients and healthy control subjects; statistic associated to age and sex distribution of the sample cohort.

Supplementary File 2. List of identified and quantified bacteria protein groups with functional and taxonomic assignments; PCA loadings and PLS-DA VIPs.

Supplementary File 3. List of identified and quantified human protein groups; PCA loadings and PLS-DA VIPs.

Supplementary File 4. Statistic associated to the comparisons between WBS and CTRL bacteria protein groups.

Supplementary File 5. Analysis of GM metaproteome of WBS patients stratified by clinical features.

Supplementary File 6. Metascape enriched terms associated to human identified protein groups.
