# Supplementary material for: Williams–Beuren syndrome shapes the gut microbiota metaproteome
Source: Sci Rep. 2023 Nov 3;13:18963. doi: 10.1038/s41598-023-46052-9 (PMC10624682; doi:10.1038/s41598-023-46052-9)
Supplement: Supplementary file 2 — Supplementary File 1. [file 41598_2023_46052_MOESM2_ESM.pdf]

WBS metadata

# Metaproteomic sample	Code	Sex	Age (years)	Weight (kg)	Height (cm)	BMI	Omnivorous diet yes = 0, no = 1*	Obesity no = 0, yes = 1	GERD** no = 0, yes = 1	Constipation no = 0, yes = 1	Diarrhea no = 0, yes = 1	Abdominal pain 0=no, 1=yes	Cardiovascular abnormalities 0 = no, 1 = yes	Hypertension 0 = no, 1 = yes	Hypothyroidism 0 = no, 1 = yes	Facial dysmorphism 0 = no, 1 = yes	Motor and/or cognitive impairments 0= no, 1 = yes	
1	WBS-1	F	10	46.4	141.3	23.24	0	0	0	0	0	0	0	0	1	1	1	
2	WBS-2	F	29	72	154.5	30.16	0	1	0	0	0	0	1	1	1	1	1	
3	WBS-3	F	16	61	145.7	28.73	0	0	0	1	0	1	1	1	1	1	1	
4	WBS-4	F	28	88.8	151.8	38.54	0	1	0	0	0	0	1	1	0	1	1	
5	WBS-5	F	1	7.1	67	15.82	1	0	1	1	0	0	1	0	0	1	0	
6	WBS-6	F	2	13	85	17.99	0	0	0	1	0	0	1	0	0	1	0	
7	WBS-7	M	22	75.2	167	26.96	0	0	1	1	0	0	1	1	0	1	0	
8	WBS-8	F	28	55.3	149	24.91	0	0	0	0	0	0	1	1	0	1	1	
9	WBS-9	F	22	58.5	147.3	26.96	0	0	0	0	0	0	1	0	1	1	0	
10	WBS-10	M	18	97	170	33.56	0	1	0	0	0	0	0	1	0	1	1	
11	WBS-11	M	8	24	122.7	15.94	0	0	0	0	1	1	0	0	1	1	0	
12	WBS-12	F	22	71	149	31.98	0	1	0	0	0	0	0	1	1	1	1	
13	WBS-13	F	17	46.1	154	19.44	1	0	1	0	0	0	0	1	0	0	0	
14	WBS-15	M	11	42.6	150	18.93	0	0	0	0	1	1	1	0	0	1	1	
15	WBS-17	M	27	62.5	167	22.41	1	0	0	0	1	0	1	1	0	1	1	
16	WBS-18	F	10	28.5	131.9	16.38	1	0	0	0	0	0	0	0	0	1	1	
17	WBS-20	M	13	101	157	40.98	0	1	0	0	0	0	0	1	1	1	1	
18	WBS-21	M	17	62	156	25.48	1	0	1	0	0	1	1	1	0	1	1	
19	WBS-22	M	28	68	169	23.81	0	0	1	1	0	1	0	1	0	1	1	
20	WBS-23	M	9	29	137	15.45	0	0	0	0	0	0	0	1	0	1	1	
21	WBS-24	F	9	40	141.6	19.95	0	0	0	0	0	0	1	0	0	0	1	
22	WBS-25	M	5	11	96	11.94	0	0	0	0	1	0	1	0	0	1	1	
23	WBS-26	F	18	41.5	147.7	19.02	0	0	0	1	0	0	0	0	0	1	0	
24	WBS-27	F	12	32.4	137	17.26	0	0	0	1	0	0	0	0	0	1	0	
25	WBS-28	M	13	46.5	146.7	21.61	0	0	0	0	1	0	1	0	0	1	1	
26	WBS-29	F	13	50	152	21.64	0	0	0	1	0	0	0	0	0	1	1	
27	WBS-30	F	3	13	90.9	15.73	0	0	0	0	0	0	1	0	0	1	0	
28	WBS-32	F	39	68.5	145	32.58	0	1	0	0	0	0	0	0	0	1	0	
29	WBS-33	F	7	18	114.4	13.75	0	0	0	0	0	0	0	0	0	1	1	
30	WBS-34	M	6	23	118.8	16.30	1	0	0	0	0	0	1	1	0	1	1	
31	WBS-35	F	13	51	144.5	24.42	0	1	1	0	0	0	1	0	1	1	1	
32	WBS-36	F	10	20.8	140	10.61	0	0	0	0	0	0	1	0	0	1	1	
33	WBS-37	F	14	36.5	146.5	17.01	1	0	0	0	1	0	0	0	0	1	1	
34	WBS-38	F	19	72	150	32.00	0	1	0	1	0	0	1	1	0	1	1	
35	WBS-39	M	4	16	106	14.24	0	0	0	0	0	0	1	1	0	1	0	
36	WBS-40	M	7	19.1	125.2	12.18	1	0	1	0	0	0	0	0	0	1	1	
37	WBS-41	M	30	45	162	17.15	0	0	0	0	0	0	1	1	0	1	1	
38	WBS-42	F	14	41	153.2	17.47	0	0	1	0	0	0	0	0	0	1	1	
39	WBS-43	M	6	20	110.4	16.41	1	0	0	0	0	0	1	0	0	1	0	
40	WBS-44	F	9	37	141	18.61	0	0	1	1	0	0	0	0	0	0	0	
41	WBS-45	M	1	11.6	79	18.59	0	0	1	0	0	1	1	0	0	0	0	
		**mean**	**14**	**44.46**	**137.08**	**21.61**	**9**	**8**	**10**	**10**	**6**	**6**	**23**	**17**	**8**	**37**	**27**	**sum**
		**s.d.**	**9**	**24.54**	**25.27**	**7.30**	**22%**	**20%**	**24%**	**24%**	**15%**	**15%**	**56%**	**41%**	**20%**	**90%**	**66%**	**%**

*** = WILL-18 AND 21 were celiac**

GERD = Gastro-esophageal reflux disease**

CTRL metadata

# Metaproteomic sample	Code	Sex	Age (years)
1	CTRL-1	F	13
2	CTRL-2	M	13
3	CTRL-3	F	13
4	CTRL-4	F	13
5	CTRL-6	M	12
6	CTRL-7	F	12
7	CTRL-11	F	11
8	CTRL-12	F	11
9	CTRL-14	M	11
10	CTRL-15	F	11
11	CTRL-16	M	10
12	CTRL-17	M	10
13	CTRL-19	F	10
14	CTRL-20	F	10
15	CTRL-21	M	10
16	CTRL-25	F	9
17	CTRL-28	M	8
18	CTRL-29	M	8
19	CTRL-30	F	8
20	CTRL-32	M	7
21	CTRL-33	F	7
22	CTRL-35	M	7
23	CTRL-36	M	7
24	CTRL-37	F	6
25	CTRL-38	F	6
26	CTRL-40	M	5
27	CTRL-47	F	2
28	CTRL-48	M	4
29	CTRL-49	F	14
30	CTRL-69	M	17
31	CTRL-70	F	16
32	CTRL-71	F	27
33	CTRL-72	M	42
34	CTRL-73	F	33
35	CTRL-74	M	32
36	CTRL-75	F	31
37	CTRL-76	M	40
38	CTRL-77	M	41
39	CTRL-78	M	1
40	CTRL-79	F	1
41	CTRL-80	F	2
42	CTRL-81	M	13
43	CTRL-82	M	14
44	CTRL-83	M	14
45	CTRL-84	F	15
		**mean**	**13**
		**s.d.**	**10**

Age, gender and statistic

Age Group	Age	# WBS	# CTRL
**0**	years < 5	5	5
**1**	5 ≤ years < 13	22	14
**2**	13 ≤ years < 18	11	9
**3**	18 ≤ years ≤ 42	7	13

Variable	Kruskal-Wallis CTRL vs WBS	Kruskal-Wallis p-value CTRL vs WBS	ANOVA CTRL vs WBS	ANOVA p-value CTRL vs WBS	Mann-Whitney CTRL vs WBS	Mann-Whitney p-value CTRL vs WBS
**Age**	0.6769	0.4107	0.1821	0.6707	827.5000	0.4131
**Gender**	0.4717	0.4922	0.4688	0.4954	854.0000	0.4954
**Age Group**	1.7706	0.1833	1.8786	0.1741	776.5000	0.1848

Variable	Group	Binom Test p-value
Gender	WBS	0.3489
Gender	CTRL	1.0000

WBS patients clinical stratification

Group	A. Diet	Celiac disease	B. Obesity	Weight status	C. Gastrointestinal (GI) symptoms<br>Gastroesophageal reflux (GERD)	Diarrhea	Constipation	Abdominal pain	Cardiovascular abnormalities	Hypertension
	omnivorous = 0, food elimination for intolerances or predilition = 1	no = 0, yes = 1	no = 0, yes = 1	normalweight = 0, obesity = 1, overweight = 2, underweight = 3	no = 0, yes = 1	no = 0, yes=1	no = 0, yes = 1	no = 0, yes = 1	no = 0, yes = 1	no = 0, yes = 1
0	32	39	33	12 (29%)	31	35	31	35	18	24
1	9	2	8	8 (20%)	10	6	10	6	23	17
2				4 (10%)						
3				17 (17%)						
**Sum**	41	41	41	41	41	41	41	41	41	41

C. Gastrointestinal (GI) symptoms

	Absence of GI symptoms	One functional GI symptoms		Two functional GI symptoms		Three functional GI symptoms		Four functional GI symptoms		≥ one functional GI symptoms	≥ two functional GI symptoms
**# patients**	19	13		8		1		0		22	9
**% respect total**	46	32		20		2		0		54	22
		**List of symptoms**	**# patients**	**List of symptoms**	**# patients**	**List of symptoms**	**# patients**	**List of symptoms**	**# patients**		
		GERD	4	GERD + Constipation	3	GERD + Constipation + Abdominal pain	1	GERD	0		
		Constipation	5	GERD + Abdominal pain	2			Constipation	0		
		Diarrhea	4	Constipation + Abdominal pain	1			Diarrhea	0		
		Abdominal pain	0	Diarrhea + Abdominal pain	2			Abdominal pain	0		
		**Sum**	**13**	**Sum**	8	**Sum**	1	**Sum**	0		
