# Supplementary material for: Williams–Beuren syndrome shapes the gut microbiota metaproteome
Source: Sci Rep. 2023 Nov 3;13:18963. doi: 10.1038/s41598-023-46052-9 (PMC10624682; doi:10.1038/s41598-023-46052-9)
Supplement: Supplementary file 3 — Supplementary File 2. [file 41598_2023_46052_MOESM3_ESM.pdf]

638 Identified Protein Group

N	Protein Group ID	Leading razor protein accession	COG accession	COG name	COG category	KEGG name	LCA	Rank	Phylum	Class	Order	Family	Genus	Species	Log_{10} (WBS/CTRL)	t-test p-value WBS/CTRL	Significance WBS/CTRL
1	112	206672.BL0597	COG0058	Glucan phosphorylase	Carbohydrate transport and metabolism [G]	Starch and sucrose metabolism	Bifidobacterium	Genus	Actinobacteria	Actinobacteria	Bifidobacteriales	Bifidobacteriaceae	Bifidobacterium		0.2153	0.0212	*
2	115	206672.BL0707	COG0126	3-phosphoglycerate kinase	Carbohydrate transport and metabolism [G]	Glycolysis / Gluconeogenesis	Bifidobacterium longum	Species	Actinobacteria	Actinobacteria	Bifidobacteriales	Bifidobacteriaceae	Bifidobacterium	Bifidobacterium longum	0.0485	0.6503	
3	118	206672.BL0951	COG1882	Pyruvate-formate lyase	Energy production and conversion [C]	Pyruvate metabolism	Bifidobacterium	Genus	Actinobacteria	Actinobacteria	Bifidobacteriales	Bifidobacteriaceae	Bifidobacterium		0.0552	0.5906	
4	122	206672.BL0988	COG0469	Pyruvate kinase	Carbohydrate transport and metabolism [G]	Glycolysis / Gluconeogenesis	Bifidobacterium	Genus	Actinobacteria	Actinobacteria	Bifidobacteriales	Bifidobacteriaceae	Bifidobacterium		-0.0081	0.9507	
5	135	206672.BL1722	COG0517	CBS domain	Signal transduction mechanisms [T]	Purine metabolism	Bifidobacterium	Genus	Actinobacteria	Actinobacteria	Bifidobacteriales	Bifidobacteriaceae	Bifidobacterium		0.0781	0.2115	
6	280	367928.BAD_0348	COG0203	Ribosomal protein L17	Translation, ribosomal structure and biogenesis [J]	Ribosome	Bifidobacterium adolescentis	Species	Actinobacteria	Actinobacteria	Bifidobacteriales	Bifidobacteriaceae	Bifidobacterium	Bifidobacterium adolescentis	-0.0869	0.0766	
7	284	367928.BAD_0378	COG0588	Phosphoglycerate mutase (BPG-dependent)	Carbohydrate transport and metabolism [G]	Glycolysis / Gluconeogenesis	Bifidobacterium	Genus	Actinobacteria	Actinobacteria	Bifidobacteriales	Bifidobacteriaceae	Bifidobacterium		-0.1967	0.0267	*
8	286	367928.BAD_0450	COG0517	CBS domain	Signal transduction mechanisms [T]	Purine metabolism	Bifidobacterium adolescentis	Species	Actinobacteria	Actinobacteria	Bifidobacteriales	Bifidobacteriaceae	Bifidobacterium	Bifidobacterium adolescentis	-0.1795	0.0887	
9	340	391904.BLIJ_0145	COG0443	Molecular chaperone DnaK (HSP70)	Posttranslational modification, protein turnover, chaperones [O]	RNA degradation	Bifidobacterium	Genus	Actinobacteria	Actinobacteria	Bifidobacteriales	Bifidobacteriaceae	Bifidobacterium		-0.0018	0.9880	
10	507	411483.FAEPRAA2165_01405	COG0330	Regulator of protease activity HflC, stomatin/prohibitin superfamily	Posttranslational modification, protein turnover, chaperones [O]	NAN	Faecalibacterium prausnitzii	Species	Firmicutes	Clostridia	Clostridiales	Ruminococcaceae	Faecalibacterium	Faecalibacterium prausnitzii	0.0032	0.9616	
11	525	411485.FAEPRAM212_01761	COG1145	Ferredoxin	Energy production and conversion [C]	Glycolysis / Gluconeogenesis	Faecalibacterium prausnitzii	Species	Firmicutes	Clostridia	Clostridiales	Ruminococcaceae	Faecalibacterium	Faecalibacterium prausnitzii	-0.2203	0.0859	
12	552	411903.COLAER_00059	COG1080	Phosphoenolpyruvate-protein kinase (PTS system EI component in bacteria)	Carbohydrate transport and metabolism [G]	Fructose and mannose metabolism	Collinsella	Genus	Actinobacteria	Coriobacteriia	Coriobacteriales	Coriobacteriaceae	Collinsella		0.1427	0.0295	*
13	572	411903.COLAER_02212	COG4166	ABC-type oligopeptide transport system, periplasmic component(3057)	Amino acid transport and metabolism [E]	ABC transporters	Collinsella aerofaciens	Species	Actinobacteria	Coriobacteriia	Coriobacteriales	Coriobacteriaceae	Collinsella	Collinsella aerofaciens	0.2769	0.0037	**
14	606	435590.BVU_0563	COG4771	Outer membrane receptor for ferrienterochelin and colicins(16693)	Inorganic ion transport and metabolism [P]	NAN	Bacteroides	Genus	Bacteroidetes	Bacteroidia	Bacteroidales	Bacteroidaceae	Bacteroides		0.2243	0.1487	
15	708	457395.BSBG_00304	COG3637	Opacity protein and related surface antigens(2204)	Cell wall/membrane/envelope biogenesis [M]	NAN	Bacteroides	Genus	Bacteroidetes	Bacteroidia	Bacteroidales	Bacteroidaceae	Bacteroides		0.0709	0.5154	
16	789	469594.HMPREF0177_01181	COG0221	Inorganic pyrophosphatase	Inorganic ion transport and metabolism [P]	Oxidative phosphorylation	Bifidobacterium	Genus	Actinobacteria	Actinobacteria	Bifidobacteriales	Bifidobacteriaceae	Bifidobacterium		-0.0527	0.4910	
17	923	483217.BACDOR_00668	COG3525	N-acetyl-beta-hexosaminidase	Carbohydrate transport and metabolism [G]	NAN	Bacteroides	Genus	Bacteroidetes	Bacteroidia	Bacteroidales	Bacteroidaceae	Bacteroides		0.1447	0.0954	
18	939	515619.EUBREC_1472	COG1145	Ferredoxin	Energy production and conversion [C]	Glycolysis / Gluconeogenesis	Clostridiales	Order	Firmicutes	Clostridia	Clostridiales				-0.4149	0.0086	**
19	960	518634.BIFBRE_03753	COG3957	Phosphoketolase	Carbohydrate transport and metabolism [G]	Pentose phosphate pathway	Bifidobacterium	Genus	Actinobacteria	Actinobacteria	Bifidobacteriales	Bifidobacteriaceae	Bifidobacterium		0.0190	0.8122	
20	1041	MH0203_GL0115820	COG0093	Ribosomal protein L14	Translation, ribosomal structure and biogenesis [J]	Ribosome	Bifidobacterium	Genus	Actinobacteria	Actinobacteria	Bifidobacteriales	Bifidobacteriaceae	Bifidobacterium		-0.0613	0.3345	
21	1139	537937.BLIG_01296	COG4166	ABC-type oligopeptide transport system, periplasmic component	Amino acid transport and metabolism [E]	ABC transporters	Bifidobacterium	Genus	Actinobacteria	Actinobacteria	Bifidobacteriales	Bifidobacteriaceae	Bifidobacterium		0.1433	0.1833	
22	1188	548480.HMPREF0175_0263	COG1070	Sugar (pentulose or hexulose) kinase	Carbohydrate transport and metabolism [G]	Pentose phosphate pathway	Bifidobacterium	Genus	Actinobacteria	Actinobacteria	Bifidobacteriales	Bifidobacteriaceae	Bifidobacterium		0.1174	0.1464	
23	1264	V1.CD21-4_GL0001620			NAN	Arginine and proline metabolism	Clostridiales	Order	Firmicutes	Clostridia	Clostridiales				0.0695	0.1945	
24	1349	566552.BIFCAT_00987	COG0282	Acetate kinase	Energy production and conversion [C]	Taurine and hypotaurine metabolism	Bifidobacterium	Genus	Actinobacteria	Actinobacteria	Bifidobacteriales	Bifidobacteriaceae	Bifidobacterium		0.0006	0.9951	
25	1438	657314.CK5_28620	COG2759	Formyltetrahydrofolate synthetase	Nucleotide transport and metabolism [F]	One carbon pool by folate	Blautia obeum	Species	Firmicutes	Clostridia	Clostridiales	Lachnospiraceae	Blautia	Blautia obeum	-0.1056	0.1305	
26	1460	657323.CK1_21680	COG4213	ABC-type xylose transport system, periplasmic component	Carbohydrate transport and metabolism [G]	ABC transporters	Clostridiales	Order	Firmicutes	Clostridia	Clostridiales				-0.0523	0.4383	
27	1633	759350.BLJ_0360	COG0228	Ribosomal protein S16	Translation, ribosomal structure and biogenesis [J]	Ribosome	Bifidobacterium	Genus	Actinobacteria	Actinobacteria	Bifidobacteriales	Bifidobacteriaceae	Bifidobacterium		0.0567	0.5208	
28	1991	BGI-33A_GL0073034	COG0822	NifU homolog involved in Fe-S cluster formation	Posttranslational modification, protein turnover, chaperones [O]	NAN	Ruminococcus bromii	Species	Firmicutes	Clostridia	Clostridiales	Ruminococcaceae	Ruminococcus	Ruminococcus bromii	0.0378	0.7016	
29	2257	DLF012_GL0017930	COG2873	O-acetylhomoserine/O-acetylserine sulfhydrylase, pyridoxal phosphate-dependent	Amino acid transport and metabolism [E]	Cysteine and methionine metabolism	Bifidobacterium breve	Species	Actinobacteria	Actinobacteria	Bifidobacteriales	Bifidobacteriaceae	Bifidobacterium	Bifidobacterium breve	-0.0982	0.2413	
30	2355	DLF012_GL0039573	COG1653	ABC-type glycerol-3-phosphate transport system, periplasmic component	Carbohydrate transport and metabolism [G]	ABC transporters	Clostridiales	Order	Firmicutes	Clostridia	Clostridiales				-0.0826	0.2693	
31	2394	DLF013_GL0024384	COG0054	6,7-dimethyl-8-ribityllumazine synthase (Riboflavin synthase beta chain)	Coenzyme transport and metabolism [H]	Riboflavin metabolism	Clostridiales	Order	Firmicutes	Clostridia	Clostridiales				0.0581	0.3714	
32	2592	DLM008_GL0027121	COG0462	Phosphoribosylpyrophosphate synthetase	Nucleotide transport and metabolism [F]	Pentose phosphate pathway	Ruminococcus bromii	Species	Firmicutes	Clostridia	Clostridiales	Ruminococcaceae	Ruminococcus	Ruminococcus bromii	0.0147	0.8383	
33	2699	DLM013_GL0037207	COG1196	Chromosome segregation ATPase	Cell cycle control, cell division, chromosome partitioning [D]	NAN	Bacteroides dorei	Species	Bacteroidetes	Bacteroidia	Bacteroidales	Bacteroidaceae	Bacteroides	Bacteroides dorei	0.4774	0.0114	*
34	2780	DLM016_GL0028957	COG1592	Rubrerythrin	Energy production and conversion [C]	NAN	Ruminococcus bromii	Species	Firmicutes	Clostridia	Clostridiales	Ruminococcaceae	Ruminococcus	Ruminococcus bromii	-0.0539	0.5734	
35	2802	DLM017_GL0065099	COG1088	dTDP-D-glucose 4,6-dehydratase	Cell wall/membrane/envelope biogenesis [M]	Amino sugar and nucleotide sugar metabolism	Clostridiales	Order	Firmicutes	Clostridia	Clostridiales				-0.0253	0.7508	
36	2809	DLM018_GL0016638	COG0092	Ribosomal protein S3	Translation, ribosomal structure and biogenesis [J]	Ribosome	Bifidobacterium	Genus	Actinobacteria	Actinobacteria	Bifidobacteriales	Bifidobacteriaceae	Bifidobacterium		0.0426	0.6627	
37	2834	DLM018_GL0063411	COG1653	ABC-type glycerol-3-phosphate transport system, periplasmic component	Carbohydrate transport and metabolism [G]	ABC transporters	Faecalibacterium prausnitzii	Species	Firmicutes	Clostridia	Clostridiales	Ruminococcaceae	Faecalibacterium	Faecalibacterium prausnitzii	0.2004	0.0760	

**38**	2839	DLM018_GL0068485	COG1866	Phosphoenolpyruvate carboxykinase, ATP-dependent	Energy production and conversion [C]	Glycolysis / Gluconeogenesis	[Eubacterium] rectale	Species	Firmicutes	Clostridia	Clostridiales	Lachnospiraceae		[Eubacterium] rectale	0.1126	0.1635	
**39**	2842	T2D-31A_GL0073439	COG0330	Regulator of protease activity HflC, stomatin/prohibitin superfamily	Posttranslational modification, protein turnover, chaperones [O]	NAN		Genus	Actinobacteria	Actinobacteria	Bifidobacteriales	Bifidobacteriaceae	Bifidobacterium		0.0811	0.2454	
**40**	3357	DOM013_GL0034020	COG0183	Acetyl-CoA acetyltransferase	Lipid transport and metabolism [I]	Fatty acid degradation	Faecalibacterium prausnitzii	Species	Firmicutes	Clostridia	Clostridiales	Ruminococcaceae	Faecalibacterium	Faecalibacterium prausnitzii	-0.1339	0.0285	*
**41**	3872	HT14A_GL0040767	COG0804	Urease alpha subunit	Amino acid transport and metabolism [E]	Purine metabolism	Firmicutes	Phylum	Firmicutes						0.044518339	0.510464557	
**42**	4187	MH0001_GL0010989	COG0050	Translation elongation factor EF-Tu, a GTPase	Translation, ribosomal structure and biogenesis [J]	Plant-pathogen interaction	Prevotella copri	Species	Bacteroidetes	Bacteroidia	Bacteroidales	Prevotellaceae	Prevotella	Prevotella copri	-0.013691868	0.899771715	
**43**	4272	MH0001_GL0015313	COG1145	Ferredoxin	Energy production and conversion [C]	Glycolysis / Gluconeogenesis	Roseburia faecis	Species	Firmicutes	Clostridia	Clostridiales	Lachnospiraceae	Roseburia	Roseburia faecis	-0.242071665	0.009329289	**
**44**	4357	MH0001_GL0019923	COG1077	Actin-like ATPase involved in cell morphogenesis	Cell cycle control, cell division, chromosome partitioning [D]	NAN	Roseburia faecis	Species	Firmicutes	Clostridia	Clostridiales	Lachnospiraceae	Roseburia	Roseburia faecis	-0.005748639	0.923481695	
**45**	4411	MH0002_GL0000742	COG0035	Uracil phosphoribosyltransferase	Nucleotide transport and metabolism [F]	Pyrimidine metabolism	Subdoligranulum variabile	Species	Firmicutes	Clostridia	Clostridiales	Ruminococcaceae	Subdoligranulum	Subdoligranulum variabile	-0.102731419	0.031288087	*
**46**	4426	MH0002_GL0001930	COG0050	Translation elongation factor EF-Tu, a GTPase	Translation, ribosomal structure and biogenesis [J]	Plant-pathogen interaction	Clostridiales	Species	Firmicutes	Clostridia	Clostridiales	Ruminococcaceae	Subdoligranulum	Subdoligranulum variabile	-0.336413211	3.16659E-05	***
**47**	4427	MH0002_GL0001931	COG0480	Translation elongation factor EF-G, a GTPase	Translation, ribosomal structure and biogenesis [J]	NAN	Clostridiales	Species	Firmicutes	Clostridia	Clostridiales	Clostridiaceae	Butyricicoccus	Butyricicoccus pullicaecorum	-0.320181384	0.000937946	***
**48**	4478	MH0002_GL0005640	COG0112	Glycine/serine hydroxymethyltransferase	Amino acid transport and metabolism [E]	Glycine, serine and threonine metabolism	Subdoligranulum variabile	Species	Firmicutes	Clostridia	Clostridiales	Ruminococcaceae	Subdoligranulum	Subdoligranulum variabile	0.012552563	0.812683185	
**49**	4487	MH0002_GL0005969	COG0151	Phosphoribosylamine-glycine ligase	Nucleotide transport and metabolism [F]	Purine metabolism	Clostridiales	Order	Firmicutes	Clostridia	Clostridiales				-0.022537577	0.616070552	
**50**	4489	MH0002_GL0006301			NAN	NAN	Roseburia faecis	Species	Firmicutes	Clostridia	Clostridiales	Lachnospiraceae	Roseburia	Roseburia faecis	-0.236612904	0.10089168	
**51**	4512	MH0002_GL0008497	COG2025	Electron transfer flavoprotein, alpha subunit	Energy production and conversion [C]	Nitrogen metabolism	Subdoligranulum variabile	Species	Firmicutes	Clostridia	Clostridiales	Ruminococcaceae	Subdoligranulum	Subdoligranulum variabile	-0.226721169	0.008450267	**
**52**	4569	MH0002_GL0013621			NAN	NAN	Alistipes putredinis	Species	Bacteroidetes	Bacteroidia	Bacteroidales	Rikenellaceae	Alistipes	Alistipes putredinis	0.111650134	0.040030495	*
**53**	4632	MH0002_GL0018942	COG0085	DNA-directed RNA polymerase, beta subunit/140 kD subunit	Transcription [K]	Purine metabolism	Bacteria	Species	Actinobacteria	Coriobacteriia	Coriobacteriales	Coriobacteriaceae	Coriobacterium	Coriobacterium glomerans	-0.192341616	0.000877222	***
**54**	4666	MH0002_GL0021758	COG0138	AICAR transformylase/IMP cyclohydrolase PurH	Nucleotide transport and metabolism [F]	Purine metabolism	Clostridiales	Order	Firmicutes	Clostridia	Clostridiales				0.059059508	0.358417857	
**55**	4706	MH0002_GL0025398	COG0086	DNA-directed RNA polymerase, beta' subunit/160 kD subunit	Transcription [K]	Purine metabolism	Clostridiales	Order	Firmicutes	Clostridia	Clostridiales				-0.144865208	0.088782752	
**56**	4746	MH0002_GL0027835	COG3968	Glutamine synthetase type III	Amino acid transport and metabolism [E]	Alanine, aspartate and glutamate metabolism	Ruminococcaceae	Species	Firmicutes	Clostridia	Clostridiales	Ruminococcaceae	Subdoligranulum	Subdoligranulum variabile	-0.215527676	0.034350749	*
**57**	4759	MH0002_GL0028993			NAN	NAN	Bacteria	Superkingdom							0.039029446	0.681806091	
**58**	4801	MH0002_GL0031887	COG0166	Glucose-6-phosphate isomerase	Carbohydrate transport and metabolism [G]	Glycolysis / Gluconeogenesis	Ruminococcaceae	Species	Firmicutes	Clostridia	Clostridiales	Ruminococcaceae	Subdoligranulum	Subdoligranulum variabile	-0.125949795	0.050469828	
**59**	4811	MH0002_GL0032818	COG3842	ABC-type Fe3+/spermidine/putrescine transport systems, ATPase components	Amino acid transport and metabolism [E]	ABC transporters	Clostridiales	Order	Firmicutes	Clostridia	Clostridiales				-0.025445766	0.80941106	
**60**	4862	MH0002_GL0037571	COG0280	Phosphotransacetylase	Energy production and conversion [C]	Taurine and hypotaurine metabolism	Subdoligranulum variabile	Species	Firmicutes	Clostridia	Clostridiales	Ruminococcaceae	Subdoligranulum	Subdoligranulum variabile	-0.056623028	0.349359798	
**61**	4868	MH0002_GL0038227	COG2885	Outer membrane protein OmpA and related peptidoglycan-associated (lipo)proteins	Cell wall/membrane/envelope biogenesis [M]	NAN	Alistipes putredinis	Species	Bacteroidetes	Bacteroidia	Bacteroidales	Rikenellaceae	Alistipes	Alistipes putredinis	0.084846485	0.207428387	
**62**	4871	MH0002_GL0038347	COG0297	Glycogen synthase	Carbohydrate transport and metabolism [G]	Galactose metabolism	Ruminococcaceae	Species	Firmicutes	Clostridia	Clostridiales	Ruminococcaceae	Subdoligranulum	Subdoligranulum variabile	-0.191849554	0.011724592	*
**63**	4872	MH0002_GL0038348	COG0448	ADP-glucose pyrophosphorylase	Carbohydrate transport and metabolism [G]	Starch and sucrose metabolism	Clostridiales	Species	Firmicutes	Clostridia	Clostridiales	Lachnospiraceae		Lachnospiraceae bacterium COE1	-0.051670792	0.435257119	
**64**	4873	MH0002_GL0038349	COG0448	ADP-glucose pyrophosphorylase	Carbohydrate transport and metabolism [G]	Starch and sucrose metabolism	Clostridiales	Order	Firmicutes	Clostridia	Clostridiales				-0.14702525	0.078310371	
**65**	4889	MH0002_GL0039425	COG1362	Aspartyl aminopeptidase	Amino acid transport and metabolism [E]	NAN	[Eubacterium] rectale	Species	Firmicutes	Clostridia	Clostridiales	Lachnospiraceae		[Eubacterium] rectale	0.129203269	0.053264071	
**66**	4911	MH0002_GL0040561	COG4799	Acetyl-CoA carboxylase, carboxyltransferase component	Lipid transport and metabolism [I]	Fatty acid biosynthesis	Subdoligranulum variabile	Species	Firmicutes	Clostridia	Clostridiales	Ruminococcaceae	Subdoligranulum	Subdoligranulum variabile	-0.101736077	0.171871664	
**67**	4914	MH0002_GL0040734	COG1866	Phosphoenolpyruvate carboxykinase, ATP-dependent	Energy production and conversion [C]	Glycolysis / Gluconeogenesis	Lachnospiraceae	Family	Firmicutes	Clostridia	Clostridiales	Lachnospiraceae			-0.062147812	0.300169387	
**68**	4915	MH0002_GL0040735	COG3957	Phosphoketolase	Carbohydrate transport and metabolism [G]	Pentose phosphate pathway	Mycobacterium bohemicum	Species	Actinobacteria	Actinobacteria	Corynebacteriales	Mycobacteriaceae	Mycobacterium	Mycobacterium bohemicum	-0.45247304	4.35888E-05	***
**69**	4916	MH0002_GL0040736	COG2407	L-fucose isomerase or related protein	Carbohydrate transport and metabolism [G]	Fructose and mannose metabolism	Subdoligranulum variabile	Species	Firmicutes	Clostridia	Clostridiales	Ruminococcaceae	Subdoligranulum	Subdoligranulum variabile	-0.509915581	2.28399E-05	***
**70**	4977	MH0002_GL0044927	COG0522	Ribosomal protein S4 or related protein	Translation, ribosomal structure and biogenesis [J]	Ribosome biogenesis in eukaryotes	Subdoligranulum variabile	Species	Firmicutes	Clostridia	Clostridiales	Ruminococcaceae	Subdoligranulum	Subdoligranulum variabile	-0.038552197	0.504524809	
**71**	4995	MH0002_GL0047744			NAN	NAN	Bacteria	Superkingdom							0.241466998	0.000862361	***
**72**	5031	MH0002_GL0050527	COG0081	Ribosomal protein L1	Translation, ribosomal structure and biogenesis [J]	Ribosome	Ruminococcaceae	Species	Firmicutes	Clostridia	Clostridiales	Ruminococcaceae	Faecalibacterium	Faecalibacterium prausnitzii	-0.172432575	0.015026248	*
**73**	5035	MH0002_GL0050697	COG1726	Na+-transporting NADH:ubiquinone oxidoreductase, subunit NqrA	Energy production and conversion [C]	NAN	Bacteroides	Genus	Bacteroidetes	Bacteroidia	Bacteroidales	Bacteroidaceae	Bacteroides		0.257016943	0.000601208	***
**74**	5041	MH0002_GL0050870	COG1048	Aconitase A	Energy production and conversion [C]	Citrate cycle (TCA cycle)	Clostridiales	Order	Firmicutes	Clostridia	Clostridiales				-0.181556636	0.008789114	**
**75**	5043	MH0002_GL0050951	COG1145	Ferredoxin	Energy production and conversion [C]	Glycolysis / Gluconeogenesis	Ruminococcaceae	Species	Firmicutes	Clostridia	Clostridiales	Ruminococcaceae	Faecalibacterium	Faecalibacterium prausnitzii	-0.280267717	0.010006572	*
**76**	5065	MH0002_GL0053021	COG0055	FoF1-type ATP synthase, beta subunit	Energy production and conversion [C]	Oxidative phosphorylation	Clostridiales	Order	Firmicutes	Clostridia	Clostridiales				-0.070608657	0.230894558	

**77**	5107	MH0002_GL0057193	COG1264	Phosphotransferase system IIB components	Carbohydrate transport and metabolism [G]	Glycolysis / Gluconeogenesis	Clostridiales	Order	Firmicutes	Clostridia	Clostridiales				-0.165371993	0.004915989	**
**78**	5172	MH0002_GL0063099	COG0423	Glycyl-tRNA synthetase (class II)	Translation, ribosomal structure and biogenesis [J]	Aminoacyl-tRNA biosynthesis	Clostridiales	Order	Firmicutes	Clostridia	Clostridiales				-0.109019864	0.069948245	
**79**	5239	MH0002_GL0070693	COG2182	Maltose-binding periplasmic protein MalE	Carbohydrate transport and metabolism [G]	ABC transporters	Clostridiales bacterium CHKCI001	Species	Firmicutes	Clostridia	Clostridiales			Clostridiales bacterium CHKCI001	-0.294359949	0.007489068	**
**80**	5254	MH0002_GL0071847	COG4206	Outer membrane cobalamin receptor protein	Coenzyme transport and metabolism [H]	NAN	Bacteroides	Genus	Bacteroidetes	Bacteroidia	Bacteroidales	Bacteroidaceae	Bacteroides		0.063801955	0.470001828	
**81**	5256	MH0002_GL0072081	COG0203	Ribosomal protein L17	Translation, ribosomal structure and biogenesis [J]	Ribosome	Clostridiales	Order	Firmicutes	Clostridia	Clostridiales				0.001406651	0.98249905	
**82**	5311	MH0002_GL0075003	COG1472	Periplasmic beta-glucosidase and related glycosidases	Carbohydrate transport and metabolism [G]	Cyanoamino acid metabolism	Bacteria	Superkingdom							-0.055867747	0.515800619	
**83**	5329	MH0002_GL0076888	COG0457	Tetratricopeptide (TPR) repeat	General function prediction only [R]	NAN	Bacteroides	Genus	Bacteroidetes	Bacteroidia	Bacteroidales	Bacteroidaceae	Bacteroides		0.401135025	0.000283216	***
**84**	5354	MH0003_GL0002731	COG0172	Seryl-tRNA synthetase	Translation, ribosomal structure and biogenesis [J]	Aminoacyl-tRNA biosynthesis	Ruminococcus bromii	Species	Firmicutes	Clostridia	Clostridiales	Ruminococcaceae	Ruminococcus	Ruminococcus bromii	-0.14504046	0.015664538	*
**85**	5385	MH0003_GL0005194	COG0050	Translation elongation factor EF-Tu, a GTPase	Translation, ribosomal structure and biogenesis [J]	Plant-pathogen interaction	Ruminococcus bromii	Species	Firmicutes	Clostridia	Clostridiales	Ruminococcaceae	Ruminococcus	Ruminococcus bromii	-0.152610691	0.151961817	
**86**	5386	MH0003_GL0005195	COG0480	Translation elongation factor EF-G, a GTPase	Translation, ribosomal structure and biogenesis [J]	NAN	Ruminococcus bromii	Species	Firmicutes	Clostridia	Clostridiales	Ruminococcaceae	Ruminococcus	Ruminococcus bromii	-0.146672968	0.161321587	
**87**	5388	MH0003_GL0005201	COG2182	Maltose-binding periplasmic protein MalE	Carbohydrate transport and metabolism [G]	ABC transporters	Ruminococcus bromii	Species	Firmicutes	Clostridia	Clostridiales	Ruminococcaceae	Ruminococcus	Ruminococcus bromii	0.048202822	0.684009006	
**88**	5421	MH0142_GL0051559	COG3033	Tryptophanase	Amino acid transport and metabolism [E]	Tyrosine metabolism	Firmicutes	Phylum	Firmicutes						-0.233622406	0.003743153	**
**89**	5428	MH0003_GL0009350	COG0057	Glyceraldehyde-3-phosphate dehydrogenase/erythrose-4-phosphate dehydrogenase	Carbohydrate transport and metabolism [G]	Glycolysis / Gluconeogenesis	Ruminococcus bromii	Species	Firmicutes	Clostridia	Clostridiales	Ruminococcaceae	Ruminococcus	Ruminococcus bromii	-0.067251827	0.363394807	
**90**	5429	MH0003_GL0009354	COG0297	Glycogen synthase	Carbohydrate transport and metabolism [G]	Galactose metabolism	Ruminococcus bromii	Species	Firmicutes	Clostridia	Clostridiales	Ruminococcaceae	Ruminococcus	Ruminococcus bromii	0.011726187	0.882514673	
**91**	5459	MH0003_GL0013354	COG0334	Glutamate dehydrogenase/leucine dehydrogenase	Amino acid transport and metabolism [E]	Alanine, aspartate and glutamate metabolism	Bacteroides	Genus	Bacteroidetes	Bacteroidia	Bacteroidales	Bacteroidaceae	Bacteroides		0.184009827	0.010840856	*
**92**	5484	MH0003_GL0018734	COG0366	Glycosidase	Carbohydrate transport and metabolism [G]	Galactose metabolism	Ruminococcus bromii	Species	Firmicutes	Clostridia	Clostridiales	Ruminococcaceae	Ruminococcus	Ruminococcus bromii	0.004409497	0.968291912	
**93**	5536	MH0003_GL0028371	COG1882	Pyruvate-formate lyase	Energy production and conversion [C]	Pyruvate metabolism	Ruminococcus bromii	Species	Firmicutes	Clostridia	Clostridiales	Ruminococcaceae	Ruminococcus	Ruminococcus bromii	0.01404151	0.879511464	
**94**	5553	MH0003_GL0030501	COG1454	Alcohol dehydrogenase, class IV	Energy production and conversion [C]	Glycolysis / Gluconeogenesis	Ruminococcus bromii	Species	Firmicutes	Clostridia	Clostridiales	Ruminococcaceae	Ruminococcus	Ruminococcus bromii	0.061219202	0.572855149	
**95**	5555	MH0003_GL0030511	COG0845	Multidrug efflux pump subunit AcrA (membrane-fusion protein)	Defense mechanisms [V]	Purine metabolism	Ruminococcus bromii	Species	Firmicutes	Clostridia	Clostridiales	Ruminococcaceae	Ruminococcus	Ruminococcus bromii	-0.055829019	0.48969723	
**96**	5557	MH0003_GL0030541	COG0047	Phosphoribosylformylglycinamidine (FGAM) synthase, glutamine amidotransferase domain	Nucleotide transport and metabolism [F]	Purine metabolism	Ruminococcus bromii	Species	Firmicutes	Clostridia	Clostridiales	Ruminococcaceae	Ruminococcus	Ruminococcus bromii	-0.148241312	0.075375228	
**97**	5650	MH0003_GL0042541	COG3637	Opacity protein and related surface antigens	Cell wall/membrane/envelope biogenesis [M]	NAN	Bacteroides	Genus	Bacteroidetes	Bacteroidia	Bacteroidales	Bacteroidaceae	Bacteroides		0.236138186	0.003557024	**
**98**	5652	MH0003_GL0042613	COG1932	Phosphoserine aminotransferase	Coenzyme transport and metabolism [H]	Glycine, serine and threonine metabolism	Ruminococcus bromii	Species	Firmicutes	Clostridia	Clostridiales	Ruminococcaceae	Ruminococcus	Ruminococcus bromii	0.092831476	0.259873812	
**99**	5672	MH0003_GL0044262	COG0058	Glucan phosphorylase	Carbohydrate transport and metabolism [G]	Starch and sucrose metabolism	Ruminococcus bromii	Species	Firmicutes	Clostridia	Clostridiales	Ruminococcaceae	Ruminococcus	Ruminococcus bromii	0.170144955	0.100660427	
**100**	5730	MH0003_GL0051189	COG0459	Chaperonin GroEL (HSP60 family)	Posttranslational modification, protein turnover, chaperones [O]	RNA degradation	Ruminococcus bromii	Species	Firmicutes	Clostridia	Clostridiales	Ruminococcaceae	Ruminococcus	Ruminococcus bromii	0.000384096	0.997364772	
**101**	5748	MH0003_GL0052996			NAN	NAN	Bacteroides	Genus	Bacteroidetes	Bacteroidia	Bacteroidales	Bacteroidaceae	Bacteroides		0.237334049	0.029677145	*
**102**	5795	MH0003_GL0062474	COG0166	Glucose-6-phosphate isomerase	Carbohydrate transport and metabolism [G]	Glycolysis / Gluconeogenesis	Ruminococcus bromii	Species	Firmicutes	Clostridia	Clostridiales	Ruminococcaceae	Ruminococcus	Ruminococcus bromii	-0.02734205	0.743735393	
**103**	5840	MH0003_GL0068286	COG0094	Ribosomal protein L5	Translation, ribosomal structure and biogenesis [J]	Ribosome	Faecalibacterium prausnitzii	Species	Firmicutes	Clostridia	Clostridiales	Ruminococcaceae	Faecalibacterium	Faecalibacterium prausnitzii	-0.056538868	0.304376503	
**104**	5939	MH0003_GL0080136	COG0059	Ketol-acid reductoisomerase	Coenzyme transport and metabolism [H]	Valine, leucine and isoleucine biosynthesis	Ruminococcus bromii	Species	Firmicutes	Clostridia	Clostridiales	Ruminococcaceae	Ruminococcus	Ruminococcus bromii	0.049424242	0.572425914	
**105**	5952	MH0003_GL0082437	COG4206	Outer membrane cobalamin receptor protein	Coenzyme transport and metabolism [H]	NAN	Bacteroides	Genus	Bacteroidetes	Bacteroidia	Bacteroidales	Bacteroidaceae	Bacteroides		0.291859258	0.032200462	*
**106**	5959	MH0003_GL0083869	COG3842	ABC-type Fe3+/spermidine/putrescine transport systems, ATPase components	Amino acid transport and metabolism [E]	ABC transporters	Ruminococcus bromii	Species	Firmicutes	Clostridia	Clostridiales	Ruminococcaceae	Ruminococcus	Ruminococcus bromii	-0.087156539	0.108532302	
**107**	5986	O2.UC48-0_GL0114931	COG0329	Dihydrodipicolinate synthase/N-acetylneuraminate lyase	Cell wall/membrane/envelope biogenesis [M]	Lysine biosynthesis	Ruminococcus bromii	Species	Firmicutes	Clostridia	Clostridiales	Ruminococcaceae	Ruminococcus	Ruminococcus bromii	0.178737774	0.023747567	*
**108**	6008	MH0003_GL0091064	COG1884	Methylmalonyl-CoA mutase, N-terminal domain/subunit	Lipid transport and metabolism [I]	Valine, leucine and isoleucine degradation	Dialister invisus	Species	Firmicutes	Negativicutes	Veillonellales	Veillonellaceae	Dialister	Dialister invisus	-0.13056969	0.098949262	
**109**	6127	MH0003_GL0109380			NAN	NAN	Bacteroides	Genus	Bacteroidetes	Bacteroidia	Bacteroidales	Bacteroidaceae	Bacteroides		0.187143077	0.062728824	
**110**	6649	MH0006_GL0003713	COG0493	NADPH-dependent glutamate synthase beta chain or related oxidoreductase	General function prediction only [R]	Alanine, aspartate and glutamate metabolism	Ruminococcus bromii	Species	Firmicutes	Clostridia	Clostridiales	Ruminococcaceae	Ruminococcus	Ruminococcus bromii	-0.119033895	0.088664122	
**111**	6834	MH0006_GL0056289	COG0493	NADPH-dependent glutamate synthase beta chain or related oxidoreductase	General function prediction only [R]	Alanine, aspartate and glutamate metabolism	Ruminococcus sp. 5_1_39BFAA	Species	Firmicutes	Clostridia	Clostridiales	Ruminococcaceae	Ruminococcus	Ruminococcus sp. 5_1_39BFAA	-0.02586361	0.598606141	
**112**	6872	V1.UC53-0_GL0115256	COG0205	6-phosphofructokinase	Carbohydrate transport and metabolism [G]	Glycolysis / Gluconeogenesis	Clostridiales	Order	Firmicutes	Clostridia	Clostridiales				0.092257129	0.080502122	
**113**	6967	MH0006_GL0091516	COG0126	3-phosphoglycerate kinase	Carbohydrate transport and metabolism [G]	Glycolysis / Gluconeogenesis	Firmicutes	Phylum	Firmicutes						-0.051546759	0.354694015	
**114**	6984	MH0006_GL0095460	COG0205	6-phosphofructokinase	Carbohydrate transport and metabolism [G]	Glycolysis / Gluconeogenesis	Clostridiales	Order	Firmicutes	Clostridia	Clostridiales				-0.063228845	0.15852851	

**115**	6996	MH0006_GL0098674	COG1129	ABC-type sugar transport system, ATPase component	Carbohydrate transport and metabolism [G]	ABC transporters	Clostridiales	Order	Firmicutes	Clostridia	Clostridiales				0.040807633	0.418001446	
**116**	7025	MH0006_GL0106010	COG1614	CO dehydrogenase/acetyl-CoA synthase beta subunit	Energy production and conversion [C]	Methane metabolism	Clostridiales	Order	Firmicutes	Clostridia	Clostridiales				-0.119028091	0.191891661	
**117**	7087	MH0006_GL0124546	COG0166	Glucose-6-phosphate isomerase	Carbohydrate transport and metabolism [G]	Glycolysis / Gluconeogenesis	Clostridiales	Order	Firmicutes	Clostridia	Clostridiales				-0.013792329	0.755802749	
**118**	7139	MH0006_GL0140949	COG1629	Outer membrane receptor proteins, mostly Fe transport	Inorganic ion transport and metabolism [P]	NAN	Alistipes putredinis	Species	Bacteroidetes	Bacteroidia	Bacteroidales	Rikenellaceae	Alistipes	Alistipes putredinis	0.134873376	0.069584073	
**119**	7197	MH0006_GL0156531	COG0696	Phosphoglycerate mutase (BPG-independent, AlkP superfamily)	Carbohydrate transport and metabolism [G]	Glycolysis / Gluconeogenesis	Ruminococcus bromii	Species	Firmicutes	Clostridia	Clostridiales	Ruminococcaceae	Ruminococcus	Ruminococcus bromii	0.155887472	0.006082017	**
**120**	7272	MH0006_GL0173348	COG0448	ADP-glucose pyrophosphorylase(3043)	Carbohydrate transport and metabolism [G]	Starch and sucrose metabolism	Clostridiales	Order	Firmicutes	Clostridia	Clostridiales				-0.033785781	0.499162706	
**121**	7287	MH0006_GL0174816	COG1712	Predicted dinucleotide-utilizing enzyme	General function prediction only [R]	Lysine biosynthesis	Clostridiales	Order	Firmicutes	Clostridia	Clostridiales				-0.030502634	0.553808106	
**122**	7291	MH0006_GL0176047	COG3063	Tfp pilus assembly protein PilF	Extracellular structures [W]	NAN	Parabacteroides	Genus	Bacteroidetes	Bacteroidia	Bacteroidales	Porphyromonadaceae	Parabacteroides		0.228963455	0.010262924	*
**123**	7429	MH0006_GL0211764	COG0202	DNA-directed RNA polymerase, alpha subunit/40 kD subunit	Transcription [K]	Purine metabolism	Ruminococcus bromii	Species	Firmicutes	Clostridia	Clostridiales	Ruminococcaceae	Ruminococcus	Ruminococcus bromii	-0.080816632	0.272433948	
**124**	7431	MH0006_GL0212607	COG2069	CO dehydrogenase/acetyl-CoA synthase delta subunit (corrinoid Fe-S protein)	Energy production and conversion [C]	Methane metabolism	Clostridiales	Order	Firmicutes	Clostridia	Clostridiales				-0.170945879	0.041248709	*
**125**	7583	MH0008_GL0014349	COG0091	Ribosomal protein L22	Translation, ribosomal structure and biogenesis [J]	Ribosome	Faecalibacterium prausnitzii	Species	Firmicutes	Clostridia	Clostridiales	Ruminococcaceae	Faecalibacterium	Faecalibacterium prausnitzii	-0.122732326	0.031441134	*
**126**	7656	MH0008_GL0044786	COG3181	Tripartite-type tricarboxylate transporter, receptor component TctC(1072)	Energy production and conversion [C]	Two-component system	Clostridiales	Order	Firmicutes	Clostridia	Clostridiales				-0.045746224	0.409283143	
**127**	7789	MH0009_GL0049397	COG0091	Ribosomal protein L22	Translation, ribosomal structure and biogenesis [J]	Ribosome	Clostridiales	Order	Firmicutes	Clostridia	Clostridiales				0.097818815	0.067421998	
**128**	7922	MH0010_GL0016261	COG0539	Ribosomal protein S1	Translation, ribosomal structure and biogenesis [J]	Terpenoid backbone biosynthesis	Faecalibacterium prausnitzii	Species	Firmicutes	Clostridia	Clostridiales	Ruminococcaceae	Faecalibacterium	Faecalibacterium prausnitzii	-0.314208299	0.010734841	*
**129**	7999	MH0010_GL0038237	COG0800	2-keto-3-deoxy-6-phosphogluconate aldolase	Carbohydrate transport and metabolism [G]	Pentose phosphate pathway	Faecalibacterium prausnitzii	Species	Firmicutes	Clostridia	Clostridiales	Ruminococcaceae	Faecalibacterium	Faecalibacterium prausnitzii	0.098594726	0.346658529	
**130**	8219	MH0011_GL0036474	COG1028	NAD(P)-dependent dehydrogenase, short-chain alcohol dehydrogenase family	General function prediction only [R]	NAN	Faecalibacterium prausnitzii	Species	Firmicutes	Clostridia	Clostridiales	Ruminococcaceae	Faecalibacterium	Faecalibacterium prausnitzii	-0.041987673	0.709289433	
**131**	8474	MH0011_GL0094125	COG0203	Ribosomal protein L17	Translation, ribosomal structure and biogenesis [J]	Ribosome	Lachnospiraceae	Family	Firmicutes	Clostridia	Clostridiales	Lachnospiraceae			-0.120235386	0.013237124	*
**132**	8579	MH0012_GL0011046	COG3842	ABC-type Fe3+/spermidine/putrescine transport systems, ATPase components	Amino acid transport and metabolism [E]	ABC transporters	Ruminococcaceae	Family	Firmicutes	Clostridia	Clostridiales	Ruminococcaceae			-0.205258005	0.007732047	**
**133**	8672	MH0012_GL0035930	COG0834	ABC-type amino acid transport/signal transduction system, periplasmic component/domain	Signal transduction mechanisms [T]	NAN	Ruminococcus bromii	Species	Firmicutes	Clostridia	Clostridiales	Ruminococcaceae	Ruminococcus	Ruminococcus bromii	0.088626158	0.135302836	
**134**	8811	MH0012_GL0079061	COG0282	Acetate kinase	Energy production and conversion [C]	Taurine and hypotaurine metabolism	Clostridium pasteurianum	Species	Firmicutes	Clostridia	Clostridiales	Clostridiaceae	Clostridium	Clostridium pasteurianum	-0.345252405	0.000112928	***
**135**	8942	MH0012_GL0119203	COG1362	Aspartyl aminopeptidase	Amino acid transport and metabolism [E]	NAN	Ruminococcus bromii	Species	Firmicutes	Clostridia	Clostridiales	Ruminococcaceae	Ruminococcus	Ruminococcus bromii	0.175416182	0.002796667	**
**136**	8957	MH0012_GL0123603			NAN	NAN	Eubacterium	Genus	Firmicutes	Clostridia	Clostridiales	Eubacteriaceae	Eubacterium		-0.192326808	0.065348315	
**137**	9003	MH0012_GL0138305	COG0443	Molecular chaperone DnaK (HSP70)	Posttranslational modification, protein turnover, chaperones [O]	RNA degradation	Subdoligranulum variabile	Species	Firmicutes	Clostridia	Clostridiales	Ruminococcaceae	Subdoligranulum	Subdoligranulum variabile	-0.152937988	0.049368924	*
**138**	9043	MH0012_GL0150149	COG1653	ABC-type glycerol-3-phosphate transport system, periplasmic component	Carbohydrate transport and metabolism [G]	ABC transporters	[Eubacterium] rectale	Species	Firmicutes	Clostridia	Clostridiales	Lachnospiraceae		[Eubacterium] rectale	0.080317283	0.540269429	
**139**	9087	V1.CD7-0-PN_GL0009048	COG0051	Ribosomal protein S10	Translation, ribosomal structure and biogenesis [J]	Ribosome	Bacteria	Superkingdom							-0.141092293	0.055287166	
**140**	9211	MH0012_GL0193632	COG4799	Acetyl-CoA carboxylase, carboxyltransferase component	Lipid transport and metabolism [I]	Fatty acid biosynthesis	Dialister	Genus	Firmicutes	Negativicutes	Veillonellales	Veillonellaceae	Dialister		-0.115752537	0.234714393	
**141**	9225	MH0012_GL0196811	COG0480	Translation elongation factor EF-G, a GTPase	Translation, ribosomal structure and biogenesis [J]	NAN	Subdoligranulum variabile	Species	Firmicutes	Clostridia	Clostridiales	Ruminococcaceae	Subdoligranulum	Subdoligranulum variabile	-0.104515289	0.103491252	
**142**	9235	MH0012_GL0199057	COG2885	Outer membrane protein OmpA and related peptidoglycan-associated (lipo)proteins	Cell wall/membrane/envelope biogenesis [M]	NAN	Bacteroidales	Order	Bacteroidetes	Bacteroidia	Bacteroidales				0.215057741	0.003539069	**
**143**	9331	MH0012_GL0231760	COG1592	Rubrerythrin	Energy production and conversion [C]	NAN	Faecalibacterium prausnitzii	Species	Firmicutes	Clostridia	Clostridiales	Ruminococcaceae	Faecalibacterium	Faecalibacterium prausnitzii	-0.056723244	0.624399731	
**144**	9336	MH0012_GL0232564	COG4771	Outer membrane receptor for ferrienterochelin and colicins(	Inorganic ion transport and metabolism [P]	NAN	Bacteroides	Genus	Bacteroidetes	Bacteroidia	Bacteroidales	Bacteroidaceae	Bacteroides		0.165942936	0.098664736	
**145**	9554	MH0014_GL0025905	COG4238	Outer membrane murein-binding lipoprotein Lpp	Cell wall/membrane/envelope biogenesis [M]	NAN	Proteobacteria	Phylum	Proteobacteria						0.116925869	0.369924153	
**146**	9600	O2.UC49-0_GL0079827	COG1883	Na+-transporting methylmalonyl-CoA/oxaloacetate decarboxylase, beta subunit	Energy production and conversion [C]	Arginine and proline metabolism	Bacteria	Superkingdom							-0.049808117	0.385500703	
**147**	9717	MH0014_GL0093892	COG0057	Glyceraldehyde-3-phosphate dehydrogenase/erythrose-4-phosphate dehydrogenase	Carbohydrate transport and metabolism [G]	Glycolysis / Gluconeogenesis	Clostridiales	Order	Firmicutes	Clostridia	Clostridiales				0.141866657	0.017159853	*
**148**	9728	MH0014_GL0097483	COG1592	Rubrerythrin	Energy production and conversion [C]	NAN	Clostridiales	Order	Firmicutes	Clostridia	Clostridiales				0.281328749	0.001500976	**
**149**	9731	MH0014_GL0098098			NAN	NAN	Ruminococcus bromii	Species	Firmicutes	Clostridia	Clostridiales	Ruminococcaceae	Ruminococcus	Ruminococcus bromii	0.068939238	0.428294518	
**150**	10572	MH0025_GL0075388	COG0510	Thiamine kinase and related kinases	Coenzyme transport and metabolism [H]	NAN	Ruminococcus bromii	Species	Firmicutes	Clostridia	Clostridiales	Ruminococcaceae	Ruminococcus	Ruminococcus bromii	-0.049024811	0.582370678	
**151**	10738	MH0026_GL0035301	COG0057	Glyceraldehyde-3-phosphate dehydrogenase/erythrose-4-phosphate dehydrogenase(2333)	Carbohydrate transport and metabolism [G]	Glycolysis / Gluconeogenesis	Eubacterium	Genus	Firmicutes	Clostridia	Clostridiales	Eubacteriaceae	Eubacterium		-0.033910331	0.676023451	
**152**	10969	MH0028_GL0071718			NAN	NAN	Faecalibacterium prausnitzii	Species	Firmicutes	Clostridia	Clostridiales	Ruminococcaceae	Faecalibacterium	Faecalibacterium prausnitzii	0.034454697	0.667992384	
**153**	11241	MH0032_GL0024527	COG0090	Ribosomal protein L2(1921)	Translation, ribosomal structure and biogenesis [J]	Ribosome	Dialister succinatiphilus	Species	Firmicutes	Negativicutes	Veillonellales	Veillonellaceae	Dialister	Dialister succinatiphilus	-0.252657558	0.000649382	***

154	11414	MH0037_GL0008359	COG0685	5,10-methylenetetrahydrofolate reductase(1603)	Amino acid transport and metabolism [E]	Cysteine and methionine metabolism	Lachnospiraceae	Family	Firmicutes	Clostridia	Clostridiales	Lachnospiraceae			-0.102089223	0.130838791	
155	11463	MH0037_GL0027576			NAN	NAN	Bacteria	Superkingdom							0.277908954	0.001122001	**
156	11465	MH0037_GL0028219	COG0166	Glucose-6-phosphate isomerase	Carbohydrate transport and metabolism [G]	Glycolysis / Gluconeogenesis	Lachnospiraceae	Family	Firmicutes	Clostridia	Clostridiales	Lachnospiraceae			-0.0231611	0.70269101	
157	11699	MH0041_GL0000003	COG0091	Ribosomal protein L22	Translation, ribosomal structure and biogenesis [J]	Ribosome	Bacteria	Superkingdom							-0.067146344	0.278882799	
158	11766	MH0041_GL0040403	COG1866	Phosphoenolpyruvate carboxykinase, ATP-dependent	Energy production and conversion [C]	Glycolysis / Gluconeogenesis	Roseburia faecis	Species	Firmicutes	Clostridia	Clostridiales	Lachnospiraceae	Roseburia	Roseburia faecis	-0.325471428	7.34119E-05	***
159	11804	MH0041_GL0063223	COG0255	Ribosomal protein L29	Translation, ribosomal structure and biogenesis [J]	Ribosome	Bifidobacterium adolescentis	Species	Actinobacteria	Actinobacteria	Bifidobacteriales	Bifidobacteriaceae	Bifidobacterium	Bifidobacterium adolescentis	-0.091532406	0.168101323	
160	11861	MH0043_GL0046307	COG0334	Glutamate dehydrogenase/leucine dehydrogenase	Amino acid transport and metabolism [E]	Alanine, aspartate and glutamate metabolism	Arthrobacter sp. YC-RL1	Species	Actinobacteria	Actinobacteria	Micrococcales	Micrococcaceae	Arthrobacter	Arthrobacter sp. YC-RL1	0.076138236	0.424887185	
161	11983	MH0045_GL0023728	COG4206	Outer membrane cobalamin receptor protein	Coenzyme transport and metabolism [H]	NAN	Bacteroides	Genus	Bacteroidetes	Bacteroidia	Bacteroidales	Bacteroidaceae	Bacteroides		0.221148778	0.065543295	
162	12181	MH0048_GL0045161	COG0334	Glutamate dehydrogenase/leucine dehydrogenase	Amino acid transport and metabolism [E]	Alanine, aspartate and glutamate metabolism	Eubacterium	Genus	Firmicutes	Clostridia	Clostridiales	Eubacteriaceae	Eubacterium		0.23736267	0.004523097	**
163	12475	MH0053_GL0032330	COG1080	Phosphoenolpyruvate-protein kinase (PTS system EI component in bacteria)	Carbohydrate transport and metabolism [G]	Pyruvate metabolism	Clostridium	Genus	Firmicutes	Clostridia	Clostridiales	Clostridiaceae	Clostridium		-0.080784914	0.141529338	
164	12661	MH0055_GL0011037	COG1960	Acyl-CoA dehydrogenase related to the alkylation response protein AidB	Lipid transport and metabolism [I]	Fatty acid degradation	Coprococcus eutactus	Species	Firmicutes	Clostridia	Clostridiales	Lachnospiraceae	Coprococcus	Coprococcus eutactus	-0.175290944	0.0248999	*
165	12697	MH0055_GL0031824	COG0050	Translation elongation factor EF-Tu, a GTPase	Translation, ribosomal structure and biogenesis [J]	Plant-pathogen interaction	Faecalibacterium prausnitzii	Species	Firmicutes	Clostridia	Clostridiales	Ruminococcaceae	Faecalibacterium	Faecalibacterium prausnitzii	-0.29571511	0.025487098	*
166	12719	MH0055_GL0039831	COG1882	Pyruvate-formate lyase	Energy production and conversion [C]	Pyruvate metabolism	Coprococcus eutactus	Species	Firmicutes	Clostridia	Clostridiales	Lachnospiraceae	Coprococcus	Coprococcus eutactus	-0.17520582	0.033230221	*
167	13102	MH0060_GL0028240	COG0524	Sugar or nucleoside kinase, ribokinase family	Carbohydrate transport and metabolism [G]	Pentose phosphate pathway	Ruminococcaceae	Family	Firmicutes	Clostridia	Clostridiales	Ruminococcaceae			0.053008901	0.462667085	
168	13344	MH0062_GL0037172	COG1024	Enoyl-CoA hydratase/carnithine racemase	Lipid transport and metabolism [I]	NAN	Clostridiales	Order	Firmicutes	Clostridia	Clostridiales				-0.009459934	0.883239159	
169	13357	MH0062_GL0041928	COG0206	Cell division GTPase FtsZ	Cell cycle control, cell division, chromosome partitioning [D]	Cell cycle - Caulobacter	Faecalibacterium prausnitzii	Species	Firmicutes	Clostridia	Clostridiales	Ruminococcaceae	Faecalibacterium	Faecalibacterium prausnitzii	-0.126091903	0.009542161	**
170	13391	MH0062_GL0058668	COG4166	ABC-type oligopeptide transport system, periplasmic component	Amino acid transport and metabolism [E]	ABC transporters	Faecalibacterium prausnitzii	Species	Firmicutes	Clostridia	Clostridiales	Ruminococcaceae	Faecalibacterium	Faecalibacterium prausnitzii	0.244931815	0.005186699	**
171	13399	MH0062_GL0061760	COG0052	Ribosomal protein S2	Translation, ribosomal structure and biogenesis [J]	Ribosome	Lachnospiraceae	Family	Firmicutes	Clostridia	Clostridiales	Lachnospiraceae			-0.088619225	0.093696059	
172	14026	MH0073_GL0056444			NAN	Galactose metabolism	Ruminococcus bromii	Species	Firmicutes	Clostridia	Clostridiales	Ruminococcaceae	Ruminococcus	Ruminococcus bromii	0.181533883	0.013897574	*
173	14108	MH0076_GL0047189			NAN	NAN	Clostridiales	Order	Firmicutes	Clostridia	Clostridiales				0.056529836	0.555335022	
174	14551	MH0086_GL0023405	COG1744	Basic membrane lipoprotein Med, periplasmic binding protein (PBP1-ABC) superfamily	Cell wall/membrane/envelope biogenesis [M]	NAN	Clostridiales	Order	Firmicutes	Clostridia	Clostridiales				0.084886991	0.162990777	
175	14579	MH0086_GL0029553	COG0517	CBS domain	Signal transduction mechanisms [T]	Purine metabolism	Faecalibacterium prausnitzii	Species	Firmicutes	Clostridia	Clostridiales	Ruminococcaceae	Faecalibacterium	Faecalibacterium prausnitzii	0.05213495	0.247628185	
176	14619	MH0086_GL0046721	COG1143	Formate hydrogenlyase subunit 6/NADH:ubiquinone oxidoreductase 23 kD subunit (chain I)	Energy production and conversion [C]	Glycolysis / Gluconeogenesis	Ruminococcus	Genus	Firmicutes	Clostridia	Clostridiales	Ruminococcaceae	Ruminococcus		-0.190376825	0.051902184	
177	14632	MH0086_GL0050799	COG1456	CO dehydrogenase/acetyl-CoA synthase gamma subunit (corrinoid Fe-S protein)	Energy production and conversion [C]	Methane metabolism	Clostridiales	Order	Firmicutes	Clostridia	Clostridiales				-0.189918364	0.006557014	**
178	14652	MH0086_GL0058027	COG4624	Iron only hydrogenase large subunit, C-terminal domain	Energy production and conversion [C]	Oxidative phosphorylation	Clostridiales	Order	Firmicutes	Clostridia	Clostridiales				-0.053189437	0.31950652	
179	14676	MH0086_GL0071322	COG1882	Pyruvate-formate lyase	Energy production and conversion [C]	Pyruvate metabolism	Clostridiales	Order	Firmicutes	Clostridia	Clostridiales				-0.086347482	0.205663134	
180	14692	MH0086_GL0077208	COG1960	Acyl-CoA dehydrogenase related to the alkylation response protein AidB	Lipid transport and metabolism [I]	Fatty acid degradation	Clostridiales	Order	Firmicutes	Clostridia	Clostridiales				0.004467646	0.965455592	
181	14751	MH0086_GL0098687	COG4166	ABC-type oligopeptide transport system, periplasmic component	Amino acid transport and metabolism [E]	ABC transporters	Clostridiales	Order	Firmicutes	Clostridia	Clostridiales				0.04187522	0.546692084	
182	14763	MH0086_GL0104589	COG1129	ABC-type sugar transport system, ATPase component	Carbohydrate transport and metabolism [G]	NAN	Subdoligranulum variabile	Species	Firmicutes	Clostridia	Clostridiales	Ruminococcaceae	Subdoligranulum	Subdoligranulum variabile	-0.053209767	0.39406396	
183	14788	MH0086_GL0116500	COG0138	AICAR transformylase/IMP cyclohydrolase PurH	Nucleotide transport and metabolism [F]	Purine metabolism	Clostridiales	Order	Firmicutes	Clostridia	Clostridiales				0.033498121	0.500049926	
184	14811	MH0087_GL0004799	COG0522	Ribosomal protein S4 or related protein	Translation, ribosomal structure and biogenesis [J]	Ribosome biogenesis in eukaryotes	Clostridiales	Order	Firmicutes	Clostridia	Clostridiales				-0.150701985	0.013426499	*
185	14833	MH0087_GL0012728	COG4656	Na+-translocating ferredoxin:NAD+ oxidoreductase RNF, RnfC subunit	Energy production and conversion [C]	NAN	Clostridiales	Order	Firmicutes	Clostridia	Clostridiales				-0.144686734	0.08407027	
186	14837	MH0087_GL0014198	COG1866	Phosphoenolpyruvate carboxykinase, ATP-dependent	Energy production and conversion [C]	Glycolysis / Gluconeogenesis	Clostridiales	Order	Firmicutes	Clostridia	Clostridiales				0.035028722	0.458436555	
187	14871	MH0087_GL0031294	COG5263	Glucan-binding domain (YG repeat)	Carbohydrate transport and metabolism [G]	NAN	Clostridium	Genus	Firmicutes	Clostridia	Clostridiales	Clostridiaceae	Clostridium		0.208762471	0.068063694	
188	14892	MH0087_GL0040866	COG0443	Molecular chaperone DnaK (HSP70)	Posttranslational modification, protein turnover, chaperones [O]	RNA degradation	Clostridiales	Order	Firmicutes	Clostridia	Clostridiales				-0.055483468	0.403216894	
189	14910	MH0087_GL0049381	COG1454	Alcohol dehydrogenase, class IV	Energy production and conversion [C]	Glycolysis / Gluconeogenesis	Blautia obeum	Species	Firmicutes	Clostridia	Clostridiales	Lachnospiraceae	Blautia	Blautia obeum	-0.040616814	0.592583591	
190	14942	MH0088_GL0009851	COG0280	Phosphotransacetylase	Energy production and conversion [C]	Taurine and hypotaurine metabolism	Lachnospiraceae	Family	Firmicutes	Clostridia	Clostridiales	Lachnospiraceae			-0.037836528	0.44593107	
191	14967	MH0088_GL0016997	COG0696	Phosphoglycerate mutase (BPG-independent, AlkP superfamily)	Carbohydrate transport and metabolism [G]	Glycolysis / Gluconeogenesis	Clostridiales	Order	Firmicutes	Clostridia	Clostridiales				-0.027495769	0.624592294	
192	15013	MH0088_GL0029088	COG0137	Argininosuccinate synthase	Amino acid transport and metabolism [E]	Alanine, aspartate and glutamate metabolism	Clostridiales	Order	Firmicutes	Clostridia	Clostridiales				-0.064749445	0.237150028	

**193**	15022	MH0088_GL0033424	COG0094	Ribosomal protein L5	Translation, ribosomal structure and biogenesis [J]	Ribosome	Blautia sp. KLE 1732	Species	Firmicutes	Clostridia	Clostridiales	Lachnospiraceae	Blautia	Blautia sp. KLE 1732	-0.113442487	0.037305463	*
**194**	15026	MH0088_GL0034073	COG0480	Translation elongation factor EF-G, a GTPase	Translation, ribosomal structure and biogenesis [J]	NAN	Clostridiales	Order	Firmicutes	Clostridia	Clostridiales				-0.196489027	0.007825366	**
**195**	15079	MH0088_GL0054024	COG0136	Aspartate-semialdehyde dehydrogenase	Amino acid transport and metabolism [E]	Glycine, serine and threonine metabolism	Clostridiales	Order	Firmicutes	Clostridia	Clostridiales				-0.013734195	0.814720849	
**196**	15122	MH0088_GL0066516	COG1454	Alcohol dehydrogenase, class IV	Energy production and conversion [C]	Glycolysis / Gluconeogenesis	Clostridiales	Order	Firmicutes	Clostridia	Clostridiales				0.148172465	0.022161215	*
**197**	15182	MH0088_GL0086499	COG0031	Cysteine synthase	Amino acid transport and metabolism [E]	Glycine, serine and threonine metabolism	Clostridiales	Order	Firmicutes	Clostridia	Clostridiales				0.131586438	0.002802731	**
**198**	15257	MH0088_GL0109165	COG0624	Acetylornithine deacetylase/Succinyl-diaminopimelate desuccinylase or related deacylase	Amino acid transport and metabolism [E]	Lysine biosynthesis	Clostridiales	Order	Firmicutes	Clostridia	Clostridiales				0.098175478	0.23403604	
**199**	15393	MH0089_GL0026063	COG0050	Translation elongation factor EF-Tu, a GTPase	Translation, ribosomal structure and biogenesis [J]	Plant-pathogen interaction	Clostridiales	Order	Firmicutes	Clostridia	Clostridiales				-0.138363219	0.0506875	
**200**	15465	MH0089_GL0075669	COG0057	Glyceraldehyde-3-phosphate dehydrogenase/erythrose-4-phosphate dehydrogenase	Carbohydrate transport and metabolism [G]	Glycolysis / Gluconeogenesis	Clostridiales	Order	Firmicutes	Clostridia	Clostridiales				-0.136440202	0.070224547	
**201**	15627	MH0092_GL0003446	COG0329	Dihydrodipicolinate synthase/N-acetylneuraminate lyase	Cell wall/membrane/envelope biogenesis [M]	Lysine biosynthesis	Faecalibacterium prausnitzii	Species	Firmicutes	Clostridia	Clostridiales	Ruminococcaceae	Faecalibacterium	Faecalibacterium prausnitzii	0.00978999	0.850211054	
**202**	15670	MH0092_GL0036208	COG1653	ABC-type glycerol-3-phosphate transport system, periplasmic component	Carbohydrate transport and metabolism [G]	NAN	Faecalibacterium prausnitzii	Species	Firmicutes	Clostridia	Clostridiales	Ruminococcaceae	Faecalibacterium	Faecalibacterium prausnitzii	-0.001930093	0.989408498	
**203**	15697	MH0092_GL0074084	COG3853	Uncharacterized conserved protein YaaN involved in tellurite resistance	Defense mechanisms [V]	NAN	Bacteria	Species	Firmicutes	Clostridia	Clostridiales	Ruminococcaceae	Faecalibacterium	Faecalibacterium prausnitzii	-0.143487964	0.047849412	*
**204**	15723	MH0092_GL0097242	COG0166	Glucose-6-phosphate isomerase	Carbohydrate transport and metabolism [G]	Glycolysis / Gluconeogenesis	Faecalibacterium prausnitzii	Species	Firmicutes	Clostridia	Clostridiales	Ruminococcaceae	Faecalibacterium	Faecalibacterium prausnitzii	-0.000728607	0.990228749	
**205**	16373	MH0101_GL0050789	COG4465	GTP-sensing pleiotropic transcriptional regulator CodY	Transcription [K]	NAN	Clostridiales	Order	Firmicutes	Clostridia	Clostridiales				-0.038360813	0.347204409	
**206**	16397	MH0101_GL0060753	COG0148	Enolase	Carbohydrate transport and metabolism [G]	Glycolysis / Gluconeogenesis	Clostridiales	Order	Firmicutes	Clostridia	Clostridiales				0.006129097	0.947096578	
**207**	16733	MH0106_GL0002286	COG1053	Succinate dehydrogenase/fumarate reductase, flavoprotein subunit	Energy production and conversion [C]	Citrate cycle (TCA cycle)	Bacteroides coprocola	Species	Bacteroidetes	Bacteroidia	Bacteroidales	Bacteroidaceae	Bacteroides	Bacteroides coprocola	0.277775491	0.001603154	**
**208**	16873	MH0108_GL0010146	COG0050	Translation elongation factor EF-Tu, a GTPase	Translation, ribosomal structure and biogenesis [J]	Plant-pathogen interaction	Clostridiales	Order	Firmicutes	Clostridia	Clostridiales				0.112516923	0.186794136	
**209**	16887	MH0108_GL0025722	COG0103	Ribosomal protein S9	Translation, ribosomal structure and biogenesis [J]	Ribosome	Faecalibacterium prausnitzii	Species	Firmicutes	Clostridia	Clostridiales	Ruminococcaceae	Faecalibacterium	Faecalibacterium prausnitzii	-0.175397424	0.020708532	*
**210**	16893	T2D-105A_GL0119985	COG0822	NifU homolog involved in Fe-S cluster formation	Posttranslational modification, protein turnover, chaperones [O]	NAN	Bacteroides	Genus	Bacteroidetes	Bacteroidia	Bacteroidales	Bacteroidaceae	Bacteroides		-0.044224363	0.458669657	
**211**	16919	MH0108_GL0057076	COG1250	3-hydroxyacyl-CoA dehydrogenase	Lipid transport and metabolism [I]	Fatty acid degradation	Faecalibacterium prausnitzii	Species	Firmicutes	Clostridia	Clostridiales	Ruminococcaceae	Faecalibacterium	Faecalibacterium prausnitzii	0.002636064	0.977468505	
**212**	16923	MH0108_GL0062043	COG0092	Ribosomal protein S3	Translation, ribosomal structure and biogenesis [J]	Ribosome	Faecalibacterium prausnitzii	Species	Firmicutes	Clostridia	Clostridiales	Ruminococcaceae	Faecalibacterium	Faecalibacterium prausnitzii	-0.187086258	0.016436142	*
**213**	16948	MH0108_GL0083509	COG0104	Adenylosuccinate synthase	Nucleotide transport and metabolism [F]	Purine metabolism	Faecalibacterium prausnitzii	Species	Firmicutes	Clostridia	Clostridiales	Ruminococcaceae	Faecalibacterium	Faecalibacterium prausnitzii	-0.097524343	0.13606868	
**214**	16977	MH0109_GL0036381	COG0149	Triosephosphate isomerase	Carbohydrate transport and metabolism [G]	Glycolysis / Gluconeogenesis	Firmicutes	Phylum	Firmicutes						-0.00561615	0.922312266	
**215**	17010	MH0110_GL0025297	COG1080	Phosphoenolpyruvate-protein kinase (PTS system EI component in bacteria)	Carbohydrate transport and metabolism [G]	Pyruvate metabolism	Clostridiales	Order	Firmicutes	Clostridia	Clostridiales				-0.223072339	0.011550857	*
**216**	17022	MH0110_GL0036341	COG1866	Phosphoenolpyruvate carboxykinase, ATP-dependent	Energy production and conversion [C]	Glycolysis / Gluconeogenesis	Faecalibacterium prausnitzii	Species	Firmicutes	Clostridia	Clostridiales	Ruminococcaceae	Faecalibacterium	Faecalibacterium prausnitzii	-0.177326322	0.057717849	
**217**	17036	MH0110_GL0060617	COG3842	ABC-type Fe3+/spermidine/putrescine transport systems, ATPase components	Amino acid transport and metabolism [E]	ABC transporters	Clostridiales	Order	Firmicutes	Clostridia	Clostridiales				-0.064125648	0.332663356	
**218**	17080	MH0111_GL0044849	COG1653	ABC-type glycerol-3-phosphate transport system, periplasmic component	Carbohydrate transport and metabolism [G]	NAN	[Eubacterium] rectale	Species	Firmicutes	Clostridia	Clostridiales	Lachnospiraceae		[Eubacterium] rectale	0.066233176	0.560754535	
**219**	17092	MH0111_GL0088449			NAN	NAN	Faecalibacterium prausnitzii	Species	Firmicutes	Clostridia	Clostridiales	Ruminococcaceae	Faecalibacterium	Faecalibacterium prausnitzii	0.073335991	0.626483243	
**220**	17212	MH0114_GL0000378	COG0255	Ribosomal protein L29	Translation, ribosomal structure and biogenesis [J]	Ribosome	Clostridiales	Order	Firmicutes	Clostridia	Clostridiales				-0.107943998	0.028291487	*
**221**	17248	MH0114_GL0065382	COG2848	Uncharacterized conserved protein, UPF0210 family	Cell cycle control, cell division, chromosome partitioning [D]	NAN	Clostridiales	Order	Firmicutes	Clostridia	Clostridiales				0.005695269	0.922669655	
**222**	17503	MH0119_GL0033639	COG1904	Glucuronate isomerase	Carbohydrate transport and metabolism [G]	Pentose and glucuronate interconversions	Faecalibacterium prausnitzii	Species	Firmicutes	Clostridia	Clostridiales	Ruminococcaceae	Faecalibacterium	Faecalibacterium prausnitzii	0.089424875	0.326023584	
**223**	17672	MH0131_GL0013416	COG1592	Rubrerythrin	Energy production and conversion [C]	NAN	Subdoligranulum variabile	Species	Firmicutes	Clostridia	Clostridiales	Ruminococcaceae	Subdoligranulum	Subdoligranulum variabile	-0.266760182	0.021855477	*
**224**	17690	MH0122_GL0011708	COG0050	Translation elongation factor EF-Tu, a GTPase	Translation, ribosomal structure and biogenesis [J]	Plant-pathogen interaction	Subdoligranulum variabile	Species	Firmicutes	Clostridia	Clostridiales	Ruminococcaceae	Subdoligranulum	Subdoligranulum variabile	-0.434455982	0.000173529	***
**225**	17710	MH0122_GL0027891	COG0098	Ribosomal protein S5	Translation, ribosomal structure and biogenesis [J]	Ribosome	Subdoligranulum variabile	Species	Firmicutes	Clostridia	Clostridiales	Ruminococcaceae	Subdoligranulum	Subdoligranulum variabile	-0.110299692	0.027891927	*
**226**	17719	MH0122_GL0036643	COG1024	Enoyl-CoA hydratase/carnithine racemase	Lipid transport and metabolism [I]	NAN	Ruminococcaceae	Species	Firmicutes	Clostridia	Clostridiales	Ruminococcaceae	Faecalibacterium	Faecalibacterium prausnitzii	-0.078774297	0.139636395	
**227**	17729	MH0122_GL0040083	COG0081	Ribosomal protein L1	Translation, ribosomal structure and biogenesis [J]	Ribosome	Bifidobacterium	Genus	Actinobacteria	Actinobacteria	Bifidobacteriales	Bifidobacteriaceae	Bifidobacterium		0.122227175	0.102323517	
**228**	17746	MH0122_GL0058467	COG1960	Acyl-CoA dehydrogenase related to the alkylation response protein AidB	Lipid transport and metabolism [I]	Fatty acid degradation	Ruminococcaceae	Species	Firmicutes	Clostridia	Clostridiales	Ruminococcaceae	Subdoligranulum	Subdoligranulum variabile	-0.150183725	0.032610294	*
**229**	17749	MH0122_GL0061910	COG0255	Ribosomal protein L29	Translation, ribosomal structure and biogenesis [J]	Ribosome	Bifidobacterium	Genus	Actinobacteria	Actinobacteria	Bifidobacteriales	Bifidobacteriaceae	Bifidobacterium		0.044949064	0.587870103	
**230**	17817	MH0122_GL0107606	COG1653	ABC-type glycerol-3-phosphate transport system, periplasmic component	Carbohydrate transport and metabolism [G]	ABC transporters	Bifidobacterium	Genus	Actinobacteria	Actinobacteria	Bifidobacteriales	Bifidobacteriaceae	Bifidobacterium		0.190126122	0.133164314	

**231**	18139	MH0127_GL0025496	COG0126	3-phosphoglycerate kinase	Carbohydrate transport and metabolism [G]	Glycolysis / Gluconeogenesis	Faecalibacterium prausnitzii	Species	Firmicutes	Clostridia	Clostridiales	Ruminococcaceae	Faecalibacterium	Faecalibacterium prausnitzii	-0.142841379	0.167519299	
**232**	18161	MH0127_GL0067595	COG3209	Uncharacterized conserved protein RhaS, contains 28 RHS repeats	General function prediction only [R]	NAN	Blautia obeum	Species	Firmicutes	Clostridia	Clostridiales	Lachnospiraceae	Blautia	Blautia obeum	0.040113806	0.824583424	
**233**	18350	MH0131_GL0003010	COG1070	Sugar (pentulose or hexulose) kinase	Carbohydrate transport and metabolism [G]	Pentose phosphate pathway	Clostridiales	Order	Firmicutes	Clostridia	Clostridiales				-0.03142067	0.540789176	
**234**	18375	MH0131_GL0009720	COG0166	Glucose-6-phosphate isomerase	Carbohydrate transport and metabolism [G]	Glycolysis / Gluconeogenesis	Bifidobacterium	Genus	Actinobacteria	Actinobacteria	Bifidobacteriales	Bifidobacteriaceae	Bifidobacterium		-0.028833491	0.679284301	
**235**	18470	MH0131_GL0036835	COG0624	Acetylornithine deacetylase/Succinyl-diaminopimelate desuccinylase or related deacylase	Amino acid transport and metabolism [E]	Lysine biosynthesis	Collinsella	Genus	Actinobacteria	Coriobacteriia	Coriobacteriales	Coriobacteriaceae	Collinsella		0.144204815	0.031923775	*
**236**	18487	MH0131_GL0040021	COG0166	Glucose-6-phosphate isomerase	Carbohydrate transport and metabolism [G]	Glycolysis / Gluconeogenesis	Clostridiales	Order	Firmicutes	Clostridia	Clostridiales				-0.12383665	0.082478019	
**237**	18490	MH0131_GL0040069	COG0544	FKBP-type peptidyl-prolyl cis-trans isomerase (trigger factor)	Posttranslational modification, protein turnover, chaperones [O]	NAN	Collinsella aerofaciens	Species	Actinobacteria	Coriobacteriia	Coriobacteriales	Coriobacteriaceae	Collinsella	Collinsella aerofaciens	0.125827538	0.020356462	*
**238**	18517	MH0131_GL0045133	COG2352	Phosphoenolpyruvate carboxylase	Energy production and conversion [C]	Pyruvate metabolism	Bifidobacterium	Genus	Actinobacteria	Actinobacteria	Bifidobacteriales	Bifidobacteriaceae	Bifidobacterium		-0.030309005	0.730301693	
**239**	18527	MH0131_GL0047971	COG0469	Pyruvate kinase	Carbohydrate transport and metabolism [G]	Glycolysis / Gluconeogenesis	Collinsella aerofaciens	Species	Actinobacteria	Coriobacteriia	Coriobacteriales	Coriobacteriaceae	Collinsella	Collinsella aerofaciens	0.240963827	0.005638074	**
**240**	18545	MH0131_GL0053906	COG0081	Ribosomal protein L1	Translation, ribosomal structure and biogenesis [J]	Ribosome	Bifidobacterium adolescentis	Species	Actinobacteria	Actinobacteria	Bifidobacteriales	Bifidobacteriaceae	Bifidobacterium	Bifidobacterium adolescentis	-0.126888935	0.113435175	
**241**	18548	MH0131_GL0055410	COG0747	ABC-type transport system, periplasmic component	Amino acid transport and metabolism [E]	ABC transporters	Collinsella aerofaciens	Species	Actinobacteria	Coriobacteriia	Coriobacteriales	Coriobacteriaceae	Collinsella	Collinsella aerofaciens	0.185723822	0.051506423	
**242**	18549	MH0131_GL0055413	COG4608	ABC-type oligopeptide transport system, ATPase component	Amino acid transport and metabolism [E]	ABC transporters		Genus	Actinobacteria	Coriobacteriia	Coriobacteriales	Coriobacteriaceae	Collinsella		0.127462181	0.09124509	
**243**	18559	MH0131_GL0059552	COG0057	Glyceraldehyde-3-phosphate dehydrogenase/erythrose-4-phosphate dehydrogenase	Carbohydrate transport and metabolism [G]	Glycolysis / Gluconeogenesis	Clostridiales	Order	Firmicutes	Clostridia	Clostridiales				-0.134382497	0.159965547	
**244**	18560	NLF015_GL0061486	COG0126	3-phosphoglycerate kinase	Carbohydrate transport and metabolism [G]	NAN	Clostridiales	Order	Firmicutes	Clostridia	Clostridiales				-0.057160551	0.321179142	
**245**	18561	MH0131_GL0059554	COG0149	Triosephosphate isomerase	Carbohydrate transport and metabolism [G]	Glycolysis / Gluconeogenesis	Clostridiales	Order	Firmicutes	Clostridia	Clostridiales				-0.184183161	0.006174535	**
**246**	18580	MH0131_GL0070829	COG1185	Polyribonucleotide nucleotidyltransferase (polynucleotide phosphorylase)	Translation, ribosomal structure and biogenesis [J]	Purine metabolism	Bifidobacterium	Genus	Actinobacteria	Actinobacteria	Bifidobacteriales	Bifidobacteriaceae	Bifidobacterium		-0.009558951	0.900896535	
**247**	18636	MH0131_GL0088691	COG1080	Phosphoenolpyruvate-protein kinase (PTS system EI component in bacteria)	Carbohydrate transport and metabolism [G]	Pyruvate metabolism	Coprococcus	Genus	Firmicutes	Clostridia	Clostridiales	Lachnospiraceae	Coprococcus		-0.209374644	0.01817082	*
**248**	18645	MH0131_GL0090479	COG1454	Alcohol dehydrogenase, class IV	Energy production and conversion [C]	Glycolysis / Gluconeogenesis	Collinsella aerofaciens	Species	Actinobacteria	Coriobacteriia	Coriobacteriales	Coriobacteriaceae	Collinsella	Collinsella aerofaciens	0.201870629	0.025311781	*
**249**	18682	MH0131_GL0099453	COG1454	Alcohol dehydrogenase, class IV	Energy production and conversion [C]	Glycolysis / Gluconeogenesis	Collinsella aerofaciens	Species	Actinobacteria	Coriobacteriia	Coriobacteriales	Coriobacteriaceae	Collinsella	Collinsella aerofaciens	0.120459183	0.174113551	
**250**	18705	MH0131_GL0105124	COG2235	Arginine deiminase	Amino acid transport and metabolism [E]	Arginine and proline metabolism	Collinsella	Genus	Actinobacteria	Coriobacteriia	Coriobacteriales	Coriobacteriaceae	Collinsella		0.403511118	6.4822E-05	***
**251**	18774	MH0307_GL0076667	COG1544	Ribosome-associated translation inhibitor RaiA	Translation, ribosomal structure and biogenesis [J]	NAN	Collinsella	Genus	Actinobacteria	Coriobacteriia	Coriobacteriales	Coriobacteriaceae	Collinsella		-0.011413885	0.835608687	
**252**	18781	MH0131_GL0123487	COG0091	Ribosomal protein L22	Translation, ribosomal structure and biogenesis [J]	Ribosome	Collinsella	Genus	Actinobacteria	Coriobacteriia	Coriobacteriales	Coriobacteriaceae	Collinsella		0.063165766	0.202935318	
**253**	18818	MH0131_GL0136999	COG4822	Cobalamin biosynthesis protein CbiK, Co2+ chelatase	Coenzyme transport and metabolism [H]	Porphyrin and chlorophyll metabolism	Clostridiales	Order	Firmicutes	Clostridia	Clostridiales				-0.066604004	0.397735711	
**254**	18821	MH0131_GL0138470	COG1882	Pyruvate-formate lyase	Energy production and conversion [C]	Pyruvate metabolism	Fusicatenibacter saccharivorans	Species	Firmicutes	Clostridia	Clostridiales	Lachnospiraceae	Fusicatenibacter	Fusicatenibacter saccharivorans	-0.121031581	0.115457666	
**255**	18826	MH0131_GL0139126	COG0264	Translation elongation factor EF-Ts	Translation, ribosomal structure and biogenesis [J]	NAN	Collinsella	Genus	Actinobacteria	Coriobacteriia	Coriobacteriales	Coriobacteriaceae	Collinsella		0.100155163	0.15126239	
**256**	18868	MH0131_GL0154213	COG0544	FKBP-type peptidyl-prolyl cis-trans isomerase (trigger factor)	Posttranslational modification, protein turnover, chaperones [O]	NAN	Bifidobacterium	Genus	Actinobacteria	Actinobacteria	Bifidobacteriales	Bifidobacteriaceae	Bifidobacterium		0.145231598	0.138014801	
**257**	18927	MH0131_GL0169225	COG0094	Ribosomal protein L5	Translation, ribosomal structure and biogenesis [J]	Ribosome	Collinsella	Genus	Actinobacteria	Coriobacteriia	Coriobacteriales	Coriobacteriaceae	Collinsella		0.129178331	0.01547835	*
**258**	18955	MH0131_GL0174780	COG0334	Glutamate dehydrogenase/leucine dehydrogenase	Amino acid transport and metabolism [E]	Alanine, aspartate and glutamate metabolism	Bifidobacterium adolescentis	Species	Actinobacteria	Actinobacteria	Bifidobacteriales	Bifidobacteriaceae	Bifidobacterium	Bifidobacterium adolescentis	-0.151239723	0.062106863	
**259**	18959	MH0131_GL0175176	COG1882	Pyruvate-formate lyase	Energy production and conversion [C]	Pyruvate metabolism	Collinsella	Genus	Actinobacteria	Coriobacteriia	Coriobacteriales	Coriobacteriaceae	Collinsella		0.32657633	0.000213822	***
**260**	19189	MH0136_GL0083951	COG1904	Glucuronate isomerase	Carbohydrate transport and metabolism [G]	Pentose and glucuronate interconversions	Faecalibacterium prausnitzii	Species	Firmicutes	Clostridia	Clostridiales	Ruminococcaceae	Faecalibacterium	Faecalibacterium prausnitzii	0.14724216	0.033922808	*
**261**	19307	MH0139_GL0041455	COG0166	Glucose-6-phosphate isomerase	Carbohydrate transport and metabolism [G]	Glycolysis / Gluconeogenesis	Clostridiales	Order	Firmicutes	Clostridia	Clostridiales				-0.041113966	0.653117695	
**262**	19410	MH0140_GL0123359	COG0205	6-phosphofructokinase	Carbohydrate transport and metabolism [G]	Glycolysis / Gluconeogenesis	Ruminococcus bromii	Species	Firmicutes	Clostridia	Clostridiales	Ruminococcaceae	Ruminococcus	Ruminococcus bromii	0.058809685	0.317390783	
**263**	19720	MH0145_GL0031362			NAN	Galactose metabolism	Ruminococcus bromii	Species	Firmicutes	Clostridia	Clostridiales	Ruminococcaceae	Ruminococcus	Ruminococcus bromii	-0.108888899	0.125160888	
**264**	19738	MH0145_GL0085935	COG5263	Glucan-binding domain (YG repeat)	Carbohydrate transport and metabolism [G]	NAN	Clostridiales	Order	Firmicutes	Clostridia	Clostridiales				0.109311534	0.079931633	
**265**	19764	MH0145_GL0159811	COG1109	Phosphomannomutase	Carbohydrate transport and metabolism [G]	Glycolysis / Gluconeogenesis	Clostridiales	Order	Firmicutes	Clostridia	Clostridiales				-0.069743714	0.124162291	
**266**	19869	MH0147_GL0083186	COG4166	ABC-type oligopeptide transport system, periplasmic component	Amino acid transport and metabolism [E]	ABC transporters	Fusicatenibacter saccharivorans	Species	Firmicutes	Clostridia	Clostridiales	Lachnospiraceae	Fusicatenibacter	Fusicatenibacter saccharivorans	0.125497593	0.081605538	
**267**	20073	MH0149_GL0094229	COG1080	Phosphoenolpyruvate-protein kinase (PTS system EI component in bacteria)	Carbohydrate transport and metabolism [G]	Pyruvate metabolism	Faecalibacterium prausnitzii	Species	Firmicutes	Clostridia	Clostridiales	Ruminococcaceae	Faecalibacterium	Faecalibacterium prausnitzii	-0.357182122	0.008884528	**
**268**	20414	MH0442_GL0264877	COG1592	Rubrerythrin	Energy production and conversion [C]	Oxidative phosphorylation	Clostridiales	Order	Firmicutes	Clostridia	Clostridiales				-0.050441433	0.306798918	

**269**	20460	MH0157_GL0012420	COG0191	Fructose/tagatose bisphosphate aldolase	Carbohydrate transport and metabolism [G]	Glycolysis / Gluconeogenesis	Lachnospiraceae	Family	Firmicutes	Clostridia	Clostridiales	Lachnospiraceae			-0.01189173	0.89015764	
**270**	20476	MH0157_GL0087463	COG0745	DNA-binding response regulator, OmpR family, contains REC and winged-helix (wHTH) domain	Signal transduction mechanisms [T]	NAN	Prevotella copri	Species	Bacteroidetes	Bacteroidia	Bacteroidales	Prevotellaceae	Prevotella	Prevotella copri	-0.009022275	0.927515046	
**271**	20577	MH0161_GL0001231	COG3842	ABC-type Fe3+/spermidine/putrescine transport systems, ATPase components	Amino acid transport and metabolism [E]	ABC transporters	Bifidobacterium	Genus	Actinobacteria	Actinobacteria	Bifidobacteriales	Bifidobacteriaceae	Bifidobacterium		-0.001698193	0.981355266	
**272**	20644	MH0161_GL0061147	COG0115	Branched-chain amino acid aminotransferase/4-amino-4-deoxychorismate lyase	Coenzyme transport and metabolism [H]	Valine, leucine and isoleucine degradation	Bifidobacterium adolescentis	Species	Actinobacteria	Actinobacteria	Bifidobacteriales	Bifidobacteriaceae	Bifidobacterium	Bifidobacterium adolescentis	-0.030384519	0.70413461	
**273**	20650	MH0161_GL0069344	COG1653	ABC-type glycerol-3-phosphate transport system, periplasmic component	Carbohydrate transport and metabolism [G]	ABC transporters	Collinsella	Genus	Actinobacteria	Coriobacteriia	Coriobacteriales	Coriobacteriaceae	Collinsella		0.169932249	0.061448722	
**274**	20665	MH0161_GL0081156	COG0149	Triosephosphate isomerase	Carbohydrate transport and metabolism [G]	Glycolysis / Gluconeogenesis	Coprococcus comes	Species	Firmicutes	Clostridia	Clostridiales	Lachnospiraceae	Coprococcus	Coprococcus comes	-0.044212929	0.394608785	
**275**	20672	MH0161_GL0083626	COG0480	Translation elongation factor EF-G, a GTPase	Translation, ribosomal structure and biogenesis [J]	NAN	Bifidobacterium adolescentis	Species	Actinobacteria	Actinobacteria	Bifidobacteriales	Bifidobacteriaceae	Bifidobacterium	Bifidobacterium adolescentis	-0.163977822	0.116057698	
**276**	20712	MH0161_GL0110049	COG2407	L-fucose isomerase or related protein	Carbohydrate transport and metabolism [G]	Fructose and mannose metabolism	Blautia obeum	Species	Firmicutes	Clostridia	Clostridiales	Lachnospiraceae	Blautia	Blautia obeum	-0.158336982	0.015577324	*
**277**	20757	MH0161_GL0142425	COG0148	Enolase	Carbohydrate transport and metabolism [G]	Glycolysis / Gluconeogenesis	Bifidobacterium adolescentis	Species	Actinobacteria	Actinobacteria	Bifidobacteriales	Bifidobacteriaceae	Bifidobacterium	Bifidobacterium adolescentis	-0.035604192	0.755221566	
**278**	20764	MH0161_GL0152524	COG1362	Aspartyl aminopeptidase	Amino acid transport and metabolism [E]	NAN	Faecalibacterium prausnitzii	Species	Firmicutes	Clostridia	Clostridiales	Ruminococcaceae	Faecalibacterium	Faecalibacterium prausnitzii	0.184591347	0.050066697	
**279**	20773	MH0161_GL0161160	COG0747	ABC-type transport system, periplasmic component	Amino acid transport and metabolism [E]	NAN	Polymorphum gilvum	Species	Proteobacteria	Alphaproteobacteria			Polymorphum	Polymorphum gilvum	0.042201391	0.444681341	
**280**	20810	MH0162_GL0053827	COG1102	Cytidylate kinase	Nucleotide transport and metabolism [F]	NAN	Subdoligranulum variabile	Species	Firmicutes	Clostridia	Clostridiales	Ruminococcaceae	Subdoligranulum	Subdoligranulum variabile	-0.107197122	0.091272503	
**281**	20867	MH0163_GL0059562	COG0246	Mannitol-1-phosphate/altronate dehydrogenases	Carbohydrate transport and metabolism [G]	Pentose and glucuronate interconversions	Subdoligranulum variabile	Species	Firmicutes	Clostridia	Clostridiales	Ruminococcaceae	Subdoligranulum	Subdoligranulum variabile	-0.079642533	0.317240526	
**282**	21083	MH0170_GL0014792	COG0235	Ribulose-5-phosphate 4-epimerase/Fuculose-1-phosphate aldolase	Carbohydrate transport and metabolism [G]	Pentose and glucuronate interconversions	Clostridiales	Order	Firmicutes	Clostridia	Clostridiales				0.163490273	0.010312633	*
**283**	21098	MH0170_GL0056715	COG1145	Ferredoxin	Energy production and conversion [C]	Glycolysis / Gluconeogenesis	Fusicatenibacter saccharivorans	Species	Firmicutes	Clostridia	Clostridiales	Lachnospiraceae	Fusicatenibacter	Fusicatenibacter saccharivorans	-0.150653275	0.03978377	*
**284**	21298	MH0177_GL0076497	COG0183	Acetyl-CoA acetyltransferase	Lipid transport and metabolism [I]	Fatty acid degradation	Faecalibacterium prausnitzii	Species	Firmicutes	Clostridia	Clostridiales	Ruminococcaceae	Faecalibacterium	Faecalibacterium prausnitzii	-0.010614241	0.904102312	
**285**	21491	MH0182_GL0009170	COG0050	Translation elongation factor EF-Tu, a GTPase	Translation, ribosomal structure and biogenesis [J]	Plant-pathogen interaction	Clostridiales	Order	Firmicutes	Clostridia	Clostridiales				-0.084672083	0.238332348	
**286**	21665	MH0184_GL0118471	COG1653	ABC-type glycerol-3-phosphate transport system, periplasmic component	Carbohydrate transport and metabolism [G]	ABC transporters	Subdoligranulum variabile	Species	Firmicutes	Clostridia	Clostridiales	Ruminococcaceae	Subdoligranulum	Subdoligranulum variabile	-0.205512164	0.05097065	
**287**	21666	MH0184_GL0127793	COG0092	Ribosomal protein S3	Translation, ribosomal structure and biogenesis [J]	Ribosome	Ruminococcus bromii	Species	Firmicutes	Clostridia	Clostridiales	Ruminococcaceae	Ruminococcus	Ruminococcus bromii	-0.156376797	0.014546772	*
**288**	21833	MH0188_GL0007212	COG0039	Malate/lactate dehydrogenase	Energy production and conversion [C]	Glycolysis / Gluconeogenesis	Bifidobacterium	Genus	Actinobacteria	Actinobacteria	Bifidobacteriales	Bifidobacteriaceae	Bifidobacterium		0.071056846	0.439958732	
**289**	21865	MH0188_GL0018903	COG0473	Isocitrate/isopropylmalate dehydrogenase	Amino acid transport and metabolism [E]	Citrate cycle (TCA cycle)	Bifidobacterium	Genus	Actinobacteria	Actinobacteria	Bifidobacteriales	Bifidobacteriaceae	Bifidobacterium		0.02433549	0.634663824	
**290**	21893	MH0188_GL0025027	COG1653	ABC-type glycerol-3-phosphate transport system, periplasmic component	Carbohydrate transport and metabolism [G]	ABC transporters	Bifidobacterium	Genus	Actinobacteria	Actinobacteria	Bifidobacteriales	Bifidobacteriaceae	Bifidobacterium		-0.044797849	0.520164338	
**291**	21896	MH0188_GL0025245	COG0021	Transketolase	Carbohydrate transport and metabolism [G]	Pentose phosphate pathway	Bifidobacterium	Genus	Actinobacteria	Actinobacteria	Bifidobacteriales	Bifidobacteriaceae	Bifidobacterium		-0.125363401	0.138239752	
**292**	21902	MH0188_GL0028315	COG1653	ABC-type glycerol-3-phosphate transport system, periplasmic component	Carbohydrate transport and metabolism [G]	ABC transporters	Clostridiales	Order	Firmicutes	Clostridia	Clostridiales				-0.070294589	0.297096526	
**293**	21905	MH0188_GL0029398	COG0528	Uridylate kinase	Nucleotide transport and metabolism [F]	Pyrimidine metabolism	Bifidobacterium	Genus	Actinobacteria	Actinobacteria	Bifidobacteriales	Bifidobacteriaceae	Bifidobacterium		-0.018581235	0.762532143	
**294**	21906	MH0188_GL0029399	COG0264	Translation elongation factor EF-Ts	Translation, ribosomal structure and biogenesis [J]	NAN	Bifidobacterium	Genus	Actinobacteria	Actinobacteria	Bifidobacteriales	Bifidobacteriaceae	Bifidobacterium		0.056933897	0.463235232	
**295**	21946	MH0188_GL0043950	COG1087	UDP-glucose 4-epimerase	Cell wall/membrane/envelope biogenesis [M]	Galactose metabolism	Bacteria	Superkingdom							-0.041964717	0.632247164	
**296**	21975	MH0188_GL0054468	COG0172	Seryl-tRNA synthetase	Translation, ribosomal structure and biogenesis [J]	Aminoacyl-tRNA biosynthesis	Bifidobacterium	Genus	Actinobacteria	Actinobacteria	Bifidobacteriales	Bifidobacteriaceae	Bifidobacterium		-0.091564791	0.205417175	
**297**	21984	MH0188_GL0055295	COG1653	ABC-type glycerol-3-phosphate transport system, periplasmic component	Carbohydrate transport and metabolism [G]	ABC transporters	Bifidobacterium	Genus	Actinobacteria	Actinobacteria	Bifidobacteriales	Bifidobacteriaceae	Bifidobacterium		0.319113625	0.004557081	**
**298**	22012	MH0188_GL0064273	COG1879	ABC-type sugar transport system, periplasmic component, contains N-terminal xre family HTH domain	Carbohydrate transport and metabolism [G]	ABC transporters	Blautia obeum	Species	Firmicutes	Clostridia	Clostridiales	Lachnospiraceae	Blautia	Blautia obeum	-0.079736285	0.226203198	
**299**	22018	MH0188_GL0066755	COG0103	Ribosomal protein S9	Translation, ribosomal structure and biogenesis [J]	Ribosome	Bifidobacterium	Genus	Actinobacteria	Actinobacteria	Bifidobacteriales	Bifidobacteriaceae	Bifidobacterium		0.013914269	0.808735832	
**300**	22024	MH0188_GL0067094	COG0086	DNA-directed RNA polymerase, beta' subunit/160 kD subunit	Transcription [K]	Purine metabolism	Bifidobacterium	Genus	Actinobacteria	Actinobacteria	Bifidobacteriales	Bifidobacteriaceae	Bifidobacterium		-0.104151827	0.127364301	
**301**	22027	MH0188_GL0067976	COG1129	ABC-type sugar transport system, ATPase component	Carbohydrate transport and metabolism [G]	ABC transporters	Bifidobacterium	Genus	Actinobacteria	Actinobacteria	Bifidobacteriales	Bifidobacteriaceae	Bifidobacterium		0.024171026	0.790606517	
**302**	22030	MH0188_GL0069412	COG0282	Acetate kinase	Energy production and conversion [C]	Taurine and hypotaurine metabolism	Bifidobacterium	Genus	Actinobacteria	Actinobacteria	Bifidobacteriales	Bifidobacteriaceae	Bifidobacterium		0.103115754	0.22012233	
**303**	22039	MH0188_GL0071819	COG0747	ABC-type transport system, periplasmic component	Amino acid transport and metabolism [E]	ABC transporters	Bifidobacterium longum	Species	Actinobacteria	Actinobacteria	Bifidobacteriales	Bifidobacteriaceae	Bifidobacterium	Bifidobacterium longum	0.062476959	0.453579537	
**304**	22064	MH0188_GL0082698	COG1544	Ribosome-associated translation inhibitor RaiA	Translation, ribosomal structure and biogenesis [J]	NAN	Bifidobacterium	Genus	Actinobacteria	Actinobacteria	Bifidobacteriales	Bifidobacteriaceae	Bifidobacterium		0.057358017	0.44994046	
**305**	22074	NLF007_GL0024227	COG0426	Flavorubredoxin	Energy production and conversion [C]	NAN	Clostridiales	Order	Firmicutes	Clostridia	Clostridiales				0.030864793	0.659081392	

**306**	22087	MH0188_GL0093557	COG0359	Ribosomal protein L9	Translation, ribosomal structure and biogenesis [J]	Ribosome	Bifidobacterium	Genus	Actinobacteria	Actinobacteria	Bifidobacteriales	Bifidobacteriaceae	Bifidobacterium		0.041297396	0.548005854	
**307**	22090	MH0188_GL0095502	COG0174	Glutamine synthetase	Amino acid transport and metabolism [E]	Alanine, aspartate and glutamate metabolism	Bifidobacterium	Genus	Actinobacteria	Actinobacteria	Bifidobacteriales	Bifidobacteriaceae	Bifidobacterium		-0.027587317	0.82235006	
**308**	22169	MH0188_GL0115288	COG0235	Ribulose-5-phosphate 4-epimerase/Fuculose-1-phosphate aldolase	Carbohydrate transport and metabolism [G]	Pentose and glucuronate interconversions	Bifidobacterium	Genus	Actinobacteria	Actinobacteria	Bifidobacteriales	Bifidobacteriaceae	Bifidobacterium		0.049018419	0.445334874	
**309**	22205	MH0188_GL0126744	COG0459	Chaperonin GroEL (HSP60 family)	Posttranslational modification, protein turnover, chaperones [O]	RNA degradation	Bifidobacterium	Genus	Actinobacteria	Actinobacteria	Bifidobacteriales	Bifidobacteriaceae	Bifidobacterium		0.032486814	0.750138335	
**310**	22221	MH0188_GL0133256	COG1087	UDP-glucose 4-epimerase	Cell wall/membrane/envelope biogenesis [M]	Galactose metabolism	Bifidobacterium longum	Species	Actinobacteria	Actinobacteria	Bifidobacteriales	Bifidobacteriaceae	Bifidobacterium	Bifidobacterium longum	0.090964669	0.3510444	
**311**	22227	MH0188_GL0137837	COG0198	Ribosomal protein L24	Translation, ribosomal structure and biogenesis [J]	Ribosome	Bifidobacterium	Genus	Actinobacteria	Actinobacteria	Bifidobacteriales	Bifidobacteriaceae	Bifidobacterium		-0.008145016	0.89981717	
**312**	22236	MH0188_GL0141556	COG0050	Translation elongation factor EF-Tu, a GTPase	Translation, ribosomal structure and biogenesis [J]	Plant-pathogen interaction	Collinsella	Genus	Actinobacteria	Coriobacteriia	Coriobacteriales	Coriobacteriaceae	Collinsella		0.129723902	0.284085286	
**313**	22249	MH0188_GL0143190	COG0176	Transaldolase	Carbohydrate transport and metabolism [G]	Pentose phosphate pathway	Bifidobacterium	Genus	Actinobacteria	Actinobacteria	Bifidobacteriales	Bifidobacteriaceae	Bifidobacterium		0.006985263	0.927481273	
**314**	22254	MH0188_GL0145450	COG1882	Pyruvate-formate lyase	Energy production and conversion [C]	Pyruvate metabolism	Bifidobacterium adolescentis	Species	Actinobacteria	Actinobacteria	Bifidobacteriales	Bifidobacteriaceae	Bifidobacterium	Bifidobacterium adolescentis	-0.146872721	0.223524954	
**315**	22257	MH0188_GL0146214	COG0459	Chaperonin GroEL (HSP60 family)	Posttranslational modification, protein turnover, chaperones [O]	RNA degradation	Collinsella	Genus	Actinobacteria	Coriobacteriia	Coriobacteriales	Coriobacteriaceae	Collinsella		0.162950819	0.184734037	
**316**	22288	MH0188_GL0156393	COG0104	Adenylosuccinate synthase	Nucleotide transport and metabolism [F]	Purine metabolism	Clostridiales	Order	Firmicutes	Clostridia	Clostridiales				-0.122544519	0.007324853	**
**317**	22302	MH0188_GL0160346	COG0058	Glucan phosphorylase	Carbohydrate transport and metabolism [G]	Starch and sucrose metabolism	Bifidobacterium adolescentis	Species	Actinobacteria	Actinobacteria	Bifidobacteriales	Bifidobacteriaceae	Bifidobacterium	Bifidobacterium adolescentis	-0.103395465	0.358302615	
**318**	22338	MH0188_GL0176742	COG0202	DNA-directed RNA polymerase, alpha subunit/40 kD subunit	Transcription [K]	Purine metabolism	Collinsella	Genus	Actinobacteria	Coriobacteriia	Coriobacteriales	Coriobacteriaceae	Collinsella		0.079555455	0.30359286	
**319**	22376	MH0188_GL0186972	COG0480	Translation elongation factor EF-G, a GTPase	Translation, ribosomal structure and biogenesis [J]	NAN	Clostridiales	Order	Firmicutes	Clostridia	Clostridiales				-0.097913509	0.046152034	*
**320**	22394	MH0188_GL0191505	COG1454	Alcohol dehydrogenase, class IV	Energy production and conversion [C]	Glycolysis / Gluconeogenesis	Bifidobacterium	Genus	Actinobacteria	Actinobacteria	Bifidobacteriales	Bifidobacteriaceae	Bifidobacterium		-0.107609758	0.259546202	
**321**	22395	MH0188_GL0191508	COG0088	Ribosomal protein L4	Translation, ribosomal structure and biogenesis [J]	Ribosome	Bacteria	Superkingdom							-0.166931176	0.024202614	*
**322**	22489	MH0189_GL0094693	COG4690	Dipeptidase	Amino acid transport and metabolism [E]	NAN	Bifidobacterium adolescentis	Species	Actinobacteria	Actinobacteria	Bifidobacteriales	Bifidobacteriaceae	Bifidobacterium	Bifidobacterium adolescentis	-0.160165498	0.0368359	*
**323**	22497	MH0189_GL0100565	COG0050	Translation elongation factor EF-Tu, a GTPase	Translation, ribosomal structure and biogenesis [J]	Plant-pathogen interaction	Bifidobacterium	Genus	Actinobacteria	Actinobacteria	Bifidobacteriales	Bifidobacteriaceae	Bifidobacterium		-0.167633825	0.083470829	
**324**	22707	MH0192_GL0016996	COG1129	ABC-type sugar transport system, ATPase component	Carbohydrate transport and metabolism [G]	ABC transporters	Clostridiales	Order	Firmicutes	Clostridia	Clostridiales				-0.074862487	0.306763313	
**325**	22837	MH0193_GL0010680	COG0480	Translation elongation factor EF-G, a GTPase	Translation, ribosomal structure and biogenesis [J]	NAN	Bifidobacterium	Genus	Actinobacteria	Actinobacteria	Bifidobacteriales	Bifidobacteriaceae	Bifidobacterium		0.038164439	0.777453823	
**326**	22838	MH0193_GL0010681	COG0050	Translation elongation factor EF-Tu, a GTPase	Translation, ribosomal structure and biogenesis [J]	Plant-pathogen interaction	Bifidobacterium longum	Species	Actinobacteria	Actinobacteria	Bifidobacteriales	Bifidobacteriaceae	Bifidobacterium	Bifidobacterium longum	-0.009317283	0.908659024	
**327**	22866	MH0193_GL0048220	COG0282	Acetate kinase(	Energy production and conversion [C]	Taurine and hypotaurine metabolism	Ruminococcus bromii	Species	Firmicutes	Clostridia	Clostridiales	Ruminococcaceae	Ruminococcus	Ruminococcus bromii	0.044484944	0.484054373	
**328**	22871	MH0193_GL0052517	COG1879	ABC-type sugar transport system, periplasmic component, contains N-terminal xre family HTH domain	Carbohydrate transport and metabolism [G]	ABC transporters	Bifidobacterium	Genus	Actinobacteria	Actinobacteria	Bifidobacteriales	Bifidobacteriaceae	Bifidobacterium		0.019495874	0.832981026	
**329**	22874	MH0193_GL0053065			NAN	NAN	Clostridiales	Order	Firmicutes	Clostridia	Clostridiales				0.061440805	0.266390799	
**330**	22987	MH0193_GL0165661	COG0334	Glutamate dehydrogenase/leucine dehydrogenase	Amino acid transport and metabolism [E]	Alanine, aspartate and glutamate metabolism	Bifidobacterium	Genus	Actinobacteria	Actinobacteria	Bifidobacteriales	Bifidobacteriaceae	Bifidobacterium		-0.087194357	0.289213876	
**331**	23014	MH0193_GL0186495	COG1454	Alcohol dehydrogenase, class IV	Energy production and conversion [C]	Glycolysis / Gluconeogenesis	Bifidobacterium	Genus	Actinobacteria	Actinobacteria	Bifidobacteriales	Bifidobacteriaceae	Bifidobacterium		0.115453604	0.33967653	
**332**	23023	MH0193_GL0193391	COG1653	ABC-type glycerol-3-phosphate transport system, periplasmic component	Carbohydrate transport and metabolism [G]	ABC transporters	Clostridiales	Order	Firmicutes	Clostridia	Clostridiales				-0.017541452	0.762463465	
**333**	23172	MH0196_GL0219356	COG4213	ABC-type xylose transport system, periplasmic component	Carbohydrate transport and metabolism [G]	ABC transporters	Clostridiales	Order	Firmicutes	Clostridia	Clostridiales				-0.1044012	0.064982463	
**334**	23262	MH0197_GL0121100	COG1653	ABC-type glycerol-3-phosphate transport system, periplasmic component	Carbohydrate transport and metabolism [G]	ABC transporters	Clostridiales bacterium KLE1615	Species	Firmicutes	Clostridia	Clostridiales			Clostridiales bacterium KLE1615	-0.03240661	0.757145493	
**335**	23510	MH0200_GL0014998	COG0280	Phosphotransacetylase	Energy production and conversion [C]	Taurine and hypotaurine metabolism	Clostridiales	Order	Firmicutes	Clostridia	Clostridiales				-0.05161116	0.35176281	
**336**	23525	MH0200_GL0037813	COG1145	Ferredoxin	Energy production and conversion [C]	Glycolysis / Gluconeogenesis	Clostridiales	Order	Firmicutes	Clostridia	Clostridiales				-0.177334967	0.053222617	
**337**	23643	MH0200_GL0183371	COG0099	Ribosomal protein S13	Translation, ribosomal structure and biogenesis [J]	Ribosome	Bifidobacteriaceae	Family	Actinobacteria	Actinobacteria	Bifidobacteriales	Bifidobacteriaceae			-0.078774953	0.262670868	
**338**	23687	MH0203_GL0013763	COG0057	Glyceraldehyde-3-phosphate dehydrogenase/erythrose-4-phosphate dehydrogenase	Carbohydrate transport and metabolism [G]	Glycolysis / Gluconeogenesis	Bifidobacterium	Genus	Actinobacteria	Actinobacteria	Bifidobacteriales	Bifidobacteriaceae	Bifidobacterium		0.077371253	0.43311984	
**339**	23777	MH0203_GL0101461	COG0080	Ribosomal protein L11	Translation, ribosomal structure and biogenesis [J]	Ribosome	Bifidobacterium bifidum	Species	Actinobacteria	Actinobacteria	Bifidobacteriales	Bifidobacteriaceae	Bifidobacterium	Bifidobacterium bifidum	-0.033847506	0.567058126	
**340**	23809	MH0203_GL0120151	COG0460	Homoserine dehydrogenase	Amino acid transport and metabolism [E]	Glycine, serine and threonine metabolism	Bifidobacterium	Genus	Actinobacteria	Actinobacteria	Bifidobacteriales	Bifidobacteriaceae	Bifidobacterium		-0.003721303	0.951470205	
**341**	23820	MH0203_GL0130171	COG0235	Ribulose-5-phosphate 4-epimerase/Fuculose-1-phosphate aldolase	Carbohydrate transport and metabolism [G]	Pentose and glucuronate interconversions	Blautia obeum	Species	Firmicutes	Clostridia	Clostridiales	Lachnospiraceae	Blautia	Blautia obeum	-0.134237286	0.0571236	

**342**	23823	MH0203_GL0133062	COG0033	Phosphoglucomutase	Carbohydrate transport and metabolism [G]	Glycolysis / Gluconeogenesis	Bifidobacterium	Genus	Actinobacteria	Actinobacteria	Bifidobacteriales	Bifidobacteriaceae	Bifidobacterium		0.02745405	0.781509489	
**343**	23826	MH0203_GL0134326			NAN	NAN	Dorea formicigenerans	Species	Firmicutes	Clostridia	Clostridiales	Lachnospiraceae	Dorea	Dorea formicigenerans	0.194937535	0.004013323	**
**344**	23919	MH0203_GL0245882	COG0366	Glycosidase	Carbohydrate transport and metabolism [G]	NAN	Bifidobacterium longum	Species	Actinobacteria	Actinobacteria	Bifidobacteriales	Bifidobacteriaceae	Bifidobacterium	Bifidobacterium longum	-0.060410222	0.594782484	
**345**	23949	MH0204_GL0017355	COG0186	Ribosomal protein S17	Translation, ribosomal structure and biogenesis [J]	Ribosome	Faecalibacterium prausnitzii	Species	Firmicutes	Clostridia	Clostridiales	Ruminococcaceae	Faecalibacterium	Faecalibacterium prausnitzii	-0.091170793	0.057266073	
**346**	24576	MH0220_GL0013104	COG1882	Pyruvate-formate lyase	Energy production and conversion [C]	Pyruvate metabolism	Blautia obeum	Species	Firmicutes	Clostridia	Clostridiales	Lachnospiraceae	Blautia	Blautia obeum	0.153460851	0.053349374	
**347**	24593	MH0220_GL0083105	COG0049	Ribosomal protein S7	Translation, ribosomal structure and biogenesis [J]	Ribosome	Collinsella	Genus	Actinobacteria	Coriobacteriia	Coriobacteriales	Coriobacteriaceae	Collinsella		0.017599312	0.754498694	
**348**	24695	MH0221_GL0065038			NAN	NAN	Bilophila wadsworthia	Species	Proteobacteria	Deltaproteobacteria	Desulfovibrionales	Desulfovibrionaceae	Bilophila	Bilophila wadsworthia	0.135077788	0.010304408	*
**349**	24743	MH0222_GL0076490	COG1145	Ferredoxin	Energy production and conversion [C]	Glycolysis / Gluconeogenesis	Clostridiales	Order	Firmicutes	Clostridia	Clostridiales				0.059467247	0.429740941	
**350**	24946	MH0227_GL0100580	COG0057	Glyceraldehyde-3-phosphate dehydrogenase/erythrose-4-phosphate dehydrogenase	Carbohydrate transport and metabolism [G]	Glycolysis / Gluconeogenesis	Bacteroides eggerthii	Species	Bacteroidetes	Bacteroidia	Bacteroidales	Bacteroidaceae	Bacteroides	Bacteroides eggerthii	0.357592478	0.002817758	**
**351**	24978	MH0227_GL0147089			NAN	NAN	Subdoligranulum variabile	Species	Firmicutes	Clostridia	Clostridiales	Ruminococcaceae	Subdoligranulum	Subdoligranulum variabile	-0.222662424	0.003146434	**
**352**	25045	MH0229_GL0083635	COG1185	Polyribonucleotide nucleotidyltransferase (polynucleotide phosphorylase)	Translation, ribosomal structure and biogenesis [J]	Purine metabolism	Faecalibacterium prausnitzii	Species	Firmicutes	Clostridia	Clostridiales	Ruminococcaceae	Faecalibacterium	Faecalibacterium prausnitzii	-0.031782387	0.636164835	
**353**	25052	MH0229_GL0094180	COG4939	Major membrane immunogen, membrane-anchored lipoprotein	Function unknown [S]	Citrate cycle (TCA cycle)	Faecalibacterium prausnitzii	Species	Firmicutes	Clostridia	Clostridiales	Ruminococcaceae	Faecalibacterium	Faecalibacterium prausnitzii	0.0395815	0.563141342	
**354**	25430	MH0233_GL0035314	COG1879	ABC-type sugar transport system, periplasmic component, contains N-terminal xre family HTH domain	Carbohydrate transport and metabolism [G]	ABC transporters	[Ruminococcus] torques	Species	Firmicutes	Clostridia	Clostridiales	Lachnospiraceae	Blautia	[Ruminococcus] torques	0.171441676	0.076620816	
**355**	25495	MH0233_GL0079986	COG1882	Pyruvate-formate lyase	Energy production and conversion [C]	Pyruvate metabolism	[Ruminococcus] torques	Species	Firmicutes	Clostridia	Clostridiales	Lachnospiraceae	Blautia	[Ruminococcus] torques	0.169501139	0.092847935	
**356**	25534	MH0233_GL0108503	COG1653	ABC-type glycerol-3-phosphate transport system, periplasmic component	Carbohydrate transport and metabolism [G]	NAN	Blautia	Genus	Firmicutes	Clostridia	Clostridiales	Lachnospiraceae	Blautia		-0.087572953	0.344897495	
**357**	25618	MH0236_GL0022013	COG0191	Fructose/tagatose bisphosphate aldolase	Carbohydrate transport and metabolism [G]	Glycolysis / Gluconeogenesis	Faecalibacterium prausnitzii	Species	Firmicutes	Clostridia	Clostridiales	Ruminococcaceae	Faecalibacterium	Faecalibacterium prausnitzii	-0.043537996	0.533563597	
**358**	25664	MH0238_GL0027853	COG1653	ABC-type glycerol-3-phosphate transport system, periplasmic component	Carbohydrate transport and metabolism [G]	ABC transporters	Firmicutes	Species	Firmicutes	Clostridia	Clostridiales	Lachnospiraceae	Butyrivibrio	Butyrivibrio crossotus	0.040926766	0.563179928	
**359**	25714	MH0238_GL0165578	COG1454	Alcohol dehydrogenase, class IV	Energy production and conversion [C]	Glycolysis / Gluconeogenesis	Ruminococcaceae	Species	Firmicutes	Clostridia	Clostridiales	Ruminococcaceae	Faecalibacterium	Faecalibacterium prausnitzii	-0.10644444	0.138905603	
**360**	25756	MH0239_GL0018928	COG1145	Ferredoxin	Energy production and conversion [C]	Glycolysis / Gluconeogenesis	Clostridiales	Order	Firmicutes	Clostridia	Clostridiales				-0.227777225	0.004064887	**
**361**	25836	MH0239_GL0084186	COG1145	Ferredoxin	Energy production and conversion [C]	Glycolysis / Gluconeogenesis	Clostridiales	Species	Firmicutes	Clostridia	Clostridiales	Ruminococcaceae	Faecalibacterium	Faecalibacterium prausnitzii	-0.014574602	0.84634049	
**362**	25842	MH0239_GL0088549	COG0747	ABC-type transport system, periplasmic component	Amino acid transport and metabolism [E]	NAN	Clostridiales	Order	Firmicutes	Clostridia	Clostridiales				-0.015687173	0.80744453	
**363**	25966	MH0243_GL0019682	COG0149	Triosephosphate isomerase	Carbohydrate transport and metabolism [G]	Glycolysis / Gluconeogenesis	Ruminococcus bromii	Species	Firmicutes	Clostridia	Clostridiales	Ruminococcaceae	Ruminococcus	Ruminococcus bromii	0.208314268	0.045178449	*
**364**	26279	MH0246_GL0124498	COG1070	Sugar (pentulose or hexulose) kinase	Carbohydrate transport and metabolism [G]	Pentose phosphate pathway	Bifidobacterium	Genus	Actinobacteria	Actinobacteria	Bifidobacteriales	Bifidobacteriaceae	Bifidobacterium		-0.090065403	0.288551443	
**365**	26284	MH0246_GL0129256			NAN	NAN	Oscillibacter sp. KLE 1745	Species	Firmicutes	Clostridia	Clostridiales	Oscillospiraceae	Oscillibacter	Oscillibacter sp. KLE 1745	0.145000781	0.225362792	
**366**	26552	MH0251_GL0023414	COG1024	Enoyl-CoA hydratase/carnithine racemase	Lipid transport and metabolism [I]	NAN	Faecalibacterium prausnitzii	Species	Firmicutes	Clostridia	Clostridiales	Ruminococcaceae	Faecalibacterium	Faecalibacterium prausnitzii	-0.125782415	0.201477795	
**367**	26559	MH0251_GL0036895	COG0427	Acyl-CoA hydrolase	Energy production and conversion [C]	Pyruvate metabolism	Faecalibacterium prausnitzii	Species	Firmicutes	Clostridia	Clostridiales	Ruminococcaceae	Faecalibacterium	Faecalibacterium prausnitzii	0.156109413	0.023202789	*
**368**	26571	MH0251_GL0069201	COG1653	ABC-type glycerol-3-phosphate transport system, periplasmic component	Carbohydrate transport and metabolism [G]	NAN	Clostridiales	Order	Firmicutes	Clostridia	Clostridiales				-0.030659477	0.654064477	
**369**	26605	MH0251_GL0128679	COG1614	CO dehydrogenase/acetyl-CoA synthase beta subunit	Energy production and conversion [C]	Methane metabolism	Clostridiales	Order	Firmicutes	Clostridia	Clostridiales				-0.099778984	0.037042647	*
**370**	26897	MH0259_GL0033209	COG1653	ABC-type glycerol-3-phosphate transport system, periplasmic component	Carbohydrate transport and metabolism [G]	ABC transporters	Bifidobacterium adolescentis	Species	Actinobacteria	Actinobacteria	Bifidobacteriales	Bifidobacteriaceae	Bifidobacterium	Bifidobacterium adolescentis	-0.145459286	0.162213932	
**371**	27099	MH0262_GL0083048	COG3716	Phosphotransferase system, mannose/fructose/N-acetylgalactosamine-specific component IID	Carbohydrate transport and metabolism [G]	Fructose and mannose metabolism	Collinsella	Genus	Actinobacteria	Coriobacteriia	Coriobacteriales	Coriobacteriaceae	Collinsella		0.114361311	0.218249901	
**372**	27223	MH0265_GL0114475	COG0094	Ribosomal protein L5	Translation, ribosomal structure and biogenesis [J]	Ribosome	Bifidobacterium	Genus	Actinobacteria	Actinobacteria	Bifidobacteriales	Bifidobacteriaceae	Bifidobacterium		0.07315157	0.316938159	
**373**	27643	MH0272_GL0188768	COG0097	Ribosomal protein L6P/L9E	Translation, ribosomal structure and biogenesis [J]	Ribosome	Bifidobacterium	Genus	Actinobacteria	Actinobacteria	Bifidobacteriales	Bifidobacteriaceae	Bifidobacterium		-0.100779757	0.168708187	
**374**	27673	MH0274_GL0008807	COG1080	Phosphoenolpyruvate-protein kinase (PTS system EI component in bacteria)	Carbohydrate transport and metabolism [G]	Pyruvate metabolism	Clostridiales	Order	Firmicutes	Clostridia	Clostridiales				-0.164485747	0.072438505	
**375**	27677	MH0274_GL0010540	COG0056	FoF1-type ATP synthase, alpha subunit	Energy production and conversion [C]	Oxidative phosphorylation	Clostridiales	Order	Firmicutes	Clostridia	Clostridiales				-0.040028528	0.39242113	
**376**	27731	MH0274_GL0075860	COG0539	Ribosomal protein S1	Translation, ribosomal structure and biogenesis [J]	Ribosome	Bifidobacterium	Genus	Actinobacteria	Actinobacteria	Bifidobacteriales	Bifidobacteriaceae	Bifidobacterium		-0.163393776	0.111915105	
**377**	27827	MH0274_GL0171300	COG0334	Glutamate dehydrogenase/leucine dehydrogenase	Amino acid transport and metabolism [E]	Alanine, aspartate and glutamate metabolism	Clostridiales	Order	Firmicutes	Clostridia	Clostridiales				-0.113859595	0.113984255	
**378**	27885	MH0275_GL0131386	COG0334	Glutamate dehydrogenase/leucine dehydrogenase	Amino acid transport and metabolism [E]	Alanine, aspartate and glutamate metabolism	Clostridiales	Order	Firmicutes	Clostridia	Clostridiales				0.019876375	0.721102534	
**379**	28126	MH0280_GL0137988	COG0088	Ribosomal protein L4	Translation, ribosomal structure and biogenesis [J]	Ribosome	Subdoligranulum variabile	Species	Firmicutes	Clostridia	Clostridiales	Ruminococcaceae	Subdoligranulum	Subdoligranulum variabile	-0.112589312	0.049739611	*

**380**	28314	MH0284_GL0116366	COG0183	Acetyl-CoA acetyltransferase	Lipid transport and metabolism [I]	Fatty acid degradation	Clostridiales	Order	Firmicutes	Clostridia	Clostridiales				-0.173069413	0.174653996	
**381**	28480	MH0288_GL0054326	COG4774	Outer membrane receptor for monomeric catechols	Inorganic ion transport and metabolism [P]	NAN	Bacteroides vulgatus	Species	Bacteroidetes	Bacteroidia	Bacteroidales	Bacteroidaceae	Bacteroides	Bacteroides vulgatus	0.255915009	0.047848168	*
**382**	28510	MH0288_GL0093098	COG1024	Enoyl-CoA hydratase/carnithine racemase	Lipid transport and metabolism [I]	Fatty acid degradation	Lachnospiraceae	Family	Firmicutes	Clostridia	Clostridiales	Lachnospiraceae			0.087057365	0.2813949	
**383**	28604	N051A_GL0052937			NAN	NAN	Faecalibacterium prausnitzii	Species	Firmicutes	Clostridia	Clostridiales	Ruminococcaceae	Faecalibacterium	Faecalibacterium prausnitzii	-0.11733677	0.310341135	
**384**	28653	MH0290_GL0142605	COG2407	L-fucose isomerase or related protein	Carbohydrate transport and metabolism [G]	Fructose and mannose metabolism	Lachnospiraceae	Family	Firmicutes	Clostridia	Clostridiales	Lachnospiraceae			0.057013709	0.468889457	
**385**	28676	MH0292_GL0072575			NAN	NAN	Clostridiales	Order	Firmicutes	Clostridia	Clostridiales				-0.010345908	0.879549443	
**386**	28707	MH0293_GL0072012	COG0031	Cysteine synthase	Amino acid transport and metabolism [E]	Glycine, serine and threonine metabolism	Clostridiales	Order	Firmicutes	Clostridia	Clostridiales				-0.044702472	0.50861498	
**387**	28712	MH0293_GL0090794	COG1080	Phosphoenolpyruvate-protein kinase (PTS system EI component in bacteria)	Carbohydrate transport and metabolism [G]	Pyruvate metabolism	Clostridiales	Order	Firmicutes	Clostridia	Clostridiales				-0.401036015	0.005392868	**
**388**	29633	MH0321_GL0040229	COG1350	Predicted alternative tryptophan synthase beta-subunit (paralog of TrpB)	Amino acid transport and metabolism [E]	Glycine, serine and threonine metabolism	Ruminococcus	Genus	Firmicutes	Clostridia	Clostridiales	Ruminococcaceae	Ruminococcus		-0.053005849	0.542587585	
**389**	29645	MH0321_GL0083392	COG0097	Ribosomal protein L6P/L9E	Translation, ribosomal structure and biogenesis [J]	Ribosome	Subdoligranulum variabile	Species	Firmicutes	Clostridia	Clostridiales	Ruminococcaceae	Subdoligranulum	Subdoligranulum variabile	-0.127139974	0.031670873	*
**390**	29780	MH0326_GL0040294	COG3959	Transketolase, N-terminal subunit	Carbohydrate transport and metabolism [G]	Pentose phosphate pathway	Clostridiales	Order	Firmicutes	Clostridia	Clostridiales				-0.049239907	0.361607345	
**391**	29830	MH0327_GL0023365	COG1866	Phosphoenolpyruvate carboxykinase, ATP-dependent	Energy production and conversion [C]	Glycolysis / Gluconeogenesis	Faecalibacterium prausnitzii	Species	Firmicutes	Clostridia	Clostridiales	Ruminococcaceae	Faecalibacterium	Faecalibacterium prausnitzii	0.015508933	0.83788927	
**392**	29843	MH0327_GL0049100	COG0443	Molecular chaperone DnaK (HSP70)	Posttranslational modification, protein turnover, chaperones [O]	RNA degradation	Collinsella aerofaciens	Species	Actinobacteria	Coriobacteriia	Coriobacteriales	Coriobacteriaceae	Collinsella	Collinsella aerofaciens	0.126708408	0.09657643	
**393**	29850	MH0327_GL0058501	COG0191	Fructose/tagatose bisphosphate aldolase	Carbohydrate transport and metabolism [G]	Glycolysis / Gluconeogenesis	Subdoligranulum variabile	Species	Firmicutes	Clostridia	Clostridiales	Ruminococcaceae	Subdoligranulum	Subdoligranulum variabile	-0.10200155	0.095896994	
**394**	30067	MH0330_GL0174601	COG1825	Ribosomal protein L25 (general stress protein Ctc)	Translation, ribosomal structure and biogenesis [J]	Ribosome	Bifidobacterium	Genus	Actinobacteria	Actinobacteria	Bifidobacteriales	Bifidobacteriaceae	Bifidobacterium		0.148374556	0.058122128	
**395**	30149	MH0333_GL0069706	COG4822	Cobalamin biosynthesis protein CbiK, Co2+ chelatase	Coenzyme transport and metabolism [H]	Porphyrin and chlorophyll metabolism	Clostridiales	Order	Firmicutes	Clostridia	Clostridiales				-0.078334001	0.312363242	
**396**	30175	MH0333_GL0121497	COG3209	Uncharacterized conserved protein RhaS, contains 28 RHS repeats	General function prediction only [R]	NAN	Blautia	Species	Firmicutes	Clostridia	Clostridiales	Lachnospiraceae	Blautia	Blautia wexlerae	0.252065478	0.078879836	
**397**	30545	MH0341_GL0000384	COG0297	Glycogen synthase(	Carbohydrate transport and metabolism [G]	Galactose metabolism	Faecalibacterium prausnitzii	Species	Firmicutes	Clostridia	Clostridiales	Ruminococcaceae	Faecalibacterium	Faecalibacterium prausnitzii	-0.134912591	0.087149189	
**398**	30556	MH0341_GL0013504	COG0176	Transaldolase	Carbohydrate transport and metabolism [G]	Pentose phosphate pathway	Bifidobacterium	Genus	Actinobacteria	Actinobacteria	Bifidobacteriales	Bifidobacteriaceae	Bifidobacterium		-0.058590798	0.644370996	
**399**	30576	MH0341_GL0038266	COG0469	Pyruvate kinase	Carbohydrate transport and metabolism [G]	Glycolysis / Gluconeogenesis	Bifidobacterium	Genus	Actinobacteria	Actinobacteria	Bifidobacteriales	Bifidobacteriaceae	Bifidobacterium		-0.101664131	0.205830535	
**400**	30617	MH0341_GL0088523	COG0281	Malic enzyme	Energy production and conversion [C]	Pyruvate metabolism	Clostridiales	Order	Firmicutes	Clostridia	Clostridiales				-0.037143052	0.626452022	
**401**	30623	MH0341_GL0099782	COG0050	Translation elongation factor EF-Tu, a GTPase(	Translation, ribosomal structure and biogenesis [J]	Plant-pathogen interaction	Bifidobacterium longum	Species	Actinobacteria	Actinobacteria	Bifidobacteriales	Bifidobacteriaceae	Bifidobacterium	Bifidobacterium longum	0.003941499	0.967394849	
**402**	30641	MH0341_GL0114479	COG0277	FAD/FMN-containing dehydrogenase	Energy production and conversion [C]	Pyruvate metabolism	Firmicutes	Phylum	Firmicutes						-0.023433255	0.75931218	
**403**	30834	MH0347_GL0010219	COG1653	ABC-type glycerol-3-phosphate transport system, periplasmic component	Carbohydrate transport and metabolism [G]	NAN	Bifidobacterium	Genus	Actinobacteria	Actinobacteria	Bifidobacteriales	Bifidobacteriaceae	Bifidobacterium		-0.037399586	0.752166361	
**404**	30909	MH0348_GL0122167	COG0085	DNA-directed RNA polymerase, beta subunit/140 kD subunit	Transcription [K]	Purine metabolism	Bifidobacterium	Genus	Actinobacteria	Actinobacteria	Bifidobacteriales	Bifidobacteriaceae	Bifidobacterium		-0.02865322	0.770279158	
**405**	30964	MH0350_GL0092393	COG2407	L-fucose isomerase or related protein	Carbohydrate transport and metabolism [G]	Fructose and mannose metabolism	Clostridiales	Order	Firmicutes	Clostridia	Clostridiales				-0.138867259	0.13519423	
**406**	31096	MH0355_GL0038120	COG1014	Pyruvate:ferredoxin oxidoreductase or related 2-oxoacid:ferredoxin oxidoreductase, gamma subunit	Energy production and conversion [C]	Glycolysis / Gluconeogenesis	Lachnospiraceae	Family	Firmicutes	Clostridia	Clostridiales	Lachnospiraceae			-0.114973221	0.160364929	
**407**	31170	MH0356_GL0041566	COG0057	Glyceraldehyde-3-phosphate dehydrogenase/erythrose-4-phosphate dehydrogenase(	Carbohydrate transport and metabolism [G]	Glycolysis / Gluconeogenesis	Bifidobacterium	Genus	Actinobacteria	Actinobacteria	Bifidobacteriales	Bifidobacteriaceae	Bifidobacterium		-0.121344957	0.068732513	
**408**	31210	MH0356_GL0130219	COG0055	FoF1-type ATP synthase, beta subunit	Energy production and conversion [C]	Oxidative phosphorylation	Bifidobacterium	Genus	Actinobacteria	Actinobacteria	Bifidobacteriales	Bifidobacteriaceae	Bifidobacterium		-0.076321506	0.417399806	
**409**	31211	MH0356_GL0130221	COG0056	FoF1-type ATP synthase, alpha subunit(1	Energy production and conversion [C]	Oxidative phosphorylation	Bifidobacterium	Genus	Actinobacteria	Actinobacteria	Bifidobacteriales	Bifidobacteriaceae	Bifidobacterium		-0.217273962	0.006975824	**
**410**	31216	MH0356_GL0133302	COG0459	Chaperonin GroEL (HSP60 family)	Posttranslational modification, protein turnover, chaperones [O]	RNA degradation	Bifidobacterium pseudocatenulatum	Species	Actinobacteria	Actinobacteria	Bifidobacteriales	Bifidobacteriaceae	Bifidobacterium	Bifidobacterium pseudocatenulatum	0.03745483	0.641606727	
**411**	31253	MH0356_GL0190005	COG0747	ABC-type transport system, periplasmic component	Amino acid transport and metabolism [E]	ABC transporters	Lachnospiraceae	Family	Firmicutes	Clostridia	Clostridiales	Lachnospiraceae			0.084222651	0.415527895	
**412**	31485	MH0359_GL0028504	COG0091	Ribosomal protein L22	Translation, ribosomal structure and biogenesis [J]	Ribosome	Ruminococcaceae	Family	Firmicutes	Clostridia	Clostridiales	Ruminococcaceae			-0.168175573	0.00359424	**
**413**	31551	MH0360_GL0125000	COG0480	Translation elongation factor EF-G, a GTPase	Translation, ribosomal structure and biogenesis [J]	NAN	Faecalibacterium prausnitzii	Species	Firmicutes	Clostridia	Clostridiales	Ruminococcaceae	Faecalibacterium	Faecalibacterium prausnitzii	-0.100483486	0.264675681	
**414**	31642	MH0363_GL0035255	COG1145	Ferredoxin	Energy production and conversion [C]	Glycolysis / Gluconeogenesis	Eubacterium	Genus	Firmicutes	Clostridia	Clostridiales	Eubacteriaceae	Eubacterium		0.006053535	0.937169777	
**415**	31655	MH0363_GL0154863	COG1653	ABC-type glycerol-3-phosphate transport system, periplasmic component	Carbohydrate transport and metabolism [G]	ABC transporters	Firmicutes	Phylum	Firmicutes						0.142100259	0.152982896	
**416**	31674	MH0364_GL0101039	COG0057	Glyceraldehyde-3-phosphate dehydrogenase/erythrose-4-phosphate dehydrogenase	Carbohydrate transport and metabolism [G]	Glycolysis / Gluconeogenesis	Ruminococcus bicirculans	Species	Firmicutes	Clostridia	Clostridiales	Ruminococcaceae	Ruminococcus	Ruminococcus bicirculans	-0.419762091	0.000366239	***

**417**	31742	MH0367_GL0050319	COG1592	Rubrerythrin	Energy production and conversion [C]	NAN	Firmicutes	Phylum	Firmicutes						-0.04890101	0.345559919	
**418**	31758	MH0367_GL0121504	COG0149	Triosephosphate isomerase	Carbohydrate transport and metabolism [G]	Glycolysis / Gluconeogenesis	Firmicutes	Phylum	Firmicutes						0.043438351	0.459497658	
**419**	31916	MH0370_GL0042621	COG4771	Outer membrane receptor for ferrienterochelin and colicins	Inorganic ion transport and metabolism [P]	NAN	Bacteroides uniformis	Species	Bacteroidetes	Bacteroidia	Bacteroidales	Bacteroidaceae	Bacteroides	Bacteroides uniformis	0.264938232	0.021840577	*
**420**	31959	MH0370_GL0059817	COG0832	Urease beta subunit	Amino acid transport and metabolism [E]	Purine metabolism	Bacteria	Superkingdom							0.016077889	0.737946274	
**421**	32014	MH0370_GL0093205	COG2222	Fructoselysine-6-P-deglycase FrlB and related proteins with duplicated sugar isomerase (SIS) domain	Cell wall/membrane/envelope biogenesis [M]	Alanine, aspartate and glutamate metabolism	Collinsella aerofaciens	Species	Actinobacteria	Coriobacteriia	Coriobacteriales	Coriobacteriaceae	Collinsella	Collinsella aerofaciens	0.245381319	0.001475739	**
**422**	32128	MH0371_GL0055922	COG1538	Outer membrane protein TolC	Cell wall/membrane/envelope biogenesis [M]	Bacterial secretion system	Bacteroides	Genus	Bacteroidetes	Bacteroidia	Bacteroidales	Bacteroidaceae	Bacteroides		0.262633841	0.00057883	***
**423**	32214	MH0372_GL0099061	COG0098	Ribosomal protein S5(	Translation, ribosomal structure and biogenesis [J]	Ribosome	Bifidobacterium	Genus	Actinobacteria	Actinobacteria	Bifidobacteriales	Bifidobacteriaceae	Bifidobacterium		-0.130667704	0.058644793	
**424**	32712	MH0383_GL0119529	COG0126	3-phosphoglycerate kinase	Carbohydrate transport and metabolism [G]	Glycolysis / Gluconeogenesis	Ruminococcus bromii	Species	Firmicutes	Clostridia	Clostridiales	Ruminococcaceae	Ruminococcus	Ruminococcus bromii	0.084358416	0.43772267	
**425**	32757	MH0385_GL0010518	COG0443	Molecular chaperone DnaK (HSP70)	Posttranslational modification, protein turnover, chaperones [O]	RNA degradation	Firmicutes	Phylum	Firmicutes						-0.168922552	0.001277391	**
**426**	33066	MH0391_GL0150753	COG0087	Ribosomal protein L3	Translation, ribosomal structure and biogenesis [J]	Ribosome	Clostridium	Genus	Firmicutes	Clostridia	Clostridiales	Clostridiaceae	Clostridium		-0.103802915	0.051957062	
**427**	33207	MH0396_GL0047332	COG3842	ABC-type Fe3+/spermidine/putrescine transport systems, ATPase components	Amino acid transport and metabolism [E]	ABC transporters	Clostridiales	Order	Firmicutes	Clostridia	Clostridiales				-0.14890362	0.143426417	
**428**	33402	MH0400_GL0071356			NAN	NAN	Ruminococcus bromii	Species	Firmicutes	Clostridia	Clostridiales	Ruminococcaceae	Ruminococcus	Ruminococcus bromii	0.005230167	0.963420724	
**429**	33560	O2.UC28-0_GL0204562	COG0254	Ribosomal protein L31	Translation, ribosomal structure and biogenesis [J]	Ribosome	Clostridiales	Order	Firmicutes	Clostridia	Clostridiales				-0.054559884	0.334022347	
**430**	33702	MH0406_GL0015896	COG1592	Rubrerythrin	Energy production and conversion [C]	NAN	Collinsella	Genus	Actinobacteria	Coriobacteriia	Coriobacteriales	Coriobacteriaceae	Collinsella		0.176580412	0.019125432	*
**431**	33861	MH0407_GL0191700	COG0246	Mannitol-1-phosphate/altronate dehydrogenases	Carbohydrate transport and metabolism [G]	Pentose and glucuronate interconversions	Clostridium	Genus	Firmicutes	Clostridia	Clostridiales	Clostridiaceae	Clostridium		-0.102439875	0.050341276	
**432**	34201	MH0415_GL0203137	COG0104	Adenylosuccinate synthase	Nucleotide transport and metabolism [F]	Purine metabolism	Ruminococcus bromii	Species	Firmicutes	Clostridia	Clostridiales	Ruminococcaceae	Ruminococcus	Ruminococcus bromii	-0.099704532	0.089560252	
**433**	34219	MH0416_GL0018356	COG5275	BRCT domain type II	General function prediction only [R]	Arginine and proline metabolism	Dorea longicatena	Species	Firmicutes	Clostridia	Clostridiales	Lachnospiraceae	Dorea	Dorea longicatena	0.095423708	0.191896058	
**434**	34380	MH0419_GL0081674	COG0228	Ribosomal protein S16	Translation, ribosomal structure and biogenesis [J]	Ribosome	Bifidobacterium	Genus	Actinobacteria	Actinobacteria	Bifidobacteriales	Bifidobacteriaceae	Bifidobacterium		-0.216944462	0.002747718	**
**435**	34449	MH0086_GL0032208	COG0539	Ribosomal protein S1	Translation, ribosomal structure and biogenesis [J]	Ribosome	Lachnospiraceae	Family	Firmicutes	Clostridia	Clostridiales	Lachnospiraceae			-0.08117472	0.07525135	
**436**	34610	MH0423_GL0103049	COG0088	Ribosomal protein L4	Translation, ribosomal structure and biogenesis [J]	Ribosome	Clostridiales	Order	Firmicutes	Clostridia	Clostridiales				-0.136561976	0.011107842	*
**437**	34973	MH0432_GL0050497	COG5263	Glucan-binding domain (YG repeat)	Carbohydrate transport and metabolism [G]	NAN	Clostridium	Genus	Firmicutes	Clostridia	Clostridiales	Clostridiaceae	Clostridium		0.354154247	0.006726435	**
**438**	35005	MH0432_GL0168684	COG0126	3-phosphoglycerate kinase	Carbohydrate transport and metabolism [G]	Glycolysis / Gluconeogenesis	Collinsella	Genus	Actinobacteria	Coriobacteriia	Coriobacteriales	Coriobacteriaceae	Collinsella		0.140947731	0.060582296	
**439**	35257	MH0435_GL0100291	COG0149	Triosephosphate isomerase	Carbohydrate transport and metabolism [G]	Glycolysis / Gluconeogenesis	Roseburia	Genus	Firmicutes	Clostridia	Clostridiales	Lachnospiraceae	Roseburia		-0.111142897	0.031194172	*
**440**	35519	MH0440_GL0182683	COG1454	Alcohol dehydrogenase, class IV	Energy production and conversion [C]	Glycolysis / Gluconeogenesis	Bacteria	Species	Actinobacteria	Coriobacteriia	Coriobacteriales	Coriobacteriaceae	Collinsella	Collinsella aerofaciens	0.136021083	0.184592251	
**441**	35568	MH0441_GL0130959	COG0334	Glutamate dehydrogenase/leucine dehydrogenase	Amino acid transport and metabolism [E]	Alanine, aspartate and glutamate metabolism	Clostridiales	Order	Firmicutes	Clostridia	Clostridiales				-0.071073519	0.182363328	
**442**	35789	MH0445_GL0066110	COG1866	Phosphoenolpyruvate carboxykinase, ATP-dependent	Energy production and conversion [C]	Glycolysis / Gluconeogenesis	Lachnospiraceae	Family	Firmicutes	Clostridia	Clostridiales	Lachnospiraceae			0.076912352	0.292885852	
**443**	35949	MH0447_GL0209994	COG0366	Glycosidase	Carbohydrate transport and metabolism [G]	Galactose metabolism	Ruminococcus bromii	Species	Firmicutes	Clostridia	Clostridiales	Ruminococcaceae	Ruminococcus	Ruminococcus bromii	0.119746552	0.107537763	
**444**	35954	MH0447_GL0228814	COG4799	Acetyl-CoA carboxylase, carboxyltransferase component	Lipid transport and metabolism [I]	Fatty acid biosynthesis	Clostridiales	Order	Firmicutes	Clostridia	Clostridiales				-0.012294545	0.881525482	
**445**	35996	MH0448_GL0031695	COG0822	NifU homolog involved in Fe-S cluster formation	Posttranslational modification, protein turnover, chaperones [O]	NAN	Faecalibacterium prausnitzii	Species	Firmicutes	Clostridia	Clostridiales	Ruminococcaceae	Faecalibacterium	Faecalibacterium prausnitzii	-0.106025292	0.307490442	
**446**	36042	MH0448_GL0176244	COG1196	Chromosome segregation ATPase	Cell cycle control, cell division, chromosome partitioning [D]	NAN	Bacteroides dorei	Species	Bacteroidetes	Bacteroidia	Bacteroidales	Bacteroidaceae	Bacteroides	Bacteroides dorei	0.066647515	0.620398168	
**447**	36197	MH0452_GL0206149	COG3203	Outer membrane protein (porin)	Cell wall/membrane/envelope biogenesis [M]	NAN	Dialister invisus	Species	Firmicutes	Negativicutes	Veillonellales	Veillonellaceae	Dialister	Dialister invisus	0.077323541	0.283684042	
**448**	36335	MH0455_GL0153195	COG0443	Molecular chaperone DnaK (HSP70)	Posttranslational modification, protein turnover, chaperones [O]	RNA degradation	Clostridiales	Order	Firmicutes	Clostridia	Clostridiales				0.044764869	0.460199554	
**449**	36549	N003A_GL0049315	COG1862	Preprotein translocase subunit YajC	Intracellular trafficking, secretion, and vesicular transport [U]	Protein export	Bacteroides	Genus	Bacteroidetes	Bacteroidia	Bacteroidales	Bacteroidaceae	Bacteroides		0.08872288	0.128798245	
**450**	36622	N013A_GL0032025	COG0059	Ketol-acid reductoisomerase	Coenzyme transport and metabolism [H]	Valine, leucine and isoleucine biosynthesis	Eubacterium	Genus	Firmicutes	Clostridia	Clostridiales	Eubacteriaceae	Eubacterium		0.000821899	0.992522532	
**451**	36712	N025A_GL0031266	COG1653	ABC-type glycerol-3-phosphate transport system, periplasmic component	Carbohydrate transport and metabolism [G]	ABC transporters	Faecalibacterium prausnitzii	Species	Firmicutes	Clostridia	Clostridiales	Ruminococcaceae	Faecalibacterium	Faecalibacterium prausnitzii	0.056872344	0.448458311	
**452**	36866	N038A_GL0001480	COG1653	ABC-type glycerol-3-phosphate transport system, periplasmic component	Carbohydrate transport and metabolism [G]	NAN	Bacteria	Superkingdom							-0.074071977	0.227676057	
**453**	36918	N042A_GL0025320	COG0012	Ribosome-binding ATPase YchF, GTP1/OBG family	Translation, ribosomal structure and biogenesis [J]	NAN	Clostridiales	Order	Firmicutes	Clostridia	Clostridiales				-0.095044063	0.026099336	*
**454**	36972	N047A_GL0051881			NAN	NAN	Bacteroides	Genus	Bacteroidetes	Bacteroidia	Bacteroidales	Bacteroidaceae	Bacteroides		0.119678303	0.295115281	
**455**	37011	N056A_GL0035081	COG0235	Ribulose-5-phosphate 4-epimerase/Fuculose-1-phosphate aldolase	Carbohydrate transport and metabolism [G]	Pentose and glucuronate interconversions	Clostridiales	Order	Firmicutes	Clostridia	Clostridiales				-0.150241011	0.02386674	*

**456**	37103	N079A_GL0025412	COG0049	Ribosomal protein S7	Translation, ribosomal structure and biogenesis [J]	Ribosome	Faecalibacterium prausnitzii	Species	Firmicutes	Clostridia	Clostridiales	Ruminococcaceae	Faecalibacterium	Faecalibacterium prausnitzii	-0.206696233	0.000399391	***
**457**	37117	N082A_GL0072780	COG0747	ABC-type transport system, periplasmic component	Amino acid transport and metabolism [E]	NAN	Lachnoclostridium phytofermentans	Species	Firmicutes	Clostridia	Clostridiales	Lachnospiraceae	Lachnoclostridium	Lachnoclostridium phytofermentans	-0.057090155	0.440771733	
**458**	37211	N086A_GL0096767	COG0092	Ribosomal protein S3	Translation, ribosomal structure and biogenesis [J]	Ribosome	Ruminococcaceae	Species	Firmicutes	Clostridia	Clostridiales	Ruminococcaceae	Subdoligranulum	Subdoligranulum variabile	-0.156102012	0.003888246	**
**459**	37290	N089A_GL0114097	COG1653	ABC-type glycerol-3-phosphate transport system, periplasmic component	Carbohydrate transport and metabolism [G]	ABC transporters	Faecalibacterium prausnitzii	Species	Firmicutes	Clostridia	Clostridiales	Ruminococcaceae	Faecalibacterium	Faecalibacterium prausnitzii	0.097272375	0.306055632	
**460**	37369	NLF005_GL0044735	COG2025	Electron transfer flavoprotein, alpha subunit	Energy production and conversion [C]	Nitrogen metabolism	Clostridiales	Order	Firmicutes	Clostridia	Clostridiales				-0.096618987	0.390972415	
**461**	37656	NLM017_GL0007726	COG0593	Chromosomal replication initiation ATPase DnaA	Replication, recombination and repair [L]	NAN	Clostridiales	Order	Firmicutes	Clostridia	Clostridiales				-0.043866813	0.447972711	
**462**	37699	NLM023_GL0007606	COG1879	ABC-type sugar transport system, periplasmic component, contains N-terminal xre family HTH domain	Carbohydrate transport and metabolism [G]	ABC transporters	Clostridium	Genus	Firmicutes	Clostridia	Clostridiales	Clostridiaceae	Clostridium		0.036955419	0.726213635	
**463**	37765	NLM027_GL0008865	COG1185	Polyribonucleotide nucleotidyltransferase (polynucleotide phosphorylase)	Translation, ribosomal structure and biogenesis [J]	Purine metabolism	Clostridiales	Order	Firmicutes	Clostridia	Clostridiales				0.005323664	0.892783292	
**464**	37777	NLM027_GL0043238	COG0013	Alanyl-tRNA synthetase	Translation, ribosomal structure and biogenesis [J]	Aminoacyl-tRNA biosynthesis	Clostridiales	Order	Firmicutes	Clostridia	Clostridiales				-0.085543676	0.084643899	
**465**	37821	NLM029_GL0062158	COG0152	Phosphoribosylaminoimidazole-succinocarboxamide synthase	Nucleotide transport and metabolism [F]	Purine metabolism	Ruminococcaceae	Species	Firmicutes	Clostridia	Clostridiales	Ruminococcaceae	Ruminococcus	Ruminococcus callidus	-0.115010995	0.028174863	*
**466**	37874	NLM032_GL0007335	COG0543	NAD(P)H-flavin reductase	Coenzyme transport and metabolism [H]	Amino sugar and nucleotide sugar metabolism	Clostridiales	Order	Firmicutes	Clostridia	Clostridiales				-0.050540297	0.378073194	
**467**	37877	NLM032_GL0020088			NAN	NAN	Paenibacillus sp. IHB B 3415	Species	Firmicutes	Bacilli	Bacillales	Paenibacillaceae	Paenibacillus	Paenibacillus sp. IHB B 3415	-0.13956227	0.034780007	*
**468**	37919	NOF005_GL0030287	COG0087	Ribosomal protein L3	Translation, ribosomal structure and biogenesis [J]	Ribosome	Clostridiales	Order	Firmicutes	Clostridia	Clostridiales				-0.107105629	0.131894263	
**469**	38121	NOM009_GL0086915	COG1250	3-hydroxyacyl-CoA dehydrogenase	Lipid transport and metabolism [I]	Fatty acid degradation	Clostridiales	Order	Firmicutes	Clostridia	Clostridiales				-0.087225976	0.396163613	
**470**	38126	NOM009_GL0108718	COG1653	ABC-type glycerol-3-phosphate transport system, periplasmic component	Carbohydrate transport and metabolism [G]	ABC transporters	Collinsella aerofaciens	Species	Actinobacteria	Coriobacteriia	Coriobacteriales	Coriobacteriaceae	Collinsella	Collinsella aerofaciens	0.165339661	0.063179047	
**471**	38160	NOM014_GL0023636	COG0334	Glutamate dehydrogenase/leucine dehydrogenase	Amino acid transport and metabolism [E]	Alanine, aspartate and glutamate metabolism	Clostridium	Genus	Firmicutes	Clostridia	Clostridiales	Clostridiaceae	Clostridium		0.130022598	0.15633944	
**472**	38293	NOM028_GL0008171	COG5016	Pyruvate/oxaloacetate carboxyltransferase	Energy production and conversion [C]	Citrate cycle (TCA cycle)	Clostridiales	Order	Firmicutes	Clostridia	Clostridiales				-0.111823449	0.05938761	
**473**	38315	NOM029_GL0053956	COG0098	Ribosomal protein S5	Translation, ribosomal structure and biogenesis [J]	Ribosome	Clostridiales	Order	Firmicutes	Clostridia	Clostridiales				-0.155426236	0.032388278	*
**474**	38448	O2.CD1-0-PT_GL0064002	COG1151	Hydroxylamine reductase (hybrid-cluster protein)	Inorganic ion transport and metabolism [P]	Nitrotoluene degradation	Clostridiales	Order	Firmicutes	Clostridia	Clostridiales				-0.081866941	0.116831539	
**475**	38463	O2.CD1-0-PT_GL0080248	COG0138	AICAR transformylase/IMP cyclohydrolase PurH	Nucleotide transport and metabolism [F]	Purine metabolism	Bifidobacterium	Genus	Actinobacteria	Actinobacteria	Bifidobacteriales	Bifidobacteriaceae	Bifidobacterium		-0.003481486	0.955410023	
**476**	38482	O2.CD1-0-PT_GL0091861	COG0021	Transketolase	Carbohydrate transport and metabolism [G]	Pentose phosphate pathway	Bifidobacterium	Genus	Actinobacteria	Actinobacteria	Bifidobacteriales	Bifidobacteriaceae	Bifidobacterium		0.136073043	0.044021613	*
**477**	38558	O2.CD2-0_GL0042351	COG1978	Predicted RNase H-related nuclease YkuK, DUF458 family	General function prediction only [R]	NAN	Dorea formicigenerans	Species	Firmicutes	Clostridia	Clostridiales	Lachnospiraceae	Dorea	Dorea formicigenerans	0.045208501	0.486636114	
**478**	38738	O2.CD2-0-PT_GL0075729	COG1653	ABC-type glycerol-3-phosphate transport system, periplasmic component	Carbohydrate transport and metabolism [G]	ABC transporters	Bifidobacterium	Genus	Actinobacteria	Actinobacteria	Bifidobacteriales	Bifidobacteriaceae	Bifidobacterium		0.113551196	0.281187587	
**479**	38881	O2.CD3-0-PT_GL0099997	COG3411	(2Fe-2S) ferredoxin	Energy production and conversion [C]	Oxidative phosphorylation	Clostridiales	Order	Firmicutes	Clostridia	Clostridiales				-0.083095413	0.189513097	
**480**	39077	O2.UC11-1_GL0020482	COG2222	Fructoselysine-6-P-deglycase FrlB and related proteins with duplicated sugar isomerase (SIS) domain	Cell wall/membrane/envelope biogenesis [M]	Alanine, aspartate and glutamate metabolism	Collinsella	Genus	Actinobacteria	Coriobacteriia	Coriobacteriales	Coriobacteriaceae	Collinsella		0.107161868	0.140228598	
**481**	39110	O2.UC11-1_GL0068784	COG1978	Predicted RNase H-related nuclease YkuK, DUF458 family	General function prediction only [R]	NAN	Dorea formicigenerans	Species	Firmicutes	Clostridia	Clostridiales	Lachnospiraceae	Dorea	Dorea formicigenerans	0.209948908	0.006294553	**
**482**	39123	O2.UC11-1_GL0109553			NAN	NAN	Dorea formicigenerans	Species	Firmicutes	Clostridia	Clostridiales	Lachnospiraceae	Dorea	Dorea formicigenerans	0.047901464	0.478650769	
**483**	39184	O2.UC11-2_GL0041094	COG3842	ABC-type Fe3+/spermidine/putrescine transport systems, ATPase components	Amino acid transport and metabolism [E]	ABC transporters	Firmicutes	Phylum	Firmicutes						0.056118272	0.345386435	
**484**	39301	O2.UC12-1_GL0041048	COG0191	Fructose/tagatose bisphosphate aldolase	Carbohydrate transport and metabolism [G]	Glycolysis / Gluconeogenesis	Collinsella	Genus	Actinobacteria	Coriobacteriia	Coriobacteriales	Coriobacteriaceae	Collinsella		0.116057368	0.115146672	
**485**	39540	O2.UC14-2_GL0091805	COG1653	ABC-type glycerol-3-phosphate transport system, periplasmic component	Carbohydrate transport and metabolism [G]	ABC transporters	Ruminococcus	Genus	Firmicutes	Clostridia	Clostridiales	Ruminococcaceae	Ruminococcus		-0.019308827	0.837004596	
**486**	39724	O2.UC15-1_GL0039389	COG0280	Phosphotransacetylase	Energy production and conversion [C]	Taurine and hypotaurine metabolism	Clostridiales	Order	Firmicutes	Clostridia	Clostridiales				-0.14151548	0.054213562	
**487**	39746	O2.UC15-1_GL0073124	COG0149	Triosephosphate isomerase	Carbohydrate transport and metabolism [G]	Glycolysis / Gluconeogenesis	Blautia	Genus	Firmicutes	Clostridia	Clostridiales	Lachnospiraceae	Blautia		-0.100538166	0.042252019	*
**488**	39751	O2.UC15-1_GL0080989	COG1063	Threonine dehydrogenase or related Zn-dependent dehydrogenase	General function prediction only [R]	Fructose and mannose metabolism	Clostridiales	Order	Firmicutes	Clostridia	Clostridiales				-0.015885836	0.833315764	
**489**	39842	O2.UC16-1_GL0052335	COG1879	ABC-type sugar transport system, periplasmic component, contains N-terminal xre family HTH domain	Carbohydrate transport and metabolism [G]	ABC transporters	Clostridiales	Order	Firmicutes	Clostridia	Clostridiales				-0.043944141	0.58614001	
**490**	39882	O2.UC16-2_GL0091224			NAN	NAN	Firmicutes	Phylum	Firmicutes						-0.060807905	0.473458159	
**491**	39918	O2.UC17-1_GL0077925	COG0539	Ribosomal protein S1	Translation, ribosomal structure and biogenesis [J]	Ribosome	Collinsella	Genus	Actinobacteria	Coriobacteriia	Coriobacteriales	Coriobacteriaceae	Collinsella		0.032205994	0.613630067	
**492**	39921	O2.UC17-1_GL0084053	COG0078	Ornithine carbamoyltransferase	Amino acid transport and metabolism [E]	Arginine and proline metabolism	Clostridiales	Order	Firmicutes	Clostridia	Clostridiales				0.077348687	0.164023142	

**493**	39980	O2.UC17-2_GL0115696	COG1145	Ferredoxin	Energy production and conversion [C]	Alanine, aspartate and glutamate metabolism	Blautia	Genus	Firmicutes	Clostridia	Clostridiales	Lachnospiraceae	Blautia		-0.024434341	0.735892358	
**494**	40091	O2.UC18-1_GL0200164	COG1063	Threonine dehydrogenase or related Zn-dependent dehydrogenase	General function prediction only [R]	Fructose and mannose metabolism	Blautia obeum	Species	Firmicutes	Clostridia	Clostridiales	Lachnospiraceae	Blautia	Blautia obeum	-0.143411249	0.014961438	*
**495**	40101	O2.UC18-2_GL0031006	COG0517	CBS domain	Signal transduction mechanisms [T]	Purine metabolism	Ruminococcaceae	Species	Firmicutes	Clostridia	Clostridiales	Ruminococcaceae	Faecalibacterium	Faecalibacterium prausnitzii	-0.135518727	0.016942219	*
**496**	40126	O2.UC19-1_GL0002247	COG4154	L-fucose mutarotase/ribose pyranase, RbsD/FucU family	Carbohydrate transport and metabolism [G]	NAN	Clostridiales	Order	Firmicutes	Clostridia	Clostridiales				0.00570513	0.896994904	
**497**	40312	O2.UC2-0_GL0002702	COG0052	Ribosomal protein S2	Translation, ribosomal structure and biogenesis [J]	Ribosome	Ruminococcaceae	Family	Firmicutes	Clostridia	Clostridiales	Ruminococcaceae			-0.112814652	0.120849081	
**498**	40455	O2.UC20-2_GL0062335	COG3203	Outer membrane protein (porin)	Cell wall/membrane/envelope biogenesis [M]	NAN	Dialister invisus	Species	Firmicutes	Negativicutes	Veillonellales	Veillonellaceae	Dialister	Dialister invisus	0.099248919	0.238377676	
**499**	40481	O2.UC2-1_GL0059641	COG0206	Cell division GTPase FtsZ	Cell cycle control, cell division, chromosome partitioning [D]	Cell cycle - Caulobacter	Clostridiales	Order	Firmicutes	Clostridia	Clostridiales				-0.179992723	0.008051822	**
**500**	40575	O2.UC21-2_GL0076310	COG3958	Transketolase, C-terminal subunit	Carbohydrate transport and metabolism [G]	Pentose phosphate pathway	Clostridiales	Order	Firmicutes	Clostridia	Clostridiales				-0.010483372	0.864976803	
**501**	40650	O2.UC22-1_GL0018255	COG0539	Ribosomal protein S1	Translation, ribosomal structure and biogenesis [J]	Ribosome	Blautia	Genus	Firmicutes	Clostridia	Clostridiales	Lachnospiraceae	Blautia		-0.074450138	0.248143758	
**502**	40802	O2.UC23-1_GL0058516	COG0149	Triosephosphate isomerase	Carbohydrate transport and metabolism [G]	Glycolysis / Gluconeogenesis	Clostridiales	Order	Firmicutes	Clostridia	Clostridiales				-0.069501108	0.278994801	
**503**	40893	O2.UC24-1_GL0171065			NAN	NAN	Ruminococcus bromii	Species	Firmicutes	Clostridia	Clostridiales	Ruminococcaceae	Ruminococcus	Ruminococcus bromii	-0.112094167	0.130162882	
**504**	40993	O2.UC26-1_GL0038440	COG3209	Uncharacterized conserved protein RhaS, contains 28 RHS repeats	General function prediction only [R]	NAN	Blautia obeum	Species	Firmicutes	Clostridia	Clostridiales	Lachnospiraceae	Blautia	Blautia obeum	0.138575662	0.213617275	
**505**	41146	O2.UC28-2_GL0061806	COG0234	Co-chaperonin GroES (HSP10)	Posttranslational modification, protein turnover, chaperones [O]	NAN	Collinsella	Genus	Actinobacteria	Coriobacteriia	Coriobacteriales	Coriobacteriaceae	Collinsella		0.015196631	0.846493499	
**506**	41174	O2.UC28-2_GL0191778	COG1653	ABC-type glycerol-3-phosphate transport system, periplasmic component	Carbohydrate transport and metabolism [G]	ABC transporters	Clostridiales	Order	Firmicutes	Clostridia	Clostridiales				-0.081689999	0.336948314	
**507**	41223	O2.UC29-2_GL0118672	COG0593	Chromosomal replication initiation ATPase DnaA	Replication, recombination and repair [L]	NAN	Subdoligranulum variabile	Species	Firmicutes	Clostridia	Clostridiales	Ruminococcaceae	Subdoligranulum	Subdoligranulum variabile	-0.137395998	0.039763243	*
**508**	41264	O2.UC30-0_GL0087476	COG0544	FKBP-type peptidyl-prolyl cis-trans isomerase (trigger factor)	Posttranslational modification, protein turnover, chaperones [O]	NAN	Clostridiales	Order	Firmicutes	Clostridia	Clostridiales				-0.020932592	0.746519803	
**509**	41277	O2.UC30-1_GL0014932	COG3842	ABC-type Fe3+/spermidine/putrescine transport systems, ATPase components	Amino acid transport and metabolism [E]	ABC transporters	Clostridium	Genus	Firmicutes	Clostridia	Clostridiales	Clostridiaceae	Clostridium		-0.059705633	0.292836464	
**510**	41419	O2.UC31-1_GL0115264	COG0085	DNA-directed RNA polymerase, beta subunit/140 kD subunit	Transcription [K]	Purine metabolism	Clostridiales	Order	Firmicutes	Clostridia	Clostridiales				-0.265025627	0.000587996	***
**511**	41485	O2.UC32-0_GL0051137	COG2160	L-arabinose isomerase	Carbohydrate transport and metabolism [G]	Pentose and glucuronate interconversions	Bifidobacterium	Genus	Actinobacteria	Actinobacteria	Bifidobacteriales	Bifidobacteriaceae	Bifidobacterium		-0.062912616	0.587762403	
**512**	41502	O2.UC32-1_GL0001634	COG1102	Cytidylate kinase	Nucleotide transport and metabolism [F]	NAN	Lachnospiraceae	Family	Firmicutes	Clostridia	Clostridiales	Lachnospiraceae			0.025863934	0.634823024	
**513**	41546	O2.UC32-1_GL0088850	COG0459	Chaperonin GroEL (HSP60 family)	Posttranslational modification, protein turnover, chaperones [O]	RNA degradation	Clostridiales	Order	Firmicutes	Clostridia	Clostridiales				-0.006388154	0.910253273	
**514**	41682	O2.UC35-1_GL0126652	COG0088	Ribosomal protein L4	Translation, ribosomal structure and biogenesis [J]	Ribosome	Faecalibacterium prausnitzii	Species	Firmicutes	Clostridia	Clostridiales	Ruminococcaceae	Faecalibacterium	Faecalibacterium prausnitzii	-0.04362253	0.562042586	
**515**	41706	O2.UC35-2_GL0138962	COG0366	Glycosidase	Carbohydrate transport and metabolism [G]	Galactose metabolism	Clostridiales	Order	Firmicutes	Clostridia	Clostridiales				0.011196258	0.865182969	
**516**	41889	O2.UC37-1_GL0081003	COG0021	Transketolase	Carbohydrate transport and metabolism [G]	Pentose phosphate pathway	Bifidobacterium bifidum	Species	Actinobacteria	Actinobacteria	Bifidobacteriales	Bifidobacteriaceae	Bifidobacterium	Bifidobacterium bifidum	0.011896329	0.914157429	
**517**	41900	O2.UC37-1_GL0100475	COG0282	Acetate kinase	Energy production and conversion [C]	Taurine and hypotaurine metabolism	Faecalibacterium prausnitzii	Species	Firmicutes	Clostridia	Clostridiales	Ruminococcaceae	Faecalibacterium	Faecalibacterium prausnitzii	-0.07237925	0.293656068	
**518**	42060	O2.UC40-1_GL0122610	COG1882	Pyruvate-formate lyase	Energy production and conversion [C]	Pyruvate metabolism	Clostridiales bacterium KLE1615	Species	Firmicutes	Clostridia	Clostridiales			Clostridiales bacterium KLE1615	-0.305746247	0.000751388	***
**519**	42140	O2.UC4-1_GL0039891	COG3835	Sugar diacid utilization regulator	Signal transduction mechanisms [T]	NAN	Ruminococcus bromii	Species	Firmicutes	Clostridia	Clostridiales	Ruminococcaceae	Ruminococcus	Ruminococcus bromii	0.067970964	0.313524357	
**520**	42159	O2.UC4-1_GL0166577	COG0334	Glutamate dehydrogenase/leucine dehydrogenase	Amino acid transport and metabolism [E]	Alanine, aspartate and glutamate metabolism	Clostridiales	Order	Firmicutes	Clostridia	Clostridiales				0.047643627	0.545779754	
**521**	42296	O2.UC4-2_GL0000649	COG0234	Co-chaperonin GroES (HSP10)	Posttranslational modification, protein turnover, chaperones [O]	NAN	Clostridiales	Order	Firmicutes	Clostridia	Clostridiales				-0.012020191	0.835898913	
**522**	42469	O2.UC44-1_GL0166896	COG3957	Phosphoketolase	Carbohydrate transport and metabolism [G]	Pentose phosphate pathway	Bifidobacterium	Genus	Actinobacteria	Actinobacteria	Bifidobacteriales	Bifidobacteriaceae	Bifidobacterium		-0.038386056	0.748008346	
**523**	42472	O2.UC44-1_GL0171886	COG0049	Ribosomal protein S7	Translation, ribosomal structure and biogenesis [J]	Ribosome	Subdoligranulum variabile	Species	Firmicutes	Clostridia	Clostridiales	Ruminococcaceae	Subdoligranulum	Subdoligranulum variabile	-0.039739148	0.364470991	
**524**	42601	O2.UC46-1_GL0109045	COG1145	Ferredoxin	Energy production and conversion [C]	Glycolysis / Gluconeogenesis	Faecalibacterium prausnitzii	Species	Firmicutes	Clostridia	Clostridiales	Ruminococcaceae	Faecalibacterium	Faecalibacterium prausnitzii	-0.210443462	0.012427576	*
**525**	42632	O2.UC46-2_GL0146529	COG1879	ABC-type sugar transport system, periplasmic component, contains N-terminal xre family HTH domain	Carbohydrate transport and metabolism [G]	ABC transporters	Clostridiales	Order	Firmicutes	Clostridia	Clostridiales				-0.063052024	0.287171389	
**526**	42701	O2.UC47-2_GL0002078	COG1250	3-hydroxyacyl-CoA dehydrogenase	Lipid transport and metabolism [I]	Fatty acid degradation	Clostridiales	Order	Firmicutes	Clostridia	Clostridiales				0.138266461	0.074917238	
**527**	42713	O2.UC47-2_GL0030333	COG1082	Sugar phosphate isomerase/epimerase	Carbohydrate transport and metabolism [G]	Inositol phosphate metabolism	Firmicutes	Phylum	Firmicutes						0.231444421	0.009260481	**
**528**	42738	O2.UC48-0_GL0000051	COG1653	ABC-type glycerol-3-phosphate transport system, periplasmic component	Carbohydrate transport and metabolism [G]	ABC transporters	Ruminococcus bromii	Species	Firmicutes	Clostridia	Clostridiales	Ruminococcaceae	Ruminococcus	Ruminococcus bromii	0.010757604	0.93963989	
**529**	42754	O2.UC48-0_GL0026589	COG1960	Acyl-CoA dehydrogenase related to the alkylation response protein AidB	Lipid transport and metabolism [I]	Fatty acid degradation	Firmicutes	Phylum	Firmicutes						0.105705299	0.115301303	

**530**	42755	O2.UC48-0_GL0026590	COG1024	Enoyl-CoA hydratase/carnithine racemase(	Lipid transport and metabolism [I]	Fatty acid degradation	Firmicutes	Phylum	Firmicutes						0.087769039	0.178780133	
**531**	42801	O2.UC48-0_GL0101313	COG4822	Cobalamin biosynthesis protein CbiK, Co2+ chelatase	Coenzyme transport and metabolism [H]	Porphyrin and chlorophyll metabolism	Clostridiales	Order	Firmicutes	Clostridia	Clostridiales				0.015353411	0.774357051	
**532**	42802	O2.UC48-0_GL0102815	COG1960	Acyl-CoA dehydrogenase related to the alkylation response protein AidB	Lipid transport and metabolism [I]	Fatty acid degradation	Firmicutes	Phylum	Firmicutes						0.136558411	0.079319718	
**533**	42803	O2.UC48-0_GL0103416	COG4992	Acetylornithine/succinyldiaminopimelate/putrescine aminotransferase	Amino acid transport and metabolism [E]	Lysine biosynthesis	Firmicutes	Phylum	Firmicutes						0.094152633	0.128039134	
**534**	42804	O2.UC48-0_GL0107610	COG3842	ABC-type Fe3+/spermidine/putrescine transport systems, ATPase components	Amino acid transport and metabolism [E]	ABC transporters	Lachnospiraceae	Family	Firmicutes	Clostridia	Clostridiales	Lachnospiraceae			-0.212894975	0.025839277	*
**535**	42831	O2.UC48-0_GL0126253	COG1454	Alcohol dehydrogenase, class IV	Energy production and conversion [C]	Glycolysis / Gluconeogenesis	Firmicutes	Phylum	Firmicutes						0.093927758	0.090534896	
**536**	42835	O2.UC48-0_GL0135226	COG1834	N-Dimethylarginine dimethylaminohydrolase	Amino acid transport and metabolism [E]	Glycine, serine and threonine metabolism	Bacteria	Superkingdom							0.493169082	2.50117E-05	***
**537**	42974	O2.UC48-1_GL0053868	COG0059	Ketol-acid reductoisomerase	Coenzyme transport and metabolism [H]	Valine, leucine and isoleucine biosynthesis	Clostridiales	Order	Firmicutes	Clostridia	Clostridiales				0.220617218	0.000369131	***
**538**	42995	O2.UC48-1_GL0148775	COG0334	Glutamate dehydrogenase/leucine dehydrogenase	Amino acid transport and metabolism [E]	Alanine, aspartate and glutamate metabolism	Ruminococcus bromii	Species	Firmicutes	Clostridia	Clostridiales	Ruminococcaceae	Ruminococcus	Ruminococcus bromii	0.067067868	0.441562246	
**539**	43085	O2.UC5-0_GL0002879	COG2407	L-fucose isomerase or related protein	Carbohydrate transport and metabolism [G]	Fructose and mannose metabolism	Clostridiales	Order	Firmicutes	Clostridia	Clostridiales				-0.063542618	0.491049705	
**540**	43145	O2.UC50-0_GL0264730	COG0091	Ribosomal protein L22	Translation, ribosomal structure and biogenesis [J]	Ribosome	Subdoligranulum variabile	Species	Firmicutes	Clostridia	Clostridiales	Ruminococcaceae	Subdoligranulum	Subdoligranulum variabile	0.123255159	0.021444501	*
**541**	43200	O2.UC5-1_GL0032422	COG0737	2',3'-cyclic-nucleotide 2'-phosphodiesterase/5'- or 3'-nucleotidase, 5'-nucleotidase family	Defense mechanisms [V]	Purine metabolism	Faecalibacterium prausnitzii	Species	Firmicutes	Clostridia	Clostridiales	Ruminococcaceae	Faecalibacterium	Faecalibacterium prausnitzii	-0.020031229	0.768719255	
**542**	43365	O2.UC55-0_GL0124819	COG0811	Biopolymer transport protein ExbB/TolQ	Intracellular trafficking, secretion, and vesicular transport [U]	Microbial metabolism in diverse environments	Parabacteroides	Genus	Bacteroidetes	Bacteroidia	Bacteroidales	Porphyromonadaceae	Parabacteroides		0.109519962	0.176597746	
**543**	43393	O2.UC56-0_GL0134141	COG0366	Glycosidase	Carbohydrate transport and metabolism [G]	Galactose metabolism	Clostridiales	Order	Firmicutes	Clostridia	Clostridiales				-0.050814949	0.236758851	
**544**	43394	O2.UC56-0_GL0138638	COG1087	UDP-glucose 4-epimerase	Cell wall/membrane/envelope biogenesis [M]	Galactose metabolism	Clostridiales	Order	Firmicutes	Clostridia	Clostridiales				-0.060121866	0.160384881	
**545**	43456	O2.UC57-2_GL0049799	COG0126	3-phosphoglycerate kinase	Carbohydrate transport and metabolism [G]	Glycolysis / Gluconeogenesis	Lachnospiraceae	Family	Firmicutes	Clostridia	Clostridiales	Lachnospiraceae			-0.049206261	0.598166577	
**546**	43480	O2.UC58-0_GL0094376	COG1762	Phosphotransferase system mannitol/fructose-specific IIA domain (Ntr-type)	Signal transduction mechanisms [T]	Fructose and mannose metabolism	Ruminococcus bromii	Species	Firmicutes	Clostridia	Clostridiales	Ruminococcaceae	Ruminococcus	Ruminococcus bromii	-0.149359031	0.115483336	
**547**	43491	O2.UC58-2_GL0017801	COG0360	Ribosomal protein S6	Translation, ribosomal structure and biogenesis [J]	Ribosome	Bifidobacterium	Genus	Actinobacteria	Actinobacteria	Bifidobacteriales	Bifidobacteriaceae	Bifidobacterium		0.129517151	0.13796905	
**548**	43499	O2.UC58-2_GL0079351	COG1454	Alcohol dehydrogenase, class IV	Energy production and conversion [C]	Glycolysis / Gluconeogenesis	Roseburia faecis	Species	Firmicutes	Clostridia	Clostridiales	Lachnospiraceae	Roseburia	Roseburia faecis	-0.108514126	0.165874732	
**549**	43649	T2D-53A_GL0140451	COG0091	Ribosomal protein L22	Translation, ribosomal structure and biogenesis [J]	Ribosome	Clostridiales	Order	Firmicutes	Clostridia	Clostridiales				-0.026693206	0.670176666	
**550**	43725	O2.UC7-1_GL0015069	COG1653	ABC-type glycerol-3-phosphate transport system, periplasmic component	Carbohydrate transport and metabolism [G]	ABC transporters	Clostridiales	Order	Firmicutes	Clostridia	Clostridiales				0.189261352	0.026261601	*
**551**	43732	O2.UC7-1_GL0034210	COG0047	Phosphoribosylformylglycinamidine (FGAM) synthase, glutamine amidotransferase domain	Nucleotide transport and metabolism [F]	Purine metabolism	Clostridiales	Order	Firmicutes	Clostridia	Clostridiales				-0.080247481	0.434797936	
**552**	43832	O2.UC8-0_GL0153477	COG0057	Glyceraldehyde-3-phosphate dehydrogenase/erythrose-4-phosphate dehydrogenase	Carbohydrate transport and metabolism [G]	Glycolysis / Gluconeogenesis	Bifidobacterium adolescentis	Species	Actinobacteria	Actinobacteria	Bifidobacteriales	Bifidobacteriaceae	Bifidobacterium	Bifidobacterium adolescentis	-0.144036598	0.101826719	
**553**	43926	O2.UC9-0_GL0086178	COG1592	Rubrerythrin	Energy production and conversion [C]	NAN	Lachnospiraceae	Family	Firmicutes	Clostridia	Clostridiales	Lachnospiraceae			-0.014951269	0.798557576	
**554**	44201	SZEY-09A_GL0049065	COG0737	2',3'-cyclic-nucleotide 2'-phosphodiesterase/5'- or 3'-nucleotidase, 5'-nucleotidase family	Defense mechanisms [V]	Purine metabolism	Faecalibacterium prausnitzii	Species	Firmicutes	Clostridia	Clostridiales	Ruminococcaceae	Faecalibacterium	Faecalibacterium prausnitzii	-0.006439585	0.950030747	
**555**	44405	SZEY-35A_GL0096220	COG0334	Glutamate dehydrogenase/leucine dehydrogenase	Amino acid transport and metabolism [E]	Alanine, aspartate and glutamate metabolism	Fusicatenibacter saccharivorans	Species	Firmicutes	Clostridia	Clostridiales	Lachnospiraceae	Fusicatenibacter	Fusicatenibacter saccharivorans	0.080692861	0.322472641	
**556**	44443	SZEY-38A_GL0037167	COG0137	Argininosuccinate synthase	Amino acid transport and metabolism [E]	Alanine, aspartate and glutamate metabolism	Ruminococcus bromii	Species	Firmicutes	Clostridia	Clostridiales	Ruminococcaceae	Ruminococcus	Ruminococcus bromii	-0.093458382	0.332378581	
**557**	44507	NLF006_GL0012500	COG1454	Alcohol dehydrogenase, class IV	Energy production and conversion [C]	NAN	Ruminococcus bromii	Species	Firmicutes	Clostridia	Clostridiales	Ruminococcaceae	Ruminococcus	Ruminococcus bromii	0.039633707	0.647513313	
**558**	44567	SZEY-59A_GL0043576	COG1653	ABC-type glycerol-3-phosphate transport system, periplasmic component	Carbohydrate transport and metabolism [G]	NAN	Ruminococcus bicirculans	Species	Firmicutes	Clostridia	Clostridiales	Ruminococcaceae	Ruminococcus	Ruminococcus bicirculans	0.050345865	0.677161009	
**559**	44603	SZEY-62A_GL0070012	COG0136	Aspartate-semialdehyde dehydrogenase	Amino acid transport and metabolism [E]	Glycine, serine and threonine metabolism	Ruminococcus bromii	Species	Firmicutes	Clostridia	Clostridiales	Ruminococcaceae	Ruminococcus	Ruminococcus bromii	0.175863915	0.037746782	*
**560**	44660	SZEY-69A_GL0070022	COG5492	Uncharacterized conserved protein YjdB, contains Ig-like domain	General function prediction only [R]	NAN	Symbiobacterium thermophilum	Species	Firmicutes	Clostridia	Clostridiales	Symbiobacteriaceae	Symbiobacterium	Symbiobacterium thermophilum	-0.270000312	0.00835433	**
**561**	44662	SZEY-69A_GL0078143	COG2222	Fructoselysine-6-P-deglycase FrlB and related proteins with duplicated sugar isomerase (SIS) domain	Cell wall/membrane/envelope biogenesis [M]	Alanine, aspartate and glutamate metabolism	Clostridiales	Order	Firmicutes	Clostridia	Clostridiales				0.002116379	0.974677778	
**562**	44707	SZEY-78A_GL0090404	COG0563	Adenylate kinase or related kinase	Nucleotide transport and metabolism [F]	Purine metabolism	Clostridiales	Order	Firmicutes	Clostridia	Clostridiales				-0.011300718	0.81881552	
**563**	44853	T2D-105A_GL0107136	COG1629	Outer membrane receptor proteins, mostly Fe transport	Inorganic ion transport and metabolism [P]	NAN	Bacteroides	Genus	Bacteroidetes	Bacteroidia	Bacteroidales	Bacteroidaceae	Bacteroides		0.21160442	0.007957095	**

**564**	45072	T2D-114A_GL0097515	COG0760	Parvulin-like peptidyl-prolyl isomerase	Posttranslational modification, protein turnover, chaperones [O]	NAN	Ruminococcus bromii	Species	Firmicutes	Clostridia	Clostridiales	Ruminococcaceae	Ruminococcus	Ruminococcus bromii	0.049881275	0.515954128	
**565**	45165	T2D-133A_GL0021831	COG1653	ABC-type glycerol-3-phosphate transport system, periplasmic component	Carbohydrate transport and metabolism [G]	NAN	Bifidobacterium	Genus	Actinobacteria	Actinobacteria	Bifidobacteriales	Bifidobacteriaceae	Bifidobacterium		-0.247393184	0.012884684	*
**566**	45166	T2D-122A_GL0083846	COG0176	Transaldolase	Carbohydrate transport and metabolism [G]	Pentose phosphate pathway	Bifidobacterium bifidum	Species	Actinobacteria	Actinobacteria	Bifidobacteriales	Bifidobacteriaceae	Bifidobacterium	Bifidobacterium bifidum	-0.042375337	0.7139688	
**567**	45245	T2D-133A_GL0070710	COG4771	Outer membrane receptor for ferrienterochelin and colicins	Inorganic ion transport and metabolism [P]	NAN	Bacteroides uniformis	Species	Bacteroidetes	Bacteroidia	Bacteroidales	Bacteroidaceae	Bacteroides	Bacteroides uniformis	0.189028803	0.099270601	
**568**	45270	T2D-135A_GL0000682	COG4771	Outer membrane receptor for ferrienterochelin and colicins	Inorganic ion transport and metabolism [P]	NAN	Bacteroides massiliensis	Species	Bacteroidetes	Bacteroidia	Bacteroidales	Bacteroidaceae	Bacteroides	Bacteroides massiliensis	0.293483408	0.004179742	**
**569**	45429	T2D-142A_GL0060662	COG1185	Polyribonucleotide nucleotidyltransferase (polynucleotide phosphorylase)	Translation, ribosomal structure and biogenesis [J]	Purine metabolism	Ruminococcus bromii	Species	Firmicutes	Clostridia	Clostridiales	Ruminococcaceae	Ruminococcus	Ruminococcus bromii	-0.070882508	0.394893392	
**570**	45433	T2D-142A_GL0079303	COG0457	Tetratricopeptide (TPR) repeat	General function prediction only [R]	NAN	Bacteroides eggerthii	Species	Bacteroidetes	Bacteroidia	Bacteroidales	Bacteroidaceae	Bacteroides	Bacteroides eggerthii	0.122181552	0.255757946	
**571**	45788	T2D-203A_GL0049645	COG0459	Chaperonin GroEL (HSP60 family)	Posttranslational modification, protein turnover, chaperones [O]	RNA degradation	Lachnospiraceae	Family	Firmicutes	Clostridia	Clostridiales	Lachnospiraceae			-0.007517061	0.942826248	
**572**	45892	T2D-26A_GL0024418	COG0078	Ornithine carbamoyltransferase	Amino acid transport and metabolism [E]	Arginine and proline metabolism	Collinsella aerofaciens	Species	Actinobacteria	Coriobacteriia	Coriobacteriales	Coriobacteriaceae	Collinsella	Collinsella aerofaciens	0.060646995	0.336908441	
**573**	45940	T2D-29A_GL0106551	COG1960	Acyl-CoA dehydrogenase related to the alkylation response protein AidB	Lipid transport and metabolism [I]	Fatty acid degradation	Clostridiales	Order	Firmicutes	Clostridia	Clostridiales				0.019735494	0.80665693	
**574**	46091	T2D-35A_GL0059333	COG0783	DNA-binding ferritin-like protein (oxidative damage protectant)	Defense mechanisms [V]	NAN	Bifidobacterium breve	Species	Actinobacteria	Actinobacteria	Bifidobacteriales	Bifidobacteriaceae	Bifidobacterium	Bifidobacterium breve	0.1874186	0.022851644	*
**575**	46103	T2D-41A_GL0070446	COG1264	Phosphotransferase system IIB components	Carbohydrate transport and metabolism [G]	Glycolysis / Gluconeogenesis	[Eubacterium] rectale	Species	Firmicutes	Clostridia	Clostridiales	Lachnospiraceae		[Eubacterium] rectale	-0.00584799	0.934448311	
**576**	46132	T2D-45A_GL0043917	COG0149	Triosephosphate isomerase	Carbohydrate transport and metabolism [G]	Glycolysis / Gluconeogenesis	Subdoligranulum variabile	Species	Firmicutes	Clostridia	Clostridiales	Ruminococcaceae	Subdoligranulum	Subdoligranulum variabile	-0.125330202	0.064023024	
**577**	46705	T2D-83A_GL0082037	COG3250	Beta-galactosidase/beta-glucuronidase	Carbohydrate transport and metabolism [G]	Pentose and glucuronate interconversions	Bacteroides vulgatus	Species	Bacteroidetes	Bacteroidia	Bacteroidales	Bacteroidaceae	Bacteroides	Bacteroides vulgatus	-0.025440684	0.730745176	
**578**	46846	V1.CD1-0-PT_GL0076248	COG3957	Phosphoketolase	Carbohydrate transport and metabolism [G]	Pentose phosphate pathway	Bifidobacterium bifidum	Species	Actinobacteria	Actinobacteria	Bifidobacteriales	Bifidobacteriaceae	Bifidobacterium	Bifidobacterium bifidum	0.019690236	0.87742313	
**579**	46855	V1.CD1-0-PT_GL0103563	COG0148	Enolase	Carbohydrate transport and metabolism [G]	Glycolysis / Gluconeogenesis	Bifidobacterium bifidum	Species	Actinobacteria	Actinobacteria	Bifidobacteriales	Bifidobacteriaceae	Bifidobacterium	Bifidobacterium bifidum	0.119774657	0.21058711	
**580**	46995	V1.CD1-3-PN_GL0123771	COG0261	Ribosomal protein L21(	Translation, ribosomal structure and biogenesis [J]	Ribosome	Bifidobacterium	Genus	Actinobacteria	Actinobacteria	Bifidobacteriales	Bifidobacteriaceae	Bifidobacterium		-0.106410059	0.063939295	
**581**	47015	V1.CD14-0_GL0091287	COG2182	Maltose-binding periplasmic protein MalE	Carbohydrate transport and metabolism [G]	ABC transporters	Clostridiales	Order	Firmicutes	Clostridia	Clostridiales				-0.195692071	0.199042568	
**582**	47016	V1.CD15-0_GL0019876	COG0747	ABC-type transport system, periplasmic component	Amino acid transport and metabolism [E]	ABC transporters	Clostridiales	Order	Firmicutes	Clostridia	Clostridiales				-0.092015711	0.258319017	
**583**	47191	V1.CD18-0_GL0025638	COG0149	Triosephosphate isomerase	Carbohydrate transport and metabolism [G]	Glycolysis / Gluconeogenesis	Clostridiales	Order	Firmicutes	Clostridia	Clostridiales				-0.092282495	0.096052906	
**584**	47296	V1.CD20-0_GL0142249	COG1410	Methionine synthase I, cobalamin-binding domain	Amino acid transport and metabolism [E]	Cysteine and methionine metabolism	Clostridiales	Order	Firmicutes	Clostridia	Clostridiales				-0.03548187	0.550131166	
**585**	47388	V1.CD2-0-PN_GL0068657	COG1063	Threonine dehydrogenase or related Zn-dependent dehydrogenase	General function prediction only [R]	Fructose and mannose metabolism	Bacteria	Species	Firmicutes	Clostridia	Clostridiales	Lachnospiraceae	Blautia	Blautia sp. KLE 1732	0.01237349	0.829750937	
**586**	47578	V1.CD24-0_GL0085096	COG3716	Phosphotransferase system, mannose/fructose/N-acetylgalactosamine-specific component IID	Carbohydrate transport and metabolism [G]	Fructose and mannose metabolism	Faecalibacterium prausnitzii	Species	Firmicutes	Clostridia	Clostridiales	Ruminococcaceae	Faecalibacterium	Faecalibacterium prausnitzii	-0.032849223	0.673665448	
**587**	47730	V1.CD27-0_GL0136511	COG0222	Ribosomal protein L7/L12	Translation, ribosomal structure and biogenesis [J]	Ribosome	Collinsella	Genus	Actinobacteria	Coriobacteriia	Coriobacteriales	Coriobacteriaceae	Collinsella		0.154548455	0.03811358	*
**588**	47747	V1.CD28-0_GL0110070	COG0448	ADP-glucose pyrophosphorylase	Carbohydrate transport and metabolism [G]	Starch and sucrose metabolism	Clostridiales	Order	Firmicutes	Clostridia	Clostridiales				0.02122764	0.740616016	
**589**	47769	V1.CD29-0_GL0134727	COG1879	ABC-type sugar transport system, periplasmic component, contains N-terminal xre family HTH domain	Carbohydrate transport and metabolism [G]	ABC transporters	Dorea longicatena	Species	Firmicutes	Clostridia	Clostridiales	Lachnospiraceae	Dorea	Dorea longicatena	-0.205115596	0.052697307	
**590**	47793	V1.CD30-0_GL0013170	COG0102	Ribosomal protein L13	Translation, ribosomal structure and biogenesis [J]	Ribosome	Clostridiales	Order	Firmicutes	Clostridia	Clostridiales				-0.066984725	0.182629032	
**591**	47807	V1.CD30-0_GL0138968	COG1070	Sugar (pentulose or hexulose) kinase	Carbohydrate transport and metabolism [G]	Pentose phosphate pathway	Anaerotruncus colihominis	Species	Firmicutes	Clostridia	Clostridiales	Ruminococcaceae	Anaerotruncus	Anaerotruncus colihominis	-0.082074591	0.256952564	
**592**	48122	V1.CD34-0_GL0042016	COG0088	Ribosomal protein L4	Translation, ribosomal structure and biogenesis [J]	Ribosome	Clostridiales	Order	Firmicutes	Clostridia	Clostridiales				-0.055446115	0.480536273	
**593**	48145	V1.CD34-0_GL0135713	COG0332	3-oxoacyl-[acyl-carrier-protein] synthase III	Lipid transport and metabolism [I]	NAN	Dorea formicigenerans	Species	Firmicutes	Clostridia	Clostridiales	Lachnospiraceae	Dorea	Dorea formicigenerans	0.139692127	0.037809947	*
**594**	48199	V1.CD36-0_GL0032080	COG0166	Glucose-6-phosphate isomerase	Carbohydrate transport and metabolism [G]	Glycolysis / Gluconeogenesis	Bifidobacterium	Genus	Actinobacteria	Actinobacteria	Bifidobacteriales	Bifidobacteriaceae	Bifidobacterium		-0.009041568	0.92977455	
**595**	48230	V1.CD36-0_GL0147854	COG0696	Phosphoglycerate mutase (BPG-independent, AlkP superfamily)	Carbohydrate transport and metabolism [G]	Glycolysis / Gluconeogenesis	Collinsella	Genus	Actinobacteria	Coriobacteriia	Coriobacteriales	Coriobacteriaceae	Collinsella		0.126509207	0.082951744	
**596**	48325	V1.CD40-0_GL0053388	COG1653	ABC-type glycerol-3-phosphate transport system, periplasmic component	Carbohydrate transport and metabolism [G]	NAN	Bifidobacterium	Species	Actinobacteria	Actinobacteria	Bifidobacteriales	Bifidobacteriaceae	Bifidobacterium	Bifidobacterium bifidum	-0.09564195	0.141684261	
**597**	48362	V1.CD40-0_GL0150812	COG0039	Malate/lactate dehydrogenase	Energy production and conversion [C]	Glycolysis / Gluconeogenesis	Clostridiales	Order	Firmicutes	Clostridia	Clostridiales				-0.0918346	0.056399357	
**598**	48400	V1.CD41-0_GL0030063	COG3173	Predicted kinase, aminoglycoside phosphotransferase (APT) family	General function prediction only [R]	NAN	Collinsella	Genus	Actinobacteria	Coriobacteriia	Coriobacteriales	Coriobacteriaceae	Collinsella		0.268754813	0.000174674	***
**599**	48444	V1.CD42-0_GL0129731	COG2222	Fructoselysine-6-P-deglycase FrlB and related proteins with duplicated sugar isomerase (SIS) domain	Cell wall/membrane/envelope biogenesis [M]	Alanine, aspartate and glutamate metabolism	Firmicutes	Phylum	Firmicutes						0.069445109	0.446494487	
**600**	48536	V1.CD46-0_GL0008945	COG1454	Alcohol dehydrogenase, class IV	Energy production and conversion [C]	Glycolysis / Gluconeogenesis	Dorea longicatena	Species	Firmicutes	Clostridia	Clostridiales	Lachnospiraceae	Dorea	Dorea longicatena	0.005553549	0.934942439	

**601**	48654	V1.CD50-0_GL0068523	COG0138	AICAR transformylase/IMP cyclohydrolase PurH	Nucleotide transport and metabolism [F]	Purine metabolism	Ruminococcus bromii	Species	Firmicutes	Clostridia	Clostridiales	Ruminococcaceae	Ruminococcus	Ruminococcus bromii	0.150865662	0.022561862	*
**602**	48661	V1.CD50-0_GL0122012	COG2070	NAD(P)H-dependent flavin oxidoreductase YrpB, nitropropane dioxygenase family	General function prediction only [R]	Purine metabolism	Clostridiales	Order	Firmicutes	Clostridia	Clostridiales				0.044006587	0.496566001	
**603**	48670	V1.CD50-0_GL0170135	COG0104	Adenylosuccinate synthase	Nucleotide transport and metabolism [F]	Purine metabolism	Ruminococcaceae	Family	Firmicutes	Clostridia	Clostridiales	Ruminococcaceae			-0.10163887	0.058416237	
**604**	48871	V1.CD54-0_GL0122997	COG0334	Glutamate dehydrogenase/leucine dehydrogenase	Amino acid transport and metabolism [E]	Alanine, aspartate and glutamate metabolism	Firmicutes	Phylum	Firmicutes						0.037617985	0.585761333	
**605**	49073	V1.CD6-4_GL0089549	COG1653	ABC-type glycerol-3-phosphate transport system, periplasmic component	Carbohydrate transport and metabolism [G]	NAN	Bacteria	Superkingdom							-0.021687544	0.780873652	
**606**	49280	V1.CD8-0-PN_GL0136266	COG1454	Alcohol dehydrogenase, class IV	Energy production and conversion [C]	Glycolysis / Gluconeogenesis	Clostridiales	Order	Firmicutes	Clostridia	Clostridiales				0.194085392	0.023295412	*
**607**	49334	V1.FI02_GL0017922	COG0542	ATP-dependent Clp protease ATP-binding subunit ClpA	Posttranslational modification, protein turnover, chaperones [O]	NAN	Blautia obeum	Species	Firmicutes	Clostridia	Clostridiales	Lachnospiraceae	Blautia	Blautia obeum	-0.118867112	0.119182504	
**608**	49363	V1.FI02_GL0189243	COG0443	Molecular chaperone DnaK (HSP70)	Posttranslational modification, protein turnover, chaperones [O]	RNA degradation	Bifidobacterium	Genus	Actinobacteria	Actinobacteria	Bifidobacteriales	Bifidobacteriaceae	Bifidobacterium		-0.071620246	0.37718282	
**609**	49391	V1.FI04_GL0033515	COG1653	ABC-type glycerol-3-phosphate transport system, periplasmic component	Carbohydrate transport and metabolism [G]	NAN	Subdoligranulum variabile	Species	Firmicutes	Clostridia	Clostridiales	Ruminococcaceae	Subdoligranulum	Subdoligranulum variabile	-0.124267522	0.232497235	
**610**	49537	V1.FI05_GL0114531	COG1653	ABC-type glycerol-3-phosphate transport system, periplasmic component	Carbohydrate transport and metabolism [G]	ABC transporters	Bifidobacteriaceae	Species	Actinobacteria	Actinobacteria	Bifidobacteriales	Bifidobacteriaceae	Scardovia	Scardovia wiggsiae	0.009613472	0.915916967	
**611**	49584	V1.FI06_GL0060106	COG0183	Acetyl-CoA acetyltransferase	Lipid transport and metabolism [I]	Fatty acid degradation	Lachnospiraceae	Family	Firmicutes	Clostridia	Clostridiales	Lachnospiraceae			0.072412645	0.310378797	
**612**	49618	V1.FI06_GL0203708	COG1653	ABC-type glycerol-3-phosphate transport system, periplasmic component	Carbohydrate transport and metabolism [G]	ABC transporters	Clostridiales	Order	Firmicutes	Clostridia	Clostridiales				-0.113207639	0.106819911	
**613**	50319	V1.FI17_GL0209634	COG0057	Glyceraldehyde-3-phosphate dehydrogenase/erythrose-4-phosphate dehydrogenase	Carbohydrate transport and metabolism [G]	Glycolysis / Gluconeogenesis	Porphyromonas endodontalis	Species	Bacteroidetes	Bacteroidia	Bacteroidales	Porphyromonadaceae	Porphyromonas	Porphyromonas endodontalis	0.022799482	0.774724903	
**614**	50520	V1.FI22_GL0003131	COG1653	ABC-type glycerol-3-phosphate transport system, periplasmic component	Carbohydrate transport and metabolism [G]	ABC transporters	Clostridiales	Order	Firmicutes	Clostridia	Clostridiales				-0.113438808	0.119932632	
**615**	50549	V1.FI22_GL0137796	COG0096	Ribosomal protein S8	Translation, ribosomal structure and biogenesis [J]	Ribosome	Clostridiales	Order	Firmicutes	Clostridia	Clostridiales				-0.048660671	0.412185419	
**616**	50615	V1.FI25_GL0042905	COG2407	L-fucose isomerase or related protein	Carbohydrate transport and metabolism [G]	Fructose and mannose metabolism	Clostridiales	Order	Firmicutes	Clostridia	Clostridiales				-0.079747703	0.293675256	
**617**	50690	V1.FI26_GL0267199	COG0737	2',3'-cyclic-nucleotide 2'-phosphodiesterase/5'- or 3'-nucleotidase, 5'-nucleotidase family	Defense mechanisms [V]	NAN	Flavobacterium gilvum	Species	Bacteroidetes	Flavobacteriia	Flavobacteriales	Flavobacteriaceae	Flavobacterium	Flavobacterium gilvum	0.098692431	0.258628031	
**618**	50707	V1.FI27_GL0072285	COG5426	Uncharacterized membrane protein	Function unknown [S]	NAN	Bifidobacterium longum	Species	Actinobacteria	Actinobacteria	Bifidobacteriales	Bifidobacteriaceae	Bifidobacterium	Bifidobacterium longum	0.013380467	0.867353229	
**619**	50727	V1.FI28_GL0009969	COG1454	Alcohol dehydrogenase, class IV	Energy production and conversion [C]	Glycolysis / Gluconeogenesis	Bacteria	Superkingdom							-0.074928111	0.299420532	
**620**	50866	V1.FI31_GL0134522	COG0334	Glutamate dehydrogenase/leucine dehydrogenase	Amino acid transport and metabolism [E]	Alanine, aspartate and glutamate metabolism	Oscillibacter	Genus	Firmicutes	Clostridia	Clostridiales	Oscillospiraceae	Oscillibacter		-0.1047713	0.1930941	
**621**	51063	V1.FI36_GL0024061	COG0191	Fructose/tagatose bisphosphate aldolase	Carbohydrate transport and metabolism [G]	Glycolysis / Gluconeogenesis	Dorea	Genus	Firmicutes	Clostridia	Clostridiales	Lachnospiraceae	Dorea		-0.081257022	0.335854833	
**622**	51159	V1.FI37_GL0030438	COG1825	Ribosomal protein L25 (general stress protein Ctc)	Translation, ribosomal structure and biogenesis [J]	Ribosome	Bifidobacterium	Genus	Actinobacteria	Actinobacteria	Bifidobacteriales	Bifidobacteriaceae	Bifidobacterium		-0.12707378	0.098642212	
**623**	51276	V1.UC11-0_GL0085451	COG0244	Ribosomal protein L10	Translation, ribosomal structure and biogenesis [J]	Ribosome	Bifidobacterium	Genus	Actinobacteria	Actinobacteria	Bifidobacteriales	Bifidobacteriaceae	Bifidobacterium		-0.019643991	0.818073164	
**624**	51478	V1.UC13-3_GL0060573	COG0360	Ribosomal protein S6	Translation, ribosomal structure and biogenesis [J]	Ribosome	Bifidobacterium	Genus	Actinobacteria	Actinobacteria	Bifidobacteriales	Bifidobacteriaceae	Bifidobacterium		-0.073497618	0.358166896	
**625**	51690	V1.UC17-2_GL0123387	COG1145	Ferredoxin	Energy production and conversion [C]	Glycolysis / Gluconeogenesis	Collinsella aerofaciens	Species	Actinobacteria	Coriobacteriia	Coriobacteriales	Coriobacteriaceae	Collinsella	Collinsella aerofaciens	0.156406784	0.118277341	
**626**	51788	V1.UC22-1_GL0166855	COG4577	Carboxysome shell and ethanolamine utilization microcompartment protein CcmL/EutN	Secondary metabolites biosynthesis, transport and catabolism [Q]	NAN	Clostridiales	Order	Firmicutes	Clostridia	Clostridiales				-0.008967148	0.845489043	
**627**	52389	V1.UC38-4_GL0131494	COG0088	Ribosomal protein L4	Translation, ribosomal structure and biogenesis [J]	Ribosome	Clostridiales	Order	Firmicutes	Clostridia	Clostridiales				-0.014880091	0.81519354	
**628**	52494	V1.UC40-0_GL0007441	COG0057	Glyceraldehyde-3-phosphate dehydrogenase/erythrose-4-phosphate dehydrogenase	Carbohydrate transport and metabolism [G]	Glycolysis / Gluconeogenesis	Anaerostipes caccae	Species	Firmicutes	Clostridia	Clostridiales	Lachnospiraceae	Anaerostipes	Anaerostipes caccae	-0.190544641	0.022222845	*
**629**	52509	V1.UC40-0_GL0130575	COG3209	Uncharacterized conserved protein RhaS, contains 28 RHS repeats	General function prediction only [R]	NAN	Bacillus subtilis	Species	Firmicutes	Bacilli	Bacillales	Bacillaceae	Bacillus	Bacillus subtilis	-0.039210396	0.72202032	
**630**	52542	V1.UC40-1_GL0106359	COG0589	Nucleotide-binding universal stress protein, UspA family	Signal transduction mechanisms [T]	NAN	Bifidobacterium	Genus	Actinobacteria	Actinobacteria	Bifidobacteriales	Bifidobacteriaceae	Bifidobacterium		-0.103353198	0.168563463	
**631**	52557	V1.UC40-1_GL0166114	COG2759	Formyltetrahydrofolate synthetase	Nucleotide transport and metabolism [F]	One carbon pool by folate	Lachnospiraceae	Family	Firmicutes	Clostridia	Clostridiales	Lachnospiraceae			-0.041065937	0.491409966	
**632**	52581	V1.UC41-0_GL0045739	COG1653	ABC-type glycerol-3-phosphate transport system, periplasmic component	Carbohydrate transport and metabolism [G]	ABC transporters	Faecalibacterium prausnitzii	Species	Firmicutes	Clostridia	Clostridiales	Ruminococcaceae	Faecalibacterium	Faecalibacterium prausnitzii	-0.08852439	0.451499607	
**633**	52610	V1.UC42-0_GL0049062	COG0329	Dihydrodipicolinate synthase/N-acetylneuraminate lyase	Cell wall/membrane/envelope biogenesis [M]	Lysine biosynthesis	Clostridiales	Order	Firmicutes	Clostridia	Clostridiales				0.120053578	0.012950606	*
**634**	52781	V1.UC48-0_GL0060046			NAN	NAN	Alistipes putredinis	Species	Bacteroidetes	Bacteroidia	Bacteroidales	Rikenellaceae	Alistipes	Alistipes putredinis	0.161601748	0.01487518	*
**635**	52954	V1.UC51-4_GL0052281	COG1879	ABC-type sugar transport system, periplasmic component, contains N-terminal xre family HTH domain	Carbohydrate transport and metabolism [G]	ABC transporters	Clostridiales	Order	Firmicutes	Clostridia	Clostridiales				-0.221944669	0.007338031	**
**636**	53140	V1.UC53-4_GL0037562	COG0087	Ribosomal protein L3	Translation, ribosomal structure and biogenesis [J]	Ribosome	Collinsella	Genus	Actinobacteria	Coriobacteriia	Coriobacteriales	Coriobacteriaceae	Collinsella		0.073439928	0.219505072	
**637**	53397	V1.UC57-0_GL0145554	COG0264	Translation elongation factor EF-Ts	Translation, ribosomal structure and biogenesis [J]	NAN	Clostridiales	Order	Firmicutes	Clostridia	Clostridiales				-0.076618939	0.170464407	

**638**	53544	V1.UC60-0_GL0052142	COG0047	Phosphoribosylformylglycinamidine (FGAM) synthase, glutamine amidotransferase domain	Nucleotide transport and metabolism [F]	Purine metabolism	Clostridiales	Order	Firmicutes	Clostridia	Clostridiales				-0.085159005	0.217843141	

PGs with loadings coefficient on PC1 of PCA > |0.08| on which MANOVA test was performed

N	Protein Group ID	Leading razor protein accession	COG accession	COG name	COG category	KEGG name	LCA	Rank	Phylum	Class	Order	Family	Genus	Species	Log_{10} (WBS/CTRL)	t-test p-value WBS/CTRL	Significance WBS/CTRL
1	115	206672.BL0707	COG0126	3-phosphoglycerate kinase	Carbohydrate transport and metabolism [G]	Glycolysis / Gluconeogenesis	Bifidobacterium longum	Species	Actinobacteria	Actinobacteria	Bifidobacteriales	Bifidobacteriaceae	Bifidobacterium	Bifidobacterium longum	0.0485	0.6503	
2	118	206672.BL0951	COG1882	Pyruvate-formate lyase	Energy production and conversion [C]	Pyruvate metabolism	Bifidobacterium	Genus	Actinobacteria	Actinobacteria	Bifidobacteriales	Bifidobacteriaceae	Bifidobacterium		0.0552	0.5906	
3	122	206672.BL0988	COG0469	Pyruvate kinase	Carbohydrate transport and metabolism [G]	Glycolysis / Gluconeogenesis	Bifidobacterium	Genus	Actinobacteria	Actinobacteria	Bifidobacteriales	Bifidobacteriaceae	Bifidobacterium		-0.0081	0.9507	
4	340	391904.BLIJ_0145	COG0443	Molecular chaperone DnaK (HSP70)	Posttranslational modification, protein turnover, chaperones [O]	RNA degradation	Bifidobacterium	Genus	Actinobacteria	Actinobacteria	Bifidobacteriales	Bifidobacteriaceae	Bifidobacterium		-0.0018	0.9880	
5	525	411485.FAEPRAM212_01761	COG1145	Ferredoxin	Energy production and conversion [C]	Glycolysis / Gluconeogenesis	Faecalibacterium prausnitzii	Species	Firmicutes	Clostridia	Clostridiales	Ruminococcaceae	Faecalibacterium	Faecalibacterium prausnitzii	-0.2203	0.0859	
6	939	515619.EUBREC_1472	COG1145	Ferredoxin	Energy production and conversion [C]	Glycolysis / Gluconeogenesis	Clostridiales	Order	Firmicutes	Clostridia	Clostridiales				-0.4149	0.0086	**
7	1139	537937.BLIG_01296	COG4166	ABC-type oligopeptide transport system, periplasmic component	Amino acid transport and metabolism [E]	ABC transporters	Bifidobacterium	Genus	Actinobacteria	Actinobacteria	Bifidobacteriales	Bifidobacteriaceae	Bifidobacterium		0.1433	0.1833	
8	1349	566552.BIFCAT_00987	COG0282	Acetate kinase	Energy production and conversion [C]	Taurine and hypotaurine metabolism	Bifidobacterium	Genus	Actinobacteria	Actinobacteria	Bifidobacteriales	Bifidobacteriaceae	Bifidobacterium		0.0006	0.9951	
9	1633	759350.BLJ_0360	COG0228	Ribosomal protein S16	Translation, ribosomal structure and biogenesis [J]	Ribosome	Bifidobacterium	Genus	Actinobacteria	Actinobacteria	Bifidobacteriales	Bifidobacteriaceae	Bifidobacterium		0.0567	0.5208	
10	2809	DLM018_GL0016638	COG0092	Ribosomal protein S3	Translation, ribosomal structure and biogenesis [J]	Ribosome	Bifidobacterium	Genus	Actinobacteria	Actinobacteria	Bifidobacteriales	Bifidobacteriaceae	Bifidobacterium		0.0426	0.6627	
11	17817	MH0122_GL0107606	COG1653	ABC-type glycerol-3-phosphate transport system, periplasmic component	Carbohydrate transport and metabolism [G]	ABC transporters	Bifidobacterium	Genus	Actinobacteria	Actinobacteria	Bifidobacteriales	Bifidobacteriaceae	Bifidobacterium		0.1901	0.1332	
12	18868	MH0131_GL0154213	COG0544	FKBP-type peptidyl-prolyl cis-trans isomerase (trigger factor)	Posttranslational modification, protein turnover, chaperones [O]	NAN	Bifidobacterium	Genus	Actinobacteria	Actinobacteria	Bifidobacteriales	Bifidobacteriaceae	Bifidobacterium		0.1452	0.1380	
13	20757	MH0161_GL0142425	COG0148	Enolase	Carbohydrate transport and metabolism [G]	Glycolysis / Gluconeogenesis	Bifidobacterium adolescentis	Species	Actinobacteria	Actinobacteria	Bifidobacteriales	Bifidobacteriaceae	Bifidobacterium	Bifidobacterium adolescentis	-0.0356	0.7552	
14	21833	MH0188_GL0007212	COG0039	Malate/lactate dehydrogenase	Energy production and conversion [C]	Glycolysis / Gluconeogenesis	Bifidobacterium	Genus	Actinobacteria	Actinobacteria	Bifidobacteriales	Bifidobacteriaceae	Bifidobacterium		0.0711	0.4400	
15	21984	MH0188_GL0055295	COG1653	ABC-type glycerol-3-phosphate transport system, periplasmic component	Carbohydrate transport and metabolism [G]	ABC transporters	Bifidobacterium	Genus	Actinobacteria	Actinobacteria	Bifidobacteriales	Bifidobacteriaceae	Bifidobacterium		0.3191	0.0046	**
16	22027	MH0188_GL0067976	COG1129	ABC-type sugar transport system, ATPase component	Carbohydrate transport and metabolism [G]	ABC transporters	Bifidobacterium	Genus	Actinobacteria	Actinobacteria	Bifidobacteriales	Bifidobacteriaceae	Bifidobacterium		0.0242	0.7906	
17	22030	MH0188_GL0069412	COG0282	Acetate kinase	Energy production and conversion [C]	Taurine and hypotaurine metabolism	Bifidobacterium	Genus	Actinobacteria	Actinobacteria	Bifidobacteriales	Bifidobacteriaceae	Bifidobacterium		0.1031	0.2201	
18	22090	MH0188_GL0095502	COG0174	Glutamine synthetase	Amino acid transport and metabolism [E]	Alanine, aspartate and glutamate metabolism	Bifidobacterium	Genus	Actinobacteria	Actinobacteria	Bifidobacteriales	Bifidobacteriaceae	Bifidobacterium		-0.0276	0.8224	
19	22205	MH0188_GL0126744	COG0459	Chaperonin GroEL (HSP60 family)	Posttranslational modification, protein turnover, chaperones [O]	RNA degradation	Bifidobacterium	Genus	Actinobacteria	Actinobacteria	Bifidobacteriales	Bifidobacteriaceae	Bifidobacterium		0.0325	0.7501	
20	22221	MH0188_GL0133256	COG1087	UDP-glucose 4-epimerase	Cell wall/membrane/envelope biogenesis [M]	Galactose metabolism	Bifidobacterium longum	Species	Actinobacteria	Actinobacteria	Bifidobacteriales	Bifidobacteriaceae	Bifidobacterium	Bifidobacterium longum	0.0910	0.3510	
21	22254	MH0188_GL0145450	COG1882	Pyruvate-formate lyase	Energy production and conversion [C]	Pyruvate metabolism	Bifidobacterium adolescentis	Species	Actinobacteria	Actinobacteria	Bifidobacteriales	Bifidobacteriaceae	Bifidobacterium	Bifidobacterium adolescentis	-0.1469	0.2235	
22	22837	MH0193_GL0010680	COG0480	Translation elongation factor EF-G, a GTPase	Translation, ribosomal structure and biogenesis [J]	NAN	Bifidobacterium	Genus	Actinobacteria	Actinobacteria	Bifidobacteriales	Bifidobacteriaceae	Bifidobacterium		0.0382	0.7775	
23	23014	MH0193_GL0186495	COG1454	Alcohol dehydrogenase, class IV	Energy production and conversion [C]	Glycolysis / Gluconeogenesis	Bifidobacterium	Genus	Actinobacteria	Actinobacteria	Bifidobacteriales	Bifidobacteriaceae	Bifidobacterium		0.1155	0.3397	
24	23687	MH0203_GL0013763	COG0057	Glyceraldehyde-3-phosphate dehydrogenase/erythrose-4-phosphate dehydrogenase	Carbohydrate transport and metabolism [G]	Glycolysis / Gluconeogenesis	Bifidobacterium	Genus	Actinobacteria	Actinobacteria	Bifidobacteriales	Bifidobacteriaceae	Bifidobacterium		0.0774	0.4331	
25	23823	MH0203_GL0133062	COG0033	Phosphoglucomutase	Carbohydrate transport and metabolism [G]	Glycolysis / Gluconeogenesis	Bifidobacterium	Genus	Actinobacteria	Actinobacteria	Bifidobacteriales	Bifidobacteriaceae	Bifidobacterium		0.0275	0.7815	
26	27731	MH0274_GL0075860	COG0539	Ribosomal protein S1	Translation, ribosomal structure and biogenesis [J]	Ribosome	Bifidobacterium	Genus	Actinobacteria	Actinobacteria	Bifidobacteriales	Bifidobacteriaceae	Bifidobacterium		-0.1634	0.1119	
27	28314	MH0284_GL0116366	COG0183	Acetyl-CoA acetyltransferase	Lipid transport and metabolism [I]	Fatty acid degradation	Clostridiales	Order	Firmicutes	Clostridia	Clostridiales				-0.1731	0.1747	
28	28712	MH0293_GL0090794	COG1080	Phosphoenolpyruvate-protein kinase (PTS system EI component in bacteria)	Carbohydrate transport and metabolism [G]	Pyruvate metabolism	Clostridiales	Order	Firmicutes	Clostridia	Clostridiales				-0.4010	0.0054	**
29	30556	MH0341_GL0013504	COG0176	Transaldolase	Carbohydrate transport and metabolism [G]	Pentose phosphate pathway	Bifidobacterium	Genus	Actinobacteria	Actinobacteria	Bifidobacteriales	Bifidobacteriaceae	Bifidobacterium		-0.0586	0.6444	
30	30623	MH0341_GL0099782	COG0050	Translation elongation factor EF-Tu, a GTPase	Translation, ribosomal structure and biogenesis [J]	Plant-pathogen interaction	Bifidobacterium longum	Species	Actinobacteria	Actinobacteria	Bifidobacteriales	Bifidobacteriaceae	Bifidobacterium	Bifidobacterium longum	0.0039	0.9674	
31	30834	MH0347_GL0010219	COG1653	ABC-type glycerol-3-phosphate transport system, periplasmic component	Carbohydrate transport and metabolism [G]	NAN	Bifidobacterium	Genus	Actinobacteria	Actinobacteria	Bifidobacteriales	Bifidobacteriaceae	Bifidobacterium		-0.0374	0.7522	
32	30909	MH0348_GL0122167	COG0085	DNA-directed RNA polymerase, beta subunit/140 kD subunit	Transcription [K]	Purine metabolism	Bifidobacterium	Genus	Actinobacteria	Actinobacteria	Bifidobacteriales	Bifidobacteriaceae	Bifidobacterium		-0.0287	0.7703	
33	41485	O2.UC32-0_GL0051137	COG2160	L-arabinose isomerase	Carbohydrate transport and metabolism [G]	Pentose and glucuronate interconversions	Bifidobacterium	Genus	Actinobacteria	Actinobacteria	Bifidobacteriales	Bifidobacteriaceae	Bifidobacterium		-0.0629	0.5878	
34	41889	O2.UC37-1_GL0081003	COG0021	Transketolase	Carbohydrate transport and metabolism [G]	Pentose phosphate pathway	Bifidobacterium bifidum	Species	Actinobacteria	Actinobacteria	Bifidobacteriales	Bifidobacteriaceae	Bifidobacterium	Bifidobacterium bifidum	0.0119	0.9142	
35	43491	O2.UC58-2_GL0017801	COG0360	Ribosomal protein S6	Translation, ribosomal structure and biogenesis [J]	Ribosome	Bifidobacterium	Genus	Actinobacteria	Actinobacteria	Bifidobacteriales	Bifidobacteriaceae	Bifidobacterium		0.1295	0.1380	
36	45166	T2D-122A_GL0083846	COG0176	Transaldolase	Carbohydrate transport and metabolism [G]	Pentose phosphate pathway	Bifidobacterium bifidum	Species	Actinobacteria	Actinobacteria	Bifidobacteriales	Bifidobacteriaceae	Bifidobacterium	Bifidobacterium bifidum	-0.0424	0.7140	

**37**	46846	V1.CD1-0-PT_GL0076248	COG3957	Phosphoketolase	Carbohydrate transport and metabolism [G]	Pentose phosphate pathway	Bifidobacterium bifidum	Species	Actinobacteria	Actinobacteria	Bifidobacteriales	Bifidobacteriaceae	Bifidobacterium	Bifidobacterium bifidum	0.0197	0.8774	
**38**	46855	V1.CD1-0-PT_GL0103563	COG0148	Enolase	Carbohydrate transport and metabolism [G]	Glycolysis / Gluconeogenesis	Bifidobacterium bifidum	Species	Actinobacteria	Actinobacteria	Bifidobacteriales	Bifidobacteriaceae	Bifidobacterium	Bifidobacterium bifidum	0.1198	0.2106	
**39**	48199	V1.CD36-0_GL0032080	COG0166	Glucose-6-phosphate isomerase	Carbohydrate transport and metabolism [G]	Glycolysis / Gluconeogenesis	Bifidobacterium	Genus	Actinobacteria	Actinobacteria	Bifidobacteriales	Bifidobacteriaceae	Bifidobacterium		-0.0090	0.9298	
**40**	51276	V1.UC11-0_GL0085451	COG0244	Ribosomal protein L10	Translation, ribosomal structure and biogenesis [J]	Ribosome	Bifidobacterium	Genus	Actinobacteria	Actinobacteria	Bifidobacteriales	Bifidobacteriaceae	Bifidobacterium		-0.0196	0.8181	

PGs with VIP > 2

N	Protein Group ID	Leading razor protein accession	COG accession	COG name	COG category	KEGG name	LCA	Rank	Phylum	Class	Order	Family	Genus	Species	Log_{10} (WBS/CTRL)	t-test p-value WBS/CTRL	Significance WBS/CTRL
1	11241	MH0032_GL0024527	COG0090	Ribosomal protein L2	Translation, ribosomal structure and biogenesis [J]	Ribosome	Dialister succinatiphilus	Species	Firmicutes	Negativicutes	Veillonellales	Veillonellaceae	Dialister	Dialister succinatiphilus	-0.2527	0.0006	***
2	11766	MH0041_GL0040403	COG1866	Phosphoenolpyruvate carboxykinase, ATP-dependent	Energy production and conversion [C]	Glycolysis / Gluconeogenesis	Roseburia faecis	Species	Firmicutes	Clostridia	Clostridiales	Lachnospiraceae	Roseburia	Roseburia faecis	-0.3255	0.0001	***
3	17690	MH0122_GL0011708	COG0050	Translation elongation factor EF-Tu, a GTPase	Translation, ribosomal structure and biogenesis [J]	Plant-pathogen interaction	Subdoligranulum variabile	Species	Firmicutes	Clostridia	Clostridiales	Ruminococcaceae	Subdoligranulum	Subdoligranulum variabile	-0.4345	0.0002	***
4	18705	MH0131_GL0105124	COG2235	Arginine deiminase	Amino acid transport and metabolism [E]	Arginine and proline metabolism	Collinsella	Genus	Actinobacteria	Coriobacteriia	Coriobacteriales	Coriobacteriaceae	Collinsella		0.4035	0.0001	***
5	18959	MH0131_GL0175176	COG1882	Pyruvate-formate lyase	Energy production and conversion [C]	Pyruvate metabolism	Collinsella	Genus	Actinobacteria	Coriobacteriia	Coriobacteriales	Coriobacteriaceae	Collinsella		0.3266	0.0002	***
6	31674	MH0364_GL0101039	COG0057	Glyceraldehyde-3-phosphate dehydrogenase/erythrose-4-phosphate dehydrogenase	Carbohydrate transport and metabolism [G]	Glycolysis / Gluconeogenesis	Ruminococcus bicirculans	Species	Firmicutes	Clostridia	Clostridiales	Ruminococcaceae	Ruminococcus	Ruminococcus bicirculans	-0.4198	0.0004	***
7	32128	MH0371_GL0055922	COG1538	Outer membrane protein TolC	Cell wall/membrane/envelope biogenesis [M]	Bacterial secretion system	Bacteroides	Genus	Bacteroidetes	Bacteroidia	Bacteroidales	Bacteroidaceae	Bacteroides		0.2626	0.0006	***
8	37103	N079A_GL0025412	COG0049	Ribosomal protein S7	Translation, ribosomal structure and biogenesis [J]	Ribosome	Faecalibacterium prausnitzii	Species	Firmicutes	Clostridia	Clostridiales	Ruminococcaceae	Faecalibacterium	Faecalibacterium prausnitzii	-0.2067	0.0004	***
9	41419	O2.UC31-1_GL0115264	COG0085	DNA-directed RNA polymerase, beta subunit/140 kD subunit	Transcription [K]	Purine metabolism	Clostridiales	Order	Firmicutes	Clostridia	Clostridiales				-0.2650	0.0006	***
10	42060	O2.UC40-1_GL0122610	COG1882	Pyruvate-formate lyase	Energy production and conversion [C]	Pyruvate metabolism	Clostridiales bacterium KLE1615	Species	Firmicutes	Clostridia	Clostridiales			Clostridiales bacterium KLE1615	-0.3057	0.0008	***
11	42835	O2.UC48-0_GL0135226	COG1834	N-Dimethylarginine dimethylaminohydrolase	Amino acid transport and metabolism [E]	Glycine, serine and threonine metabolism	Bacteria	Superkingdom							0.4932	0.0000	***
12	42974	O2.UC48-1_GL0053868	COG0059	Ketol-acid reductoisomerase	Coenzyme transport and metabolism [H]	Valine, leucine and isoleucine biosynthesis	Clostridiales	Order	Firmicutes	Clostridia	Clostridiales				0.2206	0.0004	***
13	4426	MH0002_GL0001930	COG0050	Translation elongation factor EF-Tu, a GTPase	Translation, ribosomal structure and biogenesis [J]	Plant-pathogen interaction	Clostridiales	Species	Firmicutes	Clostridia	Clostridiales	Ruminococcaceae	Subdoligranulum	Subdoligranulum variabile	-0.3364	0.0000	***
14	4632	MH0002_GL0018942	COG0085	DNA-directed RNA polymerase, beta subunit/140 kD subunit	Transcription [K]	Purine metabolism	Bacteria	Species	Actinobacteria	Coriobacteriia	Coriobacteriales	Coriobacteriaceae	Coriobacterium	Coriobacterium glomerans	-0.1923	0.0009	***
15	48400	V1.CD41-0_GL0030063	COG3173	Predicted kinase, aminoglycoside phosphotransferase (APT) family	General function prediction only [R]	NAN	Collinsella	Genus	Actinobacteria	Coriobacteriia	Coriobacteriales	Coriobacteriaceae	Collinsella		0.2688	0.0002	***
16	4915	MH0002_GL0040735	COG3957	Phosphoketolase	Carbohydrate transport and metabolism [G]	Pentose phosphate pathway	Mycobacterium bohemicum	Species	Actinobacteria	Actinobacteria	Corynebacteriales	Mycobacteriaceae	Mycobacterium	Mycobacterium bohemicum	-0.4525	0.0000	***
17	4916	MH0002_GL0040736	COG2407	L-fucose isomerase or related protein	Carbohydrate transport and metabolism [G]	Fructose and mannose metabolism	Subdoligranulum variabile	Species	Firmicutes	Clostridia	Clostridiales	Ruminococcaceae	Subdoligranulum	Subdoligranulum variabile	-0.5099	0.0000	***
18	4995	MH0002_GL0047744			NAN	NAN	Bacteria	Superkingdom							0.2415	0.0009	***
19	5035	MH0002_GL0050697	COG1726	Na+-transporting NADH:ubiquinone oxidoreductase, subunit NqrA	Energy production and conversion [C]	NAN	Bacteroides	Genus	Bacteroidetes	Bacteroidia	Bacteroidales	Bacteroidaceae	Bacteroides		0.2570	0.0006	***
20	5329	MH0002_GL0076888	COG0457	Tetratricopeptide (TPR) repeat	General function prediction only [R]	NAN	Bacteroides	Genus	Bacteroidetes	Bacteroidia	Bacteroidales	Bacteroidaceae	Bacteroides		0.4011	0.0003	***
21	8811	MH0012_GL0079061	COG0282	Acetate kinase	Energy production and conversion [C]	Taurine and hypotaurine metabolism	Clostridium pasteurianum	Species	Firmicutes	Clostridia	Clostridiales	Clostridiaceae	Clostridium	Clostridium pasteurianum	-0.3453	0.0001	***

89 differentially expressed PGs

N	Protein Group ID	Leading razor protein accession	COG accession	COG name	COG category	KEGG name	LCA	Rank	Phylum	Class	Order	Family	Genus	Species	Log_{10} (WBS/CTRL)	p-value WBS/CTRL	Significance WBS/CTRL
1	42835	O2.UC48-0_GL0135226	COG1834	N-Dimethylarginine dimethylaminohydrolase	Amino acid transport and metabolism [E]	Glycine, serine and threonine metabolism	Bacteria	Superkingdom							0.4932	0.0000	***
2	2699	DLM013_GL0037207	COG1196	Chromosome segregation ATPase	Cell cycle control, cell division, chromosome partitioning [D]	NAN	Bacteroides dorei	Species	Bacteroidetes	Bacteroidia	Bacteroidales	Bacteroidaceae	Bacteroides	Bacteroides dorei	0.4774	0.0114	*
3	18705	MH0131_GL0105124	COG2235	Arginine deiminase	Amino acid transport and metabolism [E]	Arginine and proline metabolism	Collinsella	Genus	Actinobacteria	Coriobacteriia	Coriobacteriales	Coriobacteriaceae	Collinsella		0.4035	0.0001	***
4	5329	MH0002_GL0076888	COG0457	Tetratricopeptide (TPR) repeat	General function prediction only [R]	NAN	Bacteroides	Genus	Bacteroidetes	Bacteroidia	Bacteroidales	Bacteroidaceae	Bacteroides		0.4011	0.0003	***
5	24946	MH0227_GL0100580	COG0057	Glyceraldehyde-3-phosphate dehydrogenase/erythrose-4-phosphate dehydrogenase	Carbohydrate transport and metabolism [G]	Glycolysis / Gluconeogenesis	Bacteroides eggerthii	Species	Bacteroidetes	Bacteroidia	Bacteroidales	Bacteroidaceae	Bacteroides	Bacteroides eggerthii	0.3576	0.0028	**
6	34973	MH0432_GL0050497	COG5263	Glucan-binding domain (YG repeat)	Carbohydrate transport and metabolism [G]	NAN	Clostridium	Genus	Firmicutes	Clostridia	Clostridiales	Clostridiaceae	Clostridium		0.3542	0.0067	**
7	18959	MH0131_GL0175176	COG1882	Pyruvate-formate lyase	Energy production and conversion [C]	Pyruvate metabolism	Collinsella	Genus	Actinobacteria	Coriobacteriia	Coriobacteriales	Coriobacteriaceae	Collinsella		0.3266	0.0002	***
8	21984	MH0188_GL0055295	COG1653	ABC-type glycerol-3-phosphate transport system, periplasmic component	Carbohydrate transport and metabolism [G]	ABC transporters	Bifidobacterium	Genus	Actinobacteria	Actinobacteria	Bifidobacteriales	Bifidobacteriaceae	Bifidobacterium		0.3191	0.0046	**
9	45270	T2D-135A_GL0000682	COG4771	Outer membrane receptor for ferrienterochelin and colicins	Inorganic ion transport and metabolism [P]	NAN	Bacteroides massiliensis	Species	Bacteroidetes	Bacteroidia	Bacteroidales	Bacteroidaceae	Bacteroides	Bacteroides massiliensis	0.2935	0.0042	**
10	5952	MH0003_GL0082437	COG4206	Outer membrane cobalamin receptor protein	Coenzyme transport and metabolism [H]	NAN	Bacteroides	Genus	Bacteroidetes	Bacteroidia	Bacteroidales	Bacteroidaceae	Bacteroides		0.2919	0.0322	*
11	9728	MH0014_GL0097483	COG1592	Rubrerythrin	Energy production and conversion [C]	NAN	Clostridiales	Order	Firmicutes	Clostridia	Clostridiales				0.2813	0.0015	**
12	11463	MH0037_GL0027576			NAN	NAN	Bacteria	Superkingdom							0.2779	0.0011	**
13	16733	MH0106_GL0002286	COG1053	Succinate dehydrogenase/fumarate reductase, flavoprotein subunit	Energy production and conversion [C]	Citrate cycle (TCA cycle)	Bacteroides coprocola	Species	Bacteroidetes	Bacteroidia	Bacteroidales	Bacteroidaceae	Bacteroides	Bacteroides coprocola	0.2778	0.0016	**
14	572	411903.COLAER_02212	COG4166	ABC-type oligopeptide transport system, periplasmic component	Amino acid transport and metabolism [E]	ABC transporters	Collinsella aerofaciens	Species	Actinobacteria	Coriobacteriia	Coriobacteriales	Coriobacteriaceae	Collinsella	Collinsella aerofaciens	0.2769	0.0037	**
15	48400	V1.CD41-0_GL0030063	COG3173	Predicted kinase, aminoglycoside phosphotransferase (APT) family	General function prediction only [R]	NAN	Collinsella	Genus	Actinobacteria	Coriobacteriia	Coriobacteriales	Coriobacteriaceae	Collinsella		0.2688	0.0002	***
16	31916	MH0370_GL0042621	COG4771	Outer membrane receptor for ferrienterochelin and colicins	Inorganic ion transport and metabolism [P]	NAN	Bacteroides uniformis	Species	Bacteroidetes	Bacteroidia	Bacteroidales	Bacteroidaceae	Bacteroides	Bacteroides uniformis	0.2649	0.0218	*
17	32128	MH0371_GL0055922	COG1538	Outer membrane protein TolC	Cell wall/membrane/envelope biogenesis [M]	Bacterial secretion system	Bacteroides	Genus	Bacteroidetes	Bacteroidia	Bacteroidales	Bacteroidaceae	Bacteroides		0.2626	0.0006	***
18	5035	MH0002_GL0050697	COG1726	Na+-transporting NADH:ubiquinone oxidoreductase, subunit NqrA	Energy production and conversion [C]	NAN	Bacteroides	Genus	Bacteroidetes	Bacteroidia	Bacteroidales	Bacteroidaceae	Bacteroides		0.2570	0.0006	***
19	28480	MH0288_GL0054326	COG4774	Outer membrane receptor for monomeric catechols	Inorganic ion transport and metabolism [P]	NAN	Bacteroides vulgatus	Species	Bacteroidetes	Bacteroidia	Bacteroidales	Bacteroidaceae	Bacteroides	Bacteroides vulgatus	0.2559	0.0478	*
20	32014	MH0370_GL0093205	COG2222	Fructoselysine-6-P-deglycase FrlB and related proteins with duplicated sugar isomerase (SIS) domain	Cell wall/membrane/envelope biogenesis [M]	Alanine, aspartate and glutamate metabolism	Collinsella aerofaciens	Species	Actinobacteria	Coriobacteriia	Coriobacteriales	Coriobacteriaceae	Collinsella	Collinsella aerofaciens	0.2454	0.0015	**
21	13391	MH0062_GL0058668	COG4166	ABC-type oligopeptide transport system, periplasmic component	Amino acid transport and metabolism [E]	ABC transporters	Faecalibacterium prausnitzii	Species	Firmicutes	Clostridia	Clostridiales	Ruminococcaceae	Faecalibacterium	Faecalibacterium prausnitzii	0.2449	0.0052	**
22	4995	MH0002_GL0047744			NAN	NAN	Bacteria	Superkingdom							0.2415	0.0009	***
23	18527	MH0131_GL0047971	COG0469	Pyruvate kinase	Carbohydrate transport and metabolism [G]	Glycolysis / Gluconeogenesis	Collinsella aerofaciens	Species	Actinobacteria	Coriobacteriia	Coriobacteriales	Coriobacteriaceae	Collinsella	Collinsella aerofaciens	0.2410	0.0056	**
24	12181	MH0048_GL0045161	COG0334	Glutamate dehydrogenase/leucine dehydrogenase	Amino acid transport and metabolism [E]	Alanine, aspartate and glutamate metabolism	Eubacterium	Genus	Firmicutes	Clostridia	Clostridiales	Eubacteriaceae	Eubacterium		0.2374	0.0045	**
25	5748	MH0003_GL0052996			NAN	NAN	Bacteroides	Genus	Bacteroidetes	Bacteroidia	Bacteroidales	Bacteroidaceae	Bacteroides		0.2373	0.0297	*
26	5650	MH0003_GL0042541	COG3637	Opacity protein and related surface antigens	Cell wall/membrane/envelope biogenesis [M]	NAN	Bacteroides	Genus	Bacteroidetes	Bacteroidia	Bacteroidales	Bacteroidaceae	Bacteroides		0.2361	0.0036	**
27	42713	O2.UC47-2_GL0030333	COG1082	Sugar phosphate isomerase/epimerase	Carbohydrate transport and metabolism [G]	Inositol phosphate metabolism	Firmicutes	Phylum	Firmicutes						0.2314	0.0093	**
28	7291	MH0006_GL0176047	COG3063	Tfp pilus assembly protein PilF	Extracellular structures [W]	NAN	Parabacteroides	Genus	Bacteroidetes	Bacteroidia	Bacteroidales	Porphyromonadaceae	Parabacteroides		0.2290	0.0103	*
29	42974	O2.UC48-1_GL0053868	COG0059	Ketol-acid reductoisomerase	Coenzyme transport and metabolism [H]	Valine, leucine and isoleucine biosynthesis	Clostridiales	Order	Firmicutes	Clostridia	Clostridiales				0.2206	0.0004	***
30	112	206672.BL0597	COG0058	Glucan phosphorylase	Carbohydrate transport and metabolism [G]	Starch and sucrose metabolism	Bifidobacterium	Genus	Actinobacteria	Actinobacteria	Bifidobacteriales	Bifidobacteriaceae	Bifidobacterium		0.2153	0.0212	*
31	9235	MH0012_GL0199057	COG2885	Outer membrane protein OmpA and related peptidoglycan-associated (lipo)proteins	Cell wall/membrane/envelope biogenesis [M]	NAN	Bacteroidales	Order	Bacteroidetes	Bacteroidia	Bacteroidales				0.2151	0.0035	**
32	44853	T2D-105A_GL0107136	COG1629	Outer membrane receptor proteins, mostly Fe transport	Inorganic ion transport and metabolism [P]	NAN	Bacteroides	Genus	Bacteroidetes	Bacteroidia	Bacteroidales	Bacteroidaceae	Bacteroides		0.2116	0.0080	**
33	39110	O2.UC11-1_GL0068784	COG1978	Predicted RNase H-related nuclease YkuK, DUF458 family(	General function prediction only [R]	NAN	Dorea formicigenerans	Species	Firmicutes	Clostridia	Clostridiales	Lachnospiraceae	Dorea	Dorea formicigenerans	0.2099	0.0063	**
34	25966	MH0243_GL0019682	COG0149	Triosephosphate isomerase	Carbohydrate transport and metabolism [G]	Glycolysis / Gluconeogenesis	Ruminococcus bromii	Species	Firmicutes	Clostridia	Clostridiales	Ruminococcaceae	Ruminococcus	Ruminococcus bromii	0.2083	0.0452	*
35	18645	MH0131_GL0090479	COG1454	Alcohol dehydrogenase, class IV	Energy production and conversion [C]	Glycolysis / Gluconeogenesis	Collinsella aerofaciens	Species	Actinobacteria	Coriobacteriia	Coriobacteriales	Coriobacteriaceae	Collinsella	Collinsella aerofaciens	0.2019	0.0253	*
36	23826	MH0203_GL0134326			NAN	NAN	Dorea formicigenerans	Species	Firmicutes	Clostridia	Clostridiales	Lachnospiraceae	Dorea	Dorea formicigenerans	0.1949	0.0040	**
37	49280	V1.CD8-0-PN_GL0136266	COG1454	Alcohol dehydrogenase, class IV	Energy production and conversion [C]	Glycolysis / Gluconeogenesis	Clostridiales	Order	Firmicutes	Clostridia	Clostridiales				0.1941	0.0233	*
38	43725	O2.UC7-1_GL0015069	COG1653	ABC-type glycerol-3-phosphate transport system, periplasmic component	Carbohydrate transport and metabolism [G]	ABC transporters	Clostridiales	Order	Firmicutes	Clostridia	Clostridiales				0.1893	0.0263	*

39	46091	T2D-35A_GL0059333	COG0783	DNA-binding ferritin-like protein (oxidative damage protectant)	Defense mechanisms [V]	NAN	Bifidobacterium breve	Species	Actinobacteria	Actinobacteria	Bifidobacteriales	Bifidobacteriaceae	Bifidobacterium	Bifidobacterium breve	0.1874	0.0229	*
40	5459	MH0003_GL0013354	COG0334	Glutamate dehydrogenase/leucine dehydrogenase	Amino acid transport and metabolism [E]	Alanine, aspartate and glutamate metabolism	Bacteroides	Genus	Bacteroidetes	Bacteroidia	Bacteroidales	Bacteroidaceae	Bacteroides		0.1840	0.0108	*
41	14026	MH0073_GL0056444			NAN	Galactose metabolism	Ruminococcus bromii	Species	Firmicutes	Clostridia	Clostridiales	Ruminococcaceae	Ruminococcus	Ruminococcus bromii	0.181533883	0.013897574	*
42	5986	O2.UC48-0_GL0114931	COG0329	Dihydrodipicolinate synthase/N-acetylneuraminate lyase	Cell wall/membrane/envelope biogenesis [M]	Lysine biosynthesis	Ruminococcus bromii	Species	Firmicutes	Clostridia	Clostridiales	Ruminococcaceae	Ruminococcus	Ruminococcus bromii	0.178737774	0.023747567	*
43	33702	MH0406_GL0015896	COG1592	Rubrerythrin	Energy production and conversion [C]	NAN	Collinsella	Genus	Actinobacteria	Coriobacteriia	Coriobacteriales	Coriobacteriaceae	Collinsella		0.176580412	0.019125432	*
44	40481	O2.UC2-1_GL0059641	COG0206	Cell division GTPase FtsZ	Cell cycle control, cell division, chromosome partitioning [D]	Cell cycle - Caulobacter	Clostridiales	Order	Firmicutes	Clostridia	Clostridiales				-0.179992723	0.008051822	**
45	5041	MH0002_GL0050870	COG1048	Aconitase A	Energy production and conversion [C]	Citrate cycle (TCA cycle)	Clostridiales	Order	Firmicutes	Clostridia	Clostridiales				-0.181556636	0.008789114	**
46	18561	MH0131_GL0059554	COG0149	Triosephosphate isomerase	Carbohydrate transport and metabolism [G]	Glycolysis / Gluconeogenesis	Clostridiales	Order	Firmicutes	Clostridia	Clostridiales				-0.184183161	0.006174535	**
47	16923	MH0108_GL0062043	COG0092	Ribosomal protein S3	Translation, ribosomal structure and biogenesis [J]	Ribosome	Faecalibacterium prausnitzii	Species	Firmicutes	Clostridia	Clostridiales	Ruminococcaceae	Faecalibacterium	Faecalibacterium prausnitzii	-0.187086258	0.016436142	*
48	14632	MH0086_GL0050799	COG1456	CO dehydrogenase/acetyl-CoA synthase gamma subunit (corrinoid Fe-S protein)	Energy production and conversion [C]	Methane metabolism	Clostridiales	Order	Firmicutes	Clostridia	Clostridiales				-0.189918364	0.006557014	**
49	52494	V1.UC40-0_GL0007441	COG0057	Glyceraldehyde-3-phosphate dehydrogenase/erythrose-4-phosphate dehydrogenase	Carbohydrate transport and metabolism [G]	Glycolysis / Gluconeogenesis	Anaerostipes caccae	Species	Firmicutes	Clostridia	Clostridiales	Lachnospiraceae	Anaerostipes	Anaerostipes caccae	-0.190544641	0.022222845	*
50	4871	MH0002_GL0038347	COG0297	Glycogen synthase	Carbohydrate transport and metabolism [G]	Galactose metabolism	Ruminococcaceae	Species	Firmicutes	Clostridia	Clostridiales	Ruminococcaceae	Subdoligranulum	Subdoligranulum variabile	-0.191849554	0.011724592	*
51	4632	MH0002_GL0018942	COG0085	DNA-directed RNA polymerase, beta subunit/140 kD subunit	Transcription [K]	Purine metabolism	Bacteria	Species	Actinobacteria	Coriobacteriia	Coriobacteriales	Coriobacteriaceae	Coriobacterium	Coriobacterium glomerans	-0.192341616	0.000877222	***
52	15026	MH0088_GL0034073	COG0480	Translation elongation factor EF-G, a GTPase	Translation, ribosomal structure and biogenesis [J]	NAN	Clostridiales	Order	Firmicutes	Clostridia	Clostridiales				-0.196489027	0.007825366	**
53	284	367928.BAD_0378	COG0588	Phosphoglycerate mutase (BPG-dependent)	Carbohydrate transport and metabolism [G]	Glycolysis / Gluconeogenesis	Bifidobacterium	Genus	Actinobacteria	Actinobacteria	Bifidobacteriales	Bifidobacteriaceae	Bifidobacterium		-0.196748023	0.02666976	*
54	8579	MH0012_GL0011046	COG3842	ABC-type Fe3+/spermidine/putrescine transport systems, ATPase components	Amino acid transport and metabolism [E]	ABC transporters	Ruminococcaceae	Family	Firmicutes	Clostridia	Clostridiales	Ruminococcaceae			-0.205258005	0.007732047	**
55	37103	N079A_GL0025412	COG0049	Ribosomal protein S7	Translation, ribosomal structure and biogenesis [J]	Ribosome	Faecalibacterium prausnitzii	Species	Firmicutes	Clostridia	Clostridiales	Ruminococcaceae	Faecalibacterium	Faecalibacterium prausnitzii	-0.206696233	0.000399391	***
56	18636	MH0131_GL0088691	COG1080	Phosphoenolpyruvate-protein kinase (PTS system EI component in bacteria)	Carbohydrate transport and metabolism [G]	Pyruvate metabolism	Coprococcus	Genus	Firmicutes	Clostridia	Clostridiales	Lachnospiraceae	Coprococcus		-0.209374644	0.01817082	*
57	42601	O2.UC46-1_GL0109045	COG1145	Ferredoxin	Energy production and conversion [C]	Glycolysis / Gluconeogenesis	Faecalibacterium prausnitzii	Species	Firmicutes	Clostridia	Clostridiales	Ruminococcaceae	Faecalibacterium	Faecalibacterium prausnitzii	-0.210443462	0.012427576	*
58	42804	O2.UC48-0_GL0107610	COG3842	ABC-type Fe3+/spermidine/putrescine transport systems, ATPase components	Amino acid transport and metabolism [E]	ABC transporters	Lachnospiraceae	Family	Firmicutes	Clostridia	Clostridiales	Lachnospiraceae			-0.212894975	0.025839277	*
59	4746	MH0002_GL0027835	COG3968	Glutamine synthetase type III	Amino acid transport and metabolism [E]	Alanine, aspartate and glutamate metabolism	Ruminococcaceae	Species	Firmicutes	Clostridia	Clostridiales	Ruminococcaceae	Subdoligranulum	Subdoligranulum variabile	-0.215527676	0.034350749	*
60	34380	MH0419_GL0081674	COG0228	Ribosomal protein S16	Translation, ribosomal structure and biogenesis [J]	Ribosome	Bifidobacterium	Genus	Actinobacteria	Actinobacteria	Bifidobacteriales	Bifidobacteriaceae	Bifidobacterium		-0.216944462	0.002747718	**
61	31211	MH0356_GL0130221	COG0056	FoF1-type ATP synthase, alpha subunit	Energy production and conversion [C]	Oxidative phosphorylation	Bifidobacterium	Genus	Actinobacteria	Actinobacteria	Bifidobacteriales	Bifidobacteriaceae	Bifidobacterium		-0.217273962	0.006975824	**
62	52954	V1.UC51-4_GL0052281	COG1879	ABC-type sugar transport system, periplasmic component, contains N-terminal xre family HTH domain	Carbohydrate transport and metabolism [G]	ABC transporters	Clostridiales	Order	Firmicutes	Clostridia	Clostridiales				-0.221944669	0.007338031	**
63	24978	MH0227_GL0147089			NAN	NAN	Subdoligranulum variabile	Species	Firmicutes	Clostridia	Clostridiales	Ruminococcaceae	Subdoligranulum	Subdoligranulum variabile	-0.222662424	0.003146434	**
64	17010	MH0110_GL0025297	COG1080	Phosphoenolpyruvate-protein kinase (PTS system EI component in bacteria)	Carbohydrate transport and metabolism [G]	Pyruvate metabolism	Clostridiales	Order	Firmicutes	Clostridia	Clostridiales				-0.223072339	0.011550857	*
65	4512	MH0002_GL0008497	COG2025	Electron transfer flavoprotein, alpha subunit	Energy production and conversion [C]	Nitrogen metabolism	Subdoligranulum variabile	Species	Firmicutes	Clostridia	Clostridiales	Ruminococcaceae	Subdoligranulum	Subdoligranulum variabile	-0.226721169	0.008450267	**
66	25756	MH0239_GL0018928	COG1145	Ferredoxin	Energy production and conversion [C]	Glycolysis / Gluconeogenesis	Clostridiales	Order	Firmicutes	Clostridia	Clostridiales				-0.227777225	0.004064887	**
67	5421	MH0142_GL0051559	COG3033	Tryptophanase	Amino acid transport and metabolism [E]	Tyrosine metabolism	Firmicutes	Phylum	Firmicutes						-0.233622406	0.003743153	**
68	4272	MH0001_GL0015313	COG1145	Ferredoxin	Energy production and conversion [C]	Glycolysis / Gluconeogenesis	Roseburia faecis	Species	Firmicutes	Clostridia	Clostridiales	Lachnospiraceae	Roseburia	Roseburia faecis	-0.242071665	0.009329289	**
69	45165	T2D-133A_GL0021831	COG1653	ABC-type glycerol-3-phosphate transport system, periplasmic component	Carbohydrate transport and metabolism [G]	NAN	Bifidobacterium	Genus	Actinobacteria	Actinobacteria	Bifidobacteriales	Bifidobacteriaceae	Bifidobacterium		-0.247393184	0.012884684	*
70	11241	MH0032_GL0024527	COG0090	Ribosomal protein L2	Translation, ribosomal structure and biogenesis [J]	Ribosome	Dialister succinatiphilus	Species	Firmicutes	Negativicutes	Veillonellales	Veillonellaceae	Dialister	Dialister succinatiphilus	-0.252657558	0.000649382	***
71	41419	O2.UC31-1_GL0115264	COG0085	DNA-directed RNA polymerase, beta subunit/140 kD subunit	Transcription [K]	Purine metabolism	Clostridiales	Order	Firmicutes	Clostridia	Clostridiales				-0.265025627	0.000587996	***
72	17672	MH0131_GL0013416	COG1592	Rubrerythrin	Energy production and conversion [C]	NAN	Subdoligranulum variabile	Species	Firmicutes	Clostridia	Clostridiales	Ruminococcaceae	Subdoligranulum	Subdoligranulum variabile	-0.266760182	0.021855477	*
73	44660	SZEY-69A_GL0070022	COG5492	Uncharacterized conserved protein YjdB, contains Ig-like domain	General function prediction only [R]	NAN	Symbiobacterium thermophilum	Species	Firmicutes	Clostridia	Clostridiales	Symbiobacteriaceae	Symbiobacterium	Symbiobacterium thermophilum	-0.270000312	0.00835433	**
74	5043	MH0002_GL0050951	COG1145	Ferredoxin	Energy production and conversion [C]	Glycolysis / Gluconeogenesis	Ruminococcaceae	Species	Firmicutes	Clostridia	Clostridiales	Ruminococcaceae	Faecalibacterium	Faecalibacterium prausnitzii	-0.280267717	0.010006572	*
75	5239	MH0002_GL0070693	COG2182	Maltose-binding periplasmic protein MalE	Carbohydrate transport and metabolism [G]	ABC transporters	Clostridiales bacterium CHKCI001	Species	Firmicutes	Clostridia	Clostridiales			Clostridiales bacterium CHKCI001	-0.294359949	0.007489068	**
76	12697	MH0055_GL0031824	COG0050	Translation elongation factor EF-Tu, a GTPase	Translation, ribosomal structure and biogenesis [J]	Plant-pathogen interaction	Faecalibacterium prausnitzii	Species	Firmicutes	Clostridia	Clostridiales	Ruminococcaceae	Faecalibacterium	Faecalibacterium prausnitzii	-0.29571511	0.025487098	*

**77**	42060	O2.UC40-1_GL0122610	COG1882	Pyruvate-formate lyase	Energy production and conversion [C]	Pyruvate metabolism	Clostridiales bacterium KLE1615	Species	Firmicutes	Clostridia	Clostridiales			Clostridiales bacterium KLE1615	-0.305746247	0.000751388	***
**78**	7922	MH0010_GL0016261	COG0539	Ribosomal protein S1	Translation, ribosomal structure and biogenesis [J]	Terpenoid backbone biosynthesis	Faecalibacterium prausnitzii	Species	Firmicutes	Clostridia	Clostridiales	Ruminococcaceae	Faecalibacterium	Faecalibacterium prausnitzii	-0.314208299	0.010734841	*
**79**	4427	MH0002_GL0001931	COG0480	Translation elongation factor EF-G, a GTPase	Translation, ribosomal structure and biogenesis [J]	NAN	Clostridiales	Species	Firmicutes	Clostridia	Clostridiales	Clostridiaceae	Butyricicoccus	Butyricicoccus pullicaecorum	-0.320181384	0.000937946	***
**80**	11766	MH0041_GL0040403	COG1866	Phosphoenolpyruvate carboxykinase, ATP-dependent	Energy production and conversion [C]	Glycolysis / Gluconeogenesis	Roseburia faecis	Species	Firmicutes	Clostridia	Clostridiales	Lachnospiraceae	Roseburia	Roseburia faecis	-0.325471428	7.34119E-05	***
**81**	4426	MH0002_GL0001930	COG0050	Translation elongation factor EF-Tu, a GTPase	Translation, ribosomal structure and biogenesis [J]	Plant-pathogen interaction	Clostridiales	Species	Firmicutes	Clostridia	Clostridiales	Ruminococcaceae	Subdoligranulum	Subdoligranulum variabile	-0.336413211	3.16659E-05	***
**82**	8811	MH0012_GL0079061	COG0282	Acetate kinase	Energy production and conversion [C]	Taurine and hypotaurine metabolism	Clostridium pasteurianum	Species	Firmicutes	Clostridia	Clostridiales	Clostridiaceae	Clostridium	Clostridium pasteurianum	-0.345252405	0.000112928	***
**83**	20073	MH0149_GL0094229	COG1080	Phosphoenolpyruvate-protein kinase (PTS system EI component in bacteria)	Carbohydrate transport and metabolism [G]	Pyruvate metabolism	Faecalibacterium prausnitzii	Species	Firmicutes	Clostridia	Clostridiales	Ruminococcaceae	Faecalibacterium	Faecalibacterium prausnitzii	-0.357182122	0.008884528	**
**84**	28712	MH0293_GL0090794	COG1080	Phosphoenolpyruvate-protein kinase (PTS system EI component in bacteria)	Carbohydrate transport and metabolism [G]	Pyruvate metabolism	Clostridiales	Order	Firmicutes	Clostridia	Clostridiales				-0.401036015	0.005392868	**
**85**	939	515619.EUBREC_1472	COG1145	Ferredoxin	Energy production and conversion [C]	Glycolysis / Gluconeogenesis	Clostridiales	Order	Firmicutes	Clostridia	Clostridiales				-0.414936044	0.008622791	**
**86**	31674	MH0364_GL0101039	COG0057	Glyceraldehyde-3-phosphate dehydrogenase/erythrose-4-phosphate dehydrogenase	Carbohydrate transport and metabolism [G]	Glycolysis / Gluconeogenesis	Ruminococcus bicirculans	Species	Firmicutes	Clostridia	Clostridiales	Ruminococcaceae	Ruminococcus	Ruminococcus bicirculans	-0.419762091	0.000366239	***
**87**	17690	MH0122_GL0011708	COG0050	Translation elongation factor EF-Tu, a GTPase	Translation, ribosomal structure and biogenesis [J]	Plant-pathogen interaction	Subdoligranulum variabile	Species	Firmicutes	Clostridia	Clostridiales	Ruminococcaceae	Subdoligranulum	Subdoligranulum variabile	-0.434455982	0.000173529	***
**88**	4915	MH0002_GL0040735	COG3957	Phosphoketolase	Carbohydrate transport and metabolism [G]	Pentose phosphate pathway	Mycobacterium bohemicum	Species	Actinobacteria	Actinobacteria	Corynebacteriales	Mycobacteriaceae	Mycobacterium	Mycobacterium bohemicum	-0.45247304	4.35888E-05	***
**89**	4916	MH0002_GL0040736	COG2407	L-fucose isomerase or related protein	Carbohydrate transport and metabolism [G]	Fructose and mannose metabolism	Subdoligranulum variabile	Species	Firmicutes	Clostridia	Clostridiales	Ruminococcaceae	Subdoligranulum	Subdoligranulum variabile	-0.509915581	2.28399E-05	***
