# Supplementary material for: Williams–Beuren syndrome shapes the gut microbiota metaproteome
Source: Sci Rep. 2023 Nov 3;13:18963. doi: 10.1038/s41598-023-46052-9 (PMC10624682; doi:10.1038/s41598-023-46052-9)
Supplement: Supplementary file 4 — Supplementary File 3. [file 41598_2023_46052_MOESM4_ESM.pdf]

42 identified human PGs

N	Protein Group ID	Leading razor protein accession	Leading razor protein entry name	Protein names	Gene names	KEGG ID	Log_{10} (WBS/CTRL)	t-test p-value WBS/CTRL	Significance WBS/CTRL
1	37877	P62805	H4_HUMAN	Histone H4	H4C1 H4/A H4FA HIST1H4A; H4C2 H4/I H4FI HIST1H4B; H4C3 H4/G H4FG HIST1H4C; H4C4 H4/B H4FB HIST1H4D; H4C5 H4/J H4FJ HIST1H4E; H4C6 H4/C H4FC HIST1H4F; H4C8 H4/H H4FH HIST1H4H; H4C9 H4/M H4FM HIST1H4I; H4C11 H4/E H4FE HIST1H4J; H4C12 H4/D H4FD HIST1H4K; H4C13 H4/K H4FK HIST1H4L; H4C14 H4/N H4F2 H4FN HIST2H4 HIST2H4A; H4C15 H4/O H4FO HIST2H4B; H4-16 HIST4H4	hsa:121504; hsa:554313, hsa:8294; hsa:8359; hsa:8360; hsa:8361; hsa:8362; hsa:8363; hsa:8364; hsa:8365; hsa:8366; hsa:8367; hsa:8368; hsa:8370	-0.1197	0.0728	
2	43989	O60635	TSN1_HUMAN	Tetraspanin-1 (Tspan-1) (Tetraspan NET-1) (Tetraspanin TM4-C)	TSPAN1	hsa:10103	0.0621	0.4704	
3	43995	O95497	VNN1_HUMAN	Pantetheinase (EC 3.5.1.92) (Pantetheine hydrolase) (Tiff66) (Vascular non-inflammatory molecule 1) (Vanin-1)	VNN1	hsa:8876	0.2735	0.0024	**
4	44001	P01008	ANT3_HUMAN	Antithrombin-III (ATIII) (Serpin C1)	SERPINC1 AT3 PRO0309	hsa:462	0.1105	0.0643	
5	44017	P04054	PA21B_HUMAN	Phospholipase A2 (EC 3.1.1.4) (Group IB phospholipase A2) (Phosphatidylcholine 2-acylhydrolase 1B)	PLA2G1B PLA2 PLA2A PPLA2	hsa:5319	0.1489	0.0612	
6	44020	P04118	COL_HUMAN	Colipase	CLPS	hsa:1208	-0.0560	0.5493	
7	44022	P05090	APOD_HUMAN	Apolipoprotein D (Apo-D) (ApoD)	APOD	hsa:347	0.0855	0.2079	
8	44024	P05109	S10A8_HUMAN	Protein S100-A8 (Calgranulin-A) (Calprotectin L1L subunit) (Cystic fibrosis antigen) (CFAG) (Leukocyte L1 complex light chain) (Migration inhibitory factor-related protein 8) (MRP-8) (p8) (S100 calcium-binding protein A8) (Urinary stone protein band A)	S100A8 CAGA CFAG MRP8	hsa:6279	0.3493	0.0031	**
9	44025	P05164	PERM_HUMAN	Myeloperoxidase (MPO) (EC 1.11.2.2) [Cleaved into: Myeloperoxidase; 89 kDa myeloperoxidase; 84 kDa myeloperoxidase; Myeloperoxidase light chain; Myeloperoxidase heavy chain]	MPO	hsa:4353	0.0968	0.4656	
10	44026	P06702	S10A9_HUMAN	Protein S100-A9 (Calgranulin-B) (Calprotectin L1H subunit) (Leukocyte L1 complex heavy chain) (Migration inhibitory factor-related protein 14) (MRP-14) (p14) (S100 calcium-binding protein A9)	S100A9 CAGB CFAG MRP14	hsa:6280	0.1907	0.1237	
11	44029	P06748	NPM_HUMAN	Nucleophosmin (NPM) (Nucleolar phosphoprotein B23) (Nucleolar protein NO38) (Numatrin)	NPM1 NPM	hsa:4869	0.0812	0.1346	
12	44031	P07355	ANXA2_HUMAN	Annexin A2 (Annexin II) (Annexin-2) (Calpactin I heavy chain) (Calpactin-1 heavy chain) (Chromobindin-8) (Lipocortin II) (Placental anticoagulant protein IV) (PAP-IV) (Protein I) (p36)	ANXA2 ANX2 ANX2L4 CAL1H LPC2D	hsa:302	0.2710	0.0186	*
13	44033	P07478	TRY2_HUMAN	Trypsin-2 (EC 3.4.21.4) (Anionic trypsinogen) (Serine protease 2) (Trypsin II)	PRSS2 TRY2 TRYP2	hsa:5645	0.1646	0.0769	
14	44039	P08473	NEP_HUMAN	Neprilysin (EC 3.4.24.11) (Atriopeptidase) (Common acute lymphocytic leukemia antigen) (CALLA) (Enkephalinase) (Neutral endopeptidase 24.11) (NEP) (Neutral endopeptidase) (Skin fibroblast elastase) (SFE) (CD antigen CD10)	MME EPN	hsa:4311	0.2103	0.0970	
15	44043	P09525	ANXA4_HUMAN	Annexin A4 (35-beta calcimedin) (Annexin IV) (Annexin-4) (Carbohydrate-binding protein p33/p41) (Chromobindin-4) (Endonexin I) (Lipocortin IV) (P32.5) (PP4-X) (Placental anticoagulant protein II) (PAP-II) (Protein II)	ANXA4 ANX4	hsa:307	0.2391	0.0150	*
16	44048	P0DUB6	AMY1A_HUMAN	Alpha-amylase 1A (EC 3.2.1.1) (1,4-alpha-D-glucan glucanohydrolase 1) (Salivary alpha-amylase) 1B-1C	AMY1A AMY1	hsa:276; hsa:277; hsa:278	0.0438	0.6573	
17	44050	P11678	PERE_HUMAN	Eosinophil peroxidase (EPO) (EC 1.11.1.7) [Cleaved into: Eosinophil peroxidase light chain; Eosinophil peroxidase heavy chain]	EPX EPER EPO EPP	hsa:8288	-0.2282	0.0681	
18	44056	P12882	MYH1_HUMAN	Myosin-1 (Myosin heavy chain 1) (Myosin heavy chain 2x) (MyHC-2x) (Myosin heavy chain IIx/d) (MyHC-IIx/d) (Myosin heavy chain, skeletal muscle, adult 1)	MYH1	hsa:4619	0.1499	0.2278	
19	44057	P12883	MYH7_HUMAN	Myosin-7 (Myosin heavy chain 7) (Myosin heavy chain slow isoform) (MyHC-slow) (Myosin heavy chain, cardiac muscle beta isoform) (MyHC-beta)	MYH7 MYHCB	hsa:4625	0.0817	0.2195	
20	44063	P15085	CBPA1_HUMAN	Carboxypeptidase A1 (EC 3.4.17.1)	CPA1 CPA	hsa:1357	0.1817	0.1194	
21	44064	P15086	CBPB1_HUMAN	Carboxypeptidase B (EC 3.4.17.2) (Pancreas-specific protein) (PASP)	CPB1 CPB PCPB	hsa:1360	0.0864	0.3664	
22	44065	P15144	AMPN_HUMAN	Aminopeptidase N (AP-N) (hAPN) (EC 3.4.11.2) (Alanyl aminopeptidase) (Aminopeptidase M) (AP-M) (Microsomal aminopeptidase) (Myeloid plasma membrane glycoprotein CD13) (gp150) (CD antigen CD13)	ANPEP APN CD13 PEPN	hsa:290	0.4063	0.0074	**
23	44069	P16444	DPEP1_HUMAN	Dipeptidase 1 (EC 3.4.13.19) (Beta-lactamase) (EC 3.5.2.6) (Dehydropeptidase-I) (Microsomal dipeptidase) (Renal dipeptidase) (hRDP)	DPEP1 MDP RDP	hsa:1800	0.2003	0.0258	*
24	44072	P19961	AMY2B_HUMAN	Alpha-amylase 2B (EC 3.2.1.1) (1,4-alpha-D-glucan glucanohydrolase 2B) (Carcinoid alpha-amylase)	AMY2B	hsa:280	0.0726	0.5002	
25	44092	P30626	SORCN_HUMAN	Sorcin (22 kDa protein) (CP-22) (CP22) (V19)	SRI	hsa:6717	0.0237	0.6535	
26	44097	P35237	SPB6_HUMAN	Serpin B6 (Cytoplasmic antiproteinase) (CAP) (Peptidase inhibitor 6) (PI-6) (Placental thrombin inhibitor)	SERPINB6 PI6 PTI	hsa:5269	0.1319	0.0283	*
27	44102	P48052	CBPA2_HUMAN	Carboxypeptidase A2 (EC 3.4.17.15)	CPA2	hsa:1358	0.1278	0.2519	
28	44105	P50995	ANX11_HUMAN	Annexin A11 (56 kDa autoantigen) (Annexin XI) (Annexin-11) (Calcyclin-associated annexin 50) (CAP-50)	ANXA11 ANX11	hsa:311	0.0872	0.0822	
29	44107	P56470	LEG4_HUMAN	Galectin-4 (Gal-4) (Antigen NY-CO-27) (L-36 lactose-binding protein) (L36LBP) (Lactose-binding lectin 4)	LGALS4	hsa:3960	0.0537	0.5759	
30	44110	P61626	LYSC_HUMAN	Lysozyme C (EC 3.2.1.17) (1,4-beta-N-acetylmuramidase C)	LYZ LZM	hsa:4069	-0.0018	0.9855	
31	44111	P63261	ACTG_HUMAN	Actin, cytoplasmic 2 (Gamma-actin) [Cleaved into: Actin, cytoplasmic 2, N-terminally processed]	ACTG1 ACTG	hsa:71	-0.0297	0.7951	
32	44115	P98088	MUC5A_HUMAN	Mucin-5AC (MUC-5AC) (Gastric mucin) (Major airway glycoprotein) (Mucin-5 subtype AC, tracheobronchial) (Tracheobronchial mucin) (TBM)	MUC5AC MUC5	hsa:4586	0.1242	0.0580	

**33**	44121	Q02817	MUC2_HUMAN	Mucin-2 (MUC-2) (Intestinal mucin-2)	MUC2 SMUC		0.0390	0.6415	
**34**	44123	Q03403	TFF2_HUMAN	Trefoil factor 2 (Spasmolysin) (Spasmolytic polypeptide) (SP)	TFF2 SML1	hsa:7032	0.2216	0.0449	*
**35**	44134	Q6GPI1	CTRB2_HUMAN	Chymotrypsinogen B2 (EC 3.4.21.1) [Cleaved into: Chymotrypsin B2 chain A; Chymotrypsin B2 chain B; Chymotrypsin B2 chain C]	CTRB2	hsa:440387	0.2517	0.0521	
**36**	44138	Q8IVL8	CBPO_HUMAN	Carboxypeptidase O (CPO) (EC 3.4.17.-)	CPO	hsa:130749	0.0306	0.6071	
**37**	44142	Q8WWA0	ITLN1_HUMAN	Intelectin-1 (ITLN-1) (Endothelial lectin HL-1) (Galactofuranose-binding lectin) (Intestinal lactoferrin receptor) (Omentin)	ITLN1 INTL ITLN LFR UNQ640/PRO1270	hsa:55600	-0.0483	0.5714	
**38**	44143	Q8WWU7	ITLN2_HUMAN	Intelectin-2 (Endothelial lectin HL-2)	ITLN2 UNQ2789/PRO7179	hsa:142683	0.0268	0.7334	
**39**	44144	Q8WZ42	TITIN_HUMAN	Titin (EC 2.7.11.1) (Connectin) (Rhabdomyosarcoma antigen MU-RMS-40.14)	TTN	hsa:7273	-0.0259	0.7100	
**40**	44150	Q99895	CTRC_HUMAN	Chymotrypsin-C (EC 3.4.21.2) (Caldecrin)	CTRC CLCR	hsa:11330	-0.0637	0.4967	
**41**	44153	Q9H3R2	MUC13_HUMAN	Mucin-13 (MUC-13) (Down-regulated in colon cancer 1)	MUC13 DRCC1 RECC UNQ6194/PRO20221	hsa:56667	0.1667	0.0539	
**42**	44158	Q9NR71	ASAH2_HUMAN	Neutral ceramidase (N-CDase) (NCDase) (EC 3.5.1.-) (EC 3.5.1.23) (Acylsphingosine deacylase 2) (BCDase) (LCDase) (hCD) (N-acylsphingosine amidohydrolase 2) (Non-lysosomal ceramidase) [Cleaved into: Neutral ceramidase soluble form]	ASAH2 HNAC1	hsa:56624	0.0088	0.9097	

PGs with loadings coefficient on PC1 of PCA > |0.09| on which MANOVA test was performed

N	Protein Group ID	Leading razor protein accession	Leading razor protein entry name	Protein names	Gene names	KEGG ID	Log_{10} (WBS/CTRL)	t-test p-value WBS/CTRL	Significance WBS/CTRL
1	43989	O60635	TSN1_HUMAN	Tetraspanin-1 (Tspan-1) (Tetraspan NET-1) (Tetraspanin TM4-C)	TSPAN1	hsa:10103	0.0621	0.4704	
2	43995	O95497	VNN1_HUMAN	Pantetheinase (EC 3.5.1.92) (Pantetheine hydrolase) (Tiff66) (Vascular non-inflammatory molecule 1) (Vanin-1)	VNN1	hsa:8876	0.2735	0.0024	**
3	44017	P04054	PA21B_HUMAN	Phospholipase A2 (EC 3.1.1.4) (Group IB phospholipase A2) (Phosphatidylcholine 2-acylhydrolase 1B)	PLA2G1B PLA2 PLA2A PPLA2	hsa:5319	0.1489	0.0612	
4	44020	P04118	COL_HUMAN	Colipase	CLPS	hsa:1208	-0.0560	0.5493	
5	44024	P05109	S10A8_HUMAN	Protein S100-A8 (Calgranulin-A) (Calprotectin L1L subunit) (Cystic fibrosis antigen) (CFAG) (Leukocyte L1 complex light chain) (Migration inhibitory factor-related protein 8) (MRP-8) (p8) (S100 calcium-binding protein A8) (Urinary stone protein band A)	S100A8 CAGA CFAG MRP8	hsa:6279	0.3493	0.0031	**
6	44025	P05164	PERM_HUMAN	Myeloperoxidase (MPO) (EC 1.11.2.2) [Cleaved into: Myeloperoxidase; 89 kDa myeloperoxidase; 84 kDa myeloperoxidase; Myeloperoxidase light chain; Myeloperoxidase heavy chain]	MPO	hsa:4353	0.0968	0.4656	
7	44026	P06702	S10A9_HUMAN	Protein S100-A9 (Calgranulin-B) (Calprotectin L1H subunit) (Leukocyte L1 complex heavy chain) (Migration inhibitory factor-related protein 14) (MRP-14) (p14) (S100 calcium-binding protein A9)	S100A9 CAGB CFAG MRP14	hsa:6280	0.1907	0.1237	
8	44031	P07355	ANXA2_HUMAN	Annexin A2 (Annexin II) (Annexin-2) (Calpactin I heavy chain) (Calpactin-1 heavy chain) (Chromobindin-8) (Lipocortin II) (Placental anticoagulant protein IV) (PAP-IV) (Protein I) (p36)	ANXA2 ANX2 ANX2L4 CAL1H LPC2D	hsa:302	0.2710	0.0186	*
9	44033	P07478	TRY2_HUMAN	Trypsin-2 (EC 3.4.21.4) (Anionic trypsinogen) (Serine protease 2) (Trypsin II)	PRSS2 TRY2 TRYP2	hsa:5645	0.1646	0.0769	
10	44039	P08473	NEP_HUMAN	Neprilysin (EC 3.4.24.11) (Atriopeptidase) (Common acute lymphocytic leukemia antigen) (CALLA) (Enkephalinase) (Neutral endopeptidase 24.11) (NEP) (Neutral endopeptidase) (Skin fibroblast elastase) (SFE) (CD antigen CD10)	MME EPN	hsa:4311	0.2103	0.0970	
11	44043	P09525	ANXA4_HUMAN	Annexin A4 (35-beta calcimedin) (Annexin IV) (Annexin-4) (Carbohydrate-binding protein p33/p41) (Chromobindin-4) (Endonexin I) (Lipocortin IV) (P32.5) (PP4-X) (Placental anticoagulant protein II) (PAP-II) (Protein II)	ANXA4 ANX4	hsa:307	0.2391	0.0150	*
12	44048	P0DUB6	AMY1A_HUMAN	Alpha-amylase 1A (EC 3.2.1.1) (1,4-alpha-D-glucan glucanohydrolase 1) (Salivary alpha-amylase) 1B-1C	AMY1A AMY1	hsa:276; hsa:277; hsa:278	0.0438	0.6573	
13	44050	P11678	PERE_HUMAN	Eosinophil peroxidase (EPO) (EC 1.11.1.7) [Cleaved into: Eosinophil peroxidase light chain; Eosinophil peroxidase heavy chain]	EPX EPER EPO EPP	hsa:8288	-0.2282	0.0681	
14	44063	P15085	CBPA1_HUMAN	Carboxypeptidase A1 (EC 3.4.17.1)	CPA1 CPA	hsa:1357	0.1817	0.1194	
15	44064	P15086	CBPB1_HUMAN	Carboxypeptidase B (EC 3.4.17.2) (Pancreas-specific protein) (PASP)	CPB1 CPB PCPB	hsa:1360	0.0864	0.3664	
16	44065	P15144	AMPN_HUMAN	Aminopeptidase N (AP-N) (hAPN) (EC 3.4.11.2) (Alanyl aminopeptidase) (Aminopeptidase M) (AP-M) (Microsomal aminopeptidase) (Myeloid plasma membrane glycoprotein CD13) (gp150) (CD antigen CD13)	ANPEP APN CD13 PEPN	hsa:290	0.4063	0.0074	**
17	44069	P16444	DPEP1_HUMAN	Dipeptidase 1 (EC 3.4.13.19) (Beta-lactamase) (EC 3.5.2.6) (Dehydropeptidase-I) (Microsomal dipeptidase) (Renal dipeptidase) (hRDP)	DPEP1 MDP RDP	hsa:1800	0.2003	0.0258	*
18	44072	P19961	AMY2B_HUMAN	Alpha-amylase 2B (EC 3.2.1.1) (1,4-alpha-D-glucan glucanohydrolase 2B) (Carcinoid alpha-amylase)	AMY2B	hsa:280	0.0726	0.5002	
19	44102	P48052	CBPA2_HUMAN	Carboxypeptidase A2 (EC 3.4.17.15)	CPA2	hsa:1358	0.1278	0.2519	
20	44105	P50995	ANX11_HUMAN	Annexin A11 (56 kDa autoantigen) (Annexin XI) (Annexin-11) (Calcyclin-associated annexin 50) (CAP-50)	ANXA11 ANX11	hsa:311	0.0872	0.0822	
21	44107	P56470	LEG4_HUMAN	Galectin-4 (Gal-4) (Antigen NY-CO-27) (L-36 lactose-binding protein) (L36LBP) (Lactose-binding lectin 4)	LGALS4	hsa:3960	0.0537	0.5759	
22	44110	P61626	LYSC_HUMAN	Lysozyme C (EC 3.2.1.17) (1,4-beta-N-acetylmuramidase C)	LYZ LZM	hsa:4069	-0.0018	0.9855	

**23**	44111	P63261	ACTG_HUMAN	Actin, cytoplasmic 2 (Gamma-actin) [Cleaved into: Actin, cytoplasmic 2, N-terminally processed]	ACTG1 ACTG	hsa:71	-0.0297	0.7951	
**24**	44123	Q03403	TFF2_HUMAN	Trefoil factor 2 (Spasmolysin) (Spasmolytic polypeptide) (SP)	TFF2 SML1	hsa:7032	0.2216	0.0449	*
**25**	44134	Q6GPI1	CTRB2_HUMAN	Chymotrypsinogen B2 (EC 3.4.21.1) [Cleaved into: Chymotrypsin B2 chain A; Chymotrypsin B2 chain B; Chymotrypsin B2 chain C]	CTRB2	hsa:440387	0.2517	0.0521	
**26**	44142	Q8WWA0	ITLN1_HUMAN	Intelectin-1 (ITLN-1) (Endothelial lectin HL-1) (Galactofuranose-binding lectin) (Intestinal lactoferrin receptor) (Omentin)	ITLN1 INTL ITLN LFR UNQ640/PRO1270	hsa:55600	-0.0483	0.5714	
**27**	44143	Q8WWU7	ITLN2_HUMAN	Intelectin-2 (Endothelial lectin HL-2)	ITLN2 UNQ2789/PRO7179	hsa:142683	0.0268	0.7334	
**28**	44150	Q99895	CTRC_HUMAN	Chymotrypsin-C (EC 3.4.21.2) (Caldecrin)	CTRC CLCR	hsa:11330	-0.0637	0.4967	
**29**	44153	Q9H3R2	MUC13_HUMAN	Mucin-13 (MUC-13) (Down-regulated in colon cancer 1)	MUC13 DRCC1 RECC UNQ6194/PRO20221	hsa:56667	0.1667	0.0539	

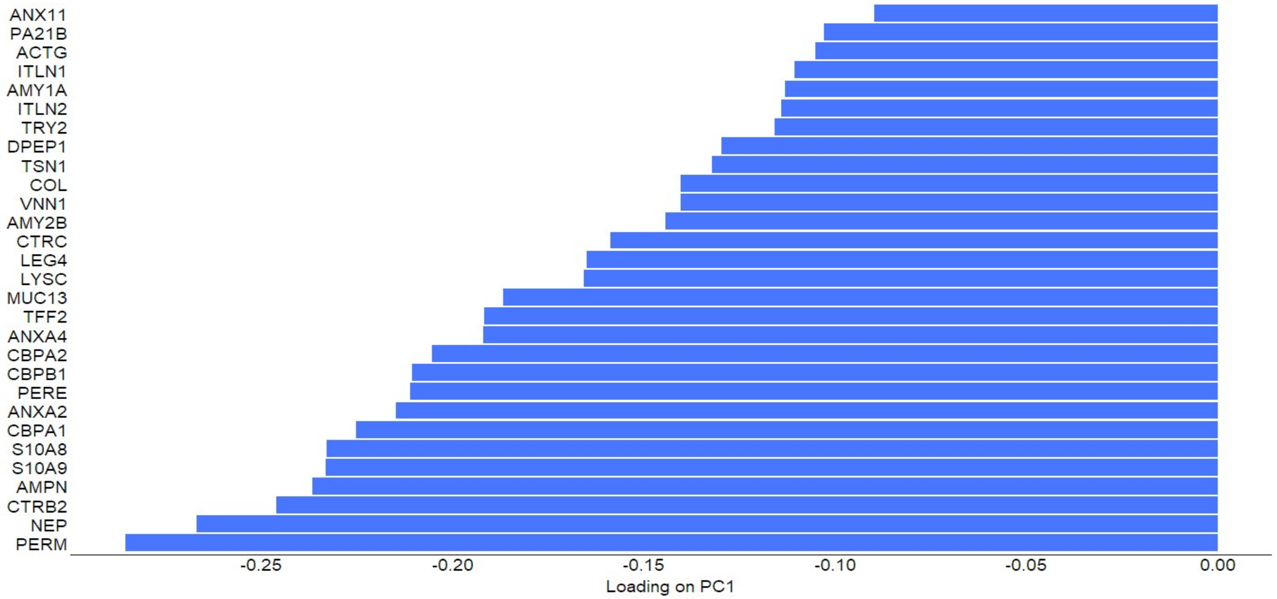

PGs with VIP > 1

N	Protein Group ID	Leading razor protein accession	Leading razor protein entry name	Protein names	Gene names	KEGG ID	Log_{10} (WBS/CTRL)	t-test p-value WBS/CTRL	Significance WBS/CTRL
1	37877	P62805	H4_HUMAN	Histone H4	H4C1 H4/A H4FA HIST1H4A; H4C2 H4/I H4FI HIST1H4B; H4C3 H4/G H4FG HIST1H4C; H4C4 H4/B H4FB HIST1H4D; H4C5 H4/J H4FJ HIST1H4E; H4C6 H4/C H4FC HIST1H4F; H4C8 H4/H H4FH HIST1H4H; H4C9 H4/M H4FM HIST1H4I; H4C11 H4/E H4FE HIST1H4J; H4C12 H4/D H4FD HIST1H4K; H4C13 H4/K H4FK HIST1H4L; H4C14 H4/N H4F2 H4FN HIST2H4 HIST2H4A; H4C15 H4/O H4FO HIST2H4B; H4-16 HIST4H4	hsa:121504; hsa:554313, hsa:8294; hsa:8359; hsa:8360; hsa:8361; hsa:8362; hsa:8363; hsa:8364; hsa:8365; hsa:8366; hsa:8367; hsa:8368; hsa:8370	-0.1197	0.0728	
2	43995	O95497	VNN1_HUMAN	Pantetheinase (EC 3.5.1.92) (Pantetheine hydrolase) (Tiff66) (Vascular non-inflammatory molecule 1) (Vanin-1)	VNN1	hsa:8876	0.2735	0.0024	**
3	44001	P01008	ANT3_HUMAN	Antithrombin-III (ATIII) (Serpin C1)	SERPINC1 AT3 PRO0309	hsa:462	0.1105	0.0643	
4	44017	P04054	PA21B_HUMAN	Phospholipase A2 (EC 3.1.1.4) (Group IB phospholipase A2) (Phosphatidylcholine 2-acylhydrolase 1B)	PLA2G1B PLA2 PLA2A PPLA2	hsa:5319	0.1489	0.0612	
5	44024	P05109	S10A8_HUMAN	Protein S100-A8 (Calgranulin-A) (Calprotectin L1L subunit) (Cystic fibrosis antigen) (CFAG) (Leukocyte L1 complex light chain) (Migration inhibitory factor-related protein 8) (MRP-8) (p8) (S100 calcium-binding protein A8) (Urinary stone protein band A)	S100A8 CAGA CFAG MRP8	hsa:6279	0.3493	0.0031	**
6	44031	P07355	ANXA2_HUMAN	Annexin A2 (Annexin II) (Annexin-2) (Calpactin I heavy chain) (Calpactin-1 heavy chain) (Chromobindin-8) (Lipocortin II) (Placental anticoagulant protein IV) (PAP-IV) (Protein I) (p36)	ANXA2 ANX2 ANX2L4 CAL1H LPC2D	hsa:302	0.2710	0.0186	*
7	44033	P07478	TRY2_HUMAN	Trypsin-2 (EC 3.4.21.4) (Anionic trypsinogen) (Serine protease 2) (Trypsin II)	PRSS2 TRY2 TRYP2	hsa:5645	0.1646	0.0769	
8	44039	P08473	NEP_HUMAN	Neprilysin (EC 3.4.24.11) (Atriopeptidase) (Common acute lymphocytic leukemia antigen) (CALLA) (Enkephalinase) (Neutral endopeptidase 24.11) (NEP) (Neutral endopeptidase) (Skin fibroblast elastase) (SFE) (CD antigen CD10)	MME EPN	hsa:4311	0.2103	0.0970	
9	44043	P09525	ANXA4_HUMAN	Annexin A4 (35-beta calcimedin) (Annexin IV) (Annexin-4) (Carbohydrate-binding protein p33/p41) (Chromobindin-4) (Endonexin I) (Lipocortin IV) (P32.5) (PP4-X) (Placental anticoagulant protein II) (PAP-II) (Protein II)	ANXA4 ANX4	hsa:307	0.2391	0.0150	*
10	44050	P11678	PERE_HUMAN	Eosinophil peroxidase (EPO) (EC 1.11.1.7) [Cleaved into: Eosinophil peroxidase light chain; Eosinophil peroxidase heavy chain]	EPX EPER EPO EPP	hsa:8288	-0.2282	0.0681	
11	44063	P15085	CBPA1_HUMAN	Carboxypeptidase A1 (EC 3.4.17.1)	CPA1 CPA	hsa:1357	0.1817	0.1194	
12	44065	P15144	AMPN_HUMAN	Aminopeptidase N (AP-N) (hAPN) (EC 3.4.11.2) (Alanyl aminopeptidase) (Aminopeptidase M) (AP-M) (Microsomal aminopeptidase) (Myeloid plasma membrane glycoprotein CD13) (gp150) (CD antigen CD13)	ANPEP APN CD13 PEPN	hsa:290	0.4063	0.0074	**
13	44069	P16444	DPEP1_HUMAN	Dipeptidase 1 (EC 3.4.13.19) (Beta-lactamase) (EC 3.5.2.6) (Dehydropeptidase-I) (Microsomal dipeptidase) (Renal dipeptidase) (hRDP)	DPEP1 MDP RDP	hsa:1800	0.2003	0.0258	*
14	44097	P35237	SPB6_HUMAN	Serpin B6 (Cytoplasmic antiproteinase) (CAP) (Peptidase inhibitor 6) (PI-6) (Placental thrombin inhibitor)	SERPINB6 PI6 PTI	hsa:5269	0.1319	0.0283	*
15	44105	P50995	ANX11_HUMAN	Annexin A11 (56 kDa autoantigen) (Annexin XI) (Annexin-11) (Calcyclin-associated annexin 50) (CAP-50)	ANXA11 ANX11	hsa:311	0.0872	0.0822	
16	44115	P98088	MUC5A_HUMAN	Mucin-5AC (MUC-5AC) (Gastric mucin) (Major airway glycoprotein) (Mucin-5 subtype AC, tracheobronchial) (Tracheobronchial mucin) (TBM)	MUC5AC MUC5	hsa:4586	0.1242	0.0580	
17	44123	Q03403	TFF2_HUMAN	Trefoil factor 2 (Spasmolysin) (Spasmolytic polypeptide) (SP)	TFF2 SML1	hsa:7032	0.2216	0.0449	*
18	44134	Q6GPI1	CTRB2_HUMAN	Chymotrypsinogen B2 (EC 3.4.21.1) [Cleaved into: Chymotrypsin B2 chain A; Chymotrypsin B2 chain B; Chymotrypsin B2 chain C]	CTRB2	hsa:440387	0.2517	0.0521	
19	44153	Q9H3R2	MUC13_HUMAN	Mucin-13 (MUC-13) (Down-regulated in colon cancer 1)	MUC13 DRCC1 RECC UNQ6194/PRO20221	hsa:56667	0.1667	0.0539	

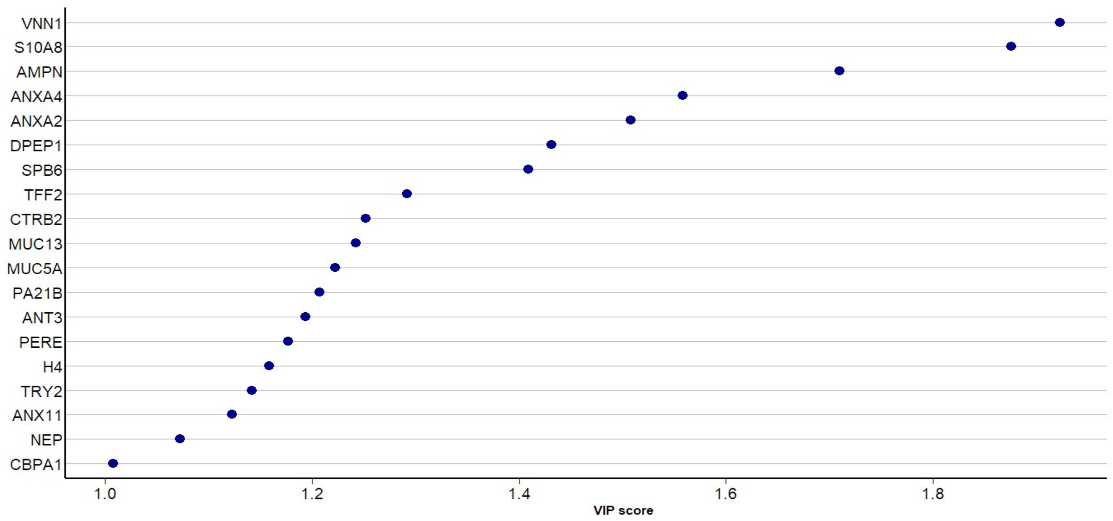
VNN1
S10A8
AMPN
ANXA4
ANXA2
DPEP1
SPB6
TFF2
CTRB2
MUC13
MUC5A
PA21B
ANT3
PERE
H4
TRY2
ANX11
NEP
CBPA1
1.0
1.2
1.4
1.6
1.8
VIP score

Differentially expressed Human PGs

N	Group ID	Leading razor protein accession	Leading razor protein entry name	Protein names	Gene names	KEGG ID	Log_{10} (WBS/CTRL)	p-value WBS/CTRL	Significance WBS/CTRL
1	44065	P15144	AMPN_HUMAN	Aminopeptidase N (AP-N) (hAPN) (EC 3.4.11.2) (Alanyl aminopeptidase) (Aminopeptidase M) (AP-M) (Microsomal aminopeptidase) (Myeloid plasma membrane glycoprotein CD13) (gp150) (CD antigen CD13)	ANPEP APN CD13 PEPN	hsa:290	0.4063	0.0074	**
2	44024	P05109	S10A8_HUMAN	Protein S100-A8 (Calgranulin-A) (Calprotectin L1L subunit) (Cystic fibrosis antigen) (CFAG) (Leukocyte L1 complex light chain) (Migration inhibitory factor-related protein 8) (MRP-8) (p8) (S100 calcium-binding protein A8) (Urinary stone protein band A)	S100A8 CAGA CFAG MRP8	hsa:6279	0.3493	0.0031	**
3	43995	O95497	VNN1_HUMAN	Pantetheinase (EC 3.5.1.92) (Pantetheine hydrolase) (Tiff66) (Vascular non-inflammatory molecule 1) (Vanin-1)	VNN1	hsa:8876	0.2735	0.0024	**
4	44031	P07355	ANXA2_HUMAN	Annexin A2 (Annexin II) (Annexin-2) (Calpactin I heavy chain) (Calpactin-1 heavy chain) (Chromobindin-8) (Lipocortin II) (Placental anticoagulant protein IV) (PAP-IV) (Protein I) (p36)	ANXA2 ANX2 ANX2L4 CAL1H LPC2D	hsa:302	0.2710	0.0186	*
5	44043	P09525	ANXA4_HUMAN	Annexin A4 (35-beta calcimedin) (Annexin IV) (Annexin-4) (Carbohydrate-binding protein p33/p41) (Chromobindin-4) (Endonexin I) (Lipocortin IV) (P32.5) (PP4-X) (Placental anticoagulant protein II) (PAP-II) (Protein II)	ANXA4 ANX4	hsa:307	0.2391	0.0150	*
6	44123	Q03403	TFF2_HUMAN	Trefoil factor 2 (Spasmolysin) (Spasmolytic polypeptide) (SP)	TFF2 SML1	hsa:7032	0.2216	0.0449	*
7	44069	P16444	DPEP1_HUMAN	Dipeptidase 1 (EC 3.4.13.19) (Beta-lactamase) (EC 3.5.2.6) (Dehydropeptidase-I) (Microsomal dipeptidase) (Renal dipeptidase) (hRDP)	DPEP1 MDP RDP	hsa:1800	0.2003	0.0258	*
