# Supplementary material for: Williams–Beuren syndrome shapes the gut microbiota metaproteome
Source: Sci Rep. 2023 Nov 3;13:18963. doi: 10.1038/s41598-023-46052-9 (PMC10624682; doi:10.1038/s41598-023-46052-9)
Supplement: Supplementary file 5 — Supplementary File 4. [file 41598_2023_46052_MOESM5_ESM.pdf]

WBS age PCA loadings

PGs with loadings coefficient on PC1 of PCA > |0.09| on which MANOVA test was performed

N	Protein Group ID	Leading razor protein accession	COG accession	COG name	COG category	KEGG name	LCA	Rank	Phylum	Class	Order	Family	Genus	Species
1	112	206672.BL0597	COG0058	Glucan phosphorylase	Carbohydrate transport and metabolism [G]	Starch and sucrose metabolism	Bifidobacterium	Genus	Actinobacteria	Actinobacteria	Bifidobacteriales	Bifidobacteriaceae	Bifidobacterium	
2	115	206672.BL0707	COG0126	3-phosphoglycerate kinase	Carbohydrate transport and metabolism [G]	Glycolysis / Gluconeogenesis	Bifidobacterium longum	Species	Actinobacteria	Actinobacteria	Bifidobacteriales	Bifidobacteriaceae	Bifidobacterium	Bifidobacterium longum
3	118	206672.BL0951	COG1882	Pyruvate-formate lyase	Energy production and conversion [C]	Pyruvate metabolism	Bifidobacterium	Genus	Actinobacteria	Actinobacteria	Bifidobacteriales	Bifidobacteriaceae	Bifidobacterium	
4	122	206672.BL0988	COG0469	Pyruvate kinase	Carbohydrate transport and metabolism [G]	Glycolysis / Gluconeogenesis	Bifidobacterium	Genus	Actinobacteria	Actinobacteria	Bifidobacteriales	Bifidobacteriaceae	Bifidobacterium	
5	340	391904.BLIJ_0145	COG0443	Molecular chaperone DnaK (HSP70)	Posttranslational modification, protein turnover, chaperones [O]	RNA degradation	Bifidobacterium	Genus	Actinobacteria	Actinobacteria	Bifidobacteriales	Bifidobacteriaceae	Bifidobacterium	
6	939	515619.EUBREC_1472	COG1145	Ferredoxin	Energy production and conversion [C]	Glycolysis / Gluconeogenesis	Clostridiales	Order	Firmicutes	Clostridia	Clostridiales			
7	1139	537937.BLIG_01296	COG4166	ABC-type oligopeptide transport system, periplasmic component	Amino acid transport and metabolism [E]	ABC transporters	Bifidobacterium	Genus	Actinobacteria	Actinobacteria	Bifidobacteriales	Bifidobacteriaceae	Bifidobacterium	
8	1349	566552.BIFCAT_00987	COG0282	Acetate kinase	Energy production and conversion [C]	Taurine and hypotaurine metabolism	Bifidobacterium	Genus	Actinobacteria	Actinobacteria	Bifidobacteriales	Bifidobacteriaceae	Bifidobacterium	
9	1633	759350.BLJ_0360	COG0228	Ribosomal protein S16	Translation, ribosomal structure and biogenesis [J]	Ribosome	Bifidobacterium	Genus	Actinobacteria	Actinobacteria	Bifidobacteriales	Bifidobacteriaceae	Bifidobacterium	
10	2809	DLM018_GL0016638	COG0092	Ribosomal protein S3	Translation, ribosomal structure and biogenesis [J]	Ribosome	Bifidobacterium	Genus	Actinobacteria	Actinobacteria	Bifidobacteriales	Bifidobacteriaceae	Bifidobacterium	
11	17817	MH0122_GL0107606	COG1653	ABC-type glycerol-3-phosphate transport system, periplasmic component	Carbohydrate transport and metabolism [G]	ABC transporters	Bifidobacterium	Genus	Actinobacteria	Actinobacteria	Bifidobacteriales	Bifidobacteriaceae	Bifidobacterium	
12	18868	MH0131_GL0154213	COG0544	FKBP-type peptidyl-prolyl cis-trans isomerase (trigger factor)	Posttranslational modification, protein turnover, chaperones [O]	NAN	Bifidobacterium	Genus	Actinobacteria	Actinobacteria	Bifidobacteriales	Bifidobacteriaceae	Bifidobacterium	
13	20757	MH0161_GL0142425	COG0148	Enolase	Carbohydrate transport and metabolism [G]	Glycolysis / Gluconeogenesis	Bifidobacterium adolescentis	Species	Actinobacteria	Actinobacteria	Bifidobacteriales	Bifidobacteriaceae	Bifidobacterium	Bifidobacterium adolescentis
14	21833	MH0188_GL0007212	COG0039	Malate/lactate dehydrogenase	Energy production and conversion [C]	Glycolysis / Gluconeogenesis	Bifidobacterium	Genus	Actinobacteria	Actinobacteria	Bifidobacteriales	Bifidobacteriaceae	Bifidobacterium	
15	21984	MH0188_GL0055295	COG1653	ABC-type glycerol-3-phosphate transport system, periplasmic component	Carbohydrate transport and metabolism [G]	ABC transporters	Bifidobacterium	Genus	Actinobacteria	Actinobacteria	Bifidobacteriales	Bifidobacteriaceae	Bifidobacterium	
16	22090	MH0188_GL0095502	COG0174	Glutamine synthetase	Amino acid transport and metabolism [E]	Alanine, aspartate and glutamate metabolism	Bifidobacterium	Genus	Actinobacteria	Actinobacteria	Bifidobacteriales	Bifidobacteriaceae	Bifidobacterium	
17	22205	MH0188_GL0126744	COG0459	Chaperonin GroEL (HSP60 family)	Posttranslational modification, protein turnover, chaperones [O]	RNA degradation	Bifidobacterium	Genus	Actinobacteria	Actinobacteria	Bifidobacteriales	Bifidobacteriaceae	Bifidobacterium	
18	22221	MH0188_GL0133256	COG1087	UDP-glucose 4-epimerase	Cell wall/membrane/envelope biogenesis [M]	Galactose metabolism	Bifidobacterium longum	Species	Actinobacteria	Actinobacteria	Bifidobacteriales	Bifidobacteriaceae	Bifidobacterium	Bifidobacterium longum
19	22254	MH0188_GL0145450	COG1882	Pyruvate-formate lyase	Energy production and conversion [C]	Pyruvate metabolism	Bifidobacterium adolescentis	Species	Actinobacteria	Actinobacteria	Bifidobacteriales	Bifidobacteriaceae	Bifidobacterium	Bifidobacterium adolescentis
20	22837	MH0193_GL0010680	COG0480	Translation elongation factor EF-G, a GTPase	Translation, ribosomal structure and biogenesis [J]	NAN	Bifidobacterium	Genus	Actinobacteria	Actinobacteria	Bifidobacteriales	Bifidobacteriaceae	Bifidobacterium	
21	23014	MH0193_GL0186495	COG1454	Alcohol dehydrogenase, class IV	Energy production and conversion [C]	Glycolysis / Gluconeogenesis	Bifidobacterium	Genus	Actinobacteria	Actinobacteria	Bifidobacteriales	Bifidobacteriaceae	Bifidobacterium	
22	23687	MH0203_GL0013763	COG0057	Glyceraldehyde-3-phosphate dehydrogenase/erythrose-4-phosphate dehydrogenase	Carbohydrate transport and metabolism [G]	Glycolysis / Gluconeogenesis	Bifidobacterium	Genus	Actinobacteria	Actinobacteria	Bifidobacteriales	Bifidobacteriaceae	Bifidobacterium	
23	23823	MH0203_GL0133062	COG0033	Phosphoglucomutase	Carbohydrate transport and metabolism [G]	Glycolysis / Gluconeogenesis	Bifidobacterium	Genus	Actinobacteria	Actinobacteria	Bifidobacteriales	Bifidobacteriaceae	Bifidobacterium	
24	28712	MH0293_GL0090794	COG1080	Phosphoenolpyruvate-protein kinase (PTS system EI component in bacteria)	Carbohydrate transport and metabolism [G]	Pyruvate metabolism	Clostridiales	Order	Firmicutes	Clostridia	Clostridiales			
25	30556	MH0341_GL0013504	COG0176	Transaldolase	Carbohydrate transport and metabolism [G]	Pentose phosphate pathway	Bifidobacterium	Genus	Actinobacteria	Actinobacteria	Bifidobacteriales	Bifidobacteriaceae	Bifidobacterium	
26	30909	MH0348_GL0122167	COG0085	DNA-directed RNA polymerase, beta subunit/140 kD subunit	Transcription [K]	Purine metabolism	Bifidobacterium	Genus	Actinobacteria	Actinobacteria	Bifidobacteriales	Bifidobacteriaceae	Bifidobacterium	
27	38121	NOM009_GL0086915	COG1250	3-hydroxyacyl-CoA dehydrogenase	Lipid transport and metabolism [I]	Fatty acid degradation	Clostridiales	Order	Firmicutes	Clostridia	Clostridiales			
28	41485	O2.UC32-0_GL0051137	COG2160	L-arabinose isomerase	Carbohydrate transport and metabolism [G]	Pentose and glucuronate interconversions	Bifidobacterium	Genus	Actinobacteria	Actinobacteria	Bifidobacteriales	Bifidobacteriaceae	Bifidobacterium	
29	41889	O2.UC37-1_GL0081003	COG0021	Transketolase	Carbohydrate transport and metabolism [G]	Pentose phosphate pathway	Bifidobacterium bifidum	Species	Actinobacteria	Actinobacteria	Bifidobacteriales	Bifidobacteriaceae	Bifidobacterium	Bifidobacterium bifidum
30	43491	O2.UC58-2_GL0017801	COG0360	Ribosomal protein S6	Translation, ribosomal structure and biogenesis [J]	Ribosome	Bifidobacterium	Genus	Actinobacteria	Actinobacteria	Bifidobacteriales	Bifidobacteriaceae	Bifidobacterium	
31	45166	T2D-122A_GL0083846	COG0176	Transaldolase	Carbohydrate transport and metabolism [G]	Pentose phosphate pathway	Bifidobacterium bifidum	Species	Actinobacteria	Actinobacteria	Bifidobacteriales	Bifidobacteriaceae	Bifidobacterium	Bifidobacterium bifidum
32	46846	V1.CD1-0-PT_GL0076248	COG3957	Phosphoketolase	Carbohydrate transport and metabolism [G]	Pentose phosphate pathway	Bifidobacterium bifidum	Species	Actinobacteria	Actinobacteria	Bifidobacteriales	Bifidobacteriaceae	Bifidobacterium	Bifidobacterium bifidum
33	46855	V1.CD1-0-PT_GL0103563	COG0148	Enolase	Carbohydrate transport and metabolism [G]	Glycolysis / Gluconeogenesis	Bifidobacterium bifidum	Species	Actinobacteria	Actinobacteria	Bifidobacteriales	Bifidobacteriaceae	Bifidobacterium	Bifidobacterium bifidum
34	51276	V1.UC11-0_GL0085451	COG0244	Ribosomal protein L10	Translation, ribosomal structure and biogenesis [J]	Ribosome	Bifidobacterium	Genus	Actinobacteria	Actinobacteria	Bifidobacteriales	Bifidobacteriaceae	Bifidobacterium	

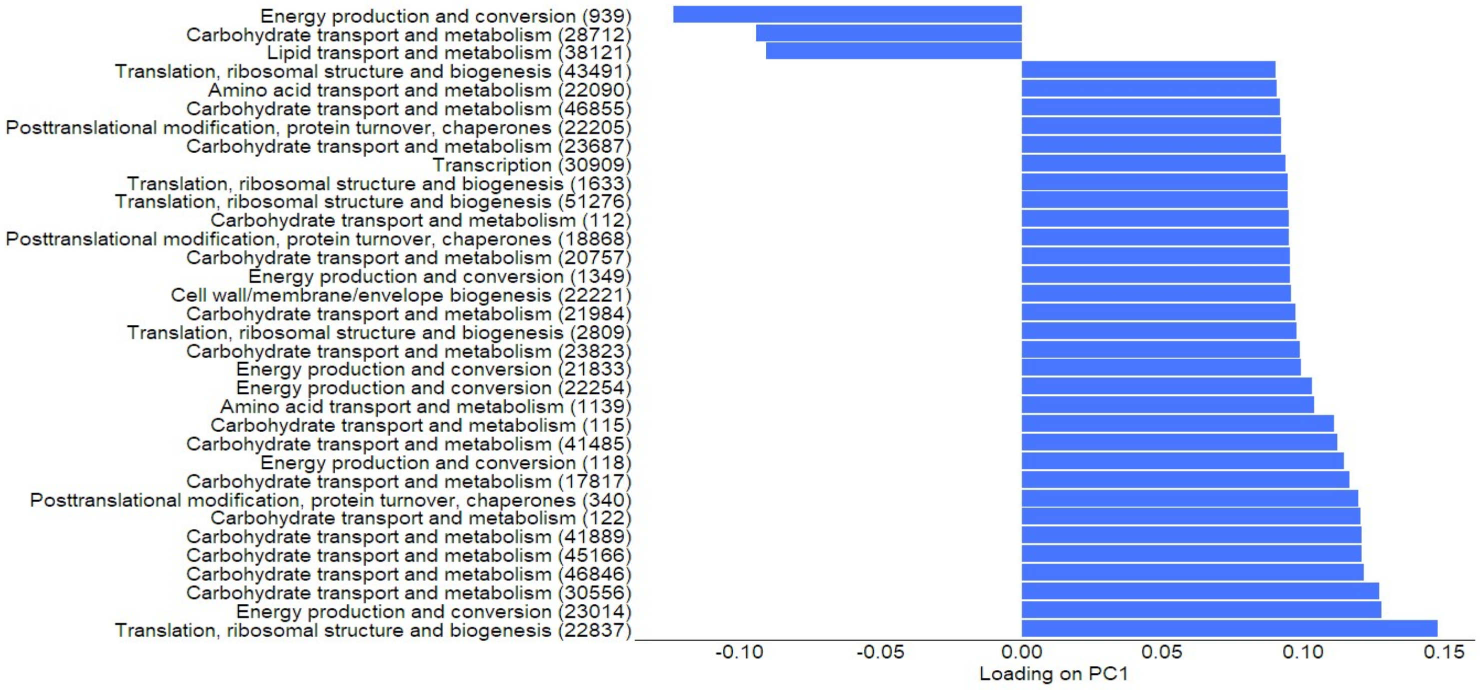
Energy production and conversion (939)
Carbohydrate transport and metabolism (28712)
Lipid transport and metabolism (38121)
Translation, ribosomal structure and biogenesis (43491)
Amino acid transport and metabolism (22090)
Carbohydrate transport and metabolism (46855)
Posttranslational modification, protein turnover, chaperones (22205)
Carbohydrate transport and metabolism (23687)
Transcription (30909)
Translation, ribosomal structure and biogenesis (1633)
Translation, ribosomal structure and biogenesis (51276)
Carbohydrate transport and metabolism (112)
Posttranslational modification, protein turnover, chaperones (18868)
Carbohydrate transport and metabolism (20757)
Energy production and conversion (1349)
Cell wall/membrane/envelope biogenesis (22221)
Carbohydrate transport and metabolism (21984)
Translation, ribosomal structure and biogenesis (2809)
Carbohydrate transport and metabolism (23823)
Energy production and conversion (21833)
Energy production and conversion (22254)
Amino acid transport and metabolism (1139)
Carbohydrate transport and metabolism (115)
Carbohydrate transport and metabolism (41485)
Energy production and conversion (118)
Carbohydrate transport and metabolism (17817)
Posttranslational modification, protein turnover, chaperones (340)
Carbohydrate transport and metabolism (122)
Carbohydrate transport and metabolism (41889)
Carbohydrate transport and metabolism (45166)
Carbohydrate transport and metabolism (46846)
Carbohydrate transport and metabolism (30556)
Energy production and conversion (23014)
Translation, ribosomal structure and biogenesis (22837)
-0.10
-0.05
0.00
0.05
0.10
0.15
Loading on PC1

CTRL age PCA loadings

PGs with loadings coefficient on PC1 of PCA > |0.09| on which MANOVA test was performed

N	Protein Group ID	Leading razor protein accession	COG accession	COG name	COG category	KEGG name	LCA	Rank	Phylum	Class	Order	Family	Genus	Species
1	1139	537937.BLIG_01296	COG4166	ABC-type oligopeptide transport system,	Amino acid transport and metabolism [E]	ABC transporters	Bifidobacterium	Genus	Actinobacteria	Actinobacteria	Bifidobacteriales	Bifidobacteriaceae	Bifidobacterium	
2	115	206672.BL0707	COG0126	3-phosphoglycerate kinase	Carbohydrate transport and metabolism [G]	Glycolysis / Gluconeogenesis	Bifidobacterium longum	Species	Actinobacteria	Actinobacteria	Bifidobacteriales	Bifidobacteriaceae	Bifidobacterium	Bifidobacterium longum
3	122	206672.BL0988	COG0469	Pyruvate kinase	Carbohydrate transport	Glycolysis /	Bifidobacterium	Genus	Actinobacteria	Actinobacteria	Bifidobacteriales	Bifidobacteriaceae	Bifidobacterium	
4	1349	566552.BIFCAT_00987	COG0282	Acetate kinase	Energy production and conversion [C]	Taurine and hypotaurine metabolism	Bifidobacterium	Genus	Actinobacteria	Actinobacteria	Bifidobacteriales	Bifidobacteriaceae	Bifidobacterium	
5	17817	MH0122_GL0107606	COG1653	ABC-type glycerol-3-phosphate transport	Carbohydrate transport and metabolism [G]	ABC transporters	Bifidobacterium	Genus	Actinobacteria	Actinobacteria	Bifidobacteriales	Bifidobacteriaceae	Bifidobacterium	
6	20672	MH0161_GL0083626	COG0480	Translation elongation factor EF-G, a GTPase	Translation, ribosomal structure and biogenesis	NAN	Bifidobacterium adolescentis	Species	Actinobacteria	Actinobacteria	Bifidobacteriales	Bifidobacteriaceae	Bifidobacterium	Bifidobacterium adolescentis
7	20757	MH0161_GL0142425	COG0148	Enolase	Carbohydrate transport and metabolism [G]	Glycolysis / Gluconeogenesis	Bifidobacterium adolescentis	Species	Actinobacteria	Actinobacteria	Bifidobacteriales	Bifidobacteriaceae	Bifidobacterium	Bifidobacterium adolescentis
8	22090	MH0188_GL0095502	COG0174	Glutamine synthetase	Amino acid transport and metabolism [E]	Alanine, aspartate and glutamate metabolism	Bifidobacterium	Genus	Actinobacteria	Actinobacteria	Bifidobacteriales	Bifidobacteriaceae	Bifidobacterium	
9	22254	MH0188_GL0145450	COG1882	Pyruvate-formate lyase	Energy production and conversion [C]	Pyruvate metabolism	Bifidobacterium adolescentis	Species	Actinobacteria	Actinobacteria	Bifidobacteriales	Bifidobacteriaceae	Bifidobacterium	Bifidobacterium adolescentis
10	22837	MH0193_GL0010680	COG0480	Translation elongation factor EF-G, a GTPase	Translation, ribosomal structure and biogenesis	NAN	Bifidobacterium	Genus	Actinobacteria	Actinobacteria	Bifidobacteriales	Bifidobacteriaceae	Bifidobacterium	
11	23014	MH0193_GL0186495	COG1454	Alcohol dehydrogenase,	Energy production and	Glycolysis /	Bifidobacterium	Genus	Actinobacteria	Actinobacteria	Bifidobacteriales	Bifidobacteriaceae	Bifidobacterium	
12	23687	MH0203_GL0013763	COG0057	Glyceraldehyde-3-phosphate	Carbohydrate transport and metabolism [G]	Glycolysis / Gluconeogenesis	Bifidobacterium	Genus	Actinobacteria	Actinobacteria	Bifidobacteriales	Bifidobacteriaceae	Bifidobacterium	
13	27731	MH0274_GL0075860	COG0539	Ribosomal protein S1	Translation, ribosomal structure and biogenesis	Ribosome	Bifidobacterium	Genus	Actinobacteria	Actinobacteria	Bifidobacteriales	Bifidobacteriaceae	Bifidobacterium	
14	28712	MH0293_GL0090794	COG1080	Phosphoenolpyruvate-protein kinase (PTS	Carbohydrate transport and metabolism [G]	Pyruvate metabolism	Clostridiales	Order	Firmicutes	Clostridia	Clostridiales			
15	30556	MH0341_GL0013504	COG0176	Transaldolase	Carbohydrate transport	Pentose phosphate	Bifidobacterium	Genus	Actinobacteria	Actinobacteria	Bifidobacteriales	Bifidobacteriaceae	Bifidobacterium	
16	30623	MH0341_GL0099782	COG0050	Translation elongation factor EF-Tu, a GTPase	Translation, ribosomal structure and biogenesis	Plant-pathogen interaction	Bifidobacterium longum	Species	Actinobacteria	Actinobacteria	Bifidobacteriales	Bifidobacteriaceae	Bifidobacterium	Bifidobacterium longum
17	30909	MH0348_GL0122167	COG0085	DNA-directed RNA polymerase, beta	Transcription [K]	Purine metabolism	Bifidobacterium	Genus	Actinobacteria	Actinobacteria	Bifidobacteriales	Bifidobacteriaceae	Bifidobacterium	
18	340	391904.BLIJ_0145	COG0443	Molecular chaperone DnaK (HSP70)	Posttranslational modification, protein	RNA degradation	Bifidobacterium	Genus	Actinobacteria	Actinobacteria	Bifidobacteriales	Bifidobacteriaceae	Bifidobacterium	
19	41485	O2.UC32-0_GL0051137	COG2160	L-arabinose isomerase	Carbohydrate transport and metabolism [G]	Pentose and glucuronate interconversions	Bifidobacterium	Genus	Actinobacteria	Actinobacteria	Bifidobacteriales	Bifidobacteriaceae	Bifidobacterium	
20	41889	O2.UC37-1_GL0081003	COG0021	Transketolase	Carbohydrate transport and metabolism [G]	Pentose phosphate pathway	Bifidobacterium bifidum	Species	Actinobacteria	Actinobacteria	Bifidobacteriales	Bifidobacteriaceae	Bifidobacterium	Bifidobacterium bifidum
21	45166	T2D-122A_GL0083846	COG0176	Transaldolase	Carbohydrate transport and metabolism [G]	Pentose phosphate pathway	Bifidobacterium bifidum	Species	Actinobacteria	Actinobacteria	Bifidobacteriales	Bifidobacteriaceae	Bifidobacterium	Bifidobacterium bifidum
22	46846	V1.CD1-0-PT_GL0076248	COG3957	Phosphoketolase	Carbohydrate transport and metabolism [G]	Pentose phosphate pathway	Bifidobacterium bifidum	Species	Actinobacteria	Actinobacteria	Bifidobacteriales	Bifidobacteriaceae	Bifidobacterium	Bifidobacterium bifidum
23	46855	V1.CD1-0-PT_GL0103563	COG0148	Enolase	Carbohydrate transport and metabolism [G]	Glycolysis / Gluconeogenesis	Bifidobacterium bifidum	Species	Actinobacteria	Actinobacteria	Bifidobacteriales	Bifidobacteriaceae	Bifidobacterium	Bifidobacterium bifidum
24	48199	V1.CD36-0_GL0032080	COG0166	Glucose-6-phosphate	Carbohydrate transport	Glycolysis /	Bifidobacterium	Genus	Actinobacteria	Actinobacteria	Bifidobacteriales	Bifidobacteriaceae	Bifidobacterium	
25	525	MH0002_GL0050951	COG1145	Ferredoxin	Energy production and conversion [C]	Glycolysis / Gluconeogenesis	Faecalibacterium prausnitzii	Species	Firmicutes	Clostridia	Clostridiales	Ruminococcaceae	Faecalibacterium	Faecalibacterium prausnitzii
26	939	515619.EUBREC_1472	COG1145	Ferredoxin	Energy production and	Glycolysis /	LVNELLDPIAK	Clostridiales	Order	Firmicutes	Clostridia	Clostridiales		

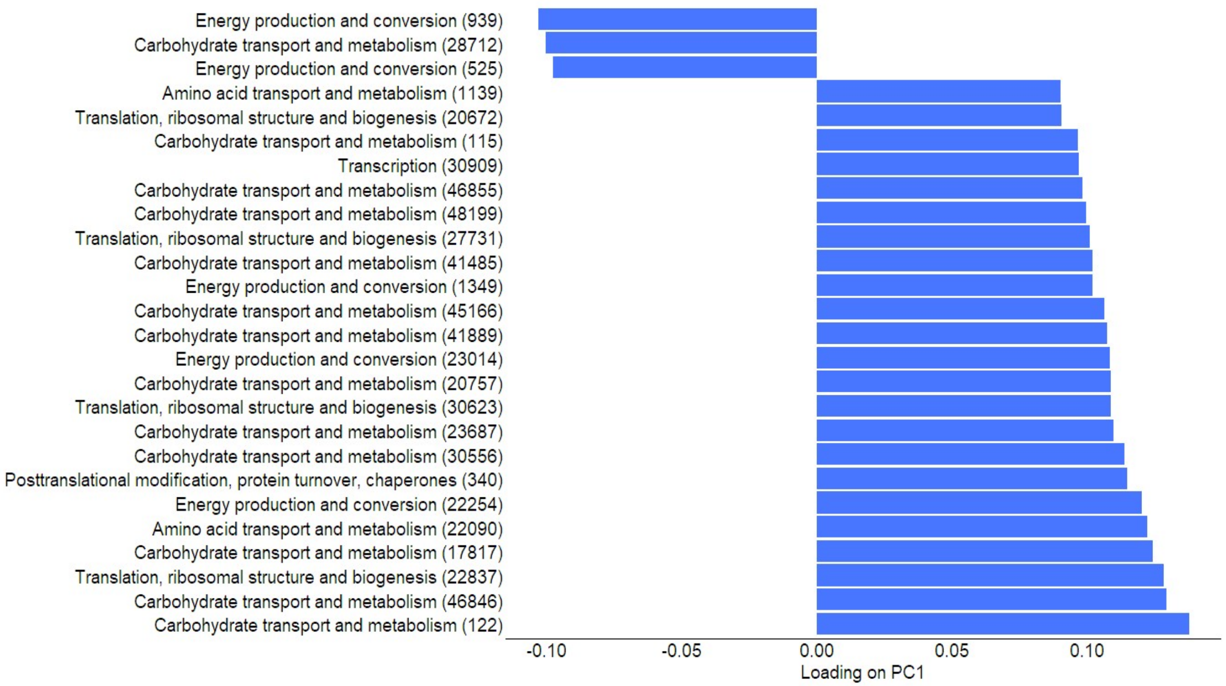
Energy production and conversion (939)
Carbohydrate transport and metabolism (28712)
Energy production and conversion (525)
Amino acid transport and metabolism (1139)
Translation, ribosomal structure and biogenesis (20672)
Carbohydrate transport and metabolism (115)
Transcription (30909)
Carbohydrate transport and metabolism (46855)
Carbohydrate transport and metabolism (48199)
Translation, ribosomal structure and biogenesis (27731)
Carbohydrate transport and metabolism (41485)
Energy production and conversion (1349)
Carbohydrate transport and metabolism (45166)
Carbohydrate transport and metabolism (41889)
Energy production and conversion (23014)
Carbohydrate transport and metabolism (20757)
Translation, ribosomal structure and biogenesis (30623)
Carbohydrate transport and metabolism (23687)
Carbohydrate transport and metabolism (30556)
Posttranslational modification, protein turnover, chaperones (340)
Energy production and conversion (22254)
Amino acid transport and metabolism (22090)
Carbohydrate transport and metabolism (17817)
Translation, ribosomal structure and biogenesis (22837)
Carbohydrate transport and metabolism (46846)
Carbohydrate transport and metabolism (122)
-0.10
-0.05
0.00
0.05
0.10
Loading on PC1

WBS+CTRL age PCA loadings

PGs with loadings coefficient on PC1 of PCA > |0.08| on which MANOVA test was performed

N	Protein Group ID	Leading razor protein accession	COG accession	COG name	COG category	KEGG name	LCA	Rank	Phylum	Class	Order	Family	Genus	Species
1	1139	537937.BLIG_01296	COG4166	ABC-type oligopeptide transport system, periplasmic component	Amino acid transport and metabolism [E]	ABC transporters	Bifidobacterium	Genus	Actinobacteria	Actinobacteria	Bifidobacteriales	Bifidobacteriaceae	Bifidobacterium	
2	115	206672.BL0707	COG0126	3-phosphoglycerate kinase	Carbohydrate transport and metabolism [G]	Glycolysis / Gluconeogenesis	Bifidobacterium longum	Species	Actinobacteria	Actinobacteria	Bifidobacteriales	Bifidobacteriaceae	Bifidobacterium	Bifidobacterium longum
3	118	206672.BL0951	COG1882	Pyruvate-formate lyase	Energy production and conversion [C]	Pyruvate metabolism	Bifidobacterium	Genus	Actinobacteria	Actinobacteria	Bifidobacteriales	Bifidobacteriaceae	Bifidobacterium	
4	122	206672.BL0988	COG0469	Pyruvate kinase	Carbohydrate transport and metabolism [G]	Glycolysis / Gluconeogenesis	Bifidobacterium	Genus	Actinobacteria	Actinobacteria	Bifidobacteriales	Bifidobacteriaceae	Bifidobacterium	
5	1349	566552.BIFCAT_00987	COG0282	Acetate kinase	Energy production and conversion [C]	Taurine and hypotaurine metabolism	Bifidobacterium	Genus	Actinobacteria	Actinobacteria	Bifidobacteriales	Bifidobacteriaceae	Bifidobacterium	
6	1633	759350.BLIJ_0360	COG0228	Ribosomal protein S16	Translation, ribosomal structure and biogenesis [J]	Ribosome	Bifidobacterium	Genus	Actinobacteria	Actinobacteria	Bifidobacteriales	Bifidobacteriaceae	Bifidobacterium	
7	17817	MH0122_GL0107606	COG1653	ABC-type glycerol-3-phosphate transport system, periplasmic component	Carbohydrate transport and metabolism [G]	ABC transporters	Bifidobacterium	Genus	Actinobacteria	Actinobacteria	Bifidobacteriales	Bifidobacteriaceae	Bifidobacterium	
8	18868	MH0131_GL0154213	COG0544	FKBP-type peptidyl-prolyl cis-trans isomerase (trigger factor)	Posttranslational modification, protein turnover, chaperones [O]	NAN	Bifidobacterium	Genus	Actinobacteria	Actinobacteria	Bifidobacteriales	Bifidobacteriaceae	Bifidobacterium	
9	20757	MH0161_GL0142425	COG0148	Enolase	Carbohydrate transport and metabolism [G]	Glycolysis / Gluconeogenesis	Bifidobacterium adolescentis	Species	Actinobacteria	Actinobacteria	Bifidobacteriales	Bifidobacteriaceae	Bifidobacterium	Bifidobacterium adolescentis
10	21833	MH0188_GL0007212	COG0039	Malate/lactate dehydrogenase	Energy production and conversion [C]	Glycolysis / Gluconeogenesis	Bifidobacterium	Genus	Actinobacteria	Actinobacteria	Bifidobacteriales	Bifidobacteriaceae	Bifidobacterium	
11	21984	MH0188_GL0055295	COG1653	ABC-type glycerol-3-phosphate transport system, periplasmic component	Carbohydrate transport and metabolism [G]	ABC transporters	Bifidobacterium	Genus	Actinobacteria	Actinobacteria	Bifidobacteriales	Bifidobacteriaceae	Bifidobacterium	
12	22027	MH0188_GL0067976	COG1129	ABC-type sugar transport system, ATPase component	Carbohydrate transport and metabolism [G]	ABC transporters	Bifidobacterium	Genus	Actinobacteria	Actinobacteria	Bifidobacteriales	Bifidobacteriaceae	Bifidobacterium	
13	22030	MH0188_GL0069412	COG0282	Acetate kinase	Energy production and conversion [C]	Taurine and hypotaurine metabolism	Bifidobacterium	Genus	Actinobacteria	Actinobacteria	Bifidobacteriales	Bifidobacteriaceae	Bifidobacterium	
14	22090	MH0188_GL0095502	COG0174	Glutamine synthetase	Amino acid transport and metabolism [E]	Alanine, aspartate and glutamate metabolism	Bifidobacterium	Genus	Actinobacteria	Actinobacteria	Bifidobacteriales	Bifidobacteriaceae	Bifidobacterium	
15	22205	MH0188_GL0126744	COG0459	Chaperonin GroEL (HSP60 family)	Posttranslational modification, protein turnover, chaperones [O]	RNA degradation	Bifidobacterium	Genus	Actinobacteria	Actinobacteria	Bifidobacteriales	Bifidobacteriaceae	Bifidobacterium	
16	22221	MH0188_GL0133256	COG1087	UDP-glucose 4-epimerase	Cell wall/membrane/envelope biogenesis [M]	Galactose metabolism	Bifidobacterium longum	Species	Actinobacteria	Actinobacteria	Bifidobacteriales	Bifidobacteriaceae	Bifidobacterium	Bifidobacterium longum
17	22254	MH0188_GL0145450	COG1882	Pyruvate-formate lyase	Energy production and conversion [C]	Pyruvate metabolism	Bifidobacterium adolescentis	Species	Actinobacteria	Actinobacteria	Bifidobacteriales	Bifidobacteriaceae	Bifidobacterium	Bifidobacterium adolescentis
18	22837	MH0193_GL0010680	COG0480	Translation elongation factor EF-G, a GTPase	Translation, ribosomal structure and biogenesis [J]	NAN	Bifidobacterium	Genus	Actinobacteria	Actinobacteria	Bifidobacteriales	Bifidobacteriaceae	Bifidobacterium	
19	23014	MH0193_GL0186495	COG1454	Alcohol dehydrogenase, class IV	Energy production and conversion [C]	Glycolysis / Gluconeogenesis	Bifidobacterium	Genus	Actinobacteria	Actinobacteria	Bifidobacteriales	Bifidobacteriaceae	Bifidobacterium	
20	23687	MH0203_GL0013763	COG0057	Glyceraldehyde-3-phosphate dehydrogenase/erythrose-4-phosphate dehydrogenase	Carbohydrate transport and metabolism [G]	Glycolysis / Gluconeogenesis	Bifidobacterium	Genus	Actinobacteria	Actinobacteria	Bifidobacteriales	Bifidobacteriaceae	Bifidobacterium	
21	23823	MH0203_GL0133062	COG0033	Phosphoglucomutase	Carbohydrate transport and metabolism [G]	Glycolysis / Gluconeogenesis	Bifidobacterium	Genus	Actinobacteria	Actinobacteria	Bifidobacteriales	Bifidobacteriaceae	Bifidobacterium	
22	27731	MH0274_GL0075860	COG0539	Ribosomal protein S1	Translation, ribosomal structure and biogenesis [J]	Ribosome	Bifidobacterium	Genus	Actinobacteria	Actinobacteria	Bifidobacteriales	Bifidobacteriaceae	Bifidobacterium	
23	2809	DLM018_GL0016638	COG0092	Ribosomal protein S3	Translation, ribosomal structure and biogenesis [J]	Ribosome	Bifidobacterium	Genus	Actinobacteria	Actinobacteria	Bifidobacteriales	Bifidobacteriaceae	Bifidobacterium	
24	28314	MH0284_GL0116366	COG0183	Acetyl-CoA acetyltransferase	Lipid transport and metabolism [I]	Fatty acid degradation	Clostridiales	Order	Firmicutes	Clostridia	Clostridiales			
25	28712	MH0293_GL0090794	COG1080	Phosphoenolpyruvate-protein kinase (PTS system EI component in bacteria)	Carbohydrate transport and metabolism [G]	Pyruvate metabolism	Clostridiales	Order	Firmicutes	Clostridia	Clostridiales			
26	30556	MH0341_GL0013504	COG0176	Transaldolase	Carbohydrate transport and metabolism [G]	Pentose phosphate pathway	Bifidobacterium	Genus	Actinobacteria	Actinobacteria	Bifidobacteriales	Bifidobacteriaceae	Bifidobacterium	
27	30623	MH0341_GL0099782	COG0050	Translation elongation factor EF-Tu, a GTPase	Translation, ribosomal structure and biogenesis [J]	Plant-pathogen interaction	Bifidobacterium longum	Species	Actinobacteria	Actinobacteria	Bifidobacteriales	Bifidobacteriaceae	Bifidobacterium	Bifidobacterium longum
28	30834	MH0347_GL0010219	COG1653	ABC-type glycerol-3-phosphate transport system, periplasmic component	Carbohydrate transport and metabolism [G]	NAN	Bifidobacterium	Genus	Actinobacteria	Actinobacteria	Bifidobacteriales	Bifidobacteriaceae	Bifidobacterium	
29	30909	MH0348_GL0122167	COG0085	DNA-directed RNA polymerase, beta subunit/140 kD subunit	Transcription [K]	Purine metabolism	Bifidobacterium	Genus	Actinobacteria	Actinobacteria	Bifidobacteriales	Bifidobacteriaceae	Bifidobacterium	
30	340	391904.BLIJ_0145	COG0443	Molecular chaperone DnaK (HSP70)	Posttranslational modification, protein turnover, chaperones [O]	RNA degradation	Bifidobacterium	Genus	Actinobacteria	Actinobacteria	Bifidobacteriales	Bifidobacteriaceae	Bifidobacterium	
31	41485	O2.UC32-0_GL0051137	COG2160	L-arabinose isomerase	Carbohydrate transport and metabolism [G]	Pentose and glucuronate interconversions	Bifidobacterium	Genus	Actinobacteria	Actinobacteria	Bifidobacteriales	Bifidobacteriaceae	Bifidobacterium	
32	41889	O2.UC37-1_GL0081003	COG0021	Transketolase	Carbohydrate transport and metabolism [G]	Pentose phosphate pathway	Bifidobacterium bifidum	Species	Actinobacteria	Actinobacteria	Bifidobacteriales	Bifidobacteriaceae	Bifidobacterium	Bifidobacterium bifidum
33	43491	O2.UC58-2_GL0017801	COG0360	Ribosomal protein S6	Translation, ribosomal structure and biogenesis [J]	Ribosome	Bifidobacterium	Genus	Actinobacteria	Actinobacteria	Bifidobacteriales	Bifidobacteriaceae	Bifidobacterium	
34	45166	T2D-122A_GL0083846	COG0176	Transaldolase	Carbohydrate transport and metabolism [G]	Pentose phosphate pathway	Bifidobacterium bifidum	Species	Actinobacteria	Actinobacteria	Bifidobacteriales	Bifidobacteriaceae	Bifidobacterium	Bifidobacterium bifidum
35	46846	V1.CD1-0-PT_GL0076248	COG3957	Phosphoketolase(559)	Carbohydrate transport and metabolism [G]	Pentose phosphate pathway	Bifidobacterium bifidum	Species	Actinobacteria	Actinobacteria	Bifidobacteriales	Bifidobacteriaceae	Bifidobacterium	Bifidobacterium bifidum
36	46855	V1.CD1-0-PT_GL0103563	COG0148	Enolase	Carbohydrate transport and metabolism [G]	Glycolysis / Gluconeogenesis	Bifidobacterium bifidum	Species	Actinobacteria	Actinobacteria	Bifidobacteriales	Bifidobacteriaceae	Bifidobacterium	Bifidobacterium bifidum
37	48199	V1.CD36-0_GL0032080	COG0166	Glucose-6-phosphate isomerase	Carbohydrate transport and metabolism [G]	Glycolysis / Gluconeogenesis	Bifidobacterium	Genus	Actinobacteria	Actinobacteria	Bifidobacteriales	Bifidobacteriaceae	Bifidobacterium	
38	51276	V1.UC11-0_GL0085451	COG0244	Ribosomal protein L10	Translation, ribosomal structure and biogenesis [J]	Ribosome	Bifidobacterium	Genus	Actinobacteria	Actinobacteria	Bifidobacteriales	Bifidobacteriaceae	Bifidobacterium	
39	525	411485.FAEPRAM212_01761	COG1145	Ferredoxin	Energy production and conversion [C]	Glycolysis / Gluconeogenesis	Faecalibacterium prausnitzii	Species	Firmicutes	Clostridia	Clostridiales	Ruminococcaceae	Faecalibacterium	Faecalibacterium prausnitzii
40	939	515619.EUBREC_1472	COG1145	Ferredoxin	Energy production and conversion [C]	Glycolysis / Gluconeogenesis	Clostridiales	Order	Firmicutes	Clostridia	Clostridiales			

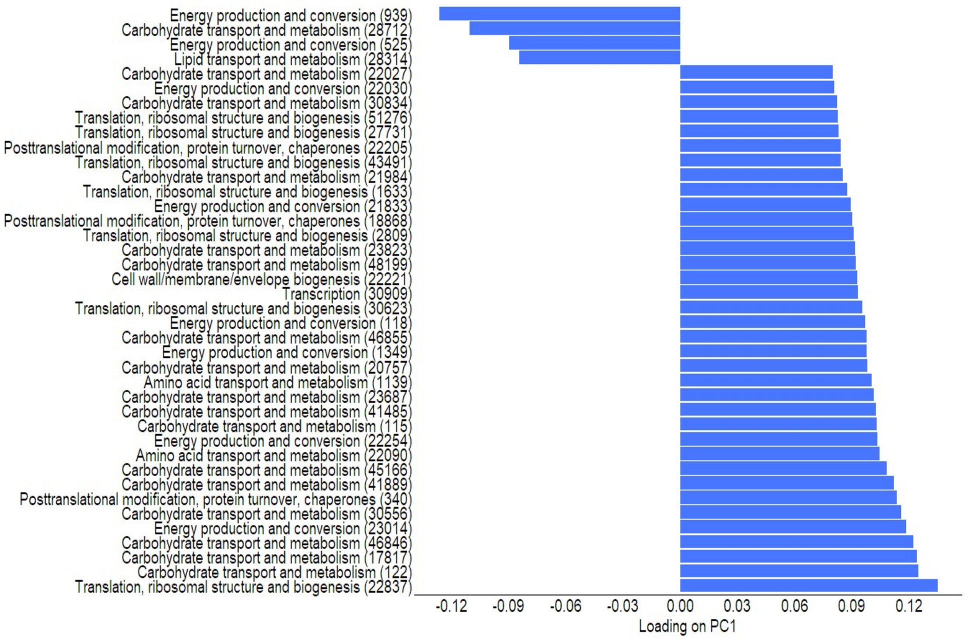

Energy production and conversion (939)
Carbohydrate transport and metabolism (28712)
Energy production and conversion (525)
Lipid transport and metabolism (28314)
Carbohydrate transport and metabolism (22027)
Energy production and conversion (22030)
Carbohydrate transport and metabolism (30834)
Translation, ribosomal structure and biogenesis (51276)
Translation, ribosomal structure and biogenesis (27731)
Posttranslational modification, protein turnover, chaperones (22205)
Translation, ribosomal structure and biogenesis (43491)
Carbohydrate transport and metabolism (21984)
Translation, ribosomal structure and biogenesis (1633)
Energy production and conversion (21833)
Posttranslational modification, protein turnover, chaperones (18868)
Translation, ribosomal structure and biogenesis (2809)
Carbohydrate transport and metabolism (23823)
Carbohydrate transport and metabolism (48199)
Cell wall/membrane/envelope biogenesis (22221)
Transcription (30909)
Translation, ribosomal structure and biogenesis (30623)
Energy production and conversion (118)
Carbohydrate transport and metabolism (46855)
Energy production and conversion (1349)
Carbohydrate transport and metabolism (20757)
Amino acid transport and metabolism (1139)
Carbohydrate transport and metabolism (23687)
Carbohydrate transport and metabolism (41485)
Carbohydrate transport and metabolism (115)
Energy production and conversion (22254)
Amino acid transport and metabolism (22090)
Carbohydrate transport and metabolism (45166)
Carbohydrate transport and metabolism (41889)
Posttranslational modification, protein turnover, chaperones (340)
Carbohydrate transport and metabolism (30556)
Energy production and conversion (23014)
Carbohydrate transport and metabolism (46846)
Carbohydrate transport and metabolism (17817)
Carbohydrate transport and metabolism (122)
Translation, ribosomal structure and biogenesis (22837)
-0.12 -0.09 -0.06 -0.03 0.00 0.03 0.06 0.09 0.12
Loading on PC1

Univariate analysis

Age Group	Age	# WBS	# CTRL
**0**	years < 5	5	5
**1**	5 ≤ years < 13	22	14
**2**	13 ≤ years < 18	11	9
**3**	18 ≤ years ≤ 42	7	13

	Comparisons	Kruskal-Wallis	Kruskal-Wallis p-value	Kruskal-Wallis Significance	ANOVA	ANOVA p-value	ANOVA Significance	Mann-Whitney	Mann-Whitney p-value	Mann-Whitney Significance
WBS	Age Group 1 VS Age Group 0	5.24064032	0.022065151	*	2.394261548	0.121807151		14077743	0.02206532	*
	Age Group 2 VS Age Group 0	1.302847678	0.253693717		1.726583621	0.188881279		9167747.5	0.253695477	
	Age Group 0 VS Age Group 3	1.278909733	0.25810185		0.228621124	0.632557167		13619170.5	0.258103158	
	Age Group 2 VS Age Group 1	1.679957472	0.194930104		0.042488854	0.836693573		26375714	0.194930783	
	Age Group 1 VS Age Group 3	2.189644214	0.138941406		2.069102584	0.150327973		37135608.5	0.138941811	
	Age Group 2 VS Age Group 3	0.008383932	0.927044601		1.18795661	0.275760689		24164900.5	0.927046264	
CTRL	Age Group 1 VS Age Group 0	32.78465411	1.02954E-08	***	26.69673167	2.40659E-07	***	24208458.5	1.02956E-08	***
	Age Group 2 VS Age Group 0	5.228589616	0.022218542	*	5.393805311	0.020227771	*	11689134	0.022218751	*
	Age Group 0 VS Age Group 3	12.49408309	0.000408243	***	11.16289635	0.00083846	***	7576339	0.000408251	***
	Age Group 2 VS Age Group 1	16.29853644	5.41057E-05	***	12.59972224	0.000386638	***	48329183	5.4106E-05	***
	Age Group 1 VS Age Group 3	114.5164717	1.00429E-26	***	102.0725979	6.16847E-24	***	35201702.5	1.0043E-26	***
	Age Group 2 VS Age Group 3	41.74084833	1.04209E-10	***	41.10628062	1.49782E-10	***	17049944	1.04211E-10	***
CTRL+WBS	Age Group 1 VS Age Group 0	9.562416332	0.001986015	**	9.884225998	0.001668665	**	72547779.5	0.00099301	***
	Age Group 2 VS Age Group 0	0.768331603	0.38073368		0.60483665	0.436748092		41025314.5	0.190367212	
	Age Group 0 VS Age Group 3	5.423880929	0.019863161	*	2.866458421	0.090459844		40495782.5	0.009931617	**
	Age Group 2 VS Age Group 1	7.449435112	0.006345636	**	8.751072795	0.003096211	**	146262031.5	0.003172823	**
	Age Group 1 VS Age Group 3	47.85468263	4.59009E-12	***	39.7825091	2.87167E-10	***	142303498	2.29506E-12	***
	Age Group 2 VS Age Group 3	13.93087298	0.000189658	***	8.95534692	0.002769212	**	80467598.5	9.48291E-05	***
CTRL vs WBS	Age Group 0	4.613507874	0.031721081	*	3.53172999	0.060250143		5008250.5	0.031721615	*
	Age Group 1	56.71869704	5.02832E-14	***	41.49143518	1.20715E-10	***	67403862	5.02836E-14	***
	Age Group 2	1.556174799	0.212226183		3.287852207	0.06981757		20727004.5	0.212227058	
	Age Group 3	32.21619044	1.37937E-08	***	34.9126126	3.53568E-09	***	17672164	1.37939E-08	***
