# Supplementary material for: Williams–Beuren syndrome shapes the gut microbiota metaproteome
Source: Sci Rep. 2023 Nov 3;13:18963. doi: 10.1038/s41598-023-46052-9 (PMC10624682; doi:10.1038/s41598-023-46052-9)
Supplement: Supplementary file 6 — Supplementary File 5. [file 41598_2023_46052_MOESM6_ESM.pdf]

WBS metadata

# Metaproteomic sample	Code	Sex	Age (years)	Weight (kg)	Height (cm)	BMI	Omnivorous diet yes = 0, no = 1*	Obesity no = 0, yes = 1	GERD** no = 0, yes = 1	Constipation no = 0, yes = 1	Diarrhea no = 0, yes = 1	Abdominal pain 0=no, 1=yes	Cardiovascular abnormalities 0 = no, 1 = yes	Hypertension 0 = no, 1 = yes
1	WBS-1	F	10	46.4	141.3	23.24	0	0	0	0	0	0	0	0
2	WBS-2	F	29	72	154.5	30.16	0	1	0	0	0	0	1	1
3	WBS-3	F	16	61	145.7	28.73	0	0	0	1	0	1	1	1
4	WBS-4	F	28	88.8	151.8	38.54	0	1	0	0	0	0	1	1
5	WBS-5	F	1	7.1	67	15.82	1	0	1	1	0	0	1	0
6	WBS-6	F	2	13	85	17.99	0	0	0	1	0	0	1	0
7	WBS-7	M	22	75.2	167	26.96	0	0	1	1	0	0	1	1
8	WBS-8	F	28	55.3	149	24.91	0	0	0	0	0	0	1	1
9	WBS-9	F	22	58.5	147.3	26.96	0	0	0	0	0	0	1	0
10	WBS-10	M	18	97	170	33.56	0	1	0	0	0	0	0	1
11	WBS-11	M	8	24	122.7	15.94	0	0	0	0	1	1	0	0
12	WBS-12	F	22	71	149	31.98	0	1	0	0	0	0	0	1
13	WBS-13	F	17	46.1	154	19.44	1	0	1	0	0	0	0	1
14	WBS-15	M	11	42.6	150	18.93	0	0	0	0	1	1	1	0
15	WBS-17	M	27	62.5	167	22.41	1	0	0	0	1	0	1	1
16	WBS-18	F	10	28.5	131.9	16.38	1	0	0	0	0	0	0	0
17	WBS-20	M	13	101	157	40.98	0	1	0	0	0	0	0	1
18	WBS-21	M	17	62	156	25.48	1	0	1	0	0	1	1	1
19	WBS-22	M	28	68	169	23.81	0	0	1	1	0	1	0	1
20	WBS-23	M	9	29	137	15.45	0	0	0	0	0	0	0	1
21	WBS-24	F	9	40	141.6	19.95	0	0	0	0	0	0	1	0
22	WBS-25	M	5	11	96	11.94	0	0	0	0	1	0	1	0
23	WBS-26	F	18	41.5	147.7	19.02	0	0	0	1	0	0	0	0
24	WBS-27	F	12	32.4	137	17.26	0	0	0	1	0	0	0	0
25	WBS-28	M	13	46.5	146.7	21.61	0	0	0	0	1	0	1	0
26	WBS-29	F	13	50	152	21.64	0	0	0	1	0	0	0	0
27	WBS-30	F	3	13	90.9	15.73	0	0	0	0	0	0	1	0
28	WBS-32	F	39	68.5	145	32.58	0	1	0	0	0	0	0	0
29	WBS-33	F	7	18	114.4	13.75	0	0	0	0	0	0	0	0
30	WBS-34	M	6	23	118.8	16.30	1	0	0	0	0	0	1	1
31	WBS-35	F	13	51	144.5	24.42	0	1	1	0	0	0	1	0
32	WBS-36	F	10	20.8	140	10.61	0	0	0	0	0	0	1	0
33	WBS-37	F	14	36.5	146.5	17.01	1	0	0	0	1	0	0	0
34	WBS-38	F	19	72	150	32.00	0	1	0	1	0	0	1	1
35	WBS-39	M	4	16	106	14.24	0	0	0	0	0	0	1	1
36	WBS-40	M	7	19.1	125.2	12.18	1	0	1	0	0	0	0	0
37	WBS-41	M	30	45	162	17.15	0	0	0	0	0	0	1	1
38	WBS-42	F	14	41	153.2	17.47	0	0	1	0	0	0	0	0
39	WBS-43	M	6	20	110.4	16.41	1	0	0	0	0	0	1	0
40	WBS-44	F	9	37	141	18.61	0	0	1	1	0	0	0	0
41	WBS-45	M	1	11.6	79	18.59	0	0	1	0	0	1	1	0
		**mean**	**14**	**44.46**	**137.08**	**21.61**	**9**	**8**	**10**	**10**	**6**	**6**	**23**	**17**
		**s.d.**	**9**	**24.54**	**25.27**	**7.30**	**22%**	**20%**	**24%**	**24%**	**15%**	**15%**	**56%**	**41%**

*** = WILL-18 AND 21 were celiac**

GERD = Gastro-esophageal reflux disease**

WBS patients stratification

Group	A. Diet	Celiac disease	C. Gastrointestinal (GI) symptoms: Gastroesophageal reflux (GERD)	Diarrhea	Constipation	Abdominal pain	B. Obesity	Weight status	Cardiovascular abnormalities	Hypertension
	omnivorous = 0, food elimination for intolerances or predilition = 1	no = 0, yes = 1	no = 0, yes = 1	no = 0, yes=1	no = 0, yes = 1	no = 0, yes = 1	no = 0, yes = 1	normalweight = 0, obesity = 1, overweight = 2, underweight = 3	no = 0, yes = 1	no = 0, yes = 1
0	32	39	31	35	31	35	33	12 (29%)	18	24
1	9	2	10	6	10	6	8	8 (20%)	23	17
2								4 (10%)		
3								17 (17%)		
**Sum**	41	41	41	41	41	41	41	41	41	41

C.. Gastrointestinal (GI) symptoms

	Absence of GI symptoms	One functional GI symptoms		Two functional GI symptoms		Three functional GI symptoms		Four functional GI symptoms		≥ one functional GI symptoms	≥ two functional GI symptoms
**# patients**	19	13		8		1		0		22	9
**% respect total**	46	32		20		2		0		54	22
		**List of symptoms**	**# patients**	**List of symptoms**	**# patients**	**List of symptoms**	**# patients**	**List of symptoms**	**# patients**		
		GERD	4	GERD + Constipation	3	GERD + Constipation + Abdominal pain	1	GERD	0		
		Constipation	5	GERD + Abdominal pain	2			Constipation	0		
		Diarrhea	4	Constipation + Abdominal pain	1			Diarrhea	0		
		Abdominal pain	0	Diarrhea + Abdominal pain	2			Abdominal pain	0		
		**Sum**	**13**	**Sum**	8	**Sum**	1	**Sum**	0		

638 identified bacteria PGs

N	Protein Group ID	Leading razor protein accession	COG accession	COG name	COG category	KEGG name	LCA	Rank	Phylum	Class	Order	Family	Genus	Species	Log_{10} (Omnivorous diet no/yes)	t-test p-value Omnivorous diet no/yes	Significance Omnivorous diet no/yes	Log_{10} (Obesity yes/no)	t-test p-value Obesity yes/no	Significance Obesity yes/no	Log_{10} (GI symptoms yes/no)	t-test p-value GI symptoms yes/no	Significance GI symptoms yes/no
1	112	206672.BL0597	COG0058	Glucan phosphorylase	Carbohydrate transport and metabolism [G]	Starch and sucrose metabolism	Bifidobacterium	Genus	Actinobacteria	Actinobacteria	Bifidobacteriales	Bifidobacteriaceae	Bifidobacterium		-0.0575	0.7747		-0.2208	0.2891		-0.1121	0.4999	
2	115	206672.BL0707	COG0126	3-phosphoglycerate kinase	Carbohydrate transport and metabolism [G]	Glycolysis / Gluconeogenesis	Bifidobacterium longum	Species	Actinobacteria	Actinobacteria	Bifidobacteriales	Bifidobacteriaceae	Bifidobacterium	Bifidobacterium longum	0.1038	0.6108		-0.1702	0.4229		0.0699	0.6798	
3	118	206672.BL0951	COG1882	Pyruvate-formate lyase	Energy production and conversion [C]	Pyruvate metabolism	Bifidobacterium	Genus	Actinobacteria	Actinobacteria	Bifidobacteriales	Bifidobacteriaceae	Bifidobacterium		0.2143	0.3087		-0.0426	0.8474		0.0942	0.5914	
4	122	206672.BL0988	COG0469	Pyruvate kinase	Carbohydrate transport and metabolism [G]	Glycolysis / Gluconeogenesis	Bifidobacterium	Genus	Actinobacteria	Actinobacteria	Bifidobacteriales	Bifidobacteriaceae	Bifidobacterium		0.0538	0.8094		-0.1031	0.6578		0.1505	0.4144	
5	135	206672.BL1722	COG0517	CBS domain	Signal transduction mechanisms [T]	Purine metabolism	Bifidobacterium	Genus	Actinobacteria	Actinobacteria	Bifidobacteriales	Bifidobacteriaceae	Bifidobacterium		0.0639	0.6021		-0.1546	0.2238		0.0565	0.5788	
6	280	367928.BAD_0348	COG0203	Ribosomal protein L17	Translation, ribosomal structure and biogenesis [J]	Ribosome	Bifidobacterium adolescentis	Species	Actinobacteria	Actinobacteria	Bifidobacteriales	Bifidobacteriaceae	Bifidobacterium	Bifidobacterium adolescentis	-0.0678	0.3935		-0.0617	0.4578		-0.0201	0.7609	
7	284	367928.BAD_0378	COG0588	Phosphoglycerate mutase (BPG-dependent)	Carbohydrate transport and metabolism [G]	Glycolysis / Gluconeogenesis	Bifidobacterium	Genus	Actinobacteria	Actinobacteria	Bifidobacteriales	Bifidobacteriaceae	Bifidobacterium		-0.1250	0.4643		-0.2137	0.2281		-0.0132	0.9258	
8	286	367928.BAD_0450	COG0517	CBS domain	Signal transduction mechanisms [T]	Purine metabolism	Bifidobacterium adolescentis	Species	Actinobacteria	Actinobacteria	Bifidobacteriales	Bifidobacteriaceae	Bifidobacterium	Bifidobacterium adolescentis	-0.1583	0.3905		0.0350	0.8565		-0.0932	0.5433	
9	340	391904.BLIJ_0145	COG0443	Molecular chaperone DnaK (HSP70)	Posttranslational modification, protein turnover, chaperones [O]	RNA degradation	Bifidobacterium	Genus	Actinobacteria	Actinobacteria	Bifidobacteriales	Bifidobacteriaceae	Bifidobacterium		-0.1734	0.4219		0.0682	0.7632		0.0568	0.7521	
10	507	411483.FAEPRAA2165_01405	COG0330	Regulator of protease activity HflC, stomatin/prohibitin superfamily(3991)	Posttranslational modification, protein turnover, chaperones [O]	NAN	Faecalibacterium prausnitzii	Species	Firmicutes	Clostridia	Clostridiales	Ruminococcaceae	Faecalibacterium	Faecalibacterium prausnitzii	-0.1682	0.2349		-0.0787	0.5973		-0.0107	0.9281	
11	525	411485.FAEPRAM212_01761	COG1145	Ferredoxin	Energy production and conversion [C]	Glycolysis / Gluconeogenesis	Faecalibacterium prausnitzii	Species	Firmicutes	Clostridia	Clostridiales	Ruminococcaceae	Faecalibacterium	Faecalibacterium prausnitzii	-0.2051	0.3692		0.1330	0.5785		-0.0475	0.8030	
12	552	411903.COLAER_00059	COG1080	Phosphoenolpyruvate-protein kinase (PTS system EI component in bacteria)(4879)	Carbohydrate transport and metabolism [G]	Fructose and mannose metabolism	Collinsella	Genus	Actinobacteria	Coriobacteriia	Coriobacteriales	Coriobacteriaceae	Collinsella		-0.2124	0.0829		-0.1355	0.2956		-0.0337	0.7449	
13	572	411903.COLAER_02212	COG4166	ABC-type oligopeptide transport system, periplasmic component(3057)	Amino acid transport and metabolism [E]	ABC transporters	Collinsella aerofaciens	Species	Actinobacteria	Coriobacteriia	Coriobacteriales	Coriobacteriaceae	Collinsella	Collinsella aerofaciens	-0.2441	0.1181		-0.1138	0.4912		-0.1159	0.3767	
14	606	435590.BVU_0563	COG4771	Outer membrane receptor for ferrienterochelin and colicins	Inorganic ion transport and metabolism [P]	NAN	Bacteroides	Genus	Bacteroidetes	Bacteroidia	Bacteroidales	Bacteroidaceae	Bacteroides		0.6697	0.0246	*	-0.1365	0.6708		0.1796	0.4806	
15	708	457395.BSBG_00304	COG3637	Opacity protein and related surface antigens	Cell wall/membrane/envelope biogenesis [M]	NAN	Bacteroides	Genus	Bacteroidetes	Bacteroidia	Bacteroidales	Bacteroidaceae	Bacteroides		0.3481	0.1056		0.0420	0.8540		0.0470	0.7958	
16	789	469594.HMPREF0177_01181	COG0221	Inorganic pyrophosphatase	Inorganic ion transport and metabolism [P]	Oxidative phosphorylation	Bifidobacterium	Genus	Actinobacteria	Actinobacteria	Bifidobacteriales	Bifidobacteriaceae	Bifidobacterium		-0.0015	0.9924		-0.1313	0.4152		-0.0548	0.6697	
17	923	483217.BACDOR_00668	COG3525	N-acetyl-beta-hexosaminidase	Carbohydrate transport and metabolism [G]	NAN	Bacteroides	Genus	Bacteroidetes	Bacteroidia	Bacteroidales	Bacteroidaceae	Bacteroides		0.3345	0.0409	*	-0.2331	0.1792		0.0692	0.6195	
18	939	515619.EUBREC_1472	COG1145	Ferredoxin	Energy production and conversion [C]	Glycolysis / Gluconeogenesis	Clostridiales	Order	Firmicutes	Clostridia	Clostridiales				-0.4512	0.1448		0.3390	0.2975		-0.2189	0.3985	
19	960	518634.BIFBRE_03753	COG3957	Phosphoketolase	Carbohydrate transport and metabolism [G]	Pentose phosphate pathway	Bifidobacterium	Genus	Actinobacteria	Actinobacteria	Bifidobacteriales	Bifidobacteriaceae	Bifidobacterium		0.0166	0.9037		-0.1263	0.3761		0.0036	0.9748	
20	1041	MH0203_GL0115820	COG0093	Ribosomal protein L14	Translation, ribosomal structure and biogenesis [J]	Ribosome	Bifidobacterium	Genus	Actinobacteria	Actinobacteria	Bifidobacteriales	Bifidobacteriaceae	Bifidobacterium		-0.0165	0.8852		-0.0923	0.4365		0.0258	0.7849	
21	1139	537937.BLIG_01296	COG4166	ABC-type oligopeptide transport system, periplasmic component	Amino acid transport and metabolism [E]	ABC transporters	Bifidobacterium	Genus	Actinobacteria	Actinobacteria	Bifidobacteriales	Bifidobacteriaceae	Bifidobacterium		0.1571	0.4600		-0.4142	0.0571		0.0795	0.6530	
22	1188	548480.HMPREF0175_0263	COG1070	Sugar (pentulose or hexulose) kinase	Carbohydrate transport and metabolism [G]	Pentose phosphate pathway	Bifidobacterium	Genus	Actinobacteria	Actinobacteria	Bifidobacteriales	Bifidobacteriaceae	Bifidobacterium		-0.0661	0.6474		-0.1781	0.2346		-0.0285	0.8123	
23	1264	V1.CD21-4_GL0001620			NAN	Arginine and proline metabolism	Clostridiales	Order	Firmicutes	Clostridia	Clostridiales				0.0987	0.3305		-0.0281	0.7919		0.1143	0.1722	
24	1349	566552.BIFCAT_00987	COG0282	Acetate kinase	Energy production and conversion [C]	Taurine and hypotaurine metabolism	Bifidobacterium	Genus	Actinobacteria	Actinobacteria	Bifidobacteriales	Bifidobacteriaceae	Bifidobacterium		-0.1206	0.5095		-0.1416	0.4581		0.0747	0.6230	

**25**	1438	657314.CK5_28620	COG2759	Formyltetrahydrofolate synthetase	Nucleotide transport and metabolism [F]	One carbon pool by folate	Blautia obeum	Species	Firmicutes	Clostridia	Clostridiales	Lachnospiraceae	Blautia	Blautia obeum	0.0730	0.5759		-0.0067	0.9608		-0.0331	0.7600	
**26**	1460	657323.CK1_21680	COG4213	ABC-type xylose transport system, periplasmic component	Carbohydrate transport and metabolism [G]	ABC transporters	Clostridiales	Order	Firmicutes	Clostridia	Clostridiales				-0.1260	0.3630		-0.3287	0.0193	*	-0.0120	0.9170	
**27**	1633	759350.BLJ_0360	COG0228	Ribosomal protein S16	Translation, ribosomal structure and biogenesis [J]	Ribosome	Bifidobacterium	Genus	Actinobacteria	Actinobacteria	Bifidobacteriales	Bifidobacteriaceae	Bifidobacterium		0.1637	0.3338		-0.0762	0.6681		0.0195	0.8905	
**28**	1991	BGI-33A_GL0073034	COG0822	NifU homolog involved in Fe-S cluster formation	Posttranslational modification, protein turnover, chaperones [O]	NAN	Ruminococcus bromii	Species	Firmicutes	Clostridia	Clostridiales	Ruminococcaceae	Ruminococcus	Ruminococcus bromii	-0.2243	0.1492		0.1829	0.2626		-0.2537	0.0466	*
**29**	2257	DLF012_GL0017930	COG2873	O-acetylhomoserine/O-acetylserine sulfhydrylase, pyridoxal phosphate-dependent	Amino acid transport and metabolism [E]	Cysteine and methionine metabolism	Bifidobacterium breve	Species	Actinobacteria	Actinobacteria	Bifidobacteriales	Bifidobacteriaceae	Bifidobacterium	Bifidobacterium breve	-0.0690	0.6160		-0.1642	0.2501		0.1016	0.3721	
**30**	2355	DLF012_GL0039573	COG1653	ABC-type glycerol-3-phosphate transport system, periplasmic component	Carbohydrate transport and metabolism [G]	ABC transporters	Clostridiales	Order	Firmicutes	Clostridia	Clostridiales				-0.0592	0.6961		-0.0763	0.6296		-0.0398	0.7520	
**31**	2394	DLF013_GL0024384	COG0054	6,7-dimethyl-8-ribityllumazine synthase (Riboflavin synthase beta chain)	Coenzyme transport and metabolism [H]	Riboflavin metabolism	Clostridiales	Order	Firmicutes	Clostridia	Clostridiales				-0.0152	0.8938		0.1123	0.3427		0.0433	0.6465	
**32**	2592	DLM008_GL0027121	COG0462	Phosphoribosylpyrophosphate synthetase	Nucleotide transport and metabolism [F]	Pentose phosphate pathway	Ruminococcus bromii	Species	Firmicutes	Clostridia	Clostridiales	Ruminococcaceae	Ruminococcus	Ruminococcus bromii	-0.0965	0.4428		0.1606	0.2184		-0.1553	0.1326	
**33**	2699	DLM013_GL0037207	COG1196	Chromosome segregation ATPase	Cell cycle control, cell division, chromosome partitioning [D]	NAN	Bacteroides dorei	Species	Bacteroidetes	Bacteroidia	Bacteroidales	Bacteroidaceae	Bacteroides	Bacteroides dorei	-0.0250	0.9415		0.0846	0.8117		0.3468	0.2147	
**34**	2780	DLM016_GL0028957	COG1592	Rubrerythrin	Energy production and conversion [C]	NAN	Ruminococcus bromii	Species	Firmicutes	Clostridia	Clostridiales	Ruminococcaceae	Ruminococcus	Ruminococcus bromii	0.0061	0.9730		0.2300	0.2178		-0.2933	0.0445	*
**35**	2802	DLM017_GL0065099	COG1088	dTDP-D-glucose 4,6-dehydratase	Cell wall/membrane/envelope biogenesis [M]	Amino sugar and nucleotide sugar metabolism	Clostridiales	Order	Firmicutes	Clostridia	Clostridiales				-0.0275	0.8132		0.3145	0.0066	**	-0.0079	0.9348	
**36**	2809	DLM018_GL0016638	COG0092	Ribosomal protein S3	Translation, ribosomal structure and biogenesis [J]	Ribosome	Bifidobacterium	Genus	Actinobacteria	Actinobacteria	Bifidobacteriales	Bifidobacteriaceae	Bifidobacterium		-0.0534	0.7565		0.0279	0.8769		-0.0205	0.8858	
**37**	2834	DLM018_GL0063411	COG1653	ABC-type glycerol-3-phosphate transport system, periplasmic component	Carbohydrate transport and metabolism [G]	ABC transporters	Faecalibacterium prausnitzii	Species	Firmicutes	Clostridia	Clostridiales	Ruminococcaceae	Faecalibacterium	Faecalibacterium prausnitzii	-0.1071	0.5844		-0.2642	0.1924		0.0041	0.9797	
**38**	2839	DLM018_GL0068485	COG1866	Phosphoenolpyruvate carboxykinase, ATP-dependent	Energy production and conversion [C]	Glycolysis / Gluconeogenesis	[Eubacterium] rectale	Species	Firmicutes	Clostridia	Clostridiales	Lachnospiraceae		[Eubacterium] rectale	0.0008	0.9946		0.2306	0.0643		0.1097	0.2750	
**39**	2842	T2D-31A_GL0073439	COG0330	Regulator of protease activity HflC, stomatin/prohibitin superfamily	Posttranslational modification, protein turnover, chaperones [O]	NAN		Genus	Actinobacteria	Actinobacteria	Bifidobacteriales	Bifidobacteriaceae	Bifidobacterium		-0.1911	0.1233		-0.0524	0.6899		0.0177	0.8656	
**40**	3357	DOM013_GL0034020	COG0183	Acetyl-CoA acetyltransferase	Lipid transport and metabolism [I]	Fatty acid degradation	Faecalibacterium prausnitzii	Species	Firmicutes	Clostridia	Clostridiales	Ruminococcaceae	Faecalibacterium	Faecalibacterium prausnitzii	0.0686	0.4571		0.0277	0.7748		0.0362	0.6372	
**41**	3872	HT14A_GL0040767	COG0804	Urease alpha subunit	Amino acid transport and metabolism [E]	Purine metabolism	Firmicutes	Phylum	Firmicutes						0.0731	0.5960		0.0369	0.7980		-0.0908	0.4266	
**42**	4187	MH0001_GL0010989	COG0050	Translation elongation factor EF-Tu, a GTPase	Translation, ribosomal structure and biogenesis [J]	Plant-pathogen interaction	Prevotella copri	Species	Bacteroidetes	Bacteroidia	Bacteroidales	Prevotellaceae	Prevotella	Prevotella copri	-0.0873	0.6071		-0.2234	0.2036		-0.0976	0.4879	
**43**	4272	MH0001_GL0015313	COG1145	Ferredoxin	Energy production and conversion [C]	Glycolysis / Gluconeogenesis	Roseburia faecis	Species	Firmicutes	Clostridia	Clostridiales	Lachnospiraceae	Roseburia	Roseburia faecis	-0.1533	0.2943		0.0331	0.8294		-0.2624	0.0267	*
**44**	4357	MH0001_GL0019923	COG1077	Actin-like ATPase involved in cell morphogenesis	Cell cycle control, cell division, chromosome partitioning [D]	NAN	Roseburia faecis	Species	Firmicutes	Clostridia	Clostridiales	Lachnospiraceae	Roseburia	Roseburia faecis	0.0078	0.9307		0.0042	0.9640		-0.0764	0.3011	
**45**	4411	MH0002_GL0000742	COG0035	Uracil phosphoribosyltransferase	Nucleotide transport and metabolism [F]	Pyrimidine metabolism	Subdoligranulum variabile	Species	Firmicutes	Clostridia	Clostridiales	Ruminococcaceae	Subdoligranulum	Subdoligranulum variabile	0.0740	0.2266		-0.0345	0.5925		0.1017	0.0415	*
**46**	4426	MH0002_GL0001930	COG0050	Translation elongation factor EF-Tu, a GTPase	Translation, ribosomal structure and biogenesis [J]	Plant-pathogen interaction	Clostridiales	Species	Firmicutes	Clostridia	Clostridiales	Ruminococcaceae	Subdoligranulum	Subdoligranulum variabile	0.1707	0.2065		-0.0353	0.8045		0.1164	0.3010	
**47**	4427	MH0002_GL0001931	COG0480	Translation elongation factor EF-G, a GTPase	Translation, ribosomal structure and biogenesis [J]	NAN	Clostridiales	Species	Firmicutes	Clostridia	Clostridiales	Clostridiaceae	Butyricicoccus	Butyricicoccus pullicaecorum	0.1020	0.5591		-0.0235	0.8976		0.0020	0.9888	
**48**	4478	MH0002_GL0005640	COG0112	Glycine/serine hydroxymethyltransferase	Amino acid transport and metabolism [E]	Glycine, serine and threonine metabolism	Subdoligranulum variabile	Species	Firmicutes	Clostridia	Clostridiales	Ruminococcaceae	Subdoligranulum	Subdoligranulum variabile	0.0264	0.7170		-0.0417	0.5830		0.0444	0.4611	

**49**	4487	MH0002_GL0005969	COG0151	Phosphoribosylamine-glycine ligase	Nucleotide transport and metabolism [F]	Purine metabolism	Clostridiales	Order	Firmicutes	Clostridia	Clostridiales				-0.0374	0.5771		0.0338	0.6298		-0.0159	0.7748	
**50**	4489	MH0002_GL0006301			NAN	NAN	Roseburia faecis	Species	Firmicutes	Clostridia	Clostridiales	Lachnospiraceae	Roseburia	Roseburia faecis	0.0837	0.7477		0.0512	0.8505		0.1502	0.4856	
**51**	4512	MH0002_GL0008497	COG2025	Electron transfer flavoprotein, alpha subunit	Energy production and conversion [C]	Nitrogen metabolism	Subdoligranulum variabile	Species	Firmicutes	Clostridia	Clostridiales	Ruminococcaceae	Subdoligranulum	Subdoligranulum variabile	0.2099	0.1162		0.0649	0.6466		0.0402	0.7214	
**52**	4569	MH0002_GL0013621			NAN	NAN	Alistipes putredinis	Species	Bacteroidetes	Bacteroidia	Bacteroidales	Rikenellaceae	Alistipes	Alistipes putredinis	-0.1092	0.2866		-0.0396	0.7127		0.0453	0.5965	
**53**	4632	MH0002_GL0018942	COG0085	DNA-directed RNA polymerase, beta subunit/140 kD subunit	Transcription [K]	Purine metabolism	Bacteria	Species	Actinobacteria	Coriobacteriia	Coriobacteriales	Coriobacteriaceae	Coriobacterium	Coriobacterium glomerans	-0.0088	0.9194		-0.0362	0.6902		0.0114	0.8748	
**54**	4666	MH0002_GL0021758	COG0138	AICAR transformylase/IMP cyclohydrolase PurH	Nucleotide transport and metabolism [F]	Purine metabolism	Clostridiales	Order	Firmicutes	Clostridia	Clostridiales				-0.1429	0.2041		0.1654	0.1581		0.0626	0.5062	
**55**	4706	MH0002_GL0025398	COG0086	DNA-directed RNA polymerase, beta' subunit/160 kD subunit	Transcription [K]	Purine metabolism	Clostridiales	Order	Firmicutes	Clostridia	Clostridiales				0.0097	0.9441		0.0709	0.6219		-0.0120	0.9166	
**56**	4746	MH0002_GL0027835	COG3968	Glutamine synthetase type III	Amino acid transport and metabolism [E]	Alanine, aspartate and glutamate metabolism	Ruminococcaceae	Species	Firmicutes	Clostridia	Clostridiales	Ruminococcaceae	Subdoligranulum	Subdoligranulum variabile	0.0773	0.6161		-0.0079	0.9608		0.0218	0.8650	
**57**	4759	MH0002_GL0028993			NAN	NAN	Bacteria	Superkingdom							0.1161	0.4785		-0.3293	0.0491	*	0.0115	0.9328	
**58**	4801	MH0002_GL0031887	COG0166	Glucose-6-phosphate isomerase	Carbohydrate transport and metabolism [G]	Glycolysis / Gluconeogenesis	Ruminococcaceae	Species	Firmicutes	Clostridia	Clostridiales	Ruminococcaceae	Subdoligranulum	Subdoligranulum variabile	0.0639	0.5214		0.1174	0.2566		0.0535	0.5179	
**59**	4811	MH0002_GL0032818	COG3842	ABC-type Fe3+/spermidine/putrescine transport systems, ATPase components	Amino acid transport and metabolism [E]	ABC transporters	Clostridiales	Order	Firmicutes	Clostridia	Clostridiales				-0.0415	0.8248		0.1793	0.3571		-0.1526	0.3236	
**60**	4862	MH0002_GL0037571	COG0280	Phosphotransacetylase	Energy production and conversion [C]	Taurine and hypotaurine metabolism	Subdoligranulum variabile	Species	Firmicutes	Clostridia	Clostridiales	Ruminococcaceae	Subdoligranulum	Subdoligranulum variabile	0.0972	0.1886		0.0623	0.4233		0.0437	0.4799	
**61**	4868	MH0002_GL0038227	COG2885	Outer membrane protein OmpA and related peptidoglycan-associated (lipo)proteins	Cell wall/membrane/envelope biogenesis [M]	NAN	Alistipes putredinis	Species	Bacteroidetes	Bacteroidia	Bacteroidales	Rikenellaceae	Alistipes	Alistipes putredinis	0.0250	0.8210		-0.0714	0.5348		0.0740	0.4170	
**62**	4871	MH0002_GL0038347	COG0297	Glycogen synthase	Carbohydrate transport and metabolism [G]	Galactose metabolism	Ruminococcaceae	Species	Firmicutes	Clostridia	Clostridiales	Ruminococcaceae	Subdoligranulum	Subdoligranulum variabile	0.1686	0.1444		-0.1336	0.2709		0.1812	0.0563	
**63**	4872	MH0002_GL0038348	COG0448	ADP-glucose pyrophosphorylase	Carbohydrate transport and metabolism [G]	Starch and sucrose metabolism	Clostridiales	Species	Firmicutes	Clostridia	Clostridiales	Lachnospiraceae		Lachnospiraceae bacterium COE1	0.0714	0.4573		0.0224	0.8243		0.0086	0.9142	
**64**	4873	MH0002_GL0038349	COG0448	ADP-glucose pyrophosphorylase	Carbohydrate transport and metabolism [G]	Starch and sucrose metabolism	Clostridiales	Order	Firmicutes	Clostridia	Clostridiales				0.0555	0.5834		0.0925	0.3799		0.0630	0.4522	
**65**	4889	MH0002_GL0039425	COG1362	Aspartyl aminopeptidase	Amino acid transport and metabolism [E]	NAN	[Eubacterium] rectale	Species	Firmicutes	Clostridia	Clostridiales	Lachnospiraceae		[Eubacterium] rectale	-0.1792	0.1552		0.1380	0.2976		0.2006	0.0526	
**66**	4911	MH0002_GL0040561	COG4799	Acetyl-CoA carboxylase, carboxyltransferase component	Lipid transport and metabolism [I]	Fatty acid biosynthesis	Subdoligranulum variabile	Species	Firmicutes	Clostridia	Clostridiales	Ruminococcaceae	Subdoligranulum	Subdoligranulum variabile	0.2331	0.0564		0.0027	0.9835		0.1426	0.1645	
**67**	4914	MH0002_GL0040734	COG1866	Phosphoenolpyruvate carboxykinase, ATP-dependent	Energy production and conversion [C]	Glycolysis / Gluconeogenesis	Lachnospiraceae	Family	Firmicutes	Clostridia	Clostridiales	Lachnospiraceae			0.0880	0.2807		0.0483	0.5726		-0.0137	0.8406	
**68**	4915	MH0002_GL0040735	COG3957	Phosphoketolase	Carbohydrate transport and metabolism [G]	Pentose phosphate pathway	Mycobacterium bohemicum	Species	Actinobacteria	Actinobacteria	Corynebacteriales	Mycobacteriaceae	Mycobacterium	Mycobacterium bohemicum	0.0377	0.8670		-0.1687	0.4720		0.0930	0.6186	
**69**	4916	MH0002_GL0040736	COG2407	L-fucose isomerase or related protein	Carbohydrate transport and metabolism [G]	Fructose and mannose metabolism	Subdoligranulum variabile	Species	Firmicutes	Clostridia	Clostridiales	Ruminococcaceae	Subdoligranulum	Subdoligranulum variabile	0.0358	0.8703		0.3352	0.1374		0.1917	0.2885	
**70**	4977	MH0002_GL0044927	COG0522	Ribosomal protein S4 or related protein	Translation, ribosomal structure and biogenesis [J]	Ribosome biogenesis in eukaryotes	Subdoligranulum variabile	Species	Firmicutes	Clostridia	Clostridiales	Ruminococcaceae	Subdoligranulum	Subdoligranulum variabile	0.0299	0.7466		0.1274	0.1823		-0.0758	0.3209	
**71**	4995	MH0002_GL0047744			NAN	NAN	Bacteria	Superkingdom							0.1615	0.2173		-0.1532	0.2631		-0.0825	0.4504	
**72**	5031	MH0002_GL0050527	COG0081	Ribosomal protein L1	Translation, ribosomal structure and biogenesis [J]	Ribosome	Ruminococcaceae	Species	Firmicutes	Clostridia	Clostridiales	Ruminococcaceae	Faecalibacterium	Faecalibacterium prausnitzii	-0.0103	0.9322		0.0072	0.9549		-0.0454	0.6514	
**73**	5035	MH0002_GL0050697	COG1726	Na+-transporting NADH:ubiquinone oxidoreductase, subunit NqrA	Energy production and conversion [C]	NAN	Bacteroides	Genus	Bacteroidetes	Bacteroidia	Bacteroidales	Bacteroidaceae	Bacteroides		0.2761	0.0476	*	-0.1717	0.2460		0.0991	0.4014	
**74**	5041	MH0002_GL0050870	COG1048	Aconitase A	Energy production and conversion [C]	Citrate cycle (TCA cycle)	Clostridiales	Order	Firmicutes	Clostridia	Clostridiales				-0.0119	0.9179		0.2212	0.0602		0.0061	0.9493	
**75**	5043	MH0002_GL0050951	COG1145	Ferredoxin	Energy production and conversion [C]	Glycolysis / Gluconeogenesis	Ruminococcaceae	Species	Firmicutes	Clostridia	Clostridiales	Ruminococcaceae	Faecalibacterium	Faecalibacterium prausnitzii	-0.0111	0.9463		0.2329	0.1718		-0.0945	0.4894	
**76**	5065	MH0002_GL0053021	COG0055	FoF1-type ATP synthase, beta subunit	Energy production and conversion [C]	Oxidative phosphorylation	Clostridiales	Order	Firmicutes	Clostridia	Clostridiales				-0.1119	0.2311		0.0254	0.7968		-0.0437	0.5761	

**77**	5107	MH0002_GL0057193	COG1264	Phosphotransferase system IIB components	Carbohydrate transport and metabolism [G]	Glycolysis / Gluconeogenesis	Clostridiales	Order	Firmicutes	Clostridia	Clostridiales				0.0838	0.3612		0.0649	0.4993		0.0767	0.3139	
**78**	5172	MH0002_GL0063099	COG0423	Glycyl-tRNA synthetase (class II)	Translation, ribosomal structure and biogenesis [J]	Aminoacyl-tRNA biosynthesis	Clostridiales	Order	Firmicutes	Clostridia	Clostridiales				0.0410	0.6024		0.0783	0.3389		-0.0525	0.4205	
**79**	5239	MH0002_GL0070693	COG2182	Maltose-binding periplasmic protein MalE	Carbohydrate transport and metabolism [G]	ABC transporters	Clostridiales bacterium CHKCI001	Species	Firmicutes	Clostridia	Clostridiales			Clostridiales bacterium CHKCI001	0.0576	0.7624		-0.2211	0.2633		0.1963	0.2106	
**80**	5254	MH0002_GL0071847	COG4206	Outer membrane cobalamin receptor protein	Coenzyme transport and metabolism [H]	NAN	Bacteroides	Genus	Bacteroidetes	Bacteroidia	Bacteroidales	Bacteroidaceae	Bacteroides		0.3177	0.0608		-0.1304	0.4697		-0.2552	0.0700	
**81**	5256	MH0002_GL0072081	COG0203	Ribosomal protein L17	Translation, ribosomal structure and biogenesis [J]	Ribosome	Clostridiales	Order	Firmicutes	Clostridia	Clostridiales				0.0679	0.5023		0.1783	0.0868		0.0370	0.6607	
**82**	5311	MH0002_GL0075003	COG1472	Periplasmic beta-glucosidase and related glycosidases	Carbohydrate transport and metabolism [G]	Cyanoamino acid metabolism	Bacteria	Superkingdom							0.1080	0.3414		-0.0691	0.5616		0.2438	0.0070	**
**83**	5329	MH0002_GL0076888	COG0457	Tetratricopeptide (TPR) repeat	General function prediction only [R]	NAN	Bacteroides	Genus	Bacteroidetes	Bacteroidia	Bacteroidales	Bacteroidaceae	Bacteroides		0.2289	0.1106		-0.0651	0.6688		0.1154	0.3378	
**84**	5354	MH0003_GL0002731	COG0172	Seryl-tRNA synthetase	Translation, ribosomal structure and biogenesis [J]	Aminoacyl-tRNA biosynthesis	Ruminococcus bromii	Species	Firmicutes	Clostridia	Clostridiales	Ruminococcaceae	Ruminococcus	Ruminococcus bromii	-0.0866	0.3030		0.0220	0.8032		-0.1393	0.0419	*
**85**	5385	MH0003_GL0005194	COG0050	Translation elongation factor EF-Tu, a GTPase	Translation, ribosomal structure and biogenesis [J]	Plant-pathogen interaction	Ruminococcus bromii	Species	Firmicutes	Clostridia	Clostridiales	Ruminococcaceae	Ruminococcus	Ruminococcus bromii	-0.3269	0.0862		0.2519	0.2094		-0.0924	0.5655	
**86**	5386	MH0003_GL0005195	COG0480	Translation elongation factor EF-G, a GTPase	Translation, ribosomal structure and biogenesis [J]	NAN	Ruminococcus bromii	Species	Firmicutes	Clostridia	Clostridiales	Ruminococcaceae	Ruminococcus	Ruminococcus bromii	-0.2081	0.2782		0.2150	0.2836		-0.2248	0.1559	
**87**	5388	MH0003_GL0005201	COG2182	Maltose-binding periplasmic protein MalE	Carbohydrate transport and metabolism [G]	ABC transporters	Ruminococcus bromii	Species	Firmicutes	Clostridia	Clostridiales	Ruminococcaceae	Ruminococcus	Ruminococcus bromii	-0.4039	0.0884		0.0662	0.7929		-0.3760	0.0548	
**88**	5421	MH0142_GL0051559	COG3033	Tryptophanase	Amino acid transport and metabolism [E]	Tyrosine metabolism	Firmicutes	Phylum	Firmicutes						-0.1553	0.2307		0.0581	0.6704		-0.1041	0.3348	
**89**	5428	MH0003_GL0009350	COG0057	Glyceraldehyde-3-phosphate dehydrogenase/erythrose-4-phosphate dehydrogenase	Carbohydrate transport and metabolism [G]	Glycolysis / Gluconeogenesis	Ruminococcus bromii	Species	Firmicutes	Clostridia	Clostridiales	Ruminococcaceae	Ruminococcus	Ruminococcus bromii	-0.1741	0.2177		0.0973	0.5127		-0.0745	0.5279	
**90**	5429	MH0003_GL0009354	COG0297	Glycogen synthase	Carbohydrate transport and metabolism [G]	Galactose metabolism	Ruminococcus bromii	Species	Firmicutes	Clostridia	Clostridiales	Ruminococcaceae	Ruminococcus	Ruminococcus bromii	-0.2620	0.0468	*	0.1277	0.3630		-0.2237	0.0405	*
**91**	5459	MH0003_GL0013354	COG0334	Glutamate dehydrogenase/leucine dehydrogenase	Amino acid transport and metabolism [E]	Alanine, aspartate and glutamate metabolism	Bacteroides	Genus	Bacteroidetes	Bacteroidia	Bacteroidales	Bacteroidaceae	Bacteroides		0.1398	0.3010		-0.0864	0.5426		0.0949	0.3989	
**92**	5484	MH0003_GL0018734	COG0366	Glycosidase	Carbohydrate transport and metabolism [G]	Galactose metabolism	Ruminococcus bromii	Species	Firmicutes	Clostridia	Clostridiales	Ruminococcaceae	Ruminococcus	Ruminococcus bromii	-0.2194	0.2544		0.2806	0.1611		-0.1903	0.2333	
**93**	5536	MH0003_GL0028371	COG1882	Pyruvate-formate lyase	Energy production and conversion [C]	Pyruvate metabolism	Ruminococcus bromii	Species	Firmicutes	Clostridia	Clostridiales	Ruminococcaceae	Ruminococcus	Ruminococcus bromii	-0.1506	0.3624		-0.0183	0.9159		-0.2467	0.0679	
**94**	5553	MH0003_GL0030501	COG1454	Alcohol dehydrogenase, class IV	Energy production and conversion [C]	Glycolysis / Gluconeogenesis	Ruminococcus bromii	Species	Firmicutes	Clostridia	Clostridiales	Ruminococcaceae	Ruminococcus	Ruminococcus bromii	-0.3486	0.0462	*	0.2305	0.2141		-0.2126	0.1479	
**95**	5555	MH0003_GL0030511	COG0845	Multidrug efflux pump subunit AcrA (membrane-fusion protein)	Defense mechanisms [V]	Purine metabolism	Ruminococcus bromii	Species	Firmicutes	Clostridia	Clostridiales	Ruminococcaceae	Ruminococcus	Ruminococcus bromii	-0.2044	0.1548		0.1988	0.1861		-0.2967	0.0105	*
**96**	5557	MH0003_GL0030541	COG0047	Phosphoribosylformylglycinamidine (FGAM) synthase, glutamine amidotransferase domain	Nucleotide transport and metabolism [F]	Purine metabolism	Ruminococcus bromii	Species	Firmicutes	Clostridia	Clostridiales	Ruminococcaceae	Ruminococcus	Ruminococcus bromii	-0.3405	0.0302	*	0.1692	0.3138		-0.3307	0.0102	*
**97**	5650	MH0003_GL0042541	COG3637	Opacity protein and related surface antigens	Cell wall/membrane/envelope biogenesis [M]	NAN	Bacteroides	Genus	Bacteroidetes	Bacteroidia	Bacteroidales	Bacteroidaceae	Bacteroides		0.4331	0.0019	**	-0.2153	0.1591		0.1614	0.1848	
**98**	5652	MH0003_GL0042613	COG1932	Phosphoserine aminotransferase	Coenzyme transport and metabolism [H]	Glycine, serine and threonine metabolism	Ruminococcus bromii	Species	Firmicutes	Clostridia	Clostridiales	Ruminococcaceae	Ruminococcus	Ruminococcus bromii	-0.2645	0.0424	*	0.1631	0.2392		-0.1487	0.1755	
**99**	5672	MH0003_GL0044262	COG0058	Glucan phosphorylase	Carbohydrate transport and metabolism [G]	Starch and sucrose metabolism	Ruminococcus bromii	Species	Firmicutes	Clostridia	Clostridiales	Ruminococcaceae	Ruminococcus	Ruminococcus bromii	-0.2901	0.0676		0.1613	0.3382		-0.2001	0.1315	
**100**	5730	MH0003_GL0051189	COG0459	Chaperonin GroEL (HSP60 family)	Posttranslational modification, protein turnover, chaperones [O]	RNA degradation	Ruminococcus bromii	Species	Firmicutes	Clostridia	Clostridiales	Ruminococcaceae	Ruminococcus	Ruminococcus bromii	-0.3617	0.1086		-0.0570	0.8116		-0.3126	0.0944	
**101**	5748	MH0003_GL0052996			NAN	NAN	Bacteroides	Genus	Bacteroidetes	Bacteroidia	Bacteroidales	Bacteroidaceae	Bacteroides		0.4337	0.0223	*	-0.2067	0.3102		0.1546	0.3399	

**102**	5795	MH0003_GL0062474	COG0166	Glucose-6-phosphate isomerase	Carbohydrate transport and metabolism [G]	Glycolysis / Gluconeogenesis	Ruminococcus bromii	Species	Firmicutes	Clostridia	Clostridiales	Ruminococcaceae	Ruminococcus	Ruminococcus bromii	-0.0614	0.6856		-0.0743	0.6389		-0.2862	0.0185	*
**103**	5840	MH0003_GL0068286	COG0094	Ribosomal protein L5	Translation, ribosomal structure and biogenesis [J]	Ribosome	Faecalibacterium prausnitzii	Species	Firmicutes	Clostridia	Clostridiales	Ruminococcaceae	Faecalibacterium	Faecalibacterium prausnitzii	-0.1363	0.1685		-0.1217	0.2408		-0.0110	0.8945	
**104**	5939	MH0003_GL0080136	COG0059	Ketol-acid reductoisomerase	Coenzyme transport and metabolism [H]	Valine, leucine and isoleucine biosynthesis	Ruminococcus bromii	Species	Firmicutes	Clostridia	Clostridiales	Ruminococcaceae	Ruminococcus	Ruminococcus bromii	-0.3493	0.0520		-0.1481	0.4395		0.1131	0.4576	
**105**	5952	MH0003_GL0082437	COG4206	Outer membrane cobalamin receptor protein	Coenzyme transport and metabolism [H]	NAN	Bacteroides	Genus	Bacteroidetes	Bacteroidia	Bacteroidales	Bacteroidaceae	Bacteroides		0.2868	0.2130		-0.3044	0.2055		0.1337	0.4872	
**106**	5959	MH0003_GL0083869	COG3842	ABC-type Fe3+/spermidine/putrescine transport systems, ATPase components	Amino acid transport and metabolism [E]	ABC transporters	Ruminococcus bromii	Species	Firmicutes	Clostridia	Clostridiales	Ruminococcaceae	Ruminococcus	Ruminococcus bromii	-0.0482	0.5803		0.1124	0.2131		-0.0994	0.1646	
**107**	5986	O2.UC48-0_GL0114931	COG0329	Dihydrodipicolinate synthase/N-acetylneuraminate lyase	Cell wall/membrane/envelope biogenesis [M]	Lysine biosynthesis	Ruminococcus bromii	Species	Firmicutes	Clostridia	Clostridiales	Ruminococcaceae	Ruminococcus	Ruminococcus bromii	-0.3028	0.0177	*	0.0721	0.6011		-0.2028	0.0589	
**108**	6008	MH0003_GL0091064	COG1884	Methylmalonyl-CoA mutase, N-terminal domain/subunit	Lipid transport and metabolism [I]	Valine, leucine and isoleucine degradation	Dialister invisus	Species	Firmicutes	Negativicutes	Veillonellales	Veillonellaceae	Dialister	Dialister invisus	0.0022	0.9881		0.0931	0.5420		-0.1216	0.3144	
**109**	6127	MH0003_GL0109380			NAN	NAN	Bacteroides	Genus	Bacteroidetes	Bacteroidia	Bacteroidales	Bacteroidaceae	Bacteroides		0.3243	0.0665		-0.2762	0.1375		0.0100	0.9466	
**110**	6649	MH0006_GL0003713	COG0493	NADPH-dependent glutamate synthase beta chain or related oxidoreductase	General function prediction only [R]	Alanine, aspartate and glutamate metabolism	Ruminococcus bromii	Species	Firmicutes	Clostridia	Clostridiales	Ruminococcaceae	Ruminococcus	Ruminococcus bromii	-0.2377	0.0602		0.3133	0.0161	*	-0.1706	0.1063	
**111**	6834	MH0006_GL0056289	COG0493	NADPH-dependent glutamate synthase beta chain or related oxidoreductase	General function prediction only [R]	Alanine, aspartate and glutamate metabolism	Ruminococcus sp. 5_1_39BFAA	Species	Firmicutes	Clostridia	Clostridiales	Ruminococcaceae	Ruminococcus	Ruminococcus sp. 5_1_39BFAA	-0.0879	0.3347		-0.0647	0.4984		-0.0013	0.9860	
**112**	6872	V1.UC53-0_GL0115256	COG0205	6-phosphofructokinase	Carbohydrate transport and metabolism [G]	Glycolysis / Gluconeogenesis	Clostridiales	Order	Firmicutes	Clostridia	Clostridiales				0.1572	0.1030		-0.0620	0.5443		0.0242	0.7660	
**113**	6967	MH0006_GL0091516	COG0126	3-phosphoglycerate kinase	Carbohydrate transport and metabolism [G]	Glycolysis / Gluconeogenesis	Firmicutes	Phylum	Firmicutes						0.1747	0.0855		0.0396	0.7143		-0.0964	0.2580	
**114**	6984	MH0006_GL0095460	COG0205	6-phosphofructokinase	Carbohydrate transport and metabolism [G]	Glycolysis / Gluconeogenesis	Clostridiales	Order	Firmicutes	Clostridia	Clostridiales				0.0951	0.2676		-0.1367	0.1243		0.0593	0.4065	
**115**	6996	MH0006_GL0098674	COG1129	ABC-type sugar transport system, ATPase component	Carbohydrate transport and metabolism [G]	ABC transporters	Clostridiales	Order	Firmicutes	Clostridia	Clostridiales				0.1120	0.2371		-0.0207	0.8357		0.0142	0.8579	
**116**	7025	MH0006_GL0106010	COG1614	CO dehydrogenase/acetyl-CoA synthase beta subunit	Energy production and conversion [C]	Methane metabolism	Clostridiales	Order	Firmicutes	Clostridia	Clostridiales				0.0516	0.7746		-0.2932	0.1132		0.1373	0.3556	
**117**	7087	MH0006_GL0124546	COG0166	Glucose-6-phosphate isomerase	Carbohydrate transport and metabolism [G]	Glycolysis / Gluconeogenesis	Clostridiales	Order	Firmicutes	Clostridia	Clostridiales				-0.1256	0.1697		-0.0526	0.5858		-0.1290	0.0872	
**118**	7139	MH0006_GL0140949	COG1629	Outer membrane receptor proteins, mostly Fe transport	Inorganic ion transport and metabolism [P]	NAN	Alistipes putredinis	Species	Bacteroidetes	Bacteroidia	Bacteroidales	Rikenellaceae	Alistipes	Alistipes putredinis	-0.0788	0.5288		0.0280	0.8310		-0.0067	0.9483	
**119**	7197	MH0006_GL0156531	COG0696	Phosphoglycerate mutase (BPG-independent, AlkP superfamily)	Carbohydrate transport and metabolism [G]	Glycolysis / Gluconeogenesis	Ruminococcus bromii	Species	Firmicutes	Clostridia	Clostridiales	Ruminococcaceae	Ruminococcus	Ruminococcus bromii	-0.1184	0.1870		0.0672	0.4772		-0.0893	0.2314	
**120**	7272	MH0006_GL0173348	COG0448	ADP-glucose pyrophosphorylase(3043)	Carbohydrate transport and metabolism [G]	Starch and sucrose metabolism	Clostridiales	Order	Firmicutes	Clostridia	Clostridiales				-0.0071	0.9379		-0.1049	0.2694		-0.0057	0.9402	
**121**	7287	MH0006_GL0174816	COG1712	Predicted dinucleotide-utilizing enzyme	General function prediction only [R]	Lysine biosynthesis	Clostridiales	Order	Firmicutes	Clostridia	Clostridiales				0.0770	0.4336		-0.0112	0.9135		-0.1384	0.0851	
**122**	7291	MH0006_GL0176047	COG3063	Tfp pilus assembly protein PilF	Extracellular structures [W]	NAN	Parabacteroides	Genus	Bacteroidetes	Bacteroidia	Bacteroidales	Porphyromonadaceae	Parabacteroides		0.2263	0.1125		-0.2003	0.1808		0.0463	0.6998	
**123**	7429	MH0006_GL0211764	COG0202	DNA-directed RNA polymerase, alpha subunit/40 kD subunit	Transcription [K]	Purine metabolism	Ruminococcus bromii	Species	Firmicutes	Clostridia	Clostridiales	Ruminococcaceae	Ruminococcus	Ruminococcus bromii	-0.1161	0.3436		0.0158	0.9022		-0.0792	0.4370	
**124**	7431	MH0006_GL0212607	COG2069	CO dehydrogenase/acetyl-CoA synthase delta subunit (corrinoid Fe-S protein)	Energy production and conversion [C]	Methane metabolism	Clostridiales	Order	Firmicutes	Clostridia	Clostridiales				0.1924	0.2311		0.0379	0.8229		0.0789	0.5568	
**125**	7583	MH0008_GL0014349	COG0091	Ribosomal protein L22	Translation, ribosomal structure and biogenesis [J]	Ribosome	Faecalibacterium prausnitzii	Species	Firmicutes	Clostridia	Clostridiales	Ruminococcaceae	Faecalibacterium	Faecalibacterium prausnitzii	0.0181	0.8579		0.0340	0.7473		0.0331	0.6929	
**126**	7656	MH0008_GL0044786	COG3181	Tripartite-type tricarboxylate transporter, receptor component TctC(1072)	Energy production and conversion [C]	Two-component system	Clostridiales	Order	Firmicutes	Clostridia	Clostridiales				0.0195	0.8405		-0.0588	0.5616		0.0037	0.9633	
**127**	7789	MH0009_GL0049397	COG0091	Ribosomal protein L22	Translation, ribosomal structure and biogenesis [J]	Ribosome	Clostridiales	Order	Firmicutes	Clostridia	Clostridiales				0.1535	0.1238		-0.0581	0.5823		-0.0287	0.7329	

**128**	7922	MH0010_GL0016261	COG0539	Ribosomal protein S1	Translation, ribosomal structure and biogenesis [J]	Terpenoid backbone biosynthesis	Faecalibacterium prausnitzii	Species	Firmicutes	Clostridia	Clostridiales	Ruminococcaceae	Faecalibacterium	Faecalibacterium prausnitzii	-0.1221	0.5665		-0.0070	0.9751		-0.1553	0.3784	
**129**	7999	MH0010_GL0038237	COG0800	2-keto-3-deoxy-6-phosphogluconate aldolase	Carbohydrate transport and metabolism [G]	Pentose phosphate pathway	Faecalibacterium prausnitzii	Species	Firmicutes	Clostridia	Clostridiales	Ruminococcaceae	Faecalibacterium	Faecalibacterium prausnitzii	-0.3253	0.1083		-0.2043	0.3393		0.0643	0.7067	
**130**	8219	MH0011_GL0036474	COG1028	NAD(P)-dependent dehydrogenase, short-chain alcohol dehydrogenase family	General function prediction only [R]	NAN	Faecalibacterium prausnitzii	Species	Firmicutes	Clostridia	Clostridiales	Ruminococcaceae	Faecalibacterium	Faecalibacterium prausnitzii	-0.2189	0.2481		-0.0580	0.7715		0.0209	0.8953	
**131**	8474	MH0011_GL0094125	COG0203	Ribosomal protein L17	Translation, ribosomal structure and biogenesis [J]	Ribosome	Lachnospiraceae	Family	Firmicutes	Clostridia	Clostridiales	Lachnospiraceae			-0.1280	0.0519		0.0151	0.8306		-0.0286	0.6094	
**132**	8579	MH0012_GL0011046	COG3842	ABC-type Fe3+/spermidine/putrescine transport systems, ATPase components	Amino acid transport and metabolism [E]	ABC transporters	Ruminococcaceae	Family	Firmicutes	Clostridia	Clostridiales	Ruminococcaceae			0.1519	0.3006		0.0556	0.7187		0.0961	0.4314	
**133**	8672	MH0012_GL0035930	COG0834	ABC-type amino acid transport/signal transduction system, periplasmic component/domain	Signal transduction mechanisms [T]	NAN	Ruminococcus bromii	Species	Firmicutes	Clostridia	Clostridiales	Ruminococcaceae	Ruminococcus	Ruminococcus bromii	-0.0379	0.7048		0.0337	0.7472		-0.1090	0.1852	
**134**	8811	MH0012_GL0079061	COG0282	Acetate kinase	Energy production and conversion [C]	Taurine and hypotaurine metabolism	Clostridium pasteurianum	Species	Firmicutes	Clostridia	Clostridiales	Clostridiaceae	Clostridium	Clostridium pasteurianum	-0.0406	0.7914		0.2054	0.1956		0.1570	0.2134	
**135**	8942	MH0012_GL0119203	COG1362	Aspartyl aminopeptidase	Amino acid transport and metabolism [E]	NAN	Ruminococcus bromii	Species	Firmicutes	Clostridia	Clostridiales	Ruminococcaceae	Ruminococcus	Ruminococcus bromii	-0.0384	0.7097		-0.0301	0.7800		-0.0004	0.9964	
**136**	8957	MH0012_GL0123603			NAN	NAN	Eubacterium	Genus	Firmicutes	Clostridia	Clostridiales	Eubacteriaceae	Eubacterium		0.3373	0.0570		-0.2464	0.1886		0.1568	0.2942	
**137**	9003	MH0012_GL0138305	COG0443	Molecular chaperone DnaK (HSP70)	Posttranslational modification, protein turnover, chaperones [O]	RNA degradation	Subdoligranulum variabile	Species	Firmicutes	Clostridia	Clostridiales	Ruminococcaceae	Subdoligranulum	Subdoligranulum variabile	0.1323	0.2943		-0.2606	0.0437	*	0.1715	0.0978	
**138**	9043	MH0012_GL0150149	COG1653	ABC-type glycerol-3-phosphate transport system, periplasmic component	Carbohydrate transport and metabolism [G]	ABC transporters	[Eubacterium] rectale	Species	Firmicutes	Clostridia	Clostridiales	Lachnospiraceae		[Eubacterium] rectale	-0.3110	0.1414		-0.1569	0.4823		0.0224	0.8999	
**139**	9087	V1.CD7-0-PN_GL0009048	COG0051	Ribosomal protein S10	Translation, ribosomal structure and biogenesis [J]	Ribosome	Bacteria	Superkingdom							0.0706	0.6092		-0.1322	0.3574		0.0599	0.6010	
**140**	9211	MH0012_GL0193632	COG4799	Acetyl-CoA carboxylase, carboxyltransferase component	Lipid transport and metabolism [I]	Fatty acid biosynthesis	Dialister	Genus	Firmicutes	Negativicutes	Veillonellales	Veillonellaceae	Dialister		0.2398	0.1871		-0.4785	0.0092	**	-0.0941	0.5364	
**141**	9225	MH0012_GL0196811	COG0480	Translation elongation factor EF-G, a GTPase	Translation, ribosomal structure and biogenesis [J]	NAN	Subdoligranulum variabile	Species	Firmicutes	Clostridia	Clostridiales	Ruminococcaceae	Subdoligranulum	Subdoligranulum variabile	0.1288	0.1480		-0.0671	0.4751		0.0323	0.6656	
**142**	9235	MH0012_GL0199057	COG2885	Outer membrane protein OmpA and related peptidoglycan-associated (lipo)proteins	Cell wall/membrane/envelope biogenesis [M]	NAN	Bacteroidales	Order	Bacteroidetes	Bacteroidia	Bacteroidales				0.3213	0.0161	*	-0.2296	0.1067		0.0229	0.8423	
**143**	9331	MH0012_GL0231760	COG1592	Rubrerythrin	Energy production and conversion [C]	NAN	Faecalibacterium prausnitzii	Species	Firmicutes	Clostridia	Clostridiales	Ruminococcaceae	Faecalibacterium	Faecalibacterium prausnitzii	-0.1619	0.4655		-0.2102	0.3634		0.2817	0.1216	
**144**	9336	MH0012_GL0232564	COG4771	Outer membrane receptor for ferrienterochelin and colicins(	Inorganic ion transport and metabolism [P]	NAN	Bacteroides	Genus	Bacteroidetes	Bacteroidia	Bacteroidales	Bacteroidaceae	Bacteroides		-0.1881	0.2899		0.1067	0.5674		0.0724	0.6255	
**145**	9554	MH0014_GL0025905	COG4238	Outer membrane murein-binding lipoprotein Lpp	Cell wall/membrane/envelope biogenesis [M]	NAN	Proteobacteria	Phylum	Proteobacteria						0.0549	0.8310		-0.2609	0.3290		0.3249	0.1227	
**146**	9600	O2.UC49-0_GL0079827	COG1883	Na+-transporting methylmalonyl-CoA/oxaloacetate decarboxylase, beta subunit	Energy production and conversion [C]	Arginine and proline metabolism	Bacteria	Superkingdom							-0.0340	0.7238		0.0433	0.6666		-0.0558	0.4836	
**147**	9717	MH0014_GL0093892	COG0057	Glyceraldehyde-3-phosphate dehydrogenase/erythrose-4-phosphate dehydrogenase	Carbohydrate transport and metabolism [G]	Glycolysis / Gluconeogenesis	Clostridiales	Order	Firmicutes	Clostridia	Clostridiales				0.1998	0.0997		-0.0837	0.5154		0.0687	0.5016	
**148**	9728	MH0014_GL0097483	COG1592	Rubrerythrin	Energy production and conversion [C]	NAN	Clostridiales	Order	Firmicutes	Clostridia	Clostridiales				0.3436	0.0368	*	-0.1042	0.5545		-0.0169	0.9042	
**149**	9731	MH0014_GL0098098			NAN	NAN	Ruminococcus bromii	Species	Firmicutes	Clostridia	Clostridiales	Ruminococcaceae	Ruminococcus	Ruminococcus bromii	-0.1938	0.2174		0.2271	0.1653		-0.1339	0.3060	
**150**	10572	MH0025_GL0075388	COG0510	Thiamine kinase and related kinases	Coenzyme transport and metabolism [H]	NAN	Ruminococcus bromii	Species	Firmicutes	Clostridia	Clostridiales	Ruminococcaceae	Ruminococcus	Ruminococcus bromii	-0.1286	0.3364		0.1861	0.1803		-0.1360	0.2187	

**151**	10738	MH0026_GL0035301	COG0057	Glyceraldehyde-3-phosphate dehydrogenase/erythrose-4-phosphate dehydrogenase(2333)	Carbohydrate transport and metabolism [G]	Glycolysis / Gluconeogenesis	Eubacterium	Genus	Firmicutes	Clostridia	Clostridiales	Eubacteriaceae	Eubacterium		-0.1008	0.4967		0.0283	0.8554		-0.0865	0.4818	
**152**	10969	MH0028_GL0071718			NAN	NAN	Faecalibacterium prausnitzii	Species	Firmicutes	Clostridia	Clostridiales	Ruminococcaceae	Faecalibacterium	Faecalibacterium prausnitzii	-0.2106	0.2057		-0.1081	0.5373		0.1856	0.1785	
**153**	11241	MH0032_GL0024527	COG0090	Ribosomal protein L2(1921)	Translation, ribosomal structure and biogenesis [J]	Ribosome	Dialister succinatiphilus	Species	Firmicutes	Negativicutes	Veillonellales	Veillonellaceae	Dialister	Dialister succinatiphilus	0.2158	0.0489	*	0.0403	0.7311		-0.0625	0.5013	
**154**	11414	MH0037_GL0008359	COG0685	5,10-methylenetetrahydrofolate reductase(1603)	Amino acid transport and metabolism [E]	Cysteine and methionine metabolism	Lachnospiraceae	Family	Firmicutes	Clostridia	Clostridiales	Lachnospiraceae			0.0368	0.7225		0.0647	0.5490		-0.0694	0.4175	
**155**	11463	MH0037_GL0027576			NAN	NAN	Bacteria	Superkingdom							0.1163	0.4429		-0.4093	0.0070	**	0.0263	0.8349	
**156**	11465	MH0037_GL0028219	COG0166	Glucose-6-phosphate isomerase	Carbohydrate transport and metabolism [G]	Glycolysis / Gluconeogenesis	Lachnospiraceae	Family	Firmicutes	Clostridia	Clostridiales	Lachnospiraceae			-0.0239	0.7874		0.0940	0.3076		0.0181	0.8059	
**157**	11699	MH0041_GL0000003	COG0091	Ribosomal protein L22	Translation, ribosomal structure and biogenesis [J]	Ribosome	Bacteria	Superkingdom							-0.0267	0.8180		-0.1963	0.0998		-0.0424	0.6600	
**158**	11766	MH0041_GL0040403	COG1866	Phosphoenolpyruvate carboxykinase, ATP-dependent	Energy production and conversion [C]	Glycolysis / Gluconeogenesis	Roseburia faecis	Species	Firmicutes	Clostridia	Clostridiales	Lachnospiraceae	Roseburia	Roseburia faecis	0.0656	0.6248		0.0282	0.8407		-0.0403	0.7180	
**159**	11804	MH0041_GL0063223	COG0255	Ribosomal protein L29	Translation, ribosomal structure and biogenesis [J]	Ribosome	Bifidobacterium adolescentis	Species	Actinobacteria	Actinobacteria	Bifidobacteriales	Bifidobacteriaceae	Bifidobacterium	Bifidobacterium adolescentis	-0.1044	0.3263		-0.0356	0.7497		-0.0133	0.8810	
**160**	11861	MH0043_GL0046307	COG0334	Glutamate dehydrogenase/leucine dehydrogenase	Amino acid transport and metabolism [E]	Alanine, aspartate and glutamate metabolism	Arthrobacter sp. YC-RL1	Species	Actinobacteria	Actinobacteria	Micrococcales	Micrococcaceae	Arthrobacter	Arthrobacter sp. YC-RL1	-0.1393	0.4309		0.1792	0.3309		-0.1336	0.3621	
**161**	11983	MH0045_GL0023728	COG4206	Outer membrane cobalamin receptor protein	Coenzyme transport and metabolism [H]	NAN	Bacteroides	Genus	Bacteroidetes	Bacteroidia	Bacteroidales	Bacteroidaceae	Bacteroides		0.3176	0.1237		0.0798	0.7153		0.0023	0.9895	
**162**	12181	MH0048_GL0045161	COG0334	Glutamate dehydrogenase/leucine dehydrogenase	Amino acid transport and metabolism [E]	Alanine, aspartate and glutamate metabolism	Eubacterium	Genus	Firmicutes	Clostridia	Clostridiales	Eubacteriaceae	Eubacterium		-0.1984	0.1222		-0.0434	0.7499		0.0429	0.6917	
**163**	12475	MH0053_GL0032330	COG1080	Phosphoenolpyruvate-protein kinase (PTS system EI component in bacteria)	Carbohydrate transport and metabolism [G]	Pyruvate metabolism	Clostridium	Genus	Firmicutes	Clostridia	Clostridiales	Clostridiaceae	Clostridium		-0.1650	0.0657		0.1624	0.0837		-0.1270	0.0889	
**164**	12661	MH0055_GL0011037	COG1960	Acyl-CoA dehydrogenase related to the alkylation response protein AidB	Lipid transport and metabolism [I]	Fatty acid degradation	Coprococcus eutactus	Species	Firmicutes	Clostridia	Clostridiales	Lachnospiraceae	Coprococcus	Coprococcus eutactus	0.0764	0.6200		-0.0467	0.7720		0.1730	0.1715	
**165**	12697	MH0055_GL0031824	COG0050	Translation elongation factor EF-Tu, a GTPase	Translation, ribosomal structure and biogenesis [J]	Plant-pathogen interaction	Faecalibacterium prausnitzii	Species	Firmicutes	Clostridia	Clostridiales	Ruminococcaceae	Faecalibacterium	Faecalibacterium prausnitzii	-0.2946	0.2208		0.1009	0.6906		-0.1743	0.3851	
**166**	12719	MH0055_GL0039831	COG1882	Pyruvate-formate lyase	Energy production and conversion [C]	Pyruvate metabolism	Coprococcus eutactus	Species	Firmicutes	Clostridia	Clostridiales	Lachnospiraceae	Coprococcus	Coprococcus eutactus	0.0808	0.6049		-0.1108	0.4966		0.0153	0.9061	
**167**	13102	MH0060_GL0028240	COG0524	Sugar or nucleoside kinase, ribokinase family	Carbohydrate transport and metabolism [G]	Pentose phosphate pathway	Ruminococcaceae	Family	Firmicutes	Clostridia	Clostridiales	Ruminococcaceae			-0.2444	0.0740		0.0278	0.8489		-0.0641	0.5794	
**168**	13344	MH0062_GL0037172	COG1024	Enoyl-CoA hydratase/carnithine racemase	Lipid transport and metabolism [I]	NAN	Clostridiales	Order	Firmicutes	Clostridia	Clostridiales				-0.1479	0.2572		-0.0122	0.9292		-0.0323	0.7671	
**169**	13357	MH0062_GL0041928	COG0206	Cell division GTPase FtsZ	Cell cycle control, cell division, chromosome partitioning [D]	Cell cycle - Caulobacter	Faecalibacterium prausnitzii	Species	Firmicutes	Clostridia	Clostridiales	Ruminococcaceae	Faecalibacterium	Faecalibacterium prausnitzii	-0.0885	0.2994		0.0151	0.8664		-0.1035	0.1407	
**170**	13391	MH0062_GL0058668	COG4166	ABC-type oligopeptide transport system, periplasmic component	Amino acid transport and metabolism [E]	ABC transporters	Faecalibacterium prausnitzii	Species	Firmicutes	Clostridia	Clostridiales	Ruminococcaceae	Faecalibacterium	Faecalibacterium prausnitzii	-0.3532	0.0314	*	-0.2372	0.1740		0.2760	0.0435	*
**171**	13399	MH0062_GL0061760	COG0052	Ribosomal protein S2	Translation, ribosomal structure and biogenesis [J]	Ribosome	Lachnospiraceae	Family	Firmicutes	Clostridia	Clostridiales	Lachnospiraceae			0.0229	0.8247		0.0705	0.5129		0.0736	0.3894	
**172**	14026	MH0073_GL0056444			NAN	Galactose metabolism	Ruminococcus bromii	Species	Firmicutes	Clostridia	Clostridiales	Ruminococcaceae	Ruminococcus	Ruminococcus bromii	-0.2497	0.0161	*	0.2287	0.0365	*	-0.1350	0.1251	
**173**	14108	MH0076_GL0047189			NAN	NAN	Clostridiales	Order	Firmicutes	Clostridia	Clostridiales				0.1810	0.2808		0.0307	0.8619		0.0859	0.5397	
**174**	14551	MH0086_GL0023405	COG1744	Basic membrane lipoprotein Med, periplasmic binding protein (PBP1-ABC) superfamily	Cell wall/membrane/envelope biogenesis [M]	NAN	Clostridiales	Order	Firmicutes	Clostridia	Clostridiales				-0.0191	0.8481		-0.0336	0.7459		0.0672	0.4138	
**175**	14579	MH0086_GL0029553	COG0517	CBS domain	Signal transduction mechanisms [T]	Purine metabolism	Faecalibacterium prausnitzii	Species	Firmicutes	Clostridia	Clostridiales	Ruminococcaceae	Faecalibacterium	Faecalibacterium prausnitzii	-0.0692	0.4038		-0.0311	0.7203		0.0579	0.3996	

**176**	14619	MH0086_GL0046721	COG1143	Formate hydrogenlyase subunit 6/NADH:ubiquinone oxidoreductase 23 kD subunit (chain I)	Energy production and conversion [C]	Glycolysis / Gluconeogenesis	Ruminococcus	Genus	Firmicutes	Clostridia	Clostridiales	Ruminococcaceae	Ruminococcus		-0.2242	0.2207		0.3307	0.0807		-0.2808	0.0612	
**177**	14632	MH0086_GL0050799	COG1456	CO dehydrogenase/acetyl-CoA synthase gamma subunit (corrinoid Fe-S protein)	Energy production and conversion [C]	Methane metabolism	Clostridiales	Order	Firmicutes	Clostridia	Clostridiales				-0.0113	0.9303		0.0405	0.7640		0.0428	0.6897	
**178**	14652	MH0086_GL0058027	COG4624	Iron only hydrogenase large subunit, C-terminal domain	Energy production and conversion [C]	Oxidative phosphorylation	Clostridiales	Order	Firmicutes	Clostridia	Clostridiales				-0.1459	0.0878		-0.0356	0.6953		-0.1338	0.0584	
**179**	14676	MH0086_GL0071322	COG1882	Pyruvate-formate lyase	Energy production and conversion [C]	Pyruvate metabolism	Clostridiales	Order	Firmicutes	Clostridia	Clostridiales				-0.0362	0.7945		0.1648	0.2520		-0.0013	0.9912	
**180**	14692	MH0086_GL0077208	COG1960	Acyl-CoA dehydrogenase related to the alkylation response protein AidB	Lipid transport and metabolism [I]	Fatty acid degradation	Clostridiales	Order	Firmicutes	Clostridia	Clostridiales				-0.2178	0.2776		0.1838	0.3815		-0.1678	0.3140	
**181**	14751	MH0086_GL0098687	COG4166	ABC-type oligopeptide transport system, periplasmic component	Amino acid transport and metabolism [E]	ABC transporters	Clostridiales	Order	Firmicutes	Clostridia	Clostridiales				0.2809	0.0142	*	0.0440	0.7234		-0.0027	0.9786	
**182**	14763	MH0086_GL0104589	COG1129	ABC-type sugar transport system, ATPase component	Carbohydrate transport and metabolism [G]	NAN	Subdoligranulum variabile	Species	Firmicutes	Clostridia	Clostridiales	Ruminococcaceae	Subdoligranulum	Subdoligranulum variabile	-0.0073	0.9235		-0.0048	0.9515		0.0775	0.2137	
**183**	14788	MH0086_GL0116500	COG0138	AICAR transformylase/IMP cyclohydrolase PurH	Nucleotide transport and metabolism [F]	Purine metabolism	Clostridiales	Order	Firmicutes	Clostridia	Clostridiales				0.1211	0.1887		0.0132	0.8919		0.0534	0.4882	
**184**	14811	MH0087_GL0004799	COG0522	Ribosomal protein S4 or related protein	Translation, ribosomal structure and biogenesis [J]	Ribosome biogenesis in eukaryotes	Clostridiales	Order	Firmicutes	Clostridia	Clostridiales				0.0560	0.6553		0.1054	0.4202		-0.0481	0.6446	
**185**	14833	MH0087_GL0012728	COG4656	Na+-translocating ferredoxin:NAD+ oxidoreductase RNF, RnfC subunit	Energy production and conversion [C]	NAN	Clostridiales	Order	Firmicutes	Clostridia	Clostridiales				-0.0178	0.9057		-0.0462	0.7678		-0.0346	0.7807	
**186**	14837	MH0087_GL0014198	COG1866	Phosphoenolpyruvate carboxykinase, ATP-dependent	Energy production and conversion [C]	Glycolysis / Gluconeogenesis	Clostridiales	Order	Firmicutes	Clostridia	Clostridiales				-0.0048	0.9562		-0.0079	0.9304		-0.0844	0.2371	
**187**	14871	MH0087_GL0031294	COG5263	Glucan-binding domain (YG repeat)	Carbohydrate transport and metabolism [G]	NAN	Clostridium	Genus	Firmicutes	Clostridia	Clostridiales	Clostridiaceae	Clostridium		0.2683	0.2547		-0.1127	0.6491		-0.0622	0.7524	
**188**	14892	MH0087_GL0040866	COG0443	Molecular chaperone DnaK (HSP70)	Posttranslational modification, protein turnover, chaperones [O]	RNA degradation	Clostridiales	Order	Firmicutes	Clostridia	Clostridiales				-0.0332	0.7641		-0.0583	0.6128		-0.1463	0.1050	
**189**	14910	MH0087_GL0049381	COG1454	Alcohol dehydrogenase, class IV	Energy production and conversion [C]	Glycolysis / Gluconeogenesis	Blautia obeum	Species	Firmicutes	Clostridia	Clostridiales	Lachnospiraceae	Blautia	Blautia obeum	0.1691	0.2616		-0.2110	0.1783		-0.0751	0.5503	
**190**	14942	MH0088_GL0009851	COG0280	Phosphotransacetylase	Energy production and conversion [C]	Taurine and hypotaurine metabolism	Lachnospiraceae	Family	Firmicutes	Clostridia	Clostridiales	Lachnospiraceae			-0.0406	0.5491		-0.0068	0.9239		-0.0076	0.8933	
**191**	14967	MH0088_GL0016997	COG0696	Phosphoglycerate mutase (BPG-independent, AlkP superfamily)	Carbohydrate transport and metabolism [G]	Glycolysis / Gluconeogenesis	Clostridiales	Order	Firmicutes	Clostridia	Clostridiales				0.0528	0.6125		-0.0200	0.8546		-0.1243	0.1461	
**192**	15013	MH0088_GL0029088	COG0137	Argininosuccinate synthase	Amino acid transport and metabolism [E]	Alanine, aspartate and glutamate metabolism	Clostridiales	Order	Firmicutes	Clostridia	Clostridiales				0.1337	0.1537		-0.0627	0.5259		0.1342	0.0826	
**193**	15022	MH0088_GL0033424	COG0094	Ribosomal protein L5	Translation, ribosomal structure and biogenesis [J]	Ribosome	Blautia sp. KLE 1732	Species	Firmicutes	Clostridia	Clostridiales	Lachnospiraceae	Blautia	Blautia sp. KLE 1732	-0.0292	0.7494		0.1390	0.1396		-0.0549	0.4678	
**194**	15026	MH0088_GL0034073	COG0480	Translation elongation factor EF-G, a GTPase	Translation, ribosomal structure and biogenesis [J]	NAN	Clostridiales	Order	Firmicutes	Clostridia	Clostridiales				0.1239	0.4038		-0.0759	0.6255		0.0516	0.6765	
**195**	15079	MH0088_GL0054024	COG0136	Aspartate-semialdehyde dehydrogenase	Amino acid transport and metabolism [E]	Glycine, serine and threonine metabolism	Clostridiales	Order	Firmicutes	Clostridia	Clostridiales				-0.0458	0.6560		-0.0948	0.3757		0.0977	0.2492	
**196**	15122	MH0088_GL0066516	COG1454	Alcohol dehydrogenase, class IV	Energy production and conversion [C]	Glycolysis / Gluconeogenesis	Clostridiales	Order	Firmicutes	Clostridia	Clostridiales				0.1164	0.2976		-0.2029	0.0783		0.0372	0.6904	
**197**	15182	MH0088_GL0086499	COG0031	Cysteine synthase	Amino acid transport and metabolism [E]	Glycine, serine and threonine metabolism	Clostridiales	Order	Firmicutes	Clostridia	Clostridiales				0.0353	0.5741		0.0113	0.8632		0.0207	0.6918	
**198**	15257	MH0088_GL0109165	COG0624	Acetylornithine deacetylase/Succinyl-diaminopimelate desuccinylase or related deacylase	Amino acid transport and metabolism [E]	Lysine biosynthesis	Clostridiales	Order	Firmicutes	Clostridia	Clostridiales				0.1374	0.3987		-0.2391	0.1561		0.0976	0.4704	
**199**	15393	MH0089_GL0026063	COG0050	Translation elongation factor EF-Tu, a GTPase	Translation, ribosomal structure and biogenesis [J]	Plant-pathogen interaction	Clostridiales	Order	Firmicutes	Clostridia	Clostridiales				-0.0303	0.7975		-0.0156	0.8992		-0.0708	0.4693	

**200**	15465	MH0089_GL0075669	COG0057	Glyceraldehyde-3-phosphate dehydrogenase/erythrose-4-phosphate dehydrogenase	Carbohydrate transport and metabolism [G]	Glycolysis / Gluconeogenesis	Clostridiales	Order	Firmicutes	Clostridia	Clostridiales				0.0330	0.8084		-0.0404	0.7761		-0.0314	0.7808	
**201**	15627	MH0092_GL0003446	COG0329	Dihydrodipicolinate synthase/N-acetylneuraminate lyase	Cell wall/membrane/envelope biogenesis [M]	Lysine biosynthesis	Faecalibacterium prausnitzii	Species	Firmicutes	Clostridia	Clostridiales	Ruminococcaceae	Faecalibacterium	Faecalibacterium prausnitzii	-0.1306	0.2622		-0.1519	0.2107		-0.0018	0.9855	
**202**	15670	MH0092_GL0036208	COG1653	ABC-type glycerol-3-phosphate transport system, periplasmic component	Carbohydrate transport and metabolism [G]	NAN	Faecalibacterium prausnitzii	Species	Firmicutes	Clostridia	Clostridiales	Ruminococcaceae	Faecalibacterium	Faecalibacterium prausnitzii	-0.6242	0.0245	*	-0.2206	0.4597		0.0156	0.9477	
**203**	15697	MH0092_GL0074084	COG3853	Uncharacterized conserved protein YaaN involved in tellurite resistance	Defense mechanisms [V]	NAN	Bacteria	Species	Firmicutes	Clostridia	Clostridiales	Ruminococcaceae	Faecalibacterium	Faecalibacterium prausnitzii	0.0706	0.4813		-0.0482	0.6457		0.0544	0.5138	
**204**	15723	MH0092_GL0097242	COG0166	Glucose-6-phosphate isomerase	Carbohydrate transport and metabolism [G]	Glycolysis / Gluconeogenesis	Faecalibacterium prausnitzii	Species	Firmicutes	Clostridia	Clostridiales	Ruminococcaceae	Faecalibacterium	Faecalibacterium prausnitzii	-0.1886	0.0772		0.0293	0.7969		0.0446	0.6209	
**205**	16373	MH0101_GL0050789	COG4465	GTP-sensing pleiotropic transcriptional regulator CodY	Transcription [K]	NAN	Clostridiales	Order	Firmicutes	Clostridia	Clostridiales				-0.1086	0.1294		0.0147	0.8465		0.0170	0.7776	
**206**	16397	MH0101_GL0060753	COG0148	Enolase	Carbohydrate transport and metabolism [G]	Glycolysis / Gluconeogenesis	Clostridiales	Order	Firmicutes	Clostridia	Clostridiales				-0.0253	0.8667		0.1147	0.4655		-0.1376	0.2684	
**207**	16733	MH0106_GL0002286	COG1053	Succinate dehydrogenase/fumarate reductase, flavoprotein subunit	Energy production and conversion [C]	Citrate cycle (TCA cycle)	Bacteroides coprocola	Species	Bacteroidetes	Bacteroidia	Bacteroidales	Bacteroidaceae	Bacteroides	Bacteroides coprocola	0.0165	0.9004		-0.1927	0.1556		0.2137	0.0447	*
**208**	16873	MH0108_GL0010146	COG0050	Translation elongation factor EF-Tu, a GTPase	Translation, ribosomal structure and biogenesis [J]	Plant-pathogen interaction	Clostridiales	Order	Firmicutes	Clostridia	Clostridiales				-0.0125	0.9413		-0.0545	0.7576		0.0790	0.5730	
**209**	16887	MH0108_GL0025722	COG0103	Ribosomal protein S9	Translation, ribosomal structure and biogenesis [J]	Ribosome	Faecalibacterium prausnitzii	Species	Firmicutes	Clostridia	Clostridiales	Ruminococcaceae	Faecalibacterium	Faecalibacterium prausnitzii	-0.1344	0.3081		-0.0067	0.9613		0.1602	0.1404	
**210**	16893	T2D-105A_GL0119985	COG0822	NifU homolog involved in Fe-S cluster formation	Posttranslational modification, protein turnover, chaperones [O]	NAN	Bacteroides	Genus	Bacteroidetes	Bacteroidia	Bacteroidales	Bacteroidaceae	Bacteroides		0.1509	0.1628		-0.1898	0.0908		0.0573	0.5275	
**211**	16919	MH0108_GL0057076	COG1250	3-hydroxyacyl-CoA dehydrogenase	Lipid transport and metabolism [I]	Fatty acid degradation	Faecalibacterium prausnitzii	Species	Firmicutes	Clostridia	Clostridiales	Ruminococcaceae	Faecalibacterium	Faecalibacterium prausnitzii	0.0994	0.6176		-0.3667	0.0723		0.3102	0.0551	
**212**	16923	MH0108_GL0062043	COG0092	Ribosomal protein S3	Translation, ribosomal structure and biogenesis [J]	Ribosome	Faecalibacterium prausnitzii	Species	Firmicutes	Clostridia	Clostridiales	Ruminococcaceae	Faecalibacterium	Faecalibacterium prausnitzii	-0.0636	0.6592		0.0141	0.9257		0.1100	0.3561	
**213**	16948	MH0108_GL0083509	COG0104	Adenylosuccinate synthase	Nucleotide transport and metabolism [F]	Purine metabolism	Faecalibacterium prausnitzii	Species	Firmicutes	Clostridia	Clostridiales	Ruminococcaceae	Faecalibacterium	Faecalibacterium prausnitzii	-0.1952	0.0798		0.1166	0.3228		-0.1854	0.0437	*
**214**	16977	MH0109_GL0036381	COG0149	Triosephosphate isomerase	Carbohydrate transport and metabolism [G]	Glycolysis / Gluconeogenesis	Firmicutes	Phylum	Firmicutes						0.2659	0.0141	*	-0.0588	0.6163		-0.0737	0.4282	
**215**	17010	MH0110_GL0025297	COG1080	Phosphoenolpyruvate-protein kinase (PTS system EI component in bacteria)	Carbohydrate transport and metabolism [G]	Pyruvate metabolism	Clostridiales	Order	Firmicutes	Clostridia	Clostridiales				-0.0804	0.6441		-0.1317	0.4680		-0.0034	0.9813	
**216**	17022	MH0110_GL0036341	COG1866	Phosphoenolpyruvate carboxykinase, ATP-dependent	Energy production and conversion [C]	Glycolysis / Gluconeogenesis	Faecalibacterium prausnitzii	Species	Firmicutes	Clostridia	Clostridiales	Ruminococcaceae	Faecalibacterium	Faecalibacterium prausnitzii	-0.1834	0.2892		0.3361	0.0589		-0.0822	0.5691	
**217**	17036	MH0110_GL0060617	COG3842	ABC-type Fe3+/spermidine/putrescine transport systems, ATPase components	Amino acid transport and metabolism [E]	ABC transporters	Clostridiales	Order	Firmicutes	Clostridia	Clostridiales				0.1510	0.1652		-0.0693	0.5458		0.0150	0.8698	
**218**	17080	MH0111_GL0044849	COG1653	ABC-type glycerol-3-phosphate transport system, periplasmic component	Carbohydrate transport and metabolism [G]	NAN	[Eubacterium] rectale	Species	Firmicutes	Clostridia	Clostridiales	Lachnospiraceae		[Eubacterium] rectale	-0.0046	0.9789		0.1451	0.4253		0.0446	0.7586	
**219**	17092	MH0111_GL0088449			NAN	NAN	Faecalibacterium prausnitzii	Species	Firmicutes	Clostridia	Clostridiales	Ruminococcaceae	Faecalibacterium	Faecalibacterium prausnitzii	0.1511	0.6250		0.0038	0.9906		0.3594	0.1566	
**220**	17212	MH0114_GL0000378	COG0255	Ribosomal protein L29	Translation, ribosomal structure and biogenesis [J]	Ribosome	Clostridiales	Order	Firmicutes	Clostridia	Clostridiales				0.0464	0.5261		-0.0124	0.8714		-0.0772	0.1997	
**221**	17248	MH0114_GL0065382	COG2848	Uncharacterized conserved protein, UPF0210 family	Cell cycle control, cell division, chromosome partitioning [D]	NAN	Clostridiales	Order	Firmicutes	Clostridia	Clostridiales				-0.0029	0.9796		-0.1117	0.3362		0.0171	0.8536	
**222**	17503	MH0119_GL0033639	COG1904	Glucuronate isomerase	Carbohydrate transport and metabolism [G]	Pentose and glucuronate interconversions	Faecalibacterium prausnitzii	Species	Firmicutes	Clostridia	Clostridiales	Ruminococcaceae	Faecalibacterium	Faecalibacterium prausnitzii	-0.1329	0.4806		-0.0245	0.9011		0.0803	0.6082	
**223**	17672	MH0131_GL0013416	COG1592	Rubrerythrin	Energy production and conversion [C]	NAN	Subdoligranulum variabile	Species	Firmicutes	Clostridia	Clostridiales	Ruminococcaceae	Subdoligranulum	Subdoligranulum variabile	-0.3511	0.1326		-0.0438	0.8595		-0.1201	0.5402	

**224**	17690	MH0122_GL0011708	COG0050	Translation elongation factor EF-Tu, a GTPase	Translation, ribosomal structure and biogenesis [J]	Plant-pathogen interaction	Subdoligranulum variabile	Species	Firmicutes	Clostridia	Clostridiales	Ruminococcaceae	Subdoligranulum	Subdoligranulum variabile	-0.5313	0.0169	*	0.0648	0.7879		0.0234	0.9027	
**225**	17710	MH0122_GL0027891	COG0098	Ribosomal protein S5	Translation, ribosomal structure and biogenesis [J]	Ribosome	Subdoligranulum variabile	Species	Firmicutes	Clostridia	Clostridiales	Ruminococcaceae	Subdoligranulum	Subdoligranulum variabile	0.0566	0.4125		0.1112	0.1192		-0.0777	0.1724	
**226**	17719	MH0122_GL0036643	COG1024	Enoyl-CoA hydratase/carnithine racemase	Lipid transport and metabolism [I]	NAN	Ruminococcaceae	Species	Firmicutes	Clostridia	Clostridiales	Ruminococcaceae	Faecalibacterium	Faecalibacterium prausnitzii	0.0018	0.9826		0.0327	0.7069		0.0799	0.2433	
**227**	17729	MH0122_GL0040083	COG0081	Ribosomal protein L1	Translation, ribosomal structure and biogenesis [J]	Ribosome	Bifidobacterium	Genus	Actinobacteria	Actinobacteria	Bifidobacteriales	Bifidobacteriaceae	Bifidobacterium		0.0627	0.6482		-0.0934	0.5143		-0.0332	0.7711	
**228**	17746	MH0122_GL0058467	COG1960	Acyl-CoA dehydrogenase related to the alkylation response protein AidB	Lipid transport and metabolism [I]	Fatty acid degradation	Ruminococcaceae	Species	Firmicutes	Clostridia	Clostridiales	Ruminococcaceae	Subdoligranulum	Subdoligranulum variabile	0.0024	0.9819		-0.1321	0.2214		0.1301	0.1274	
**229**	17749	MH0122_GL0061910	COG0255	Ribosomal protein L29	Translation, ribosomal structure and biogenesis [J]	Ribosome	Bifidobacterium	Genus	Actinobacteria	Actinobacteria	Bifidobacteriales	Bifidobacteriaceae	Bifidobacterium		0.1565	0.2952		-0.0572	0.7159		0.0842	0.4991	
**230**	17817	MH0122_GL0107606	COG1653	ABC-type glycerol-3-phosphate transport system, periplasmic component	Carbohydrate transport and metabolism [G]	ABC transporters	Bifidobacterium	Genus	Actinobacteria	Actinobacteria	Bifidobacteriales	Bifidobacteriaceae	Bifidobacterium		-0.1377	0.5627		0.0028	0.9910		0.1154	0.5590	
**231**	18139	MH0127_GL0025496	COG0126	3-phosphoglycerate kinase	Carbohydrate transport and metabolism [G]	Glycolysis / Gluconeogenesis	Faecalibacterium prausnitzii	Species	Firmicutes	Clostridia	Clostridiales	Ruminococcaceae	Faecalibacterium	Faecalibacterium prausnitzii	-0.2738	0.1134		0.2247	0.2164		-0.1222	0.4003	
**232**	18161	MH0127_GL0067595	COG3209	Uncharacterized conserved protein RhaS, contains 28 RHS repeats	General function prediction only [R]	NAN	Blautia obeum	Species	Firmicutes	Clostridia	Clostridiales	Lachnospiraceae	Blautia	Blautia obeum	0.2043	0.5822		0.1131	0.7708		0.3144	0.3052	
**233**	18350	MH0131_GL0003010	COG1070	Sugar (pentulose or hexulose) kinase	Carbohydrate transport and metabolism [G]	Pentose phosphate pathway	Clostridiales	Order	Firmicutes	Clostridia	Clostridiales				-0.1635	0.0868		0.0389	0.7013		-0.0256	0.7509	
**234**	18375	MH0131_GL0009720	COG0166	Glucose-6-phosphate isomerase	Carbohydrate transport and metabolism [G]	Glycolysis / Gluconeogenesis	Bifidobacterium	Genus	Actinobacteria	Actinobacteria	Bifidobacteriales	Bifidobacteriaceae	Bifidobacterium		-0.0467	0.6701		0.0307	0.7890		0.0001	0.9995	
**235**	18470	MH0131_GL0036835	COG0624	Acetylornithine deacetylase/Succinyl-diaminopimelate desuccinylase or related deacylase	Amino acid transport and metabolism [E]	Lysine biosynthesis	Collinsella	Genus	Actinobacteria	Coriobacteriia	Coriobacteriales	Coriobacteriaceae	Collinsella		-0.2066	0.1319		0.1195	0.4088		-0.0772	0.5030	
**236**	18487	MH0131_GL0040021	COG0166	Glucose-6-phosphate isomerase	Carbohydrate transport and metabolism [G]	Glycolysis / Gluconeogenesis	Clostridiales	Order	Firmicutes	Clostridia	Clostridiales				-0.0659	0.5947		0.0442	0.7327		-0.1234	0.2266	
**237**	18490	MH0131_GL0040069	COG0544	FKBP-type peptidyl-prolyl cis-trans isomerase (trigger factor)	Posttranslational modification, protein turnover, chaperones [O]	NAN	Collinsella aerofaciens	Species	Actinobacteria	Coriobacteriia	Coriobacteriales	Coriobacteriaceae	Collinsella	Collinsella aerofaciens	-0.1321	0.1347		-0.0627	0.5013		-0.0376	0.6120	
**238**	18517	MH0131_GL0045133	COG2352	Phosphoenolpyruvate carboxylase	Energy production and conversion [C]	Pyruvate metabolism	Bifidobacterium	Genus	Actinobacteria	Actinobacteria	Bifidobacteriales	Bifidobacteriaceae	Bifidobacterium		-0.0434	0.7810		-0.0447	0.7841		0.0132	0.9192	
**239**	18527	MH0131_GL0047971	COG0469	Pyruvate kinase	Carbohydrate transport and metabolism [G]	Glycolysis / Gluconeogenesis	Collinsella aerofaciens	Species	Actinobacteria	Coriobacteriia	Coriobacteriales	Coriobacteriaceae	Collinsella	Collinsella aerofaciens	-0.2472	0.0814		-0.0904	0.5487		-0.0541	0.6517	
**240**	18545	MH0131_GL0053906	COG0081	Ribosomal protein L1	Translation, ribosomal structure and biogenesis [J]	Ribosome	Bifidobacterium adolescentis	Species	Actinobacteria	Actinobacteria	Bifidobacteriales	Bifidobacteriaceae	Bifidobacterium	Bifidobacterium adolescentis	0.0258	0.8494		-0.0581	0.6822		-0.0254	0.8222	
**241**	18548	MH0131_GL0055410	COG0747	ABC-type transport system, periplasmic component	Amino acid transport and metabolism [E]	ABC transporters	Collinsella aerofaciens	Species	Actinobacteria	Coriobacteriia	Coriobacteriales	Coriobacteriaceae	Collinsella	Collinsella aerofaciens	-0.2086	0.1620		0.1210	0.4415		-0.1858	0.1328	
**242**	18549	MH0131_GL0055413	COG4608	ABC-type oligopeptide transport system, ATPase component	Amino acid transport and metabolism [E]	ABC transporters		Genus	Actinobacteria	Coriobacteriia	Coriobacteriales	Coriobacteriaceae	Collinsella		-0.2001	0.1392		-0.0524	0.7144		-0.1356	0.2295	
**243**	18559	MH0131_GL0059552	COG0057	Glyceraldehyde-3-phosphate dehydrogenase/erythrose-4-phosphate dehydrogenase	Carbohydrate transport and metabolism [G]	Glycolysis / Gluconeogenesis	Clostridiales	Order	Firmicutes	Clostridia	Clostridiales				-0.1351	0.3792		0.0646	0.6885		-0.2108	0.0940	
**244**	18560	NLF015_GL0061486	COG0126	3-phosphoglycerate kinase	Carbohydrate transport and metabolism [G]	NAN	Clostridiales	Order	Firmicutes	Clostridia	Clostridiales				0.0897	0.3694		-0.1272	0.2212		0.0025	0.9758	
**245**	18561	MH0131_GL0059554	COG0149	Triosephosphate isomerase	Carbohydrate transport and metabolism [G]	Glycolysis / Gluconeogenesis	Clostridiales	Order	Firmicutes	Clostridia	Clostridiales				0.0222	0.8429		-0.0130	0.9112		-0.0804	0.3849	
**246**	18580	MH0131_GL0070829	COG1185	Polyribonucleotide nucleotidyltransferase (polynucleotide phosphorylase)	Translation, ribosomal structure and biogenesis [J]	Purine metabolism	Bifidobacterium	Genus	Actinobacteria	Actinobacteria	Bifidobacteriales	Bifidobacteriaceae	Bifidobacterium		-0.1486	0.3083		-0.1993	0.1890		0.0030	0.9804	
**247**	18636	MH0131_GL0088691	COG1080	Phosphoenolpyruvate-protein kinase (PTS system EI component in bacteria)	Carbohydrate transport and metabolism [G]	Pyruvate metabolism	Coprococcus	Genus	Firmicutes	Clostridia	Clostridiales	Lachnospiraceae	Coprococcus		0.0232	0.8610		0.0311	0.8220		0.2153	0.0441	*

**248**	18645	MH0131_GL0090479	COG1454	Alcohol dehydrogenase, class IV	Energy production and conversion [C]	Glycolysis / Gluconeogenesis	Collinsella aerofaciens	Species	Actinobacteria	Coriobacteriia	Coriobacteriales	Coriobacteriaceae	Collinsella	Collinsella aerofaciens	-0.1705	0.2509		-0.0615	0.6941		-0.1166	0.3453	
**249**	18682	MH0131_GL0099453	COG1454	Alcohol dehydrogenase, class IV	Energy production and conversion [C]	Glycolysis / Gluconeogenesis	Collinsella aerofaciens	Species	Actinobacteria	Coriobacteriia	Coriobacteriales	Coriobacteriaceae	Collinsella	Collinsella aerofaciens	-0.1792	0.2235		-0.1285	0.4056		-0.1793	0.1404	
**250**	18705	MH0131_GL0105124	COG2235	Arginine deiminase	Amino acid transport and metabolism [E]	Arginine and proline metabolism	Collinsella	Genus	Actinobacteria	Coriobacteriia	Coriobacteriales	Coriobacteriaceae	Collinsella		-0.2091	0.1256		0.1442	0.3159		-0.1068	0.3506	
**251**	18774	MH0307_GL0076667	COG1544	Ribosome-associated translation inhibitor RaiA	Translation, ribosomal structure and biogenesis [J]	NAN	Collinsella	Genus	Actinobacteria	Coriobacteriia	Coriobacteriales	Coriobacteriaceae	Collinsella		-0.0559	0.5776		-0.1459	0.1596		-0.0293	0.7250	
**252**	18781	MH0131_GL0123487	COG0091	Ribosomal protein L22	Translation, ribosomal structure and biogenesis [J]	Ribosome	Collinsella	Genus	Actinobacteria	Coriobacteriia	Coriobacteriales	Coriobacteriaceae	Collinsella		-0.1195	0.2425		-0.1506	0.1568		0.0043	0.9601	
**253**	18818	MH0131_GL0136999	COG4822	Cobalamin biosynthesis protein CbiK, Co2+ chelatase	Coenzyme transport and metabolism [H]	Porphyrin and chlorophyll metabolism	Clostridiales	Order	Firmicutes	Clostridia	Clostridiales				-0.0854	0.5611		0.0260	0.8659		-0.2159	0.0714	
**254**	18821	MH0131_GL0138470	COG1882	Pyruvate-formate lyase	Energy production and conversion [C]	Pyruvate metabolism	Fusicatenibacter saccharivorans	Species	Firmicutes	Clostridia	Clostridiales	Lachnospiraceae	Fusicatenibacter	Fusicatenibacter saccharivorans	0.0036	0.9742		-0.1424	0.2202		0.1791	0.0488	*
**255**	18826	MH0131_GL0139126	COG0264	Translation elongation factor EF-Ts	Translation, ribosomal structure and biogenesis [J]	NAN	Collinsella	Genus	Actinobacteria	Coriobacteriia	Coriobacteriales	Coriobacteriaceae	Collinsella		-0.1657	0.2321		-0.1022	0.4829		-0.0842	0.4670	
**256**	18868	MH0131_GL0154213	COG0544	FKBP-type peptidyl-prolyl cis-trans isomerase (trigger factor)	Posttranslational modification, protein turnover, chaperones [O]	NAN	Bifidobacterium	Genus	Actinobacteria	Actinobacteria	Bifidobacteriales	Bifidobacteriaceae	Bifidobacterium		0.1061	0.5385		-0.1017	0.5728		0.0089	0.9504	
**257**	18927	MH0131_GL0169225	COG0094	Ribosomal protein L5	Translation, ribosomal structure and biogenesis [J]	Ribosome	Collinsella	Genus	Actinobacteria	Coriobacteriia	Coriobacteriales	Coriobacteriaceae	Collinsella		-0.1020	0.2237		-0.0107	0.9037		-0.0639	0.3604	
**258**	18955	MH0131_GL0174780	COG0334	Glutamate dehydrogenase/leucine dehydrogenase	Amino acid transport and metabolism [E]	Alanine, aspartate and glutamate metabolism	Bifidobacterium adolescentis	Species	Actinobacteria	Actinobacteria	Bifidobacteriales	Bifidobacteriaceae	Bifidobacterium	Bifidobacterium adolescentis	-0.0309	0.8022		-0.0362	0.7792		0.1412	0.1631	
**259**	18959	MH0131_GL0175176	COG1882	Pyruvate-formate lyase	Energy production and conversion [C]	Pyruvate metabolism	Collinsella	Genus	Actinobacteria	Coriobacteriia	Coriobacteriales	Coriobacteriaceae	Collinsella		-0.1898	0.1336		-0.0219	0.8700		-0.1354	0.1992	
**260**	19189	MH0136_GL0083951	COG1904	Glucuronate isomerase	Carbohydrate transport and metabolism [G]	Pentose and glucuronate interconversions	Faecalibacterium prausnitzii	Species	Firmicutes	Clostridia	Clostridiales	Ruminococcaceae	Faecalibacterium	Faecalibacterium prausnitzii	-0.1924	0.1349		-0.1113	0.4125		0.1840	0.0835	
**261**	19307	MH0139_GL0041455	COG0166	Glucose-6-phosphate isomerase	Carbohydrate transport and metabolism [G]	Glycolysis / Gluconeogenesis	Clostridiales	Order	Firmicutes	Clostridia	Clostridiales				-0.0332	0.8423		0.0865	0.6193		-0.0604	0.6626	
**262**	19410	MH0140_GL0123359	COG0205	6-phosphofructokinase	Carbohydrate transport and metabolism [G]	Glycolysis / Gluconeogenesis	Ruminococcus bromii	Species	Firmicutes	Clostridia	Clostridiales	Ruminococcaceae	Ruminococcus	Ruminococcus bromii	-0.0792	0.3847		0.1295	0.1704		-0.1175	0.1162	
**263**	19720	MH0145_GL0031362			NAN	Galactose metabolism	Ruminococcus bromii	Species	Firmicutes	Clostridia	Clostridiales	Ruminococcaceae	Ruminococcus	Ruminococcus bromii	-0.1801	0.1649		0.1402	0.3032		-0.1496	0.1646	
**264**	19738	MH0145_GL0085935	COG5263	Glucan-binding domain (YG repeat)	Carbohydrate transport and metabolism [G]	NAN	Clostridiales	Order	Firmicutes	Clostridia	Clostridiales				-0.1142	0.1765		-0.0023	0.9796		-0.0367	0.6048	
**265**	19764	MH0145_GL0159811	COG1109	Phosphomannomutase	Carbohydrate transport and metabolism [G]	Glycolysis / Gluconeogenesis	Clostridiales	Order	Firmicutes	Clostridia	Clostridiales				-0.0299	0.7089		-0.0024	0.9768		0.0623	0.3463	
**266**	19869	MH0147_GL0083186	COG4166	ABC-type oligopeptide transport system, periplasmic component	Amino acid transport and metabolism [E]	ABC transporters	Fusicatenibacter saccharivorans	Species	Firmicutes	Clostridia	Clostridiales	Lachnospiraceae	Fusicatenibacter	Fusicatenibacter saccharivorans	-0.0004	0.9977		-0.0069	0.9585		0.0145	0.8907	
**267**	20073	MH0149_GL0094229	COG1080	Phosphoenolpyruvate-protein kinase (PTS system EI component in bacteria)	Carbohydrate transport and metabolism [G]	Pyruvate metabolism	Faecalibacterium prausnitzii	Species	Firmicutes	Clostridia	Clostridiales	Ruminococcaceae	Faecalibacterium	Faecalibacterium prausnitzii	-0.2595	0.3308		-0.0993	0.7229		-0.0812	0.7152	
**268**	20414	MH0442_GL0264877	COG1592	Rubrerythrin	Energy production and conversion [C]	Oxidative phosphorylation	Clostridiales	Order	Firmicutes	Clostridia	Clostridiales				0.0325	0.7164		0.0449	0.6306		-0.0967	0.1879	
**269**	20460	MH0157_GL0012420	COG0191	Fructose/tagatose bisphosphate aldolase	Carbohydrate transport and metabolism [G]	Glycolysis / Gluconeogenesis	Lachnospiraceae	Family	Firmicutes	Clostridia	Clostridiales	Lachnospiraceae			-0.2360	0.1225		0.2064	0.1981		0.0785	0.5414	
**270**	20476	MH0157_GL0087463	COG0745	DNA-binding response regulator, OmpR family, contains REC and winged-helix (wHTH) domain	Signal transduction mechanisms [T]	NAN	Prevotella copri	Species	Bacteroidetes	Bacteroidia	Bacteroidales	Prevotellaceae	Prevotella	Prevotella copri	0.0389	0.8486		-0.3315	0.1137		0.0726	0.6675	
**271**	20577	MH0161_GL0001231	COG3842	ABC-type Fe3+/spermidine/putrescine transport systems, ATPase components	Amino acid transport and metabolism [E]	ABC transporters	Bifidobacterium	Genus	Actinobacteria	Actinobacteria	Bifidobacteriales	Bifidobacteriaceae	Bifidobacterium		-0.0257	0.8277		-0.0809	0.5104		0.1187	0.2211	
**272**	20644	MH0161_GL0061147	COG0115	Branched-chain amino acid aminotransferase/4-amino-4-deoxychorismate lyase	Coenzyme transport and metabolism [H]	Valine, leucine and isoleucine degradation	Bifidobacterium adolescentis	Species	Actinobacteria	Actinobacteria	Bifidobacteriales	Bifidobacteriaceae	Bifidobacterium	Bifidobacterium adolescentis	-0.0696	0.6289		-0.0726	0.6294		-0.0572	0.6323	

**273**	20650	MH0161_GL0069344	COG1653	ABC-type glycerol-3-phosphate transport system, periplasmic component	Carbohydrate transport and metabolism [G]	ABC transporters	Collinsella	Genus	Actinobacteria	Coriobacteriia	Coriobacteriales	Coriobacteriaceae	Collinsella		-0.1887	0.2738		0.0435	0.8106		-0.0976	0.4972	
**274**	20665	MH0161_GL0081156	COG0149	Triosephosphate isomerase	Carbohydrate transport and metabolism [G]	Glycolysis / Gluconeogenesis	Coprococcus comes	Species	Firmicutes	Clostridia	Clostridiales	Lachnospiraceae	Coprococcus	Coprococcus comes	-0.1279	0.1382		0.0749	0.4105		0.0046	0.9496	
**275**	20672	MH0161_GL0083626	COG0480	Translation elongation factor EF-G, a GTPase	Translation, ribosomal structure and biogenesis [J]	NAN	Bifidobacterium adolescentis	Species	Actinobacteria	Actinobacteria	Bifidobacteriales	Bifidobacteriaceae	Bifidobacterium	Bifidobacterium adolescentis	-0.2513	0.1587		-0.1899	0.3109		-0.0343	0.8192	
**276**	20712	MH0161_GL0110049	COG2407	L-fucose isomerase or related protein	Carbohydrate transport and metabolism [G]	Fructose and mannose metabolism	Blautia obeum	Species	Firmicutes	Clostridia	Clostridiales	Lachnospiraceae	Blautia	Blautia obeum	-0.0561	0.5643		-0.1126	0.2649		-0.0648	0.4215	
**277**	20757	MH0161_GL0142425	COG0148	Enolase	Carbohydrate transport and metabolism [G]	Glycolysis / Gluconeogenesis	Bifidobacterium adolescentis	Species	Actinobacteria	Actinobacteria	Bifidobacteriales	Bifidobacteriaceae	Bifidobacterium	Bifidobacterium adolescentis	-0.0237	0.8969		-0.0093	0.9610		0.1125	0.4566	
**278**	20764	MH0161_GL0152524	COG1362	Aspartyl aminopeptidase	Amino acid transport and metabolism [E]	NAN	Faecalibacterium prausnitzii	Species	Firmicutes	Clostridia	Clostridiales	Ruminococcaceae	Faecalibacterium	Faecalibacterium prausnitzii	-0.2481	0.1551		-0.0744	0.6869		0.2302	0.1108	
**279**	20773	MH0161_GL0161160	COG0747	ABC-type transport system, periplasmic component	Amino acid transport and metabolism [E]	NAN	Polymorphum gilvum	Species	Proteobacteria	Alphaproteobacteria			Polymorphum	Polymorphum gilvum	-0.0498	0.5625		-0.0485	0.5898		-0.0672	0.3454	
**280**	20810	MH0162_GL0053827	COG1102	Cytidylate kinase	Nucleotide transport and metabolism [F]	NAN	Subdoligranulum variabile	Species	Firmicutes	Clostridia	Clostridiales	Ruminococcaceae	Subdoligranulum	Subdoligranulum variabile	0.0825	0.2810		-0.0168	0.8346		0.0831	0.1891	
**281**	20867	MH0163_GL0059562	COG0246	Mannitol-1-phosphate/altronate dehydrogenases	Carbohydrate transport and metabolism [G]	Pentose and glucuronate interconversions	Subdoligranulum variabile	Species	Firmicutes	Clostridia	Clostridiales	Ruminococcaceae	Subdoligranulum	Subdoligranulum variabile	0.1723	0.0736		-0.0580	0.5715		0.0345	0.6724	
**282**	21083	MH0170_GL0014792	COG0235	Ribulose-5-phosphate 4-epimerase/Fuculose-1-phosphate aldolase	Carbohydrate transport and metabolism [G]	Pentose and glucuronate interconversions	Clostridiales	Order	Firmicutes	Clostridia	Clostridiales				0.0756	0.4857		0.0152	0.8938		0.0738	0.4113	
**283**	21098	MH0170_GL0056715	COG1145	Ferredoxin	Energy production and conversion [C]	Glycolysis / Gluconeogenesis	Fusicatenibacter saccharivorans	Species	Firmicutes	Clostridia	Clostridiales	Lachnospiraceae	Fusicatenibacter	Fusicatenibacter saccharivorans	-0.0553	0.5894		-0.0471	0.6601		-0.1075	0.2023	
**284**	21298	MH0177_GL0076497	COG0183	Acetyl-CoA acetyltransferase	Lipid transport and metabolism [I]	Fatty acid degradation	Faecalibacterium prausnitzii	Species	Firmicutes	Clostridia	Clostridiales	Ruminococcaceae	Faecalibacterium	Faecalibacterium prausnitzii	-0.1418	0.4134		0.1797	0.3199		-0.0696	0.6296	
**285**	21491	MH0182_GL0009170	COG0050	Translation elongation factor EF-Tu, a GTPase	Translation, ribosomal structure and biogenesis [J]	Plant-pathogen interaction	Clostridiales	Order	Firmicutes	Clostridia	Clostridiales				0.0505	0.6940		-0.0905	0.4993		-0.1281	0.2259	
**286**	21665	MH0184_GL0118471	COG1653	ABC-type glycerol-3-phosphate transport system, periplasmic component	Carbohydrate transport and metabolism [G]	ABC transporters	Subdoligranulum variabile	Species	Firmicutes	Clostridia	Clostridiales	Ruminococcaceae	Subdoligranulum	Subdoligranulum variabile	0.0314	0.8654		-0.0499	0.7963		0.0293	0.8489	
**287**	21666	MH0184_GL0127793	COG0092	Ribosomal protein S3	Translation, ribosomal structure and biogenesis [J]	Ribosome	Ruminococcus bromii	Species	Firmicutes	Clostridia	Clostridiales	Ruminococcaceae	Ruminococcus	Ruminococcus bromii	-0.0267	0.8131		0.0345	0.7695		-0.1770	0.0530	
**288**	21833	MH0188_GL0007212	COG0039	Malate/lactate dehydrogenase	Energy production and conversion [C]	Glycolysis / Gluconeogenesis	Bifidobacterium	Genus	Actinobacteria	Actinobacteria	Bifidobacteriales	Bifidobacteriaceae	Bifidobacterium		0.1018	0.5619		-0.0264	0.8858		0.0321	0.8259	
**289**	21865	MH0188_GL0018903	COG0473	Isocitrate/isopropylmalate dehydrogenase	Amino acid transport and metabolism [E]	Citrate cycle (TCA cycle)	Bifidobacterium	Genus	Actinobacteria	Actinobacteria	Bifidobacteriales	Bifidobacteriaceae	Bifidobacterium		0.0069	0.9362		0.0016	0.9862		-0.0051	0.9432	
**290**	21893	MH0188_GL0025027	COG1653	ABC-type glycerol-3-phosphate transport system, periplasmic component	Carbohydrate transport and metabolism [G]	ABC transporters	Bifidobacterium	Genus	Actinobacteria	Actinobacteria	Bifidobacteriales	Bifidobacteriaceae	Bifidobacterium		0.1668	0.1795		-0.1141	0.3823		0.1450	0.1592	
**291**	21896	MH0188_GL0025245	COG0021	Transketolase	Carbohydrate transport and metabolism [G]	Pentose phosphate pathway	Bifidobacterium	Genus	Actinobacteria	Actinobacteria	Bifidobacteriales	Bifidobacteriaceae	Bifidobacterium		-0.1907	0.2066		0.0960	0.5460		-0.0326	0.7969	
**292**	21902	MH0188_GL0028315	COG1653	ABC-type glycerol-3-phosphate transport system, periplasmic component	Carbohydrate transport and metabolism [G]	ABC transporters	Clostridiales	Order	Firmicutes	Clostridia	Clostridiales				-0.0596	0.6272		-0.2308	0.0661		-0.0244	0.8108	
**293**	21905	MH0188_GL0029398	COG0528	Uridylate kinase	Nucleotide transport and metabolism [F]	Pyrimidine metabolism	Bifidobacterium	Genus	Actinobacteria	Actinobacteria	Bifidobacteriales	Bifidobacteriaceae	Bifidobacterium		-0.0816	0.4400		-0.0866	0.4325		0.0819	0.3493	
**294**	21906	MH0188_GL0029399	COG0264	Translation elongation factor EF-Ts	Translation, ribosomal structure and biogenesis [J]	NAN	Bifidobacterium	Genus	Actinobacteria	Actinobacteria	Bifidobacteriales	Bifidobacteriaceae	Bifidobacterium		0.0396	0.7771		-0.0449	0.7587		0.0508	0.6615	
**295**	21946	MH0188_GL0043950	COG1087	UDP-glucose 4-epimerase	Cell wall/membrane/envelope biogenesis [M]	Galactose metabolism	Bacteria	Superkingdom							-0.0658	0.6779		-0.0548	0.7405		0.0019	0.9884	
**296**	21975	MH0188_GL0054468	COG0172	Seryl-tRNA synthetase	Translation, ribosomal structure and biogenesis [J]	Aminoacyl-tRNA biosynthesis	Bifidobacterium	Genus	Actinobacteria	Actinobacteria	Bifidobacteriales	Bifidobacteriaceae	Bifidobacterium		-0.1122	0.3887		0.0276	0.8398		0.0334	0.7583	

**297**	21984	MH0188_GL0055295	COG1653	ABC-type glycerol-3-phosphate transport system, periplasmic component	Carbohydrate transport and metabolism [G]	ABC transporters	Bifidobacterium	Genus	Actinobacteria	Actinobacteria	Bifidobacteriales	Bifidobacteriaceae	Bifidobacterium		0.0227	0.9145		0.0294	0.8939		-0.0878	0.6158	
**298**	22012	MH0188_GL0064273	COG1879	ABC-type sugar transport system, periplasmic component, contains N-terminal xre family HTH domain	Carbohydrate transport and metabolism [G]	ABC transporters	Blautia obeum	Species	Firmicutes	Clostridia	Clostridiales	Lachnospiraceae	Blautia	Blautia obeum	0.0310	0.7349		0.0619	0.5173		-0.0893	0.2365	
**299**	22018	MH0188_GL0066755	COG0103	Ribosomal protein S9	Translation, ribosomal structure and biogenesis [J]	Ribosome	Bifidobacterium	Genus	Actinobacteria	Actinobacteria	Bifidobacteriales	Bifidobacteriaceae	Bifidobacterium		0.0103	0.9188		-0.1950	0.0574		0.0067	0.9364	
**300**	22024	MH0188_GL0067094	COG0086	DNA-directed RNA polymerase, beta' subunit/160 kD subunit	Transcription [K]	Purine metabolism	Bifidobacterium	Genus	Actinobacteria	Actinobacteria	Bifidobacteriales	Bifidobacteriaceae	Bifidobacterium		-0.0520	0.6746		-0.0477	0.7125		-0.0368	0.7202	
**301**	22027	MH0188_GL0067976	COG1129	ABC-type sugar transport system, ATPase component	Carbohydrate transport and metabolism [G]	ABC transporters	Bifidobacterium	Genus	Actinobacteria	Actinobacteria	Bifidobacteriales	Bifidobacteriaceae	Bifidobacterium		0.1457	0.3114		-0.2782	0.0600		0.1080	0.3667	
**302**	22030	MH0188_GL0069412	COG0282	Acetate kinase	Energy production and conversion [C]	Taurine and hypotaurine metabolism	Bifidobacterium	Genus	Actinobacteria	Actinobacteria	Bifidobacteriales	Bifidobacteriaceae	Bifidobacterium		0.1150	0.4808		-0.0298	0.8617		0.0440	0.7460	
**303**	22039	MH0188_GL0071819	COG0747	ABC-type transport system, periplasmic component	Amino acid transport and metabolism [E]	ABC transporters	Bifidobacterium longum	Species	Actinobacteria	Actinobacteria	Bifidobacteriales	Bifidobacteriaceae	Bifidobacterium	Bifidobacterium longum	0.0565	0.7079		-0.1486	0.3436		0.0588	0.6388	
**304**	22064	MH0188_GL0082698	COG1544	Ribosome-associated translation inhibitor RaiA	Translation, ribosomal structure and biogenesis [J]	NAN	Bifidobacterium	Genus	Actinobacteria	Actinobacteria	Bifidobacteriales	Bifidobacteriaceae	Bifidobacterium		0.1227	0.3733		-0.0404	0.7799		0.0444	0.6991	
**305**	22074	NLF007_GL0024227	COG0426	Flavorubredoxin	Energy production and conversion [C]	NAN	Clostridiales	Order	Firmicutes	Clostridia	Clostridiales				-0.0286	0.8086		-0.0224	0.8556		0.0047	0.9616	
**306**	22087	MH0188_GL0093557	COG0359	Ribosomal protein L9	Translation, ribosomal structure and biogenesis [J]	Ribosome	Bifidobacterium	Genus	Actinobacteria	Actinobacteria	Bifidobacteriales	Bifidobacteriaceae	Bifidobacterium		0.0704	0.6182		-0.0216	0.8837		0.0116	0.9211	
**307**	22090	MH0188_GL0095502	COG0174	Glutamine synthetase	Amino acid transport and metabolism [E]	Alanine, aspartate and glutamate metabolism	Bifidobacterium	Genus	Actinobacteria	Actinobacteria	Bifidobacteriales	Bifidobacteriaceae	Bifidobacterium		-0.0979	0.6411		-0.2836	0.1918		0.2336	0.1758	
**308**	22169	MH0188_GL0115288	COG0235	Ribulose-5-phosphate 4-epimerase/Fuculose-1-phosphate aldolase	Carbohydrate transport and metabolism [G]	Pentose and glucuronate interconversions	Bifidobacterium	Genus	Actinobacteria	Actinobacteria	Bifidobacteriales	Bifidobacteriaceae	Bifidobacterium		0.0525	0.5975		-0.0473	0.6489		-0.1038	0.2047	
**309**	22205	MH0188_GL0126744	COG0459	Chaperonin GroEL (HSP60 family)	Posttranslational modification, protein turnover, chaperones [O]	RNA degradation	Bifidobacterium	Genus	Actinobacteria	Actinobacteria	Bifidobacteriales	Bifidobacteriaceae	Bifidobacterium		0.0279	0.8858		-0.2632	0.1896		0.1609	0.3152	
**310**	22221	MH0188_GL0133256	COG1087	UDP-glucose 4-epimerase	Cell wall/membrane/envelope biogenesis [M]	Galactose metabolism	Bifidobacterium longum	Species	Actinobacteria	Actinobacteria	Bifidobacteriales	Bifidobacteriaceae	Bifidobacterium	Bifidobacterium longum	0.1067	0.5194		0.0190	0.9129		0.0453	0.7421	
**311**	22227	MH0188_GL0137837	COG0198	Ribosomal protein L24	Translation, ribosomal structure and biogenesis [J]	Ribosome	Bifidobacterium	Genus	Actinobacteria	Actinobacteria	Bifidobacteriales	Bifidobacteriaceae	Bifidobacterium		-0.0070	0.9407		-0.0614	0.5313		0.0565	0.4682	
**312**	22236	MH0188_GL0141556	COG0050	Translation elongation factor EF-Tu, a GTPase	Translation, ribosomal structure and biogenesis [J]	Plant-pathogen interaction	Collinsella	Genus	Actinobacteria	Coriobacteriia	Coriobacteriales	Coriobacteriaceae	Collinsella		-0.6933	0.0013	**	0.3018	0.2035		-0.2259	0.2317	
**313**	22249	MH0188_GL0143190	COG0176	Transaldolase	Carbohydrate transport and metabolism [G]	Pentose phosphate pathway	Bifidobacterium	Genus	Actinobacteria	Actinobacteria	Bifidobacteriales	Bifidobacteriaceae	Bifidobacterium		0.0586	0.6951		0.0473	0.7621		-0.0143	0.9086	
**314**	22254	MH0188_GL0145450	COG1882	Pyruvate-formate lyase	Energy production and conversion [C]	Pyruvate metabolism	Bifidobacterium adolescentis	Species	Actinobacteria	Actinobacteria	Bifidobacteriales	Bifidobacteriaceae	Bifidobacterium	Bifidobacterium adolescentis	-0.1268	0.5390		0.0538	0.8034		-0.0129	0.9403	
**315**	22257	MH0188_GL0146214	COG0459	Chaperonin GroEL (HSP60 family)	Posttranslational modification, protein turnover, chaperones [O]	RNA degradation	Collinsella	Genus	Actinobacteria	Coriobacteriia	Coriobacteriales	Coriobacteriaceae	Collinsella		-0.1249	0.5171		0.0392	0.8462		-0.1130	0.4799	
**316**	22288	MH0188_GL0156393	COG0104	Adenylosuccinate synthase	Nucleotide transport and metabolism [F]	Purine metabolism	Clostridiales	Order	Firmicutes	Clostridia	Clostridiales				0.1153	0.1282		0.0278	0.7293		0.0583	0.3585	
**317**	22302	MH0188_GL0160346	COG0058	Glucan phosphorylase	Carbohydrate transport and metabolism [G]	Starch and sucrose metabolism	Bifidobacterium adolescentis	Species	Actinobacteria	Actinobacteria	Bifidobacteriales	Bifidobacteriaceae	Bifidobacterium	Bifidobacterium adolescentis	-0.2718	0.1836		-0.0939	0.6633		0.1157	0.4989	
**318**	22338	MH0188_GL0176742	COG0202	DNA-directed RNA polymerase, alpha subunit/40 kD subunit	Transcription [K]	Purine metabolism	Collinsella	Genus	Actinobacteria	Coriobacteriia	Coriobacteriales	Coriobacteriaceae	Collinsella		-0.0904	0.5279		-0.1022	0.4941		-0.0625	0.5994	
**319**	22376	MH0188_GL0186972	COG0480	Translation elongation factor EF-G, a GTPase	Translation, ribosomal structure and biogenesis [J]	NAN	Clostridiales	Order	Firmicutes	Clostridia	Clostridiales				-0.1269	0.1108		0.0978	0.2429		-0.0571	0.3927	
**320**	22394	MH0188_GL0191505	COG1454	Alcohol dehydrogenase, class IV	Energy production and conversion [C]	Glycolysis / Gluconeogenesis	Bifidobacterium	Genus	Actinobacteria	Actinobacteria	Bifidobacteriales	Bifidobacteriaceae	Bifidobacterium		-0.1918	0.2431		0.0395	0.8194		0.0373	0.7863	

**321**	22395	MH0188_GL0191508	COG0088	Ribosomal protein L4	Translation, ribosomal structure and biogenesis [J]	Ribosome	Bacteria	Superkingdom							-0.1418	0.2763		-0.0388	0.7773		-0.0562	0.6055	
**322**	22489	MH0189_GL0094693	COG4690	Dipeptidase	Amino acid transport and metabolism [E]	NAN	Bifidobacterium adolescentis	Species	Actinobacteria	Actinobacteria	Bifidobacteriales	Bifidobacteriaceae	Bifidobacterium	Bifidobacterium adolescentis	-0.1020	0.4104		0.1489	0.2478		0.0616	0.5498	
**323**	22497	MH0189_GL0100565	COG0050	Translation elongation factor EF-Tu, a GTPase	Translation, ribosomal structure and biogenesis [J]	Plant-pathogen interaction	Bifidobacterium	Genus	Actinobacteria	Actinobacteria	Bifidobacteriales	Bifidobacteriaceae	Bifidobacterium		-0.1506	0.4030		-0.1153	0.5409		-0.1634	0.2730	
**324**	22707	MH0192_GL0016996	COG1129	ABC-type sugar transport system, ATPase component	Carbohydrate transport and metabolism [G]	ABC transporters	Clostridiales	Order	Firmicutes	Clostridia	Clostridiales				0.0358	0.7700		-0.1150	0.3659		-0.1088	0.2808	
**325**	22837	MH0193_GL0010680	COG0480	Translation elongation factor EF-G, a GTPase	Translation, ribosomal structure and biogenesis [J]	NAN	Bifidobacterium	Genus	Actinobacteria	Actinobacteria	Bifidobacteriales	Bifidobacteriaceae	Bifidobacterium		0.1694	0.5313		0.1724	0.5420		0.0908	0.6863	
**326**	22838	MH0193_GL0010681	COG0050	Translation elongation factor EF-Tu, a GTPase	Translation, ribosomal structure and biogenesis [J]	Plant-pathogen interaction	Bifidobacterium longum	Species	Actinobacteria	Actinobacteria	Bifidobacteriales	Bifidobacteriaceae	Bifidobacterium	Bifidobacterium longum	0.1192	0.4079		-0.0975	0.5177		0.0442	0.7126	
**327**	22866	MH0193_GL0048220	COG0282	Acetate kinase(	Energy production and conversion [C]	Taurine and hypotaurine metabolism	Ruminococcus bromii	Species	Firmicutes	Clostridia	Clostridiales	Ruminococcaceae	Ruminococcus	Ruminococcus bromii	-0.0807	0.4498		0.1308	0.2386		-0.1578	0.0700	
**328**	22871	MH0193_GL0052517	COG1879	ABC-type sugar transport system, periplasmic component, contains N-terminal xre family HTH domain	Carbohydrate transport and metabolism [G]	ABC transporters	Bifidobacterium	Genus	Actinobacteria	Actinobacteria	Bifidobacteriales	Bifidobacteriaceae	Bifidobacterium		0.2214	0.1876		0.0318	0.8578		0.0420	0.7659	
**329**	22874	MH0193_GL0053065			NAN	NAN	Clostridiales	Order	Firmicutes	Clostridia	Clostridiales				-0.0065	0.9498		0.1121	0.2984		-0.0158	0.8546	
**330**	22987	MH0193_GL0165661	COG0334	Glutamate dehydrogenase/leucine dehydrogenase	Amino acid transport and metabolism [E]	Alanine, aspartate and glutamate metabolism	Bifidobacterium	Genus	Actinobacteria	Actinobacteria	Bifidobacteriales	Bifidobacteriaceae	Bifidobacterium		0.0105	0.9510		0.0062	0.9724		0.0623	0.6611	
**331**	23014	MH0193_GL0186495	COG1454	Alcohol dehydrogenase, class IV	Energy production and conversion [C]	Glycolysis / Gluconeogenesis	Bifidobacterium	Genus	Actinobacteria	Actinobacteria	Bifidobacteriales	Bifidobacteriaceae	Bifidobacterium		0.1580	0.4961		-0.0475	0.8449		0.2335	0.2224	
**332**	23023	MH0193_GL0193391	COG1653	ABC-type glycerol-3-phosphate transport system, periplasmic component	Carbohydrate transport and metabolism [G]	ABC transporters	Clostridiales	Order	Firmicutes	Clostridia	Clostridiales				0.0427	0.7211		0.0520	0.6772		0.0596	0.5478	
**333**	23172	MH0196_GL0219356	COG4213	ABC-type xylose transport system, periplasmic component	Carbohydrate transport and metabolism [G]	ABC transporters	Clostridiales	Order	Firmicutes	Clostridia	Clostridiales				0.0774	0.3824		0.0414	0.6561		-0.0166	0.8223	
**334**	23262	MH0197_GL0121100	COG1653	ABC-type glycerol-3-phosphate transport system, periplasmic component	Carbohydrate transport and metabolism [G]	ABC transporters	Clostridiales bacterium KLE1615	Species	Firmicutes	Clostridia	Clostridiales			Clostridiales bacterium KLE1615	0.0688	0.6635		-0.3401	0.0343	*	0.1173	0.3697	
**335**	23510	MH0200_GL0014998	COG0280	Phosphotransacetylase	Energy production and conversion [C]	Taurine and hypotaurine metabolism	Clostridiales	Order	Firmicutes	Clostridia	Clostridiales				0.0277	0.7731		-0.0634	0.5265		0.0096	0.9039	
**336**	23525	MH0200_GL0037813	COG1145	Ferredoxin	Energy production and conversion [C]	Glycolysis / Gluconeogenesis	Clostridiales	Order	Firmicutes	Clostridia	Clostridiales				-0.1311	0.4429		0.1472	0.4092		-0.1579	0.2637	
**337**	23643	MH0200_GL0183371	COG0099	Ribosomal protein S13	Translation, ribosomal structure and biogenesis [J]	Ribosome	Bifidobacteriaceae	Family	Actinobacteria	Actinobacteria	Bifidobacteriales	Bifidobacteriaceae			-0.0446	0.7155		-0.0662	0.6037		0.0042	0.9670	
**338**	23687	MH0203_GL0013763	COG0057	Glyceraldehyde-3-phosphate dehydrogenase/erythrose-4-phosphate dehydrogenase	Carbohydrate transport and metabolism [G]	Glycolysis / Gluconeogenesis	Bifidobacterium	Genus	Actinobacteria	Actinobacteria	Bifidobacteriales	Bifidobacteriaceae	Bifidobacterium		0.0524	0.7398		-0.0044	0.9787		0.1397	0.2834	
**339**	23777	MH0203_GL0101461	COG0080	Ribosomal protein L11	Translation, ribosomal structure and biogenesis [J]	Ribosome	Bifidobacterium bifidum	Species	Actinobacteria	Actinobacteria	Bifidobacteriales	Bifidobacteriaceae	Bifidobacterium	Bifidobacterium bifidum	-0.1340	0.1889		0.0549	0.6094		-0.0156	0.8555	
**340**	23809	MH0203_GL0120151	COG0460	Homoserine dehydrogenase	Amino acid transport and metabolism [E]	Glycine, serine and threonine metabolism	Bifidobacterium	Genus	Actinobacteria	Actinobacteria	Bifidobacteriales	Bifidobacteriaceae	Bifidobacterium		-0.0313	0.7713		-0.0601	0.5928		-0.0356	0.6905	
**341**	23820	MH0203_GL0130171	COG0235	Ribulose-5-phosphate 4-epimerase/Fuculose-1-phosphate aldolase	Carbohydrate transport and metabolism [G]	Pentose and glucuronate interconversions	Blautia obeum	Species	Firmicutes	Clostridia	Clostridiales	Lachnospiraceae	Blautia	Blautia obeum	0.1653	0.1565		-0.1316	0.2828		-0.0480	0.6244	
**342**	23823	MH0203_GL0133062	COG0033	Phosphoglucomutase	Carbohydrate transport and metabolism [G]	Glycolysis / Gluconeogenesis	Bifidobacterium	Genus	Actinobacteria	Actinobacteria	Bifidobacteriales	Bifidobacteriaceae	Bifidobacterium		0.0550	0.7542		-0.2017	0.2683		-0.0417	0.7748	
**343**	23826	MH0203_GL0134326			NAN	NAN	Dorea formicigenerans	Species	Firmicutes	Clostridia	Clostridiales	Lachnospiraceae	Dorea	Dorea formicigenerans	-0.0279	0.7995		-0.1031	0.3669		-0.1078	0.2332	
**344**	23919	MH0203_GL0245882	COG0366	Glycosidase	Carbohydrate transport and metabolism [G]	NAN	Bifidobacterium longum	Species	Actinobacteria	Actinobacteria	Bifidobacteriales	Bifidobacteriaceae	Bifidobacterium	Bifidobacterium longum	-0.0447	0.8317		-0.0752	0.7322		-0.0875	0.6158	

**345**	23949	MH0204_GL0017355	COG0186	Ribosomal protein S17	Translation, ribosomal structure and biogenesis [J]	Ribosome	Faecalibacterium prausnitzii	Species	Firmicutes	Clostridia	Clostridiales	Ruminococcaceae	Faecalibacterium	Faecalibacterium prausnitzii	0.0385	0.6709		-0.0892	0.3430		0.0252	0.7376	
**346**	24576	MH0220_GL0013104	COG1882	Pyruvate-formate lyase	Energy production and conversion [C]	Pyruvate metabolism	Blautia obeum	Species	Firmicutes	Clostridia	Clostridiales	Lachnospiraceae	Blautia	Blautia obeum	0.1330	0.3152		-0.1058	0.4455		0.0047	0.9659	
**347**	24593	MH0220_GL0083105	COG0049	Ribosomal protein S7	Translation, ribosomal structure and biogenesis [J]	Ribosome	Collinsella	Genus	Actinobacteria	Coriobacteriia	Coriobacteriales	Coriobacteriaceae	Collinsella		-0.1860	0.0757		0.1199	0.2794		-0.0539	0.5423	
**348**	24695	MH0221_GL0065038			NAN	NAN	Bilophila wadsworthia	Species	Proteobacteria	Deltaproteobacteria	Desulfovibrionales	Desulfovibrionaceae	Bilophila	Bilophila wadsworthia	0.1625	0.0298	*	0.1135	0.1533		-0.0838	0.1856	
**349**	24743	MH0222_GL0076490	COG1145	Ferredoxin	Energy production and conversion [C]	Glycolysis / Gluconeogenesis	Clostridiales	Order	Firmicutes	Clostridia	Clostridiales				0.0745	0.6078		-0.1289	0.3940		-0.0360	0.7657	
**350**	24946	MH0227_GL0100580	COG0057	Glyceraldehyde-3-phosphate dehydrogenase/erythrose-4-phosphate dehydrogenase	Carbohydrate transport and metabolism [G]	Glycolysis / Gluconeogenesis	Bacteroides eggerthii	Species	Bacteroidetes	Bacteroidia	Bacteroidales	Bacteroidaceae	Bacteroides	Bacteroides eggerthii	0.0759	0.6537		-0.0718	0.6845		0.0187	0.8940	
**351**	24978	MH0227_GL0147089			NAN	NAN	Subdoligranulum variabile	Species	Firmicutes	Clostridia	Clostridiales	Ruminococcaceae	Subdoligranulum	Subdoligranulum variabile	-0.0551	0.7015		-0.1494	0.3171		0.0969	0.4153	
**352**	25045	MH0229_GL0083635	COG1185	Polyribonucleotide nucleotidyltransferase (polynucleotide phosphorylase)	Translation, ribosomal structure and biogenesis [J]	Purine metabolism	Faecalibacterium prausnitzii	Species	Firmicutes	Clostridia	Clostridiales	Ruminococcaceae	Faecalibacterium	Faecalibacterium prausnitzii	-0.2545	0.0795		0.1468	0.3397		0.1477	0.2250	
**353**	25052	MH0229_GL0094180	COG4939	Major membrane immunogen, membrane-anchored lipoprotein	Function unknown [S]	Citrate cycle (TCA cycle)	Faecalibacterium prausnitzii	Species	Firmicutes	Clostridia	Clostridiales	Ruminococcaceae	Faecalibacterium	Faecalibacterium prausnitzii	-0.1345	0.2753		0.0597	0.6447		0.0889	0.3863	
**354**	25430	MH0233_GL0035314	COG1879	ABC-type sugar transport system, periplasmic component, contains N-terminal xre family HTH domain	Carbohydrate transport and metabolism [G]	ABC transporters	[Ruminococcus] torques	Species	Firmicutes	Clostridia	Clostridiales	Lachnospiraceae	Blautia	[Ruminococcus] torques	0.2784	0.1195		-0.0549	0.7723		0.0997	0.5073	
**355**	25495	MH0233_GL0079986	COG1882	Pyruvate-formate lyase	Energy production and conversion [C]	Pyruvate metabolism	[Ruminococcus] torques	Species	Firmicutes	Clostridia	Clostridiales	Lachnospiraceae	Blautia	[Ruminococcus] torques	0.1006	0.6152		-0.1701	0.4144		0.0496	0.7657	
**356**	25534	MH0233_GL0108503	COG1653	ABC-type glycerol-3-phosphate transport system, periplasmic component	Carbohydrate transport and metabolism [G]	NAN	Blautia	Genus	Firmicutes	Clostridia	Clostridiales	Lachnospiraceae	Blautia		-0.1869	0.2284		-0.1911	0.2385		-0.0110	0.9323	
**357**	25618	MH0236_GL0022013	COG0191	Fructose/tagatose bisphosphate aldolase	Carbohydrate transport and metabolism [G]	Glycolysis / Gluconeogenesis	Faecalibacterium prausnitzii	Species	Firmicutes	Clostridia	Clostridiales	Ruminococcaceae	Faecalibacterium	Faecalibacterium prausnitzii	-0.2148	0.0860		-0.0587	0.6592		0.1607	0.1230	
**358**	25664	MH0238_GL0027853	COG1653	ABC-type glycerol-3-phosphate transport system, periplasmic component	Carbohydrate transport and metabolism [G]	ABC transporters	Firmicutes	Species	Firmicutes	Clostridia	Clostridiales	Lachnospiraceae	Butyrivibrio	Butyrivibrio crossotus	-0.2080	0.1358		0.0571	0.6987		0.0715	0.5417	
**359**	25714	MH0238_GL0165578	COG1454	Alcohol dehydrogenase, class IV	Energy production and conversion [C]	Glycolysis / Gluconeogenesis	Ruminococcaceae	Species	Firmicutes	Clostridia	Clostridiales	Ruminococcaceae	Faecalibacterium	Faecalibacterium prausnitzii	-0.1864	0.1570		-0.0259	0.8524		-0.0914	0.4071	
**360**	25756	MH0239_GL0018928	COG1145	Ferredoxin	Energy production and conversion [C]	Glycolysis / Gluconeogenesis	Clostridiales	Order	Firmicutes	Clostridia	Clostridiales				-0.0950	0.5199		-0.0785	0.6112		-0.0136	0.9116	
**361**	25836	MH0239_GL0084186	COG1145	Ferredoxin	Energy production and conversion [C]	Glycolysis / Gluconeogenesis	Clostridiales	Species	Firmicutes	Clostridia	Clostridiales	Ruminococcaceae	Faecalibacterium	Faecalibacterium prausnitzii	-0.0624	0.6648		0.0176	0.9072		-0.2518	0.0302	*
**362**	25842	MH0239_GL0088549	COG0747	ABC-type transport system, periplasmic component	Amino acid transport and metabolism [E]	NAN	Clostridiales	Order	Firmicutes	Clostridia	Clostridiales				0.1181	0.2685		-0.0356	0.7511		-0.0537	0.5468	
**363**	25966	MH0243_GL0019682	COG0149	Triosephosphate isomerase	Carbohydrate transport and metabolism [G]	Glycolysis / Gluconeogenesis	Ruminococcus bromii	Species	Firmicutes	Clostridia	Clostridiales	Ruminococcaceae	Ruminococcus	Ruminococcus bromii	-0.1934	0.2141		0.0892	0.5863		-0.2177	0.0892	
**364**	26279	MH0246_GL0124498	COG1070	Sugar (pentulose or hexulose) kinase	Carbohydrate transport and metabolism [G]	Pentose phosphate pathway	Bifidobacterium	Genus	Actinobacteria	Actinobacteria	Bifidobacteriales	Bifidobacteriaceae	Bifidobacterium		0.0200	0.9053		-0.0454	0.7963		-0.1974	0.1526	
**365**	26284	MH0246_GL0129256			NAN	NAN	Oscillibacter sp. KLE 1745	Species	Firmicutes	Clostridia	Clostridiales	Oscillospiraceae	Oscillibacter	Oscillibacter sp. KLE 1745	0.2901	0.2418		0.1229	0.6375		-0.3015	0.1407	
**366**	26552	MH0251_GL0023414	COG1024	Enoyl-CoA hydratase/carnithine racemase	Lipid transport and metabolism [I]	NAN	Faecalibacterium prausnitzii	Species	Firmicutes	Clostridia	Clostridiales	Ruminococcaceae	Faecalibacterium	Faecalibacterium prausnitzii	0.0643	0.7314		0.0411	0.8337		-0.0859	0.5804	
**367**	26559	MH0251_GL0036895	COG0427	Acyl-CoA hydrolase	Energy production and conversion [C]	Pyruvate metabolism	Faecalibacterium prausnitzii	Species	Firmicutes	Clostridia	Clostridiales	Ruminococcaceae	Faecalibacterium	Faecalibacterium prausnitzii	0.0706	0.5636		-0.0949	0.4566		0.1141	0.2581	
**368**	26571	MH0251_GL0069201	COG1653	ABC-type glycerol-3-phosphate transport system, periplasmic component	Carbohydrate transport and metabolism [G]	NAN	Clostridiales	Order	Firmicutes	Clostridia	Clostridiales				-0.2721	0.0094	**	-0.0324	0.7762		-0.0597	0.5097	
**369**	26605	MH0251_GL0128679	COG1614	CO dehydrogenase/acetyl-CoA synthase beta subunit	Energy production and conversion [C]	Methane metabolism	Clostridiales	Order	Firmicutes	Clostridia	Clostridiales				0.1703	0.0211	*	-0.0598	0.4517		-0.0198	0.7548	
**370**	26897	MH0259_GL0033209	COG1653	ABC-type glycerol-3-phosphate transport system, periplasmic component	Carbohydrate transport and metabolism [G]	ABC transporters	Bifidobacterium adolescentis	Species	Actinobacteria	Actinobacteria	Bifidobacteriales	Bifidobacteriaceae	Bifidobacterium	Bifidobacterium adolescentis	-0.3243	0.0479	*	0.0917	0.6011		-0.0770	0.5804	

**371**	27099	MH0262_GL0083048	COG3716	Phosphotransferase system, mannose/fructose/N-acetylgalactosamine-specific component IID	Carbohydrate transport and metabolism [G]	Fructose and mannose metabolism	Collinsella	Genus	Actinobacteria	Coriobacteriia	Coriobacteriales	Coriobacteriaceae	Collinsella		-0.3490	0.0264	*	0.0225	0.8945		-0.0674	0.6164	
**372**	27223	MH0265_GL0114475	COG0094	Ribosomal protein L5	Translation, ribosomal structure and biogenesis [J]	Ribosome	Bifidobacterium	Genus	Actinobacteria	Actinobacteria	Bifidobacteriales	Bifidobacteriaceae	Bifidobacterium		0.0635	0.6523		-0.1499	0.3055		0.0132	0.9100	
**373**	27643	MH0272_GL0188768	COG0097	Ribosomal protein L6P/L9E	Translation, ribosomal structure and biogenesis [J]	Ribosome	Bifidobacterium	Genus	Actinobacteria	Actinobacteria	Bifidobacteriales	Bifidobacteriaceae	Bifidobacterium		-0.1315	0.2865		-0.0356	0.7834		-0.0011	0.9914	
**374**	27673	MH0274_GL0008807	COG1080	Phosphoenolpyruvate-protein kinase (PTS system EI component in bacteria)	Carbohydrate transport and metabolism [G]	Pyruvate metabolism	Clostridiales	Order	Firmicutes	Clostridia	Clostridiales				-0.0693	0.6991		0.0058	0.9754		-0.0982	0.5085	
**375**	27677	MH0274_GL0010540	COG0056	FoF1-type ATP synthase, alpha subunit	Energy production and conversion [C]	Oxidative phosphorylation	Clostridiales	Order	Firmicutes	Clostridia	Clostridiales				-0.0826	0.3213		-0.1098	0.2050		-0.0213	0.7590	
**376**	27731	MH0274_GL0075860	COG0539	Ribosomal protein S1	Translation, ribosomal structure and biogenesis [J]	Ribosome	Bifidobacterium	Genus	Actinobacteria	Actinobacteria	Bifidobacteriales	Bifidobacteriaceae	Bifidobacterium		-0.0978	0.5688		-0.1261	0.4809		0.0379	0.7904	
**377**	27827	MH0274_GL0171300	COG0334	Glutamate dehydrogenase/leucine dehydrogenase	Amino acid transport and metabolism [E]	Alanine, aspartate and glutamate metabolism	Clostridiales	Order	Firmicutes	Clostridia	Clostridiales				-0.1823	0.1486		0.1229	0.3549		-0.0933	0.3774	
**378**	27885	MH0275_GL0131386	COG0334	Glutamate dehydrogenase/leucine dehydrogenase	Amino acid transport and metabolism [E]	Alanine, aspartate and glutamate metabolism	Clostridiales	Order	Firmicutes	Clostridia	Clostridiales				-0.0314	0.7575		-0.0988	0.3490		0.0351	0.6774	
**379**	28126	MH0280_GL0137988	COG0088	Ribosomal protein L4	Translation, ribosomal structure and biogenesis [J]	Ribosome	Subdoligranulum variabile	Species	Firmicutes	Clostridia	Clostridiales	Ruminococcaceae	Subdoligranulum	Subdoligranulum variabile	0.0719	0.3356		0.0587	0.4530		-0.0577	0.3520	
**380**	28314	MH0284_GL0116366	COG0183	Acetyl-CoA acetyltransferase	Lipid transport and metabolism [I]	Fatty acid degradation	Clostridiales	Order	Firmicutes	Clostridia	Clostridiales				-0.1106	0.6541		0.3384	0.1850		-0.1076	0.5993	
**381**	28480	MH0288_GL0054326	COG4774	Outer membrane receptor for monomeric catechols	Inorganic ion transport and metabolism [P]	NAN	Bacteroides vulgatus	Species	Bacteroidetes	Bacteroidia	Bacteroidales	Bacteroidaceae	Bacteroides	Bacteroides vulgatus	0.6661	0.0003	***	0.0560	0.7889		0.0092	0.9559	
**382**	28510	MH0288_GL0093098	COG1024	Enoyl-CoA hydratase/carnithine racemase	Lipid transport and metabolism [I]	Fatty acid degradation	Lachnospiraceae	Family	Firmicutes	Clostridia	Clostridiales	Lachnospiraceae			-0.2030	0.1330		0.1658	0.2428		0.1736	0.1212	
**383**	28604	N051A_GL0052937			NAN	NAN	Faecalibacterium prausnitzii	Species	Firmicutes	Clostridia	Clostridiales	Ruminococcaceae	Faecalibacterium	Faecalibacterium prausnitzii	-0.1385	0.5638		-0.0203	0.9357		-0.0330	0.8688	
**384**	28653	MH0290_GL0142605	COG2407	L-fucose isomerase or related protein	Carbohydrate transport and metabolism [G]	Fructose and mannose metabolism	Lachnospiraceae	Family	Firmicutes	Clostridia	Clostridiales	Lachnospiraceae			-0.2363	0.0531		0.0117	0.9290		0.1187	0.2491	
**385**	28676	MH0292_GL0072575			NAN	NAN	Clostridiales	Order	Firmicutes	Clostridia	Clostridiales				0.0581	0.5993		0.1036	0.3685		-0.0393	0.6692	
**386**	28707	MH0293_GL0072012	COG0031	Cysteine synthase	Amino acid transport and metabolism [E]	Glycine, serine and threonine metabolism	Clostridiales	Order	Firmicutes	Clostridia	Clostridiales				0.0377	0.6829		0.0908	0.3442		0.1129	0.1356	
**387**	28712	MH0293_GL0090794	COG1080	Phosphoenolpyruvate-protein kinase (PTS system EI component in bacteria)	Carbohydrate transport and metabolism [G]	Pyruvate metabolism	Clostridiales	Order	Firmicutes	Clostridia	Clostridiales				-0.2021	0.4560		-0.0091	0.9743		0.0270	0.9048	
**388**	29633	MH0321_GL0040229	COG1350	Predicted alternative tryptophan synthase beta-subunit (paralog of TrpB)	Amino acid transport and metabolism [E]	Glycine, serine and threonine metabolism	Ruminococcus	Genus	Firmicutes	Clostridia	Clostridiales	Ruminococcaceae	Ruminococcus		-0.2347	0.0735		0.3567	0.0076	**	-0.2121	0.0504	
**389**	29645	MH0321_GL0083392	COG0097	Ribosomal protein L6P/L9E	Translation, ribosomal structure and biogenesis [J]	Ribosome	Subdoligranulum variabile	Species	Firmicutes	Clostridia	Clostridiales	Ruminococcaceae	Subdoligranulum	Subdoligranulum variabile	0.1012	0.1418		-0.0412	0.5717		0.0743	0.1949	
**390**	29780	MH0326_GL0040294	COG3959	Transketolase, N-terminal subunit	Carbohydrate transport and metabolism [G]	Pentose phosphate pathway	Clostridiales	Order	Firmicutes	Clostridia	Clostridiales				-0.0300	0.6800		-0.0647	0.3930		-0.0301	0.6181	
**391**	29830	MH0327_GL0023365	COG1866	Phosphoenolpyruvate carboxykinase, ATP-dependent	Energy production and conversion [C]	Glycolysis / Gluconeogenesis	Faecalibacterium prausnitzii	Species	Firmicutes	Clostridia	Clostridiales	Ruminococcaceae	Faecalibacterium	Faecalibacterium prausnitzii	-0.1105	0.1899		-0.0337	0.7050		0.0400	0.5711	
**392**	29843	MH0327_GL0049100	COG0443	Molecular chaperone DnaK (HSP70)	Posttranslational modification, protein turnover, chaperones [O]	RNA degradation	Collinsella aerofaciens	Species	Actinobacteria	Coriobacteriia	Coriobacteriales	Coriobacteriaceae	Collinsella	Collinsella aerofaciens	-0.4156	0.0063	**	-0.0741	0.6560		-0.1209	0.3586	
**393**	29850	MH0327_GL0058501	COG0191	Fructose/tagatose bisphosphate aldolase	Carbohydrate transport and metabolism [G]	Glycolysis / Gluconeogenesis	Subdoligranulum variabile	Species	Firmicutes	Clostridia	Clostridiales	Ruminococcaceae	Subdoligranulum	Subdoligranulum variabile	0.0778	0.4577		-0.0942	0.3888		0.0771	0.3742	
**394**	30067	MH0330_GL0174601	COG1825	Ribosomal protein L25 (general stress protein Ctc)	Translation, ribosomal structure and biogenesis [J]	Ribosome	Bifidobacterium	Genus	Actinobacteria	Actinobacteria	Bifidobacteriales	Bifidobacteriaceae	Bifidobacterium		0.1277	0.3997		0.0354	0.8237		0.0181	0.8864	
**395**	30149	MH0333_GL0069706	COG4822	Cobalamin biosynthesis protein CbiK, Co2+ chelatase	Coenzyme transport and metabolism [H]	Porphyrin and chlorophyll metabolism	Clostridiales	Order	Firmicutes	Clostridia	Clostridiales				0.1406	0.3550		-0.1439	0.3648		-0.0274	0.8287	

**396**	30175	MH0333_GL0121497	COG3209	Uncharacterized conserved protein RhaS, contains 28 RHS repeats	General function prediction only [R]	NAN	Blautia	Species	Firmicutes	Clostridia	Clostridiales	Lachnospiraceae	Blautia	Blautia wexlerae	0.4518	0.0286	*	0.1384	0.5325		0.0086	0.9610	
**397**	30545	MH0341_GL0000384	COG0297	Glycogen synthase(	Carbohydrate transport and metabolism [G]	Galactose metabolism	Faecalibacterium prausnitzii	Species	Firmicutes	Clostridia	Clostridiales	Ruminococcaceae	Faecalibacterium	Faecalibacterium prausnitzii	-0.1791	0.2112		-0.0141	0.9257		0.0323	0.7879	
**398**	30556	MH0341_GL0013504	COG0176	Transaldolase	Carbohydrate transport and metabolism [G]	Pentose phosphate pathway	Bifidobacterium	Genus	Actinobacteria	Actinobacteria	Bifidobacteriales	Bifidobacteriaceae	Bifidobacterium		-0.0663	0.7930		0.0397	0.8806		0.2581	0.2144	
**399**	30576	MH0341_GL0038266	COG0469	Pyruvate kinase	Carbohydrate transport and metabolism [G]	Glycolysis / Gluconeogenesis	Bifidobacterium	Genus	Actinobacteria	Actinobacteria	Bifidobacteriales	Bifidobacteriaceae	Bifidobacterium		-0.1530	0.2408		-0.1205	0.3784		0.0243	0.8240	
**400**	30617	MH0341_GL0088523	COG0281	Malic enzyme	Energy production and conversion [C]	Pyruvate metabolism	Clostridiales	Order	Firmicutes	Clostridia	Clostridiales				0.2349	0.1070		-0.2709	0.0740		0.0996	0.4164	
**401**	30623	MH0341_GL0099782	COG0050	Translation elongation factor EF-Tu, a GTPase(	Translation, ribosomal structure and biogenesis [J]	Plant-pathogen interaction	Bifidobacterium longum	Species	Actinobacteria	Actinobacteria	Bifidobacteriales	Bifidobacteriaceae	Bifidobacterium	Bifidobacterium longum	0.0020	0.9896		-0.0608	0.7037		0.0674	0.5957	
**402**	30641	MH0341_GL0114479	COG0277	FAD/FMN-containing dehydrogenase	Energy production and conversion [C]	Pyruvate metabolism	Firmicutes	Phylum	Firmicutes						-0.3440	0.0131	*	-0.0469	0.7552		-0.0027	0.9819	
**403**	30834	MH0347_GL0010219	COG1653	ABC-type glycerol-3-phosphate transport system, periplasmic component	Carbohydrate transport and metabolism [G]	NAN	Bifidobacterium	Genus	Actinobacteria	Actinobacteria	Bifidobacteriales	Bifidobacteriaceae	Bifidobacterium		0.1351	0.5164		-0.1043	0.6321		0.0548	0.7517	
**404**	30909	MH0348_GL0122167	COG0085	DNA-directed RNA polymerase, beta subunit/140 kD subunit	Transcription [K]	Purine metabolism	Bifidobacterium	Genus	Actinobacteria	Actinobacteria	Bifidobacteriales	Bifidobacteriaceae	Bifidobacterium		-0.0143	0.9355		-0.2779	0.1259		0.0573	0.6953	
**405**	30964	MH0350_GL0092393	COG2407	L-fucose isomerase or related protein	Carbohydrate transport and metabolism [G]	Fructose and mannose metabolism	Clostridiales	Order	Firmicutes	Clostridia	Clostridiales				-0.0560	0.7681		-0.3917	0.0429	*	-0.1637	0.2962	
**406**	31096	MH0355_GL0038120	COG1014	Pyruvate:ferredoxin oxidoreductase or related 2-oxoacid:ferredoxin oxidoreductase, gamma subunit	Energy production and conversion [C]	Glycolysis / Gluconeogenesis	Lachnospiraceae	Family	Firmicutes	Clostridia	Clostridiales	Lachnospiraceae			-0.0169	0.9169		-0.0070	0.9671		-0.1149	0.3898	
**407**	31170	MH0356_GL0041566	COG0057	Glyceraldehyde-3-phosphate dehydrogenase/erythrose-4-phosphate dehydrogenase(	Carbohydrate transport and metabolism [G]	Glycolysis / Gluconeogenesis	Bifidobacterium	Genus	Actinobacteria	Actinobacteria	Bifidobacteriales	Bifidobacteriaceae	Bifidobacterium		-0.1349	0.1342		0.0603	0.5267		0.0631	0.4036	
**408**	31210	MH0356_GL0130219	COG0055	FoF1-type ATP synthase, beta subunit	Energy production and conversion [C]	Oxidative phosphorylation	Bifidobacterium	Genus	Actinobacteria	Actinobacteria	Bifidobacteriales	Bifidobacteriaceae	Bifidobacterium		-0.2335	0.1976		-0.0661	0.7294		-0.0253	0.8677	
**409**	31211	MH0356_GL0130221	COG0056	FoF1-type ATP synthase, alpha subunit(1	Energy production and conversion [C]	Oxidative phosphorylation	Bifidobacterium	Genus	Actinobacteria	Actinobacteria	Bifidobacteriales	Bifidobacteriaceae	Bifidobacterium		-0.0954	0.5175		-0.1216	0.4291		0.0777	0.5257	
**410**	31216	MH0356_GL0133302	COG0459	Chaperonin GroEL (HSP60 family)	Posttranslational modification, protein turnover, chaperones [O]	RNA degradation	Bifidobacterium pseudocatenulatum	Species	Actinobacteria	Actinobacteria	Bifidobacteriales	Bifidobacteriaceae	Bifidobacterium	Bifidobacterium pseudocatenulatum	-0.1376	0.2700		-0.0653	0.6186		0.0745	0.4739	
**411**	31253	MH0356_GL0190005	COG0747	ABC-type transport system, periplasmic component	Amino acid transport and metabolism [E]	ABC transporters	Lachnospiraceae	Family	Firmicutes	Clostridia	Clostridiales	Lachnospiraceae			0.2036	0.1831		0.1190	0.4598		0.1218	0.3401	
**412**	31485	MH0359_GL0028504	COG0091	Ribosomal protein L22	Translation, ribosomal structure and biogenesis [J]	Ribosome	Ruminococcaceae	Family	Firmicutes	Clostridia	Clostridiales	Ruminococcaceae			-0.0391	0.7242		0.0924	0.4237		-0.0135	0.8837	
**413**	31551	MH0360_GL0125000	COG0480	Translation elongation factor EF-G, a GTPase	Translation, ribosomal structure and biogenesis [J]	NAN	Faecalibacterium prausnitzii	Species	Firmicutes	Clostridia	Clostridiales	Ruminococcaceae	Faecalibacterium	Faecalibacterium prausnitzii	-0.0619	0.6856		-0.0686	0.6676		-0.0939	0.4586	
**414**	31642	MH0363_GL0035255	COG1145	Ferredoxin	Energy production and conversion [C]	Glycolysis / Gluconeogenesis	Eubacterium	Genus	Firmicutes	Clostridia	Clostridiales	Eubacteriaceae	Eubacterium		-0.1477	0.1133		-0.0050	0.9599		-0.0303	0.7001	
**415**	31655	MH0363_GL0154863	COG1653	ABC-type glycerol-3-phosphate transport system, periplasmic component	Carbohydrate transport and metabolism [G]	ABC transporters	Firmicutes	Phylum	Firmicutes						-0.3930	0.0253	*	-0.1017	0.5908		0.0546	0.7165	
**416**	31674	MH0364_GL0101039	COG0057	Glyceraldehyde-3-phosphate dehydrogenase/erythrose-4-phosphate dehydrogenase	Carbohydrate transport and metabolism [G]	Glycolysis / Gluconeogenesis	Ruminococcus bicirculans	Species	Firmicutes	Clostridia	Clostridiales	Ruminococcaceae	Ruminococcus	Ruminococcus bicirculans	-0.3861	0.0816		0.2449	0.2961		0.0299	0.8735	
**417**	31742	MH0367_GL0050319	COG1592	Rubrerythrin	Energy production and conversion [C]	NAN	Firmicutes	Phylum	Firmicutes						-0.2127	0.0332	*	0.0208	0.8460		0.0726	0.3927	
**418**	31758	MH0367_GL0121504	COG0149	Triosephosphate isomerase	Carbohydrate transport and metabolism [G]	Glycolysis / Gluconeogenesis	Firmicutes	Phylum	Firmicutes						0.0146	0.8883		-0.0261	0.8104		-0.0076	0.9295	
**419**	31916	MH0370_GL0042621	COG4771	Outer membrane receptor for ferrienterochelin and colicins	Inorganic ion transport and metabolism [P]	NAN	Bacteroides uniformis	Species	Bacteroidetes	Bacteroidia	Bacteroidales	Bacteroidaceae	Bacteroides	Bacteroides uniformis	0.1930	0.3900		-0.1186	0.6141		0.0123	0.9474	
**420**	31959	MH0370_GL0059817	COG0832	Urease beta subunit	Amino acid transport and metabolism [E]	Purine metabolism	Bacteria	Superkingdom							0.0630	0.5310		0.1009	0.3346		-0.0203	0.8085	

**421**	32014	MH0370_GL0093205	COG2222	Fructoselysine-6-P-deglycase FrlB and related proteins with duplicated sugar isomerase (SIS) domain	Cell wall/membrane/envelope biogenesis [M]	Alanine, aspartate and glutamate metabolism	Collinsella aerofaciens	Species	Actinobacteria	Coriobacteriia	Coriobacteriales	Coriobacteriaceae	Collinsella	Collinsella aerofaciens	-0.2652	0.0412	*	0.0715	0.6075		0.0725	0.5117	
**422**	32128	MH0371_GL0055922	COG1538	Outer membrane protein TolC	Cell wall/membrane/envelope biogenesis [M]	Bacterial secretion system	Bacteroides	Genus	Bacteroidetes	Bacteroidia	Bacteroidales	Bacteroidaceae	Bacteroides		0.4613	0.0045	**	0.2127	0.2292		0.0681	0.6309	
**423**	32214	MH0372_GL0099061	COG0098	Ribosomal protein S5(	Translation, ribosomal structure and biogenesis [J]	Ribosome	Bifidobacterium	Genus	Actinobacteria	Actinobacteria	Bifidobacteriales	Bifidobacteriaceae	Bifidobacterium		-0.1000	0.3984		-0.1454	0.2376		0.0107	0.9136	
**424**	32712	MH0383_GL0119529	COG0126	3-phosphoglycerate kinase	Carbohydrate transport and metabolism [G]	Glycolysis / Gluconeogenesis	Ruminococcus bromii	Species	Firmicutes	Clostridia	Clostridiales	Ruminococcaceae	Ruminococcus	Ruminococcus bromii	-0.2344	0.2154		0.3604	0.0649		-0.1461	0.3544	
**425**	32757	MH0385_GL0010518	COG0443	Molecular chaperone DnaK (HSP70)	Posttranslational modification, protein turnover, chaperones [O]	RNA degradation	Firmicutes	Phylum	Firmicutes						0.0316	0.6594		-0.0348	0.6421		0.0357	0.5481	
**426**	33066	MH0391_GL0150753	COG0087	Ribosomal protein L3	Translation, ribosomal structure and biogenesis [J]	Ribosome	Clostridium	Genus	Firmicutes	Clostridia	Clostridiales	Clostridiaceae	Clostridium		0.1122	0.0757		-0.0115	0.8645		0.0713	0.1780	
**427**	33207	MH0396_GL0047332	COG3842	ABC-type Fe3+/spermidine/putrescine transport systems, ATPase components	Amino acid transport and metabolism [E]	ABC transporters	Clostridiales	Order	Firmicutes	Clostridia	Clostridiales				0.0305	0.8394		0.3136	0.0407	*	-0.0349	0.7805	
**428**	33402	MH0400_GL0071356			NAN	NAN	Ruminococcus bromii	Species	Firmicutes	Clostridia	Clostridiales	Ruminococcaceae	Ruminococcus	Ruminococcus bromii	-0.2488	0.2223		0.1446	0.4999		-0.1152	0.4989	
**429**	33560	O2.UC28-0_GL0204562	COG0254	Ribosomal protein L31	Translation, ribosomal structure and biogenesis [J]	Ribosome	Clostridiales	Order	Firmicutes	Clostridia	Clostridiales				0.1622	0.0584		-0.0950	0.2960		0.0866	0.2300	
**430**	33702	MH0406_GL0015896	COG1592	Rubrerythrin	Energy production and conversion [C]	NAN	Collinsella	Genus	Actinobacteria	Coriobacteriia	Coriobacteriales	Coriobacteriaceae	Collinsella		-0.2206	0.1194		-0.0717	0.6327		-0.1133	0.3401	
**431**	33861	MH0407_GL0191700	COG0246	Mannitol-1-phosphate/altronate dehydrogenases	Carbohydrate transport and metabolism [G]	Pentose and glucuronate interconversions	Clostridium	Genus	Firmicutes	Clostridia	Clostridiales	Clostridiaceae	Clostridium		0.0302	0.6592		-0.0896	0.2058		0.0515	0.3625	
**432**	34201	MH0415_GL0203137	COG0104	Adenylosuccinate synthase	Nucleotide transport and metabolism [F]	Purine metabolism	Ruminococcus bromii	Species	Firmicutes	Clostridia	Clostridiales	Ruminococcaceae	Ruminococcus	Ruminococcus bromii	-0.0390	0.6936		0.1325	0.1953		-0.1871	0.0183	*
**433**	34219	MH0416_GL0018356	COG5275	BRCT domain type II	General function prediction only [R]	Arginine and proline metabolism	Dorea longicatena	Species	Firmicutes	Clostridia	Clostridiales	Lachnospiraceae	Dorea	Dorea longicatena	-0.1282	0.3049		-0.1132	0.3863		-0.0804	0.4396	
**434**	34380	MH0419_GL0081674	COG0228	Ribosomal protein S16	Translation, ribosomal structure and biogenesis [J]	Ribosome	Bifidobacterium	Genus	Actinobacteria	Actinobacteria	Bifidobacteriales	Bifidobacteriaceae	Bifidobacterium		0.0175	0.8914		0.0388	0.7726		0.0215	0.8403	
**435**	34449	MH0086_GL0032208	COG0539	Ribosomal protein S1	Translation, ribosomal structure and biogenesis [J]	Ribosome	Lachnospiraceae	Family	Firmicutes	Clostridia	Clostridiales	Lachnospiraceae			-0.0095	0.8968		-0.1005	0.1831		0.0411	0.4969	
**436**	34610	MH0423_GL0103049	COG0088	Ribosomal protein L4	Translation, ribosomal structure and biogenesis [J]	Ribosome	Clostridiales	Order	Firmicutes	Clostridia	Clostridiales				-0.1821	0.0502		-0.1518	0.1215		0.0617	0.4333	
**437**	34973	MH0432_GL0050497	COG5263	Glucan-binding domain (YG repeat)	Carbohydrate transport and metabolism [G]	NAN	Clostridium	Genus	Firmicutes	Clostridia	Clostridiales	Clostridiaceae	Clostridium		0.1302	0.5800		0.0236	0.9236		-0.0032	0.9869	
**438**	35005	MH0432_GL0168684	COG0126	3-phosphoglycerate kinase	Carbohydrate transport and metabolism [G]	Glycolysis / Gluconeogenesis	Collinsella	Genus	Actinobacteria	Coriobacteriia	Coriobacteriales	Coriobacteriaceae	Collinsella		-0.1864	0.1736		0.0413	0.7753		-0.0610	0.5953	
**439**	35257	MH0435_GL0100291	COG0149	Triosephosphate isomerase	Carbohydrate transport and metabolism [G]	Glycolysis / Gluconeogenesis	Roseburia	Genus	Firmicutes	Clostridia	Clostridiales	Lachnospiraceae	Roseburia		0.0601	0.5445		-0.1254	0.2226		-0.0023	0.9777	
**440**	35519	MH0440_GL0182683	COG1454	Alcohol dehydrogenase, class IV	Energy production and conversion [C]	Glycolysis / Gluconeogenesis	Bacteria	Species	Actinobacteria	Coriobacteriia	Coriobacteriales	Coriobacteriaceae	Collinsella	Collinsella aerofaciens	-0.2461	0.2133		0.1532	0.4611		-0.2532	0.1209	
**441**	35568	MH0441_GL0130959	COG0334	Glutamate dehydrogenase/leucine dehydrogenase	Amino acid transport and metabolism [E]	Alanine, aspartate and glutamate metabolism	Clostridiales	Order	Firmicutes	Clostridia	Clostridiales				-0.0734	0.3542		0.1460	0.0733		-0.1924	0.0020	**
**442**	35789	MH0445_GL0066110	COG1866	Phosphoenolpyruvate carboxykinase, ATP-dependent	Energy production and conversion [C]	Glycolysis / Gluconeogenesis	Lachnospiraceae	Family	Firmicutes	Clostridia	Clostridiales	Lachnospiraceae			0.2493	0.0933		-0.0415	0.7926		-0.0282	0.8221	
**443**	35949	MH0447_GL0209994	COG0366	Glycosidase	Carbohydrate transport and metabolism [G]	Galactose metabolism	Ruminococcus bromii	Species	Firmicutes	Clostridia	Clostridiales	Ruminococcaceae	Ruminococcus	Ruminococcus bromii	-0.2482	0.0260	*	-0.0379	0.7527		-0.1094	0.2479	
**444**	35954	MH0447_GL0228814	COG4799	Acetyl-CoA carboxylase, carboxyltransferase component	Lipid transport and metabolism [I]	Fatty acid biosynthesis	Clostridiales	Order	Firmicutes	Clostridia	Clostridiales				0.1752	0.2291		-0.2322	0.1246		0.0359	0.7687	

**445**	35996	MH0448_GL0031695	COG0822	NifU homolog involved in Fe-S cluster formation	Posttranslational modification, protein turnover, chaperones [O]	NAN	Faecalibacterium prausnitzii	Species	Firmicutes	Clostridia	Clostridiales	Ruminococcaceae	Faecalibacterium	Faecalibacterium prausnitzii	-0.0669	0.7281		-0.1083	0.5894		-0.0994	0.5329	
**446**	36042	MH0448_GL0176244	COG1196	Chromosome segregation ATPase	Cell cycle control, cell division, chromosome partitioning [D]	NAN	Bacteroides dorei	Species	Bacteroidetes	Bacteroidia	Bacteroidales	Bacteroidaceae	Bacteroides	Bacteroides dorei	-0.1609	0.4849		0.1505	0.5317		0.0036	0.9851	
**447**	36197	MH0452_GL0206149	COG3203	Outer membrane protein (porin)	Cell wall/membrane/envelope biogenesis [M]	NAN	Dialister invisus	Species	Firmicutes	Negativicutes	Veillonellales	Veillonellaceae	Dialister	Dialister invisus	0.0331	0.7652		-0.1628	0.1540		-0.0544	0.5530	
**448**	36335	MH0455_GL0153195	COG0443	Molecular chaperone DnaK (HSP70)	Posttranslational modification, protein turnover, chaperones [O]	RNA degradation	Clostridiales	Order	Firmicutes	Clostridia	Clostridiales				0.1677	0.2139		-0.1175	0.4069		-0.0192	0.8652	
**449**	36549	N003A_GL0049315	COG1862	Preprotein translocase subunit YajC	Intracellular trafficking, secretion, and vesicular transport [U]	Protein export	Bacteroides	Genus	Bacteroidetes	Bacteroidia	Bacteroidales	Bacteroidaceae	Bacteroides		0.3573	0.0004	***	-0.0798	0.4833		0.0383	0.6726	
**450**	36622	N013A_GL0032025	COG0059	Ketol-acid reductoisomerase	Coenzyme transport and metabolism [H]	Valine, leucine and isoleucine biosynthesis	Eubacterium	Genus	Firmicutes	Clostridia	Clostridiales	Eubacteriaceae	Eubacterium		0.0153	0.9277		0.1153	0.5100		0.0057	0.9674	
**451**	36712	N025A_GL0031266	COG1653	ABC-type glycerol-3-phosphate transport system, periplasmic component	Carbohydrate transport and metabolism [G]	ABC transporters	Faecalibacterium prausnitzii	Species	Firmicutes	Clostridia	Clostridiales	Ruminococcaceae	Faecalibacterium	Faecalibacterium prausnitzii	-0.0454	0.7124		-0.1633	0.1995		0.1106	0.2756	
**452**	36866	N038A_GL0001480	COG1653	ABC-type glycerol-3-phosphate transport system, periplasmic component	Carbohydrate transport and metabolism [G]	NAN	Bacteria	Superkingdom							-0.0685	0.5452		-0.0444	0.7078		0.1397	0.1325	
**453**	36918	N042A_GL0025320	COG0012	Ribosome-binding ATPase YchF, GTP1/OBG family	Translation, ribosomal structure and biogenesis [J]	NAN	Clostridiales	Order	Firmicutes	Clostridia	Clostridiales				-0.1013	0.1510		0.1058	0.1509		0.0048	0.9359	
**454**	36972	N047A_GL0051881			NAN	NAN	Bacteroides	Genus	Bacteroidetes	Bacteroidia	Bacteroidales	Bacteroidaceae	Bacteroides		0.4491	0.0198	*	-0.0913	0.6611		0.2141	0.1914	
**455**	37011	N056A_GL0035081	COG0235	Ribulose-5-phosphate 4-epimerase/Fuculose-1-phosphate aldolase	Carbohydrate transport and metabolism [G]	Pentose and glucuronate interconversions	Clostridiales	Order	Firmicutes	Clostridia	Clostridiales				0.0528	0.6736		-0.0890	0.4953		-0.0630	0.5443	
**456**	37103	N079A_GL0025412	COG0049	Ribosomal protein S7	Translation, ribosomal structure and biogenesis [J]	Ribosome	Faecalibacterium prausnitzii	Species	Firmicutes	Clostridia	Clostridiales	Ruminococcaceae	Faecalibacterium	Faecalibacterium prausnitzii	0.0610	0.5475		-0.0320	0.7629		0.0534	0.5255	
**457**	37117	N082A_GL0072780	COG0747	ABC-type transport system, periplasmic component	Amino acid transport and metabolism [E]	NAN	Lachnoclostridium phytofermentans	Species	Firmicutes	Clostridia	Clostridiales	Lachnospiraceae	Lachnoclostridium	Lachnoclostridium phytofermentans	0.1254	0.1904		0.0263	0.7948		0.0562	0.4825	
**458**	37211	N086A_GL0096767	COG0092	Ribosomal protein S3	Translation, ribosomal structure and biogenesis [J]	Ribosome	Ruminococcaceae	Species	Firmicutes	Clostridia	Clostridiales	Ruminococcaceae	Subdoligranulum	Subdoligranulum variabile	0.1395	0.1147		0.1348	0.1453		-0.0558	0.4531	
**459**	37290	N089A_GL0114097	COG1653	ABC-type glycerol-3-phosphate transport system, periplasmic component	Carbohydrate transport and metabolism [G]	ABC transporters	Faecalibacterium prausnitzii	Species	Firmicutes	Clostridia	Clostridiales	Ruminococcaceae	Faecalibacterium	Faecalibacterium prausnitzii	-0.3075	0.0841		-0.0453	0.8109		-0.0675	0.6535	
**460**	37369	NLF005_GL0044735	COG2025	Electron transfer flavoprotein, alpha subunit	Energy production and conversion [C]	Nitrogen metabolism	Clostridiales	Order	Firmicutes	Clostridia	Clostridiales				-0.0367	0.8539		0.2138	0.3010		0.0162	0.9221	
**461**	37656	NLM017_GL0007726	COG0593	Chromosomal replication initiation ATPase DnaA	Replication, recombination and repair [L]	NAN	Clostridiales	Order	Firmicutes	Clostridia	Clostridiales				0.0888	0.3379		-0.0046	0.9620		-0.0756	0.3256	
**462**	37699	NLM023_GL0007606	COG1879	ABC-type sugar transport system, periplasmic component, contains N-terminal xre family HTH domain	Carbohydrate transport and metabolism [G]	ABC transporters	Clostridium	Genus	Firmicutes	Clostridia	Clostridiales	Clostridiaceae	Clostridium		-0.0199	0.9158		-0.5646	0.0023	**	-0.0473	0.7624	
**463**	37765	NLM027_GL0008865	COG1185	Polyribonucleotide nucleotidyltransferase (polynucleotide phosphorylase)	Translation, ribosomal structure and biogenesis [J]	Purine metabolism	Clostridiales	Order	Firmicutes	Clostridia	Clostridiales				0.0282	0.6776		-0.0572	0.4181		-0.0728	0.1922	
**464**	37777	NLM027_GL0043238	COG0013	Alanyl-tRNA synthetase	Translation, ribosomal structure and biogenesis [J]	Aminoacyl-tRNA biosynthesis	Clostridiales	Order	Firmicutes	Clostridia	Clostridiales				-0.0485	0.5306		-0.0828	0.3033		-0.0631	0.3240	
**465**	37821	NLM029_GL0062158	COG0152	Phosphoribosylaminoimidazole-succinocarboxamide synthase	Nucleotide transport and metabolism [F]	Purine metabolism	Ruminococcaceae	Species	Firmicutes	Clostridia	Clostridiales	Ruminococcaceae	Ruminococcus	Ruminococcus callidus	0.1341	0.0738		0.0267	0.7388		0.0118	0.8524	
**466**	37874	NLM032_GL0007335	COG0543	NAD(P)H-flavin reductase	Coenzyme transport and metabolism [H]	Amino sugar and nucleotide sugar metabolism	Clostridiales	Order	Firmicutes	Clostridia	Clostridiales				-0.1141	0.2506		0.0366	0.7266		0.0633	0.4450	
**467**	37877	NLM032_GL0020088			NAN	NAN	Paenibacillus sp. IHB B 3415	Species	Firmicutes	Bacilli	Bacillales	Paenibacillaceae	Paenibacillus	Paenibacillus sp. IHB B 3415	-0.0187	0.8776		0.0608	0.6318		-0.0346	0.7315	

**468**	37919	NOF005_GL0030287	COG0087	Ribosomal protein L3	Translation, ribosomal structure and biogenesis [J]	Ribosome	Clostridiales	Order	Firmicutes	Clostridia	Clostridiales				0.2014	0.1130		0.0378	0.7794		-0.0461	0.6670	
**469**	38121	NOM009_GL0086915	COG1250	3-hydroxyacyl-CoA dehydrogenase	Lipid transport and metabolism [I]	Fatty acid degradation	Clostridiales	Order	Firmicutes	Clostridia	Clostridiales				-0.1360	0.5238		0.2453	0.2686		-0.1031	0.5607	
**470**	38126	NOM009_GL0108718	COG1653	ABC-type glycerol-3-phosphate transport system, periplasmic component	Carbohydrate transport and metabolism [G]	ABC transporters	Collinsella aerofaciens	Species	Actinobacteria	Coriobacteriia	Coriobacteriales	Coriobacteriaceae	Collinsella	Collinsella aerofaciens	-0.2449	0.0928		0.1122	0.4678		-0.1420	0.2450	
**471**	38160	NOM014_GL0023636	COG0334	Glutamate dehydrogenase/leucine dehydrogenase	Amino acid transport and metabolism [E]	Alanine, aspartate and glutamate metabolism	Clostridium	Genus	Firmicutes	Clostridia	Clostridiales	Clostridiaceae	Clostridium		-0.0063	0.9726		0.0118	0.9510		0.1362	0.3704	
**472**	38293	NOM028_GL0008171	COG5016	Pyruvate/oxaloacetate carboxyltransferase	Energy production and conversion [C]	Citrate cycle (TCA cycle)	Clostridiales	Order	Firmicutes	Clostridia	Clostridiales				0.0364	0.7467		-0.0530	0.6526		0.0173	0.8537	
**473**	38315	NOM029_GL0053956	COG0098	Ribosomal protein S5	Translation, ribosomal structure and biogenesis [J]	Ribosome	Clostridiales	Order	Firmicutes	Clostridia	Clostridiales				0.0288	0.8308		0.0251	0.8588		0.0017	0.9878	
**474**	38448	O2.CD1-0-PT_GL0064002	COG1151	Hydroxylamine reductase (hybrid-cluster protein)	Inorganic ion transport and metabolism [P]	Nitrotoluene degradation	Clostridiales	Order	Firmicutes	Clostridia	Clostridiales				-0.0473	0.6193		0.1562	0.1108		-0.1255	0.1070	
**475**	38463	O2.CD1-0-PT_GL0080248	COG0138	AICAR transformylase/IMP cyclohydrolase PurH	Nucleotide transport and metabolism [F]	Purine metabolism	Bifidobacterium	Genus	Actinobacteria	Actinobacteria	Bifidobacteriales	Bifidobacteriaceae	Bifidobacterium		-0.0039	0.9715		-0.0414	0.7185		-0.0530	0.5609	
**476**	38482	O2.CD1-0-PT_GL0091861	COG0021	Transketolase	Carbohydrate transport and metabolism [G]	Pentose phosphate pathway	Bifidobacterium	Genus	Actinobacteria	Actinobacteria	Bifidobacteriales	Bifidobacteriaceae	Bifidobacterium		0.0179	0.8830		-0.0716	0.5733		0.0668	0.5077	
**477**	38558	O2.CD2-0_GL0042351	COG1978	Predicted RNase H-related nuclease YkuK, DUF458 family	General function prediction only [R]	NAN	Dorea formicigenerans	Species	Firmicutes	Clostridia	Clostridiales	Lachnospiraceae	Dorea	Dorea formicigenerans	0.0549	0.6690		-0.1483	0.2655		-0.1845	0.0776	
**478**	38738	O2.CD2-0-PT_GL0075729	COG1653	ABC-type glycerol-3-phosphate transport system, periplasmic component	Carbohydrate transport and metabolism [G]	ABC transporters	Bifidobacterium	Genus	Actinobacteria	Actinobacteria	Bifidobacteriales	Bifidobacteriaceae	Bifidobacterium		0.2772	0.1900		-0.1856	0.4040		-0.1371	0.4381	
**479**	38881	O2.CD3-0-PT_GL0099997	COG3411	(2Fe-2S) ferredoxin	Energy production and conversion [C]	Oxidative phosphorylation	Clostridiales	Order	Firmicutes	Clostridia	Clostridiales				-0.0677	0.5593		0.0225	0.8532		-0.1278	0.1800	
**480**	39077	O2.UC11-1_GL0020482	COG2222	Fructoselysine-6-P-deglycase FrlB and related proteins with duplicated sugar isomerase (SIS) domain	Cell wall/membrane/envelope biogenesis [M]	Alanine, aspartate and glutamate metabolism	Collinsella	Genus	Actinobacteria	Coriobacteriia	Coriobacteriales	Coriobacteriaceae	Collinsella		-0.1800	0.1952		-0.0077	0.9584		-0.0930	0.4230	
**481**	39110	O2.UC11-1_GL0068784	COG1978	Predicted RNase H-related nuclease YkuK, DUF458 family	General function prediction only [R]	NAN	Dorea formicigenerans	Species	Firmicutes	Clostridia	Clostridiales	Lachnospiraceae	Dorea	Dorea formicigenerans	-0.1244	0.2899		-0.0468	0.7050		-0.0458	0.6404	
**482**	39123	O2.UC11-1_GL0109553			NAN	NAN	Dorea formicigenerans	Species	Firmicutes	Clostridia	Clostridiales	Lachnospiraceae	Dorea	Dorea formicigenerans	0.0898	0.3993		-0.1058	0.3411		-0.1606	0.0645	
**483**	39184	O2.UC11-2_GL0041094	COG3842	ABC-type Fe3+/spermidine/putrescine transport systems, ATPase components	Amino acid transport and metabolism [E]	ABC transporters	Firmicutes	Phylum	Firmicutes						0.0562	0.6468		-0.1554	0.2210		0.1521	0.1298	
**484**	39301	O2.UC12-1_GL0041048	COG0191	Fructose/tagatose bisphosphate aldolase	Carbohydrate transport and metabolism [G]	Glycolysis / Gluconeogenesis	Collinsella	Genus	Actinobacteria	Coriobacteriia	Coriobacteriales	Coriobacteriaceae	Collinsella		-0.2517	0.0797		-0.1443	0.3428		-0.0110	0.9277	
**485**	39540	O2.UC14-2_GL0091805	COG1653	ABC-type glycerol-3-phosphate transport system, periplasmic component	Carbohydrate transport and metabolism [G]	ABC transporters	Ruminococcus	Genus	Firmicutes	Clostridia	Clostridiales	Ruminococcaceae	Ruminococcus		0.0943	0.6141		-0.1904	0.3278		0.0954	0.5387	
**486**	39724	O2.UC15-1_GL0039389	COG0280	Phosphotransacetylase	Energy production and conversion [C]	Taurine and hypotaurine metabolism	Clostridiales	Order	Firmicutes	Clostridia	Clostridiales				0.0764	0.6060		-0.0306	0.8436		0.0290	0.8136	
**487**	39746	O2.UC15-1_GL0073124	COG0149	Triosephosphate isomerase	Carbohydrate transport and metabolism [G]	Glycolysis / Gluconeogenesis	Blautia	Genus	Firmicutes	Clostridia	Clostridiales	Lachnospiraceae	Blautia		0.0727	0.4071		-0.1995	0.0252	*	-0.0050	0.9457	
**488**	39751	O2.UC15-1_GL0080989	COG1063	Threonine dehydrogenase or related Zn-dependent dehydrogenase	General function prediction only [R]	Fructose and mannose metabolism	Clostridiales	Order	Firmicutes	Clostridia	Clostridiales				0.0172	0.8980		-0.0338	0.8091		0.0576	0.6032	
**489**	39842	O2.UC16-1_GL0052335	COG1879	ABC-type sugar transport system, periplasmic component, contains N-terminal xre family HTH domain	Carbohydrate transport and metabolism [G]	ABC transporters	Clostridiales	Order	Firmicutes	Clostridia	Clostridiales				-0.3714	0.0093	**	0.0532	0.7326		-0.0696	0.5734	
**490**	39882	O2.UC16-2_GL0091224			NAN	NAN	Firmicutes	Phylum	Firmicutes						-0.0947	0.5823		0.1886	0.2920		-0.0091	0.9494	
**491**	39918	O2.UC17-1_GL0077925	COG0539	Ribosomal protein S1	Translation, ribosomal structure and biogenesis [J]	Ribosome	Collinsella	Genus	Actinobacteria	Coriobacteriia	Coriobacteriales	Coriobacteriaceae	Collinsella		-0.0955	0.3444		-0.0598	0.5721		-0.0429	0.6099	
**492**	39921	O2.UC17-1_GL0084053	COG0078	Ornithine carbamoyltransferase	Amino acid transport and metabolism [E]	Arginine and proline metabolism	Clostridiales	Order	Firmicutes	Clostridia	Clostridiales				0.1100	0.2369		-0.0672	0.4918		-0.0837	0.2791	

**493**	39980	O2.UC17-2_GL0115696	COG1145	Ferredoxin	Energy production and conversion [C]	Alanine, aspartate and glutamate metabolism	Blautia	Genus	Firmicutes	Clostridia	Clostridiales	Lachnospiraceae	Blautia		-0.2267	0.1054		0.0287	0.8467		0.0362	0.7594	
**494**	40091	O2.UC18-1_GL0200164	COG1063	Threonine dehydrogenase or related Zn-dependent dehydrogenase	General function prediction only [R]	Fructose and mannose metabolism	Blautia obeum	Species	Firmicutes	Clostridia	Clostridiales	Lachnospiraceae	Blautia	Blautia obeum	-0.0805	0.4559		0.0099	0.9303		-0.1424	0.1072	
**495**	40101	O2.UC18-2_GL0031006	COG0517	CBS domain	Signal transduction mechanisms [T]	Purine metabolism	Ruminococcaceae	Species	Firmicutes	Clostridia	Clostridiales	Ruminococcaceae	Faecalibacterium	Faecalibacterium prausnitzii	0.0089	0.9199		0.0051	0.9564		0.0219	0.7667	
**496**	40126	O2.UC19-1_GL0002247	COG4154	L-fucose mutarotase/ribose pyranase, RbsD/FucU family	Carbohydrate transport and metabolism [G]	NAN	Clostridiales	Order	Firmicutes	Clostridia	Clostridiales				-0.0244	0.7649		-0.0285	0.7377		0.0006	0.9932	
**497**	40312	O2.UC2-0_GL0002702	COG0052	Ribosomal protein S2	Translation, ribosomal structure and biogenesis [J]	Ribosome	Ruminococcaceae	Family	Firmicutes	Clostridia	Clostridiales	Ruminococcaceae			0.0428	0.6836		0.0257	0.8146		-0.0620	0.4760	
**498**	40455	O2.UC20-2_GL0062335	COG3203	Outer membrane protein (porin)	Cell wall/membrane/envelope biogenesis [M]	NAN	Dialister invisus	Species	Firmicutes	Negativicutes	Veillonellales	Veillonellaceae	Dialister	Dialister invisus	0.1349	0.1759		-0.1658	0.1096		0.0221	0.7916	
**499**	40481	O2.UC2-1_GL0059641	COG0206	Cell division GTPase FtsZ	Cell cycle control, cell division, chromosome partitioning [D]	Cell cycle - Caulobacter	Clostridiales	Order	Firmicutes	Clostridia	Clostridiales				-0.0863	0.4526		0.0548	0.6483		-0.1292	0.1717	
**500**	40575	O2.UC21-2_GL0076310	COG3958	Transketolase, C-terminal subunit	Carbohydrate transport and metabolism [G]	Pentose phosphate pathway	Clostridiales	Order	Firmicutes	Clostridia	Clostridiales				-0.0598	0.5535		0.0123	0.9074		-0.0948	0.2550	
**501**	40650	O2.UC22-1_GL0018255	COG0539	Ribosomal protein S1	Translation, ribosomal structure and biogenesis [J]	Ribosome	Blautia	Genus	Firmicutes	Clostridia	Clostridiales	Lachnospiraceae	Blautia		0.0824	0.4481		-0.0838	0.4605		0.0178	0.8437	
**502**	40802	O2.UC23-1_GL0058516	COG0149	Triosephosphate isomerase	Carbohydrate transport and metabolism [G]	Glycolysis / Gluconeogenesis	Clostridiales	Order	Firmicutes	Clostridia	Clostridiales				0.0190	0.8355		0.0543	0.5698		-0.1247	0.0953	
**503**	40893	O2.UC24-1_GL0171065			NAN	NAN	Ruminococcus bromii	Species	Firmicutes	Clostridia	Clostridiales	Ruminococcaceae	Ruminococcus	Ruminococcus bromii	0.0587	0.6410		0.0205	0.8766		0.1341	0.1952	
**504**	40993	O2.UC26-1_GL0038440	COG3209	Uncharacterized conserved protein RhaS, contains 28 RHS repeats	General function prediction only [R]	NAN	Blautia obeum	Species	Firmicutes	Clostridia	Clostridiales	Lachnospiraceae	Blautia	Blautia obeum	0.2068	0.3668		-0.2678	0.2619		0.2395	0.2059	
**505**	41146	O2.UC28-2_GL0061806	COG0234	Co-chaperonin GroES (HSP10)	Posttranslational modification, protein turnover, chaperones [O]	NAN	Collinsella	Genus	Actinobacteria	Coriobacteriia	Coriobacteriales	Coriobacteriaceae	Collinsella		-0.0564	0.6582		-0.0384	0.7735		-0.0758	0.4732	
**506**	41174	O2.UC28-2_GL0191778	COG1653	ABC-type glycerol-3-phosphate transport system, periplasmic component	Carbohydrate transport and metabolism [G]	ABC transporters	Clostridiales	Order	Firmicutes	Clostridia	Clostridiales				-0.1260	0.4623		0.0284	0.8745		-0.0511	0.7202	
**507**	41223	O2.UC29-2_GL0118672	COG0593	Chromosomal replication initiation ATPase DnaA	Replication, recombination and repair [L]	NAN	Subdoligranulum variabile	Species	Firmicutes	Clostridia	Clostridiales	Ruminococcaceae	Subdoligranulum	Subdoligranulum variabile	0.0993	0.1658		-0.0476	0.5291		0.0210	0.7265	
**508**	41264	O2.UC30-0_GL0087476	COG0544	FKBP-type peptidyl-prolyl cis-trans isomerase (trigger factor)	Posttranslational modification, protein turnover, chaperones [O]	NAN	Clostridiales	Order	Firmicutes	Clostridia	Clostridiales				0.0243	0.7879		-0.0376	0.6905		-0.0298	0.6917	
**509**	41277	O2.UC30-1_GL0014932	COG3842	ABC-type Fe3+/spermidine/putrescine transport systems, ATPase components	Amino acid transport and metabolism [E]	ABC transporters	Clostridium	Genus	Firmicutes	Clostridia	Clostridiales	Clostridiaceae	Clostridium		0.1844	0.0529		0.0333	0.7436		0.0883	0.2719	
**510**	41419	O2.UC31-1_GL0115264	COG0085	DNA-directed RNA polymerase, beta subunit/140 kD subunit	Transcription [K]	Purine metabolism	Clostridiales	Order	Firmicutes	Clostridia	Clostridiales				-0.0483	0.7142		0.0440	0.7496		-0.0534	0.6253	
**511**	41485	O2.UC32-0_GL0051137	COG2160	L-arabinose isomerase	Carbohydrate transport and metabolism [G]	Pentose and glucuronate interconversions	Bifidobacterium	Genus	Actinobacteria	Actinobacteria	Bifidobacteriales	Bifidobacteriaceae	Bifidobacterium		0.0819	0.7069		0.1150	0.6128		0.0359	0.8427	
**512**	41502	O2.UC32-1_GL0001634	COG1102	Cytidylate kinase	Nucleotide transport and metabolism [F]	NAN	Lachnospiraceae	Family	Firmicutes	Clostridia	Clostridiales	Lachnospiraceae			-0.0719	0.4280		0.1221	0.1945		-0.0001	0.9993	
**513**	41546	O2.UC32-1_GL0088850	COG0459	Chaperonin GroEL (HSP60 family)	Posttranslational modification, protein turnover, chaperones [O]	RNA degradation	Clostridiales	Order	Firmicutes	Clostridia	Clostridiales				-0.1100	0.2860		-0.1839	0.0839		0.0192	0.8236	
**514**	41682	O2.UC35-1_GL0126652	COG0088	Ribosomal protein L4	Translation, ribosomal structure and biogenesis [J]	Ribosome	Faecalibacterium prausnitzii	Species	Firmicutes	Clostridia	Clostridiales	Ruminococcaceae	Faecalibacterium	Faecalibacterium prausnitzii	-0.1452	0.3334		0.0766	0.6268		0.0302	0.8095	
**515**	41706	O2.UC35-2_GL0138962	COG0366	Glycosidase	Carbohydrate transport and metabolism [G]	Galactose metabolism	Clostridiales	Order	Firmicutes	Clostridia	Clostridiales				0.1859	0.0950		-0.0336	0.7762		0.0713	0.4468	
**516**	41889	O2.UC37-1_GL0081003	COG0021	Transketolase	Carbohydrate transport and metabolism [G]	Pentose phosphate pathway	Bifidobacterium bifidum	Species	Actinobacteria	Actinobacteria	Bifidobacteriales	Bifidobacteriaceae	Bifidobacterium	Bifidobacterium bifidum	-0.0504	0.8078		0.1239	0.5663		0.1161	0.4986	

**517**	41900	O2.UC37-1_GL0100475	COG0282	Acetate kinase	Energy production and conversion [C]	Taurine and hypotaurine metabolism	Faecalibacterium prausnitzii	Species	Firmicutes	Clostridia	Clostridiales	Ruminococcaceae	Faecalibacterium	Faecalibacterium prausnitzii	-0.0468	0.7147		0.0124	0.9261		-0.0050	0.9627	
**518**	42060	O2.UC40-1_GL0122610	COG1882	Pyruvate-formate lyase	Energy production and conversion [C]	Pyruvate metabolism	Clostridiales bacterium KLE1615	Species	Firmicutes	Clostridia	Clostridiales			Clostridiales bacterium KLE1615	-0.0430	0.7528		0.3487	0.0108	*	-0.1419	0.2060	
**519**	42140	O2.UC4-1_GL0039891	COG3835	Sugar diacid utilization regulator	Signal transduction mechanisms [T]	NAN	Ruminococcus bromii	Species	Firmicutes	Clostridia	Clostridiales	Ruminococcaceae	Ruminococcus	Ruminococcus bromii	-0.0588	0.6055		0.1116	0.3459		-0.1335	0.1526	
**520**	42159	O2.UC4-1_GL0166577	COG0334	Glutamate dehydrogenase/leucine dehydrogenase	Amino acid transport and metabolism [E]	Alanine, aspartate and glutamate metabolism	Clostridiales	Order	Firmicutes	Clostridia	Clostridiales				0.1003	0.5055		-0.2155	0.1665		-0.0453	0.7175	
**521**	42296	O2.UC4-2_GL0000649	COG0234	Co-chaperonin GroES (HSP10)	Posttranslational modification, protein turnover, chaperones [O]	NAN	Clostridiales	Order	Firmicutes	Clostridia	Clostridiales				0.1552	0.1226		0.0343	0.7473		0.0267	0.7528	
**522**	42469	O2.UC44-1_GL0166896	COG3957	Phosphoketolase	Carbohydrate transport and metabolism [G]	Pentose phosphate pathway	Bifidobacterium	Genus	Actinobacteria	Actinobacteria	Bifidobacteriales	Bifidobacteriaceae	Bifidobacterium		-0.2495	0.1425		-0.0305	0.8656		0.0135	0.9251	
**523**	42472	O2.UC44-1_GL0171886	COG0049	Ribosomal protein S7	Translation, ribosomal structure and biogenesis [J]	Ribosome	Subdoligranulum variabile	Species	Firmicutes	Clostridia	Clostridiales	Ruminococcaceae	Subdoligranulum	Subdoligranulum variabile	-0.0181	0.7632		-0.0612	0.3253		-0.0897	0.0655	
**524**	42601	O2.UC46-1_GL0109045	COG1145	Ferredoxin	Energy production and conversion [C]	Glycolysis / Gluconeogenesis	Faecalibacterium prausnitzii	Species	Firmicutes	Clostridia	Clostridiales	Ruminococcaceae	Faecalibacterium	Faecalibacterium prausnitzii	-0.1054	0.5026		0.1741	0.2870		0.0745	0.5683	
**525**	42632	O2.UC46-2_GL0146529	COG1879	ABC-type sugar transport system, periplasmic component, contains N-terminal xre family HTH domain	Carbohydrate transport and metabolism [G]	ABC transporters	Clostridiales	Order	Firmicutes	Clostridia	Clostridiales				0.0840	0.3402		-0.0189	0.8382		-0.0904	0.2142	
**526**	42701	O2.UC47-2_GL0002078	COG1250	3-hydroxyacyl-CoA dehydrogenase	Lipid transport and metabolism [I]	Fatty acid degradation	Clostridiales	Order	Firmicutes	Clostridia	Clostridiales				-0.0016	0.9901		0.0314	0.8105		-0.0254	0.8073	
**527**	42713	O2.UC47-2_GL0030333	COG1082	Sugar phosphate isomerase/epimerase	Carbohydrate transport and metabolism [G]	Inositol phosphate metabolism	Firmicutes	Phylum	Firmicutes						0.3678	0.0282	*	-0.2054	0.2513		0.1220	0.3933	
**528**	42738	O2.UC48-0_GL0000051	COG1653	ABC-type glycerol-3-phosphate transport system, periplasmic component	Carbohydrate transport and metabolism [G]	ABC transporters	Ruminococcus bromii	Species	Firmicutes	Clostridia	Clostridiales	Ruminococcaceae	Ruminococcus	Ruminococcus bromii	-0.5143	0.0766		-0.3245	0.2911		-0.4214	0.0807	
**529**	42754	O2.UC48-0_GL0026589	COG1960	Acyl-CoA dehydrogenase related to the alkylation response protein AidB	Lipid transport and metabolism [I]	Fatty acid degradation	Firmicutes	Phylum	Firmicutes						0.0454	0.6796		0.0639	0.5768		-0.1120	0.2152	
**530**	42755	O2.UC48-0_GL0026590	COG1024	Enoyl-CoA hydratase/carnithine racemase(	Lipid transport and metabolism [I]	Fatty acid degradation	Firmicutes	Phylum	Firmicutes						0.0966	0.2721		-0.0077	0.9334		-0.0318	0.6656	
**531**	42801	O2.UC48-0_GL0101313	COG4822	Cobalamin biosynthesis protein CbiK, Co2+ chelatase	Coenzyme transport and metabolism [H]	Porphyrin and chlorophyll metabolism	Clostridiales	Order	Firmicutes	Clostridia	Clostridiales				0.0686	0.4106		-0.0486	0.5776		-0.0836	0.2247	
**532**	42802	O2.UC48-0_GL0102815	COG1960	Acyl-CoA dehydrogenase related to the alkylation response protein AidB	Lipid transport and metabolism [I]	Fatty acid degradation	Firmicutes	Phylum	Firmicutes						-0.0021	0.9853		0.1006	0.3936		-0.1068	0.2529	
**533**	42803	O2.UC48-0_GL0103416	COG4992	Acetylornithine/succinyldiaminopimelate/putrescine aminotransferase	Amino acid transport and metabolism [E]	Lysine biosynthesis	Firmicutes	Phylum	Firmicutes						-0.0023	0.9764		-0.0157	0.8490		-0.0827	0.2022	
**534**	42804	O2.UC48-0_GL0107610	COG3842	ABC-type Fe3+/spermidine/putrescine transport systems, ATPase components	Amino acid transport and metabolism [E]	ABC transporters	Lachnospiraceae	Family	Firmicutes	Clostridia	Clostridiales	Lachnospiraceae			0.0884	0.5286		-0.0325	0.8248		-0.1027	0.3770	
**535**	42831	O2.UC48-0_GL0126253	COG1454	Alcohol dehydrogenase, class IV	Energy production and conversion [C]	Glycolysis / Gluconeogenesis	Firmicutes	Phylum	Firmicutes						0.0766	0.3075		0.0063	0.9368		-0.0634	0.3091	
**536**	42835	O2.UC48-0_GL0135226	COG1834	N-Dimethylarginine dimethylaminohydrolase	Amino acid transport and metabolism [E]	Glycine, serine and threonine metabolism	Bacteria	Superkingdom							0.0793	0.6387		-0.2104	0.2295		-0.0066	0.9624	
**537**	42974	O2.UC48-1_GL0053868	COG0059	Ketol-acid reductoisomerase	Coenzyme transport and metabolism [H]	Valine, leucine and isoleucine biosynthesis	Clostridiales	Order	Firmicutes	Clostridia	Clostridiales				-0.0285	0.8029		0.0224	0.8512		0.0336	0.7231	
**538**	42995	O2.UC48-1_GL0148775	COG0334	Glutamate dehydrogenase/leucine dehydrogenase	Amino acid transport and metabolism [E]	Alanine, aspartate and glutamate metabolism	Ruminococcus bromii	Species	Firmicutes	Clostridia	Clostridiales	Ruminococcaceae	Ruminococcus	Ruminococcus bromii	-0.5155	0.0025	**	-0.0528	0.7795		0.0135	0.9284	
**539**	43085	O2.UC5-0_GL0002879	COG2407	L-fucose isomerase or related protein	Carbohydrate transport and metabolism [G]	Fructose and mannose metabolism	Clostridiales	Order	Firmicutes	Clostridia	Clostridiales				-0.0999	0.5493		0.0908	0.6028		0.0800	0.5638	
**540**	43145	O2.UC50-0_GL0264730	COG0091	Ribosomal protein L22	Translation, ribosomal structure and biogenesis [J]	Ribosome	Subdoligranulum variabile	Species	Firmicutes	Clostridia	Clostridiales	Ruminococcaceae	Subdoligranulum	Subdoligranulum variabile	-0.0950	0.3003		-0.0933	0.3304		0.0438	0.5670	
**541**	43200	O2.UC5-1_GL0032422	COG0737	2',3'-cyclic-nucleotide 2'-phosphodiesterase/5'- or 3'-nucleotidase, 5'-nucleotidase family	Defense mechanisms [V]	Purine metabolism	Faecalibacterium prausnitzii	Species	Firmicutes	Clostridia	Clostridiales	Ruminococcaceae	Faecalibacterium	Faecalibacterium prausnitzii	0.0149	0.8941		-0.0874	0.4530		0.0774	0.4026	

**542**	43365	O2.UC55-0_GL0124819	COG0811	Biopolymer transport protein ExbB/TolQ	Intracellular trafficking, secretion, and vesicular transport [U]	Microbial metabolism in diverse environments	Parabacteroides	Genus	Bacteroidetes	Bacteroidia	Bacteroidales	Porphyromonadaceae	Parabacteroides		0.3608	0.0133	*	-0.2983	0.0536		0.0445	0.7233	
**543**	43393	O2.UC56-0_GL0134141	COG0366	Glycosidase	Carbohydrate transport and metabolism [G]	Galactose metabolism	Clostridiales	Order	Firmicutes	Clostridia	Clostridiales				0.0362	0.5935		-0.0969	0.1667		-0.0203	0.7185	
**544**	43394	O2.UC56-0_GL0138638	COG1087	UDP-glucose 4-epimerase	Cell wall/membrane/envelope biogenesis [M]	Galactose metabolism	Clostridiales	Order	Firmicutes	Clostridia	Clostridiales				0.0058	0.9344		-0.0914	0.2125		0.0341	0.5620	
**545**	43456	O2.UC57-2_GL0049799	COG0126	3-phosphoglycerate kinase	Carbohydrate transport and metabolism [G]	Glycolysis / Gluconeogenesis	Lachnospiraceae	Family	Firmicutes	Clostridia	Clostridiales	Lachnospiraceae			0.0270	0.8756		0.2606	0.1417		0.2603	0.0625	
**546**	43480	O2.UC58-0_GL0094376	COG1762	Phosphotransferase system mannitol/fructose-specific IIA domain (Ntr-type)	Signal transduction mechanisms [T]	Fructose and mannose metabolism	Ruminococcus bromii	Species	Firmicutes	Clostridia	Clostridiales	Ruminococcaceae	Ruminococcus	Ruminococcus bromii	-0.2293	0.1551		0.2094	0.2153		-0.1154	0.3927	
**547**	43491	O2.UC58-2_GL0017801	COG0360	Ribosomal protein S6	Translation, ribosomal structure and biogenesis [J]	Ribosome	Bifidobacterium	Genus	Actinobacteria	Actinobacteria	Bifidobacteriales	Bifidobacteriaceae	Bifidobacterium		0.1102	0.4829		-0.0465	0.7776		0.0649	0.6192	
**548**	43499	O2.UC58-2_GL0079351	COG1454	Alcohol dehydrogenase, class IV	Energy production and conversion [C]	Glycolysis / Gluconeogenesis	Roseburia faecis	Species	Firmicutes	Clostridia	Clostridiales	Lachnospiraceae	Roseburia	Roseburia faecis	-0.2491	0.0434	*	0.0924	0.4834		-0.1095	0.2939	
**549**	43649	T2D-53A_GL0140451	COG0091	Ribosomal protein L22	Translation, ribosomal structure and biogenesis [J]	Ribosome	Clostridiales	Order	Firmicutes	Clostridia	Clostridiales				-0.0927	0.4453		0.0628	0.6214		-0.1071	0.2864	
**550**	43725	O2.UC7-1_GL0015069	COG1653	ABC-type glycerol-3-phosphate transport system, periplasmic component	Carbohydrate transport and metabolism [G]	ABC transporters	Clostridiales	Order	Firmicutes	Clostridia	Clostridiales				0.1595	0.3753		0.0275	0.8842		0.1696	0.2546	
**551**	43732	O2.UC7-1_GL0034210	COG0047	Phosphoribosylformylglycinamidine (FGAM) synthase, glutamine amidotransferase domain	Nucleotide transport and metabolism [F]	Purine metabolism	Clostridiales	Order	Firmicutes	Clostridia	Clostridiales				0.0027	0.9859		0.1800	0.2516		-0.0292	0.8167	
**552**	43832	O2.UC8-0_GL0153477	COG0057	Glyceraldehyde-3-phosphate dehydrogenase/erythrose-4-phosphate dehydrogenase	Carbohydrate transport and metabolism [G]	Glycolysis / Gluconeogenesis	Bifidobacterium adolescentis	Species	Actinobacteria	Actinobacteria	Bifidobacteriales	Bifidobacteriaceae	Bifidobacterium	Bifidobacterium adolescentis	-0.1350	0.4008		0.0874	0.6036		0.0282	0.8331	
**553**	43926	O2.UC9-0_GL0086178	COG1592	Rubrerythrin	Energy production and conversion [C]	NAN	Lachnospiraceae	Family	Firmicutes	Clostridia	Clostridiales	Lachnospiraceae			-0.1299	0.1550		-0.0789	0.4122		0.0005	0.9952	
**554**	44201	SZEY-09A_GL0049065	COG0737	2',3'-cyclic-nucleotide 2'-phosphodiesterase/5'- or 3'-nucleotidase, 5'-nucleotidase family	Defense mechanisms [V]	Purine metabolism	Faecalibacterium prausnitzii	Species	Firmicutes	Clostridia	Clostridiales	Ruminococcaceae	Faecalibacterium	Faecalibacterium prausnitzii	0.3561	0.0223	*	-0.3354	0.0407	*	-0.1131	0.3960	
**555**	44405	SZEY-35A_GL0096220	COG0334	Glutamate dehydrogenase/leucine dehydrogenase	Amino acid transport and metabolism [E]	Alanine, aspartate and glutamate metabolism	Fusicatenibacter saccharivorans	Species	Firmicutes	Clostridia	Clostridiales	Lachnospiraceae	Fusicatenibacter	Fusicatenibacter saccharivorans	-0.0874	0.5379		0.0434	0.7704		0.2107	0.0684	
**556**	44443	SZEY-38A_GL0037167	COG0137	Argininosuccinate synthase	Amino acid transport and metabolism [E]	Alanine, aspartate and glutamate metabolism	Ruminococcus bromii	Species	Firmicutes	Clostridia	Clostridiales	Ruminococcaceae	Ruminococcus	Ruminococcus bromii	-0.0629	0.7137		0.3841	0.0271	*	-0.2217	0.1138	
**557**	44507	NLF006_GL0012500	COG1454	Alcohol dehydrogenase, class IV	Energy production and conversion [C]	NAN	Ruminococcus bromii	Species	Firmicutes	Clostridia	Clostridiales	Ruminococcaceae	Ruminococcus	Ruminococcus bromii	-0.0944	0.5110		0.0166	0.9123		-0.2502	0.0311	*
**558**	44567	SZEY-59A_GL0043576	COG1653	ABC-type glycerol-3-phosphate transport system, periplasmic component	Carbohydrate transport and metabolism [G]	NAN	Ruminococcus bicirculans	Species	Firmicutes	Clostridia	Clostridiales	Ruminococcaceae	Ruminococcus	Ruminococcus bicirculans	-0.1265	0.4921		0.1054	0.5842		0.1636	0.2823	
**559**	44603	SZEY-62A_GL0070012	COG0136	Aspartate-semialdehyde dehydrogenase	Amino acid transport and metabolism [E]	Glycine, serine and threonine metabolism	Ruminococcus bromii	Species	Firmicutes	Clostridia	Clostridiales	Ruminococcaceae	Ruminococcus	Ruminococcus bromii	-0.1015	0.5137		-0.0103	0.9495		-0.1615	0.2071	
**560**	44660	SZEY-69A_GL0070022	COG5492	Uncharacterized conserved protein YjdB, contains Ig-like domain	General function prediction only [R]	NAN	Symbiobacterium thermophilum	Species	Firmicutes	Clostridia	Clostridiales	Symbiobacteriaceae	Symbiobacterium	Symbiobacterium thermophilum	0.2371	0.1406		0.1802	0.2869		0.1345	0.3177	
**561**	44662	SZEY-69A_GL0078143	COG2222	Fructoselysine-6-P-deglycase FrlB and related proteins with duplicated sugar isomerase (SIS) domain	Cell wall/membrane/envelope biogenesis [M]	Alanine, aspartate and glutamate metabolism	Clostridiales	Order	Firmicutes	Clostridia	Clostridiales				-0.0009	0.9946		-0.1031	0.4277		-0.0129	0.9009	
**562**	44707	SZEY-78A_GL0090404	COG0563	Adenylate kinase or related kinase	Nucleotide transport and metabolism [F]	Purine metabolism	Clostridiales	Order	Firmicutes	Clostridia	Clostridiales				-0.0059	0.9388		-0.0928	0.2430		-0.0874	0.1647	
**563**	44853	T2D-105A_GL0107136	COG1629	Outer membrane receptor proteins, mostly Fe transport	Inorganic ion transport and metabolism [P]	NAN	Bacteroides	Genus	Bacteroidetes	Bacteroidia	Bacteroidales	Bacteroidaceae	Bacteroides		0.3993	0.0048	**	-0.0296	0.8488		-0.0297	0.8101	
**564**	45072	T2D-114A_GL0097515	COG0760	Parvulin-like peptidyl-prolyl isomerase	Posttranslational modification, protein turnover, chaperones [O]	NAN	Ruminococcus bromii	Species	Firmicutes	Clostridia	Clostridiales	Ruminococcaceae	Ruminococcus	Ruminococcus bromii	-0.1729	0.1741		0.0239	0.8587		-0.0832	0.4344	

**565**	45165	T2D-133A_GL0021831	COG1653	ABC-type glycerol-3-phosphate transport system, periplasmic component	Carbohydrate transport and metabolism [G]	NAN	Bifidobacterium	Genus	Actinobacteria	Actinobacteria	Bifidobacteriales	Bifidobacteriaceae	Bifidobacterium		0.0603	0.7146		-0.2584	0.1282		-0.0264	0.8473	
**566**	45166	T2D-122A_GL0083846	COG0176	Transaldolase	Carbohydrate transport and metabolism [G]	Pentose phosphate pathway	Bifidobacterium bifidum	Species	Actinobacteria	Actinobacteria	Bifidobacteriales	Bifidobacteriaceae	Bifidobacterium	Bifidobacterium bifidum	0.0283	0.9012		0.1044	0.6609		0.1915	0.3087	
**567**	45245	T2D-133A_GL0070710	COG4771	Outer membrane receptor for ferrienterochelin and colicins	Inorganic ion transport and metabolism [P]	NAN	Bacteroides uniformis	Species	Bacteroidetes	Bacteroidia	Bacteroidales	Bacteroidaceae	Bacteroides	Bacteroides uniformis	0.1618	0.4311		-0.4077	0.0526		0.0947	0.5796	
**568**	45270	T2D-135A_GL0000682	COG4771	Outer membrane receptor for ferrienterochelin and colicins	Inorganic ion transport and metabolism [P]	NAN	Bacteroides massiliensis	Species	Bacteroidetes	Bacteroidia	Bacteroidales	Bacteroidaceae	Bacteroides	Bacteroides massiliensis	0.2491	0.1527		-0.0697	0.7051		-0.0282	0.8475	
**569**	45429	T2D-142A_GL0060662	COG1185	Polyribonucleotide nucleotidyltransferase (polynucleotide phosphorylase)	Translation, ribosomal structure and biogenesis [J]	Purine metabolism	Ruminococcus bromii	Species	Firmicutes	Clostridia	Clostridiales	Ruminococcaceae	Ruminococcus	Ruminococcus bromii	-0.1409	0.3602		0.1479	0.3577		-0.1725	0.1744	
**570**	45433	T2D-142A_GL0079303	COG0457	Tetratricopeptide (TPR) repeat	General function prediction only [R]	NAN	Bacteroides eggerthii	Species	Bacteroidetes	Bacteroidia	Bacteroidales	Bacteroidaceae	Bacteroides	Bacteroides eggerthii	0.1944	0.3600		-0.4191	0.0544		0.2641	0.1306	
**571**	45788	T2D-203A_GL0049645	COG0459	Chaperonin GroEL (HSP60 family)	Posttranslational modification, protein turnover, chaperones [O]	RNA degradation	Lachnospiraceae	Family	Firmicutes	Clostridia	Clostridiales	Lachnospiraceae			-0.1335	0.4728		0.0068	0.9722		-0.0631	0.6836	
**572**	45892	T2D-26A_GL0024418	COG0078	Ornithine carbamoyltransferase	Amino acid transport and metabolism [E]	Arginine and proline metabolism	Collinsella aerofaciens	Species	Actinobacteria	Coriobacteriia	Coriobacteriales	Coriobacteriaceae	Collinsella	Collinsella aerofaciens	-0.0727	0.5266		-0.1091	0.3619		-0.1440	0.1260	
**573**	45940	T2D-29A_GL0106551	COG1960	Acyl-CoA dehydrogenase related to the alkylation response protein AidB	Lipid transport and metabolism [I]	Fatty acid degradation	Clostridiales	Order	Firmicutes	Clostridia	Clostridiales				-0.0702	0.6667		0.0266	0.8758		-0.0526	0.6978	
**574**	46091	T2D-35A_GL0059333	COG0783	DNA-binding ferritin-like protein (oxidative damage protectant)	Defense mechanisms [V]	NAN	Bifidobacterium breve	Species	Actinobacteria	Actinobacteria	Bifidobacteriales	Bifidobacteriaceae	Bifidobacterium	Bifidobacterium breve	-0.0257	0.8643		-0.1327	0.3956		0.1416	0.2524	
**575**	46103	T2D-41A_GL0070446	COG1264	Phosphotransferase system IIB components	Carbohydrate transport and metabolism [G]	Glycolysis / Gluconeogenesis	[Eubacterium] rectale	Species	Firmicutes	Clostridia	Clostridiales	Lachnospiraceae		[Eubacterium] rectale	-0.0385	0.6938		0.1033	0.3085		0.1075	0.1801	
**576**	46132	T2D-45A_GL0043917	COG0149	Triosephosphate isomerase	Carbohydrate transport and metabolism [G]	Glycolysis / Gluconeogenesis	Subdoligranulum variabile	Species	Firmicutes	Clostridia	Clostridiales	Ruminococcaceae	Subdoligranulum	Subdoligranulum variabile	0.0587	0.5864		-0.0170	0.8801		0.0570	0.5247	
**577**	46705	T2D-83A_GL0082037	COG3250	Beta-galactosidase/beta-glucuronidase	Carbohydrate transport and metabolism [G]	Pentose and glucuronate interconversions	Bacteroides vulgatus	Species	Bacteroidetes	Bacteroidia	Bacteroidales	Bacteroidaceae	Bacteroides	Bacteroides vulgatus	0.2967	0.0287	*	-0.0515	0.7244		0.0632	0.5858	
**578**	46846	V1.CD1-0-PT_GL0076248	COG3957	Phosphoketolase	Carbohydrate transport and metabolism [G]	Pentose phosphate pathway	Bifidobacterium bifidum	Species	Actinobacteria	Actinobacteria	Bifidobacteriales	Bifidobacteriaceae	Bifidobacterium	Bifidobacterium bifidum	-0.0294	0.8990		0.0859	0.7226		0.3192	0.0911	
**579**	46855	V1.CD1-0-PT_GL0103563	COG0148	Enolase	Carbohydrate transport and metabolism [G]	Glycolysis / Gluconeogenesis	Bifidobacterium bifidum	Species	Actinobacteria	Actinobacteria	Bifidobacteriales	Bifidobacteriaceae	Bifidobacterium	Bifidobacterium bifidum	0.0404	0.8067		-0.0478	0.7816		0.1521	0.2637	
**580**	46995	V1.CD1-3-PN_GL0123771	COG0261	Ribosomal protein L21(	Translation, ribosomal structure and biogenesis [J]	Ribosome	Bifidobacterium	Genus	Actinobacteria	Actinobacteria	Bifidobacteriales	Bifidobacteriaceae	Bifidobacterium		-0.0098	0.9322		-0.0606	0.6159		0.0632	0.5095	
**581**	47015	V1.CD14-0_GL0091287	COG2182	Maltose-binding periplasmic protein MalE	Carbohydrate transport and metabolism [G]	ABC transporters	Clostridiales	Order	Firmicutes	Clostridia	Clostridiales				-0.2237	0.3517		0.0266	0.9161		-0.0308	0.8778	
**582**	47016	V1.CD15-0_GL0019876	COG0747	ABC-type transport system, periplasmic component	Amino acid transport and metabolism [E]	ABC transporters	Clostridiales	Order	Firmicutes	Clostridia	Clostridiales				0.1305	0.3879		0.0078	0.9610		-0.0225	0.8581	
**583**	47191	V1.CD18-0_GL0025638	COG0149	Triosephosphate isomerase	Carbohydrate transport and metabolism [G]	Glycolysis / Gluconeogenesis	Clostridiales	Order	Firmicutes	Clostridia	Clostridiales				-0.1159	0.2528		0.0809	0.4472		-0.0162	0.8486	
**584**	47296	V1.CD20-0_GL0142249	COG1410	Methionine synthase I, cobalamin-binding domain	Amino acid transport and metabolism [E]	Cysteine and methionine metabolism	Clostridiales	Order	Firmicutes	Clostridia	Clostridiales				0.0817	0.4978		-0.0679	0.5900		-0.0490	0.6250	
**585**	47388	V1.CD2-0-PN_GL0068657	COG1063	Threonine dehydrogenase or related Zn-dependent dehydrogenase	General function prediction only [R]	Fructose and mannose metabolism	Bacteria	Species	Firmicutes	Clostridia	Clostridiales	Lachnospiraceae	Blautia	Blautia sp. KLE 1732	-0.0769	0.4496		0.0709	0.5049		-0.0605	0.4740	
**586**	47578	V1.CD24-0_GL0085096	COG3716	Phosphotransferase system, mannose/fructose/N-acetylgalactosamine-specific component IID	Carbohydrate transport and metabolism [G]	Fructose and mannose metabolism	Faecalibacterium prausnitzii	Species	Firmicutes	Clostridia	Clostridiales	Ruminococcaceae	Faecalibacterium	Faecalibacterium prausnitzii	0.0612	0.6573		-0.2224	0.1172		0.0097	0.9326	
**587**	47730	V1.CD27-0_GL0136511	COG0222	Ribosomal protein L7/L12	Translation, ribosomal structure and biogenesis [J]	Ribosome	Collinsella	Genus	Actinobacteria	Coriobacteriia	Coriobacteriales	Coriobacteriaceae	Collinsella		-0.1371	0.2916		-0.0821	0.5472		-0.0373	0.7312	
**588**	47747	V1.CD28-0_GL0110070	COG0448	ADP-glucose pyrophosphorylase	Carbohydrate transport and metabolism [G]	Starch and sucrose metabolism	Clostridiales	Order	Firmicutes	Clostridia	Clostridiales				0.1055	0.3622		-0.1291	0.2849		0.0726	0.4506	
**589**	47769	V1.CD29-0_GL0134727	COG1879	ABC-type sugar transport system, periplasmic component, contains N-terminal xre family HTH domain	Carbohydrate transport and metabolism [G]	ABC transporters	Dorea longicatena	Species	Firmicutes	Clostridia	Clostridiales	Lachnospiraceae	Dorea	Dorea longicatena	0.1294	0.5403		-0.4148	0.0550		0.0097	0.9560	

**590**	47793	V1.CD30-0_GL0013170	COG0102	Ribosomal protein L13	Translation, ribosomal structure and biogenesis [J]	Ribosome	Clostridiales	Order	Firmicutes	Clostridia	Clostridiales				0.0068	0.9446		-0.0602	0.5532		0.0596	0.4598	
**591**	47807	V1.CD30-0_GL0138968	COG1070	Sugar (pentulose or hexulose) kinase	Carbohydrate transport and metabolism [G]	Pentose phosphate pathway	Anaerotruncus colihominis	Species	Firmicutes	Clostridia	Clostridiales	Ruminococcaceae	Anaerotruncus	Anaerotruncus colihominis	0.1002	0.3851		-0.0859	0.4766		0.0584	0.5427	
**592**	48122	V1.CD34-0_GL0042016	COG0088	Ribosomal protein L4	Translation, ribosomal structure and biogenesis [J]	Ribosome	Clostridiales	Order	Firmicutes	Clostridia	Clostridiales				0.1136	0.4301		0.2508	0.0905		-0.0942	0.4307	
**593**	48145	V1.CD34-0_GL0135713	COG0332	3-oxoacyl-[acyl-carrier-protein] synthase III	Lipid transport and metabolism [I]	NAN	Dorea formicigenerans	Species	Firmicutes	Clostridia	Clostridiales	Lachnospiraceae	Dorea	Dorea formicigenerans	0.0481	0.6621		-0.0161	0.8889		-0.0965	0.2877	
**594**	48199	V1.CD36-0_GL0032080	COG0166	Glucose-6-phosphate isomerase	Carbohydrate transport and metabolism [G]	Glycolysis / Gluconeogenesis	Bifidobacterium	Genus	Actinobacteria	Actinobacteria	Bifidobacteriales	Bifidobacteriaceae	Bifidobacterium		-0.3143	0.0851		0.0769	0.6922		0.0000	0.9998	
**595**	48230	V1.CD36-0_GL0147854	COG0696	Phosphoglycerate mutase (BPG-independent, AlkP superfamily)	Carbohydrate transport and metabolism [G]	Glycolysis / Gluconeogenesis	Collinsella	Genus	Actinobacteria	Coriobacteriia	Coriobacteriales	Coriobacteriaceae	Collinsella		-0.2336	0.1026		-0.0466	0.7591		-0.0387	0.7487	
**596**	48325	V1.CD40-0_GL0053388	COG1653	ABC-type glycerol-3-phosphate transport system, periplasmic component	Carbohydrate transport and metabolism [G]	NAN	Bifidobacterium	Species	Actinobacteria	Actinobacteria	Bifidobacteriales	Bifidobacteriaceae	Bifidobacterium	Bifidobacterium bifidum	0.1227	0.2617		0.1767	0.1190		0.1214	0.1794	
**597**	48362	V1.CD40-0_GL0150812	COG0039	Malate/lactate dehydrogenase	Energy production and conversion [C]	Glycolysis / Gluconeogenesis	Clostridiales	Order	Firmicutes	Clostridia	Clostridiales				-0.0404	0.6698		0.0398	0.6874		0.0843	0.2797	
**598**	48400	V1.CD41-0_GL0030063	COG3173	Predicted kinase, aminoglycoside phosphotransferase (APT) family	General function prediction only [R]	NAN	Collinsella	Genus	Actinobacteria	Coriobacteriia	Coriobacteriales	Coriobacteriaceae	Collinsella		-0.1802	0.1155		-0.0717	0.5542		-0.0768	0.4241	
**599**	48444	V1.CD42-0_GL0129731	COG2222	Fructoselysine-6-P-deglycase FrlB and related proteins with duplicated sugar isomerase (SIS) domain	Cell wall/membrane/envelope biogenesis [M]	Alanine, aspartate and glutamate metabolism	Firmicutes	Phylum	Firmicutes						0.2918	0.1217		0.0357	0.8582		0.0384	0.8088	
**600**	48536	V1.CD46-0_GL0008945	COG1454	Alcohol dehydrogenase, class IV	Energy production and conversion [C]	Glycolysis / Gluconeogenesis	Dorea longicatena	Species	Firmicutes	Clostridia	Clostridiales	Lachnospiraceae	Dorea	Dorea longicatena	0.0668	0.5495		-0.1044	0.3688		-0.0378	0.6831	
**601**	48654	V1.CD50-0_GL0068523	COG0138	AICAR transformylase/IMP cyclohydrolase PurH	Nucleotide transport and metabolism [F]	Purine metabolism	Ruminococcus bromii	Species	Firmicutes	Clostridia	Clostridiales	Ruminococcaceae	Ruminococcus	Ruminococcus bromii	-0.1736	0.0656		0.1836	0.0622		-0.0420	0.5992	
**602**	48661	V1.CD50-0_GL0122012	COG2070	NAD(P)H-dependent flavin oxidoreductase YrpB, nitropropane dioxygenase family	General function prediction only [R]	Purine metabolism	Clostridiales	Order	Firmicutes	Clostridia	Clostridiales				-0.0502	0.6384		0.0725	0.5157		-0.0196	0.8255	
**603**	48670	V1.CD50-0_GL0170135	COG0104	Adenylosuccinate synthase	Nucleotide transport and metabolism [F]	Purine metabolism	Ruminococcaceae	Family	Firmicutes	Clostridia	Clostridiales	Ruminococcaceae			0.0549	0.4213		0.0467	0.5131		0.0746	0.1854	
**604**	48871	V1.CD54-0_GL0122997	COG0334	Glutamate dehydrogenase/leucine dehydrogenase	Amino acid transport and metabolism [E]	Alanine, aspartate and glutamate metabolism	Firmicutes	Phylum	Firmicutes						-0.0329	0.7599		-0.0685	0.5421		-0.1702	0.0513	
**605**	49073	V1.CD6-4_GL0089549	COG1653	ABC-type glycerol-3-phosphate transport system, periplasmic component	Carbohydrate transport and metabolism [G]	NAN	Bacteria	Superkingdom							-0.0181	0.8620		0.0775	0.4750		-0.0620	0.4723	
**606**	49280	V1.CD8-0-PN_GL0136266	COG1454	Alcohol dehydrogenase, class IV	Energy production and conversion [C]	Glycolysis / Gluconeogenesis	Clostridiales	Order	Firmicutes	Clostridia	Clostridiales				0.2270	0.1929		-0.2642	0.1458		-0.0230	0.8749	
**607**	49334	V1.FI02_GL0017922	COG0542	ATP-dependent Clp protease ATP-binding subunit ClpA	Posttranslational modification, protein turnover, chaperones [O]	NAN	Blautia obeum	Species	Firmicutes	Clostridia	Clostridiales	Lachnospiraceae	Blautia	Blautia obeum	-0.0601	0.7000		-0.3183	0.0451	*	-0.1516	0.2378	
**608**	49363	V1.FI02_GL0189243	COG0443	Molecular chaperone DnaK (HSP70)	Posttranslational modification, protein turnover, chaperones [O]	RNA degradation	Bifidobacterium	Genus	Actinobacteria	Actinobacteria	Bifidobacteriales	Bifidobacteriaceae	Bifidobacterium		-0.1263	0.3290		0.0648	0.6335		-0.0304	0.7786	
**609**	49391	V1.FI04_GL0033515	COG1653	ABC-type glycerol-3-phosphate transport system, periplasmic component	Carbohydrate transport and metabolism [G]	NAN	Subdoligranulum variabile	Species	Firmicutes	Clostridia	Clostridiales	Ruminococcaceae	Subdoligranulum	Subdoligranulum variabile	0.2323	0.1824		-0.0050	0.9782		0.0231	0.8744	
**610**	49537	V1.FI05_GL0114531	COG1653	ABC-type glycerol-3-phosphate transport system, periplasmic component	Carbohydrate transport and metabolism [G]	ABC transporters	Bifidobacteriaceae	Species	Actinobacteria	Actinobacteria	Bifidobacteriales	Bifidobacteriaceae	Scardovia	Scardovia wiggsiae	-0.0524	0.6988		-0.1152	0.4135		-0.0377	0.7373	
**611**	49584	V1.FI06_GL0060106	COG0183	Acetyl-CoA acetyltransferase	Lipid transport and metabolism [I]	Fatty acid degradation	Lachnospiraceae	Family	Firmicutes	Clostridia	Clostridiales	Lachnospiraceae			-0.0677	0.6186		0.0798	0.5741		0.0398	0.7248	
**612**	49618	V1.FI06_GL0203708	COG1653	ABC-type glycerol-3-phosphate transport system, periplasmic component	Carbohydrate transport and metabolism [G]	ABC transporters	Clostridiales	Order	Firmicutes	Clostridia	Clostridiales				-0.2372	0.0766		0.1226	0.3883		-0.1116	0.3221	

**613**	50319	V1.FI17_GL0209634	COG0057	Glyceraldehyde-3-phosphate dehydrogenase/erythrose-4-phosphate dehydrogenase	Carbohydrate transport and metabolism [G]	Glycolysis / Gluconeogenesis	Porphyromonas endodontalis	Species	Bacteroidetes	Bacteroidia	Bacteroidales	Porphyromonadaceae	Porphyromonas	Porphyromonas endodontalis	0.1943	0.1768		-0.0741	0.6252		0.0321	0.7905	
**614**	50520	V1.FI22_GL0003131	COG1653	ABC-type glycerol-3-phosphate transport system, periplasmic component	Carbohydrate transport and metabolism [G]	ABC transporters	Clostridiales	Order	Firmicutes	Clostridia	Clostridiales				-0.0754	0.6073		-0.0475	0.7567		-0.1579	0.1905	
**615**	50549	V1.FI22_GL0137796	COG0096	Ribosomal protein S8	Translation, ribosomal structure and biogenesis [J]	Ribosome	Clostridiales	Order	Firmicutes	Clostridia	Clostridiales				0.1551	0.1831		-0.0970	0.4289		0.0605	0.5353	
**616**	50615	V1.FI25_GL0042905	COG2407	L-fucose isomerase or related protein	Carbohydrate transport and metabolism [G]	Fructose and mannose metabolism	Clostridiales	Order	Firmicutes	Clostridia	Clostridiales				0.0665	0.6127		-0.0424	0.7575		-0.0151	0.8899	
**617**	50690	V1.FI26_GL0267199	COG0737	2',3'-cyclic-nucleotide 2'-phosphodiesterase/5'- or 3'-nucleotidase, 5'-nucleotidase family	Defense mechanisms [V]	NAN	Flavobacterium gilvum	Species	Bacteroidetes	Flavobacteriia	Flavobacteriales	Flavobacteriaceae	Flavobacterium	Flavobacterium gilvum	0.2217	0.1241		-0.0116	0.9393		-0.0959	0.4282	
**618**	50707	V1.FI27_GL0072285	COG5426	Uncharacterized membrane protein	Function unknown [S]	NAN	Bifidobacterium longum	Species	Actinobacteria	Actinobacteria	Bifidobacteriales	Bifidobacteriaceae	Bifidobacterium	Bifidobacterium longum	0.0108	0.9397		0.0218	0.8837		0.0143	0.9041	
**619**	50727	V1.FI28_GL0009969	COG1454	Alcohol dehydrogenase, class IV	Energy production and conversion [C]	Glycolysis / Gluconeogenesis	Bacteria	Superkingdom							-0.0853	0.4554		-0.0294	0.8058		-0.0137	0.8854	
**620**	50866	V1.FI31_GL0134522	COG0334	Glutamate dehydrogenase/leucine dehydrogenase	Amino acid transport and metabolism [E]	Alanine, aspartate and glutamate metabolism	Oscillibacter	Genus	Firmicutes	Clostridia	Clostridiales	Oscillospiraceae	Oscillibacter		0.0176	0.9045		-0.0438	0.7742		-0.0917	0.4487	
**621**	51063	V1.FI36_GL0024061	COG0191	Fructose/tagatose bisphosphate aldolase	Carbohydrate transport and metabolism [G]	Glycolysis / Gluconeogenesis	Dorea	Genus	Firmicutes	Clostridia	Clostridiales	Lachnospiraceae	Dorea		-0.0779	0.6248		-0.0108	0.9482		-0.1470	0.2628	
**622**	51159	V1.FI37_GL0030438	COG1825	Ribosomal protein L25 (general stress protein Ctc)	Translation, ribosomal structure and biogenesis [J]	Ribosome	Bifidobacterium	Genus	Actinobacteria	Actinobacteria	Bifidobacteriales	Bifidobacteriaceae	Bifidobacterium		-0.0385	0.7650		-0.0471	0.7261		0.0304	0.7761	
**623**	51276	V1.UC11-0_GL0085451	COG0244	Ribosomal protein L10	Translation, ribosomal structure and biogenesis [J]	Ribosome	Bifidobacterium	Genus	Actinobacteria	Actinobacteria	Bifidobacteriales	Bifidobacteriaceae	Bifidobacterium		-0.0926	0.5628		0.0633	0.7052		0.0325	0.8074	
**624**	51478	V1.UC13-3_GL0060573	COG0360	Ribosomal protein S6	Translation, ribosomal structure and biogenesis [J]	Ribosome	Bifidobacterium	Genus	Actinobacteria	Actinobacteria	Bifidobacteriales	Bifidobacteriaceae	Bifidobacterium		-0.0918	0.4861		-0.0617	0.6547		0.0085	0.9384	
**625**	51690	V1.UC17-2_GL0123387	COG1145	Ferredoxin	Energy production and conversion [C]	Glycolysis / Gluconeogenesis	Collinsella aerofaciens	Species	Actinobacteria	Coriobacteriia	Coriobacteriales	Coriobacteriaceae	Collinsella	Collinsella aerofaciens	-0.2053	0.2613		-0.0601	0.7547		-0.2043	0.1767	
**626**	51788	V1.UC22-1_GL0166855	COG4577	Carboxysome shell and ethanolamine utilization microcompartment protein CcmL/EutN	Secondary metabolites biosynthesis, transport and catabolism [Q]	NAN	Clostridiales	Order	Firmicutes	Clostridia	Clostridiales				-0.0912	0.2707		-0.0128	0.8829		-0.0551	0.4246	
**627**	52389	V1.UC38-4_GL0131494	COG0088	Ribosomal protein L4	Translation, ribosomal structure and biogenesis [J]	Ribosome	Clostridiales	Order	Firmicutes	Clostridia	Clostridiales				0.0554	0.6179		-0.0350	0.7635		0.0647	0.4827	
**628**	52494	V1.UC40-0_GL0007441	COG0057	Glyceraldehyde-3-phosphate dehydrogenase/erythrose-4-phosphate dehydrogenase	Carbohydrate transport and metabolism [G]	Glycolysis / Gluconeogenesis	Anaerostipes caccae	Species	Firmicutes	Clostridia	Clostridiales	Lachnospiraceae	Anaerostipes	Anaerostipes caccae	-0.1997	0.1593		-0.0575	0.7016		-0.1859	0.1132	
**629**	52509	V1.UC40-0_GL0130575	COG3209	Uncharacterized conserved protein RhaS, contains 28 RHS repeats	General function prediction only [R]	NAN	Bacillus subtilis	Species	Firmicutes	Bacilli	Bacillales	Bacillaceae	Bacillus	Bacillus subtilis	0.1810	0.4047		-0.5498	0.0120	*	0.0993	0.5829	
**630**	52542	V1.UC40-1_GL0106359	COG0589	Nucleotide-binding universal stress protein, UspA family	Signal transduction mechanisms [T]	NAN	Bifidobacterium	Genus	Actinobacteria	Actinobacteria	Bifidobacteriales	Bifidobacteriaceae	Bifidobacterium		0.0393	0.7800		0.0328	0.8232		-0.0154	0.8949	
**631**	52557	V1.UC40-1_GL0166114	COG2759	Formyltetrahydrofolate synthetase	Nucleotide transport and metabolism [F]	One carbon pool by folate	Lachnospiraceae	Family	Firmicutes	Clostridia	Clostridiales	Lachnospiraceae			0.0543	0.6065		-0.0223	0.8394		0.1009	0.2452	
**632**	52581	V1.UC41-0_GL0045739	COG1653	ABC-type glycerol-3-phosphate transport system, periplasmic component	Carbohydrate transport and metabolism [G]	ABC transporters	Faecalibacterium prausnitzii	Species	Firmicutes	Clostridia	Clostridiales	Ruminococcaceae	Faecalibacterium	Faecalibacterium prausnitzii	-0.1263	0.5243		-0.4085	0.0434	*	0.0289	0.8608	
**633**	52610	V1.UC42-0_GL0049062	COG0329	Dihydrodipicolinate synthase/N-acetylneuraminate lyase	Cell wall/membrane/envelope biogenesis [M]	Lysine biosynthesis	Clostridiales	Order	Firmicutes	Clostridia	Clostridiales				0.0053	0.9518		-0.2069	0.0192	*	-0.0073	0.9201	
**634**	52781	V1.UC48-0_GL0060046			NAN	NAN	Alistipes putredinis	Species	Bacteroidetes	Bacteroidia	Bacteroidales	Rikenellaceae	Alistipes	Alistipes putredinis	-0.0867	0.4082		0.0302	0.7833		0.0812	0.3497	

**635**	52954	V1.UC51-4_GL0052281	COG1879	ABC-type sugar transport system, periplasmic component, contains N-terminal xre family HTH domain	Carbohydrate transport and metabolism [G]	ABC transporters	Clostridiales	Order	Firmicutes	Clostridia	Clostridiales				0.0538	0.7361		-0.2364	0.1512		0.0615	0.6425	
**636**	53140	V1.UC53-4_GL0037562	COG0087	Ribosomal protein L3	Translation, ribosomal structure and biogenesis [J]	Ribosome	Collinsella	Genus	Actinobacteria	Coriobacteriia	Coriobacteriales	Coriobacteriaceae	Collinsella		-0.1050	0.3090		-0.1468	0.1710		-0.0373	0.6647	
**637**	53397	V1.UC57-0_GL0145554	COG0264	Translation elongation factor EF-Ts	Translation, ribosomal structure and biogenesis [J]	NAN	Clostridiales	Order	Firmicutes	Clostridia	Clostridiales				0.1376	0.2237		-0.0775	0.5147		-0.0764	0.4184	
**638**	53544	V1.UC60-0_GL0052142	COG0047	Phosphoribosylformylglycinamidine (FGAM) synthase, glutamine amidotransferase domain	Nucleotide transport and metabolism [F]	Purine metabolism	Clostridiales	Order	Firmicutes	Clostridia	Clostridiales				-0.0223	0.8543		-0.0728	0.5649		0.0334	0.7398	

A-B-C PCA

PGs with loadings coefficient on PC1 of PCA > |0.09| on which MANOVA test was performed

N	Protein Group ID	Leading razor protein accession	COG accession	COG name	COG category	KEGG name	LCA	Rank	Phylum	Class	Order	Family	Genus	Species	Log_{10} (Omnivorous diet no/yes)	t-test p-value Omnivorous diet no/yes	Significance Omnivorous diet no/yes	Log_{10} (Obesity yes/no)	t-test p-value Obesity yes/no	Significance Obesity yes/no	Log_{10} (GI symptoms yes/no)	t-test p-value GI symptoms yes/no	Significance GI symptoms yes/no
1	112	206672.BL0597	COG0058	Glucan phosphorylase	Carbohydrate transport and metabolism [G]	Starch and sucrose metabolism	Bifidobacterium	Genus	Actinobacteria	Actinobacteria	Bifidobacteriales	Bifidobacteriaceae	Bifidobacterium		-0.0575	0.7747		-0.2208	0.2891		-0.1121	0.4999	
2	1139	537937.BLIG_01296	COG4166	ABC-type oligopeptide transport system, periplasmic component	Amino acid transport and metabolism [E]	ABC transporters	Bifidobacterium	Genus	Actinobacteria	Actinobacteria	Bifidobacteriales	Bifidobacteriaceae	Bifidobacterium		0.1571	0.4600		-0.4142	0.0571		0.0795	0.6530	
3	115	206672.BL0707	COG0126	3-phosphoglycerate kinase	Carbohydrate transport and metabolism [G]	Glycolysis / Gluconeogenesis	Bifidobacterium longum	Species	Actinobacteria	Actinobacteria	Bifidobacteriales	Bifidobacteriaceae	Bifidobacterium	Bifidobacterium longum	0.1038	0.6108		-0.1702	0.4229		0.0699	0.6798	
4	118	206672.BL0951	COG1882	Pyruvate-formate lyase	Energy production and conversion [C]	Pyruvate metabolism	Bifidobacterium	Genus	Actinobacteria	Actinobacteria	Bifidobacteriales	Bifidobacteriaceae	Bifidobacterium		0.2143	0.3087		-0.0426	0.8474		0.0942	0.5914	
5	122	206672.BL0988	COG0469	Pyruvate kinase	Carbohydrate transport and metabolism [G]	Glycolysis / Gluconeogenesis	Bifidobacterium	Genus	Actinobacteria	Actinobacteria	Bifidobacteriales	Bifidobacteriaceae	Bifidobacterium		0.0538	0.8094		-0.1031	0.6578		0.1505	0.4144	
6	1349	566552.BIFCAT_00987	COG0282	Acetate kinase	Energy production and conversion [C]	Taurine and hypotaurine metabolism	Bifidobacterium	Genus	Actinobacteria	Actinobacteria	Bifidobacteriales	Bifidobacteriaceae	Bifidobacterium		-0.1206	0.5095		-0.1416	0.4581		0.0747	0.6230	
7	1633	759350.BLJ_0360	COG0228	Ribosomal protein S16	Translation, ribosomal structure and biogenesis [J]	Ribosome	Bifidobacterium	Genus	Actinobacteria	Actinobacteria	Bifidobacteriales	Bifidobacteriaceae	Bifidobacterium		0.1637	0.3338		-0.0762	0.6681		0.0195	0.8905	
8	17817	MH0122_GL0107606	COG1653	ABC-type glycerol-3-phosphate transport system, periplasmic component	Carbohydrate transport and metabolism [G]	ABC transporters	Bifidobacterium	Genus	Actinobacteria	Actinobacteria	Bifidobacteriales	Bifidobacteriaceae	Bifidobacterium		-0.1377	0.5627		0.0028	0.9910		0.1154	0.5590	
9	18868	MH0131_GL0154213	COG0544	FKBP-type peptidyl-prolyl cis-trans isomerase (trigger factor)	Posttranslational modification, protein turnover, chaperones [O]	NAN	Bifidobacterium	Genus	Actinobacteria	Actinobacteria	Bifidobacteriales	Bifidobacteriaceae	Bifidobacterium		0.1061	0.5385		-0.1017	0.5728		0.0089	0.9504	
10	20757	MH0161_GL0142425	COG0148	Enolase	Carbohydrate transport and metabolism [G]	Glycolysis / Gluconeogenesis	Bifidobacterium adolescentis	Species	Actinobacteria	Actinobacteria	Bifidobacteriales	Bifidobacteriaceae	Bifidobacterium	Bifidobacterium adolescentis	-0.0237	0.8969		-0.0093	0.9610		0.1125	0.4566	
11	21833	MH0188_GL0007212	COG0039	Malate/lactate dehydrogenase	Energy production and conversion [C]	Glycolysis / Gluconeogenesis	Bifidobacterium	Genus	Actinobacteria	Actinobacteria	Bifidobacteriales	Bifidobacteriaceae	Bifidobacterium		0.1018	0.5619		-0.0264	0.8858		0.0321	0.8259	
12	21984	MH0188_GL0055295	COG1653	ABC-type glycerol-3-phosphate transport system, periplasmic component	Carbohydrate transport and metabolism [G]	ABC transporters	Bifidobacterium	Genus	Actinobacteria	Actinobacteria	Bifidobacteriales	Bifidobacteriaceae	Bifidobacterium		0.0227	0.9145		0.0294	0.8939		-0.0878	0.6158	
13	22090	MH0188_GL0095502	COG0174	Glutamine synthetase	Amino acid transport and metabolism [E]	Alanine, aspartate and glutamate metabolism	Bifidobacterium	Genus	Actinobacteria	Actinobacteria	Bifidobacteriales	Bifidobacteriaceae	Bifidobacterium		-0.0979	0.6411		-0.2836	0.1918		0.2336	0.1758	
14	22205	MH0188_GL0126744	COG0459	Chaperonin GroEL (HSP60 family)	Posttranslational modification, protein turnover, chaperones [O]	RNA degradation	Bifidobacterium	Genus	Actinobacteria	Actinobacteria	Bifidobacteriales	Bifidobacteriaceae	Bifidobacterium		0.0279	0.8858		-0.2632	0.1896		0.1609	0.3152	
15	22221	MH0188_GL0133256	COG1087	UDP-glucose 4-epimerase	Cell wall/membrane/envelope biogenesis [M]	Galactose metabolism	Bifidobacterium longum	Species	Actinobacteria	Actinobacteria	Bifidobacteriales	Bifidobacteriaceae	Bifidobacterium	Bifidobacterium longum	0.1067	0.5194		0.0190	0.9129		0.0453	0.7421	
16	22254	MH0188_GL0145450	COG1882	Pyruvate-formate lyase	Energy production and conversion [C]	Pyruvate metabolism	Bifidobacterium adolescentis	Species	Actinobacteria	Actinobacteria	Bifidobacteriales	Bifidobacteriaceae	Bifidobacterium	Bifidobacterium adolescentis	-0.1268	0.5390		0.0538	0.8034		-0.0129	0.9403	
17	22837	MH0193_GL0010680	COG0480	Translation elongation factor EF-G, a GTPase	Translation, ribosomal structure and biogenesis [J]	NAN	Bifidobacterium	Genus	Actinobacteria	Actinobacteria	Bifidobacteriales	Bifidobacteriaceae	Bifidobacterium		0.1694	0.5313		0.1724	0.5420		0.0908	0.6863	
18	23014	MH0193_GL0186495	COG1454	Alcohol dehydrogenase, class IV	Energy production and conversion [C]	Glycolysis / Gluconeogenesis	Bifidobacterium	Genus	Actinobacteria	Actinobacteria	Bifidobacteriales	Bifidobacteriaceae	Bifidobacterium		0.1580	0.4961		-0.0475	0.8449		0.2335	0.2224	
19	23687	MH0203_GL0013763	COG0057	Glyceraldehyde-3-phosphate dehydrogenase/erythrose-4-phosphate dehydrogenase	Carbohydrate transport and metabolism [G]	Glycolysis / Gluconeogenesis	Bifidobacterium	Genus	Actinobacteria	Actinobacteria	Bifidobacteriales	Bifidobacteriaceae	Bifidobacterium		0.0524	0.7398		-0.0044	0.9787		0.1397	0.2834	
20	23823	MH0203_GL0133062	COG0033	Phosphoglucomutase	Carbohydrate transport and metabolism [G]	Glycolysis / Gluconeogenesis	Bifidobacterium	Genus	Actinobacteria	Actinobacteria	Bifidobacteriales	Bifidobacteriaceae	Bifidobacterium		0.0550	0.7542		-0.2017	0.2683		-0.0417	0.7748	
21	2809	DLM018_GL0016638	COG0092	Ribosomal protein S3	Translation, ribosomal structure and biogenesis [J]	Ribosome	Bifidobacterium	Genus	Actinobacteria	Actinobacteria	Bifidobacteriales	Bifidobacteriaceae	Bifidobacterium		-0.0534	0.7565		0.0279	0.8769		-0.0205	0.8858	
22	28712	MH0293_GL0090794	COG1080	Phosphoenolpyruvate-protein kinase (PTS system EI component in bacteria)	Carbohydrate transport and metabolism [G]	Pyruvate metabolism	Clostridiales	Order	Firmicutes	Clostridia	Clostridiales				-0.2021	0.4560		-0.0091	0.9743		0.0270	0.9048	
23	30556	MH0341_GL0013504	COG0176	Transaldolase	Carbohydrate transport and metabolism [G]	Pentose phosphate pathway	Bifidobacterium	Genus	Actinobacteria	Actinobacteria	Bifidobacteriales	Bifidobacteriaceae	Bifidobacterium		-0.0663	0.7930		0.0397	0.8806		0.2581	0.2144	
24	30909	MH0348_GL0122167	COG0085	DNA-directed RNA polymerase, beta subunit/140 kD subunit	Transcription [K]	Purine metabolism	Bifidobacterium	Genus	Actinobacteria	Actinobacteria	Bifidobacteriales	Bifidobacteriaceae	Bifidobacterium		-0.0143	0.9355		-0.2779	0.1259		0.0573	0.6953	
25	340	391904.BLIJ_0145	COG0443	Molecular chaperone DnaK (HSP70)	Posttranslational modification, protein turnover, chaperones [O]	RNA degradation	Bifidobacterium	Genus	Actinobacteria	Actinobacteria	Bifidobacteriales	Bifidobacteriaceae	Bifidobacterium		-0.1734	0.4219		0.0682	0.7632		0.0568	0.7521	
26	38121	NOM009_GL0086915	COG1250	3-hydroxyacyl-CoA dehydrogenase	Lipid transport and metabolism [I]	Fatty acid degradation	Clostridiales	Order	Firmicutes	Clostridia	Clostridiales				-0.1360	0.5238		0.2453	0.2686		-0.1031	0.5607	

**27**	41485	O2.UC32-0_GL0051137	COG2160	L-arabinose isomerase	Carbohydrate transport and metabolism [G]	Pentose and glucuronate interconversions	Bifidobacterium	Genus	Actinobacteria	Actinobacteria	Bifidobacteriales	Bifidobacteriaceae	Bifidobacterium		0.0819	0.7069		0.1150	0.6128		0.0359	0.8427	
**28**	41889	O2.UC37-1_GL0081003	COG0021	Transketolase	Carbohydrate transport and metabolism [G]	Pentose phosphate pathway	Bifidobacterium bifidum	Species	Actinobacteria	Actinobacteria	Bifidobacteriales	Bifidobacteriaceae	Bifidobacterium	Bifidobacterium bifidum	-0.0504	0.8078		0.1239	0.5663		0.1161	0.4986	
**29**	43491	O2.UC58-2_GL0017801	COG0360	Ribosomal protein S6	Translation, ribosomal structure and biogenesis [J]	Ribosome	Bifidobacterium	Genus	Actinobacteria	Actinobacteria	Bifidobacteriales	Bifidobacteriaceae	Bifidobacterium		0.1102	0.4829		-0.0465	0.7776		0.0649	0.6192	
**30**	45166	T2D-122A_GL0083846	COG0176	Transaldolase	Carbohydrate transport and metabolism [G]	Pentose phosphate pathway	Bifidobacterium bifidum	Species	Actinobacteria	Actinobacteria	Bifidobacteriales	Bifidobacteriaceae	Bifidobacterium	Bifidobacterium bifidum	0.0283	0.9012		0.1044	0.6609		0.1915	0.3087	
**31**	46846	V1.CD1-0-PT_GL0076248	COG3957	Phosphoketolase	Carbohydrate transport and metabolism [G]	Pentose phosphate pathway	Bifidobacterium bifidum	Species	Actinobacteria	Actinobacteria	Bifidobacteriales	Bifidobacteriaceae	Bifidobacterium	Bifidobacterium bifidum	-0.0294	0.8990		0.0859	0.7226		0.3192	0.0911	
**32**	46855	V1.CD1-0-PT_GL0103563	COG0148	Enolase	Carbohydrate transport and metabolism [G]	Glycolysis / Gluconeogenesis	Bifidobacterium bifidum	Species	Actinobacteria	Actinobacteria	Bifidobacteriales	Bifidobacteriaceae	Bifidobacterium	Bifidobacterium bifidum	0.0404	0.8067		-0.0478	0.7816		0.1521	0.2637	
**33**	51276	V1.UC11-0_GL0085451	COG0244	Ribosomal protein L10	Translation, ribosomal structure and biogenesis [J]	Ribosome	Bifidobacterium	Genus	Actinobacteria	Actinobacteria	Bifidobacteriales	Bifidobacteriaceae	Bifidobacterium		-0.0926	0.5628		0.0633	0.7052		0.0325	0.8074	
**34**	939	515619.EUBREC_1472	COG1145	Ferredoxin	Energy production and conversion [C]	Glycolysis / Gluconeogenesis	Clostridiales	Order	Firmicutes	Clostridia	Clostridiales				-0.4512	0.1448		0.3390	0.2975		-0.2189	0.3985	

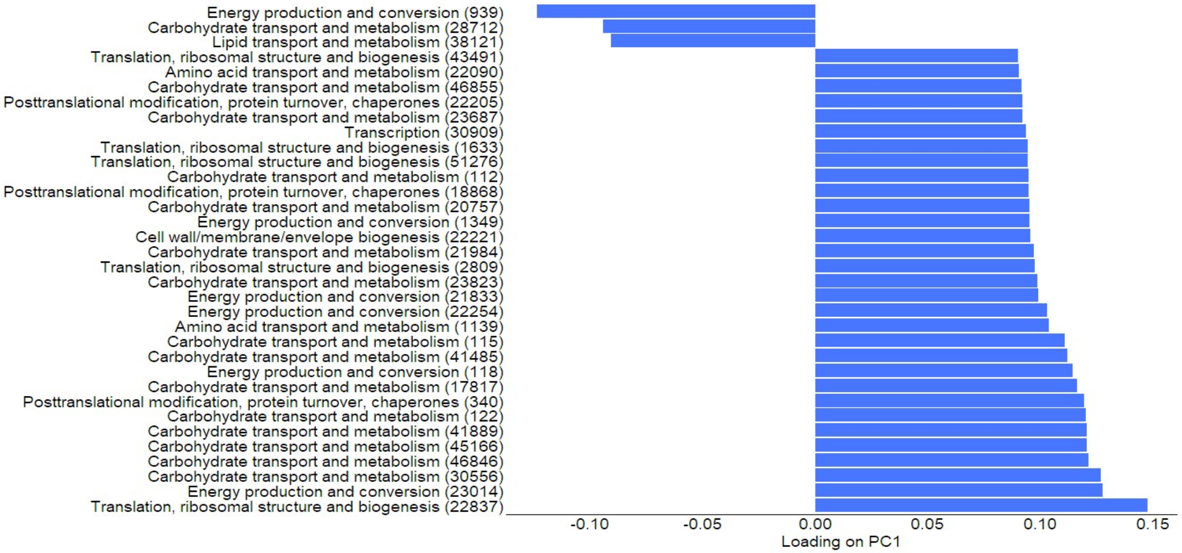

A (Diet) - PLS-DA
PGs with VIP > 2

N	Protein Group ID	Leading razor protein accession	COG accession	COG name	COG category	KEGG name	LCA	Rank	Phylum	Class	Order	Family	Genus	Species	Log_{10} Omnivorous diet no/yes	t-test p-value Omnivorous diet no/yes	Significance Omnivorous diet no/yes
1	28480	MH0288_GL0054326	COG4774	Outer membrane receptor for monomeric catechols	Inorganic ion transport and metabolism [P]	NAN	Bacteroides vulgatus	Species	Bacteroidetes	Bacteroidia	Bacteroidales	Bacteroidaceae	Bacteroides	Bacteroides vulgatus	0.6661	0.0003	***
2	32128	MH0371_GL0055922	COG1538	Outer membrane protein TolC	Cell wall/membrane/envelope biogenesis [M]	Bacterial secretion system	Bacteroides	Genus	Bacteroidetes	Bacteroidia	Bacteroidales	Bacteroidaceae	Bacteroides		0.4613	0.0045	**
3	36972	N047A_GL0051881			NAN	NAN	Bacteroides	Genus	Bacteroidetes	Bacteroidia	Bacteroidales	Bacteroidaceae	Bacteroides		0.4491	0.0198	*
4	5748	MH0003_GL0052996			NAN	NAN	Bacteroides	Genus	Bacteroidetes	Bacteroidia	Bacteroidales	Bacteroidaceae	Bacteroides		0.4337	0.0223	*
5	5650	MH0003_GL0042541	COG3637	Opacity protein and related surface antigens	Cell wall/membrane/envelope biogenesis [M]	NAN	Bacteroides	Genus	Bacteroidetes	Bacteroidia	Bacteroidales	Bacteroidaceae	Bacteroides		0.4331	0.0019	**
6	44853	T2D-105A_GL0107136	COG1629	Outer membrane receptor proteins, mostly Fe transport	Inorganic ion transport and metabolism [P]	NAN	Bacteroides	Genus	Bacteroidetes	Bacteroidia	Bacteroidales	Bacteroidaceae	Bacteroides		0.3993	0.0048	**
7	43365	O2.UC55-0_GL0124819	COG0811	Biopolymer transport protein ExbB/TolQ	Intracellular trafficking, secretion, and vesicular transport [U]	Microbial metabolism in diverse environments	Parabacteroides	Genus	Bacteroidetes	Bacteroidia	Bacteroidales	Porphyromonadaceae	Parabacteroides		0.3608	0.0133	*
8	36549	N003A_GL0049315	COG1862	Preprotein translocase subunit YajC	Intracellular trafficking, secretion, and vesicular transport [U]	Protein export	Bacteroides	Genus	Bacteroidetes	Bacteroidia	Bacteroidales	Bacteroidaceae	Bacteroides		0.3573	0.0004	***
9	44201	SZEY-09A_GL0049065	COG0737	2',3'-cyclic-nucleotide 2'-phosphodiesterase/5'- or 3'-nucleotidase, 5'-nucleotidase family	Defense mechanisms [V]	Purine metabolism	Faecalibacterium prausnitzii	Species	Firmicutes	Clostridia	Clostridiales	Ruminococcaceae	Faecalibacterium	Faecalibacterium prausnitzii	0.3561	0.0223	*
10	9235	MH0012_GL0199057	COG2885	Outer membrane protein OmpA and related peptidoglycan-associated (lipo)proteins	Cell wall/membrane/envelope biogenesis [M]	NAN	Bacteroidales	Order	Bacteroidetes	Bacteroidia	Bacteroidales				0.3213	0.0161	*
11	14751	MH0086_GL0098687	COG4166	ABC-type oligopeptide transport system, periplasmic component	Amino acid transport and metabolism [E]	ABC transporters	Clostridiales	Order	Firmicutes	Clostridia	Clostridiales				0.2809	0.0142	*
12	16977	MH0109_GL0036381	COG0149	Triosephosphate isomerase	Carbohydrate transport and metabolism [G]	Glycolysis / Gluconeogenesis	Firmicutes	Phylum	Firmicutes						0.2659	0.0141	*
13	26605	MH0251_GL0128679	COG1614	CO dehydrogenase/acetyl-CoA synthase beta subunit	Energy production and conversion [C]	Methane metabolism	Clostridiales	Order	Firmicutes	Clostridia	Clostridiales				0.1703	0.0211	*
14	14026	MH0073_GL0056444			NAN	Galactose metabolism	Ruminococcus bromii	Species	Firmicutes	Clostridia	Clostridiales	Ruminococcaceae	Ruminococcus	Ruminococcus bromii	-0.2497	0.0161	*
15	26571	MH0251_GL0069201	COG1653	ABC-type glycerol-3-phosphate transport system, periplasmic component	Carbohydrate transport and metabolism [G]	NAN	Clostridiales	Order	Firmicutes	Clostridia	Clostridiales				-0.2721	0.0094	**
16	5986	O2.UC48-0_GL0114931	COG0329	Dihydrodipicolinate synthase/N-acetylneuraminate lyase	Cell wall/membrane/envelope biogenesis [M]	Lysine biosynthesis	Ruminococcus bromii	Species	Firmicutes	Clostridia	Clostridiales	Ruminococcaceae	Ruminococcus	Ruminococcus bromii	-0.3028	0.0177	*
17	30641	MH0341_GL0114479	COG0277	FAD/FMN-containing dehydrogenase	Energy production and conversion [C]	Pyruvate metabolism	Firmicutes	Phylum	Firmicutes						-0.3440	0.0131	*
18	39842	O2.UC16-1_GL0052335	COG1879	ABC-type sugar transport system, periplasmic component, contains N-terminal xre family HTH domain	Carbohydrate transport and metabolism [G]	ABC transporters	Clostridiales	Order	Firmicutes	Clostridia	Clostridiales				-0.3714	0.0093	**
19	29843	MH0327_GL0049100	COG0443	Molecular chaperone DnaK (HSP70)	Posttranslational modification, protein turnover, chaperones [O]	RNA degradation	Collinsella aerofaciens	Species	Actinobacteria	Coriobacteriia	Coriobacteriales	Coriobacteriaceae	Collinsella	Collinsella aerofaciens	-0.4156	0.0063	**
20	42995	O2.UC48-1_GL0148775	COG0334	Glutamate dehydrogenase/leucine dehydrogenase	Amino acid transport and metabolism [E]	Alanine, aspartate and glutamate metabolism	Ruminococcus bromii	Species	Firmicutes	Clostridia	Clostridiales	Ruminococcaceae	Ruminococcus	Ruminococcus bromii	-0.5155	0.0025	**
21	17690	MH0122_GL0011708	COG0050	Translation elongation factor EF-Tu, a GTPase	Translation, ribosomal structure and biogenesis [J]	Plant-pathogen interaction	Subdoligranulum variabile	Species	Firmicutes	Clostridia	Clostridiales	Ruminococcaceae	Subdoligranulum	Subdoligranulum variabile	-0.5313	0.0169	*
22	22236	MH0188_GL0141556	COG0050	Translation elongation factor EF-Tu, a GTPase	Translation, ribosomal structure and biogenesis [J]	Plant-pathogen interaction	Collinsella	Genus	Actinobacteria	Coriobacteriia	Coriobacteriales	Coriobacteriaceae	Collinsella		-0.6933	0.0013	**

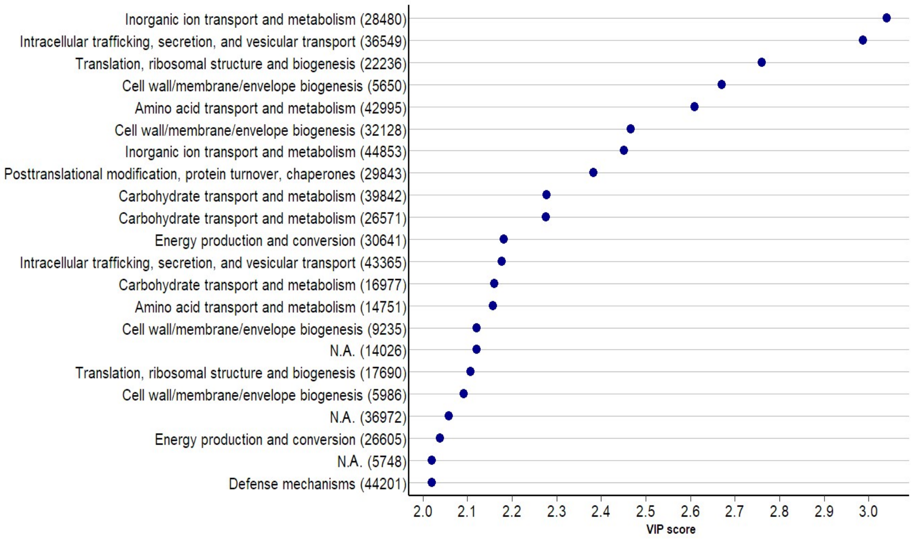

A (Diet) - differentially expressed PGs

N	Protein Group ID	Leading razor protein accession	COG accession	COG name	COG category	KEGG name	LCA	Rank	Phylum	Class	Order	Family	Genus	Species	Log_{10} (Omnivorous diet no/yes)	t-test p-value Omnivorous diet no/yes	Significance Omnivorous diet no/yes
1	606	435590.BVU_0563	COG4771	Outer membrane receptor for ferrienterochelin and colicins(16693)	Inorganic ion transport and metabolism [P]	NAN	Bacteroides	Genus	Bacteroidetes	Bacteroidia	Bacteroidales	Bacteroidaceae	Bacteroides		0.6697	0.0246	*
2	28480	MH0288_GL0054326	COG4774	Outer membrane receptor for monomeric catechols	Inorganic ion transport and metabolism [P]	NAN	Bacteroides vulgatus	Species	Bacteroidetes	Bacteroidia	Bacteroidales	Bacteroidaceae	Bacteroides	Bacteroides vulgatus	0.6661	0.0003	***
3	32128	MH0371_GL0055922	COG1538	Outer membrane protein TolC	Cell wall/membrane/envelope biogenesis [M]	Bacterial secretion system	Bacteroides	Genus	Bacteroidetes	Bacteroidia	Bacteroidales	Bacteroidaceae	Bacteroides		0.4613	0.0045	**
4	30175	MH0333_GL0121497	COG3209	Uncharacterized conserved protein RhaS, contains 28 RHS repeats	General function prediction only [R]	NAN	Blautia	Species	Firmicutes	Clostridia	Clostridiales	Lachnospiraceae	Blautia	Blautia wexlerae	0.4518	0.0286	*
5	36972	N047A_GL0051881			NAN	NAN	Bacteroides	Genus	Bacteroidetes	Bacteroidia	Bacteroidales	Bacteroidaceae	Bacteroides		0.4491	0.0198	*
6	5748	MH0003_GL0052996			NAN	NAN	Bacteroides	Genus	Bacteroidetes	Bacteroidia	Bacteroidales	Bacteroidaceae	Bacteroides		0.4337	0.0223	*
7	5650	MH0003_GL0042541	COG3637	Opacity protein and related surface antigens	Cell wall/membrane/envelope biogenesis [M]	NAN	Bacteroides	Genus	Bacteroidetes	Bacteroidia	Bacteroidales	Bacteroidaceae	Bacteroides		0.4331	0.0019	**
8	44853	T2D-105A_GL0107136	COG1629	Outer membrane receptor proteins, mostly Fe transport	Inorganic ion transport and metabolism [P]	NAN	Bacteroides	Genus	Bacteroidetes	Bacteroidia	Bacteroidales	Bacteroidaceae	Bacteroides		0.3993	0.0048	**
9	42713	O2.UC47-2_GL0030333	COG1082	Sugar phosphate isomerase/epimerase	Carbohydrate transport and metabolism [G]	Inositol phosphate metabolism	Firmicutes	Phylum	Firmicutes						0.3678	0.0282	*
10	43365	O2.UC55-0_GL0124819	COG0811	Biopolymer transport protein ExbB/TolQ	Intracellular trafficking, secretion, and vesicular transport [U]	Microbial metabolism in diverse environments	Parabacteroides	Genus	Bacteroidetes	Bacteroidia	Bacteroidales	Porphyromonadaceae	Parabacteroides		0.3608	0.0133	*
11	36549	N003A_GL0049315	COG1862	Preprotein translocase subunit YajC	Intracellular trafficking, secretion, and vesicular transport [U]	Protein export	Bacteroides	Genus	Bacteroidetes	Bacteroidia	Bacteroidales	Bacteroidaceae	Bacteroides		0.3573	0.0004	***
12	44201	SZEY-09A_GL0049065	COG0737	2',3'-cyclic-nucleotide 2'-phosphodiesterase/5'- or 3'-nucleotidase, 5'-nucleotidase family	Defense mechanisms [V]	Purine metabolism	Faecalibacterium prausnitzii	Species	Firmicutes	Clostridia	Clostridiales	Ruminococcaceae	Faecalibacterium	Faecalibacterium prausnitzii	0.3561	0.0223	*
13	9728	MH0014_GL0097483	COG1592	Rubrerythrin	Energy production and conversion [C]	NAN	Clostridiales	Order	Firmicutes	Clostridia	Clostridiales				0.3436	0.0368	*
14	923	483217.BACDOR_00668	COG3525	N-acetyl-beta-hexosaminidase(4602)	Carbohydrate transport and metabolism [G]	NAN	Bacteroides	Genus	Bacteroidetes	Bacteroidia	Bacteroidales	Bacteroidaceae	Bacteroides		0.3345	0.0409	*
15	9235	MH0012_GL0199057	COG2885	Outer membrane protein OmpA and related peptidoglycan-associated (lipo)proteins	Cell wall/membrane/envelope biogenesis [M]	NAN	Bacteroidales	Order	Bacteroidetes	Bacteroidia	Bacteroidales				0.3213	0.0161	*
16	46705	T2D-83A_GL0082037	COG3250	Beta-galactosidase/beta-glucuronidase	Carbohydrate transport and metabolism [G]	Pentose and glucuronate interconversions	Bacteroides vulgatus	Species	Bacteroidetes	Bacteroidia	Bacteroidales	Bacteroidaceae	Bacteroides	Bacteroides vulgatus	0.2967	0.0287	*
17	14751	MH0086_GL0098687	COG4166	ABC-type oligopeptide transport system, periplasmic component	Amino acid transport and metabolism [E]	ABC transporters	Clostridiales	Order	Firmicutes	Clostridia	Clostridiales				0.2809	0.0142	*
18	5035	MH0002_GL0050697	COG1726	Na+-transporting NADH:ubiquinone oxidoreductase, subunit NqrA	Energy production and conversion [C]	NAN	Bacteroides	Genus	Bacteroidetes	Bacteroidia	Bacteroidales	Bacteroidaceae	Bacteroides		0.2761	0.0476	*
19	16977	MH0109_GL0036381	COG0149	Triosephosphate isomerase	Carbohydrate transport and metabolism [G]	Glycolysis / Gluconeogenesis	Firmicutes	Phylum	Firmicutes						0.2659	0.0141	*
20	11241	MH0032_GL0024527	COG0090	Ribosomal protein L2(1921)	Translation, ribosomal structure and biogenesis [J]	Ribosome	Dialister succinatiphilus	Species	Firmicutes	Negativicutes	Veillonellales	Veillonellaceae	Dialister	Dialister succinatiphilus	0.2158	0.0489	*
21	31742	MH0367_GL0050319	COG1592	Rubrerythrin	Energy production and conversion [C]	NAN	Firmicutes	Phylum	Firmicutes						-0.2127	0.0332	*
22	35949	MH0447_GL0209994	COG0366	Glycosidase	Carbohydrate transport and metabolism [G]	Galactose metabolism	Ruminococcus bromii	Species	Firmicutes	Clostridia	Clostridiales	Ruminococcaceae	Ruminococcus	Ruminococcus bromii	-0.2482	0.0260	*
23	43499	O2.UC58-2_GL0079351	COG1454	Alcohol dehydrogenase, class IV	Energy production and conversion [C]	Glycolysis / Gluconeogenesis	Roseburia faecis	Species	Firmicutes	Clostridia	Clostridiales	Lachnospiraceae	Roseburia	Roseburia faecis	-0.2491	0.0434	*
24	14026	MH0073_GL0056444			NAN	Galactose metabolism	Ruminococcus bromii	Species	Firmicutes	Clostridia	Clostridiales	Ruminococcaceae	Ruminococcus	Ruminococcus bromii	-0.2497	0.0161	*
25	5429	MH0003_GL0009354	COG0297	Glycogen synthase	Carbohydrate transport and metabolism [G]	Galactose metabolism	Ruminococcus bromii	Species	Firmicutes	Clostridia	Clostridiales	Ruminococcaceae	Ruminococcus	Ruminococcus bromii	-0.2620	0.0468	*
26	5652	MH0003_GL0042613	COG1932	Phosphoserine aminotransferase	Coenzyme transport and metabolism [H]	Glycine, serine and threonine metabolism	Ruminococcus bromii	Species	Firmicutes	Clostridia	Clostridiales	Ruminococcaceae	Ruminococcus	Ruminococcus bromii	-0.2645	0.0424	*
27	32014	MH0370_GL0093205	COG2222	Fructoselysine-6-P-deglycase FrlB and related proteins with duplicated sugar isomerase (SIS) domain	Cell wall/membrane/envelope biogenesis [M]	Alanine, aspartate and glutamate metabolism	Collinsella aerofaciens	Species	Actinobacteria	Coriobacteriia	Coriobacteriales	Coriobacteriaceae	Collinsella	Collinsella aerofaciens	-0.2652	0.0412	*
28	26571	MH0251_GL0069201	COG1653	ABC-type glycerol-3-phosphate transport system, periplasmic component	Carbohydrate transport and metabolism [G]	NAN	Clostridiales	Order	Firmicutes	Clostridia	Clostridiales				-0.2721	0.0094	**
29	5986	O2.UC48-0_GL0114931	COG0329	Dihydrodipicolinate synthase/N-acetylneuraminate lyase	Cell wall/membrane/envelope biogenesis [M]	Lysine biosynthesis	Ruminococcus bromii	Species	Firmicutes	Clostridia	Clostridiales	Ruminococcaceae	Ruminococcus	Ruminococcus bromii	-0.3028	0.0177	*
30	26897	MH0259_GL0033209	COG1653	ABC-type glycerol-3-phosphate transport system, periplasmic component	Carbohydrate transport and metabolism [G]	ABC transporters	Bifidobacterium adolescentis	Species	Actinobacteria	Actinobacteria	Bifidobacteriales	Bifidobacteriaceae	Bifidobacterium	Bifidobacterium adolescentis	-0.3243	0.0479	*
31	5557	MH0003_GL0030541	COG0047	Phosphoribosylformylglycinamidine (FGAM) synthase, glutamine amidotransferase domain	Nucleotide transport and metabolism [F]	Purine metabolism	Ruminococcus bromii	Species	Firmicutes	Clostridia	Clostridiales	Ruminococcaceae	Ruminococcus	Ruminococcus bromii	-0.3405	0.0302	*
32	30641	MH0341_GL0114479	COG0277	FAD/FMN-containing dehydrogenase	Energy production and conversion [C]	Pyruvate metabolism	Firmicutes	Phylum	Firmicutes						-0.3440	0.0131	*
33	5553	MH0003_GL0030501	COG1454	Alcohol dehydrogenase, class IV	Energy production and conversion [C]	Glycolysis / Gluconeogenesis	Ruminococcus bromii	Species	Firmicutes	Clostridia	Clostridiales	Ruminococcaceae	Ruminococcus	Ruminococcus bromii	-0.3486	0.0462	*
34	27099	MH0262_GL0083048	COG3716	Phosphotransferase system, mannose/fructose/N-acetylgalactosamine-specific component IID	Carbohydrate transport and metabolism [G]	Fructose and mannose metabolism	Collinsella	Genus	Actinobacteria	Coriobacteriia	Coriobacteriales	Coriobacteriaceae	Collinsella		-0.3490	0.0264	*
35	13391	MH0062_GL0058668	COG4166	ABC-type oligopeptide transport system, periplasmic component	Amino acid transport and metabolism [E]	ABC transporters	Faecalibacterium prausnitzii	Species	Firmicutes	Clostridia	Clostridiales	Ruminococcaceae	Faecalibacterium	Faecalibacterium prausnitzii	-0.3532	0.0314	*
36	39842	O2.UC16-1_GL0052335	COG1879	ABC-type sugar transport system, periplasmic component, contains N-terminal xre family HTH domain	Carbohydrate transport and metabolism [G]	ABC transporters	Clostridiales	Order	Firmicutes	Clostridia	Clostridiales				-0.3714	0.0093	**
37	31655	MH0363_GL0154863	COG1653	ABC-type glycerol-3-phosphate transport system, periplasmic component	Carbohydrate transport and metabolism [G]	ABC transporters	Firmicutes	Phylum	Firmicutes						-0.3930	0.0253	*
38	29843	MH0327_GL0049100	COG0443	Molecular chaperone DnaK (HSP70)	Posttranslational modification, protein turnover, chaperones [O]	RNA degradation	Collinsella aerofaciens	Species	Actinobacteria	Coriobacteriia	Coriobacteriales	Coriobacteriaceae	Collinsella	Collinsella aerofaciens	-0.4156	0.0063	**
39	42995	O2.UC48-1_GL0148775	COG0334	Glutamate dehydrogenase/leucine dehydrogenase	Amino acid transport and metabolism [E]	Alanine, aspartate and glutamate metabolism	Ruminococcus bromii	Species	Firmicutes	Clostridia	Clostridiales	Ruminococcaceae	Ruminococcus	Ruminococcus bromii	-0.5155	0.0025	**
40	17690	MH0122_GL0011708	COG0050	Translation elongation factor EF-Tu, a GTPase	Translation, ribosomal structure and biogenesis [J]	Plant-pathogen interaction	Subdoligranulum variabile	Species	Firmicutes	Clostridia	Clostridiales	Ruminococcaceae	Subdoligranulum	Subdoligranulum variabile	-0.5313	0.0169	*
41	15670	MH0092_GL0036208	COG1653	ABC-type glycerol-3-phosphate transport system, periplasmic component	Carbohydrate transport and metabolism [G]	NAN	Faecalibacterium prausnitzii	Species	Firmicutes	Clostridia	Clostridiales	Ruminococcaceae	Faecalibacterium	Faecalibacterium prausnitzii	-0.6242	0.0245	*
42	22236	MH0188_GL0141556	COG0050	Translation elongation factor EF-Tu, a GTPase	Translation, ribosomal structure and biogenesis [J]	Plant-pathogen interaction	Collinsella	Genus	Actinobacteria	Coriobacteriia	Coriobacteriales	Coriobacteriaceae	Collinsella		-0.6933	0.0013	**

B (Obesity) - PLS-DA
PGs with VIP > 2

N	Protein Group ID	Leading razor protein accession	COG accession	COG name	COG category	KEGG name	LCA	Rank	Phylum	Class	Order	Family	Genus	Species	Log_{10} (Obesity yes/no)	t-test p-value Obesity yes/no	Significance Obesity yes/no
1	1139	537937.BLIG_01296	COG4166	ABC-type oligopeptide transport system, periplasmic component(3057)	Amino acid transport and metabolism [E]	ABC transporters	Bifidobacterium	Genus	Actinobacteria	Actinobacteria	Bifidobacteriales	Bifidobacteriaceae	Bifidobacterium		-0.4142	0.0571	
2	11463	MH0037_GL0027576			NAN	NAN	Bacteria	Superkingdom							-0.4093	0.0070	**
3	14026	MH0073_GL0056444			NAN	Galactose metabolism	Ruminococcus bromii	Species	Firmicutes	Clostridia	Clostridiales	Ruminococcaceae	Ruminococcus	Ruminococcus bromii	0.2287	0.0365	*
4	1460	657323.CK1_21680	COG4213	ABC-type xylose transport system, periplasmic component(416)	Carbohydrate transport and metabolism [G]	ABC transporters	Clostridiales	Order	Firmicutes	Clostridia	Clostridiales				-0.3287	0.0193	*
5	17022	MH0110_GL0036341	COG1866	Phosphoenolpyruvate carboxykinase, ATP-dependent(1458)	Energy production and conversion [C]	Glycolysis / Gluconeogenesis	Faecalibacterium prausnitzii	Species	Firmicutes	Clostridia	Clostridiales	Ruminococcaceae	Faecalibacterium	Faecalibacterium prausnitzii	0.3361	0.0589	
6	21902	MH0188_GL0028315	COG1653	ABC-type glycerol-3-phosphate transport system, periplasmic component(15818)	Carbohydrate transport and metabolism [G]	ABC transporters	Clostridiales	Order	Firmicutes	Clostridia	Clostridiales				-0.2308	0.0661	
7	22018	MH0188_GL0066755	COG0103	Ribosomal protein S9(1679)	Translation, ribosomal structure and biogenesis [J]	Ribosome	Bifidobacterium	Genus	Actinobacteria	Actinobacteria	Bifidobacteriales	Bifidobacteriaceae	Bifidobacterium		-0.1950	0.0574	
8	22027	MH0188_GL0067976	COG1129	ABC-type sugar transport system, ATPase component(5507)	Carbohydrate transport and metabolism [G]	ABC transporters	Bifidobacterium	Genus	Actinobacteria	Actinobacteria	Bifidobacteriales	Bifidobacteriaceae	Bifidobacterium		-0.2782	0.0600	
9	23262	MH0197_GL0121100	COG1653	ABC-type glycerol-3-phosphate transport system, periplasmic component(15818)	Carbohydrate transport and metabolism [G]	ABC transporters	Clostridiales bacterium KLE1615	Species	Firmicutes	Clostridia	Clostridiales			Clostridiales bacterium KLE1615	-0.3401	0.0343	*
10	2802	DLM017_GL0065099	COG1088	dTDP-D-glucose 4,6-dehydratase(3533)	Cell wall/membrane/envelope biogenesis [M]	Amino sugar and nucleotide sugar metabolism	Clostridiales	Order	Firmicutes	Clostridia	Clostridiales				0.3145	0.0066	**
11	2839	DLM018_GL0068485	COG1866	Phosphoenolpyruvate carboxykinase, ATP-dependent(1458)	Energy production and conversion [C]	Glycolysis / Gluconeogenesis	[Eubacterium] rectale	Species	Firmicutes	Clostridia	Clostridiales	Lachnospiraceae		[Eubacterium] rectale	0.2306	0.0643	
12	29633	MH0321_GL0040229	COG1350	Predicted alternative tryptophan synthase beta-subunit (paralog of TrpB)(894)	Amino acid transport and metabolism [E]	Glycine, serine and threonine metabolism	Ruminococcus	Genus	Firmicutes	Clostridia	Clostridiales	Ruminococcaceae	Ruminococcus		0.3567	0.0076	**
13	30964	MH0350_GL0092393	COG2407	L-fucose isomerase or related protein(2583)	Carbohydrate transport and metabolism [G]	Fructose and mannose metabolism	Clostridiales	Order	Firmicutes	Clostridia	Clostridiales				-0.3917	0.0429	*
14	32712	MH0383_GL0119529	COG0126	3-phosphoglycerate kinase(2251)	Carbohydrate transport and metabolism [G]	Glycolysis / Gluconeogenesis	Ruminococcus bromii	Species	Firmicutes	Clostridia	Clostridiales	Ruminococcaceae	Ruminococcus	Ruminococcus bromii	0.3604	0.0649	
15	33207	MH0396_GL0047332	COG3842	ABC-type Fe3+/spermidine/putrescine transport systems, ATPase components(6455)	Amino acid transport and metabolism [E]	ABC transporters	Clostridiales	Order	Firmicutes	Clostridia	Clostridiales				0.3136	0.0407	*
16	37699	NLM023_GL0007606	COG1879	ABC-type sugar transport system, periplasmic component, contains N-terminal xre family HTH domain	Carbohydrate transport and metabolism [G]	ABC transporters	Clostridium	Genus	Firmicutes	Clostridia	Clostridiales	Clostridiaceae	Clostridium		-0.5646	0.0023	**
17	39746	O2.UC15-1_GL0073124	COG0149	Triosephosphate isomerase	Carbohydrate transport and metabolism [G]	Glycolysis / Gluconeogenesis	Blautia	Genus	Firmicutes	Clostridia	Clostridiales	Lachnospiraceae	Blautia		-0.1995	0.0252	*
18	42060	O2.UC40-1_GL0122610	COG1882	Pyruvate-formate lyase	Energy production and conversion [C]	Pyruvate metabolism	Clostridiales bacterium KLE1615	Species	Firmicutes	Clostridia	Clostridiales			Clostridiales bacterium KLE1615	0.3487	0.0108	*
19	43365	O2.UC55-0_GL0124819	COG0811	Biopolymer transport protein ExbB/TolQ	Intracellular trafficking, secretion, and vesicular transport [U]	Microbial metabolism in diverse environments	Parabacteroides	Genus	Bacteroidetes	Bacteroidia	Bacteroidales	Porphyromonadaceae	Parabacteroides		-0.2983	0.0536	
20	44201	SZEY-09A_GL0049065	COG0737	2',3'-cyclic-nucleotide 2'-phosphodiesterase/5'- or 3'-nucleotidase, 5'-nucleotidase family	Defense mechanisms [V]	Purine metabolism	Faecalibacterium prausnitzii	Species	Firmicutes	Clostridia	Clostridiales	Ruminococcaceae	Faecalibacterium	Faecalibacterium prausnitzii	-0.3354	0.0407	*
21	44443	SZEY-38A_GL0037167	COG0137	Argininosuccinate synthase	Amino acid transport and metabolism [E]	Alanine, aspartate and glutamate metabolism	Ruminococcus bromii	Species	Firmicutes	Clostridia	Clostridiales	Ruminococcaceae	Ruminococcus	Ruminococcus bromii	0.3841	0.0271	*
22	45245	T2D-133A_GL0070710	COG4771	Outer membrane receptor for ferrienterochelin and colicins	Inorganic ion transport and metabolism [P]	NAN	Bacteroides uniformis	Species	Bacteroidetes	Bacteroidia	Bacteroidales	Bacteroidaceae	Bacteroides	Bacteroides uniformis	-0.4077	0.0526	
23	45433	T2D-142A_GL0079303	COG0457	Tetratricopeptide (TPR) repeat	General function prediction only [R]	NAN	Bacteroides eggerthii	Species	Bacteroidetes	Bacteroidia	Bacteroidales	Bacteroidaceae	Bacteroides	Bacteroides eggerthii	-0.4191	0.0544	
24	4759	MH0002_GL0028993			NAN	NAN	Bacteria	Superkingdom							-0.3293	0.0491	*
25	47769	V1.CD29-0_GL0134727	COG1879	ABC-type sugar transport system, periplasmic component, contains N-terminal xre family HTH domain	Carbohydrate transport and metabolism [G]	ABC transporters	Dorea longicatena	Species	Firmicutes	Clostridia	Clostridiales	Lachnospiraceae	Dorea	Dorea longicatena	-0.4148	0.0550	
26	48654	V1.CD50-0_GL0068523	COG0138	AICAR transformylase/IMP cyclohydrolase PurH	Nucleotide transport and metabolism [F]	Purine metabolism	Ruminococcus bromii	Species	Firmicutes	Clostridia	Clostridiales	Ruminococcaceae	Ruminococcus	Ruminococcus bromii	0.1836	0.0622	
27	49334	V1.FI02_GL0017922	COG0542	ATP-dependent Clp protease ATP-binding subunit ClpA	Posttranslational modification, protein turnover, chaperones [O]	NAN	Blautia obeum	Species	Firmicutes	Clostridia	Clostridiales	Lachnospiraceae	Blautia	Blautia obeum	-0.3183	0.0451	*
28	5041	MH0002_GL0050870	COG1048	Aconitase A	Energy production and conversion [C]	Citrate cycle (TCA cycle)	Clostridiales	Order	Firmicutes	Clostridia	Clostridiales				0.2212	0.0602	
29	52509	V1.UC40-0_GL0130575	COG3209	Uncharacterized conserved protein RhaS, contains 28 RHS repeats	General function prediction only [R]	NAN	Bacillus subtilis	Species	Firmicutes	Bacilli	Bacillales	Bacillaceae	Bacillus	Bacillus subtilis	-0.5498	0.0120	*
30	52581	V1.UC41-0_GL0045739	COG1653	ABC-type glycerol-3-phosphate transport system, periplasmic component	Carbohydrate transport and metabolism [G]	ABC transporters	Faecalibacterium prausnitzii	Species	Firmicutes	Clostridia	Clostridiales	Ruminococcaceae	Faecalibacterium	Faecalibacterium prausnitzii	-0.4085	0.0434	*
31	52610	V1.UC42-0_GL0049062	COG0329	Dihydrodipicolinate synthase/N-acetylneuraminate lyase	Cell wall/membrane/envelope biogenesis [M]	Lysine biosynthesis	Clostridiales	Order	Firmicutes	Clostridia	Clostridiales				-0.2069	0.0192	*
32	6649	MH0006_GL0003713	COG0493	NADPH-dependent glutamate synthase beta chain or related oxidoreductase	General function prediction only [R]	Alanine, aspartate and glutamate metabolism	Ruminococcus bromii	Species	Firmicutes	Clostridia	Clostridiales	Ruminococcaceae	Ruminococcus	Ruminococcus bromii	0.3133	0.0161	*
33	9003	MH0012_GL0138305	COG0443	Molecular chaperone DnaK (HSP70)	Posttranslational modification, protein turnover, chaperones [O]	RNA degradation	Subdoligranulum variabile	Species	Firmicutes	Clostridia	Clostridiales	Ruminococcaceae	Subdoligranulum	Subdoligranulum variabile	-0.2606	0.0437	*
34	9211	MH0012_GL0193632	COG4799	Acetyl-CoA carboxylase, carboxyltransferase component	Lipid transport and metabolism [I]	Fatty acid biosynthesis	Dialister	Genus	Firmicutes	Negativicutes	Veillonellales	Veillonellaceae	Dialister		-0.4785	0.0092	**

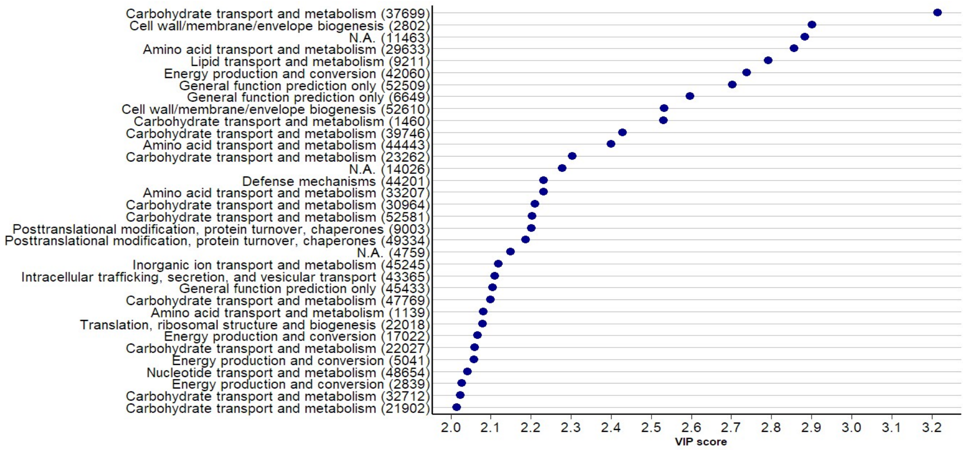
Carbohydrate transport and metabolism (37699)
Cell wall/membrane/envelope biogenesis (2802)
N.A. (11463)
Amino acid transport and metabolism (29633)
Lipid transport and metabolism (9211)
Energy production and conversion (42060)
General function prediction only (52509)
General function prediction only (6649)
Cell wall/membrane/envelope biogenesis (52610)
Carbohydrate transport and metabolism (1460)
Carbohydrate transport and metabolism (39746)
Amino acid transport and metabolism (44443)
Carbohydrate transport and metabolism (23262)
N.A. (14026)
Defense mechanisms (44201)
Amino acid transport and metabolism (33207)
Carbohydrate transport and metabolism (30964)
Carbohydrate transport and metabolism (52581)
Posttranslational modification, protein turnover, chaperones (9003)
Posttranslational modification, protein turnover, chaperones (49334)
N.A. (4759)
Inorganic ion transport and metabolism (45245)
Intracellular trafficking, secretion, and vesicular transport (43365)
General function prediction only (45433)
Carbohydrate transport and metabolism (47769)
Amino acid transport and metabolism (1139)
Translation, ribosomal structure and biogenesis (22018)
Energy production and conversion (17022)
Carbohydrate transport and metabolism (22027)
Energy production and conversion (5041)
Nucleotide transport and metabolism (48654)
Energy production and conversion (2839)
Carbohydrate transport and metabolism (32712)
Carbohydrate transport and metabolism (21902)
2.0 2.1 2.2 2.3 2.4 2.5 2.6 2.7 2.8 2.9 3.0 3.1 3.2
VIP score

B (Obesity) - differentially expressed PGs

N	Protein Group ID	Leading razor protein accession	COG accession	COG name	COG category	KEGG name	LCA	Rank	Phylum	Class	Order	Family	Genus	Species	Log10 (Obesity yes/no)	t-test p-value Obesity yes/no	Significance Obesity yes/no
1	44443	SZEY-38A_GL0037167	COG0137	Argininosuccinate synthase	Amino acid transport and metabolism [E]	Alanine, aspartate and glutamate metabolism	Ruminococcus bromii	Species	Firmicutes	Clostridia	Clostridiales	Ruminococcaceae	Ruminococcus	Ruminococcus bromii	0.3841	0.0271	*
2	29633	MH0321_GL0040229	COG1350	Predicted alternative tryptophan synthase beta-subunit (paralog of TrpB)	Amino acid transport and metabolism [E]	Glycine, serine and threonine metabolism	Ruminococcus	Genus	Firmicutes	Clostridia	Clostridiales	Ruminococcaceae	Ruminococcus		0.3567	0.0076	**
3	42060	O2.UC40-1_GL0122610	COG1882	Pyruvate-formate lyase	Energy production and conversion [C]	Pyruvate metabolism	Clostridiales bacterium KLE1615	Species	Firmicutes	Clostridia	Clostridiales			Clostridiales bacterium KLE1615	0.3487	0.0108	*
4	2802	DLM017_GL0065099	COG1088	dTDP-D-glucose 4,6-dehydratase	Cell wall/membrane/envelope biogenesis [M]	Amino sugar and nucleotide sugar metabolism	Clostridiales	Order	Firmicutes	Clostridia	Clostridiales				0.3145	0.0066	**
5	33207	MH0396_GL0047332	COG3842	ABC-type Fe3+/spermidine/putrescine transport systems, ATPase components	Amino acid transport and metabolism [E]	ABC transporters	Clostridiales	Order	Firmicutes	Clostridia	Clostridiales				0.3136	0.0407	*
6	6649	MH0006_GL0003713	COG0493	NADPH-dependent glutamate synthase beta chain or related oxidoreductase	General function prediction only [R]	Alanine, aspartate and glutamate metabolism	Ruminococcus bromii	Species	Firmicutes	Clostridia	Clostridiales	Ruminococcaceae	Ruminococcus	Ruminococcus bromii	0.3133	0.0161	*
7	14026	MH0073_GL0056444			NAN	Galactose metabolism	Ruminococcus bromii	Species	Firmicutes	Clostridia	Clostridiales	Ruminococcaceae	Ruminococcus	Ruminococcus bromii	0.2287	0.0365	*
8	39746	O2.UC15-1_GL0073124	COG0149	Triosephosphate isomerase	Carbohydrate transport and metabolism [G]	Glycolysis / Gluconeogenesis	Blautia	Genus	Firmicutes	Clostridia	Clostridiales	Lachnospiraceae	Blautia		-0.1995	0.0252	*
9	52610	V1.UC42-0_GL0049062	COG0329	Dihydrodipicolinate synthase/N-acetylneuraminate lyase	Cell wall/membrane/envelope biogenesis [M]	Lysine biosynthesis	Clostridiales	Order	Firmicutes	Clostridia	Clostridiales				-0.2069	0.0192	*
10	9003	MH0012_GL0138305	COG0443	Molecular chaperone DnaK (HSP70)	Posttranslational modification, protein turnover, chaperones [O]	RNA degradation	Subdoligranulum variabile	Species	Firmicutes	Clostridia	Clostridiales	Ruminococcaceae	Subdoligranulum	Subdoligranulum variabile	-0.2606	0.0437	*
11	49334	V1.FI02_GL0017922	COG0542	ATP-dependent Clp protease ATP-binding subunit ClpA	Posttranslational modification, protein turnover, chaperones [O]	NAN	Blautia obeum	Species	Firmicutes	Clostridia	Clostridiales	Lachnospiraceae	Blautia	Blautia obeum	-0.3183	0.0451	*
12	1460	657323.CK1_21680	COG4213	ABC-type xylose transport system, periplasmic component	Carbohydrate transport and metabolism [G]	ABC transporters	Clostridiales	Order	Firmicutes	Clostridia	Clostridiales				-0.3287	0.0193	*
13	4759	MH0002_GL0028993			NAN	NAN	Bacteria	Superkingdom							-0.3293	0.0491	*
14	44201	SZEY-09A_GL0049065	COG0737	2',3'-cyclic-nucleotide 2'-phosphodiesterase/5'- or 3'-nucleotidase, 5'-nucleotidase family	Defense mechanisms [V]	Purine metabolism	Faecalibacterium prausnitzii	Species	Firmicutes	Clostridia	Clostridiales	Ruminococcaceae	Faecalibacterium	Faecalibacterium prausnitzii	-0.3354	0.0407	*
15	23262	MH0197_GL0121100	COG1653	ABC-type glycerol-3-phosphate transport system, periplasmic component	Carbohydrate transport and metabolism [G]	ABC transporters	Clostridiales bacterium KLE1615	Species	Firmicutes	Clostridia	Clostridiales			Clostridiales bacterium KLE1615	-0.3401	0.0343	*
16	30964	MH0350_GL0092393	COG2407	L-fucose isomerase or related protein	Carbohydrate transport and metabolism [G]	Fructose and mannose metabolism	Clostridiales	Order	Firmicutes	Clostridia	Clostridiales				-0.3917	0.0429	*
17	52581	V1.UC41-0_GL0045739	COG1653	ABC-type glycerol-3-phosphate transport system, periplasmic component	Carbohydrate transport and metabolism [G]	ABC transporters	Faecalibacterium prausnitzii	Species	Firmicutes	Clostridia	Clostridiales	Ruminococcaceae	Faecalibacterium	Faecalibacterium prausnitzii	-0.4085	0.0434	*
18	11463	MH0037_GL0027576			NAN	NAN	Bacteria	Superkingdom							-0.4093	0.0070	**
19	9211	MH0012_GL0193632	COG4799	Acetyl-CoA carboxylase, carboxyltransferase component	Lipid transport and metabolism [I]	Fatty acid biosynthesis	Dialister	Genus	Firmicutes	Negativicutes	Veillonellales	Veillonellaceae	Dialister		-0.4785	0.0092	**
20	52509	V1.UC40-0_GL0130575	COG3209	Uncharacterized conserved protein RhaS, contains 28 RHS repeats	General function prediction only [R]	NAN	Bacillus subtilis	Species	Firmicutes	Bacilli	Bacillales	Bacillaceae	Bacillus	Bacillus subtilis	-0.5498	0.0120	*
21	37699	NLM023_GL0007606	COG1879	ABC-type sugar transport system, periplasmic component, contains N-terminal xre family HTH domain	Carbohydrate transport and metabolism [G]	ABC transporters	Clostridium	Genus	Firmicutes	Clostridia	Clostridiales	Clostridiaceae	Clostridium		-0.5646	0.0023	**

C (GI symptoms) - PLS-DA
PGs with VIP > 2

N	Protein Group ID	Leading razor protein accession	COG accession	COG name	COG category	KEGG name	LCA	Rank	Phylum	Class	Order	Family	Genus	Species	Log_{10} (GI symptoms yes/no)	t-test p-value GI symptoms yes/no	Significance GI symptoms yes/no
1	1991	BGI-33A_GL0073034	COG0822	NifU homolog involved in Fe-S cluster formation	Posttranslational modification, protein turnover, chaperones [O]	NAN	Ruminococcus bromii	Species	Firmicutes	Clostridia	Clostridiales	Ruminococcaceae	Ruminococcus	Ruminococcus bromii	-0.2537	0.0466	*
2	2780	DLM016_GL0028957	COG1592	Rubrerythrin	Energy production and conversion [C]	NAN	Ruminococcus bromii	Species	Firmicutes	Clostridia	Clostridiales	Ruminococcaceae	Ruminococcus	Ruminococcus bromii	-0.2933	0.0445	*
3	4272	MH0001_GL0015313	COG1145	Ferredoxin	Energy production and conversion [C]	Glycolysis / Gluconeogenesis	Roseburia faecis	Species	Firmicutes	Clostridia	Clostridiales	Lachnospiraceae	Roseburia	Roseburia faecis	-0.2624	0.0267	*
4	4411	MH0002_GL0000742	COG0035	Uracil phosphoribosyltransferase	Nucleotide transport and metabolism [F]	Pyrimidine metabolism	Subdoligranulum variabile	Species	Firmicutes	Clostridia	Clostridiales	Ruminococcaceae	Subdoligranulum	Subdoligranulum variabile	0.1017	0.0415	*
5	4871	MH0002_GL0038347	COG0297	Glycogen synthase	Carbohydrate transport and metabolism [G]	Galactose metabolism	Ruminococcaceae	Species	Firmicutes	Clostridia	Clostridiales	Ruminococcaceae	Subdoligranulum	Subdoligranulum variabile	0.1812	0.0563	
6	4889	MH0002_GL0039425	COG1362	Aspartyl aminopeptidase	Amino acid transport and metabolism [E]	NAN	[Eubacterium] rectale	Species	Firmicutes	Clostridia	Clostridiales	Lachnospiraceae		[Eubacterium] rectale	0.2006	0.0526	
7	5311	MH0002_GL0075003	COG1472	Periplasmic beta-glucosidase and related glycosidases	Carbohydrate transport and metabolism [G]	Cyanoamino acid metabolism	Bacteria	Superkingdom							0.2438	0.0070	**
8	5354	MH0003_GL0002731	COG0172	Seryl-tRNA synthetase	Translation, ribosomal structure and biogenesis [J]	Aminoacyl-tRNA biosynthesis	Ruminococcus bromii	Species	Firmicutes	Clostridia	Clostridiales	Ruminococcaceae	Ruminococcus	Ruminococcus bromii	-0.1393	0.0419	*
9	5388	MH0003_GL0005201	COG2182	Maltose-binding periplasmic protein MalE	Carbohydrate transport and metabolism [G]	ABC transporters	Ruminococcus bromii	Species	Firmicutes	Clostridia	Clostridiales	Ruminococcaceae	Ruminococcus	Ruminococcus bromii	-0.3760	0.0548	
10	5429	MH0003_GL0009354	COG0297	Glycogen synthase	Carbohydrate transport and metabolism [G]	Galactose metabolism	Ruminococcus bromii	Species	Firmicutes	Clostridia	Clostridiales	Ruminococcaceae	Ruminococcus	Ruminococcus bromii	-0.2237	0.0405	*
11	5555	MH0003_GL0030511	COG0845	Multidrug efflux pump subunit AcrA (membrane-fusion protein)	Defense mechanisms [V]	Purine metabolism	Ruminococcus bromii	Species	Firmicutes	Clostridia	Clostridiales	Ruminococcaceae	Ruminococcus	Ruminococcus bromii	-0.2967	0.0105	*
12	5557	MH0003_GL0030541	COG0047	Phosphoribosylformylglycinamidine (FGAM) synthase, glutamine amidotransferase domain	Nucleotide transport and metabolism [F]	Purine metabolism	Ruminococcus bromii	Species	Firmicutes	Clostridia	Clostridiales	Ruminococcaceae	Ruminococcus	Ruminococcus bromii	-0.3307	0.0102	*
13	5795	MH0003_GL0062474	COG0166	Glucose-6-phosphate isomerase	Carbohydrate transport and metabolism [G]	Glycolysis / Gluconeogenesis	Ruminococcus bromii	Species	Firmicutes	Clostridia	Clostridiales	Ruminococcaceae	Ruminococcus	Ruminococcus bromii	-0.2862	0.0185	*
14	5986	O2.UC48-0_GL0114931	COG0329	Dihydrodipicolinate synthase/N-acetylneuraminate lyase	Cell wall/membrane/envelope biogenesis [M]	Lysine biosynthesis	Ruminococcus bromii	Species	Firmicutes	Clostridia	Clostridiales	Ruminococcaceae	Ruminococcus	Ruminococcus bromii	-0.2028	0.0589	
15	13391	MH0062_GL0058668	COG4166	ABC-type oligopeptide transport system, periplasmic component	Amino acid transport and metabolism [E]	ABC transporters	Faecalibacterium prausnitzii	Species	Firmicutes	Clostridia	Clostridiales	Ruminococcaceae	Faecalibacterium	Faecalibacterium prausnitzii	0.2760	0.0435	*
16	14619	MH0086_GL0046721	COG1143	Formate hydrogenlyase subunit 6/NADH:ubiquinone oxidoreductase 23 kD subunit (chain I)	Energy production and conversion [C]	Glycolysis / Gluconeogenesis	Ruminococcus	Genus	Firmicutes	Clostridia	Clostridiales	Ruminococcaceae	Ruminococcus		-0.2808	0.0612	
17	14652	MH0086_GL0058027	COG4624	Iron only hydrogenase large subunit, C-terminal domain	Energy production and conversion [C]	Oxidative phosphorylation	Clostridiales	Order	Firmicutes	Clostridia	Clostridiales				-0.1338	0.0584	
18	16733	MH0106_GL0002286	COG1053	Succinate dehydrogenase/fumarate reductase, flavoprotein subunit	Energy production and conversion [C]	Citrate cycle (TCA cycle)	Bacteroides coprocola	Species	Bacteroidetes	Bacteroidia	Bacteroidales	Bacteroidaceae	Bacteroides	Bacteroides coprocola	0.2137	0.0447	*
19	16919	MH0136_GL0026411	COG1250	3-hydroxyacyl-CoA dehydrogenase	Lipid transport and metabolism [I]	Fatty acid degradation	Faecalibacterium prausnitzii	Species	Firmicutes	Clostridia	Clostridiales	Ruminococcaceae	Faecalibacterium	Faecalibacterium prausnitzii	0.3102	0.0551	
20	16948	MH0108_GL0083509	COG0104	Adenylosuccinate synthase	Nucleotide transport and metabolism [F]	Purine metabolism	Faecalibacterium prausnitzii	Species	Firmicutes	Clostridia	Clostridiales	Ruminococcaceae	Faecalibacterium	Faecalibacterium prausnitzii	-0.1854	0.0437	*
21	18636	MH0131_GL0088691	COG1080	Phosphoenolpyruvate-protein kinase (PTS system EI component in bacteria)	Carbohydrate transport and metabolism [G]	Pyruvate metabolism	Coprococcus	Genus	Firmicutes	Clostridia	Clostridiales	Lachnospiraceae	Coprococcus		0.2153	0.0441	*
22	18821	MH0131_GL0138470	COG1882	Pyruvate-formate lyase	Energy production and conversion [C]	Pyruvate metabolism	Fusicatenibacter saccharivorans	Species	Firmicutes	Clostridia	Clostridiales	Lachnospiraceae	Fusicatenibacter	Fusicatenibacter saccharivorans	0.1791	0.0488	*
23	21666	MH0184_GL0127793	COG0092	Ribosomal protein S3	Translation, ribosomal structure and biogenesis [J]	Ribosome	Ruminococcus bromii	Species	Firmicutes	Clostridia	Clostridiales	Ruminococcaceae	Ruminococcus	Ruminococcus bromii	-0.1770	0.0530	
24	25836	MH0002_GL0050951	COG1145	Ferredoxin	Energy production and conversion [C]	Glycolysis / Gluconeogenesis	Clostridiales	Species	Firmicutes	Clostridia	Clostridiales	Ruminococcaceae	Faecalibacterium	Faecalibacterium prausnitzii	-0.2518	0.0302	*
25	29633	MH0321_GL0040229	COG1350	Predicted alternative tryptophan synthase beta-subunit (paralog of TrpB)	Amino acid transport and metabolism [E]	Glycine, serine and threonine metabolism	Ruminococcus	Genus	Firmicutes	Clostridia	Clostridiales	Ruminococcaceae	Ruminococcus		-0.2121	0.0504	
26	34201	MH0415_GL0203137	COG0104	Adenylosuccinate synthase	Nucleotide transport and metabolism [F]	Purine metabolism	Ruminococcus bromii	Species	Firmicutes	Clostridia	Clostridiales	Ruminococcaceae	Ruminococcus	Ruminococcus bromii	-0.1871	0.0183	*
27	35568	MH0441_GL0130959	COG0334	Glutamate dehydrogenase/leucine dehydrogenase	Amino acid transport and metabolism [E]	Alanine, aspartate and glutamate metabolism	Clostridiales	Order	Firmicutes	Clostridia	Clostridiales				-0.1924	0.0020	**
28	44507	SZEY-44A_GL0015657	COG1454	Alcohol dehydrogenase, class IV	Energy production and conversion [C]	NAN	Ruminococcus bromii	Species	Firmicutes	Clostridia	Clostridiales	Ruminococcaceae	Ruminococcus	Ruminococcus bromii	-0.2502	0.0311	*
29	48871	V1.CD54-0_GL0122997	COG0334	Glutamate dehydrogenase/leucine dehydrogenase	Amino acid transport and metabolism [E]	Alanine, aspartate and glutamate metabolism	Firmicutes	Phylum	Firmicutes						-0.1702	0.0513	

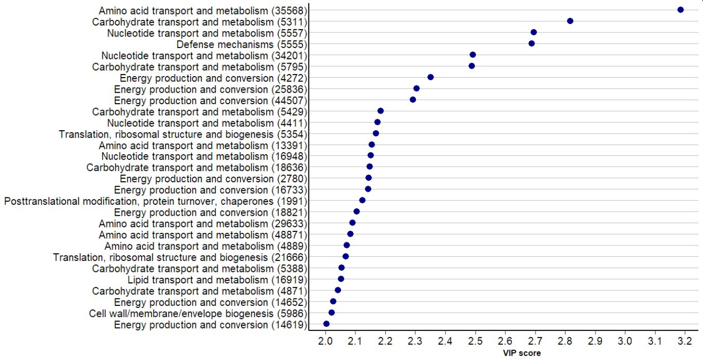

C (GI symptoms) - differentially expressed PGs

N	Protein Group ID	Leading razor protein accession	COG accession	COG name	COG category	KEGG name	LCA	Rank	Phylum	Class	Order	Family	Genus	Species	Log_{10} (GI symptoms yes/no)	t-test p-value GI symptoms yes/no	Significance GI symptoms yes/no
1	13391	MH0062_GL0058668	COG4166	ABC-type oligopeptide transport system, periplasmic component	Amino acid transport and metabolism [E]	ABC transporters	Faecalibacterium prausnitzii	Species	Firmicutes	Clostridia	Clostridiales	Ruminococcaceae	Faecalibacterium	Faecalibacterium prausnitzii	0.2760	0.0435	*
2	5311	MH0002_GL0075003	COG1472	Periplasmic beta-glucosidase and related glycosidases	Carbohydrate transport and metabolism [G]	Cyanoamino acid metabolism	Bacteria	Superkingdom							0.2438	0.0070	**
3	18636	MH0131_GL0088691	COG1080	Phosphoenolpyruvate-protein kinase (PTS system EI component in bacteria)	Carbohydrate transport and metabolism [G]	Pyruvate metabolism	Coprococcus	Genus	Firmicutes	Clostridia	Clostridiales	Lachnospiraceae	Coprococcus		0.2153	0.0441	*
4	16733	MH0106_GL0002286	COG1053	Succinate dehydrogenase/fumarate reductase, flavoprotein subunit	Energy production and conversion [C]	Citrate cycle (TCA cycle)	Bacteroides coprocola	Species	Bacteroidetes	Bacteroidia	Bacteroidales	Bacteroidaceae	Bacteroides	Bacteroides coprocola	0.2137	0.0447	*
5	18821	MH0131_GL0138470	COG1882	Pyruvate-formate lyase	Energy production and conversion [C]	Pyruvate metabolism	Fusicatenibacter saccharivorans	Species	Firmicutes	Clostridia	Clostridiales	Lachnospiraceae	Fusicatenibacter	Fusicatenibacter saccharivorans	0.1791	0.0488	*
6	16948	MH0108_GL0083509	COG0104	Adenylosuccinate synthase	Nucleotide transport and metabolism [F]	Purine metabolism	Faecalibacterium prausnitzii	Species	Firmicutes	Clostridia	Clostridiales	Ruminococcaceae	Faecalibacterium	Faecalibacterium prausnitzii	-0.1854	0.0437	*
7	34201	MH0415_GL0203137	COG0104	Adenylosuccinate synthase	Nucleotide transport and metabolism [F]	Purine metabolism	Ruminococcus bromii	Species	Firmicutes	Clostridia	Clostridiales	Ruminococcaceae	Ruminococcus	Ruminococcus bromii	-0.1871	0.0183	*
8	35568	MH0441_GL0130959	COG0334	Glutamate dehydrogenase/leucine dehydrogenase	Amino acid transport and metabolism [E]	Alanine, aspartate and glutamate metabolism	Clostridiales	Order	Firmicutes	Clostridia	Clostridiales				-0.1924	0.0020	**
9	5429	MH0003_GL0009354	COG0297	Glycogen synthase	Carbohydrate transport and metabolism [G]	Galactose metabolism	Ruminococcus bromii	Species	Firmicutes	Clostridia	Clostridiales	Ruminococcaceae	Ruminococcus	Ruminococcus bromii	-0.2237	0.0405	*
10	44507	NLF006_GL0012500	COG1454	Alcohol dehydrogenase, class IV	Energy production and conversion [C]	NAN	Ruminococcus bromii	Species	Firmicutes	Clostridia	Clostridiales	Ruminococcaceae	Ruminococcus	Ruminococcus bromii	-0.2502	0.0311	*
11	25836	MH0239_GL0084186	COG1145	Ferredoxin	Energy production and conversion [C]	Glycolysis / Gluconeogenesis	Clostridiales	Species	Firmicutes	Clostridia	Clostridiales	Ruminococcaceae	Faecalibacterium	Faecalibacterium prausnitzii	-0.2518	0.0302	*
12	1991	BGI-33A_GL0073034	COG0822	NifU homolog involved in Fe-S cluster formation	Posttranslational modification, protein turnover, chaperones [O]	NAN	Ruminococcus bromii	Species	Firmicutes	Clostridia	Clostridiales	Ruminococcaceae	Ruminococcus	Ruminococcus bromii	-0.2537	0.0466	*
13	4272	MH0001_GL0015313	COG1145	Ferredoxin	Energy production and conversion [C]	Glycolysis / Gluconeogenesis	Roseburia faecis	Species	Firmicutes	Clostridia	Clostridiales	Lachnospiraceae	Roseburia	Roseburia faecis	-0.2624	0.0267	*
14	5795	MH0003_GL0062474	COG0166	Glucose-6-phosphate isomerase	Carbohydrate transport and metabolism [G]	Glycolysis / Gluconeogenesis	Ruminococcus bromii	Species	Firmicutes	Clostridia	Clostridiales	Ruminococcaceae	Ruminococcus	Ruminococcus bromii	-0.2862	0.0185	*
15	2780	DLM016_GL0028957	COG1592	Rubrerythrin	Energy production and conversion [C]	NAN	Ruminococcus bromii	Species	Firmicutes	Clostridia	Clostridiales	Ruminococcaceae	Ruminococcus	Ruminococcus bromii	-0.2933	0.0445	*
16	5555	MH0003_GL0030511	COG0845	Multidrug efflux pump subunit AcrA (membrane-fusion protein)	Defense mechanisms [V]	Purine metabolism	Ruminococcus bromii	Species	Firmicutes	Clostridia	Clostridiales	Ruminococcaceae	Ruminococcus	Ruminococcus bromii	-0.2967	0.0105	*
17	5557	MH0003_GL0030541	COG0047	Phosphoribosylformylglycinamidine (FGAM) synthase, glutamine amidotransferase domain	Nucleotide transport and metabolism [F]	Purine metabolism	Ruminococcus bromii	Species	Firmicutes	Clostridia	Clostridiales	Ruminococcaceae	Ruminococcus	Ruminococcus bromii	-0.3307	0.0102	*

Univariate analysis

Comparisons			Kruskal-Wallis	Kruskal-Wallis p-value	Kruskal-Wallis Significance	ANOVA	ANOVA p-value	ANOVA Significance	Mann-Whitney	Mann-Whitney p-value	Mann-Whitney Significance	0 count	0 mean	0 std	1 count	1 mean	1 std
**A.**	**Omnivorous diet: no (= 1) vs yes (= 0)**	1 vs 0	3.375324071	0.066179563		6.097185	0.013546029	*	60476336	0.066179707		20576	7.580099623	0.525328492	5787	7.561162938	0.478364152
**B**	**Obesity: yes (1) vs no (0)**	1 vs 0	8.30368836	0.003956465	**	6.359583	0.011680742	*	55986386.5	0.003956477	**	21219	7.579884202	0.515438273	5144	7.559684465	0.515157078
**C**	**Presence of GI symtoms: yes (1) vs no (0)**	1 vs 0	1.116209465	0.290736121		1.779225	0.182256064		87061850	0.290736491		12217	7.580499351	0.513686525	14146	7.572007578	0.516927629
