# Supplementary material for: Williams–Beuren syndrome shapes the gut microbiota metaproteome
Source: Sci Rep. 2023 Nov 3;13:18963. doi: 10.1038/s41598-023-46052-9 (PMC10624682; doi:10.1038/s41598-023-46052-9)
Supplement: Supplementary file 7 — Supplementary File 6. [file 41598_2023_46052_MOESM7_ESM.pdf]

42 identified human PGs, Enriched terms

<table>
<tr><th>Category</th><th>CategoryID</th><th>GO</th><th>Description</th><th>PARENT_GO</th><th>LogP</th><th>P-value</th><th>Enrichment</th><th>Z-score</th><th>#TotalGeneInLibrary</th><th>#GeneInGO</th><th>#GeneInHitList</th><th>#GeneInGOAndHitList</th><th>%InGO</th><th>STDV %InGO</th><th>GeneID</th><th>Hits</th><th>Log (q-value)</th><th>q-value</th></tr>
<tr><td>KEGG Pathway</td><td>24</td><td>hsa04972</td><td>Pancreatic secretion</td><td></td><td>-12</td><td>1E-12</td><td>58</td><td>21</td><td>30198</td><td>102</td><td>41</td><td>8</td><td>20</td><td>6.2</td><td>276|280|1357|1358|1360|5319|5645|440387</td><td>AMY1A|AMY2B|CPA1|CPA2|CPB1|PLA2G1B|PRSS2|CTRB2</td><td>-7.5</td><td>3.16228E-08</td></tr>
<tr><td>Canonical Pathways</td><td>11</td><td>M5885</td><td>NABA MATRISOME ASSOCIATED</td><td></td><td>-11</td><td>1E-11</td><td>13</td><td>12</td><td>30198</td><td>751</td><td>41</td><td>13</td><td>32</td><td>7.3</td><td>302|307|311|462|3960|4586|5269|5645|6279|6280|55600|56667|142683</td><td>ANXA2|ANXA4|ANXA11|SERPINC1|LGALS4|MUC5AC|SERPINB6|PRSS2|S100A8|S100A9|ITLN1|MUC13|ITLN2</td><td>-6.8</td><td>1.58489E-07</td></tr>
<tr><td>Reactome Gene Sets</td><td>6</td><td>R-HSA-6798695</td><td>Neutrophil degranulation</td><td></td><td>-11</td><td>1E-11</td><td>17</td><td>13</td><td>30198</td><td>482</td><td>41</td><td>11</td><td>27</td><td>6.9</td><td>290|302|4069|4311|4353|5269|5645|6279|6280|8288|8876</td><td>ANPEP|ANXA2|LYZ|MME|MPO|SERPINB6|PRSS2|S100A8|S100A9|EPX|VNN1</td><td>-6.5</td><td>3.16228E-07</td></tr>
<tr><td>GO Molecular Functions</td><td>21</td><td>GO:0008238</td><td>exopeptidase activity</td><td></td><td>-10</td><td>1E-10</td><td>53</td><td>19</td><td>30198</td><td>97</td><td>41</td><td>7</td><td>17</td><td>5.9</td><td>290|1357|1358|1360|1800|4311|130749</td><td>ANPEP|CPA1|CPA2|CPB1|DPEP1|MME|CPO</td><td>-6.4</td><td>3.98107E-07</td></tr>
<tr><td>Canonical Pathways</td><td>11</td><td>M5880</td><td>NABA ECM AFFILIATED</td><td></td><td>-10</td><td>1E-10</td><td>35</td><td>16</td><td>30198</td><td>170</td><td>41</td><td>8</td><td>20</td><td>6.2</td><td>302|307|311|3960|4586|55600|56667|142683</td><td>ANXA2|ANXA4|ANXA11|LGALS4|MUC5AC|ITLN1|MUC13|ITLN2</td><td>-6.4</td><td>3.98107E-07</td></tr>
<tr><td>GO Molecular Functions</td><td>21</td><td>GO:0005509</td><td>calcium ion binding</td><td></td><td>-9.9</td><td>1.25893E-10</td><td>12</td><td>11</td><td>30198</td><td>714</td><td>41</td><td>12</td><td>29</td><td>7.1</td><td>276|302|307|311|5319|5645|6279|6280|6717|7273|55600|56624</td><td>AMY1A|ANXA2|ANXA4|ANXA11|PLA2G1B|PRSS2|S100A8|S100A9|SRI|TTN|ITLN1|ASAH2</td><td>-6.3</td><td>5.01187E-07</td></tr>
<tr><td>GO Molecular Functions</td><td>21</td><td>GO:0008237</td><td>metallopeptidase activity</td><td></td><td>-9.9</td><td>1.25893E-10</td><td>32</td><td>16</td><td>30198</td><td>185</td><td>41</td><td>8</td><td>20</td><td>6.2</td><td>290|1357|1358|1360|1800|4311|5645|130749</td><td>ANPEP|CPA1|CPA2|CPB1|DPEP1|MME|PRSS2|CPO</td><td>-6.3</td><td>5.01187E-07</td></tr>
<tr><td>GO Molecular Functions</td><td>21</td><td>GO:0008235</td><td>metalloexopeptidase activity</td><td></td><td>-9.4</td><td>3.98107E-10</td><td>67</td><td>20</td><td>30198</td><td>66</td><td>41</td><td>6</td><td>15</td><td>5.5</td><td>290|1357|1358|1360|1800|130749</td><td>ANPEP|CPA1|CPA2|CPB1|DPEP1|CPO</td><td>-5.9</td><td>1.25893E-06</td></tr>
<tr><td>KEGG Pathway</td><td>24</td><td>hsa04974</td><td>Protein digestion and absorption</td><td></td><td>-8.3</td><td>5.01187E-09</td><td>43</td><td>16</td><td>30198</td><td>103</td><td>41</td><td>6</td><td>15</td><td>5.5</td><td>1357|1358|1360|4311|5645|440387</td><td>CPA1|CPA2|CPB1|MME|PRSS2|CTRB2</td><td>-4.8</td><td>1.58489E-05</td></tr>
<tr><td>GO Molecular Functions</td><td>21</td><td>GO:0008233</td><td>peptidase activity</td><td></td><td>-8.2</td><td>6.30957E-09</td><td>12</td><td>10</td><td>30198</td><td>608</td><td>41</td><td>10</td><td>24</td><td>6.7</td><td>290|1357|1358|1360|1800|4311|5645|11330|130749|440387</td><td>ANPEP|CPA1|CPA2|CPB1|DPEP1|MME|PRSS2|CTRC|CPO|CTRB2</td><td>-4.8</td><td>1.58489E-05</td></tr>
<tr><td>GO Biological Processes</td><td>19</td><td>GO:0007586</td><td>digestion</td><td>19_GO:0032501 multicellular organismal process</td><td>-8.2</td><td>6.30957E-09</td><td>41</td><td>15</td><td>30198</td><td>107</td><td>41</td><td>6</td><td>15</td><td>5.5</td><td>1208|5645|7032|56624|56667|440387</td><td>CLPS|PRSS2|TFF2|ASAH2|MUC13|CTRB2</td><td>-4.8</td><td>1.58489E-05</td></tr>
<tr><td>GO Molecular Functions</td><td>21</td><td>GO:0004181</td><td>metallocarboxypeptidase activity</td><td></td><td>-7.2</td><td>6.30957E-08</td><td>1.00E+02</td><td>20</td><td>30198</td><td>29</td><td>41</td><td>4</td><td>9.8</td><td>4.6</td><td>1357|1358|1360|130749</td><td>CPA1|CPA2|CPB1|CPO</td><td>-3.8</td><td>0.0002</td></tr>
<tr><td>GO Molecular Functions</td><td>21</td><td>GO:0048306</td><td>calcium-dependent protein binding</td><td></td><td>-6.9</td><td>1.25893E-07</td><td>43</td><td>14</td><td>30198</td><td>86</td><td>41</td><td>5</td><td>12</td><td>5.1</td><td>302|307|311|6279|6280</td><td>ANXA2|ANXA4|ANXA11|S100A8|S100A9</td><td>-3.6</td><td>0.0003</td></tr>
<tr><td>GO Biological Processes</td><td>19</td><td>GO:0002544</td><td>chronic inflammatory response</td><td>19_GO:0050896 response to stimulus</td><td>-6.9</td><td>1.25893E-07</td><td>2.80E+02</td><td>29</td><td>30198</td><td>8</td><td>41</td><td>3</td><td>7.3</td><td>4.1</td><td>6279|6280|8876</td><td>S100A8|S100A9|VNN1</td><td>-3.6</td><td>0.0003</td></tr>
<tr><td>Reactome Gene Sets</td><td>6</td><td>R-HSA-6803157</td><td>Antimicrobial peptides</td><td></td><td>-6.7</td><td>1.99526E-07</td><td>39</td><td>14</td><td>30198</td><td>95</td><td>41</td><td>5</td><td>12</td><td>5.1</td><td>4069|5645|6279|6280|55600</td><td>LYZ|PRSS2|S100A8|S100A9|ITLN1</td><td>-3.5</td><td>0.0003</td></tr>
<tr><td>GO Molecular Functions</td><td>21</td><td>GO:0004180</td><td>carboxypeptidase activity</td><td></td><td>-6.4</td><td>3.98107E-07</td><td>67</td><td>16</td><td>30198</td><td>44</td><td>41</td><td>4</td><td>9.8</td><td>4.6</td><td>1357|1358|1360|130749</td><td>CPA1|CPA2|CPB1|CPO</td><td>-3.2</td><td>0.0006</td></tr>
<tr><td>GO Biological Processes</td><td>19</td><td>GO:0051238</td><td>sequestering of metal ion</td><td>19_GO:0051179 localization</td><td>-6.3</td><td>5.01187E-07</td><td>1.80E+02</td><td>23</td><td>30198</td><td>12</td><td>41</td><td>3</td><td>7.3</td><td>4.1</td><td>6279|6280|6717</td><td>S100A8|S100A9|SRI</td><td>-3.1</td><td>0.0008</td></tr>
<tr><td>GO Molecular Functions</td><td>21</td><td>GO:0002020</td><td>protease binding</td><td></td><td>-5.9</td><td>1.25893E-06</td><td>27</td><td>11</td><td>30198</td><td>136</td><td>41</td><td>5</td><td>12</td><td>5.1</td><td>302|462|5269|6717|7273</td><td>ANXA2|SERPINC1|SERPINB6|SRI|TTN</td><td>-2.8</td><td>0.0016</td></tr>
<tr><td>Reactome Gene Sets</td><td>6</td><td>R-HSA-2022377</td><td>Metabolism of Angiotensinogen to Angiotensins</td><td></td><td>-5.7</td><td>1.99526E-06</td><td>1.20E+02</td><td>19</td><td>30198</td><td>18</td><td>41</td><td>3</td><td>7.3</td><td>4.1</td><td>290|1360|4311</td><td>ANPEP|CPB1|MME</td><td>-2.6</td><td>0.0025</td></tr>
</table>

Reactome Gene Sets	6	R-HSA-8935690	Digestion		-5.5	3.16228E-06	1.00E+02	17	30198	22	41	3	7.3	4.1	276|280|1208	AMY1A|AMY2B|CLPS	-2.3	0.0050
Reactome Gene Sets	6	R-HSA-8963743	Digestion and absorption		-5.2	6.30957E-06	82	15	30198	27	41	3	7.3	4.1	276|280|1208	AMY1A|AMY2B|CLPS	-2.1	0.0079
Hallmark Gene Sets	23	M5956	HALLMARK KRAS SIGNALING DN		-5.1	7.94328E-06	18	9.1	30198	200	41	5	12	5.1	1208|1358|1360|4625|7032	CLPS|CPA2|CPB1|MYH7|TFF2	-2	0.0100
GO Biological Processes	19	GO:0042742	defense response to bacterium	19_GO:0044419 biological process involved in interspecies interaction between organisms	-5.1	7.94328E-06	13	8.1	30198	351	41	6	15	5.5	4069|4353|5319|6279|6280|8288	LYZ|MPO|PLA2G1B|S100A8|S100A9|EPX	-2	0.0100
GO Biological Processes	19	GO:0097530	granulocyte migration	19_GO:0040011 locomotion	-5	0.00001	30	11	30198	97	41	4	9.8	4.6	5319|6279|6280|8288	PLA2G1B|S100A8|S100A9|EPX	-2	0.0100
GO Molecular Functions	21	GO:0008289	lipid binding		-5	0.00001	7.4	6.7	30198	797	41	8	20	6.2	302|307|311|347|1800|4311|6279|6280	ANXA2|ANXA4|ANXA11|APOD|DPEP1|MME|S100A8|S100A9	-2	0.0100
GO Molecular Functions	21	GO:0004857	enzyme inhibitor activity		-4.9	1.25893E-05	11	7.6	30198	390	41	6	15	5.5	302|307|462|1800|4869|5269	ANXA2|ANXA4|SERPINC1|DPEP1|NPM1|SERPINB6	-1.8	0.0158
GO Biological Processes	19	GO:0019730	antimicrobial humoral response	19_GO:0044419 biological process involved in interspecies interaction between organisms	-4.8	1.58489E-05	26	9.9	30198	112	41	4	9.8	4.6	4069|5319|5645|6280	LYZ|PLA2G1B|PRSS2|S100A9	-1.8	0.0158
GO Biological Processes	19	GO:0052548	regulation of endopeptidase activity	19_GO:0008152 metabolic process	-4.6	2.51189E-05	10	7.2	30198	426	41	6	15	5.5	302|462|1800|5269|6279|6280	ANXA2|SERPINC1|DPEP1|SERPINB6|S100A8|S100A9	-1.7	0.0200
GO Biological Processes	19	GO:0097529	myeloid leukocyte migration	19_GO:0040011 locomotion	-4.6	2.51189E-05	24	9.3	30198	125	41	4	9.8	4.6	5319|6279|6280|8288	PLA2G1B|S100A8|S100A9|EPX	-1.6	0.0251
GO Biological Processes	19	GO:0051090	regulation of DNA-binding transcription factor activity	19_GO:0008152 metabolic process	-4.5	3.16228E-05	9.8	6.9	30198	452	41	6	15	5.5	307|4869|5319|6279|6280|6717	ANXA4|NPM1|PLA2G1B|S100A8|S100A9|SRI	-1.5	0.0316
GO Biological Processes	19	GO:0052547	regulation of peptidase activity	19_GO:0008152 metabolic process	-4.5	3.16228E-05	9.7	6.9	30198	456	41	6	15	5.5	302|462|1800|5269|6279|6280	ANXA2|SERPINC1|DPEP1|SERPINB6|S100A8|S100A9	-1.5	0.0316
GO Biological Processes	19	GO:0050832	defense response to fungus	19_GO:0044419 biological process involved in interspecies interaction between organisms	-4.4	3.98107E-05	46	12	30198	48	41	3	7.3	4.1	4353|6279|6280	MPO|S100A8|S100A9	-1.5	0.0316
GO Biological Processes	19	GO:0051592	response to calcium ion	19_GO:0050896 response to stimulus	-4.4	3.98107E-05	20	8.6	30198	144	41	4	9.8	4.6	71|311|1800|7273	ACTG1|ANXA11|DPEP1|TTN	-1.4	0.0398
GO Biological Processes	19	GO:0009617	response to bacterium	19_GO:0044419 biological process involved in interspecies interaction between organisms	-4.3	5.01187E-05	7	6.1	30198	732	41	7	17	5.9	1208|4069|4353|5319|6279|6280|8288	CLPS|LYZ|MPO|PLA2G1B|S100A8|S100A9|EPX	-1.4	0.0398
GO Biological Processes	19	GO:0051047	positive regulation of secretion	19_GO:0051179 localization	-4.3	5.01187E-05	12	7.2	30198	303	41	5	12	5.1	302|5319|6279|6717|7273	ANXA2|PLA2G1B|S100A8|SRI|TTN	-1.4	0.0398
GO Molecular Functions	21	GO:0005544	calcium-dependent phospholipid binding		-4.3	5.01187E-05	41	11	30198	54	41	3	7.3	4.1	302|307|311	ANXA2|ANXA4|ANXA11	-1.4	0.0398
GO Biological Processes	19	GO:0051092	positive regulation of NF-kappaB transcription factor activity	19_GO:0008152 metabolic process	-4.2	6.30957E-05	19	8.3	30198	154	41	4	9.8	4.6	4869|5319|6279|6280	NPM1|PLA2G1B|S100A8|S100A9	-1.4	0.0398
GO Biological Processes	19	GO:0010035	response to inorganic substance	19_GO:0050896 response to stimulus	-4.2	6.30957E-05	8.5	6.4	30198	521	41	6	15	5.5	71|311|1800|4353|6279|7273	ACTG1|ANXA11|DPEP1|MPO|S100A8|TTN	-1.3	0.0501
GO Biological Processes	19	GO:0009620	response to fungus	19_GO:0044419 biological process involved in interspecies interaction between organisms	-4.1	7.94328E-05	37	10	30198	59	41	3	7.3	4.1	4353|6279|6280	MPO|S100A8|S100A9	-1.3	0.0501
GO Molecular Functions	21	GO:0004866	endopeptidase inhibitor activity		-4	0.0001	16	7.6	30198	180	41	4	9.8	4.6	302|462|1800|5269	ANXA2|SERPINC1|DPEP1|SERPINB6	-1.1	0.0794
GO Biological Processes	19	GO:0010038	response to metal ion	19_GO:0050896 response to stimulus	-4	0.0001	10	6.6	30198	351	41	5	12	5.1	71|311|1800|6279|7273	ACTG1|ANXA11|DPEP1|S100A8|TTN	-1.1	0.0794
GO Molecular Functions	21	GO:0030414	peptidase inhibitor activity		-3.9	0.000125893	16	7.5	30198	187	41	4	9.8	4.6	302|462|1800|5269	ANXA2|SERPINC1|DPEP1|SERPINB6	-1.1	0.0794
GO Molecular Functions	21	GO:0061135	endopeptidase regulator activity		-3.9	0.000125893	15	7.3	30198	194	41	4	9.8	4.6	302|462|1800|5269	ANXA2|SERPINC1|DPEP1|SERPINB6	-1.1	0.0794
GO Biological Processes	19	GO:0051046	regulation of secretion	19_GO:0051179 localization	-3.8	0.000158489	7.2	5.7	30198	610	41	6	15	5.5	302|5319|6279|6717|7032|7273	ANXA2|PLA2G1B|S100A8|SRI|TFF2|TTN	-0.99	0.1023
GO Biological Processes	19	GO:0030593	neutrophil chemotaxis	19_GO:0040011 locomotion	-3.8	0.000158489	28	8.9	30198	79	41	3	7.3	4.1	5319|6279|6280	PLA2G1B|S100A8|S100A9	-0.98	0.1047
GO Molecular Functions	21	GO:0033293	monocarboxylic acid binding		-3.7	0.000199526	27	8.7	30198	81	41	3	7.3	4.1	5319|6279|6280	PLA2G1B|S100A8|S100A9	-0.96	0.1096
GO Biological Processes	19	GO:0071621	granulocyte chemotaxis	19_GO:0040011 locomotion	-3.7	0.000199526	26	8.6	30198	84	41	3	7.3	4.1	5319|6279|6280	PLA2G1B|S100A8|S100A9	-0.93	0.1175
Canonical Pathways	11	M195	PID CMYB PATHWAY		-3.7	0.000199526	26	8.6	30198	84	41	3	7.3	4.1	290|4069|4353	ANPEP|LYZ|MPO	-0.93	0.1175

GO Molecular Functions	21	GO:0016209	antioxidant activity		-3.7	0.000199526	26	8.5	30198	85	41	3	7.3	4.1	4353|6280|8288	MPO|S100A9|EPX	-0.92	0.1202
GO Biological Processes	19	GO:1990266	neutrophil migration	19_GO:0040011 locomotion	-3.6	0.000251189	25	8.3	30198	89	41	3	7.3	4.1	5319|6279|6280	PLA2G1B|S100A8|S100A9	-0.89	0.1288
GO Biological Processes	19	GO:0098869	cellular oxidant detoxification	19_GO:0098754 detoxification	-3.6	0.000251189	25	8.3	30198	90	41	3	7.3	4.1	4353|6280|8288	MPO|S100A9|EPX	-0.89	0.1288
KEGG Pathway	24	hsa05410	Hypertrophic cardiomyopathy		-3.6	0.000251189	25	8.3	30198	90	41	3	7.3	4.1	71|4625|7273	ACTG1|MYH7|TTN	-0.89	0.1288
Reactome Gene Sets	6	R-HSA-2980736	Peptide hormone metabolism		-3.6	0.000251189	24	8.2	30198	91	41	3	7.3	4.1	290|1360|4311	ANPEP|CPB1|MME	-0.89	0.1288
GO Molecular Functions	21	GO:0061134	peptidase regulator activity		-3.6	0.000251189	13	6.6	30198	230	41	4	9.8	4.6	302|462|1800|5269	ANXA2|SERPINC1|DPEP1|SERPINB6	-0.89	0.1288
GO Biological Processes	19	GO:0050900	leukocyte migration	19_GO:0040011 locomotion	-3.6	0.000251189	13	6.6	30198	230	41	4	9.8	4.6	5319|6279|6280|8288	PLA2G1B|S100A8|S100A9|EPX	-0.89	0.1288
KEGG Pathway	24	hsa04970	Salivary secretion		-3.6	0.000251189	24	8.2	30198	92	41	3	7.3	4.1	276|280|4069	AMY1A|AMY2B|LYZ	-0.89	0.1288
GO Molecular Functions	21	GO:0072341	modified amino acid binding		-3.6	0.000251189	24	8.1	30198	93	41	3	7.3	4.1	302|1800|4311	ANXA2|DPEP1|MME	-0.89	0.1288
GO Biological Processes	19	GO:0051651	maintenance of location in cell	19_GO:0051179 localization	-3.6	0.000251189	24	8.1	30198	93	41	3	7.3	4.1	6279|6280|6717	S100A8|S100A9|SRI	-0.89	0.1288
GO Molecular Functions	21	GO:0004553	hydrolase activity, hydrolyzing O-glycosyl compounds		-3.5	0.000316228	24	8.1	30198	94	41	3	7.3	4.1	276|280|4069	AMY1A|AMY2B|LYZ	-0.89	0.1288
KEGG Pathway	24	hsa04657	IL-17 signaling pathway		-3.5	0.000316228	24	8.1	30198	94	41	3	7.3	4.1	4586|6279|6280	MUC5AC|S100A8|S100A9	-0.89	0.1288
GO Biological Processes	19	GO:0001894	tissue homeostasis	19_GO:0032501 multicellular organismal process	-3.5	0.000316228	13	6.5	30198	235	41	4	9.8	4.6	71|4069|7032|56667	ACTG1|LYZ|TFF2|MUC13	-0.89	0.1288
KEGG Pathway	24	hsa05414	Dilated cardiomyopathy		-3.5	0.000316228	23	8	30198	96	41	3	7.3	4.1	71|4625|7273	ACTG1|MYH7|TTN	-0.87	0.1349
GO Molecular Functions	21	GO:0004867	serine-type endopeptidase inhibitor activity		-3.5	0.000316228	23	7.9	30198	98	41	3	7.3	4.1	302|462|5269	ANXA2|SERPINC1|SERPINB6	-0.85	0.1413
GO Biological Processes	19	GO:0010951	negative regulation of endopeptidase activity	19_GO:0048519 negative regulation of biological process	-3.4	0.000398107	12	6.3	30198	251	41	4	9.8	4.6	302|462|1800|5269	ANXA2|SERPINC1|DPEP1|SERPINB6	-0.8	0.1585
GO Biological Processes	19	GO:1990748	cellular detoxification	19_GO:0098754 detoxification	-3.4	0.000398107	21	7.6	30198	105	41	3	7.3	4.1	4353|6280|8288	MPO|S100A9|EPX	-0.78	0.1660
GO Molecular Functions	21	GO:0005543	phospholipid binding		-3.4	0.000398107	7.9	5.5	30198	467	41	5	12	5.1	302|307|311|1800|4311	ANXA2|ANXA4|ANXA11|DPEP1|MME	-0.77	0.1698
GO Biological Processes	19	GO:0046916	cellular transition metal ion homeostasis	19_GO:0065007 biological regulation	-3.4	0.000398107	20	7.5	30198	108	41	3	7.3	4.1	6279|6280|6717	S100A8|S100A9|SRI	-0.75	0.1778
GO Biological Processes	19	GO:0010466	negative regulation of peptidase activity	19_GO:0048519 negative regulation of biological process	-3.4	0.000398107	11	6.1	30198	262	41	4	9.8	4.6	302|462|1800|5269	ANXA2|SERPINC1|DPEP1|SERPINB6	-0.75	0.1778
GO Biological Processes	19	GO:0051091	positive regulation of DNA-binding transcription factor activity	19_GO:0008152 metabolic process	-3.3	0.000501187	11	6.1	30198	265	41	4	9.8	4.6	4869|5319|6279|6280	NPM1|PLA2G1B|S100A8|S100A9	-0.74	0.1820
GO Biological Processes	19	GO:0030162	regulation of proteolysis	19_GO:0008152 metabolic process	-3.3	0.000501187	5.9	5	30198	745	41	6	15	5.5	302|462|1800|5269|6279|6280	ANXA2|SERPINC1|DPEP1|SERPINB6|S100A8|S100A9	-0.73	0.1862
GO Biological Processes	19	GO:0097237	cellular response to toxic substance	19_GO:0050896 response to stimulus	-3.3	0.000501187	20	7.3	30198	113	41	3	7.3	4.1	4353|6280|8288	MPO|S100A9|EPX	-0.72	0.1905
GO Biological Processes	19	GO:1903532	positive regulation of secretion by cell	19_GO:0051179 localization	-3.3	0.000501187	11	6	30198	276	41	4	9.8	4.6	302|5319|6717|7273	ANXA2|PLA2G1B|SRI|TTN	-0.69	0.2042
GO Biological Processes	19	GO:0060249	anatomical structure homeostasis	19_GO:0065007 biological regulation	-3.3	0.000501187	11	5.9	30198	279	41	4	9.8	4.6	71|4069|7032|56667	ACTG1|LYZ|TFF2|MUC13	-0.68	0.2089
GO Biological Processes	19	GO:0043086	negative regulation of catalytic activity	19_GO:0065007 biological regulation	-3.2	0.000630957	5.6	4.8	30198	786	41	6	15	5.5	302|307|462|1800|4869|5269	ANXA2|ANXA4|SERPINC1|DPEP1|NPM1|SERPINB6	-0.63	0.2344
GO Biological Processes	19	GO:0006959	humoral immune response	19_GO:0002376 immune system process	-3.2	0.000630957	10	5.8	30198	291	41	4	9.8	4.6	4069|5319|5645|6280	LYZ|PLA2G1B|PRSS2|S100A9	-0.63	0.2344
GO Molecular Functions	21	GO:0016810	hydrolase activity, acting on carbon-nitrogen (but not peptide) bonds		-3.2	0.000630957	17	6.8	30198	127	41	3	7.3	4.1	1800|8876|56624	DPEP1|VNN1|ASAH2	-0.6	0.2512
GO Biological Processes	19	GO:0050714	positive regulation of protein secretion	19_GO:0051179 localization	-3.2	0.000630957	17	6.8	30198	128	41	3	7.3	4.1	5319|6717|7273	PLA2G1B|SRI|TTN	-0.6	0.2512
GO Biological Processes	19	GO:0098754	detoxification	19_GO:0098754 detoxification	-3.1	0.000794328	17	6.7	30198	132	41	3	7.3	4.1	4353|6280|8288	MPO|S100A9|EPX	-0.57	0.2692
GO Biological Processes	19	GO:0098609	cell-cell adhesion	19_GO:0009987 cellular process	-3.1	0.000794328	6.9	5.1	30198	537	41	5	12	5.1	71|302|6279|6280|8876	ACTG1|ANXA2|S100A8|S100A9|VNN1	-0.57	0.2692
GO Biological Processes	19	GO:0055076	transition metal ion homeostasis	19_GO:0065007 biological regulation	-3.1	0.000794328	16	6.6	30198	136	41	3	7.3	4.1	6279|6280|6717	S100A8|S100A9|SRI	-0.54	0.2884
GO Biological Processes	19	GO:0006875	cellular metal ion homeostasis	19_GO:0065007 biological regulation	-3.1	0.000794328	9.3	5.5	30198	316	41	4	9.8	4.6	6279|6280|6717|11330	S100A8|S100A9|SRI|CTRC	-0.53	0.2951
GO Biological Processes	19	GO:0001525	angiogenesis	19_GO:0032502 developmental process	-3	0.001	9.1	5.4	30198	323	41	4	9.8	4.6	71|290|302|347	ACTG1|ANPEP|ANXA2|APOD	-0.5	0.3162
GO Molecular Functions	21	GO:0016798	hydrolase activity, acting on glycosyl bonds		-3	0.001	15	6.4	30198	144	41	3	7.3	4.1	276|280|4069	AMY1A|AMY2B|LYZ	-0.49	0.3236
Hallmark Gene Sets	23	M5942	HALLMARK UV RESPONSE DN		-3	0.001	15	6.4	30198	144	41	3	7.3	4.1	302|307|6717	ANXA2|ANXA4|SRI	-0.49	0.3236
Reactome Gene Sets	6	R-HSA-195258	RHO GTPase Effectors		-3	0.001	9	5.4	30198	327	41	4	9.8	4.6	71|6279|6280|8294	ACTG1|S100A8|S100A9|H4C9	-0.49	0.3236

<table>
<tr><th></th><th></th><th></th><th></th><th></th><th></th><th></th><th></th><th></th><th></th><th></th><th></th><th></th><th></th><th></th><th></th><th></th><th></th><th></th></tr>
<tr><td>GO Biological Processes</td><td>19</td><td>GO:0030595</td><td>leukocyte chemotaxis</td><td>19_GO:0040011 locomotion</td><td>-3</td><td>0.001</td><td>15</td><td>6.3</td><td>30198</td><td>145</td><td>41</td><td>3</td><td>7.3</td><td>4.1</td><td>5319|6279|6280</td><td>PLA2G1B|S100A8|S100A9</td><td>-0.49</td><td>0.3236</td></tr>
<tr><td>GO Biological Processes</td><td>19</td><td>GO:0045861</td><td>negative regulation of proteolysis</td><td>19_GO:0048519 negative regulation of biological process</td><td>-2.9</td><td>0.001258925</td><td>8.4</td><td>5.1</td><td>30198</td><td>352</td><td>41</td><td>4</td><td>9.8</td><td>4.6</td><td>302|462|1800|5269</td><td>ANXA2|SERPINC1|DPEP1|SERPINB6</td><td>-0.38</td><td>0.4169</td></tr>
<tr><td>GO Biological Processes</td><td>19</td><td>GO:0051235</td><td>maintenance of location</td><td>19_GO:0051179 localization</td><td>-2.9</td><td>0.001258925</td><td>14</td><td>6</td><td>30198</td><td>161</td><td>41</td><td>3</td><td>7.3</td><td>4.1</td><td>6279|6280|6717</td><td>S100A8|S100A9|SRI</td><td>-0.37</td><td>0.4266</td></tr>
<tr><td>GO Biological Processes</td><td>19</td><td>GO:0048871</td><td>multicellular organismal homeostasis</td><td>19_GO:0032501 multicellular organismal process</td><td>-2.8</td><td>0.001584893</td><td>8.1</td><td>5</td><td>30198</td><td>363</td><td>41</td><td>4</td><td>9.8</td><td>4.6</td><td>71|4069|7032|56667</td><td>ACTG1|LYZ|TFF2|MUC13</td><td>-0.35</td><td>0.4467</td></tr>
<tr><td>GO Biological Processes</td><td>19</td><td>GO:0007568</td><td>aging</td><td>19_GO:0032502 developmental process</td><td>-2.8</td><td>0.001584893</td><td>13</td><td>5.9</td><td>30198</td><td>165</td><td>41</td><td>3</td><td>7.3</td><td>4.1</td><td>347|4311|4353</td><td>APOD|MME|MPO</td><td>-0.35</td><td>0.4467</td></tr>
<tr><td>Reactome Gene Sets</td><td>6</td><td>R-HSA-109582</td><td>Hemostasis</td><td></td><td>-2.8</td><td>0.001584893</td><td>5.9</td><td>4.6</td><td>30198</td><td>621</td><td>41</td><td>5</td><td>12</td><td>5.1</td><td>302|462|5269|6717|7273</td><td>ANXA2|SERPINC1|SERPINB6|SRI|TTN</td><td>-0.35</td><td>0.4467</td></tr>
<tr><td>GO Biological Processes</td><td>19</td><td>GO:0006979</td><td>response to oxidative stress</td><td>19_GO:0050896 response to stimulus</td><td>-2.8</td><td>0.001584893</td><td>8.1</td><td>5</td><td>30198</td><td>365</td><td>41</td><td>4</td><td>9.8</td><td>4.6</td><td>347|4353|8288|8876</td><td>APOD|MPO|EPX|VNN1</td><td>-0.35</td><td>0.4467</td></tr>
<tr><td>GO Biological Processes</td><td>19</td><td>GO:0030307</td><td>positive regulation of cell growth</td><td>19_GO:0040007 growth</td><td>-2.8</td><td>0.001584893</td><td>13</td><td>5.8</td><td>30198</td><td>168</td><td>41</td><td>3</td><td>7.3</td><td>4.1</td><td>5645|6279|6280</td><td>PRSS2|S100A8|S100A9</td><td>-0.34</td><td>0.4571</td></tr>
<tr><td>GO Biological Processes</td><td>19</td><td>GO:2001233</td><td>regulation of apoptotic signaling pathway</td><td>19_GO:0023052 signaling</td><td>-2.8</td><td>0.001584893</td><td>8</td><td>5</td><td>30198</td><td>370</td><td>41</td><td>4</td><td>9.8</td><td>4.6</td><td>6279|6280|8876|56624</td><td>S100A8|S100A9|VNN1|ASAH2</td><td>-0.34</td><td>0.4571</td></tr>
<tr><td>GO Biological Processes</td><td>19</td><td>GO:0051346</td><td>negative regulation of hydrolase activity</td><td>19_GO:0065007 biological regulation</td><td>-2.8</td><td>0.001584893</td><td>7.9</td><td>5</td><td>30198</td><td>371</td><td>41</td><td>4</td><td>9.8</td><td>4.6</td><td>302|462|1800|5269</td><td>ANXA2|SERPINC1|DPEP1|SERPINB6</td><td>-0.34</td><td>0.4571</td></tr>
<tr><td>GO Biological Processes</td><td>19</td><td>GO:2001242</td><td>regulation of intrinsic apoptotic signaling pathway</td><td>19_GO:0023052 signaling</td><td>-2.8</td><td>0.001584893</td><td>13</td><td>5.8</td><td>30198</td><td>171</td><td>41</td><td>3</td><td>7.3</td><td>4.1</td><td>6279|6280|8876</td><td>S100A8|S100A9|VNN1</td><td>-0.33</td><td>0.4677</td></tr>
<tr><td>GO Molecular Functions</td><td>21</td><td>GO:0031406</td><td>carboxylic acid binding</td><td></td><td>-2.8</td><td>0.001584893</td><td>13</td><td>5.7</td><td>30198</td><td>173</td><td>41</td><td>3</td><td>7.3</td><td>4.1</td><td>5319|6279|6280</td><td>PLA2G1B|S100A8|S100A9</td><td>-0.32</td><td>0.4786</td></tr>
<tr><td>GO Molecular Functions</td><td>21</td><td>GO:0004252</td><td>serine-type endopeptidase activity</td><td></td><td>-2.8</td><td>0.001584893</td><td>13</td><td>5.7</td><td>30198</td><td>174</td><td>41</td><td>3</td><td>7.3</td><td>4.1</td><td>5645|11330|440387</td><td>PRSS2|CTRC|CTRB2</td><td>-0.32</td><td>0.4786</td></tr>
<tr><td>GO Biological Processes</td><td>19</td><td>GO:0030003</td><td>cellular cation homeostasis</td><td>19_GO:0065007 biological regulation</td><td>-2.7</td><td>0.001995262</td><td>7.5</td><td>4.8</td><td>30198</td><td>394</td><td>41</td><td>4</td><td>9.8</td><td>4.6</td><td>6279|6280|6717|11330</td><td>S100A8|S100A9|SRI|CTRC</td><td>-0.26</td><td>0.5495</td></tr>
<tr><td>GO Biological Processes</td><td>19</td><td>GO:0055065</td><td>metal ion homeostasis</td><td>19_GO:0065007 biological regulation</td><td>-2.7</td><td>0.001995262</td><td>7.4</td><td>4.7</td><td>30198</td><td>399</td><td>41</td><td>4</td><td>9.8</td><td>4.6</td><td>6279|6280|6717|11330</td><td>S100A8|S100A9|SRI|CTRC</td><td>-0.25</td><td>0.5623</td></tr>
<tr><td>Reactome Gene Sets</td><td>6</td><td>R-HSA-196854</td><td>Metabolism of vitamins and cofactors</td><td></td><td>-2.7</td><td>0.001995262</td><td>12</td><td>5.4</td><td>30198</td><td>190</td><td>41</td><td>3</td><td>7.3</td><td>4.1</td><td>1208|8876|440387</td><td>CLPS|VNN1|CTRB2</td><td>-0.23</td><td>0.5888</td></tr>
<tr><td>KEGG Pathway</td><td>24</td><td>hsa04613</td><td>Neutrophil extracellular trap formation</td><td></td><td>-2.7</td><td>0.001995262</td><td>12</td><td>5.4</td><td>30198</td><td>190</td><td>41</td><td>3</td><td>7.3</td><td>4.1</td><td>71|4353|8294</td><td>ACTG1|MPO|H4C9</td><td>-0.23</td><td>0.5888</td></tr>
<tr><td>GO Molecular Functions</td><td>21</td><td>GO:0008236</td><td>serine-type peptidase activity</td><td></td><td>-2.6</td><td>0.002511886</td><td>12</td><td>5.4</td><td>30198</td><td>192</td><td>41</td><td>3</td><td>7.3</td><td>4.1</td><td>5645|11330|440387</td><td>PRSS2|CTRC|CTRB2</td><td>-0.22</td><td>0.6026</td></tr>
<tr><td>GO Biological Processes</td><td>19</td><td>GO:0006873</td><td>cellular ion homeostasis</td><td>19_GO:0065007 biological regulation</td><td>-2.6</td><td>0.002511886</td><td>7.2</td><td>4.7</td><td>30198</td><td>409</td><td>41</td><td>4</td><td>9.8</td><td>4.6</td><td>6279|6280|6717|11330</td><td>S100A8|S100A9|SRI|CTRC</td><td>-0.22</td><td>0.6026</td></tr>
<tr><td>GO Molecular Functions</td><td>21</td><td>GO:0017171</td><td>serine hydrolase activity</td><td></td><td>-2.6</td><td>0.002511886</td><td>11</td><td>5.3</td><td>30198</td><td>196</td><td>41</td><td>3</td><td>7.3</td><td>4.1</td><td>5645|11330|440387</td><td>PRSS2|CTRC|CTRB2</td><td>-0.2</td><td>0.6310</td></tr>
<tr><td>Reactome Gene Sets</td><td>6</td><td>R-HSA-397014</td><td>Muscle contraction</td><td></td><td>-2.6</td><td>0.002511886</td><td>11</td><td>5.3</td><td>30198</td><td>197</td><td>41</td><td>3</td><td>7.3</td><td>4.1</td><td>302|6717|7273</td><td>ANXA2|SRI|TTN</td><td>-0.2</td><td>0.6310</td></tr>
<tr><td>Hallmark Gene Sets</td><td>23</td><td>M5947</td><td>HALLMARK IL2 STAT5 SIGNALING</td><td></td><td>-2.6</td><td>0.002511886</td><td>11</td><td>5.3</td><td>30198</td><td>199</td><td>41</td><td>3</td><td>7.3</td><td>4.1</td><td>307|462|5269</td><td>ANXA4|SERPINC1|SERPINB6</td><td>-0.2</td><td>0.6310</td></tr>
<tr><td>GO Biological Processes</td><td>19</td><td>GO:0048514</td><td>blood vessel morphogenesis</td><td>19_GO:0032502 developmental process</td><td>-2.6</td><td>0.002511886</td><td>7</td><td>4.6</td><td>30198</td><td>421</td><td>41</td><td>4</td><td>9.8</td><td>4.6</td><td>71|290|302|347</td><td>ACTG1|ANPEP|ANXA2|APOD</td><td>-0.2</td><td>0.6310</td></tr>
<tr><td>GO Molecular Functions</td><td>21</td><td>GO:0005516</td><td>calmodulin binding</td><td></td><td>-2.6</td><td>0.002511886</td><td>11</td><td>5.3</td><td>30198</td><td>200</td><td>41</td><td>3</td><td>7.3</td><td>4.1</td><td>4619|4625|7273</td><td>MYH1|MYH7|TTN</td><td>-0.2</td><td>0.6310</td></tr>
<tr><td>Hallmark Gene Sets</td><td>23</td><td>M5909</td><td>HALLMARK MYOGENESIS</td><td></td><td>-2.6</td><td>0.002511886</td><td>11</td><td>5.3</td><td>30198</td><td>200</td><td>41</td><td>3</td><td>7.3</td><td>4.1</td><td>347|4619|4625</td><td>APOD|MYH1|MYH7</td><td>-0.2</td><td>0.6310</td></tr>
<tr><td>Reactome Gene Sets</td><td>6</td><td>R-HSA-194315</td><td>Signaling by Rho GTPases</td><td></td><td>-2.6</td><td>0.002511886</td><td>5.2</td><td>4.2</td><td>30198</td><td>707</td><td>41</td><td>5</td><td>12</td><td>5.1</td><td>71|6279|6280|8294|56667</td><td>ACTG1|S100A8|S100A9|H4C9|MUC13</td><td>-0.19</td><td>0.6457</td></tr>
<tr><td>GO Biological Processes</td><td>19</td><td>GO:0009612</td><td>response to mechanical stimulus</td><td>19_GO:0050896 response to stimulus</td><td>-2.6</td><td>0.002511886</td><td>11</td><td>5.2</td><td>30198</td><td>203</td><td>41</td><td>3</td><td>7.3</td><td>4.1</td><td>71|4353|7273</td><td>ACTG1|MPO|TTN</td><td>-0.19</td><td>0.6457</td></tr>
<tr><td>GO Biological Processes</td><td>19</td><td>GO:0043281</td><td>regulation of cysteine-type endopeptidase activity involved in apoptotic process</td><td>19_GO:0008152 metabolic process</td><td>-2.6</td><td>0.002511886</td><td>11</td><td>5.2</td><td>30198</td><td>204</td><td>41</td><td>3</td><td>7.3</td><td>4.1</td><td>1800|6279|6280</td><td>DPEP1|S100A8|S100A9</td><td>-0.19</td><td>0.6457</td></tr>
<tr><td>GO Molecular Functions</td><td>21</td><td>GO:0004175</td><td>endopeptidase activity</td><td></td><td>-2.6</td><td>0.002511886</td><td>6.8</td><td>4.5</td><td>30198</td><td>433</td><td>41</td><td>4</td><td>9.8</td><td>4.6</td><td>4311|5645|11330|440387</td><td>MME|PRSS2|CTRC|CTRB2</td><td>-0.18</td><td>0.6607</td></tr>
<tr><td>GO Biological Processes</td><td>19</td><td>GO:0060326</td><td>cell chemotaxis</td><td>19_GO:0040011 locomotion</td><td>-2.6</td><td>0.002511886</td><td>11</td><td>5.1</td><td>30198</td><td>207</td><td>41</td><td>3</td><td>7.3</td><td>4.1</td><td>5319|6279|6280</td><td>PLA2G1B|S100A8|S100A9</td><td>-0.18</td><td>0.6607</td></tr>
<tr><td>GO Biological Processes</td><td>19</td><td>GO:0009611</td><td>response to wounding</td><td>19_GO:0050896 response to stimulus</td><td>-2.5</td><td>0.003162278</td><td>6.8</td><td>4.5</td><td>30198</td><td>435</td><td>41</td><td>4</td><td>9.8</td><td>4.6</td><td>71|347|462|6279</td><td>ACTG1|APOD|SERPINC1|S100A8</td><td>-0.17</td><td>0.6761</td></tr>
<tr><td>Reactome Gene Sets</td><td>6</td><td>R-HSA-9716542</td><td>Signaling by Rho GTPases, Miro GTPases and RHOBTB3</td><td></td><td>-2.5</td><td>0.003162278</td><td>5.1</td><td>4.1</td><td>30198</td><td>723</td><td>41</td><td>5</td><td>12</td><td>5.1</td><td>71|6279|6280|8294|56667</td><td>ACTG1|S100A8|S100A9|H4C9|MUC13</td><td>-0.17</td><td>0.6761</td></tr>
<tr><td>GO Molecular Functions</td><td>21</td><td>GO:0051015</td><td>actin filament binding</td><td></td><td>-2.5</td><td>0.003162278</td><td>10</td><td>5</td><td>30198</td><td>215</td><td>41</td><td>3</td><td>7.3</td><td>4.1</td><td>4619|4625|7273</td><td>MYH1|MYH7|TTN</td><td>-0.14</td><td>0.7244</td></tr>
<tr><td>GO Biological Processes</td><td>19</td><td>GO:0072503</td><td>cellular divalent inorganic cation homeostasis</td><td>19_GO:0065007 biological regulation</td><td>-2.5</td><td>0.003162278</td><td>10</td><td>5</td><td>30198</td><td>220</td><td>41</td><td>3</td><td>7.3</td><td>4.1</td><td>6279|6280|11330</td><td>S100A8|S100A9|CTRC</td><td>-0.12</td><td>0.7586</td></tr>
<tr><td>GO Biological Processes</td><td>19</td><td>GO:0006936</td><td>muscle contraction</td><td>19_GO:0032501 multicellular organismal process</td><td>-2.4</td><td>0.003981072</td><td>9.7</td><td>4.9</td><td>30198</td><td>228</td><td>41</td><td>3</td><td>7.3</td><td>4.1</td><td>4619|4625|7273</td><td>MYH1|MYH7|TTN</td><td>-0.082</td><td>0.8279</td></tr>
<tr><td>GO Biological Processes</td><td>19</td><td>GO:0034764</td><td>positive regulation of transmembrane transport</td><td>19_GO:0051179 localization</td><td>-2.4</td><td>0.003981072</td><td>9.7</td><td>4.9</td><td>30198</td><td>228</td><td>41</td><td>3</td><td>7.3</td><td>4.1</td><td>5319|6717|55600</td><td>PLA2G1B|SRI|ITLN1</td><td>-0.082</td><td>0.8279</td></tr>
<tr><td>GO Biological Processes</td><td>19</td><td>GO:2000116</td><td>regulation of cysteine-type endopeptidase activity</td><td>19_GO:0008152 metabolic process</td><td>-2.4</td><td>0.003981072</td><td>9.6</td><td>4.8</td><td>30198</td><td>229</td><td>41</td><td>3</td><td>7.3</td><td>4.1</td><td>1800|6279|6280</td><td>DPEP1|S100A8|S100A9</td><td>-0.082</td><td>0.8279</td></tr>
</table>

GO Biological Processes	19	GO:0009636	response to toxic substance	19_GO:0050896 response to stimulus	-2.4	0.003981072	9.6	4.8	30198	229	41	3	7.3	4.1	4353|6280|8288	MPO|S100A9|EPX	-0.082	0.8279
GO Biological Processes	19	GO:0051051	negative regulation of transport	19_GO:0051179 localization	-2.4	0.003981072	6.2	4.2	30198	474	41	4	9.8	4.6	302|347|6717|7032	ANXA2|APOD|SRI|TFF2	-0.071	0.8492
GO Biological Processes	19	GO:0061061	muscle structure development	19_GO:0032502 developmental process	-2.4	0.003981072	6.1	4.2	30198	484	41	4	9.8	4.6	71|4625|6717|7273	ACTG1|MYH7|SRI|TTN	-0.046	0.8995
Canonical Pathways	11	M3468	NABA ECM REGULATORS		-2.4	0.003981072	9.3	4.7	30198	238	41	3	7.3	4.1	462|5269|5645	SERPINC1|SERPINB6|PRSS2	-0.046	0.8995
GO Biological Processes	19	GO:0055080	cation homeostasis	19_GO:0065007 biological regulation	-2.4	0.003981072	6.1	4.2	30198	485	41	4	9.8	4.6	6279|6280|6717|11330	S100A8|S100A9|SRI|CTRC	-0.046	0.8995
GO Biological Processes	19	GO:0043603	cellular amide metabolic process	19_GO:0008152 metabolic process	-2.4	0.003981072	4.6	3.8	30198	792	41	5	12	5.1	290|1800|4311|8876|56624	ANPEP|DPEP1|MME|VNN1|ASAH2	-0.045	0.9016
GO Biological Processes	19	GO:0098771	inorganic ion homeostasis	19_GO:0065007 biological regulation	-2.4	0.003981072	6	4.1	30198	494	41	4	9.8	4.6	6279|6280|6717|11330	S100A8|S100A9|SRI|CTRC	-0.025	0.9441
GO Biological Processes	19	GO:0051223	regulation of protein transport	19_GO:0051179 localization	-2.3	0.005011872	6	4.1	30198	495	41	4	9.8	4.6	347|5319|6717|7273	APOD|PLA2G1B|SRI|TTN	-0.025	0.9441
GO Biological Processes	19	GO:0001568	blood vessel development	19_GO:0032502 developmental process	-2.3	0.005011872	5.8	4	30198	505	41	4	9.8	4.6	71|290|302|347	ACTG1|ANPEP|ANXA2|APOD	0	1.0000
GO Biological Processes	19	GO:0050708	regulation of protein secretion	19_GO:0051179 localization	-2.3	0.005011872	8.8	4.6	30198	252	41	3	7.3	4.1	5319|6717|7273	PLA2G1B|SRI|TTN	0	1.0000
GO Biological Processes	19	GO:0072507	divalent inorganic cation homeostasis	19_GO:0065007 biological regulation	-2.3	0.005011872	8.8	4.6	30198	252	41	3	7.3	4.1	6279|6280|11330	S100A8|S100A9|CTRC	0	1.0000
GO Biological Processes	19	GO:0050801	ion homeostasis	19_GO:0065007 biological regulation	-2.3	0.005011872	5.8	4	30198	511	41	4	9.8	4.6	6279|6280|6717|11330	S100A8|S100A9|SRI|CTRC	0	1.0000
GO Biological Processes	19	GO:0070201	regulation of establishment of protein localization	19_GO:0051179 localization	-2.3	0.005011872	5.7	4	30198	520	41	4	9.8	4.6	347|5319|6717|7273	APOD|PLA2G1B|SRI|TTN	0	1.0000
GO Biological Processes	19	GO:0045927	positive regulation of growth	19_GO:0040007 growth	-2.3	0.005011872	8.5	4.5	30198	261	41	3	7.3	4.1	5645|6279|6280	PRSS2|S100A8|S100A9	0	1.0000
GO Biological Processes	19	GO:0055082	cellular chemical homeostasis	19_GO:0065007 biological regulation	-2.3	0.005011872	5.7	4	30198	521	41	4	9.8	4.6	6279|6280|6717|11330	S100A8|S100A9|SRI|CTRC	0	1.0000
GO Biological Processes	19	GO:0006954	inflammatory response	19_GO:0050896 response to stimulus	-2.3	0.005011872	5.6	3.9	30198	523	41	4	9.8	4.6	4069|6279|6280|8876	LYZ|S100A8|S100A9|VNN1	0	1.0000
GO Biological Processes	19	GO:0001944	vasculature development	19_GO:0032502 developmental process	-2.3	0.005011872	5.6	3.9	30198	526	41	4	9.8	4.6	71|290|302|347	ACTG1|ANPEP|ANXA2|APOD	0	1.0000
GO Biological Processes	19	GO:0051493	regulation of cytoskeleton organization	19_GO:0050789 regulation of biological process	-2.2	0.006309573	5.5	3.9	30198	531	41	4	9.8	4.6	71|4869|6279|6280	ACTG1|NPM1|S100A8|S100A9	0	1.0000
GO Molecular Functions	21	GO:0030246	carbohydrate binding		-2.2	0.006309573	8.2	4.4	30198	270	41	3	7.3	4.1	3960|55600|142683	LGALS4|ITLN1|ITLN2	0	1.0000
GO Biological Processes	19	GO:0003012	muscle system process	19_GO:0032501 multicellular organismal process	-2.2	0.006309573	7.8	4.3	30198	282	41	3	7.3	4.1	4619|4625|7273	MYH1|MYH7|TTN	0	1.0000
GO Biological Processes	19	GO:1903530	regulation of secretion by cell	19_GO:0051179 localization	-2.2	0.006309573	5.3	3.8	30198	554	41	4	9.8	4.6	302|5319|6717|7273	ANXA2|PLA2G1B|SRI|TTN	0	1.0000
GO Biological Processes	19	GO:0016042	lipid catabolic process	19_GO:0008152 metabolic process	-2.2	0.006309573	7.8	4.2	30198	283	41	3	7.3	4.1	1208|5319|56624	CLPS|PLA2G1B|ASAH2	0	1.0000
GO Biological Processes	19	GO:0007517	muscle organ development	19_GO:0032502 developmental process	-2.1	0.007943282	7.5	4.1	30198	293	41	3	7.3	4.1	4625|6717|7273	MYH7|SRI|TTN	0	1.0000
GO Biological Processes	19	GO:0051222	positive regulation of protein transport	19_GO:0051179 localization	-2.1	0.007943282	7.5	4.1	30198	293	41	3	7.3	4.1	5319|6717|7273	PLA2G1B|SRI|TTN	0	1.0000
GO Biological Processes	19	GO:1904951	positive regulation of establishment of protein localization	19_GO:0051179 localization	-2.1	0.007943282	7.2	4	30198	309	41	3	7.3	4.1	5319|6717|7273	PLA2G1B|SRI|TTN	0	1.0000
GO Biological Processes	19	GO:0140694	non-membrane-bounded organelle assembly	19_GO:0009987 cellular process	-2.1	0.007943282	7.1	4	30198	312	41	3	7.3	4.1	71|4869|7273	ACTG1|NPM1|TTN	0	1.0000
GO Biological Processes	19	GO:0030029	actin filament-based process	19_GO:0009987 cellular process	-2.1	0.007943282	4.9	3.6	30198	601	41	4	9.8	4.6	71|4625|5319|7273	ACTG1|MYH7|PLA2G1B|TTN	0	1.0000
GO Biological Processes	19	GO:0104004	cellular response to environmental stimulus	19_GO:0050896 response to stimulus	-2	0.01	7	4	30198	315	41	3	7.3	4.1	4311|4869|5269	MME|NPM1|SERPINB6	0	1.0000
GO Biological Processes	19	GO:0071214	cellular response to abiotic stimulus	19_GO:0050896 response to stimulus	-2	0.01	7	4	30198	315	41	3	7.3	4.1	4311|4869|5269	MME|NPM1|SERPINB6	0	1.0000
GO Biological Processes	19	GO:0032496	response to lipopolysaccharide	19_GO:0044419 biological process involved in interspecies interaction between organisms	-2	0.01	6.9	3.9	30198	318	41	3	7.3	4.1	4353|6279|6280	MPO|S100A8|S100A9	0	1.0000
GO Biological Processes	19	GO:0019725	cellular homeostasis	19_GO:0065007 biological regulation	-2	0.01	4.7	3.5	30198	621	41	4	9.8	4.6	6279|6280|6717|11330	S100A8|S100A9|SRI|CTRC	0	1.0000

7 differentially expressed human PGs, Enriched terms

<table>
<tr><th>Category</th><th>CategoryID</th><th>GO</th><th>Description</th><th>PARENT_GO</th><th>LogP</th><th>P-value</th><th>Enrichment</th><th>Z-score</th><th>#TotalGeneInLibrary</th><th>#GeneInGO</th><th>#GeneInHitList</th><th>#GeneInGOAndHitList</th><th>%InGO</th><th>STDV %InGO</th><th>GeneID</th><th>Hits</th><th>Log (q-value)</th><th>q-value</th></tr>
<tr><td>Reactome Gene Sets</td><td>6</td><td>R-HSA-6798695</td><td>Neutrophil degranulation</td><td></td><td>-5.7</td><td>1.99526E-06</td><td>36</td><td>12</td><td>30198</td><td>482</td><td>7</td><td>4</td><td>57</td><td>19</td><td>290|302|6279|8876</td><td>ANPEP|ANXA2|S100A8|VNN1</td><td>-1.3</td><td>0.050118723</td></tr>
<tr><td>GO Biological Processes</td><td>19</td><td>GO:0052548</td><td>regulation of endopeptidase activity</td><td>19_GO:0008152 metabolic process</td><td>-4</td><td>0.0001</td><td>30</td><td>9.3</td><td>30198</td><td>426</td><td>7</td><td>3</td><td>43</td><td>19</td><td>302|1800|6279</td><td>ANXA2|DPEP1|S100A8</td><td>-0.072</td><td>0.847227414</td></tr>
<tr><td>GO Biological Processes</td><td>19</td><td>GO:0052547</td><td>regulation of peptidase activity</td><td>19_GO:0008152 metabolic process</td><td>-3.9</td><td>0.000125893</td><td>28</td><td>9</td><td>30198</td><td>456</td><td>7</td><td>3</td><td>43</td><td>19</td><td>302|1800|6279</td><td>ANXA2|DPEP1|S100A8</td><td>-0.072</td><td>0.847227414</td></tr>
<tr><td>GO Biological Processes</td><td>19</td><td>GO:0098609</td><td>cell-cell adhesion</td><td>19_GO:0009987 cellular process</td><td>-3.7</td><td>0.000199526</td><td>24</td><td>8.2</td><td>30198</td><td>537</td><td>7</td><td>3</td><td>43</td><td>19</td><td>302|6279|8876</td><td>ANXA2|S100A8|VNN1</td><td>0</td><td>1</td></tr>
<tr><td>GO Biological Processes</td><td>19</td><td>GO:0051046</td><td>regulation of secretion</td><td>19_GO:0051179 localization</td><td>-3.6</td><td>0.000251189</td><td>21</td><td>7.7</td><td>30198</td><td>610</td><td>7</td><td>3</td><td>43</td><td>19</td><td>302|6279|7032</td><td>ANXA2|S100A8|TFF2</td><td>0</td><td>1</td></tr>
<tr><td>GO Biological Processes</td><td>19</td><td>GO:0030162</td><td>regulation of proteolysis</td><td>19_GO:0008152 metabolic process</td><td>-3.3</td><td>0.000501187</td><td>17</td><td>6.9</td><td>30198</td><td>745</td><td>7</td><td>3</td><td>43</td><td>19</td><td>302|1800|6279</td><td>ANXA2|DPEP1|S100A8</td><td>0</td><td>1</td></tr>
<tr><td>Canonical Pathways</td><td>11</td><td>M5885</td><td>NABA MATRISOME ASSOCIATED</td><td></td><td>-3.3</td><td>0.000501187</td><td>17</td><td>6.9</td><td>30198</td><td>751</td><td>7</td><td>3</td><td>43</td><td>19</td><td>302|307|6279</td><td>ANXA2|ANXA4|S100A8</td><td>0</td><td>1</td></tr>
<tr><td>GO Biological Processes</td><td>19</td><td>GO:0043086</td><td>negative regulation of catalytic activity</td><td>19_GO:0065007 biological regulation</td><td>-3.2</td><td>0.000630957</td><td>16</td><td>6.7</td><td>30198</td><td>786</td><td>7</td><td>3</td><td>43</td><td>19</td><td>302|307|1800</td><td>ANXA2|ANXA4|DPEP1</td><td>0</td><td>1</td></tr>
<tr><td>GO Biological Processes</td><td>19</td><td>GO:0043603</td><td>cellular amide metabolic process</td><td>19_GO:0008152 metabolic process</td><td>-3.2</td><td>0.000630957</td><td>16</td><td>6.7</td><td>30198</td><td>792</td><td>7</td><td>3</td><td>43</td><td>19</td><td>290|1800|8876</td><td>ANPEP|DPEP1|VNN1</td><td>0</td><td>1</td></tr>
</table>
